# Structural Requirements for Ga^3+^ Coordination in Synthetic Analogues of the Siderophore Piscibactin Deduced by Chemical Synthesis and Density Functional Theory Calculations

**DOI:** 10.1021/acs.inorgchem.3c00787

**Published:** 2023-05-04

**Authors:** M. Carmen de la Fuente, Lucía Ageitos, Marta A. Lages, Diana Martínez-Matamoros, Abel M. Forero, Miguel Balado, Manuel L. Lemos, Jaime Rodríguez, Carlos Jiménez

**Affiliations:** † CICA—Centro Interdisciplinar de Química e Bioloxía, Departamento de Química, Facultade de Ciencias, Universidade da Coruña, A Coruña 15071, Spain; ‡ Departamento de Microbiología y Parasitología, Instituto de Acuicultura, Universidade de Santiago de Compostela, Santiago de Compostela 15782, Spain

## Abstract

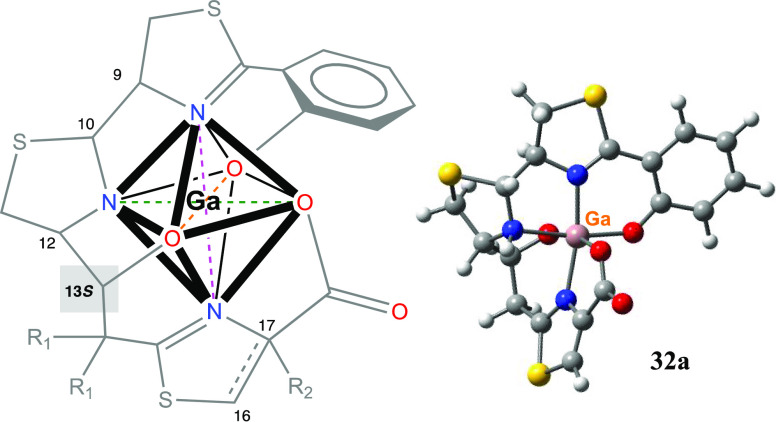

Stereoselective total synthesis of several analogues of piscibactin (Pcb), the siderophore produced by different pathogenic Gram-negative bacteria, was performed. The acid-sensitive α**-**methylthiazoline moiety was replaced by a more stable thiazole ring, differing in the configuration of the OH group at the C-13 position. The ability of these Pcb analogues to form complexes with Ga^3+^ as a mimic of Fe^3+^ showed that the configuration of the hydroxyl group at C-13 as 13*S* is crucial for the chelation of Ga^3+^ to preserve the metal coordination, while the presence of a thiazole ring instead of the α**-**methylthiazoline moiety does not affect such coordination. A complete ^1^H and ^13^C NMR chemical shift assignment of the diastereoisomer mixtures around C9/C10 was done for diagnostic stereochemical disposition. Additionally, density functional theory calculations were performed not only for confirming the stereochemistry of the Ga^3+^ complex among the six possible diastereoisomers but also for deducing the ability of these to form octahedral coordination spheres with gallium. Finally, the lack of antimicrobial activity of Pcb and Pcb thiazole analogue Ga^3+^ complexes against *Vibrio anguillarum* agrees with one of the roles of siderophores in protecting pathogens from metal ion toxicity. The efficient metal coordination shown by this scaffold suggests its possible use as a starting point for the design of new chelating agents or vectors for the development of new antibacterials that exploit the “Trojan horse” strategy using the microbial iron uptake mechanisms. The results obtained will be of great help in the development of biotechnological applications for these types of compounds.

## Abstract 2

Stereoselective total synthesis of several analogues of piscibactin (Pcb), the siderophore produced by several pathogenic Gram-negative bacteria, was performed.Keywordspiscibactin; siderophore; Ga^3+^ complex; DFT; *Vibrio anguillarum*; Trojan horse strategy

## Introduction

Iron is an essential nutrient crucial for a wide variety of cellular processes. Although iron is one of the most abundant elements on the Earth’s crust, its bioavailability is very low. While most bacteria require 10^–6^ M (cytoplasmic concentration) for growth, the concentration of free iron in oceans is estimated to be 0.01–2 × 10^–9^ M^1^ and in human serum 10^–24^ M.^2^ The secretion of siderophores is a common mechanism developed by bacteria to overcome iron shortages. Siderophores are low-molecular-weight compounds that scavenge iron by forming soluble Fe^3+^ complexes that are acquired by bacteria via specific transporters.^3,4^ Siderophores can also exhibit other biological functions^5^ (interfering with quorum sensing regulation,^6^ displaying antimicrobial properties,^7^ metal detoxification,^8^ etc.), and so they play an important role in directly shaping the microbial community.^9^ Notably, siderophores have shown a wide range of applications in medicine,^10,11^ biotechnology,^12^ bioremediation,^13^ molecular imaging,^14^ and plant growth enhancement,^15^ among others. For example, the conjugation of siderophores with antibiotics using the “Trojan horse strategy” significantly increases the antimicrobial activity and specificity of the conjugated compound against those bacteria that are able to use the siderophore.^16,17^ A catechol-substituted siderophore conjugated with a cephalosporin, cefiderocol (**1**), was the first approved drug using this strategy for the treatment of infections in humans caused by aerobic Gram-negative bacteria.^18^

**Figure 1:**
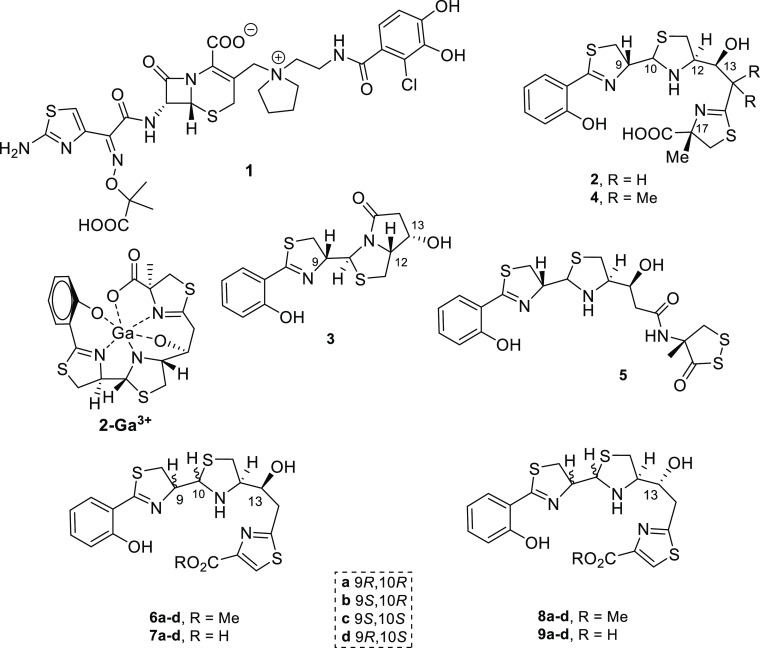
Structures of cefiderecol (**1**), piscibactin (**2**), prepiscibactin (**3**), yersiniabactin (**4**), photoxenobactin C (**5**), and the thiazole-Pcb analogues **6–9** synthetized in this work.

In a previous work, we reported the isolation and characterization of piscibactin (Pcb, **2**)^19^ as the siderophore responsible for iron uptake in the fish pathogenic Gram-negative bacterium *Photobacterium damselae* (*P. damselae*) subsp. *piscicida*. Pcb was characterized as its Ga^3+^ complex due to the low stability of its apo form. An intermediate of the biosynthesis of Pcb, prepiscibactin (PrePcb, **3**), was also isolated from the same bacterium.^20^ The structure of Pcb (**2**) is very similar to that of yersiniabactin (**4**), the siderophore involved in the iron uptake of some *Yersinia* species such as *Y. pestis*, responsible for the bubonic plague or Black Death, and *Y. enterocolitica*, the causative agent of severe enteric disorders in humans.^21^ Notably, the Pcb system is widespread among bacteria from the *Vibrionaceae* family, including potential human pathogens.^22^ In addition, Pcb was shown to be an important virulence factor of some fish pathogens such as the above mentioned *P. damselae* subsp. *piscicida* and *Vibrio anguillarum* (*V. anguillarum*), two of the most relevant bacterial pathogens in fish aquaculture worldwide.^23,24^ It has also been reported as a key virulence factor of some *Vibrio* species pathogenic for bivalve mollusks.^25^ Pcb (**2**) and PrePcb (**3**), along with several analogues of Pcb named as photoxenobactins A–E (see photoxenobactin C (**5**) in Figure 1), were reported recently from *Xenorhabdus szentirmaii* DSM 16338. Some of these compounds were associated with the insecticidal activity of *Xenorhabdus*.^26,27^

The efficient metal coordination displayed by this scaffold suggests its application as a starting point for the design of new chelating agents or to rationally design vectors that exploit the “Trojan horse” strategy. However, the high instability of these types of structures, probably to avoid their use as xenosiderophores by other competing organisms from the same environment,^28^ hinders its future applications. For this reason, to design stable analogues of Pcb, the structural requirements for metal coordination must be characterized. Ga^3+^ is an ideal metal model for the exploration of siderophore complexes due to its close mimicry with Fe^3+^, sharing various physicochemical characteristics, and its diamagnetism that allows the study of Ga^3+^ complexes by nuclear magnetic resonance (NMR).

Herein, we report the total synthesis of several Pcb analogues where the α**-**methylthiazoline moiety present in Pcb is replaced by a thiazole ring (compounds **6** and **7**), along with some others that also present a modification in the configuration of the hydroxyl group at position 13 as R (compounds **8** and **9**). The evaluation of their capacity to coordinate Ga^3+^ allowed us to deduce some structural requirements for the coordination of metals that will be very useful in the search for a more stable and simpler analogue to develop biotechnological applications.

## Results and Discussion

### Analogue Design

The presence of a labile thiazolidine ring and the acid-sensitive β-hydroxy-2,4-disubstituted α**-**methylthiazoline moiety could explain the low stability of Pcb (**2**). To explore one of these structural features, we carried out the total stereoselective synthesis of two series of Pcb analogues (**7** and **9**), where the α**-**methylthiazoline ring in **2** was replaced by a more stable thiazole ring due to its aromatic character. To evaluate the influence of the hydroxy group at the C-13 position for Ga^3+^ coordination, the two series differed in the configuration at that position.

**Scheme 1:**
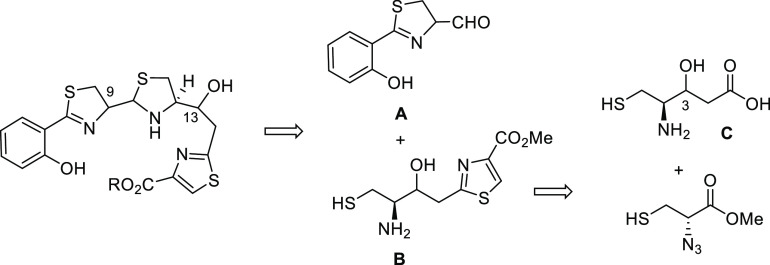
Retrosynthetic Analysis of Pcb Thiazole Analogues **6–9**

Following a similar synthetic strategy employed in the convergent synthesis of the Ga^3+^-Pcb complex (**2**-Ga^3+^), recently accomplished by our research group,^29^ Pcb analogues **6–9** would be prepared from the condensation of different stereoisomers of fragments A and B where fragment B bears a thiazole ring instead of the α**-**methylthiazoline ring present in Pcb (**1**). Fragment B would be obtained by coupling a β-hydroxy acid (fragment C) and the (*S*)-α-azidocysteine methyl ester (Scheme 1).

**Scheme 2:**
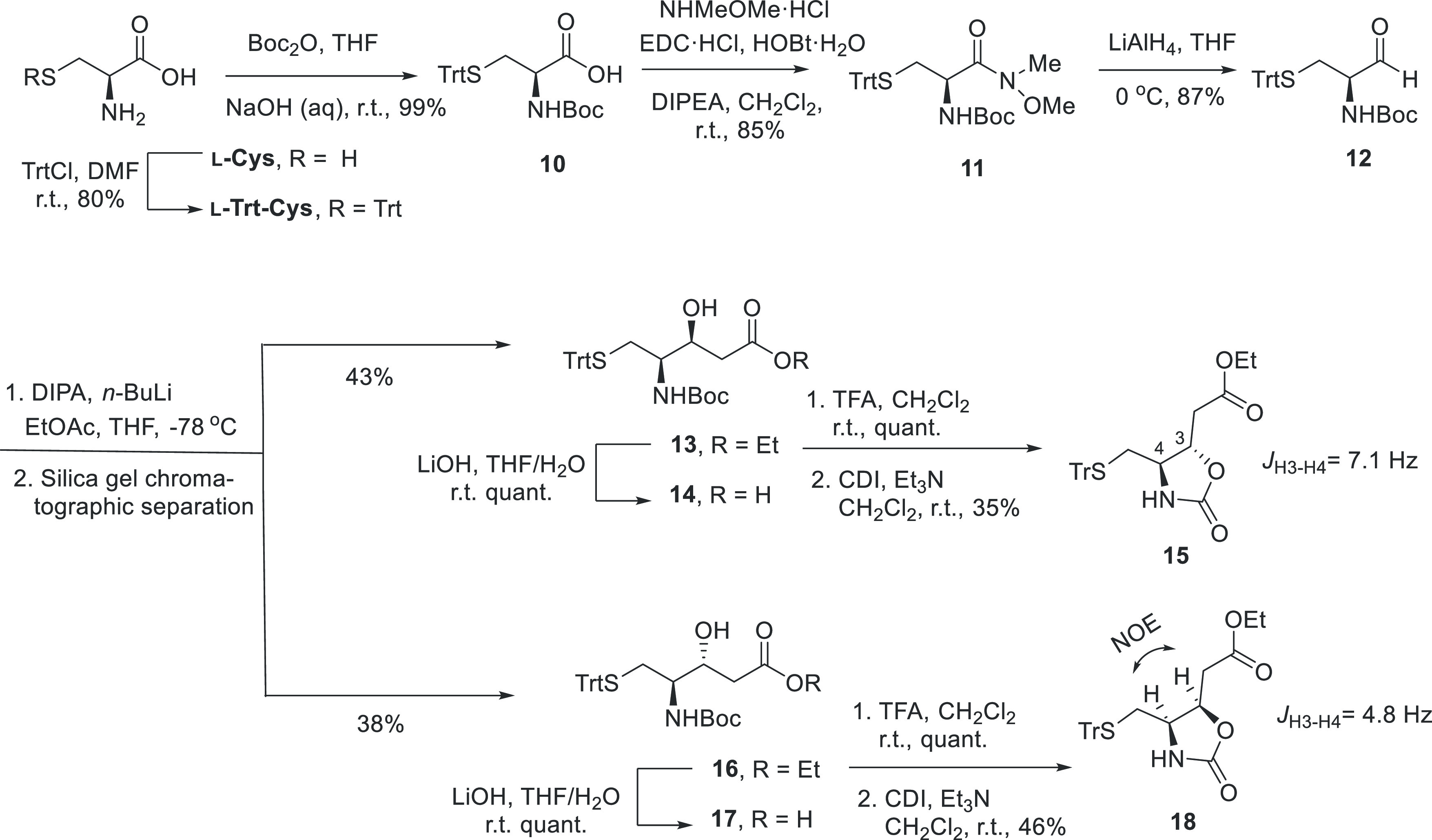
Synthesis of Epimeric β-Hydroxycarboxylic Acids **14** and **17**

The synthetic plan to obtain the two epimeric β-hydroxycarboxylic acids at C-3 in fragment C (**14** and **17**) was initially based on an aldol reaction between the suitably protected aminoaldehyde **12** and ethyl acetate. **12** was then prepared from L-cysteine in four steps as it is described in the literature.^30^ Thus, protection of the thiol group of L-cysteine hydrochloride by treatment with trityl chloride followed by protection of the amine group with the *tert*-butoxycarbonyl group gave the protected aminoacid Boc-L-Cys(Trt)-OH (**10**). Then, the Weinreb amide **11**, obtained by the treatment of acid **10** with *N*,*O*-dimethylhydroxyamine hydrochloride, was reduced to the aminoaldehyde **12** with LiAlH_4_.^31,32^ Next, the aldol reaction of ethyl acetate with **12** using *n*-BuLi^33^ gave, after chromatographic separation, the epimeric esters **13** and **16** in an equimolecular ratio (d.r. of 1:1). Their respective configurations were determined by NMR analysis of the oxazolidinone derivatives **15** and **18**, obtained by Boc-deprotection of the ethyl esters **13** and **16**, respectively, followed by cyclization with carbonyldiimidazole (CDI). Nuclear Overhauser Effect SpectroscopY (NOESY) experiments along with the displayed proton–proton coupling constants established the *syn*-**15** (^3^*J*_H4,H3_ = 7.1 Hz) and *anti*-**18** (^3^*J*_H4,H3_ = 4.8 Hz and strong Nuclear Overhauser Effect (NOE) correlation between H-3 and H-4 protons) configurations for these oxazolidinone derivatives (Scheme 2).^34^

### Synthesis of 13*S*-Pcb Thiazole Analogues **6** and **7**

**Scheme 3:**
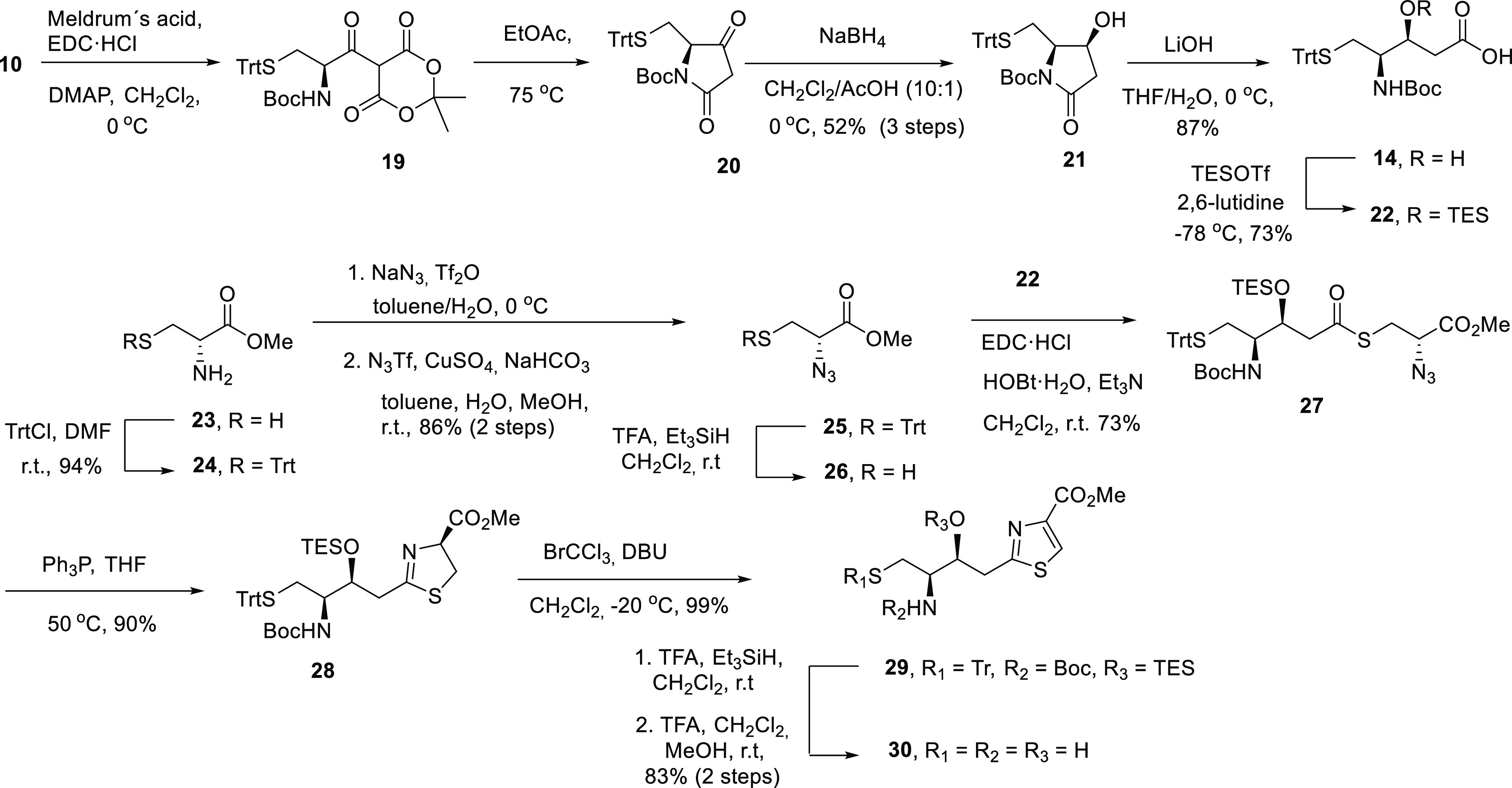
Synthesis of the Intermediate Thiazole *Syn-*Epimer **30**

Alternatively, a very highly stereoselective synthesis of the *syn*-epimer β-hydroxy acid **14** was carried out by diastereoselective reduction of tetramic acid **20** (Scheme 3).^35^ Thus, the synthesis of the tetramic acid core was performed by the condensation of *N*-protected amino acid **10** (see Scheme 2) with Meldrum’s acid in the presence of *N*-ethyl-*N*′-(3-dimethylaminopropyl)carbodiimide (EDC) and 4-(dimethylamino)pyridine (DMAP) to give **19**. Heating the resulting acyl malonate **19** induced the cycloelimination of carbon dioxide and acetone, to form an acylketene intermediate, which undergoes an intramolecular cyclization to the corresponding tetramic acid **20**. Reduction of **20** with NaBH_4_ gave alcohol **21** as a single diastereomer, which was obtained with a 52% yield. Basic hydrolysis of lactam **21** with LiOH led to *N*-Boc-γ-amino-β-hydroxy acid **14**, which displayed the same NMR spectral data and optical rotation as those of one of the compounds obtained by the aldol reaction of ethyl acetate with **12** shown in Scheme 2. *O*-Silylation of **14** was carried out with triethylsilyl trifluoromethane sulfonate and 2,6-lutidine to yield the *syn*-epimer β-hydroxy acid **22** with three convenient protecting groups.

In a parallel procedure, the trityl-protected (*S*)-α-azidocysteine methyl ester (**25**) was obtained from D-cysteine methyl ester (**23**) in two steps following a described method.^36,37^ Thus, the thiol group of D-cysteine methyl ester (**23**) was protected with trityl to give amine **24**, which was subjected to a diazo transfer reaction, using the electrophilically activated azide TfN_3_, NaHCO_3_ as the base, and CuSO_4_ as the catalyst, to afford azide **25** in a very good yield (86%) (Scheme 3).

Coupling of the triprotected acid **22**, previously activated with EDC using 1-hydroxybenzotriazole (HOBt) and triethylamine (Et_3_N) in CH_2_Cl_2_, with a freshly prepared solution of the thiol **26**, obtained by deprotection of azide **25** using trifluoroacetic acid (TFA) and triethylsilane in CH_2_Cl_2_, gave thioester **27** in a 73% yield. Subsequent one-pot Staudinger reduction–intramolecular aza-Wittig reaction of thioester **27** gave thiazoline **28** under very mild neutral conditions in an excellent yield (90%),^37^ which was then almost quantitatively oxidized to thiazole **29** with 1,8-diazabicyclo(5.4.0)undec-7-ene (DBU) and BrCCl_3_.^38^ Finally, thiazole **29** was treated with TFA and triethylsilane in CH_2_Cl_2_ with the aim of removing the three protecting groups *S*-Trt, *N*-Boc, and *O*-triethylsylil (TES). However, the TES group remained partially in these conditions. Therefore, the resulting mixture was treated again with TFA in CH_2_Cl_2_/MeOH to give the required deprotected amino thiol alcohol thiazole ester **30** (Scheme 2). It is worth highlighting the stability of this thiazole derivative during the deprotection in acid media in relation to the deprotection of a similar compound used in the synthesis of Pcb (**2**), bearing the acid-sensitive β-hydroxy-2,4-disubstituted thiazoline moiety instead, which decomposes under these acidic conditions.^29^

**Scheme 4:**
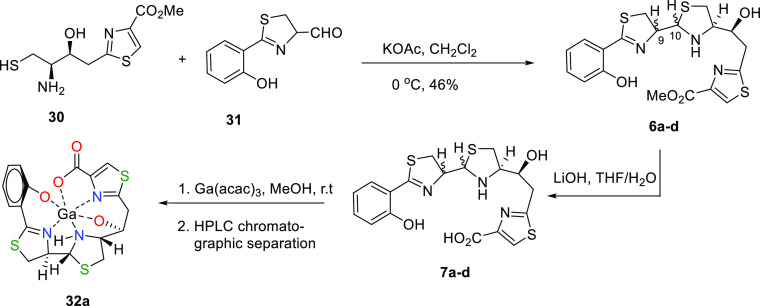
Synthesis of 13*S*-Pcb Thiazole Analogues **6** and **7** and Ga^3+^ Complex **32a**

The synthesis of 13*S*-Pcb thiazole analogues was completed by the condensation of an enantiomeric mixture of thiazolinic aldehyde **31**, the preparation of which from D-cysteine hydrochloride was already reported in the synthesis of Pcb (**1**),^29^ and the amino thiol **30** to yield a mixture of four C-9/C-10 diastereoisomeric thiazolidine methyl esters (**6a–d**) (see Scheme 4).^39,40^

The formation of the labile thiazolidine ring in **6a–d** was confirmed by the ^13^C NMR spectrum of the reaction crude showing the characteristic carbon chemical shifts of C-9 and C-10 around 81.72–79.93 and 72.70–71.80 ppm, respectively, and by its ^1^H NMR spectrum displaying the distinctive proton chemical shifts of H-9 as a multiplet around 4.99–4.83 ppm and H-10 as a doublet in the range of 4.91–4.69 ppm.

The mixture was separated by high-performance liquid chromatography (HPLC) into two main fractions, which were analyzed by 1D/2D NMR (in CD_2_Cl_2_) and MS studies. In the first fraction, the major product of the reaction, the methyl ester **6a**, along with some trace of its epimer at C-10 **6d**, was eluted with a retention time of 14.2 min. The (+)-HRESIMS data of **6a** confirmed its molecular formula as C_19_H_21_N_3_O_4_S_3_ by displaying the [M + H]^+^ ion peak at *m*/*z* 452.0769 (calc. for C_19_H_21_N_3_O_4_S_3_*m*/*z* 452.0772). ^1^H, ^13^C NMR, Distortionless Enhacement by Polarization Transfer (DEPT), COrrelation SpectroscopY (COSY), and Hetero Single Quantum Correlation (HSQC) of **6a** allowed the assignment of all the carbons to their corresponding protons. The carbon chemical shift of C-9 resonates at δ_C_ 80.72, which correlates by an HSQC experiment with H-9 at δ_H_ 4.96 as ddd (*J* = 8.8, 7.8 and 6.8 Hz), while that of C-10 resonates at δ_C_ 72.66, which correlates by HSQC with H-10 at δ_H_ 4.75 as a d with a *J* value of 6.8 Hz. The *anti*-disposition between H-9 and H-10 was deduced from the *J* value of 6.8 Hz between these protons, the NOE correlation between H-12 at δ_H_ 3.30 ddd (*J* = 9.9, 6.1, and 3.6 Hz) and H-10 at δ_H_ 4.75, and the absence of NOE between the H-9 and H-10 protons (Tables S1 and S2). In this way, the configuration of thiazole piscibactin methyl ester **6a** was established as 9*R*,10*R*,12*R*,13*S*.

The second fraction, eluted with a retention time of 17.2 min, was assigned to a mixture of two diastereomers, **6b** and **6c**, in a ca. 3:2 ratio, being the minor products of the reaction. Again, 1D and 2D NMR analysis and MS of the mixture enabled us to determine their respective configurations. In this case, the δ_C_/δ_H_ 81.72 (CH)/4.99 (1H, dt, *J* = 5.5, 8.8, and 8.8 Hz) values were assigned to C-9/H-9 in **6b**, while δ_C_/δ_H_ 79.93 (CH)/4.83 (1H, ddd, *J* = 7.9, 8.2, and 8.7 Hz) were assigned to that position in **6c**. Additionally, δ_C_/δ_H_ 72.39 (CH)/4.91 (1H, d, *J* = 5.5 Hz) and 71.78 (CH)/4.69 (1H, d, *J* = 8.2 Hz) were assigned to C-10/H-10 resonances in **6b** and **6c**, respectively (Tables S1 and S3). Then, the small value of ^3^*J*_H9H10_ = 5.5 Hz in diastereoisomer **6b**, indicating a *syn*-disposition between H-9 and H-10, along with the NOE correlation between H-10 at δ_H_ 4.91 and H-12 at δ_H_ 3.32 dd (*J* = 6.0 and 9.8 Hz), suggested the 9*S*,10*R*,12*R*,13*S* configuration for **6b**. On the other hand, the large value of ^3^*J*_H9H10_ = 8.2 Hz, in agreement with an *anti*-disposition between H-9 and H-10 protons in diastereoisomer **6c** and the lack of NOE correlation between H-10 at δ_H_ 4.91 and H-12 at δ_H_ 3.43 dd (*J* = 5.9 and 10.9 Hz), allowed us to propose the 9*S*,10*S*,12*R*,13*S* configuration for **6c**.

**Scheme 5:**
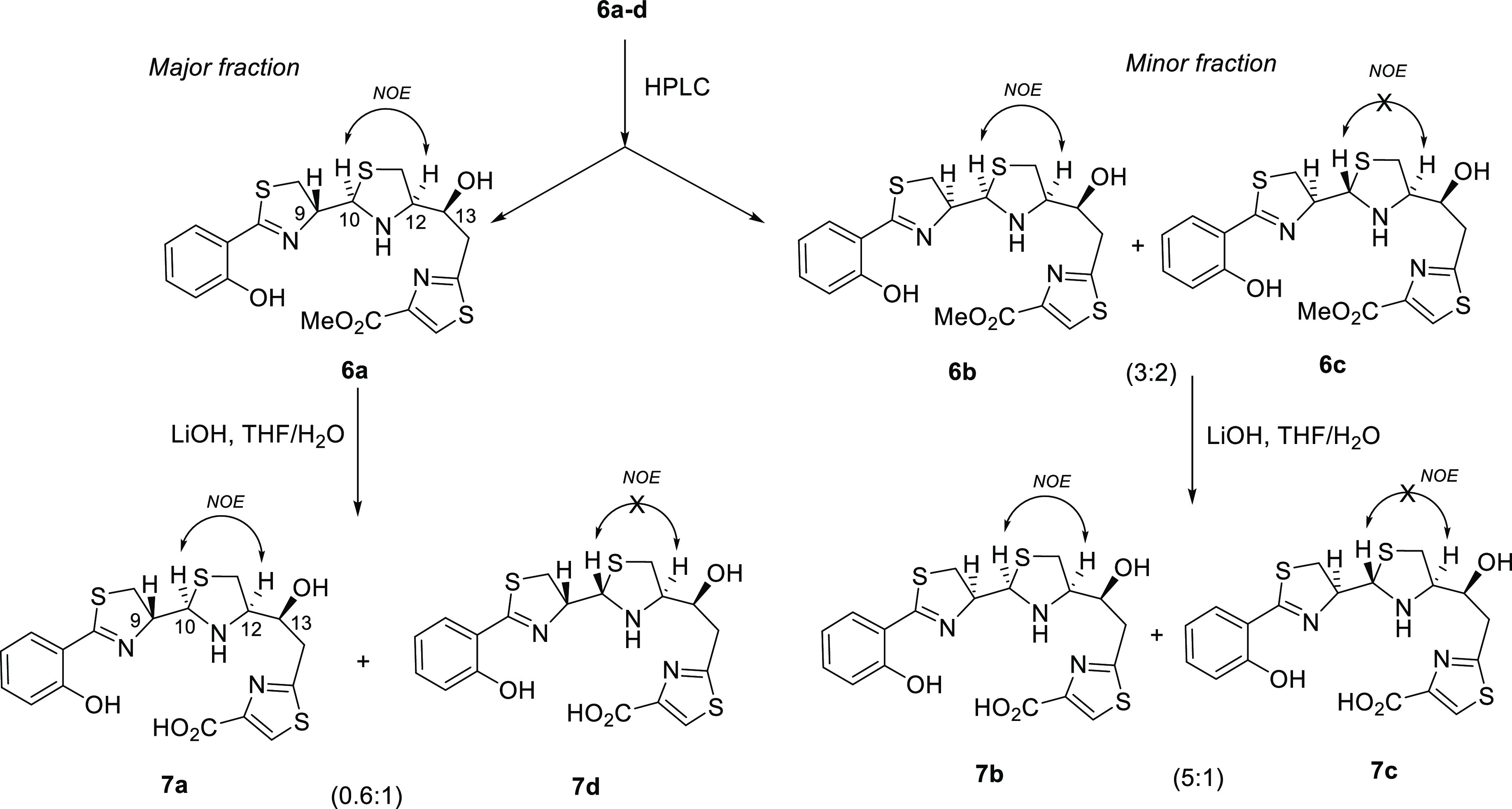
HPLC Separation of 13*S*-Pcb Thiazole Analogues **6a–d** and Conversion in **7a–d**

Thiazole methyl ester **6a** and the **6b**/**6c** mixture were submitted separately to basic hydrolysis with LiOH to afford carboxylic acids **7a–d**. The lack of the carbon and proton signals corresponding to the methyl group in their NMR spectra and the [M – H]^−^ ion peak at *m*/*z* 436.0457 (calcd. for C_18_H_18_N_3_O_4_S_3_, *m*/*z* 436.0464) in its (−)-HRESIMS confirmed the formation of the desired product. Even though attempts to separate the diastereoisomeric mixture by HPLC were unsuccessful, NMR analysis allowed us to identify carboxylic acids **7a** and **7d** as a diastereoisomeric mixture in a ca. 0.6:1 ratio, and carboxylic acids **7b** and **7c** as a diastereoisomeric mixture in a ca. 5:1 ratio was obtained from hydrolysis of the **6b** and **6c** mixture (Scheme 5).

### Ga^3+^ Complexation of 13*S*-Pcb Thiazole Analogues **7** and DFT Calculations of Ga^3+^ Complexes **32**

To explore the ability of 13*S*-Pcb thiazole analogues **7a–d** to chelate gallium(III), the mixture of the four diastereoisomeric carboxylic acids obtained directly from basic hydrolysis of **6a–d** was complexed with gallium(III) using Ga(acac)_3_ without previous purification.^41^ RP-HPLC analysis of the crude reaction, using an analytical column and a H_2_O/CH_3_CN gradient mixture, allowed the detection of the Ga^3+^ complex **32a** as the major compound eluted with a retention time of 13.0 min. Final semipreparative HPLC separation led to the purification of Ga^3+^ complex **32a** from the chromatographic peak eluted with a retention time of 25.8 min. The [M – H]^−^ ion peak cluster at *m*/*z* 501.9485/503.9477 (calcd. for C_18_H_15_^69^GaN_3_O_4_S_3_*m*/*z* 501.9486) in a ca. 3:2 ratio allowed us to determine the molecular formula of the Ga^3+^ complex **32a** as C_18_H_16_GaN_3_O_4_S_3_. ^1^H, ^13^C, DEPT, NMR, COSY, and HSQC experiments allowed us to assign all the protons to their corresponding carbons. The configuration was fixed through the *J* values and a NOESY experiment. Indeed, the large ^3^*J*_H9H10_ value of 10.0 Hz was indicative of the *anti*-disposition between H-9 and H-10 protons, while the NOE correlation between H-10 at δ_H_ 4.81 and H-12 at δ_H_ 3.76 displays that these protons are on the same face of the thiazolidine ring. Besides, NOE cross-peaks between H8_h_/H10, H11_l_/H12/H14_l_ and between H8_l_/H9/H11_h_/H13/H14_h_ revealed their respective *cis* relationships. All these data together indicate that the configuration for the Ga^3+^ complex **32a** is 9*R*,10*R*,12*R*,13*S* (Scheme 4).

**Scheme 6:**
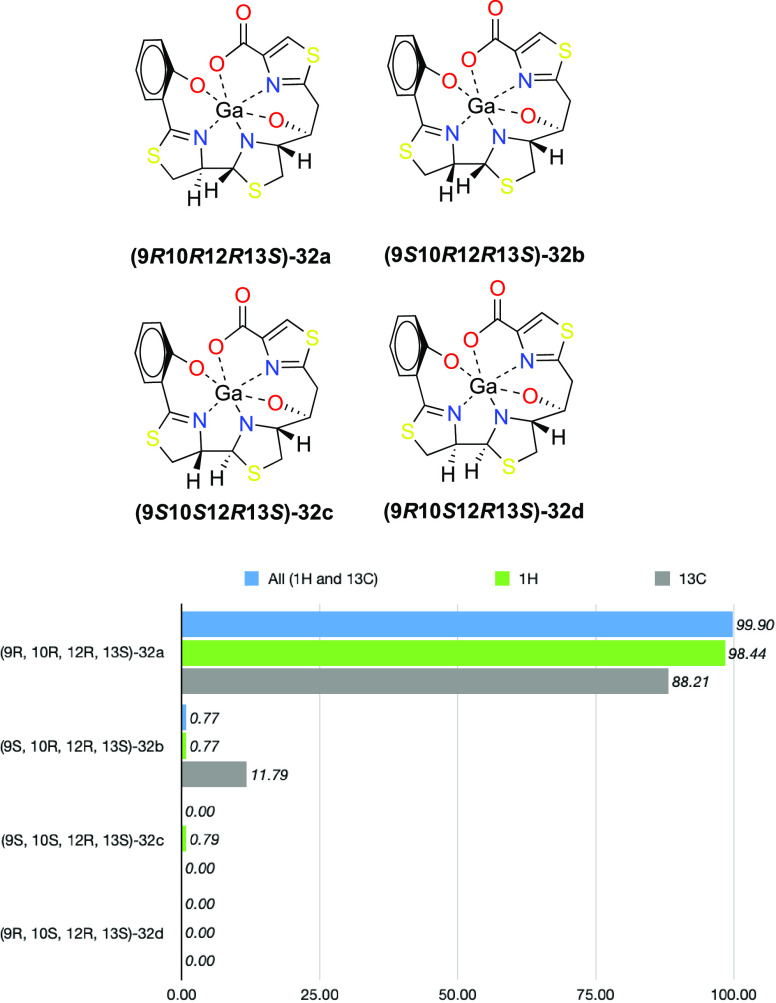
DP4+ Parameters Calculated by DFT-NMR Calculations for **32a–d**

To confirm the stereochemistry of **32a**, NMR-Gauge Independent Atomic Orbital (GIAO)-DFT calculations were performed in four possible gallium-complexed diastereoisomers at C9, C10 (**32a–d**), keeping the stereocenters C12 and C13 fixed as *R* and *S*, respectively. Therefore, configurations (9*R*,10*R*,12*R*,13*S*), (9*R*,10*S*,12*R*,13*S*), (9*S*,10*R*,12*R*,13*S*), and (9*S*,10*S*,12*R*,13*S*) were minimized with the functional B3LYP/6-31G+(d,p) level of theory using the methanol PCM model. NMR chemical shifts and coupling constants were calculated at the same level of theory, and then DP4+ statistical parameters were computed for each diastereoisomer by using Sarotti’s spreadsheet.^42,43^ Configuration (9*R*,10*R*,12*R*,13*S*) yielded the best fit with values of 99.90% taking into consideration both ^1^H and ^13^C chemical shifts, 98.44% counting the ^1^H values, and 88.21% with ^13^C values (see Scheme 6).

### Synthesis of 13*R*-Pcb Thiazole Analogues **8** and **9**

**Scheme 7:**
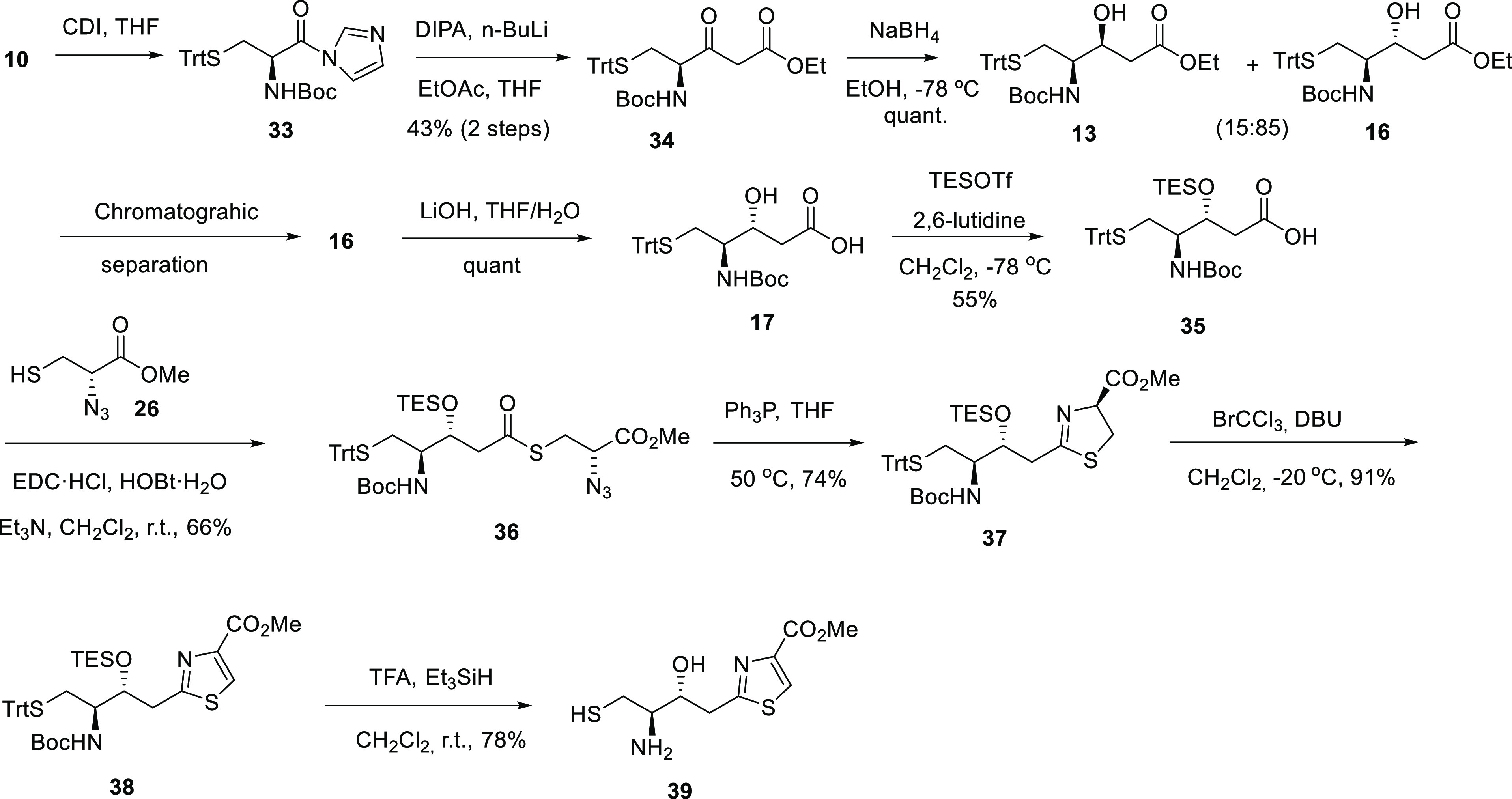
Stereoselective Synthesis of Intermediate Thiazole *Anti-*Epimer **39**

The 13*R*-Pcb thiazole analogues **9** were designed to test the influence of the OH configuration at C-13 in the coordination with Ga^3+^. The synthesis was carried out using the β-hydroxy acid **17**. As outlined in Scheme 7, we developed a stereoselective route for the preparation of **17** as an alternative method to the aldol reaction performed with ethyl acetate (Scheme 2). This synthesis started by activation of the protected amino acid Boc-L-Cys(Trt)-OH (**10**) using 1,1′-carbonyldiimidazole (CDI), to give **33**, followed by in situ condensation with the lithium enolate of ethyl acetate to produce the β-keto ester **34** in a 43% yield. Next, stereoselective reduction of **34** with NaBH_4_ afforded a mixture of epimeric alcohols **13** and **16**, being the *anti*-epimer **16** the major product. Analysis of the ^1^H-NMR spectrum of the crude reaction shows a 15:85 ratio of **13–16** (Scheme 7).^44^ After chromatographic separation of the epimeric mixture of **13** and **16**, basic hydrolysis of β-hydroxy ethyl ester **16** afforded *N*-Boc-γ-amino-β-hydroxy acid **17**, which was protected as triethylsilyl ether to give acid **35** and subsequently coupled with a freshly prepared azide **26** to furnish thioester **36**. Transformation of thioester **36** into thiazoline **37**, via intramolecular Staudinger reduction/aza-Wittig reaction, followed by oxidation to thiazole **38** and a final deprotection step gave thiazole **39** using a similar sequence to that employed in the preparation of its epimer thiazole **30** (Scheme 7).

**Scheme 8:**
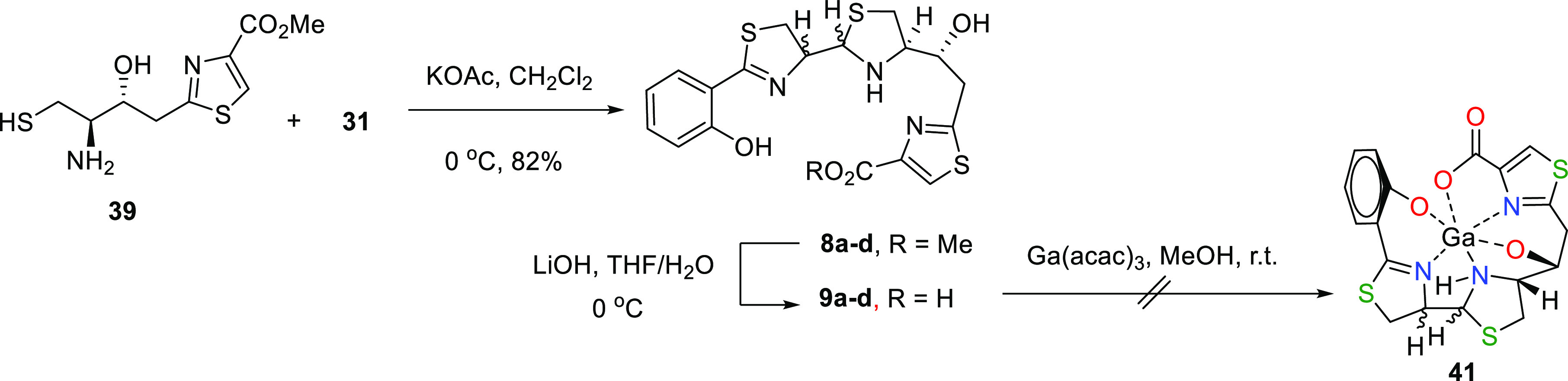
Synthesis of (13*R*)-Pcb Thiazole Analogues **8a–d**, **9a–d**, and Ga^3+^ Complexation Attempts

Aminothiol **39** was coupled with aldehyde **31** to obtain the thiazole methyl esters **8a–d** as a mixture of four diastereoisomers at C-9 and C-10 positions (see Scheme 8).

**Scheme 9:**
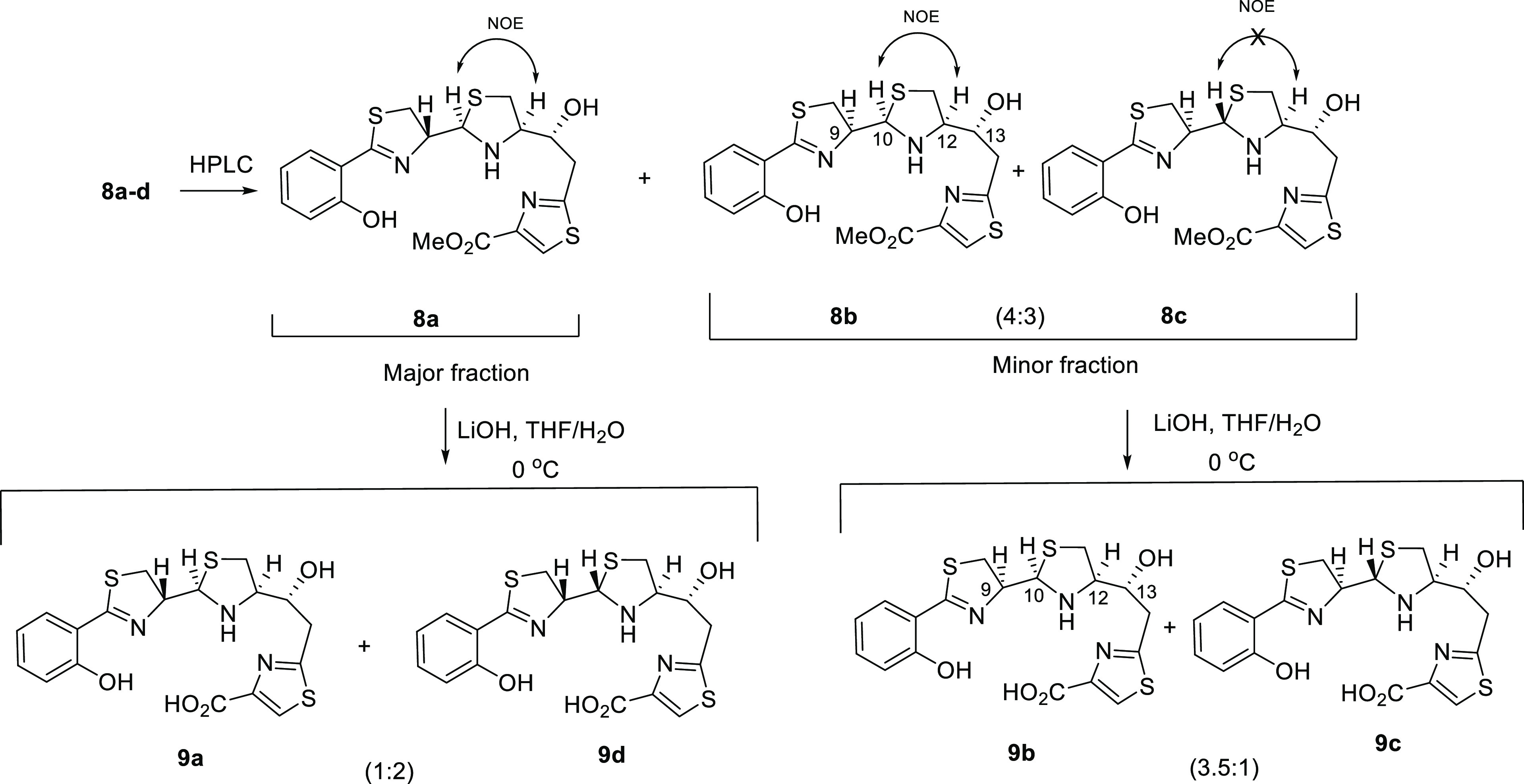
HPLC Separation of Compounds **8a–d** and **9a–d**

HPLC separation of the mixture gave two fractions (see Scheme 9). In the first fraction, eluted with a retention time of 19.0 min, **8a** was isolated as an almost pure compound being the major product of the reaction. The molecular formula of C_19_H_21_N_3_O_4_S_3_ for **8a** was deduced from the [M + H]^+^ ion peak at *m*/*z* 452.0774 observed in its (+)-HRESIMS (calcd. for C_19_H_22_N_3_O_4_S_3_^+^, *m*/*z* 451.0766). The *syn*-disposition of H-9/H-10 protons in **8a** was deduced from the 1D and 2D NMR (in CD_2_Cl_2_) analyses, which display the C-9/H-9 resonances at δ_C_/δ_H_ 80.56 (CH)/4.96 (1H, ddd, *J* = 6.5, 7.7, and 8.9 Hz) and those of C-10/H-10 at δ_C_/δ_H_ 72.12 (CH)/4.78 (1H, d, *J* = 6.5 Hz). Moreover, the NOE correlation between H-12 at δ_H_ 3.32 (1H, ddd, *J* = 4.8, 6.0, and 10.0 Hz) and H-10 at δ_H_ 4.78 indicated the *syn*-disposition of these protons. All these information suggest a 9*R*,10*R*,12*R*,13*R* configuration for **8a**.

The second fraction eluted from the HPLC analysis (see Scheme 9), with a retention time of 21.30 min, resulted to be a ca. 4:3 mixture of two diastereomers, **8b** and **8c**, as the minor products of the reaction. Again, 1D and 2D NMR analysis in CD_2_Cl_2_ and MS of that mixture allowed us to determine their structures. The carbon and proton chemical shifts of the key C-9 and C-10 positions in **8b** and **8c** are shown in Table S1. The *syn*-disposition between H-9 and H-10 in diastereoisomer **8b** was deduced from the small ^3^*J*_H9H10_ value of 5.7 Hz. In parallel, the NOE correlation between H-10 at δ_H_ 4.93 and H-12 at δ_H_ 3.34 (1H, ddd, *J* = 3.1, 5.5, and 9.8 Hz) indicated a *syn*-configuration of these protons in the thiazolidine ring. From these data, we suggest the configuration of 9*S*,10*R*,12*R*,13*R* for **8b**. On the other hand, the large value of ^3^*J*_H9H10_ of 8.2 Hz in **8c**, indicating the *anti*-disposition of H-9 and H-10 and the lack of an NOE correlation between H-10 at δ_H_ 4.67 and H-12 at δ_H_ 3.43 (1H, dd, *J* = 5.9 and 10.9 Hz), suggested the configuration of 9*S*,10*S*,12*R*,13*R* for **8c** (Table S1).

Basic hydrolysis of thiazole methyl ester **8a** with LiOH gave a diastereoisomeric mixture of carboxylic acids **9a** and **9d** in a ca. 1:2 ratio, while basic hydrolysis of the **8b**/**8c** mixture yielded **9b**/**9c** as a mixture in a ca. 3.5:1 ratio. In both cases, the molecular formula of C_19_H_21_N_3_O_4_S_3_ of **9** was deduced from the [M – H]^−^ ion peak at *m*/*z* 436.0468 or 436.0457 observed in their (−)-HRESIMS data (calcd. for C_18_H_18_N_3_O_4_S_3_, *m*/*z* 436.0464). The NMR data of acids **9a–d** show a trend of ^1^H and ^13^C chemical shifts, along with the *J*_H9–H10_ coupling constant very similar to those found for **7a–d**, which allowed us to confirm the stereochemistry at C-9 and C-10 positions for each diastereoisomer (Table S1).

### Ga^3+^ Complexation Attempts of 13*R*-Pcb Thiazole Analogues **9**

Finally, although carboxylic acids **9a–d** were submitted to complexation with gallium using Ga(acac)_3_ under different conditions, the NMR and MS analyses of the crude reactions did not show any indication of gallium chelation (Scheme 8). The nonformation of the corresponding Ga^3+^ complex from **9a–d** suggests that the configuration of the hydroxyl group at C-13 is crucial for complexation with Ga^3+^ in these types of structures.

### DFT Calculations to Predict the Potential Formation of Gallium Complexes

**Figure 2:**
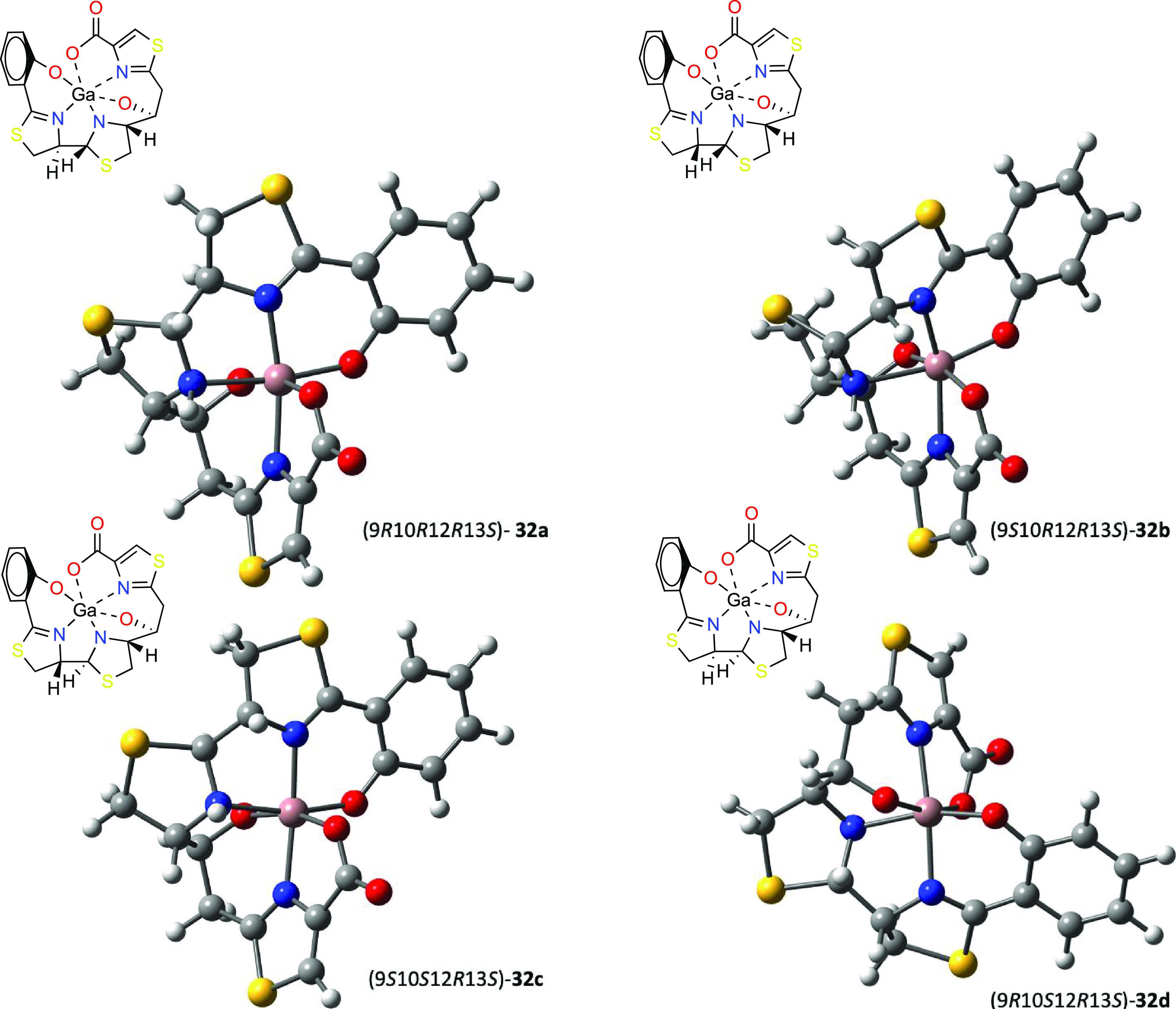
DFT geometries of the gallium(III) complexes **32a–d**.

To gain further insight into the nonformation of the Ga^3+^ complexes with (*13R*)-Pcb thiazole analogues **9a–d**, we analyzed the structures of **2-Ga^3+^**, the four possible gallium-complexed diastereoisomers at C9, C10 (**32a–d**), Fe(III)-yersiniabactin complex (**4-Fe^3+^**), and the hypothetical gallium complex **41** with 9*R*,10*R*,12*R*,13*R* configuration, using computational methods. Subsequent DFT optimizations of all compounds allowed us to deduce further details from the coordination sphere of the gallium atom, which has a distorted octahedral geometry. Although the transverse angles of an ideal octahedral gallium(III) d^2^sp^3^ hybrid should be 180°, this optimal structure cannot be achieved due to the asymmetric distribution of the six coordinated atoms (three oxygens and three nitrogens). Based on our previous studies^19^ on **2-Ga^3+^** and the X-ray structure^45^ of **4-Fe^3+^**, just the meridional (*mer*) isomers were considered. In our calculations, we observed average distances of 1.95 Å for the Ga–O bond in the O–Ga–O, 2.06 Å for the Ga–N bond in the O–Ga–N, and 2.04 Å for the Ga–N bond in the N–Ga–N dispositions in **32a–d** and **2-Ga^3+^**, values which are in good agreement with similar gallium complexes reported in the literature.^46,47^ We would expect the sum of the transverse angles of the ideal octahedral structure to be around Σ_angles_ = 540° (three times 180°), and a deviation from this value could provide an indication of the stability of the complex. Additionally, the difference between the sum of the Ga-heteroatom bond lengths for N–Ga–N, O–Ga–N, and O–Ga–O dispositions and the transverse distance between heteroatoms N···N, O···N, and O···O, respectively, would give us a degree of distortion of the octahedral structure. Thus, B3LYP6-31 G(d,p) geometries of **32a–d** (see Figure 2) and (9*R*,10*R*,12*R*,13*R*)-**41** were calculated and then compared with the DFT-calculated structure of the gallium(III) piscibactin complex (**2-Ga^3+^**)^19^ and the X-ray data of the Fe(III)-yersiniabactin complex (**4-Fe^3+^**),^45^ which have adequate values to form the gallium/iron complexes. The sum of transverse angles for the N–Ga–N, O–Ga–N, and O–Ga–O dispositions of the DFT structures **32a–d** and **2-Ga^3+^** (also in **4-Fe^3+^** bearing Fe instead of Ga) shows a range value of Σ_angles_ = 483–500°, while the DFT structure of (9*R*,10*R*,12*R*,13*R*)-**41** (epimer of **32a** at C13) displays a sum of transverse angles of Σ_angles_ = 452° (Table 1). On the other hand, the difference values between the transverse distance and the sum of metal–heteroatom (N or O) distances (in absolute value) are smaller (0.01–0.14) in the DFT-determined structures **32a–d** and **2-Ga^3+^** (also in **4-Fe^3+^** bearing Fe instead of Ga) than that of (9*R*,10*R*,12*R*,13*R*)-**41** (0.13–0.31). Moreover, the standard deviation (STD) of those values is smaller for the case of **32a–d** and **2-Ga^3+^** (STD = 0.03–0.07) than that of (9*R*,10*R*,12*R*,13*R*)-**41** (STD = 0.16) (Table 2). These results would explain the nonformation of the gallium complex for the analogue with the configuration 13*S*. Furthermore, the better key values observed in **32a** (Σ_angles_ = 500°, STD = 0.03) than those of the other three possible diastereoisomers **32b–d** (Σ_angles_ = 488–490°, STD = 0.04–0.07) could explain the preference in the formation of this gallium complex. The strategy using these DFT calculations could be very useful to predict the potential formation of gallium complexes in these types of compounds.

**Table 1:**
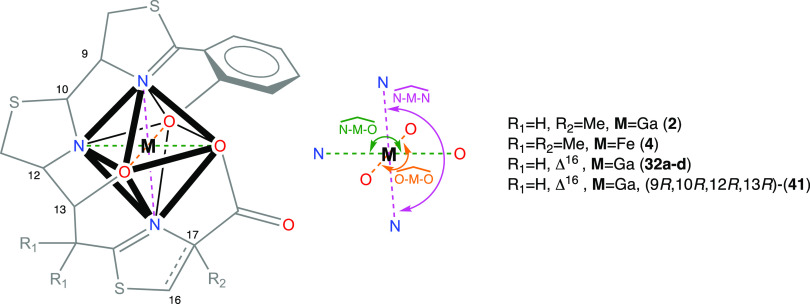
Octahedral Environmental Transverse Angles for **2-Ga^3+^**, **4-Fe^3+^**, **32a–d**, and **41** Complexes

comp	O–M–O^a^	O–M–N^b^	N–M–N^c^	sum^d^
**2-Ga^3+^**	154	168	167	489
**4-Fe^3+^**	158	162	163	483
**32a**	166	171	163	500
**32b**	164	168	156	488
**32c**	164	165	162	491
**32d**	163	165	162	490
**41**	132	153	167	452

a O–M–O transverse angle.

b O–**M**–N transverse angle.

c N–**M**–N transverse angle.

d Sum = Σ_angles_ (O–**M**–O transverse angle + O–**M**–N transverse angle + N–**M**–N transverse angle).

**Table 2:**
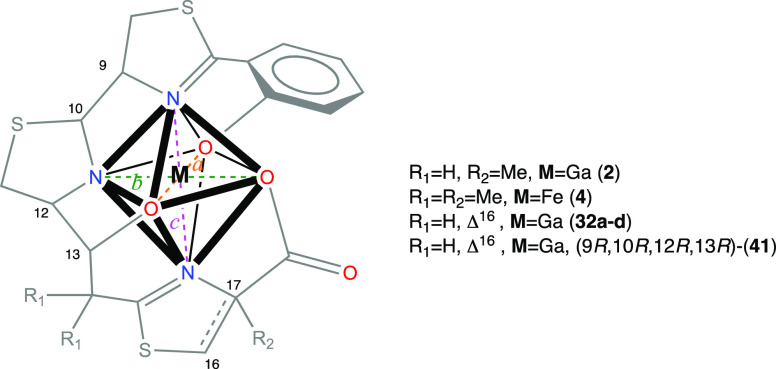
Octahedral Environmental Distances for **2-Ga^3+^**, **4-Fe^3+^**, **32a–d**, and **41** Complexes^a^

comp	metal–O distances		metal–N or metal–O distances		metal–N distances		O–O, O–N, or N–N transverse distances			Δ_O–O_^b^	Δ_O–N_^b^	Δ_N–N_^b^	STD^c^
	O–M–O	O–M–O	N–M–O	N–M–O	N–M–N	N–M–N	O–O(a)	O–N(b)	N–N(c)				
**2-Ga^3+^**	1.98	1.91	2.34	1.91	2.02	2.13	3.79	4.23	4.13	0.10	0.02	0.02	0.05
**4-Fe^3+^**	2.08	1.90	2.21	1.93	2.06	2.13	3.91	4.10	4.14	0.07	0.04	0.05	0.05
**32a**	1.99	1.90	2.20	1.92	2.06	1.97	3.86	4.10	3.99	0.03	0.02	0.04	0.03
**32b**	1.99	1.90	2.22	1.92	2.07	1.98	3.86	4.13	3.96	0.03	0.01	0.09	0.04
**32c**	1.98	1.92	2.13	1.91	2.08	2.02	3.86	4.01	4.05	0.04	0.03	0.05	0.04
**32d**	2.01	2.01	2.12	1.91	2.12	1.91	3.88	4.00	4.07	0.14	0.03	0.04	0.07
**41**	1.90	1.83	1.87	3.49	2.19	2.07	3.42	5.23	4.23	0.31	0.13	0.03	0.16

a Bond lengths in Å.

b Difference between transverse distance and the sum of metal–heteroatom (N or O) distances (in absolute value).

c Standard deviation of Δ values from entry b.

### Antimicrobial Assays of Gallium Complexes

Gallium has been described as an antibacterial agent, displaying ranges of minimum inhibitory concentrations (MICs) against some bacteria, comparable to those of conventional antibiotics.^48^ Therefore, we tested the antimicrobial activity of gallium complex **32a** and Pcb-Ga^3+^ (**2-Ga^3+^**) against *V. anguillarum*. Neither gallium complex **32a** nor Pcb-Ga^3+^ (**2-Ga^3+^**) inhibited the growth of *V. anguillarum* at concentrations of 5–500 μM. By contrast, the MIC of GaBr_3_ was 50 μM. The negative results in these biological assays agree with one of the reported roles for siderophores, protecting pathogens from metal ion toxicity.^49^

## Conclusions

Piscibactin (**2**) is the siderophore responsible for iron uptake and a major virulence factor for several relevant Gram-negative bacteria pathogenic for humans and animals.^22,23^ The low stability of this compound avoids its use as a vector to develop interesting biotechnological applications, so the search for structural requirements for metal coordination is an important step to find a stable analogue of Pcb. To pursue this goal, we have prepared two sets of Pcb analogues, and they were evaluated as Ga^3+^ chelators as mimics for Fe^3+^ coordination. The results showed that Ga^3+^ coordination is not affected by the substitution of the thiazoline ring in the acid-sensitive β-hydroxy-2,4-disubstituted α**-**methylthiazoline moiety in Pcb (**2**) for a more stable thiazole ring. However, the configuration of the hydroxyl group at C-13 is crucial for the chelation of Ga^3+^, and the 13*S* configuration must be maintained to preserve the metal coordination. The Ga^3+^ chelation studies with the present Pcb analogues agree with the positive chrome azurol-S liquid assay shown by **7** and the negative outcome displayed by **9**. Moreover, these results are also in agreement with our reported studies of the siderophore activity of these Pcb thiazole analogues measuring their ability to be internalized through FrpA, the outer membrane transporter for ferri-piscibactin in *V. anguillarum*. These studies displayed the ability of the methyl esters **6a–d** and carboxylic acids **7a–d** to promote the growth of *V. anguillarum* under iron deficiency and the lack of this activity for methyl esters **8a–d** and carboxylic acids **9a–d**.^50^ The stereochemistry of Ga^3+^ complex **32a**, among the six possible diastereoisomers, was confirmed by using DFT calculations, which were also used for deducing the ability of these thiazole analogues to form octahedral coordination spheres with gallium. These structural requirements found with synthetic and DFT-computational arguments are very useful in the search of a more stable Pcb analogue that can be used as a vector for future conjugation with antibiotics following the “Trojan horse strategy” or with fluorophores for designing fluorescent probes.^8,51^

## Experimental Section

### Materials and Methods

#### General Procedure for Synthesis

All moisture-sensitive reactions were carried out under an atmosphere of argon in a flame-dried glassware, closed by rubber septum unless otherwise noted. Solvents were distilled before use under an argon atmosphere and dried according to standard procedures using the following desiccants: Na/benzophenone for tetrahydrofuran and Et_2_O, CaH_2_ for dichloromethane or, alternatively, a solvent purification system (MBraun 800) was used for drying. 2,6-Lutidine was distilled under vacuum from KOH and stored over 4 Å MS under argon. Diisopropylamine (DIPA) was distilled from KOH and stored over 4 Å MS under argon. Ethyl acetate was distilled from molecular sieves and stored over 4 Å MS under argon. Anhydrous solvents (dimethylformamide, methanol, triethylamine, and diisopropylethylamine) were purchased from commercial sources. Solutions and solvents were added via a syringe or cannula. Thin-layer chromatography was performed using silica gel GF-254 Merck, spots were revealed employing UV light (254 nm) and/or by heating the plate pre-treated with an ethanolic solution of phosphomolybdic acid. Cryocool apparatus was used for low-temperature reactions. When necessary, reactions were heated using a stir plate equipped with an aluminum heating block. Medium-pressure chromatographic separations were carried out on silica gel 60 (230–400 mesh). HPLC purification was carried out in equipment coupled to a Photodiode Array (PDA) detector using Milli-Q water and HPLC-grade solvents.

#### Spectroscopic Measurements and Electrospray Ionization Mass Spectrometry

The ^1^H and ^13^C NMR spectra were recorded on Bruker AVANCE 500, AVANCE III HD 400 or NEO 300 spectrometers, using CDCl_3_, CD_2_Cl_2_, CD_3_OD, or DMSO-d_6_ as the solvents and internal standards. Optical rotations were determined on a JASCO DIP-1000 polarimeter, with a Na (589 nm) lamp and a filter. LR-ESIMS and HR-ESIMS were measured using an Applied Biosystems QSTAR Elite mass spectrometer or a ThermoLTQ-Orbitrap Discovery mass spectrometer.

#### Computational Methods

Conformational searches for DP4+ analyses were performed by employing MAESTRO software, using an energy window of 5 kcal/mol. DFT geometries were calculated using Gaussian 16 with the combination B3LYP/6-31G+(d,p) and an IEFPCM model of MeOH. Vibrational frequency calculations were also performed to confirm the presence of minima energy conformers.. Chemical shielding tensors (CSTs) were computed at GIAO/B3LYP/6-31G(d,p) (in gas phase) level of theory. For octahedral geometries of compounds **32a–d** and **41**, B3LYP/6-31G(d,p) calculations were performed.

#### Bacterial Growth Inhibition Assays

To test the susceptibility of *V. anguillarum* to gallium(III), the RV22 *vabF* mutant strain was challenged to grow in the presence of GaBr_3_, gallium complex **32a**, or Pcb-Ga^3+^ (**2-Ga^3+^**). Growth was evaluated in 96-well microtiter plates containing 200 μL per well as the final volume. An overnight culture of *V. anguillarum* was adjusted to an OD_600_ = 0.5, and a final dilution of 1:30 was inoculated in a CM9 minimal medium supplemented with 30 μM 2,2′-dipyridyl. GaBr_3_, gallium complexes **32a**, and Pcb-Ga^3+^ (**2-Ga^3+^**) were tested at increasing concentrations between 5 and 500 μM. The microplate was incubated at 25 °C with shaking at 120 rpm. Growth was recorded for 18 h in an iMACK Microplate reader (Bio-Rad). Each condition was assayed in duplicate in the microplate, and three independent experiments were performed.

## Supporting Information Available

Detailed data including experimental synthetic procedures of Pcb thiazole analogues and all intermediates, 1D (^1^H and ^13^C) and 2D NMR spectral data, HRESIM spectra, and DFT calculations (PDF)
