# Supplementary material for: Structural Requirements for Ga3+ Coordination in Synthetic Analogues of the Siderophore Piscibactin Deduced by Chemical Synthesis and Density Functional Theory Calculations
Source: Inorg Chem. 2023 May 4;62(19):7503–14. doi: 10.1021/acs.inorgchem.3c00787 (PMC10189737; doi:10.1021/acs.inorgchem.3c00787)
Supplement: Supplementary file 1 — ic3c00787_si_001.pdf [file ic3c00787_si_001.pdf]

Supporting Information

Structural requirements for Ga^{3+} coordination in synthetic analogues of the siderophore piscibactin deduced by chemical synthesis and DFT calculations

M. Carmen de la Fuente†, Lucía Ageitos†, Marta A. Lages‡, Diana Martínez-Matamoros†, Abel M. Forero†, Miguel Balado‡*, Manuel L. Lemos‡, Jaime Rodríguez†*and Carlos Jiménez†*

† CICA – Centro Interdisciplinar de Química e Bioloxía, Departamento de Química, Facultade de Ciencias, Universidade da Coruña, 15071 A Coruña, Spain

Emails: jaime.rodriguez@udc.es; carlos.jimenez@udc.es

‡ Departamento de Microbiología y Parasitología, Instituto de Acuicultura, Universidade de Santiago de Compostela, 15782 Santiago de Compostela, Spain

Email: miguel.balado@usc.es

Index:

1. Synthetic procedures and characterization data for the prepared compounds 8

1.1 Synthesis of epimeric β-hydroxy carboxylic acids 14 and 17 (Scheme 2) 8

1.2 Synthesis of intermediate thiazole syn epimer 30 (Scheme 3) 11

1.3 Synthesis of 13S Pcb thiazole analogues 6 and 7 and Ga^{+3} complex 32 (Schemes 4 and 5) 16

1.4 Stereoselective synthesis of thiazole intermediate epimer 39 (Scheme 7) 21

1.5 Attempts of Gallium (III) complexation of 9a-d 28

Table S1. Diagnostic NMR chemical shifts and coupling constants at C-9 and C-10 positions in 6–9.. 28

2. NMR spectra and MS of intermediates of the synthesis of epimeric β-hydroxy carboxylic acids 14 and 17 (Scheme 2)...29

2.1 ^{1}H-NMR (300.13 MHz, DMSO-d_6) of S-trityl-L-cysteine 29

2.2 ^{1}H NMR (300.13 MHz, $CDCl_3$) of Boc-Cys(Trt)-OH (10).. 30

2.3 ^{1}H NMR (300.13 MHz, $CDCl_3$) of 11 31

2.4 ^{1}H NMR (300.13 MHz, $CDCl_3$) of 12 32

2.5. NMR and MS of 13 33

^{1}H NMR (400.13 MHz, $CDCl_3$) of **13**.. 33

^{13}C NMR (100.13 MHz, $CDCl_3$) of **13**.. 34

^{1}H-^{1}H COSY (400.13 MHz, $CDCl_3$) of **13** 35

HSQC (400.13 MHz, $CDCl_3$) of **13** 36

(+)-HRESIMS of **13**: Ion: *m/z*: 558.2303 $[M+Na]^+$ 37

2.6 NMR and MS of 14 38

^{1}H NMR (300.13 MHz, $CDCl_3$) of **14**.. 38

^{13}C NMR (75 MHz, $CDCl_3$) of **14**.. 39

^{1}H-^{1}H COSY (75 MHz, $CDCl_3$) of **14** 40

HSQC (75 MHz, $CDCl_3$) of **14** 41

(+)-HRESIMS of **14**: Ion: *m/z*: 530.1980 ($[M+Na]^+$)... 42

2.7 NMR and MS of 15 43

^{1}H NMR (400 MHz, $CDCl_3$) of **15**.. 43

^{13}C NMR (100.13 MHz, $CDCl_3$) of **15**.. 44

^{1}H-^{1}H COSY (100.13 MHz, $CDCl_3$) of **15** 45

HSQC (100.13 MHz, $CDCl_3$) of **15** 46

^{1}H-^{1}H NOESY of **15** 47

(+)-HRESIMS of **15**: Ion: *m/z*: 484.1563 ($[M+Na]^+$)... 48

2.8 NMR and MS of 16 49

^{1}H NMR (400 MHz, $CDCl_3$) of **16**.. 49

^{13}C NMR (100.13 MHz, $CDCl_3$) of **16**.. 50

^{1}H-^{1}H COSY (400.13 MHz, $CDCl_3$) of **16** 51

HSQC (400.13 MHz, $CDCl_3$) of **16** 52

HRESIMS of **16**: Ion: *m/z*: 558.2302 ($[M+Na]^+$) 53

2.9 NMR and MS of 17 54

^{1}H NMR (400.13 MHz, $CDCl_3$) of **17** ... 54
^{13}C NMR (100.13 MHz, $CDCl_3$) of **17** ... 55
^{1}H-^{1}H COSY (400.13 MHz, $CDCl_3$) of **17** ... 56
HSQC (400.13 MHz, $CDCl_3$) of **17** ... 57
(+)-HRESIMS of **17**: Ion: *m/z*: 530.1968 $[M+Na]^+$ ... 58

2.10 NMR and MS of 18 ... 59
^{1}H NMR (400.13 MHz, $CDCl_3$) of **18** ... 59
^{13}C NMR (100.13 MHz, $CDCl_3$) of **18** ... 60
^{1}H-^{1}H COSY (400.13 MHz, $CDCl_3$) of **18** ... 61
HSQC (400.13 MHz, $CDCl_3$) of **18** ... 62
^{1}H-^{1}H NOESY of **18** ... 63
(+)-HRESIMS of **18**: Ion: *m/z*: 484.1574 $[M+Na]^+$ ... 64

3. NMR and MS of intermediates of the synthesis of thiazole syn epimer 30 (Scheme 3) ... 65

3.1 NMR and MS of compound 19 ... 65
^{1}H NMR (300.13 MHz, $CDCl_3$) of **19** ... 65
^{13}C NMR (75 MHz, $CDCl_3$) of **19** ... 66
^{1}H-^{1}H COSY (300.13 MHz, $CDCl_3$) of **19** ... 67
HSQC (300.13 MHz, $CDCl_3$) of **19** ... 68

3.2 NMR and MS of 20 ... 69
^{1}H NMR (400.13 MHz, $CDCl_3$) of **20** ... 69
^{13}C NMR (100.13 MHz, $CDCl_3$) of **20** ... 70
^{1}H-^{1}H COSY (100.13 MHz, $CDCl_3$) of **20** ... 71
HSQC (400.13 MHz, $CDCl_3$) of **20** ... 72
(+)-HRESIMS of **20**: Ion *m/z*: 510.1710 ($[M+Na]^+$ ... 73

3.3 NMR and MS of 21 ... 74
^{1}H NMR (300.13 MHz, $CDCl_3$) of **21** ... 74
^{13}C NMR (75 MHz, $CDCl_3$) of **21** ... 75
^{1}H-^{1}H COSY (300.13 MHz, $CDCl_3$) of **21** ... 76
HSQC (300.13 MHz, $CDCl_3$) of **21** ... 77
(+)-HRESIMS of **21**: Ion: *m/z*: 490.2035 $[M+H]^+$ ... 78

3.4 NMR and MS of 22 ... 79
^{1}H NMR (400.13 MHz, $CDCl_3$) of **22** ... 79
^{13}C NMR (100.13 MHz, $CDCl_3$) of **22** ... 80
^{1}H-^{1}H COSY (400.13 MHz, $CDCl_3$) of **22** ... 81
HSQC (400.13 MHz, $CDCl_3$) of **22** ... 82
(+)-HRESIMS of **22**: Ion: m/z: 644.2831 $[M+Na]^+$ ... 83

3.5 ^{1}H NMR (300 MHz, $CDCl_3$) of 24 ... 84

3.6 NMR and MS of compound 25 ... 84
^{1}H NMR (300 MHz, $CDCl_3$) of **25** ... 85
^{13}C NMR (75 MHz, $CDCl_3$) of **25** ... 86
(+)-HRESIMS of **25**: Ion: *m/z*: 426.1242 ($[M+Na]^+$) ... 87

3.7 NMR and MS of 27 ... 88
^{1}H NMR (300 MHz, $CDCl_3$) of **27** ... 88
^{13}C NMR (75 MHz, $CDCl_3$) of **27** ... 89
^{1}H-^{1}H COSY (300.13 MHz, $CDCl_3$) of **27** ... 90
HSQC (300.13 MHz, $CDCl_3$) of **27** ... 91

(+)-HRESIMS of **27**: Ion: *m/z*: 765.3179 ([M+H]$^+$) 92

3.8 NMR and MS of 28 ..... **93**

^{1}H NMR (400 MHz, $CDCl_3$) of **28** 93

^{13}C NMR (100.13 MHz, $CDCl_3$) of **28** 94

^{1}H-^{1}H COSY (100.13 MHz, $CDCl_3$) of **28** 95

HSQC (100.13 MHz, $CDCl_3$) of **28** 96

(+)-HRESIMS of **28**: Ion: *m/z*: 721.3173 ([M+H]$^+$) 97

3.9 NMR and MS of 29 ..... **98**

^{1}H NMR (400 MHz, $CDCl_3$) of **29** 98

^{13}C NMR (100.13 MHz, $CDCl_3$) of **29** 99

^{1}H-^{1}H COSY (400.13 MHz, $CDCl_3$) of **29** 100

HSQC (400.13 MHz, $CDCl_3$) of **29** 101

(+)-HRESIMS of **29**: Ion *m/z*: 719.3026 [M+H]$^+$ 102

3.10 NMR and MS of 30 ..... **103**

^{1}H NMR (300.13 MHz, ... 103

^{13}C NMR (75 MHz, CD_3OD) of **30** 104

^{13}C NMR (75 MHz, $CDCl_3$ + CD_3OD) of **30** 105

^{1}H-^{1}H COSY (300.13 MHz, CD_3OD) of **30** 106

HSQC (300.13 MHz, CD_3OD) of **30** 108

(+)-HRESIMS of **30**: Ion: m/z: 263.0529 ([M+H]$^+$) 109

4. NMR and MS of intermediates from the synthesis of 13S-Pcb analogues 6 and 7 and Ga^{+3} complex 32 (Schemes 4 and 5) ***110***

4.1 NMR and MS of 31 ..... **110**

^{1}H NMR (300.13 MHz, $CDCl_3$) of **31** 110

^{13}C NMR (75 MHz, $CDCl_3$) of **31** 111

4.2 NMR and MS of 6a **112**

Table S2: ^{1}H NMR (500.13 MHz) and ^{13}C NMR (125.13 MHz) spectral data of **6a** in CD_2Cl_2 112

^{1}H NMR (500.13 MHz, CD_2Cl_2) of **6a** 113

^{13}C NMR (125.13 MHz, CD_2Cl_2) of **6a** 114

^{1}H-^{1}H COSY (500.13 MHz, CD_2Cl_2) of **6a** 115

HSQC (500.13 MHz, CD_2Cl_2) of **6a** 116

HMBC (500.13 MHz, CD_2Cl_2) of **6a** 117

^{1}H-^{1}H NOESY (500.13 MHz, CD_2Cl_2) of **6a** 118

(+)-HRESIMS of **6a**: Ion: *m/z*: 452.0769 [M+H]$^+$ 119

HPLC chromatogram and UV spectrum of **6a** 120

4.3 NMR and MS of a mixture of 6b and 6c (3:2) **121**

Table S3: ^{1}H NMR and ^{13}C NMR chemical shift list of a mixture of **6b** and **6c** (3:2) in CD_2Cl_2. 121

^{1}H NMR (500 MHz, CD_2Cl_2) of a mixture of **6b** and **6c** (3:2) 122

^{13}C NMR (125.13 MHz, CD_2Cl_2) of a mixture of **6b** and **6c** (3:2) 123

^{1}H-^{1}H COSY (500.13 MHz, CD_2Cl_2) of a mixture of **6b** and **6c** (3:2) 124

HSQC (500 MHz, CD_2Cl_2) of a mixture of **6b** and **6c** (3:2) 125

HMBC (500 MHz, CD_2Cl_2) of a mixture of **6b** and **6c** (3:2) 126

^{1}H-^{1}H NOESY of a mixture of **6b** and **6c** (3:2) 127

(+)-HRESIMS of a mixture of **6b** and **6c** (3:2): Ion: *m/z*: 452.0774 ([M+H]$^+$) 128

4.4 NMR and MS of 7a and 7d **129**

^{1}H NMR (500.13 MHz, CD_3OD) of **7a** and **7d** (0.6:1) 129

^{13}C NMR (500.13 MHz, CD_3OD) of **7a** and **7d** (0.6:1) 130
^{1}H-^{1}H COSY (500.13 MHz, CD_3OD) of **7a** and **7d** (0.6:1) 131
HSQC (500.13 MHz, CD_3OD) of **7a** and **7d** (0.6:1) 132
HMBC (500.13 MHz, CD_3OD) of **7a** and **7d** (0.6:1) 133
^{1}H-^{1}H NOESY (500.13 MHz, CD_3OD) of **7a** and **7d** (0.6:1) 134
^{1}H-^{1}H ROESY (500.13 MHz, CD_3OD) of **7a** and **7d** (0.6:1) 135
(-)-HRESIMS of **7a** and **7d** Ion: *m/z*: 436.0457 $[M-H]^-$ 136
(+)-HRESIMS of **7a** and **7d** Ion: *m/z*: 438.0611 $[M-H]^+$ 136

4.5 NMR and MS of a mixture of 7b and 7c (5:1) 137

Table S4: ^{1}H NMR and ^{13}C NMR chemical shift list of a mixture of **7b**:**7c** (5:1) in CD_3OD 137
^{1}H NMR (500.13 MHz, CD_3OD) of a mixture of **7b** and **7c** (5:1) 138
^{13}C NMR (125 MHz, CD_3OD) of a mixture of **7b** and **7c** (5:1) 139
^{1}H-^{1}H COSY (500.13 MHz, CD_3OD) of a mixture of **7b** and **7c** (5:1) 140
HSQC (500.13 MHz, CD_3OD) of a mixture of **7b** and **7c** (5:1) 141
HMBC (500.13 MHz, CD_3OD) of a mixture of **7b** and **7c** (5:1) 142
^{1}H-^{1}H NOESY (500.13 MHz, CD_3OD) of a mixture of **7b** and **7c** (5:1) 143
^{1}H-^{1}H ROESY (500.13 MHz, CD_3OD) of a mixture of **7b** and **7c** (5:1) 144
(-)-HRESIMS of a mixture of **7b** and **7c** (5:1): Ion: *m/z*: 436.0456 $[M-H]^-$ 145
(+)-HRESIMS of a mixture of **7b:7c** (5:1): Ion: *m/z*: 438.0610 $[M+H]^+$ 145

4.6 NMR and MS of a mixture of 7a-d 146

^{1}H NMR (500.13 MHz, CD_3OD) of **7a-d** 146
^{13}C NMR (125.13 MHz, CD_3COD) of **7a-d** 147
^{1}H-^{1}H COSY (500.13 MHz, CD_3OD) of **7a-d** 148
HSQC (500.13 MHz, CD_2Cl_2) of **7a-d** 149
HMBC (500.13 MHz, CD_2Cl_2) of **7a-d** 150
UV spectrum of **7a-d** 151
(-)-HRESIMS of **7a-d**: Ion: *m/z*: 436.0465 $[M-H]^-$ 152

4.7 NMR and MS of compound of Ga^{+3} complex 32a 153

^{1}H NMR (500.13 MHz, CD_3OD) of **32** 153
^{13}C NMR (125.13 MHz, CD_3OD) of **32a** 154
^{1}H-^{1}H COSY (125.13 MHz, CD_3OD) of **32a** 155
HSQC (500.13 MHz, CD_3OD) of **32a** 156
HMBC (500.13 MHz, CD_3OD) of **32a** 157
^{1}H-^{1}H NOESY of **32a** (CD_3OD) 158
UV spectrum of **32a** 159
(-)-HRESIMS of **32a**: Ion: *m/z*: 501.9485 ($[M-H]^-$) 160
(+)-HRESIMS of **32**: Ion: *m/z*: 503.9636 ($[M+H]^+$), 525.9456 ($[M+Na]^+$) 161

5. NMR and MS of the intermediates from the stereoselective synthesis of thiazole intermediate anti epimer 39 (Scheme 7) 162

5.1 NMR and MS of 34 162

^{1}H NMR (400 MHz, $CDCl_3$) of **34** 162
^{13}C NMR (100.13 MHz, $CDCl_3$) of **34** 163
^{1}H-^{1}H COSY (400.13 MHz, CDCl3) of **34** 164
HSQC (400.13 MHz, $CDCl_3$) of **34** 165
(+)-HRESIMS of **34**: Ion: *m/z*: 556.2133 ($[M+Na]^+$) 166

5.2 NMR and MS of 35 167

^{1}H NMR (300 MHz, $CDCl_3$) of **35** 167

^{13}C NMR (75 MHz, $CDCl_3$) of **35** 168
^{1}H-^{1}H COSY (75 MHz, $CDCl_3$) of **35** 169
HSQC (300.13 MHz, $CDCl_3$) of **35** 170
(+)-HRESIMS of **35**: Ion: *m/z*: 644.2828 $[M+Na]^+$ 171

5.3 NMR and MS of 36 172
^{1}H NMR (400 MHz, $CDCl_3$) of **36** 172
^{13}C NMR (100.13 MHz, $CDCl_3$) of **36** 173
^{1}H-^{1}H COSY (400.13 MHz, $CDCl_3$) of **36** 174
HSQC (400.13 MHz, CDC_{l3}) of **36** 175
(+)-HRESIMS of **36**: Ion: *m/z*: 765.3181 ($[M+H]^+$) 176

5.4 NMR and MS of 37 177
^{1}H NMR (400 MHz, $CDCl_3$) of **37** 177
^{13}C NMR (100.13 MHz, $CDCl_3$) of **37** 178
^{1}H-^{1}H COSY (400.13 MHz, $CDCl_3$) of **37** 179
HSQC (400.13 MHz, $CDCl_3$) of **37** 180
(+)-HRESIMS of **37**: Ion: *m/z*: 721.3170 $[M+H]^+$ 181

5.5 NMR and MS of 38 182
^{1}H NMR (300 MHz, $CDCl_3$) of **38** 182
^{13}C NMR (75 MHz, $CDCl_3$) of **38** 183
^{1}H-^{1}H COSY (300.13 MHz, $CDCl_3$) of **38** 184
HSQC (300.13 MHz, $CDCl_3$) of **38** 185
(+)-HRESIMS of **38**: Ion: *m/z*: 719.3024 $[M+H]^+$ 186

5.6 NMR and MS of 39 187
^{1}H NMR (500.13 MHz, $CDCl_3$) of **39** 187
^{13}C NMR (125.13 MHz, $CDCl_3$) of **39** 188
^{1}H-^{1}H COSY (500.13 MHz, $CDCl_3$) of **39** 189
HSQC (500.13 MHz, $CDCl_3$) of **39** 190
(+)-HRESIMS of **39**: Ion: *m/z*: 263.0520 $[M+H]^+$ 191

6. NMR and MS of the synthesis of (13R) Pcb thiazole analogues 9a-d and Ga^{3+} complexation attempts (Schemes 8 and 9). 192

6.1 NMR spectra of 8a 192
^{1}H NMR (500 MHz; CD_2Cl_2) of **8a** 192
^{13}C NMR (125 MHz) of **8a** 193
^{1}H-^{1}H COSY (500 MHz) of **8a** 194
HSQC (500.13 MHz) of **8a** 195
HMBC (500.13 MHz) of **8a** 196
^{1}H-^{1}H NOESY (500.13 MHz) of **8a** 197
UV spectrum of **8a** 198
(+)-HRESIMS of **8a**: Ion: *m/z*: 474.0594 $[M+Na]^+$ and 452.0774 $[M+H]^+$ 199

6.2 NMR and MS of NMR spectra of the mixture of (9*S*,10*R*,12*R*,13*R*)-8b and (9*S*,10*S*,12*R*,13*R*)-8c. 200
^{1}H NMR (500.13 MHz; CD_2Cl_2) of the mixture of **8b** and **8c** (4:3). 200
^{13}C NMR (125 MHz; CD_2Cl_2) of the mixture of **8b** and **8c** (4:3). 201
^{1}H-^{1}H COSY (500.13 MHz; CD_2Cl_2) of the mixture of **8b** and **8c** (4:3). 202
HSQC (500.13 MHz; CD_2Cl_2) of the mixture of **8b** and **8c** (4:3). 203
HMBC (500.13 MHz; CD_2Cl_2) of the mixture of **8b** and **8c** (4:3). 204

^{1}H-^{1}H NOESY (500.13 MHz; CD_2Cl_2) of the mixture of **8b** and **8c** (4:3) 205
UV spectrum of the mixture **8b** and **8c** 206
(+)-HRESIMS of **8b** and **8c**: Ion: *m/z*: 452.0767 $[M+H]^+$ 207

6.3 NMR spectra of the mixture of 9a and 9d 208
^{1}H NMR (500.13 MHz) in CD_3OD of the mixture **9a** and **9d** (1:2) 208
^{13}C NMR (125 MHz) in CD_3OD of the mixture **9a** and **9d** (1:2) 209
^{1}H-^{1}H COSY (500.13 MHz) in CD_3OD of the mixture **9a** and **9d** (1:2) 210
HSQC (500.13 MHz) in CD_3OD of the mixture **9a** and **9d** (1:2) 211
(+)-HRESIMS of the mixture of **9a** and **9d**: Ion: *m/z*: 438.0610 $[M+H]^+$ 212

6.4 NMR spectra of the mixture of 9b and 9c 213
^{1}H NMR (500.13 MHz) in CD_3OD of the mixture of **9b** and **9c** (3.5:1) 213
^{13}C NMR (125 MHz) in CD_3OD of the mixture of **9b** and **9c** (3.5:1) 214
^{1}H-^{1}H COSY (500.13 MHz) in CD_3OD of the mixture of **9b** and **9c**(3.5:1) 215
HSQC (500.13 MHz) in CD_3OD of the mixture of **9b** and **9c** (3.5:1) 216
^{1}H-^{1}H NOESY (500.13 MHz) in CD_3OD of the mixture of **9b** and **9c** (3.5:1) 217
(-)-HRESIMS of the mixture of **9b** and **9c**: Ion: *m/z*: 436.0468 $[M-H]^-$ 218

7. DFT Calculations 219

7.1 DFT coordinates of 32a 219

7.2 DFT coordinates of 32b 219

7.3 DFT coordinates of 32c 220

7.4 DFT coordinates of 32d 221

7.5 DFT coordinates of 41 222

7.6 DFT-Model of the Ga^{3+} complex 41 223

7.7 DP4+ Analysis of 32a-d 224

1. Synthetic procedures and characterization data for the prepared compounds

1.1 Synthesis of epimeric β-hydroxy carboxylic acids 14 and 17 (Scheme 2)

*Synthesis of **13** and **16**.*

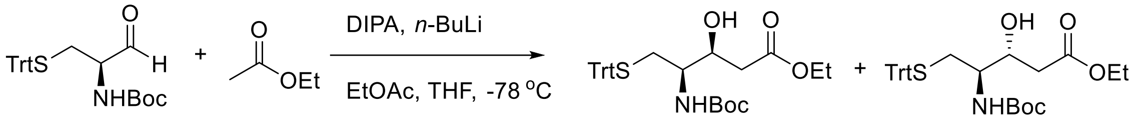

12 **13** **16**

To a solution of DIPA (1 mL, 7.15 mmol, 3.2 eqiv.) in THF (8 mL), *n*-BuLi (2.5 M, 2.86 mL, 7.15 mmol, 3.2 equiv.) was added at –78 °C. After 15 minutes under stirring, ethyl acetate (0.7 mL, 7.15 mmol, 3.2 equiv.) was also added and the solution was stirred for another 30 min at –78 °C. *tert*-butyl (*R*)-(1-oxo-3-(tritylthio)propan-2-yl)carbamate **12** (1.00 g, 2.23 mmol, 1 equiv.) was then added to the resulting enolate solution and stirred for 1 h at the same temperature. Subsequently, the reaction mixture was quenched by addition of saturated aqueous NH_4Cl solution (4 mL) and it was warmed to room temperature. The reaction mixture was then extracted with EtOAc (3 × 30 mL), and the combined organic phases were dried over $MgSO_4$, filtered, and concentrated under reduced pressure. The crude 1:1-mixture was purified by flash column chromatography (SiO_2, hexane:Et_2O =3:1) to afford **13** (0.515 g, 43%) and **16** (0.455 g, 38%), both as a foam. Compound **13** ^{1}H NMR (400 MHz, $CDCl_3$) δ ppm: 7.36-7.34 (m, 6H, Trt), 7.27 – 7.16 (m, 6H, Trt), 7.16 – 7.05 (m, 3H, Trt), 4.66 (d, *J* = 9.4 Hz, 1H, NH), 4.05 (q, *J* = 7.1 Hz, 2H, OEt), 3.97 (d, *J* = 8.7 Hz, 1H, H-3), 3.34 (dd, *J* = 14.7, 7.1 Hz, 1H, H-4), 2.89 (d, *J* = 3.6 Hz, 1H, OH), 2.47 – 2.33 (m, 2H, H-5), 2.30 (d, *J* = 9.9 Hz, 1H, H-2), 2.26 – 2.13 (m, 1H, H-2), 1.34 (s, 9H, Boc), 1.16 (t, *J* = 7.1 Hz, 3H, OEt); ^{13}C NMR (101 MHz, CDCl3) δ ppm: 172.9 (CO_2Et), 155.7 (CO, Boc), 144.8 (C, Trt), 129.7 (CH, Trt), 128.0 (CH, Trt), 126.8 (CH, Trt), 79.6 (C, Boc), 68.1 (CH, C-3), 67.1 (C, Trt), 60.9 (CH_2, OEt), 53.4 (CH, C-4), 38.7 (CH_2, C-2), 34.3 (CH_2, C-5), 28.5 (CH_3,Boc), 14.3 (CH_3, OEt); (+)-LRMS (ESI) *m/z*: 558.23 $[M+Na]^+$, (+)-HRMS (ESI) *m/z*: $[M + Na]^+$ Calcd. for $C_{31}H_{37}NO_5NaS$: 558.2284; Found: 558.2303; $[\alpha]_D^{29}$ = +28.75 (c = 0.083, $CHCl_3$). Compound **16** ^{1}H NMR (400 MHz, $CDCl_3$) δ ppm: 7.37 – 7.31 (m, 6H, Trt), 7.24 – 7.18 (m, 6H, Trt), 7.16 – 7.10 (m, 3H, Trt), 4.59 (d, *J* = 8.2 Hz, 1H, NH), 4.04 (q, *J* = 7.1 Hz, 2H, OEt), 3.82 (bs, 1H, H-3), 3.44 (bs, 1H, H-4), 3.20 (bs, 1H, OH), 2.52 (dd, *J* = 12.5, 7.5 Hz, 1H, H-5), 2.32 (dd, *J* = 12.5, 3.9 Hz, 1H, H-5), 2.25-2.21 (m, 1H, H-2), 2.15 (dd, *J* = 16.5, 9.1 Hz, 1H- H2), 1.35 (s, 9H,Boc), 1.17 (t, *J* = 7.1 Hz, 3H, OEt); ^{13}C NMR (101 MHz, $CDCl_3$) δ ppm: 172.7 (CO_2Et), 155.7 (CO, Boc), 144.7 (C, Trt), 129.7 (CH, Trt), 128.1 (CH, Trt), 126.9 (CH, Trt), 79.8 (C, Boc), 70.1 (CH, C-3), 67.1 (C, Trt), 60.9 (CH_2, OEt), 53.9 (CH, C-4), 38.2 (CH_2, C-2), 32.8 (CH_2, C-5), 28.5 (CH_3, Boc), 14.3 (CH_3, OEt); (+)-LRMS (ESI) *m/z*: 558.23 $[M+Na]^+$, (+)-HRMS (ESI) *m/z*: $[M + Na]^+$ Calcd. for $C_{31}H_{37}NO_5NaS$: 558.2284; Found: 558.2302; $[\alpha]_D^{29}$ = +18.0 (c = 0.17, $CHCl_3$).

*Synthesis of **14**.*

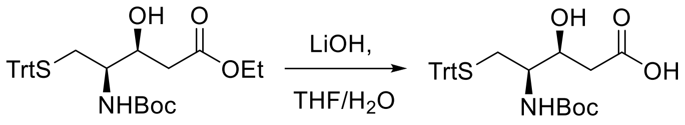

13 **14**

To a solution of ester **13** (400 mg, 0.746 mmol) in 3:1 THF/H_2O (8 mL) at 0 °C, aqueous LiOH (1.2 M mmol, 1.6 mL) was added, and the reaction mixture was stirred at 0 °C for 15 h. After that time, the reaction mixture was quenched with 5% HCl and then extracted with CH_2Cl_2 (15 mL x 3). The combined organic layers were dried over $MgSO_4$, filtered, and concentrated under vacuum to give **14** (379 mg, quantitative). ^{1}H NMR (300 MHz, $CDCl_3$) δ ppm: 7.44-7.41 (m, 6H, Trt), 7.35 – 7.16 (m, 9H, Trt), 4.74 (d, *J* = 9.1 Hz, 1H, NH), 4.01 (d, *J* = 8.2 Hz, 1H, H-3), 3.31 (dd, *J* = 16.8, 9.0 Hz, 1H, H-4), 2.50-2.43 (m, 2H, H-5), 2.40-2.31 (m, 2H, H-2), 1.42 (s, 9H, Boc); ^{13}C NMR (75 MHz, $CDCl_3$) δ ppm: 176.7 (CO_2H), 156.0 (CO, Boc), 144.7 (C, Trt), 129.7 (CH, Trt), 128.0 (CH, Trt), 126.8 (CH, Trt), 80.0 (C, Boc), 68.2 (CH, C-3), 67.1 (C, Trt), 53.5 (CH, C-4), 38.6 (CH_2, C-2), 34.0 (CH_2, C-5), 28.4 (CH_3, Boc). (+)-LRMS (ESI) *m/z*: 530.20 $[M+Na]^+$, (-)-LRMS (ESI) *m/z*: 506.09 $[M-H]^-$, (+)-HRMS (ESI) *m/z*: $[M + Na]^+$ Calcd. for $C_{29}H_{33}NO_5NaS$: 530.1971; Found: 530.1980; $[\alpha]_D^{25}$ = +22.82 (c = 0.165, $CHCl_3$).

*Synthesis of **15**.*

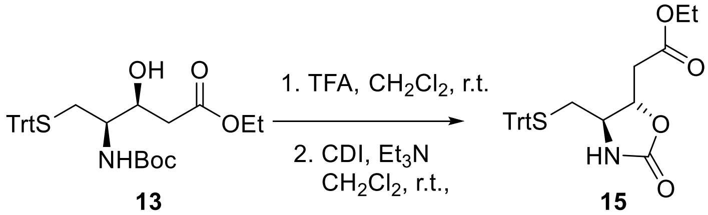

13 **15**

TFA (1 mL) was added to a solution of **13** (30 mg, 0.056 mmol) in CH_2Cl_2 (1 mL). The yellow solution was stirred for 1 h at room temperature and concentrated under reduced pressure. The obtained oil was dissolved in CH_2Cl_2 (2 mL) and concentrated again in *vacuo*. The crude product was used for the next transformation without further purification. Thus, to a solution of the *Boc*-deprotected amine (30 mg, 0.056 mmol) in dry CH_2Cl_2 (3 mL), trimethylamine (20 µL, 0.14 mmol) and carbonyl diimidazole (10 mg, 0.063 mmol) were added and the reaction mixture was stirred for 2 h and concentrated under vacuum. The crude product was purified by flash column chromatography (SiO_2, ethyl acetate/hexane 1:2) to give the oxazolidinone **15** (9 mg, 35%). ^{1}H NMR (400 MHz, $CDCl_3$) δ ppm: 7.44 – 7.40 (m, 6H, Trt), 7.33 – 7.27 (m, 6H, Trt), 7.26 – 7.21 (m, 3H, Trt), 5.02 (b, 1H, NH), 4.38 (td, *J* = 6.4, 4.8 Hz, 1H, H-3), 4.12 (q, *J* = 7.1 Hz, 2H, OEt), 2.89-2.84 (m Hz, 1H, H-4), 2.57 (dd, *J* = 16.5, 6.7 Hz, 1H, H-2), 2.54 – 2.49 (m, 2H, H-5), 2.45 (dd, *J* = 16.5, 6.1 Hz, 2H, H-2), 1.26 – 1.21 (t, *J* = 7.1 Hz, 3H, OEt); ^{13}C NMR (101 MHz, $CDCl_3$) δ ppm: 169.0 (CO_2Et), 157.4 (NCO_2), 144.3 (C, Trt), 129.6 (CH, Trt), 128.3 (CH, Trt), 127.2 (CH, Trt), 77.4 (CH, C-3), 67.5 (C, Trt), 61.3 (CH_2, OEt), 56.3 (CH, C-4), 39.3 (CH_2, C-2), 36.7 (CH_2, C-5), 14.2 (CH_3, OEt); (+)-LRMS (ESI) *m/z*: 484.16 $[M+Na]^+$, (+)-HRMS (ESI) *m/z*: $[M + Na]^+$ Calcd. for $C_{27}H_{27}NO_4NaS$: 484.1553; Found. 484.1563; $[\alpha]_D^{26}$ = -5.95 (c = 0.065, $CHCl_3$).

*Synthesis of **17**.*

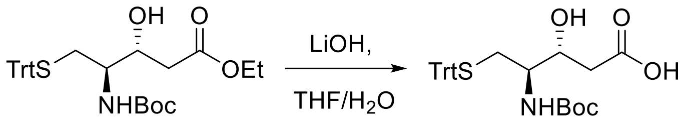

16 **17**

Aqueous LiOH (1.2 M mmol, 2 mL) was added to a solution of the ester **16** (509 mg, 0.95 mmol) in 3:1 THF/H_2O (10 mL) at 0 °C, and the reaction mixture was stirred at 0 °C for 15 hours. After that time, the reaction mixture was quenched with 5% HCl and then, extracted with CH_2Cl_2 (15 mL x 3). The combined organic phases were dried over $MgSO_4$, filtered and the solvent was removed under vacuum to give **17** (482 mg, quantitative). ^{1}H NMR (400 MHz, $CDCl_3$) δ ppm: 7.32 (d, *J* = 7.8 Hz, 6H, Trt), 7.23 – 7.14 (m, 6H, Trt), 7.13-7.09 (m, 3H, Trt), 4.70 (d, *J* = 45.4 Hz, 1H, N-H)), 3.79 (bs, 1H, H-3), 3.40 (bs, 1H, H-4), 2.46 (d, *J* = 7.0 Hz, 1H, H-5), 2.35 – 2.23 (m, 1H, H-5), 2.23 – 1.96 (m, 2H, H-2), 1.33 (s, 9H, Boc); ^{13}C NMR (101 MHz, $CDCl_3$) δ ppm: 176.5 (CO_2H), 156.2 (CO, Boc), 144.6 (CH, Trt), 129.7 (CH, Trt), 128.1 (CH, Trt), 126.9(CH, Trt), 80.4 (C, Boc), 70.1 (CH, C-3), 67.2 (C, Trt), 54.0 (CH, C-4), 37.9 (CH_2, C-2), 32.6, (CH_2, C-5), 28.4 (CH_3, Boc); (+)-LRMS (ESI) *m/z*: 530.20 $[M+Na]^+$; (+)-HRMS (ESI) *m/z*: $[M + Na]^+$ Calcd. for $C_{29}H_{33}NO_5NaS$: 530.1971; Found: 530.1968; $[\alpha]_D^{29}$ = + 19.09 (c = 0.205, $CHCl_3$)

*Synthesis of **18**.*

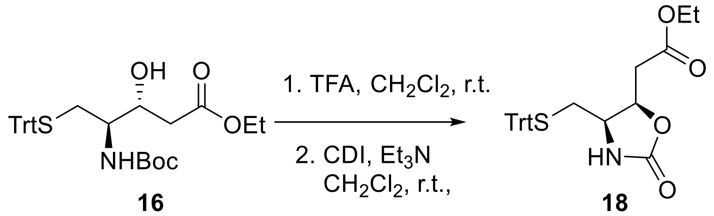

16 **18**

To a solution of **16** (30 mg, 0.056 mmol) in CH_2Cl_2 (1 mL) was added TFA (1 mL). The yellow solution was stirred for 1 h at room temperature and concentrated under vacuum. The resultant oil was dissolved in CH_2Cl_2 (2 mL) and concentrated again under vacuum. The crude product was used for the next transformation without further purification. Thus, trimethylamine (20 µL, 0.14 mmol) and carbonyl diimidazole (10 mg, 0.063 mmol) were added to a solution of the *Boc*-deprotected amine (30 mg, 0.056 mmol) in dry CH_2Cl_2 (3 mL), and the reaction mixture was stirred for 2 h and concentrated under vacuum. The crude product was purified by flash column chromatography (SiO_2, ethyl acetate/hexane 1:2) to give the oxazolidinone **18** (12 mg, 46%). ^{1}H NMR (400 MHz, $CDCl_3$) δ ppm: 7.43 – 7.38 (m, 6H, Trt), 7.33 – 7.28 (m, 6H, Trt), 7.26 – 7.21 (m, 3H, Trt), 5.06 (bs, 1H, NH), 4.84 (td, *J* = 7.4, 6.9 Hz, 1H, H-3), 4.14 – 4.02 (m, 2H, OEt), 3.38 (td, *J* = 8.0, 4.9 Hz, 1H, H-4), 2.63 (dd, *J* = 16.6, 7.7 Hz, 1H, H-2), 2.44 (dd, *J* = 16.6, 6.7 Hz, 1H, H-2), 2.35 (dd, *J* = 12.8, 8.7 Hz, 1H, H-5), 2.29 (dd, *J* = 12.8, 4.9 Hz, 1H, H-5), 1.21 (t, *J* = 7.1 Hz, 3H, OEt); ^{13}C NMR (101 MHz, $CDCl_3$) δ ppm: 169.2 (CO_2Et), 157.5 (NCO_2), 144.1 (C, Trt), 129.5 (CH, Trt), 128.3 (CH, Trt), 127.3 (CH, Trt), 75.1 (CH, C-3), 67.8 (C, Trt), 61.4 (CH_2, OEt), 53.9 (CH, C-4), 34.5 (CH_2, C-2), 32.6 (CH_2, C-5), 14.2 (CH_3, OEt); (+)-LRMS (ESI) *m/z*: 484.16 $[M+Na]^+$, (+)-HRMS (ESI) *m/z*: $[M + Na]^+$ Calcd. for $C_{27}H_{27}NO_4NaS$: 484.1553; Found: 482.1574; $[\alpha]_D^{27}$ = +3.11 (c = 0.09, $CHCl_3$)

1.2 Synthesis of intermediate thiazole syn epimer 30 (Scheme 3)

*Synthesis of **19**.*

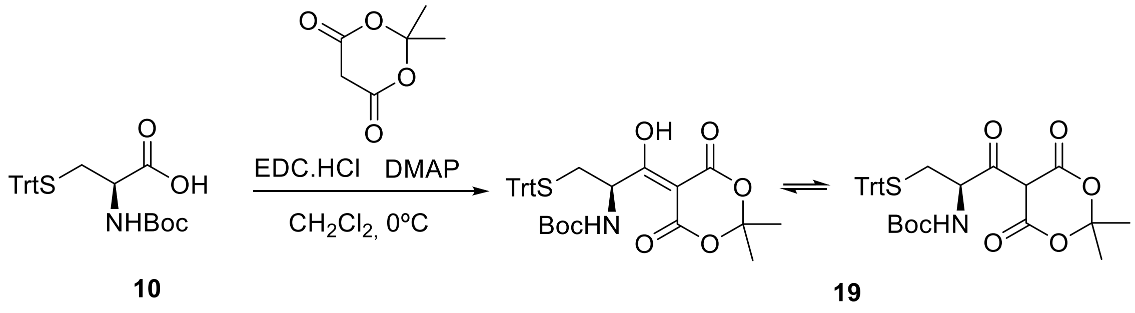

Amino acid Boc-Cys(Trt)-OH (**10**) (4.3 g, 9.18 mmol) was added to a previously cooled solution at 0 °C of Meldrum's acid (1.47 g, 10.2 mmol) and DMAP (1.25 g, 10.2 mmol) in dry CH_2Cl_2 (35 mL). Then, EDC HCl (1.96 mg, 10.2 mmol) was added, and the reaction was stirred at 0 °C for 3 h. After that time, the reaction mixture was diluted with CH_2Cl_2 (70 mL) and then, it was subsequently washed with 0.5M $KHSO_4$ aqueous solution and cold brine (2 x 100 mL), dried over $MgSO_4$, filtered, and concentrated under vacuum. The crude product **19** (5.24 g) was used in the next step without further purification. ^{1}H NMR (300 MHz, $CDCl_3$) δ ppm: 7.56 – 7.34 (m, 8H, Trt), 7.33 – 7.13 (m, 7H, Trt), 5.58 (bs, 1H, H-4), 5.04 (d, *J* = 8.3 Hz, 1H, NH), 2.97 – 2.50 (m, 2H, H-5), 1.72 (s, 6H, Me x 2), 1.42 (s, 9H, Boc); ^{13}C NMR (75 MHz, $CDCl_3$) δ ppm: 193.7 (CO, C-3), 170.8 (CO_2, C-1), 159.5 (CO, Boc), 144.3 (C, Trt), 129.6 (CH, Trt), 128.2 (CH, Trt), 127.1 (CH, Trt), 105.7 (C, CMe_2), 91.4 (C, C-2), 67.3 (C, Trt), 52.2 (CH, C-4), 33.9 (CH_2, C-5), 28.4 (CH_3, Boc), 27.1 (CH_3, Me), 26.9 (CH_3, Me).

*Synthesis of **20**.*

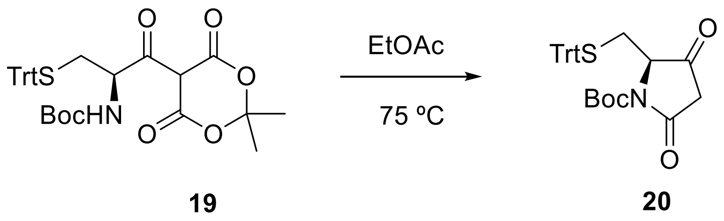

Compound **19** was dissolved in ethyl acetate (35 mL) and refluxed for 1 h. After that time, the reaction mixture was cooled to room temperature and then, the solvent was evaporated under vacuum. The crude product was used in the following transformation without further purification. An aliquot of that material (30 mg) was purified by flash column chromatography (SiO_2, CH_2Cl_2: MeOH gradient from 2% to 5%) to afford **20** as a white solid (15 mg). ^{1}H NMR (400 MHz, $CDCl_3$) δ ppm: 7.48 – 7.05 (m, 15H, Trt), 4.47 (dt, *J* = 4.9, 2.2 Hz, 1H, H-4), 3.34 (dd, *J* = 22.6, 1.6 Hz, 1H, H-2), 3.15 (d, *J* = 22.6 Hz, 1H, H-2), 2.90 (dd, *J* = 11.8, 5.1 Hz, 1H, H-5), 2.55 (dd, *J* = 11.7, 2.4 Hz, 1H, H-5), 1.48 (s, 9H, Boc); ^{13}C NMR (101 MHz, $CDCl_3$) δ ppm: 202.4 (CO, C-3), 167.6 (CON, C-1), 148.6 (CO, Boc), 144.0 (C, Trt), 129.5 CH, Trt), 128.2 (CH, Trt), 127.1 (CH, Trt), 84.5 (C, Boc), 66.7(C, Trt), 66.1 (CH, C-4), 43.4(CH_2, C-2), 32.5 (CH_2, C-5), 28.0 (CH_3, Boc); (+)-HRMS (ESI) *m/z*: $[M + Na]^+$ Calcd. for $C_{29}H_{29}NO_4SNa$ 510.1710, Found: 510.1710; $[\alpha]_D^{24}$ = +21.22 (c = 0.21, $CHCl_3$).

*Synthesis of **21**.*

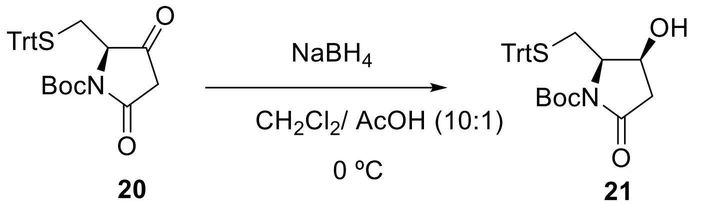

20 **21**

The crude product **20** (4.52 g) was dissolved in 33 mL of a 10:1 mixture of CH_2Cl_2 and acetic acid and cooled to 0 °C. Sodium borohydride (705 mg, 18.5 mmol) was added over 30 min. After stirring at 0 °C for 1 h, cooled saturated $NaHCO_3$ (20 mL) was added. The resulting mixture was extracted with CH_2Cl_2, and the organic layer was washed with brine, dried over $MgSO_4$, filtered, and concentrated under vacuum. Purification of the crude product by flash column chromatography (SiO_2, hexane/EtOAc, 1.5:1), afforded **21** (2.39 g, 52% from **10**) as a white foam. ^{1}H NMR (300 MHz, $CDCl_3$) δ ppm: 7.52 – 7.36 (m, 6H, 9H, Trt), 7.33-7.23 (m, 9H, Trt), 4.41 (dd, *J* = 13.5, 6.6 Hz, 1H, H-3), 4.04 (dd, *J* = 12.4, 6.2 Hz, 1H, H-4), 2.71 – 2.66 (m, 2H, H-5), 2.62-2.59 (m, 1H, H-2), 2.51 (dd, *J* = 17.5, 6.6 Hz, 1H, H-2), 2.35 (bs, 1H, OH), 1.46 (s, 9H, Boc); ^{13}C NMR (75 MHz, $CDCl_3$) δ ppm: 171.7 (CON), 149.5 (CO, Boc), 144.3(C, Trt), 129.6 (CH, Trt), 128.2 (CH, Trt), 127.0 (CH, Trt), 83.5 (C, Boc), 67.2 (C, Trt), 65.0 (CH, C-3), 60.49 (CH, C-4), 40.7 (CH_2, C-2), 30.6 (CH_2, C-5), 28.1 (CH_3, Boc); (+)-LRMS (ESI) *m/z*: 490.20 $[M+H]^+$, 512.19 $[M+Na]^+$, 1001.39 $[2M+Na]^+$; (+)-HRMS (ESI) *m/z*: $[M+H]^+$ Calcd. for $C_{29}H_{32}NO_4S$: 490.2046; Found: 490.2035; $[\alpha]_D^{26}$ = +18.4 (c = 0.395, $CHCl_3$).

*Synthesis of **14**.*

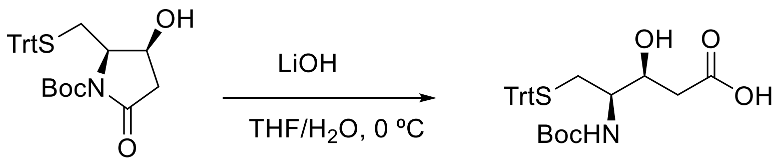

21 **14**

To a solution of **21** (1.032g, 2.11 mmol) in 10 mL of a 4:1 mixture of THF and H_2O at 0 °C, aqueous LiOH (1.2 M mmol, 2 mL) was added, and the mixture was stirred for 1 h. After that time, the solvent was evaporated under vacuum and the residue was diluted with H_2O. The aqueous phase was acidified with 1 M HCl and extracted with CH_2Cl_2 (3x40 mL). The combined organic extracts were dried over $MgSO_4$ and concentrated under vacuum to yield **14** (947 mg, 87%).

*Synthesis of **22**.*

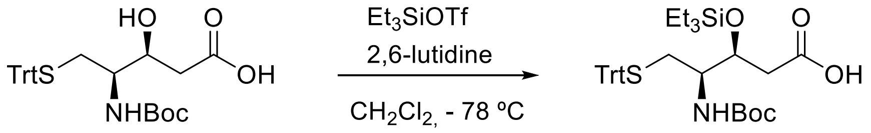

14 **22**

To a solution of hydroxyacid **14** (379 mg, 0.75 mmol) in dry CH_2Cl_2 (10 mL) was added 2,6-lutidine (304 µL, 2.61 mmol) at –78 °C, followed by dropwise addition of TESOTf (422 µL, 1.87 mmol). After 3 h under stirring, the reaction mixture was poured into pH 4 phthalate buffer solution, the layers were separated, and the aqueous layer was extracted with CH_2Cl_2 (x5). The combined organic fractions were

dried over $MgSO_4$, filtered, and concentrated under reduced pressure. The TES ether-acid was purified by flash column chromatography (SiO_2, CH_2Cl_2/MeOH 1% to 10%) to give **22** (340 mg, 73%) as a colourless foam. ^1H NMR (400 MHz, $CDCl_3$) δ ppm: 7.35 – 7.31 (m, 6H, Trt), 7.23 – 7.16 (m, 6H, Trt), 7.16 – 7.10 (m, 3H, Trt), 4.45 (d, *J* = 9.6 Hz, 1H, NH), 3.97 (t, *J* = 6.7 Hz, 1H, H-3), 3.64 – 3.57 (m, 1H, H-4), 2.37 (dd, *J* = 15.6, 6.6 Hz, 1H, H-2), 2.31 (d, *J* = 6.1 Hz, 1H, H-2), 2.25 (dd, *J* = 12.4, 5.4 Hz, 1H, H-5), 2.13 (dd, *J* = 12.3, 8.8 Hz, 1H, H-5), 0.77 (t, *J* = 7.9 Hz, 9H, OTES), 0.48 – 0.30 (m, 6H, OTES); ^{13}C NMR (101 MHz, $CDCl_3$) δ ppm: 176.0 (CO_2H, minor), 175.0 (CO_2H), 156.2 (CO, Boc), 144.8 (C, Trt), 129.7 (CH, Trt), 128.0 (CH, Trt), 126.8 (CH, Trt), 80.7 (C, Boc, minor), 80.1 (C, Boc), 70.6 (CH, C-3), 69.8 (CH, C-3, minor), 66.9 (C, Trt), 54.7 (CH, C-4, minor), 53.8 (CH, C-4), 40.2 (CH_2, C-2) 39.31 (CH_2, C-2 , minor), 35.0 (CH_2, C-5), 28.5 (CH_3, Boc), 7.0 (CH_3, OTES), 5.0 (CH_2, OTES); (+)-LRMS (ESI) *m/z*: 644.28 $[M+Na]^+$; (+)-HRMS (ESI) *m/z*: $[M + Na]^+$ Calcd. for $C_{35}H_{47}NO_5NaSiS$: 644.2836; Found: 644.2831; $[\alpha]_D^{22}$ = -8.306 (c = 0.155, $CHCl_3$).

*Synthesis of **24**.*

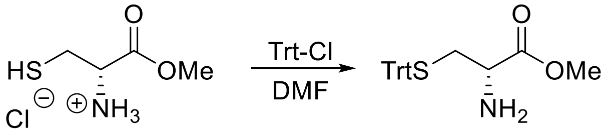

23 **24**

To a solution of D-cysteine methyl ester hydrochloride (1 g, 5.83 mmol) in DMF (10 mL) was added trityl chloride (2.7 g, 9.67 mmol) and the reaction mixture was stirred at room temperature for 2 days. After that time, an aqueous saturated solution of $NaHCO_3$ (20 mL) was added and then, the resulting mixture was extracted with CH_2Cl_2. The organic layer was washed with saturated aqueous NaCl, dried over $MgSO_4$, filtered, and concentrated under vacuum. Purification of the crude product by flash column chromatography (SiO_2, hexane/EtOAc, 1.5:1), afforded **24** (2.07g, 94%). ^1H NMR (300 MHz, $CDCl_3$) δ ppm: 7.51 – 7.38 (m, 6H, Trt), 7.33 – 7.08 (m, 9H, Trt), 3.66 (s, 3H, OMe), 3.20 (dd, *J* = 7.8, 4.8 Hz, 1H), 2.60 (dd, *J* = 12.4, 4.8 Hz, 1H), 2.47 (dd, *J* = 12.4, 7.8 Hz, 1H).

*Synthesis of **25**.*

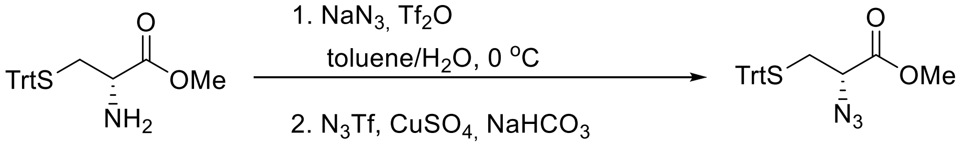

24 **25**

A solution of NaN_3 (772 mg, 11.89 mmol) in H_2O (2 mL) was mixed with toluene (2 mL) and cooled to 0 °C. Tf_2O (1 mL, 5.94 mmol) was added dropwise, and the reaction mixture was stirred vigorously for 30 min at 0 °C, the temperature was raised to 10 °C and the biphasic mixture was stirred for 2 h. The mixture was neutralized with saturated aqueous $NaHCO_3$ solution (1 mL), and the aqueous layer was extracted with toluene (2 x 2 mL). The resulting TfN_3 solution in toluene was used directly without further purification. Compound **24** (1.072 mg, 2.84 mmol), $CuSO_4$ (35 mg, 0.142 mmol) and $NaHCO_3$ (951 mg, 11.33 mmol) were suspended in H_2O (3.5 mL). The mixture was cooled to 0 °C, and the TfN_3 solution in toluene was added dropwise, followed by MeOH (23 mL). The mixture was stirred at room temperature overnight and the solution was then extracted with ethyl acetate (30 mL x 3). The

combined organic layers were washed with saturated aqueous NaCl, dried over $MgSO_4$, filtered, and concentrated under vacuum keeping the temperature below 25 °C. Purification of the crude product by flash column chromatography (SiO_2, hexane/EtOAc, 96:4), afforded **25** (968 mg, 86%) as a white solid. ^1H NMR (500 MHz, $CDCl_3$) δ ppm: 7.47 – 7.42 (m, 6H, Trt), 7.33 – 7.27 (m, 6H, Trt), 7.25 – 7.19 (m, 3H, Trt), 3.70 (s, 3H, OMe), 3.20 (dd, *J* = 8.1, 5.9 Hz, 1H), 2.69 (dd, *J* = 13.4, 5.9 Hz, 1H), 2.56 (dd, *J* = 13.4, 8.1 Hz, 1H); ^{13}C-RMN (75 MHz, $CDCl_3$) δ ppm: 169.4 (CO, C-1), 144.4 (C, Tr), 129.7 (CH, Tr), 128.2 (CH, Tr), 127.1 (CH, Tr), 67.5(C, Tr), 61.5 (C, C-2), 52.9 (CH_3, OMe), 33.3 (CH_2, C-3). (+)-HRMS (ESI) *m/z*: $[M + Na]^+$ Calcd. for $C_{25}H_{21}N_3O_2NaS$: 426.1252; Found: 426.1242

*Synthesis of **26**.*

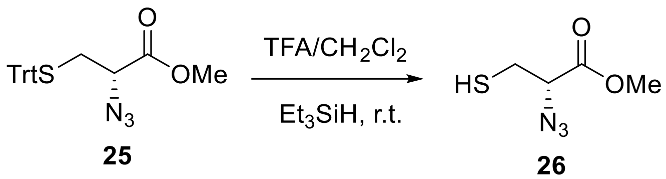

25 **26**

A solution of methyl (*S*)-2-azido-2-methyl-3-(tritylthio)propanoate (**25**) (159 mg, 0.394 mmol, 1.29 equiv.) in dry CH_2Cl_2 (3.5 mL) was treated with TFA (175 µL, 5 vol.%) and Et_3SiH (70 µL, 0.439 mmol, 1.44 equiv.) at room temperature for 1 h. After that time, all volatiles were evaporated to give **26** which was used directly in the next reaction without further purification.

*Synthesis of **27**.*

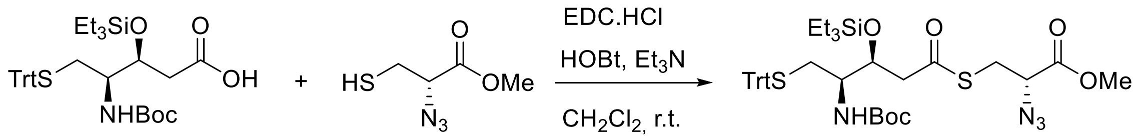

22 **26** **27**

The carboxylic acid **22** (190 mg, 0.305 mmol, 1.0 equiv.) and HOBt (50 mg, 0.366 mmol, 1.2 equiv.) were dissolved in dry CH_2Cl_2 (3.5 mL) and subsequently treated with EDC·HCl (73 mg, 0.381 mmol, 1.25 equiv.) and triethylamine (62 µL, 0.478 mmol, 1.56 equiv.) at 0 °C. The freshly prepared thiol solution of **26** in 1 mL of driy CH_2Cl_2 was added dropwise over after 30 min., and the reaction mixture was stirred at 0 °C for 1 h and at room temperature overnight. The crude reaction mixture was concentrated under vacuum and purified by flash column chromatography (SiO_2, hexane/EtOAc, 92:8), to give thioester **27** as a colourless oil (170 mg, 73 %). ^1H NMR (300 MHz, $CDCl_3$) δ ppm: 7.42-7.40 (m, 6H, Trt), 7.30-7.18 (m, 9H, Trt), 4.48 (d, *J* = 9.3 Hz, 1H, NH), 4.18 (t, *J* = 6.3 Hz, 1H, H-3), 4.08 (dd, *J* = 7.8, 5.5 Hz, 1H, H-2´), 3.82 (s, 3H, OMe), 3.73-3.61 (m, 1H, H-4), 3.49 – 3.31 (m, 1H, H-3´), 3.14 (dd, *J* = 14.0, 7.8 Hz, 1H, H-3´), 2.73-2.58 (m, 2H, H-2), 2.41 – 2.27 (m, 1H, H-5), 2.17 (dd, *J* = 12.0, 8.5 Hz, 1H, H-5), 1.45 (s, 9H, Boc), 0.85 (t, *J* = 7.9 Hz, 9H, OTES), 0.54 – 0.39 (m, 6H, OTES); ^{13}C NMR (75 MHz, $CDCl_3$) δ ppm: 195.6 (COS), 169.2 (CO_2Me), 155.4 (CO, Boc), 144.8 (C, Trt), 129.7 (CH, Trt), 128.0 (CH, Trt), 126.8 (CH, Trt), 79.6 (C, Boc), 70.1 (CH, C-3), 66.7 (C, Trt), 61.4 (CH, C-2´), 53.7 (CH, C-4), 53.1 (CH_3, OMe), 48.9 (CH_2, C-2), 34.7 (CH_2, C-5), 30.1 (CH_2, C-3´), 28.5 (CH_3, Boc), 7.0 (CH_3, OTES), 4.9 (CH_2, OTES); (+)-LRMS (ESI) *m/z*: 787.30 $[M+Na]^+$, 765.31 $[M+H]^+$; (+)-HRMS (ESI) *m/z*: $[M+H]^+$ Calcd. for $C_{39}H_{53}N_4O_6SiS_2$: 765.3170; Found: 765.3179; $[\alpha]_D^{26}$ = +34.63 (c =0.175, $CHCl_3$).

*Synthesis of **28**.*

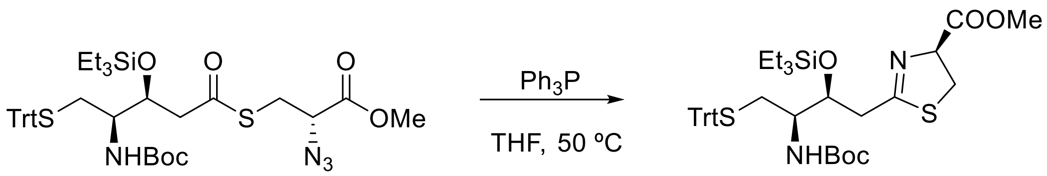

27 **28**

Azido(thio)ester **27** (315 mg, 0.41 mmol) was dissolved in dry THF (7 mL) and was treated dropwise at 0 °C with PPh_3 (162 mg, 0.617 mmol, 1.5 equiv.) in THF (2 mL). After 45 min stirring at 0 °C, the reaction mixture was warmed to 50 °C and stirred for 16 h. The solvent was then evaporated, and the residue was purified by flash column chromatography (SiO_2, hexane/EtOAc, 7:1), obtaining thiazoline **28** (267 mg, 90%) as a colorless oil. ^{1}H NMR (400 MHz, $CDCl_3$) Rotamers: δ ppm: 7.36 – 7.29 (m, 6H, Trt), 7.2-7.18 (m, 6H, Trt), 7.15 – 7.08 (m, 3H, Trt), 4.93 (t, *J* = 9.4 Hz, 1H, H-2´), 4.49 (d, *J* = 9.4 Hz, NH, major), 4.44 (d, *J* = 9.5 Hz, NH, minor). 4.06-4.01 (m, 1H, H-3), 3.72 (s, 3H, OMe), 3.72–3.73 (m, 1H, H-4), 3.50-3.40 (m, 2H, H-3´), 2.55 (dd, *J* = 14.3, 6.2 Hz, 1H, H-2), 2.48 (dd, *J* = 14.3, 5.8 Hz, 1H, H-2), 2.28 (dd, *J* = 12.1, 4.6 Hz, 1H, H-5), 2.16 – 2.06 (m, 1H, H-5), 1.37 (s, 9H, Boc), 0.78 (t, *J* = 7.9 Hz, 9H, OTES), 0.46 – 0.35 (m, 6H, OTES); ^{13}C NMR (101 MHz, $CDCl_3$) Rotamers: δ ppm: 171.5 (CO_2Me), 171.4 (C, C-1), 155.42 (CO, Boc), 144.9 (C, Trt), 129.7 (CH, Trt), 128.0 (CH, Trt), 126.7 (CH, Trt), 79.4 (C, Boc), 78.0 (CH, C-2´), 71.9 (CH, C-3 minor), 71.7 (CH, C-3), 66.6 (C, Trt), 53.8 (CH,C-4), 53.6 (CH, C-4, minor), 52.9 (CH_3, OMe, minor), 52.8 9 (CH_3, OMe) , 39.5 (CH_2,C-2 , minor) , 39.4 (CH_2,C-2), 35.7 (CH_2, C-3´), 34.8 (CH_2, C-5, minor), 34.6 (CH_2, C-5), 28.5 (CH_3, Boc), 7.0 (CH_3, OTES), 5.1 (CH_2, minor, OTES), 5.0 (CH_2, minor, OTES); (+)-LRMS (ESI) *m/z*: 743.30 $[M+Na]^+$, 721.32 $[M+H]^+$; (+)-HRMS (ESI) *m/z*: $[M+H]^+$ Calcd. for $C_{39}H_{53}N_2O_5SiS_2$: 721.3159, Found: 721.3173; $[\alpha]_D^{27}$ = +2.67 (c = 0.15, $CHCl_3$).

*Synthesis of **29**.*

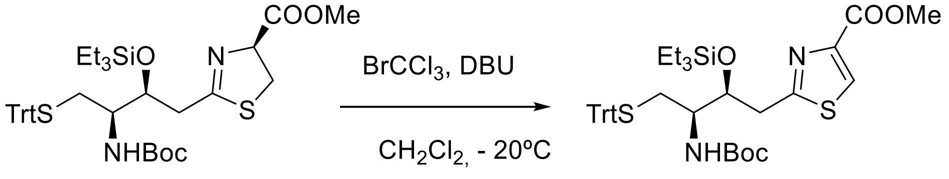

28 **29**

DBU (60 µL, 0.410 mmol) was added to a solution of **28** (108 mg, 0.149 mmol) in dry CH_2Cl_2 (7 mL) and the resulting solution was stirred at –20 °C. After 5 min, $BrCCl_3$ (23 µL, 0.235 mmol) was added. The solution was slowly warmed to room temperature and stirred for 30 min, then cooled to 0 °C and treated with pH 4 phthalate buffer solution. The layers were separated and the aqueous layer was extracted with CH_2Cl_2. The combined organic extracts were dried over $MgSO_4$, filtered, and concentrated under vacuum. Purification by flash column chromatography (SiO_2, hexane/EtOAc 3:1) afforded thiazole **29** as a colorless oil (107 mg, 99%). ^{1}H NMR (400 MHz, $CDCl_3$) δ ppm: 7.99 (d, *J* = 4.8 Hz, 1H, H-3´), 7.41 – 7.27 (m, 6H, Trt), 7.25 – 7.16 (m, 6H, Trt), 7.16 – 7.08 (m, 3H, Trt), 4.44 (d, *J* = 9.4 Hz, 1H, NH), 4.10 (dt, *J* = 6.2, 3.1 Hz, 1H, H-3), 3.86 (s, 3H, OMe), 3.71-3.65 (m, 1H, H-4), 3.10 – 2.92 (m, 2H, H-2), 2.28 (dd, *J* = 12.4, 5.0 Hz, 1H, H-5), 2.17 – 1.99 (m, 1H, H-5), 1.38 (s, 9H, Boc), 0.73 (t, *J* = 7.9 Hz, 9H), 0.31 (q, *J* = 7.9 Hz, 6H, OTES); ^{13}C NMR (75 MHz, $CDCl_3$) δ ppm: 167.7 (C, C-1), 161.9 (CO_2Me), 155.3 (CO, Boc), 146.4 (C, C-2´), 144.7 (C, Trt), 129.6 (CH, Trt), 127.9 (CH, Trt, C-3´), 126.7 (CH, Trt), 79.50 (C, Boc), 73.0 (CH, C-3), 66.6 (C, Trt), 53.6 (CH, C-4), 52.4 (CH_3, OMe), 38.2 (CH_2, C-2), 34.3 (CH_2, C-5), 28.4 (CH_3, Boc), 6.9 (CH_3, OTES), 4.9 (CH_2, OTES); (+)-LRMS (ESI) *m/z*: 741.28 $[M+Na]^+$, 719.30

$[M+H]^+$; (+)-HRMS (ESI) *m/z*: $[M+H]^+$ $C_{39}H_{51}N_2O_5SiS_2$: 719.3003; Found: 719.3026; $[\alpha]_D^{27}$ = +4.9 (c = 0.1, $CHCl_3$).

Synthesis of ***30***.

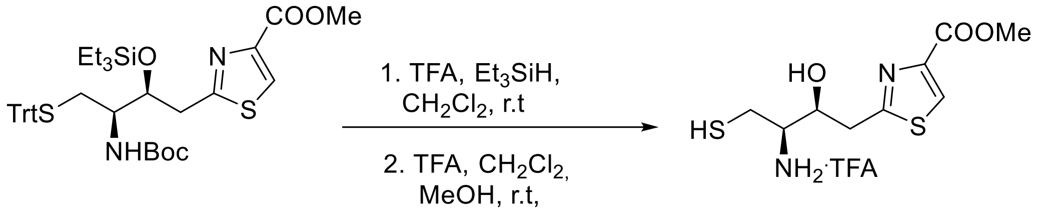

29 **30**

Compound **29** was dissolved in deoxygenated CH_2Cl_2 (6 mL) and treated with TFA (300 µL, 5 %, v/v) and Et_3SiH (17 µL, 0.105 mmol, 2 equiv.) at room temperature for 1 h. All volatiles were evaporated at room temperature. The residue was then redissolved in deoxygenated CH_2Cl_2 (2.5 mL) and MeOH (0.5 mL) and treated with TFA (375 µL, 12.5 %, v/v). After stirring for 1 h, all volatiles were evaporated at room temperature. The resulting residue was redissolved in H_2O (10 mL) and washed with hexane (10 mL) and Et_2O (10 mL). The aqueous phase was concentrated under reduced pressure to give compound **30** (15 mg, 83%) as a white foam which was used in the next step without any further purification. ^1H NMR (300 MHz, CD_3OD) δ ppm: 8.34 (s, 1H, H-3´), 4.27 (dt, *J* = 7.8, 4.6 Hz, 1H, H-3), 3.92 (s, 3H, OMe), 3.48 – 3.16 (m, 3H,2 x H-2, H-4), 2.96 (dd, *J* = 14.5, 5.7 Hz, 1H, H-5), 2.83 (dd, *J* = 14.5, 6.8 Hz, 1H, H-5); ^{13}C NMR (75 MHz, CD_3OD) δ ppm: 169.2 (C, C-1), 163.1 (CO_2Me), 147.0 (C, C-2´), 129.9 (CH, C-3´), 69.1 (CH, C-3), 58.4 (CH, C-4), 52.8 (CH_3, OMe), 38.3 (CH_2, C-2), 25.1 (CH_2, C-5); (+)-LRMS (ESI) *m/z*: 263.05 $[M+H]^+$, (+)-HRMS (ESI) *m/z*: $[M+H]^+$ Calcd. for $C_9H_{15}N_2O_3S_2$: 263.0518; Found: 263.0529; $[\alpha]_D^{28}$ = -3.4 (c = 0.05, MeOH).

1.3 Synthesis of 13S Pcb thiazole analogues 6 and 7 and Ga^{+3} complex 32 (Schemes 4 and 5)

Synthesis of ***6a-d***.

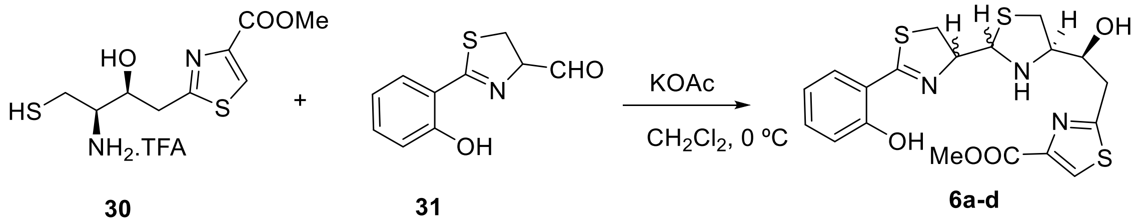

30 **31** **6a-d**

To a suspension of amino thiol **30** (15 mg, 0.041mmol) and potassium acetate (4 mg, 0.044 mmol) in deoxygenated CH_2Cl_2 (2 mL) at 0°C was added aldehyde **31** (9 mg, 0.042 mmol) in deoxygenated CH_2Cl_2 (2 mL). This suspension was stirred 16 h (overnight) at room temperature in the dark, after which it was diluted with CH_2Cl_2 (5 mL) and mili-Q H_2O was added. The layers were separated, and the aqueous layer was extracted with CH_2Cl_2 (2 × 5 mL). The organic layers were collected, dried ($MgSO_4$), filtered and evaporated under reduced pressure. The residue (18 mg) was separated by HPLC into two main fractions. HPLC conditions: Semi-Prep HPLC Discovery column HS-F5 (25 cm x 10 mm, 5 µm) with a mobile phase consisting of a 5 min gradient from 30 % to 55% CH_3CN in H_2O (v/v), followed by a 15 min isocratic at 55%, then 5 min gradient from 55% to 100% and 5 min from 100% to 30%, and finally 5 min gradient from 100% to 30%, and 10 min isocratic at 30%, using a flow rate of 4 mL/min (UV

detector operating at λ = 214 and 265 nm.). First fraction with R_t = 14.99 min and second fraction with R_t = 17.16 min, both eluted with 55% of CH_3CN in H_2O, were concentrated under vacuum to give 4.4 mg and 3.0 mg, respectively. Spectroscopic analysis of both fractions identified compound **6a** in the first fraction and a mixture of compounds **6b** and **6c** in a *ca* 3:2 ratio. 3.3 mg of fraction 1 were re-purified by Semi-Prep HPLC Discovery column HS-F5 (25cm x 10 mm, 5 µm) with a mobile phase consisting of a 10 min gradient from 30 % to 50% CH_3CN in H_2O (v/v), then 15 min at 50%, then 5 min from 50% to 100%, then 5 min at 100% CH_3CN, followed by 5 min from 100% to 30%, and finally 5 min at 30%, all at a flow rate of 4 mL/min (UV detector operating at λ =214 and 265 nm). The fraction eluted with t_R = 19.01 min (corresponding to 50% of CH_3CN in H_2O) was concentrated under vacuum to afford 1.4 mg of **6a**.

(9*R*,10*R*,12*R*,13*S*)-6a ^{1}H NMR (500 MHz, CD_2Cl_2) δ ppm: 8.09 (s, 1H, H-16), 7.41 (dd, *J* = 7.7, 1.4 Hz, 1H, H-5), 7.36 (t, *J* = 7.7 Hz, 1H, H-3), 6.97 (bd, *J* = 7.7 Hz, 1H, H-2), 6.87 (t, *J* = 7.7 Hz, 1H, H-4), 4.96 (ddd, *J* = 8.8, 7.8, 6.8 Hz, 1H, H-9), 4.75 (d, *J* = 6.8 Hz, 1H, H-10), 4.24 (dt, *J* = 9.1, 3.6, 3.6 Hz, 1H, H-13), 3.88 (s, OMe), 3.55 (dd, *J* = 11.3, 8.8 Hz, 1H, H-8), 3.38 (dd, *J* = 11.3, 7.8 Hz, 1H, H-8), 3.38 (dd, *J* = 17.0, 9.1 Hz, 1H, H-14), 3.30 (dd, *J* = 17.0, 3.6 Hz, 1H, H-14), 3.30 (ddd, *J* = 9.9, 6.1, 3.6 Hz, 1H, H-12), 3.05 (dd, *J* = 9.9, 6.1 Hz, 1H, H-11), 2.93 (t, *J* = 9.9 Hz, 1H, H-11); ^{13}C NMR (126 MHz, CD_2Cl_2) δ ppm: 173.37 (C, C-7), 168.76 (C, C-15), 161.92 (CO_2Me), 159.59 (C, C-1), 146.74 (C, C-17), 133.67 (CH, C-3), 131.08 (CH, C-5), 128.00 (CH, C-16), 119.27 (CH, C-4), 117.41 (CH, C-2), 116.53 (C, C-6), 80.72 (CH, C-9), 72.66 (CH, C-10), 70.37 (CH, C-13), 69.10 (CH, C-12), 52.53 (CH_3, OMe), 39.42 (CH_2, C-14), 36.02 (CH_2, C-11), 34.95 (CH_2, C-8); (+)-LRMS (ESI) *m/z*: 452.08 $[M+H]^+$, 474.06 $[M+Na]^+$; (+)-HRMS (ESI) *m/z*: $[M+H]^+$ Calcd. for $C_{19}H_{22}N_3O_4S_3$: 452.0766; Found: 452.0769; $[\alpha]_D^{21}$ = +46.6 (c = 0.02, CH_2Cl_2).

The mixture of compounds **6b** and **6c** in *ca* 3:2 ratio were identified in the fraction 2. **9*S*,10*R*,12*R*,13*S*)-6b** ^{1}H NMR (500 MHz, CD_2Cl_2) δ ppm: 8.10 (s, 1H, H-16), 7.42 (dd, *J* = 7.7, 1.4 Hz, 1H, H-5), 7.37 (t, *J* = 7.7 Hz, 1H, H-3), 6.96 (bd, *J* = 7.7 Hz, 1H, H-2), 6.88 (t, *J* = 7.7 Hz, 1H, H-4), 4.99 (ddd, *J* = 8.8, 8.8, 5.5 Hz, 1H, H-9), 4.91 (d, *J* = 5.5 Hz, 1H, H-10), 4.21 (bs, 1H, H-13), 3.88 (s, OMe), 3.49 (dd, *J* = 10.8, 8.8 Hz, 1H, H-8), 3.37 (m, 1H, H-8), 3.36 – 3.31 (m, 2H, H-14), 3.32 (dd, *J* = 9.8, 6.0 Hz, 1H, H-12), 3.07 (dd, *J* = 9.8, 6.0 Hz, 1H, H-11), 2.94 (t, *J* = 9.8 Hz, 1H, H-11); ^{13}C NMR (126 MHz, CD_2Cl_2) δ ppm: 173.37 (C, C-7), 168.58 (C, C-15), 161.87 (CO_2Me), 159.49 (C, C-1), 146.57 (C, C-17), 133.57 (CH, C-3), 130.93 (CH, C-5), 128.05 (CH, C-16), 119.19 (CH, C-4), 117.34 (CH, C-2), 116.46 (C, C-6), 81.72 (CH, C-9), 72.39 (CH, C-10), 70.41 (CH, C-13), 69.27 (CH, C-12), 52.51 (CH_3, OMe), 39.26 (CH_2, C-14), 36.22 (CH_2, C-11), 34.16 (CH_2, C-8); (+)-LRMS (ESI) *m/z*: 452.08 $[M+H]^+$, 474.06 $[M+Na]^+$; (+)-HRMS (ESI) *m/z*: $[M + H]^+$ Calcd. for $C_{19}H_{22}N_3O_4S_3$: 452.0766, Found: 452.0774.

(9*S*,10*S*,12*R*,13*S*)-6c ^{1}H NMR (500 MHz, CD_2Cl_2) δ ppm: 8.09 (s, 1H, H-16), 7.42 (dd, *J* = 7.7, 1.4 Hz, 1H, H-5), 7.37 (t, *J* = 7.7 Hz, 1H, H-3), 6.96 (bd, *J* = 7.7 Hz, 1H, H-2), 6.88 (t, *J* = 7.7 Hz, 1H, H-4), 4.83 (ddd, *J* = 8.7, 8.2, 7.9 Hz, 1H, H-9), 4.69 (d, *J* = 8.2 Hz, 1H, H-10), 4.21 (bs, 1H, H-13), 3.88 (s, OMe), 3.55 (dd, *J* = 10.9, 7.9 Hz, 1H, H-8), 3.38 (m, 1H, H-8), 3.36 – 3.31 (m, 2H, H-14), 3.43 (dd, *J* = 10.9, 5.9 Hz, 1H, H-c) [illegible])9 (dd, *J* = 9.8, 5.9 Hz, 1H, H-11), 2.93 (t, *J* = 9.8 Hz, 1H, H-11); ^{13}C NMR (126 MHz, CD_2Cl_2) δ ppm: 173.43 (C, C-7), 168.71 (C, C-15), 161.87 (CO_2Me), 159.49 (C, C-1), 146.57 (C, C-17), 133.63 (CH, C-3), 130.84 (CH, C-5), 128.07 (CH, C-16), 119.16 (CH, C-4), 117.31 (CH, C-2), 116.54 (C, C-6), 81.72 (CH, C-9), 79.93 (CH, C-10), 71.78 (CH, C-13), 69.63 (CH, C-12), 52.52 (CH_3, OMe), 39.05 (CH_2, C-14), 36.76 (CH_2, C-11), 35.49 (CH_2, C-8). (+)-LRMS (ESI) *m/z*: 452.08 $[M+H]^+$, 474.06 $[M+Na]^+$, (+)-HRMS (ESI) *m/z*: $[M + H]^+$ Calcd. for $C_{19}H_{22}N_3O_4S_3$: 452.0766, Found: 452.0774.

*Synthesis of **7a-d**.*

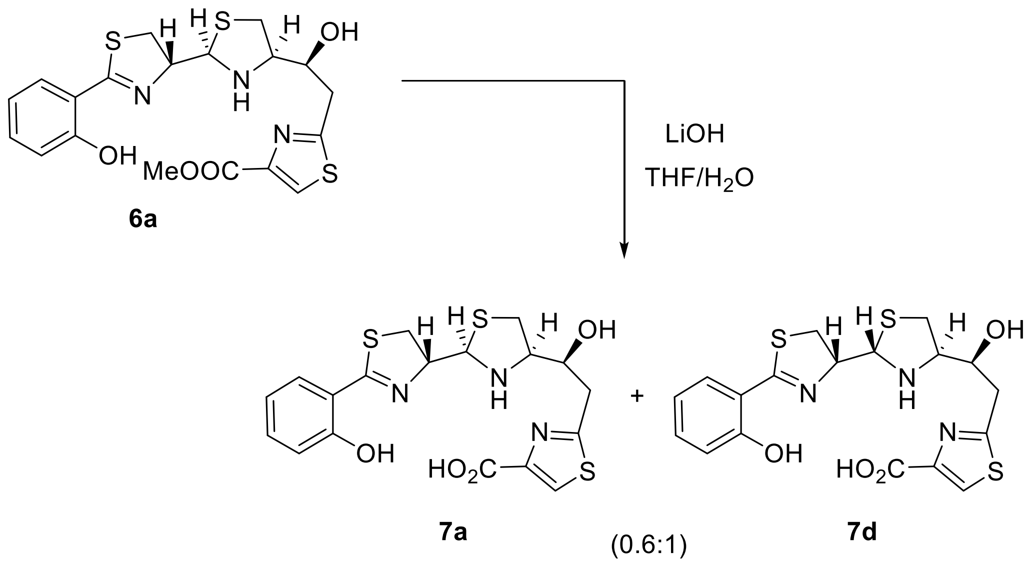

Compound **6a** (1.7 mg, 0.003 mmol) was dissolved in THF:Mili-Q H_2O (0.4 mL: 0.1 mL) and LiOH (0.5 mg, 0.021 mmol) was added at 0°C. After stirring for 1h, the reaction mixture was quenched by the addition of NH_4Cl in mili-Q H_2O (x mg, 0.2 mL), the solvents were concentrated under vacuum and the residue (8.6 mg) was characterized a mixture of carboxylic acids **7a** and **7d** in a *ca* ratio 0.6: 1. The residue was passed through a Hypersep C18 (200 mg) cartridge which was eluted with following mixtures of mili-Q H_2O and CH_3CN 9:1, 1:1 and 0:1. The fraction eluted with a mixture of H_2O and CH_3CN (9:1) was concentrated under vacuum to afford 1.27 mg as a mixture of **7a** and **7d**. (-)-HRMS (ESI) *m/z*: $[M-H]^-$ Calcd. for $C_{18}H_{18}N_3O_4S_3$: 436.0464; Found: 436.0457; (+)-HRMS (ESI) *m/z*: $[M+H]^+$ Calcd. for $C_{18}H_{20}N_3O_4S_3$: 438.0610; Found: 438.0611.

(9*R*,10*R*,12*R*,13*S*)-7a ^1H NMR (500 MHz, CD_3OD) δ ppm: 7.97 (s, 1H, H-16), 7.43 (dd, *J* = 8.0, 1.4 Hz, 1H, H-5), 7.39 (m, 1H, H-3), 6.94 (bd, *J* = 8.2 Hz 1H, H-2), 6.92 (m, 1H, H-4), 4.98 (u.s., 1H, H-9), 4.67 (d, *J* = 7.5 Hz, 1H, H-10), 4.17 (m, 1H, H-13), 3.67 (dd, *J* = 11.4, 8.6 Hz, 1H, H-8), 3.38 (dd, *J* = 11.4, 8.4 Hz, 1H, H-8), 3.26 (m, 1H, H-12), 3.35-3.29 (m, 2H, H-14), 3.02 (dd, *J* = 9.8, 6.0 Hz, H-11), 2.86 (t, *J* = 9.8 Hz, H-11); ^{13}C NMR (126 MHz, CD_3OD) δ ppm: 174.07 (C, C-7), 169.28 (CO_2H, C-18), 168.41 (C, C-15), 160.10 (C, C-1), 153.28 (C, C-17), 134.39 (CH, C-3), 131.75 (CH, C-5), 125.45 (CH, C-16), 120.11 (CH, C-4), 117.92 (CH, C-2), 117.32 (C, C-6), 81.83 (CH, C-9), 73.82 (CH, C-10), 71.28 (CH, C-13), 70.37 (CH, C-12), 40.36 (CH_2, C-14), 36.34 (CH_2, C-11), 35.72 (CH_2, C-8).

(9*R*,10*S*,12*R*,13*S*)-7d ^1H NMR (500 MHz, CD_3OD) δ 7.79 (s, 1H, H-16), 7.41 (dd, *J* = 8.0, 1.4 Hz, 1H, H-5), 7.37 (m, 1H, H-3), 6.92 (m, 1H, H-2), 6.91 (m, 1H, H-4), 4.99 (d, u.s., 1H, H-10), 4.85 (u.s., 1H, H-9), 4.14 (m, 1H, H-13), 3.50 (dd, *J* = 11.0, 8.0 Hz, 1H, H-8), 3.40 (dd, *J* = 11.0, 9.5 Hz, 1H, H-8), 3.40 (m, 1H, H-12), 3.35-3.29 (m, 2H, H-14), 3.07 (dd, *J* = 9.9, 5.9 Hz, H-11), 2.87 (dd, *J* = 9.9, 9.0 Hz, H-11); ^{13}C NMR (126 MHz, CD_3OD) δ 174.34 (C, C-7), 168.96 (CO_2H, C-18), 168.21 (C, C-15), 160.24 (C, C-1), 153.19 (C, C-17), 134.38 (CH, C-3), 131.48 (CH, C-5), 125.33 (CH, C-16), 120.03 (CH, C-4), 117.95 (CH, C-2), 117.49 (C, C-6), 82.97 (CH, C-9), 72.76 (CH, C-10), 70.91 (CH, C-13), 68.26 (CH, C-12), 40.20 (CH_2, C-14), 37.16 (CH_2, C-11), 34.64 (CH_2, C-8);

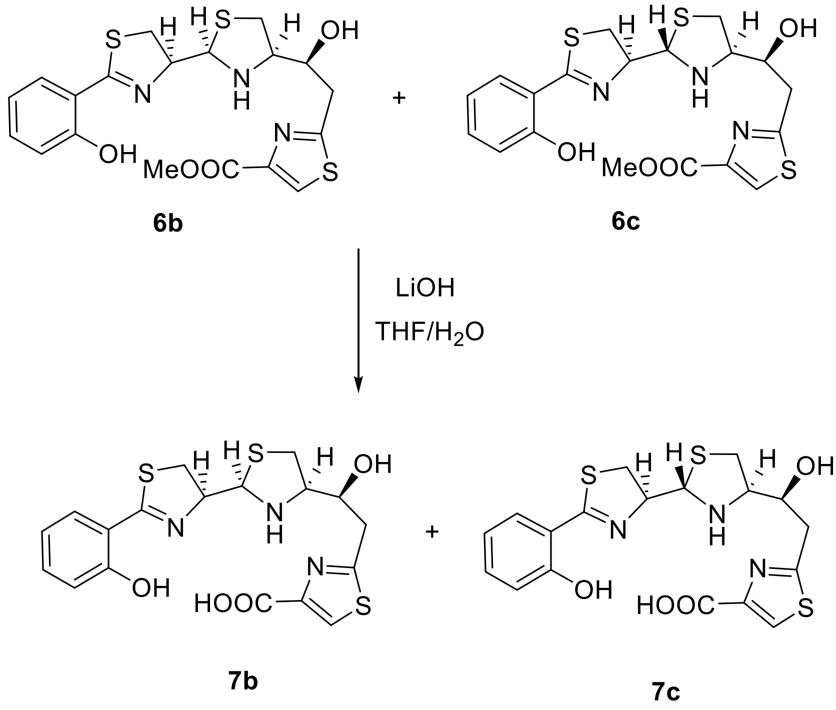

The mixture of compounds **6b** and **6c** (1.7 mg, 0.003 mmol) was dissolved in THF:Mili-Q H_2O (0.4 mL: 0.1 mL) and then, LiOH (0.6 mg, 0.025 mmol) was added at 0°C. After stirring for 1 h, the reaction mixture was quenched by the addition of NH_4Cl in mili-Q H_2O (4 mg, 0.2 mL), the solvents were concentrated under vacuum and the residue was characterized as a mixture of carboxylic acids **7b** and **7c** in a *ca* 5:1 ratio. The residue was passed through a Hypersep C18 (200 mg) cartridge which was eluted with following mixtures of mili-Q H_2O and CH_3CN 9:1, 1:1 and 0:1. The fractions eluted with a mixture of H_2O and CH_3CN (9:1) were concentrated under vacuum to afford 0.98 mg as a mixture of **7b** and **7c**. (-)-HRMS (ESI) *m/z*: $[M - H]^-$ Calcd. For $C_{18}H_{18}N_3O_4S_3$: 436.0465; Found: 436.0456; (+)-HRMS (ESI) *m/z*: $[M + H]^+$ Calcd. for $C_{18}H_{20}N_3O_4S_3$: 438.0610; Found: 438.0610.

(9*S*,10*R*,12*R*,13*S*)-7b ^1H NMR (500 MHz, CD_3OD) δ ppm: 7.95 (s, 1H, H-16), 7.43 (dd, *J* = 8.0, 1.0 Hz, 1H, H-5), 7.38 (bt, *J* = 8.0 Hz, 1H, H-3), 6.94 (dd, *J* = 8.0, 1.0 Hz, 1H, H-2), 6.91 (t, *J* = 8.0 Hz, 1H, H-4), 5.11 (m, 1H, H-9), 4.99 (u.s., 1H, H-10), 4.16 (dt, *J* = 8.3, 4.3, 4.3 Hz, 1H, H-13), 3.56 (dd, *J* = 10.9, 8.8 Hz, 1H, H-8), 3.42 (dd, *J* = 10.9, 9.2 Hz, 1H, H-8), 3.38 (m, 1H, H-14), 3.30 (m, 1H, H-14), 3.33 (u.s., 1H, H-12), 3.03 (dd, *J* = 9.9, 5.9 Hz, 1H, H-11), 2.87 (t, *J* = 9.9 Hz, 1H, H-11); ^{13}C NMR (126 MHz, CD_3OD) δ ppm: 174.13 (C, C-7), 169.25 (CO_2H, C-18), 168.45 (C, C-15), 160.17 (C, C-1), 153.40 (C, C-17), 134.39 (CH, C-3), 131.66 (CH, C-5), 125.35 (CH, C-16), 120.11 (CH, C-4), 117.93 (CH, C-2), 117.35 (C, C-6), 80.67 (CH, C-9), 73.16 (CH, C-10), 71.31 (CH, C-13), 70.36 (CH, C-12), 40.36 (CH_2, C-14), 36.39 (CH_2, C-11), 34.11 (CH_2, C-8);

(9*S*,10*S*,12*R*,13*S*)-7c ^1H NMR (500 MHz, CD_3OD) δ ppm: 7.97 (s, 1H, H-16), 7.43 (dd, *J* = 8.0, 1.4 Hz, 1H, H-5), 7.39 (m, 1H, H-3), 6.93 (m, 1H, H-2), 6.70 (m, 1H, H-4), 4.79 (m, 1H, H-9), 4.67 (d, *J* = 9.1 Hz 1H, H-10), 4.11 (m, 1H, H-13), 3.59 (dd, *J* = 10.9, 8.4 Hz, 1H, H-8), 3.43 (m, 1H, H-8), 3.39 (m, 1H, H-12), 3.04 (m, 1H, H-14), 2.85 (m, 1H, H-14), 3.05 (m, 1H, H-11), 2.85 (m, 1H, H-11); ^{13}C NMR (126 MHz, CD_3OD) δ ppm: 174.13 (C, C-7), 169.25 (CO_2H, C-18), 168.45 (C, C-15), 160.17 (C, C-1), 153.40 (C, C-17), 134.26 (CH, C-3), 131.54 (CH, C-5), 125.35 (CH, C-16), 120.04 (CH, C-4), 117.93 (CH, C-2), 117.88 (C, C-6), 83.78 (CH, C-9), 72.99 (CH, C-10), 70.77 (CH, C-13), 68.28 (CH, C-12), 40.39 (CH_2, C-14), 37.31 (CH_2, C-11), 35.95 (CH_2, C-8).

*Synthesis of **32a**.*

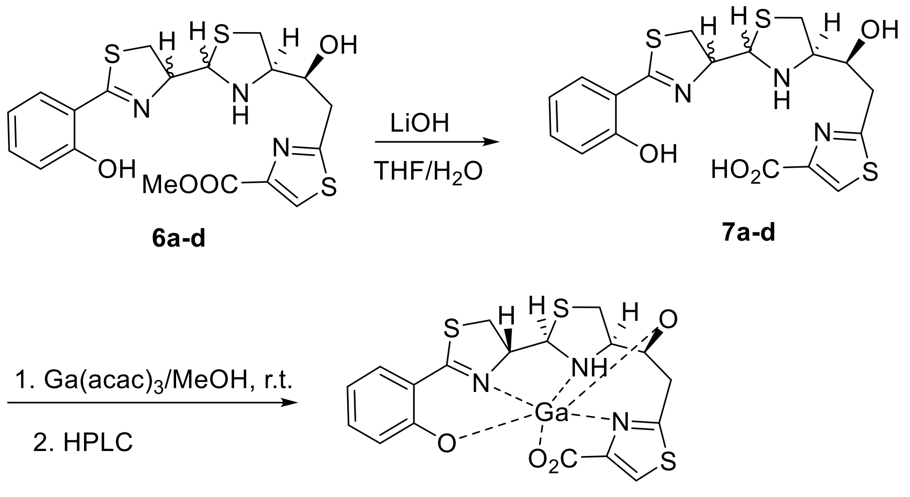

6a-d **7a-d** **32a**

To a solution of crude **6a-d** (51 mg, 0.113 mmol) in THF:Mili-Q H_2O (2 mL: 0.5 mL) was added LiOH (7 mg, 0.297 mmol) at 0ºC. After stirring for 1 h, the reaction mixture was quenched by the addition of NH_4Cl in mili-Q H_2O (24 mg, 0.2 mL). The solvents were concentrated under vacuum to give a mixture of four isomers **7a-d**. 4.8 mg of the crude mixture **7a-d** was solved in CH_3OH (0.7 mL) and then, treated with gallium acetylacetonate (2.5 mg, 0.007 mmol) and stirred for 1 h. After that time, the solvent was concentrated under vacuum to give a residue which was submitted to analytical and semi preparative HPLC to give 0.8 mg of **32** as pure compound.

Analytical conditions: Analytical HPLC Discovery column HS-F5 (4.6 nm x 100 mm, 5 µm) with a mobile phase consisting of 5 min at 10%, 5 min gradient from 10 % to 20% CH_3CN in H_2O (v/v), then 10 min at 20%, then 5 min from 20% to 100%, then 5 min at 100% CH_3CN, followed by 5 min from 100% to 10%, and finally 5 min at 10%, all at a flow rate of 1 mL/min (UV detector operating at λ = 220 nm, 265 nm). The fraction eluted with R_t = 13 min was identified as **32a**.

Semipreparative conditions: Semi-Prep HPLC Discovery column HS-F5 (25 cm x 10 mm, 5 µm) with a mobile phase consisting of 10 min at 10%, 5 min gradient from 10 % to 20% CH_3CN in H_2O (v/v), then 20 min at 20%, then 5 min from 20% to 100%, then 5 min at 100% CH_3CN, followed by 5 min from 100% to 10%, and finally 5 min at 10%, at a flow rate of 3 mL/min (UV detector operating at λ = 220 nm). The fraction eluted with R_t = 25.8 min (corresponding to 20% of CH_3CN in H_2O) was identified as **32a**.

Compound **32a** ^{1}H NMR (500 MHz, CD_3OD) δ ppm: 8.12 (s, 1H, H-16), 7.46 (dd, *J* = 8.0, 1.7 Hz, 1H, H-5), 7.36 (ddd, *J* = 8.7, 7.1, 1.8 Hz, 1H H-3), 6.81 (dd, *J* = 8.6, 0.8 Hz, 1H, H-2), 6.72 (ddd, *J* = 8.1, 7.1, 1.1 Hz, 1H, H-4), 4.81 (d, *J* = 10.1 Hz, 1H, H-10), 4.68 (ddd, *J* = 13.4, 10.0, 7.6 Hz, 1H, H-9), 4.43 (dd, *J* = 4.1, 2.0 Hz, 1H, H-13), 3.76 (dd, *J* = 10.7, 7.2 Hz, 1H, H-12), 3.66 (dd, *J* = 10.9, 7.6 Hz, 1H, H-8), 3.58 (dd, *J* = 12.7, 7.0 Hz, 1H, H-11), 3.55 (dd, *J* = 18.3, 4.6 Hz, 1H, H-14), 3.34 (dd, *J* = 18.2, 2.3 Hz, 1H, H-14), 3.33 – 3.32 (m, 1H, H-8), 3.13 (dd, *J* = 12.6, 10.8 Hz, 1H, H-11); ^{13}C NMR (126 MHz, CD_3OD) δ ppm: 180.7 (C, C-7), 173.0 (CO_2H), 167.7 (C, C-1), 164.5 (C, C-15), 147.5 (C, C-17), 137.2 (CH, C-3), 133.1 (CH, C-5), 124.1 and 123.9 (CH, C-2 and C-16), 117.8 (CH, C-4), 116.8 (C, C-6), 77.2 (C, C-9), 72.5 (C, C-13), 70.3 and 70.2 (CH, C-10, C-12), 39.0 and 38.9 (CH_2, C-11, C-14), 35.1 (CH_2, C-8); (+)-LRMS (ESI) *m/z*: 525.94 $[M+Na]^+$; (-)- LRMS (ESI) *m/z*: 501.93 $[M-H]^-$; (-)-HRMS (ESI) *m/z*: $[M-H]^-$ 501.9485/503.9477 Calcd. for $C_{18}H_{15}{}^{69}GaN_3O_4S_3$: 501.9486; Found: 501.9485.

1.4 Stereoselective synthesis of thiazole intermediate epimer 39 (Scheme 7)

*Synthesis of **34**.*

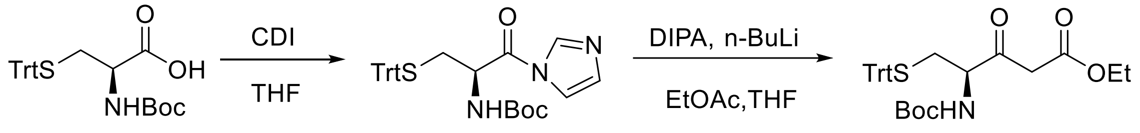

10 **33** **34**

To a solution of STrt-Boc-Cys (**10**) (0.1 g, 0.215 mmol) in anhydrous THF (3 mL) at 0 °C, was added CDI (2625 mg, 0,26 mmol). The reaction mixture was stirred at 0 °C for half a hour, and then at 20 °C for 2 hours. To a solution of freshly distilled DIPA (1.45 mL, 10.37 mmol, 3.2 eqiv.) in THF (12 mL) was added *n*-BuLi (4.15 mL, 10.37 mmol, 2.5 M, 3.2 equiv.) at –78 °C and the resulting solution was stirred for 15 min. Ethyl acetate (1.39 mL, 10.37 mmol) was added via syringe, and after stirring for 1 hour, the solution of the activated acid **33** in THF was added. After 45 minutes, the reaction mixture was quenched with saturated aqueous NH_4Cl (6 mL) and was extracted with EtOAc, dried ($MgSO_4$), filtered, and the solvent removed. The residue was purified by flash column chromatography (SiO_2, 5-25% EtOAc/hexanes) to obtain the β–keto ester **34** as colorless oil (50 mg, 43%). ^{1}H NMR (400 MHz, $CDCl_3$) δ ppm: 7.43-7.41 (m, 6H), 7.33 – 7.27 (m, 6H), 7.25 – 7.18 (m, 3H), 5.05 (d, *J* = 7.8 Hz, 1H, NH), 4,18-4.14 (m, 1H, H-4), 4.13 (q, *J* = 7.1 Hz, 2H, OEt), 3.35 (d, *J* = 16.1 Hz, 1H, H-2), 3.29 (d, *J* = 16.1 Hz, 1H, H-2), 2.72 (dd, *J* = 12.8, 4.5 Hz, 1H, H-5), 2.53 (dd, *J* = 12.8, 6.9 Hz, 1H, H-5), 1.45 (s, 9H, Boc), 1.23 (t, *J* = 7.1 Hz, 3H, OEt); ^{13}C NMR (101 MHz, $CDCl_3$) δ ppm: 200.6 (CO), 166.8 (CO_2Et), 155.2 (CO, Boc), 144.3 (C, Trt), 129.7 (CH, Trt), 128.2 (CH, Trt), 127.0 (CH, Trt), 80.4 (C, Boc), 67.2 (C, Trt), 61.5 (CH_2, OEt), 58.8 (CH, C-3), 46.1 (CH_2, C-2), 32.8 (CH_2, C-5), 28.4 (CH_3, Boc), 14.8 (OCH_3, Et); (+)-LRMS (ESI) *m/z*: 556.21 $[M+Na]^+$; (+)-HRMS (ESI) *m/z*: $[M+Na]^+$ Calcd. for $C_{31}H_{35}NO_5NaS$: 556.2128; Found: 556.2133; $[\alpha]_D^{24}$ = -3.81 (c = 0.11, $CHCl_3$).

*Synthesis of **13** and **16**.*

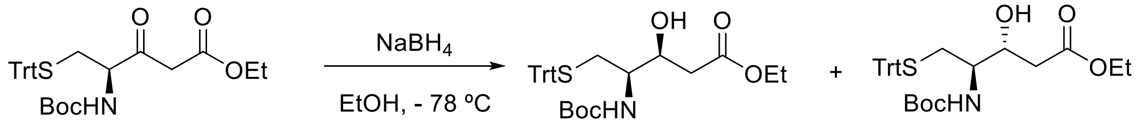

34 **13** **16**

To a solution of **34** (10 mg, 0.019 mmol, 1 equiv) in EtOH (1 mL) at –78 °C, was added $NaBH_4$ (1.5 mg, 0.037 mmol). The reaction was stirred at –78 °C. After 2 h, the reaction mixture was quenched by dropwise addition of NH_4Cl aq solution. The mixture was concentrated under vacuum and CH_2Cl_2/water (1:1, 10 mL) were added. The layers were separated, and the aqueous layer was extracted with CH_2Cl_2 (2 x 10 mL). The combined organic layers were dried over $MgSO_4$ and concentrated in *vacuo* (10 mg). Analysis of the ^{1}H-NMR spectrum of the crude reaction shows a 15:85 ratio of **13** and **16**.

*Synthesis of **35**.*

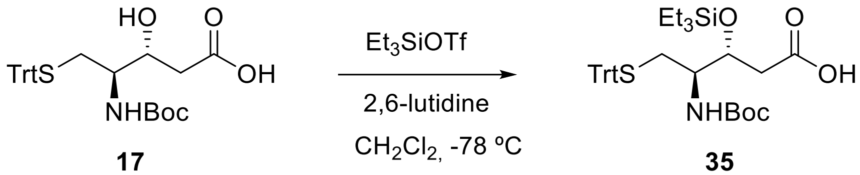

17 **35**

To a solution of hydroxyacid **17** (473 mg, 0.931 mmol) in dry CH_2Cl_2 (12 mL) was added 2,6-lutidine (0.379 mL, 3.26 mmol) at –78 °C, followed by dropwise addition of TESOTf (0.526 mL, 2.33 mmol) and the reaction mixture was stirred for 3 h. The solution was poured into pH 4 phthalate buffer solution. The layers were separated, the aqueous layer was extracted with CH_2Cl_2 (x 5) and the combined organic fractions were dried ($MgSO_4$), filtered and concentrated under reduced pressure. The triethylsilyl ether-acid was purified by flash column chromatography (SiO_2, CH_2Cl_2/MeOH 1% to 10%) to give **35** as a colourless foam (317 mg, 55%). ^1H NMR (300 MHz, $CDCl_3$) δ ppm: 7.43-7.41 (m, Trt), 7.32-7.20 (m, 10H, Trt), 4.56 (bs, 1H, NH), 4.08 (bs, 1H, H-3), 3.56 (bs, 1H, H-4), 2.45-2.35 (m, 2H, H-5), 2.34-2.24 (m, 2H, H-2), 1.46 (s, 9H, Boc), 0.88 (t, *J* = 7.9 Hz, 9H, OTES), 0.53 (dd, *J* = 15.1, 7.5 Hz, 6H, OTES); ^{13}C NMR (75 MHz, $CDCl_3$) δ ppm: 175.8 (CO_2H), 155.5 (CO, Boc), 144.8 (C, Trt), 129.7 (CH, Trt), 128.1 (CH, Trt), 126.8 (CH, Trt), 79.8 (C, Boc), 71.0 (CH, C-3), 67.0 (C, Trt), 54.7 (CH. C-4), 39.9 (CH_2, C-2), 32.5 (CH_2, C-5), 28.5 (CH_3, Boc), 7.0 (CH_3, OTES), 5.0 (CH_2, OTES); (+)-LRMS (ESI) *m/z*: 644.28 $[M+Na]^+$; (+)-HRMS (ESI) *m/z*: $[M+Na]^+$ Calcd. for $C_{35}H_{47}NO_5NaSiS$: 644.2836; Found: 644.2828 $[M+Na]^+$; $[\alpha]_D^{27}$ = +4.69 (c = 0.115, $CHCl_3$)

*Synthesis of **36**.*

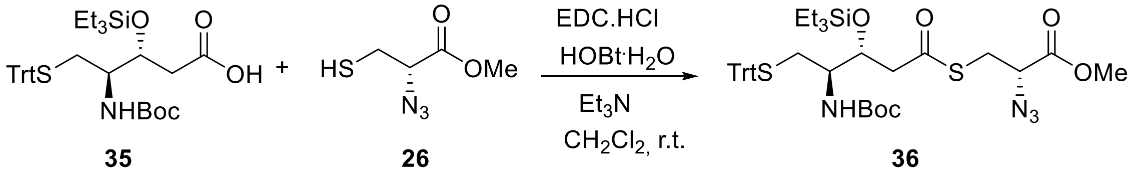

35 **26** **36**

The carboxylic acid **35** (238 mg, 0.328 mmol, 1.0 equiv.) and HOBt (62 mg, 0.459 mmol, 1.2 equiv.) were dissolved in CH_2Cl_2 (7 mL) and subsequently treated with EDC·HCl (92 mg, 0.478 mmol, 1.25 equiv.) and triethylamine (120 µL, 0.460 mmol, 2.4 equiv.) at 0 °C. The freshly prepared thiol solution (2 mL) of **26** was added dropwise after 30 minutes and the reaction mixture was stirred at 0 °C for 1 h and at room temp overnight. The crude reaction mixture was concentrated under vacuum. After flash column chromatography (SiO_2, hexane/EtOAc, 92:8), thioester **36** was isolated as a colourless oil (193 mg, 66 %). ^1H NMR (400 MHz, $CDCl_3$) δ ppm: 7.37 – 7.30 (m, 6H, Trt), 7.27 – 7.17 (m, 6H, Trt), 7.17 – 7.10 (m, 3H, Trt), 4.39 (d, *J* = 5.7 Hz, 1H, NH), 4.09 (dd, *J* = 10.9, 5.4 Hz, 1H, H-3)), 3.98 (dt, *J* = 7.8, 5.0 Hz, 1H, H-2´), 3.73 (s, 3H.OMe), 3.42 (bs, 1H, H-4), 3.29 (dd, *J* = 13.9, 5.7 Hz, 1H, H-3´), , 3.03 (dd, *J* = 13.9, 7.9 Hz, 1H, H-3´), 2.52 – 2.36 (m, 2H, H-2), 2.35 – 2.26 (m, 1H, H-5), 2.26 – 2.19 (m, 1H, H-5), 1.36 (s, 9H, Boc), 0.79 (t, *J* = 7.9 Hz, 9H), 0.41 (dt, *J* = 8.3, 4.8 Hz, 6H); ^{13}C NMR (101 MHz, $CDCl_3$) δ ppm: 195.4 (COS), 169.1 (CO_2Me), 155.1 (CO, Boc), 144.8 (C, Trt), 129.7 (CH, Trt), 128.1 (CH, Trt), 126.9 (CH, Trt), 79.6 (C, Boc), 70.7 (CH, C-3), 67.0 (C, Trt), 61.4 (CH, C-2´), 54.8 (CH, C-4), 53.1 (CH_3, OMe), 49.2 (CH_2, C-2), 32.3 (CH_2, C-5), 30.1 (CH_2, C-3´), 28.5 (CH_3, Boc), 7.0 (CH_3, OTES), 5.0 (CH_2, OTES); (+)-LRMS (ESI) *m/z*: 787.30 $[M+Na]^+$, 765.32 $[M+H]^+$; (+)-HRMS (ESI) *m/z*: $[M+H]^+$ Calcd. For $C_{39}H_{53}N_4O_6SiS_2$: 765.3170; Found: 765.3181; $[\alpha]_D^{24}$ = +21.71 (c = 0.105, $CHCl_3$).

*Synthesis of **37**.*

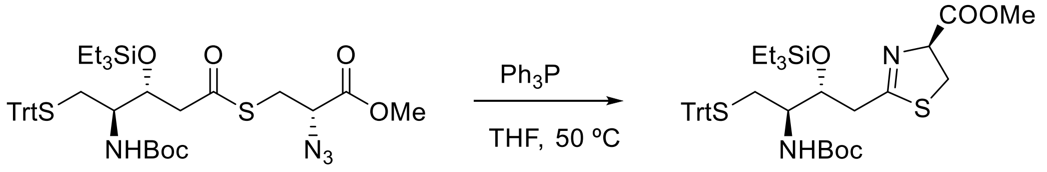

36 **37**

The azido (thio)ester **36** (264 mg, 0.345 mmol) was dissolved in dry THF (0.5 mL) and was treated dropwise at 0 °C with PPh_3 (135 mg, 0.517 mmol, 1.5 equiv.) in THF (1.2 mL). After 45 min stirring at 0 °C, the reaction mixture was warmed to 50 °C and stirred for 16 h. The solvent was evaporated, and the residue was purified by flash column chromatography (SiO_2, hexane/EtOAc, 8:1) **37** (185 mg, 74%). ^{1}H NMR (400 MHz, $CDCl_3$) δ ppm: 7.37 – 7.29 (m, 6H, Trt), 7.25 – 7.17 (m, 6H, Trt), 7.15 – 7.06 (m, 3H, Trt), 4.96 (dt, *J* = 11.6, 5.8 Hz, 1H, H-2´), 4.57 (d, *J* = 8.1 Hz, 1H, NH), 4.09 (d, *J* = 4.8 Hz, 1H, H-3), 3.72 (s, 3H, OMe), 3.53 (bs, 1H, H-4), 3.48 (dd, *J* = 11.2, 8.6 Hz, 1H, H-3´), 3.40 (dd, *J* = 11.2, 9.7 Hz, 1H, H-5, H-3´), 2.46 (dd, *J* = 15.0, 6.4 Hz, 1H, H-2), 2.40 (ddd, *J* = 15.2, 5.9, 1.4 Hz, 1H, H-2), 1.36 (s, 9H, Boc), 0.86 – 0.73 (m, 9H, OTES), 0.50 – 0.35 (m, 6H, OTES); ^{13}C NMR (101 MHz, $CDCl_3$) δ ppm: 171.6 (CO_2Me), 171.4 (C, C-1), 155.2 (CO, Boc), 144.9 (C, Trt), 129.7 (CH, Trt), 128.0 (CH, Trt), 126.8 (CH, Trt), 79.3 (C, Boc), 77.8 (CH, C-2´), 72.2 (CH, C-3), 66.8 (C, Trt), 54.5 (CH, C-4), 52.8 (CH_3, OMe), 39.9 (CH_2, C-2), 35.7 (CH_2, C3´), 32.4 (CH_2, C-5), 28.6 (CH_3, Boc), 7.0 (CH_3, OTES), 5.0 (CH_2, OTES); (+)-LRMS (ESI) *m/z*: 743.30 $[M+H]^+$, 721.32 $[M+H]^+$; (+)-HRMS (ESI) *m/z*: $[M+H]^+$ Calcd. for $C_{39}H_{53}N_2O_5SiS_2$: 721.3159; Found: 721.3170; $[\alpha]_D^{21}$ = -19.68 (c = 0.5, $CHCl_3$).

*Synthesis of **38**.*

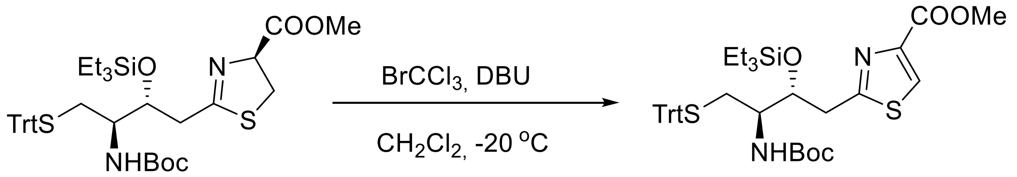

37 **38**

To a solution of **37** (185 mg, 0.256 mmol) in dry CH_2Cl_2 (12 mL) was added DBU (103 µL, 0.692 mmol) and the resulting solution was stirred at –20 °C. After 5 min, $BrCl_3C$ (40 µL, 0.410 mmol) was added. The solution was slowly warmed to room temperature and stirred for 30 min, then cooled to 0 °C and treated with pH 4 phthalate buffer solution. The layers were separated, and the aqueous layer was extracted with CH_2Cl_2. The combined organic extracts were dried ($MgSO_4$), filtered, and concentrated. Purification by flash column chromatography (SiO_2, hexane/EtOAc 6:1) afforded thiazole **38** as a colorless oil (167 mg, 91%). ^{1}H NMR (400 MHz, $CDCl_3$) δ ppm: 8.00 (s, 1H,H-3´), 7.35 – 7.28 (m, 6H, Trt), 7.22-7.18 (m, 6H, Trt), 7.16 – 7.08 (m, 3H, Trt), 4.43 (d, *J* = 6.6 Hz, 1H, NH), 4.11 (bs, 1H, H-3), 3.86 (m, 3H, OMe), 3.40 (ddd, *J* = 13.5, 8.6, 4.9 Hz, 1H, H-4), 3.00 (dd, *J* = 15.0, 6.9 Hz, 1H, H-2), 2.89 (dd, *J* = 15.0, 4.5 Hz, 1H, H-2), 2.44 – 2.21 (m, 2H, H-5), 1.34 (s, 9H, Boc), 0.74 (t, *J* = 7.9 Hz, 9H, OTES), 0.32 (q, *J* = 7.9 Hz, 6H, OTES); ^{13}C NMR (101 MHz, $CDCl_3$) δ ppm: 167.9 (C, C-1), 162.0 (CO_2Me), 155.1 (CO, Boc), 146.3 (C, C-2´), 144.8 (C, Trt), 129.7 (CH, Trt), 128.2 (CH, C-3´), 128.0 (CH, Trt), 126.8 (CH, Trt), 79.5 (C, Boc), 73.2 (CH, C-3), 66.9 (C, Trt), 54.5 (CH, C-4), 52.4 (CH_3, OMe), 38.5 (CH_2, C-2), 32.2 (CH_2, C-5), 28.5 (CH_3, Boc), 7.0 (CH_3, OTES), 4.9 (CH_2, OTES); (+)-LRMS (ESI) *m/z*: 741.28 $[M+Na]^+$, 719.30 $[M+H]^+$; (+)-

HRMS (ESI) *m/z*: $[M + Na]^+$ Calcd. for $C_{39}H_{51}N_2O_5SiS_2$: 719.3003; Found: 719.3024; $[\alpha]_D^{20}$ =-7.41 (c = 0.125, $CHCl_3$).

*Synthesis of **39**.*

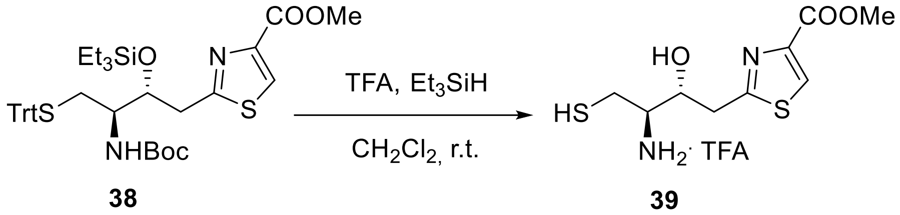

Compound **38** (51 mg, 0.071 mmol, 1.0 equiv.) was dissolved in deoxygenated CH_2Cl_2 (8 mL) and treated with TFA (400 µL, 5 vol.-%) and Et_3SiH (14 µL, 0.085 mmol, 1.2 equiv.) at room temperature for 2 h. All volatiles were evaporated at room temperature. The residue was dissolved in H_2O (10 mL) and washed with Et_2O (10 mL x 2). The aqueous phase was concentrated under reduced pressure to give without any further purification compound **39** (21 mg, 78%) as a white foam. ^1H NMR (500 MHz, $CDCl_3$) δ ppm: 8.28 (bs, 1H), 8.05 (s, 1H, H-3´), 5.40 (bs, 1H), 4.57 (bs, 1H, H-3), 3.89 (s, 3H, OMe), 3.59-3.56 (m, 1H, H-4), 3.35-3.30 (m, 2H, H-2), 2.95 (bs, 2H, H-5), 1.87 (bs. 1H, SH); ^{13}C NMR (126 MHz, $CDCl_3$) δ ppm: 168.1 (C, C-1), 162.3 (CO_2Me), 145.8 (C, C-2´), 128.6 (CH, C-3´), 69.1 (CH, C-3), 58.0 (CH, C-4), 52.8 (CH_3, OMe), 36.1 (CH_2, C-2), 22.4 (CH_2, C-5); (+)-LRMS *m/z*: 523.08 $[disulfide+H]^+$, 263.05 $[M+H]^+$; (+)-HRMS *m/z*: $[M+H]^+$ Calcd. for $C_9H_{15}N_2O_3S_2$: 263.0518; Found: 263.0520; $[\alpha]_D^{23}$ = -9.86 (c =0.075, $CHCl_3$).

*Synthesis of compounds **8a-d** (Schemes 8 and 9).*

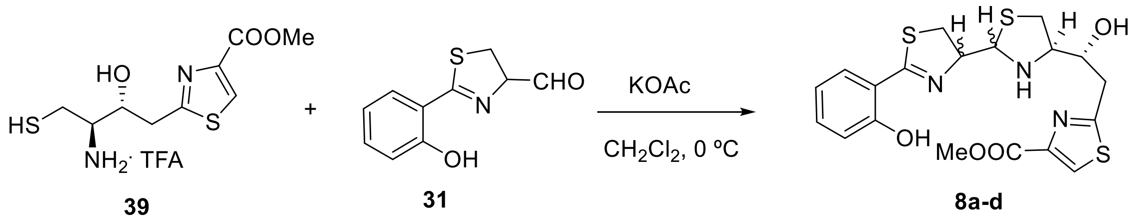

To a suspension of amino thiol **39** (21.5 mg, 0.06 mmol) and potassium acetate (6 mg, 0.06 mmol) in deoxygenated dry CH_2Cl_2 (1.5 mL) at 0°C was added aldehyde **31** (12.4 mg, 0.06 mmol) in deoxygenated dry CH_2Cl_2 (1.5 mL). This suspension was stirred 16 h (overnight) at room temperature in the dark. After that time, the reaction mixture was diluted with CH_2Cl_2 (5 mL) and mili-Q H_2O was added. The layers were separated, and the aqueous layer was extracted with CH_2Cl_2 (2 × 5 mL). The organic layers were collected, dried ($MgSO_4$), filtered and evaporated to dryness under reduced pressure to give a mixture of 29 mg of a residue (18 mg) which was separated by HPLC into two main fractions. HPLC conditions: Semi-Prep HPLC Discovery column HS-F5 (25 cm x 10 mm, 5 µm) with a mobile phase consisting of a 5 min gradient from 30 % to 55% CH_3CN in H_2O (v/v), followed by a 15 min isocratic at 55%, then 5 min gradient from 55% to 100% and 5 min from 100% to 30%, and finally 5 min gradient from 100% to 30%, and 10 min isocratic at 30%, using a flow rate of 4 mL/min (UV detector operating at λ = 214 and 265 nm.). First fraction, with R_t = 19.23 min and second fraction with R_t = 20.41 min, both eluted with 55% of CH_3CN in H_2O, were concentrated under vacuum to give 8.5 mg and 8.3 mg, respectively.

Spectroscopic analysis of both fractions identified **8a** as the major compound along with **8d** as a very minor compound in the first fraction, and a mixture of compounds **8b** and **8c** in a *ca* 4:3 ratio in the second fraction.

First fraction: compound **8a** with **8d** as a very minor compound. (+)-LRMS (ESI) *m/z*: 474.06 $[M+Na]^+$, 452.07 $[M+H]^+$; (+)-HRMS (ESI) *m/z*: $[M+H]^+$ Calcd. for $C_{19}H_{22}N_3O_4S_3$: 452.0766; Found: 452.0774.

(9*R*,10*R*,12*R*,13*R*)-8a ^1H NMR (500 MHz, CD_2Cl_2) δ 8.09 (s, 1H, H-16), 7.42 (dd, *J* = 7.7, 1.4 Hz, 1H, H-5), 7.36 (t, *J* = 7.7 Hz, 1H, H-3), 6.97 (bd, *J* = 7.7 Hz, 1H, H-2), 6.89 (t, *J* = 7.7 Hz, 1H, H-4), 4.96 (ddd, *J* = 8.9, 7.7, 6.5 Hz, 1H, H-9), 4.78 (d, *J* = 6.5 Hz, 1H, H-10), 4.24 (dt, *J* = 9.2, 4.8, 3.0 Hz, 1H, H-13), 3.88 (s, OMe), 3.56 (dd, *J* = 11.2, 8.9 Hz, 1H, H-8), 3.38 (dd, *J* = 11.2, 7.7 Hz, 1H, H-8), 3.32 (dd, *J* = 15.4, 9.2 Hz, 1H, H-14), 3.18 (dd, *J* = 15.4, 3.0 Hz, 1H, H-14), 3.32 (ddd, *J* = 10.0, 6.0, 4.8 Hz, 1H, H-12), 3.05 (dd, *J* = 10.0, 6.0 Hz, 1H, H-11), 2.95 (t, *J* = 10.0 Hz, 1H, H-11); ^{13}C NMR (126 MHz, CD_2Cl_2) δ 173.45 (C, C-7), 168.68 (C, C-15), 161.90 (CO_2Me), 159.50 (C, C-1), 146.84 (C, C-17), 133.69 (CH, C-3), 131.08 (CH, C-5), 127.90 (CH, C-16), 117.40 (CH, C-4), 117.40 (CH, C-2), 116.52 (C, C-6), 80.56 (CH, C-9), 72.12 (CH, C-10), 70.65 (CH, C-13), 68.83 (CH, C-12), 52.53 (CH_3, OMe), 38.65 (CH_2, C-14), 34.89 (CH_2, C-8), 34.12 (CH_2, C-11).

(9*S*,10*S*,12*R*,13*S*)-8d ^1H NMR (500 MHz, CD_2Cl_2) δ 8.02 (s, 1H, H-16), 7.42 (dd, *J* = 7.7, 1.4 Hz, 1H, H-5), 7.37 (t, *J* = 7.7 Hz, 1H, H-3), 6.96 (bd, *J* = 7.7 Hz, 1H, H-2), 6.88 (t, *J* = 7.7 Hz, 1H, H-4), 4.84 (m, 1H, H-9), 4.83 (d, *J* = 6.5 Hz, 1H, H-10), 4.33 (ddd, *J* = 8.9, 6.3, 2.9 Hz, 1H, H-13), 3.88 (s, OMe), 3.46 (dd, *J* = 10.9, 7.9 Hz, 1H, H-8), 3.31 (m, 1H, H-8), 3.44 (m, 1H, H-14), 3.17 (m, 1H, H-14), 3.47 (dd, *J* = 10.9, 5.9 Hz, 1H, H-12), 3.08 (dd, *J* = 9.8, 5.9 Hz, 1H, H-11), 2.98 (t, *J* = 9.8 Hz, 1H, H-11); ^{13}C NMR (126 MHz, CD_2Cl_2) δ 173.07 (C, C-7), 168.84 (C, C-15), 161.87 (CO_2Me), 159.61 (C, C-1), 146.76 (C, C-17), 133.70 (CH, C-3), 130.89 (CH, C-5), 127.90 (CH, C-16), 119.18 (CH, C-4), 117.42 (CH, C-2), 116.62 (C, C-6), 81.26 (CH, C-9), 71.88 (CH, C-10), 70.37 (CH, C-13), 69.63 (CH, C-12), 52.52 (CH_3, OMe), 38.57 (CH_2, C-14), 35.49 (CH_2, C-8), 34.55 (CH_2, C-11). Second fraction: compounds **8b** and **8c**. (+) HRMS (ESI) *m/z*: $[M+H]^+$. Calcd. for $C_{19}H_{22}N_3O_4S_3$: 452.0766; Found: 452.0767.

(9*S*,10*R*,12*R*,13*R*)-8b ^1H NMR (500 MHz, CD_2Cl_2) δ 8.08 (s, 1H, H-16), 7.42 (dd, *J* = 7.7, 1.4 Hz, 1H, H-5), 7.37 (t, *J* = 7.7 Hz, 1H, H-3), 6.96 (bd, *J* = 7.7 Hz, 1H, H-2), 6.88 (t, *J* = 7.7 Hz, 1H, H-4), 4.99 (m, 1H, H-9), 4.93 (d, *J* = 5.7 Hz, 1H, H-10), 4.38 (ddd, *J* = 9.2, 5.0, 3.1 Hz, 1H, H-13), 3.88 (s, OMe), 3.49 (m, 1H, H-8), 3.37 (m, 1H, H-8), 3.34 (m, 1H, H-14), 3.18 (m, 1H, H-14), 3.34 (ddd, *J* = 9.8, 5.5, 3.1 Hz, 1H, H-12), 3.08 (dd, *J* = 9.8, 5.5 Hz, 1H, H-11), 2.98 (t, *J* = 9.8 Hz, 1H, H-11); ^{13}C NMR (126 MHz, CD_2Cl_2) δ 173.59 (C, C-7), 168.66 (C, C-15), 161.89 (CO_2Me), 159.56 (C, C-1), 146.81 (C, C-17), 133.70 (CH, C-3), 130.99 (CH, C-5), 127.90 (CH, C-16), 119.29 (CH, C-4), 117.42 (CH, C-2), 116.52 (C, C-6), 82.11 (CH, C-9), 71.79 (CH, C-10), 70.79 (CH, C-13), 69.02 (CH, C-12), 52.51 (CH_3, OMe), 38.69 (CH_2, C-14), 34.28 (CH_2, C-8), 34.28 (CH_2, C-11).

(9*S*,10*R*,12*R*,13*R*)-8c ^1H NMR (500 MHz, CD_2Cl_2) δ 8.09 (s, 1H, H-16), 7.42 (dd, *J* = 7.7, 1.4 Hz, 1H, H-5), 7.37 (t, *J* = 7.7 Hz, 1H, H-3), 6.96 (bd, *J* = 7.7 Hz, 1H, H-2), 6.88 (t, *J* = 7.7 Hz, 1H, H-4), 4.83 (m, 1H, H-9), 4.67 (d, *J* = 8.2 Hz, 1H, H-10), 4.33 (ddd, *J* = 8.8, 5.7, 2.9 Hz, 1H, H-13), 3.89 (s, OMe), 3.53 (m, 1H, H-8), 3.39 (m, 1H, H-8), 3.43 (m, 1H, H-14), 3.19 (m, 1H, H-14), 3.43 (dd, *J* = 10.9, 5.9 Hz, 1H, H-12), 3.08 (dd, *J* = 10.2, 5.5 Hz, 1H, H-11), 2.98 (dd, *J* = 10.2, 8.4 Hz, 1H, H-11); ^{13}C NMR (126 MHz, CD_2Cl_2) δ 173.48 (C, C-7), 168.80 (C, C-15), 161.87 (CO_2Me), 159.59 (C, C-1), 146.87 (C, C-17), 133.60 (CH, C-3), 130.92 (CH, C-5), 127.82 (CH, C-16), 119.25 (CH, C-4), 117.38 (CH, C-2), 116.65 (C, C-6), 79.97 (CH, C-9), 71.79 (CH, C-10), 70.37 (CH, C-13), 69.63 (CH, C-12), 52.52 (CH_3, OMe), 38.80 (CH_2, C-14), 35.49 (CH_2, C-8), 34.53 (CH_2, C-11).

*Synthesis of compounds **9a** and **9d**.*

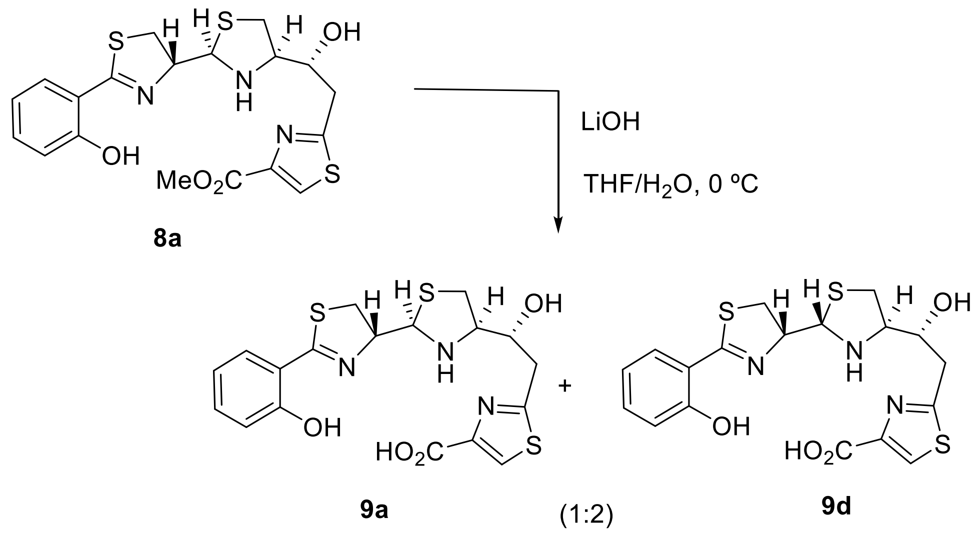

To a solution of **8a** (4.2 mg, 0.01 mmol) in THF:Mili-Q H_2O (0.4 mL: 0.1 mL) was added LiOH (1.2 mg, 0.05 mmol) at 0°C. After stirring for 1h, the reaction mixture was quenched by the addition of NH_4Cl in Mili-Q H_2O (11 mg, 0.2 mL) and the solvents were concentrated under vacuum to give a mixture of **9a** and **9d** in a *ca* 1:2 ratio. (+)-HRMS (ESI) *m/z*: $[M+H]^+$ Calcd. for $C_{18}H_{20}N_3O_4S_3$: 438.0610; Found: 438.0610. (-)-HRMS (ESI) *m/z*: $[M-H]^-$ Calcd. for $C_{18}H_{18}N_3O_4S_3$: 436.0465; Found: 436.0457.

(9*R*,10*R*,12*R*,13*R*)-9a ^1H NMR (500 MHz, CD_3OD) δ 7.90 (s, 1H, H-16), 7.42 (m, 1H, H-5), 7.37 (m, 1H, H-3), 6.96 (m, 1H, H-2), 6.88 (m, 1H, H-4), 4.93 (u.s., 1H, H-9), 4.66 (d, *J* = 7.7 Hz, 1H, H-10), 4.26 (dt, *J* = 9.0, 5.6, 3.6 Hz, 1H, H-13), 3.65 (dd, *J* = 11.3, 8.7 Hz, 1H, H-8), 3.39 (m, 1H, H-8), 3.40 (m, 1H, H-14), 3.17 (m, 1H, H-14), 3.26 (m, 1H, H-12), 3.09 (m, 1H, H-11), 2.91 (t, *J* = 9.8 Hz, 1H, H-11); ^{13}C NMR (126 MHz, CD_3OD) δ 174.02 (C, C-7), 169.24 (CO_2H, C-18), 168.61 (C, C-15), 160.13 (C, C-1), 154.66 (C, C-17), 134.37 (CH, C-3), 131.73 (CH, C-5), 124.50 (CH, C-16), 120.13 (CH, C-4), 117.90 (CH, C-2), 117.35 (C, C-6), 82.11 (CH, C-9), 73.48 (CH, C-10), 72.12 (CH, C-13), 70.44 (CH, C-12), 40.12 (CH_2, C-14), 36.36 (CH_2, C-11), 35.31 (CH_2, C-8).

(9*R*,10*S*,12*R*,13*R*)-9d ^1H NMR (500 MHz, CD_3OD) δ 7.82 (s, 1H, H-16), 7.42 (m, 1H, H-5), 7.37 (m, 1H, H-3), 6.96 (m, 1H, H-2), 6.88 (m, 1H, H-4), 4.95 (u.s., 1H, H-10), 4.87 (u.s., 1H, H-9), 4.10 (ddd, *J* = 8.5, 6.9, 3.4 Hz 1H, H-13), 3.50 (dd, *J* = 11.0, 8.6 Hz, 1H, H-8), 3.36 (dd, *J* = 11.0, 9.2 Hz, 1H, H-8), 3.44 (m, 1H, H-14), 3.14 (m, 1H, H-14), 3.43 (m, 1H, H-12), 3.11 (dd, *J* = 10.2, 5.9 Hz, 1H, H-11), 2.98 (dd, *J* = 10.2, 7.3 Hz, 1H, H-11); ^{13}C NMR (126 MHz, CD_3OD) δ 173.39 (C, C-7), 169.24 (CO_2H, C-18), 168.73 (C, C-15), 160.21 (C, C-1), 154.53 (C, C-17), 134.25 (CH, C-3), 131.52 (CH, C-5), 124.43 (CH, C-16), 120.02 (CH, C-4), 117.93 (CH, C-2), 117.48 (C, C-6), 82.62 (CH, C-9), 73.03 (CH, C-10), 71.81 (CH, C-13), 69.33 (CH, C-12), 40.05 (CH_2, C-14), 36.70 (CH_2, C-11), 34.61 (CH_2, C-8).

*Synthesis of **9b** and **9c**.*

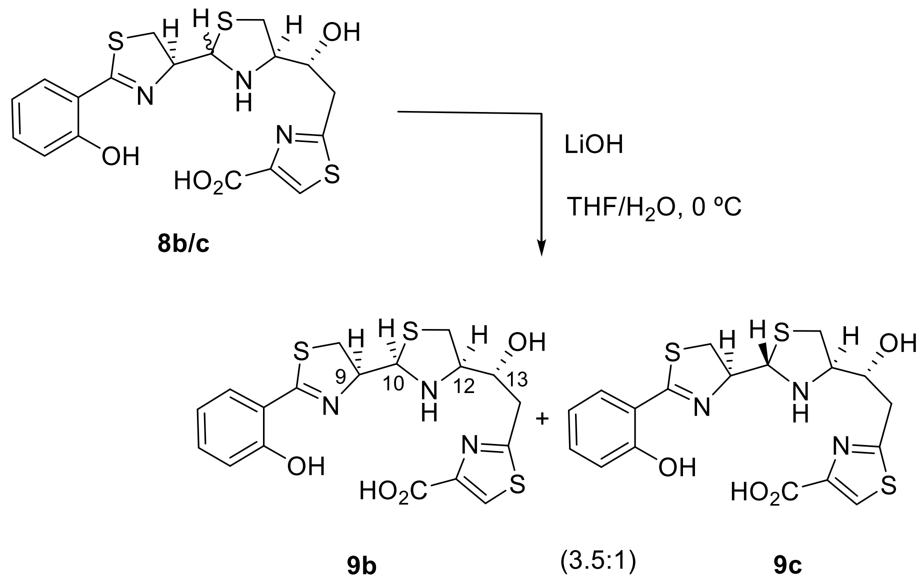

To a solution of **8b,c** (4.4 mg, 0.01 mmol) in THF:Mili-Q H_2O (0.4 mL: 0.1 mL) was added LiOH (1.2 mg, 0.05 mmol) at 0°C. After stirring for 1h, the reaction mixture was quenched by the addition of NH_4Cl in mili-Q H_2O (6.5 mg, 0.2 mL) and the solvents were concentrated under vacuum to give a mixture of **9b/9c** as a mixture in a *ca*. 3.5:1 ratio. (-)-HRMS (ESI) *m/z*: $[M-H]^-$ Calcd. for $C_{18}H_{18}N_3O_4S_3$: 436.0465; Found: 436.0468.

(9*S*,10*R*,12*R*,13*R*)-9b ^1H NMR (500 MHz, CD_3OD) δ 7.89 (s, 1H, H-16), 7.42 (dd, *J* = 8.0, 1.4 Hz, 1H, H-5), 7.37 (bt, *J* = 8.0 Hz, 1H, H-3), 6.93 (dd, *J* = 8.0, 1.0 Hz, 1H, H-2), 6.89 (bt, *J* = 8.0 Hz, 1H, H-4), 5.04 (u.s., 1H, H-9), 4.95 (u.s., 1H, H-10), 4.26 (dt, *J* = 9.0, 5.6, 3.6 Hz, 1H, H-13), 3.55 (dd, *J* = 10.9, 8.8 Hz, 1H, H-8), 3.39 (dd, *J* = 10.9, 9.2 Hz, 1H, H-8), 3.39 (dd, *J* = 15.1, 3.6 Hz, 1H, H-14), 3.17 (dd, *J* = 15.1, 9.2 Hz, 1H, H-14), 3.25 (dt, *J* = 9.8, 5.6, 5.6 Hz, 1H, H-12), 3.11 (dd, *J* = 9.8, 5.6 Hz, 1H, H-11), 2.90 (t, *J* = 9.8 Hz, 1H, H-11); ^{13}C NMR (126 MHz, CD_3OD) δ 174.10 (C, C-7), 169.24 (CO_2H, C-18), 168.54 (C, C-15), 160.18 (C, C-1), 154.62 (C, C-17), 134.37 (CH, C-3), 131.64 (CH, C-5), 124.53 (CH, C-16), 120.09 (CH, C-4), 117.93 (CH, C-2), 117.36 (C, C-6), 80.89 (CH, C-9), 73.00 (CH, C-10), 71.99 (CH, C-13), 70.50 (CH, C-12), 40.08 (CH_2, C-14), 35.33 (CH_2, C-11), 34.36 (CH_2, C-8).

(9*S*,10*S*,12*R*,13*R*)-9c ^1H NMR (500 MHz, CD_3OD) δ 7.89 (s, 1H, H-16), 7.41 (m, 1H, H-5), 7.37 (m, 1H, H-3), 6.93 (m, 1H, H-2), 6.91 (m, 1H, H-4), 4.79 (m, 1H, H-9), 4.64 (d, *J* = 9.1 Hz, 1H, H-10), 4.12 (m, 1H, H-13), 3.56 (dd, *J* = 11.2, 6.4 Hz, 1H, H-8), 3.40 (m, 1H, H-8), 3.46 (m, 1H, H-14), 3.20 (m, 1H, H-14), 3.42 (m, 1H, H-12), 3.11 (m, 1H, H-11), 2.97 (dd, *J* = 10.2, 7.2 Hz, 1H, H-11); ^{13}C NMR (126 MHz, CD_3OD) δ 177.92 (C, C-7), 169.31 (CO_2H, C-18), 168.74 (C, C-15), 160.22 (C, C-1), 154.66 (C, C-17), 134.25 (CH, C-3), 131.57 (CH, C-5), 124.31 (CH, C-16), 120.03 (CH, C-4), 117.89 (CH, C-2), 117.48 (C, C-6), 83.63 (CH, C-9), 73.03 (CH, C-10), 71.84 (CH, C-13), 68.10 (CH, C-12), 40.39 (CH_2, C-14), 35.96 (CH_2, C-11), 34.60 (CH_2, C-8).

1.5 Attempts of Gallium (III) complexation of 9a-d

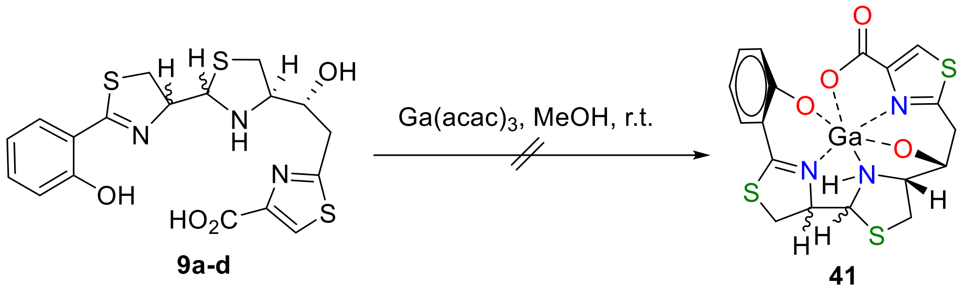

9a-d **41**

Gallium acetylacetonate (5 mg, 0.013 mmol) was added to a solution of crude compound **9a-d** (4 mg) in CD_3OD (0.7 mL), placed in a NMR tube. The reaction was monitored via ^{1}H-NMR spectroscopy. The solvent was concentrated under vacuum after 24 h and submitted to ESI and analytical HPLC. The residue was passed through a Hypersep C18 (250 mg) cartridge which was eluted with following mixtures of mili-Q H_2O and CH_3CN 10:0, 7:3, 1:1, 3:7 and 0:1. The fractions were submitted to ESI and ^{1}H-NMR, showing no evidence of gallium chelation.

Table S1. Diagnostic NMR chemical shifts and coupling constants at C-9 and C-10 positions in 6–9.

	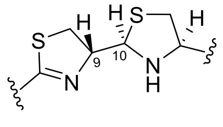		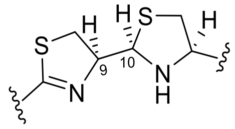		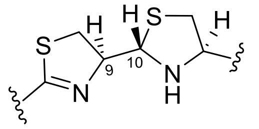		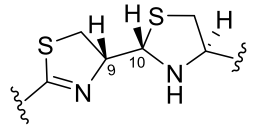	
	9*R*/10*R*		9*S*/10*R*		9*S*/10*S*		9*R*/10*S*	
**^{1}H NMR**	H9	H10	H9	H10	H9	H10	H9	H10
**6** (13*S*)	**6a**		**6b**		**6c**		**6d**	
	4.96	4.75 d (6.8)	4.99	4.91 d (5.5)	4.83	4.69 d (8.2)	-	-
**7** (13*R*)	**7a**		**7b**		**7c**		**7d**	
	4.98	4.67 d (7.5)	5.11	4.99	4.79	4.67 d (9.1)	4.85	4.99
**8** (13*S*)	**8a**		**8b**		**8c**		**8d**	
	4.96	4.78 d (6.5)	4.99	4.93 d (5.7)	4.83	4.67 d (8.2)	4.84	4.83 d (6.5)
**9** (13*R*)	**9a**		**9b**		**9c**		**9d**	
	4.93	4.66	5.04	4.95	4.79	4.64 d (9.1)	4.87	4.95
**^{13}C NMR**	C9	C10	C9	C10	C9	C10	C9	C10
**6** (13*S*)	**6a**		**6b**		**6c**		**6d**	
	80.72	72.66	81.72	72.39	79.93	71.78	-	-
**7** (13*R*)	**7a**		**7b**		**7c**		**7d**	
	81.83	73.82	80.67	73.16	83.78	72.99	82.97	72.76
**8** (13*S*)	**8a**		**8b**		**8c**		**8d**	
	80.56	72.12	82.11	71.79	79.97	71.79	81.26	71.88
**9** (13*R*)	**9a**		**9b**		**9c**		**9d**	
	82.11	73.48	80.89	73.00	83.63	73.03	82.62	73.03

2. NMR spectra and MS of intermediates of the synthesis of epimeric β-hydroxy carboxylic acids 14 and 17 (Scheme 2)

2.1 ^{1}H-NMR (300.13 MHz, DMSO-d_6) of S-trityl-L-cysteine

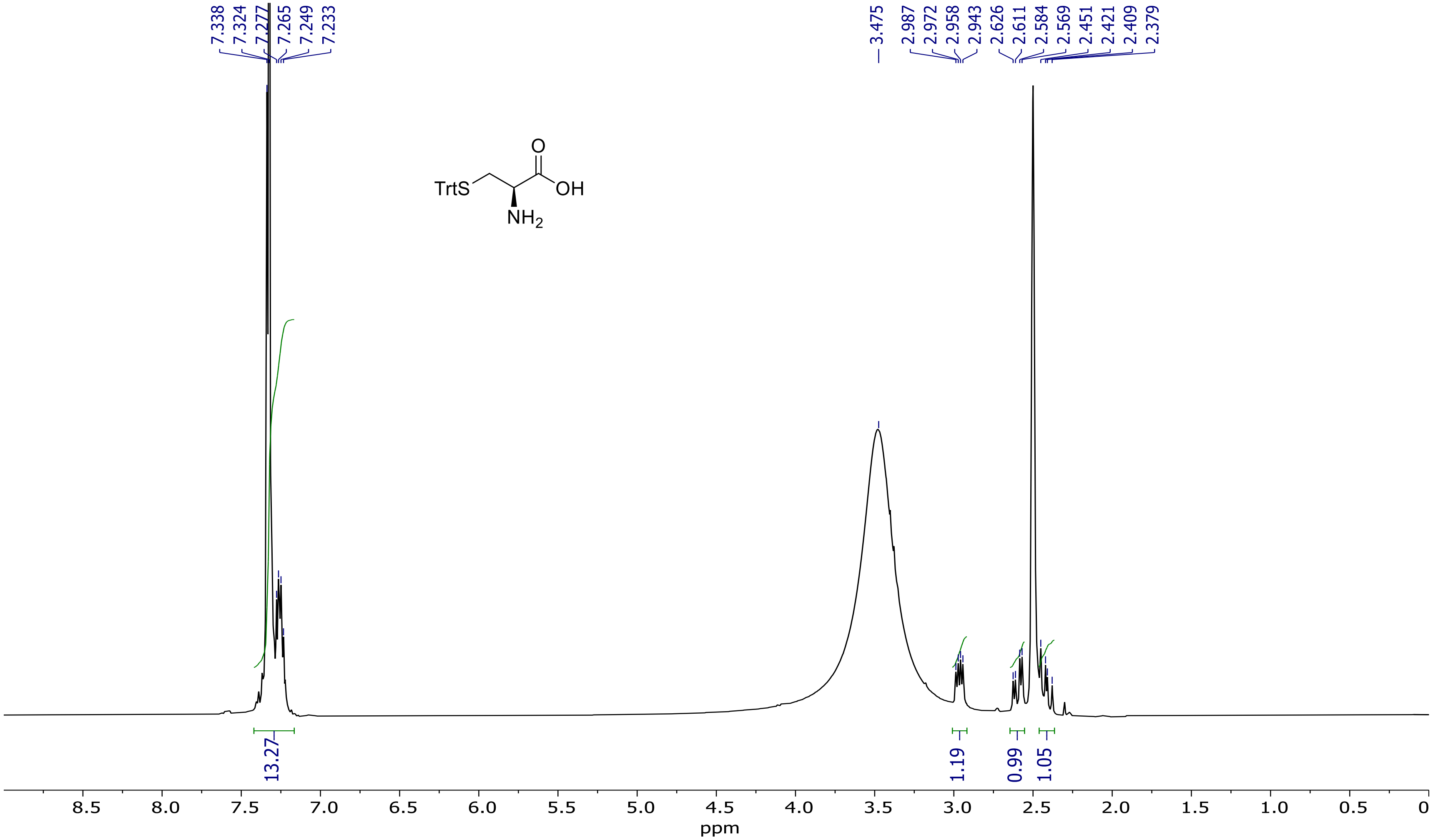

2.2 ^{1}H NMR (300.13 MHz, $CDCl_3$) of Boc-Cys(Trt)-OH (10)

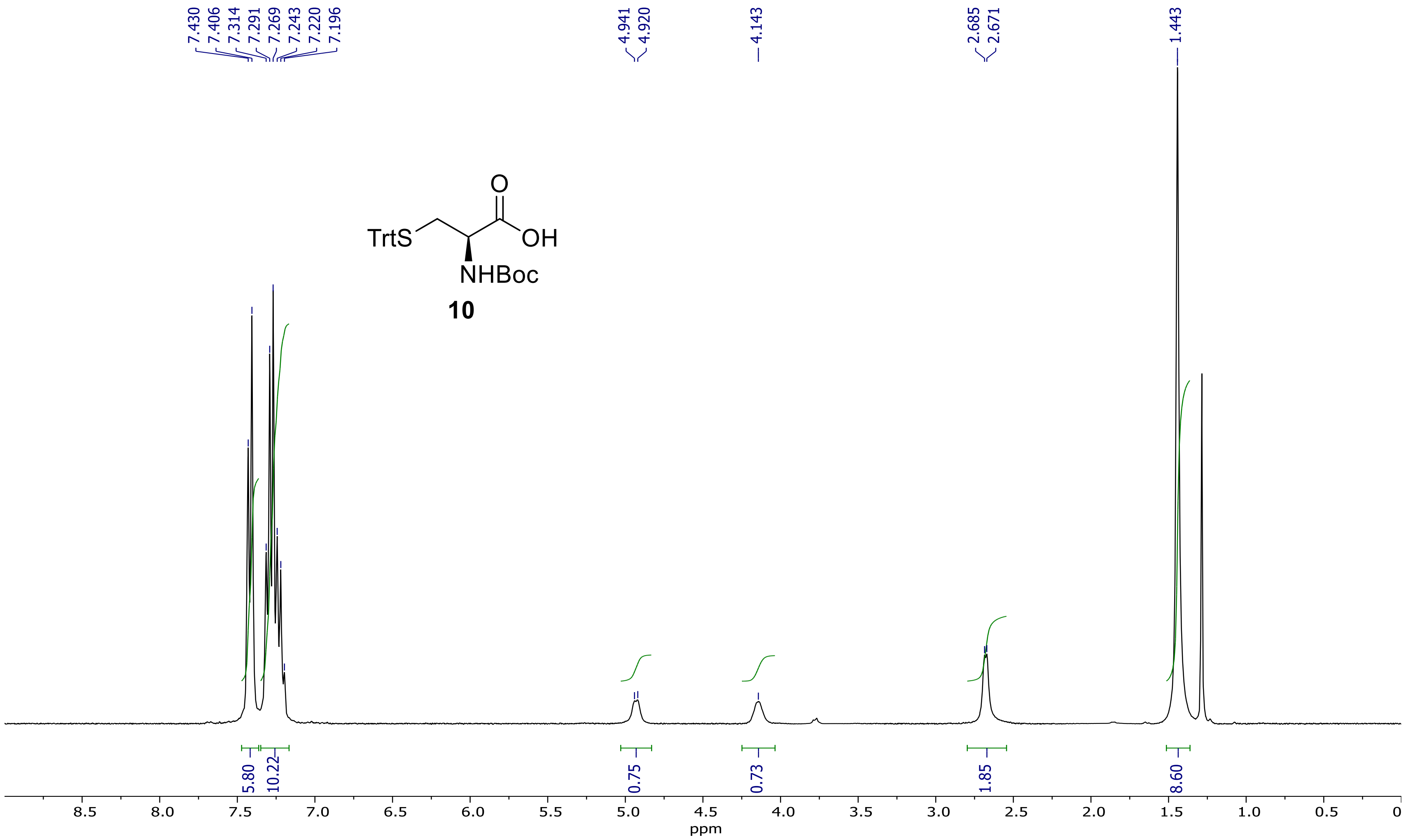

2.3 ^{1}H NMR (300.13 MHz, $CDCl_3$) of 11

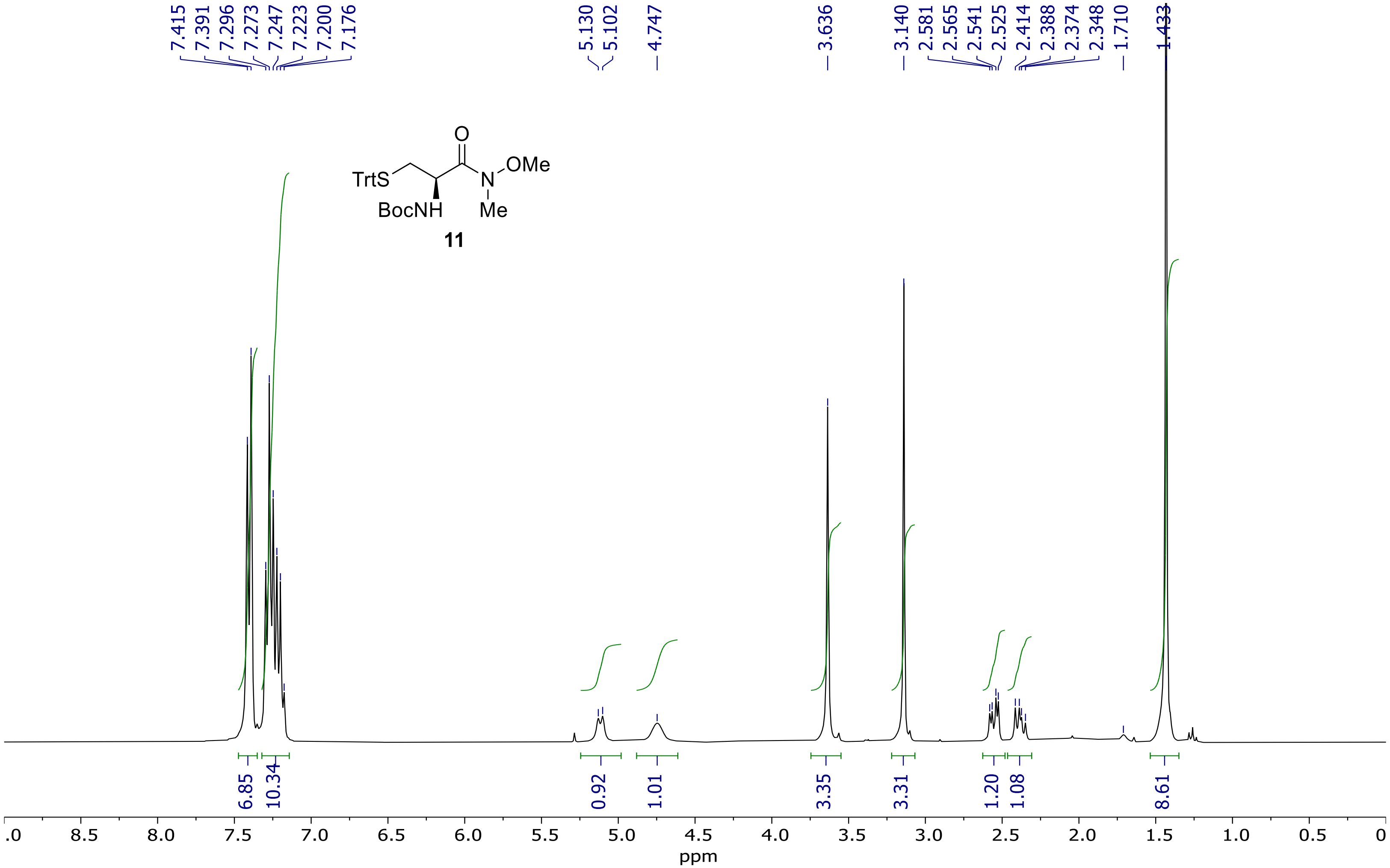

2.4 ^{1}H NMR (300.13 MHz, $CDCl_3$) of 12

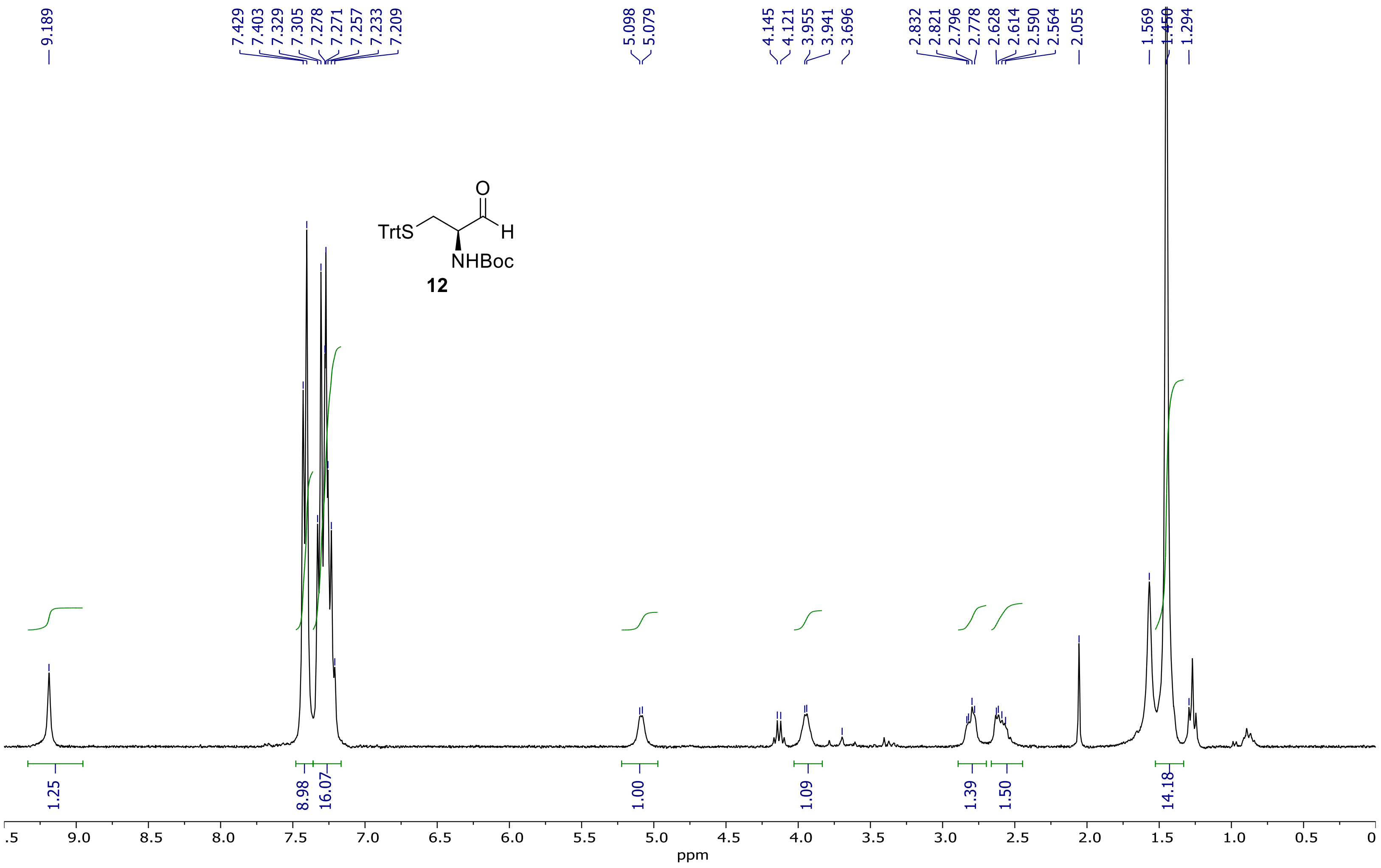

2.5. NMR and MS of 13

^{1}H NMR (400.13 MHz, $CDCl_3$) of **13**

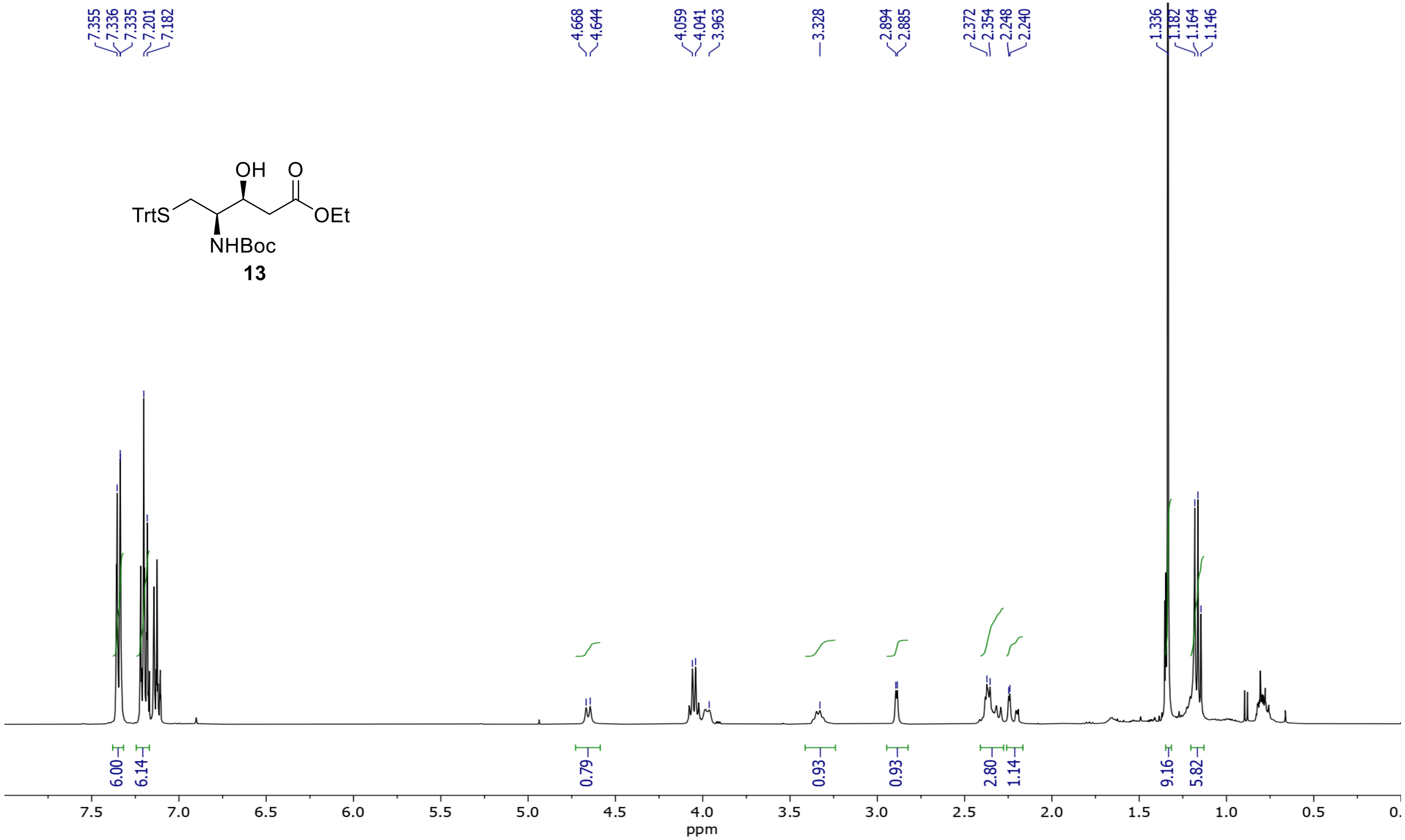

^{13}C NMR (100.13 MHz, $CDCl_3$) of **13**

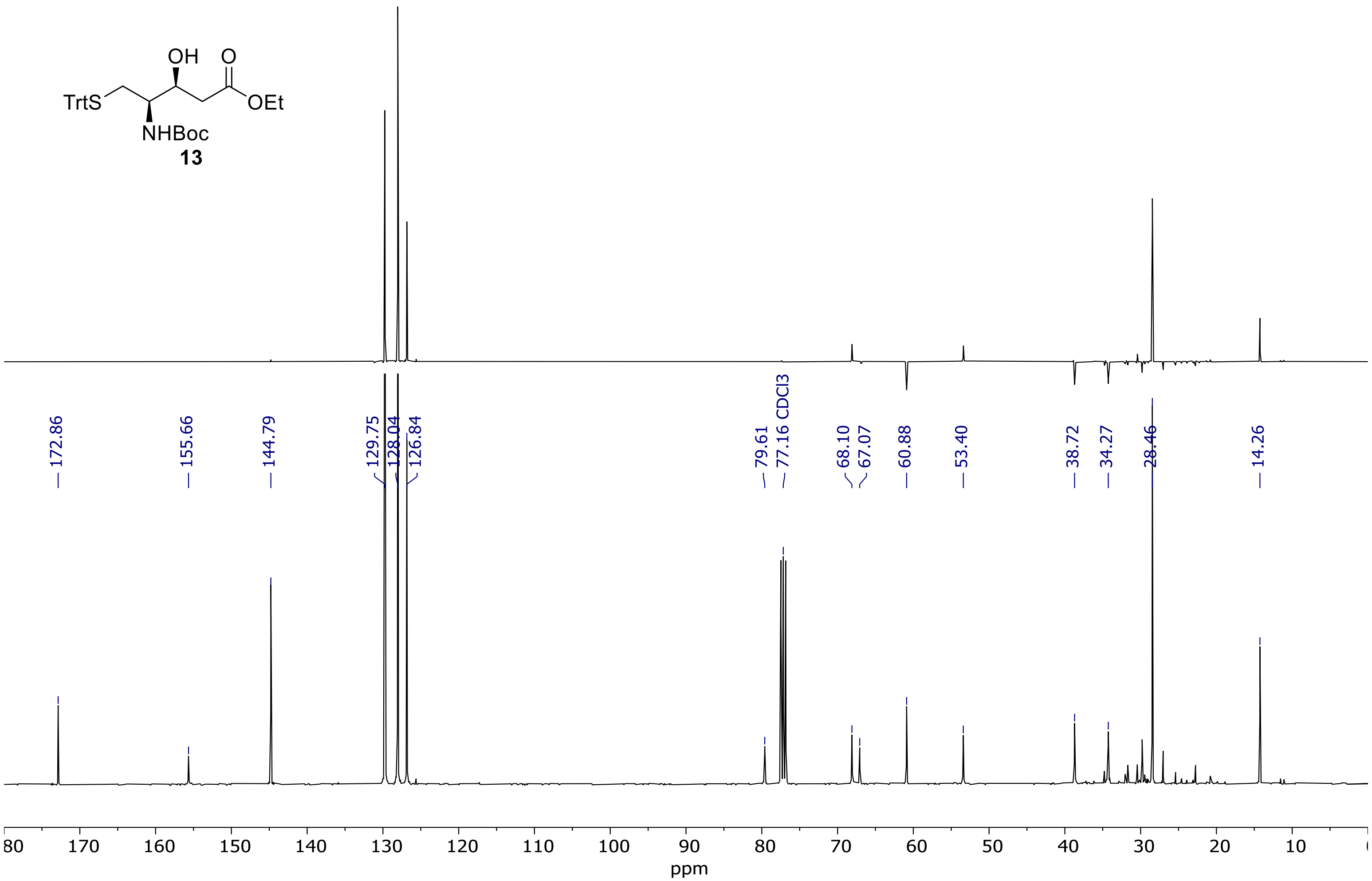

^{1}H-^{1}H COSY (400.13 MHz, $CDCl_3$) of **13**

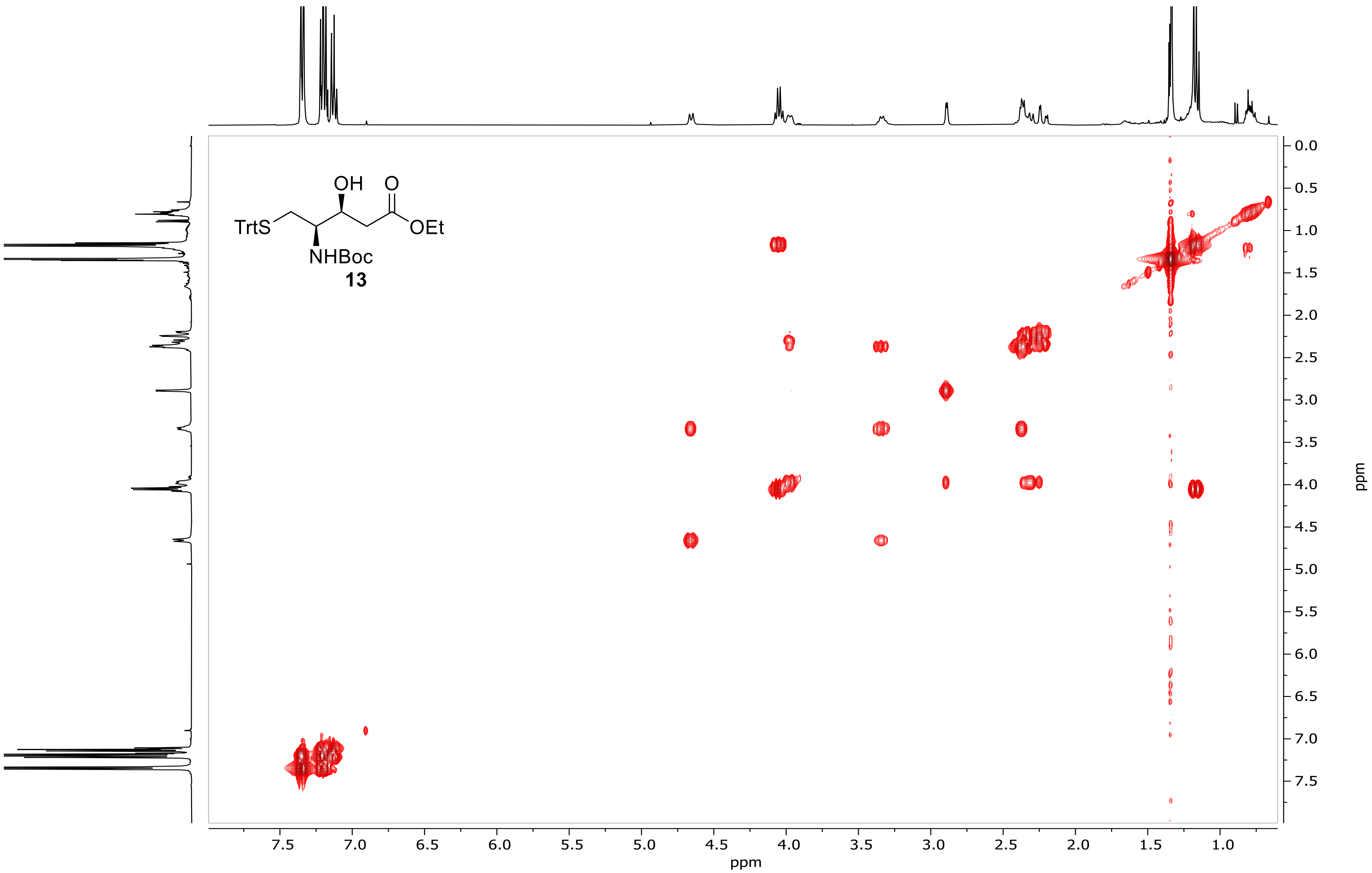

HSQC (400.13 MHz, $CDCl_3$) of **13**

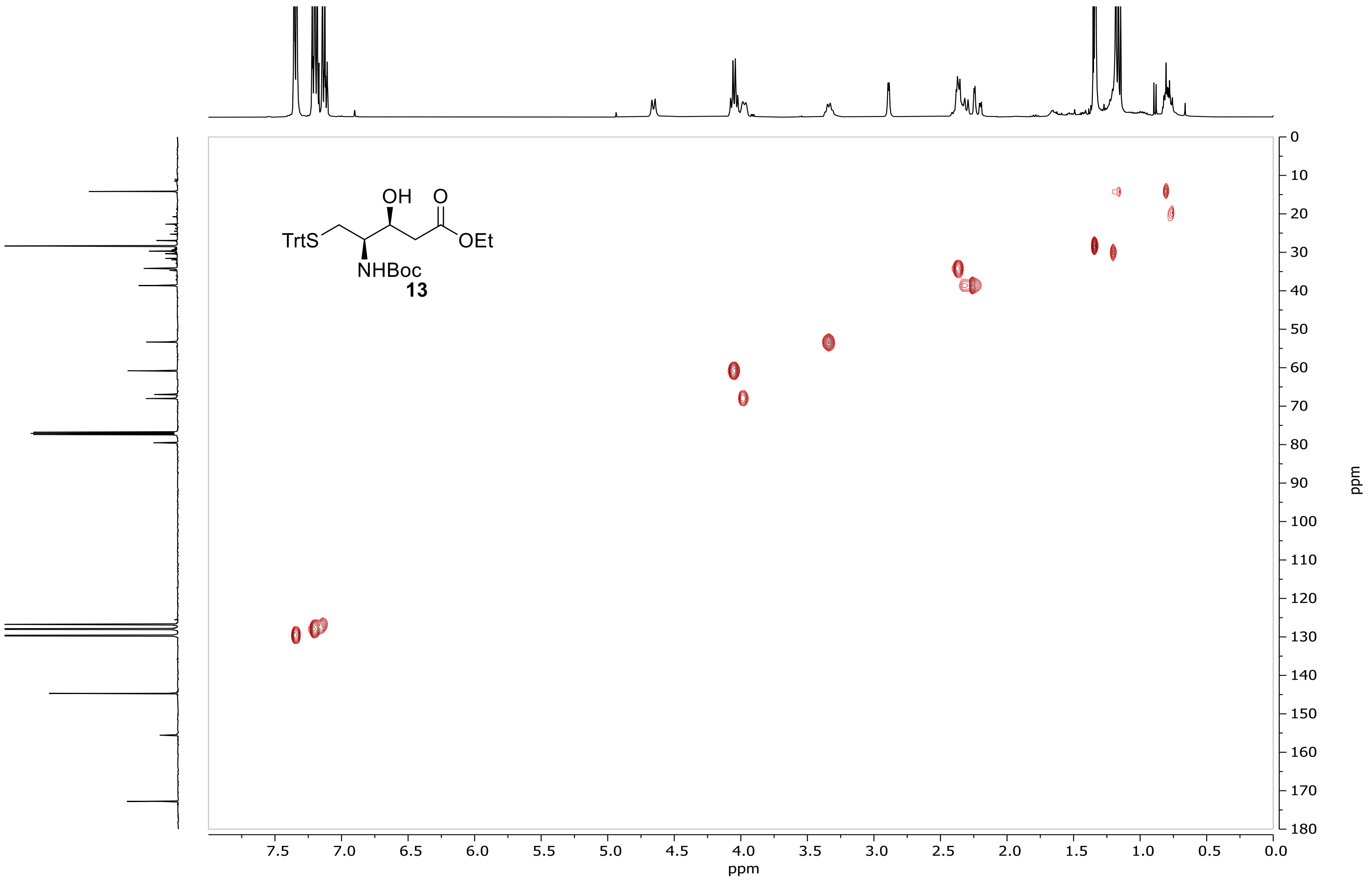

(+)-HRESIMS of **13**: Ion: *m/z*: 558.2303 [M+Na]$^+$

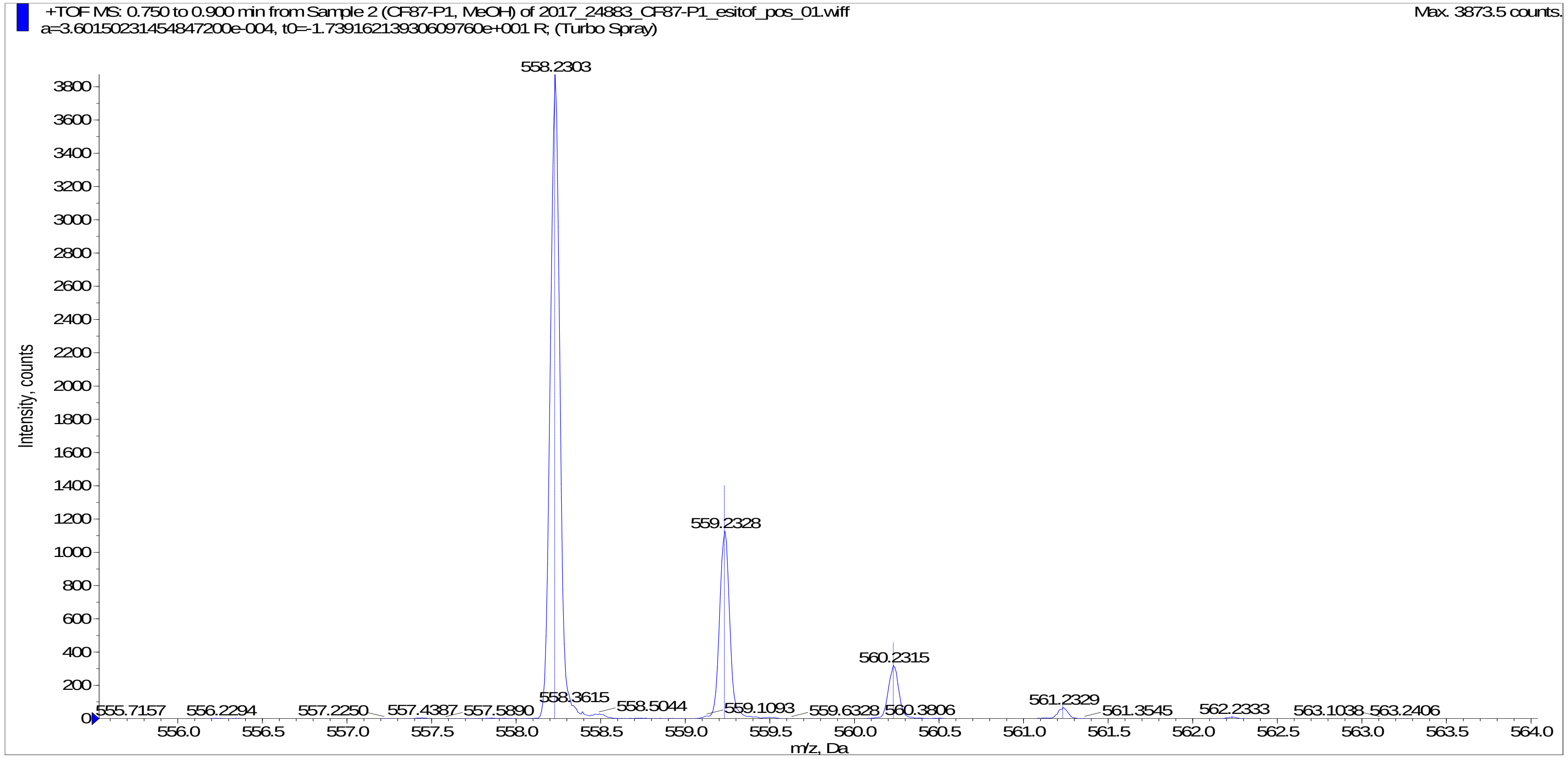

Formula	Calc m/z	Δ, mDa	Δ, ppm	DBE
$C_{31}H_{37}NO_5NaS$	558.2284	1.8333	3.2842	13.5

2.6 NMR and MS of 14

^{1}H NMR (300.13 MHz, $CDCl_3$) of **14**

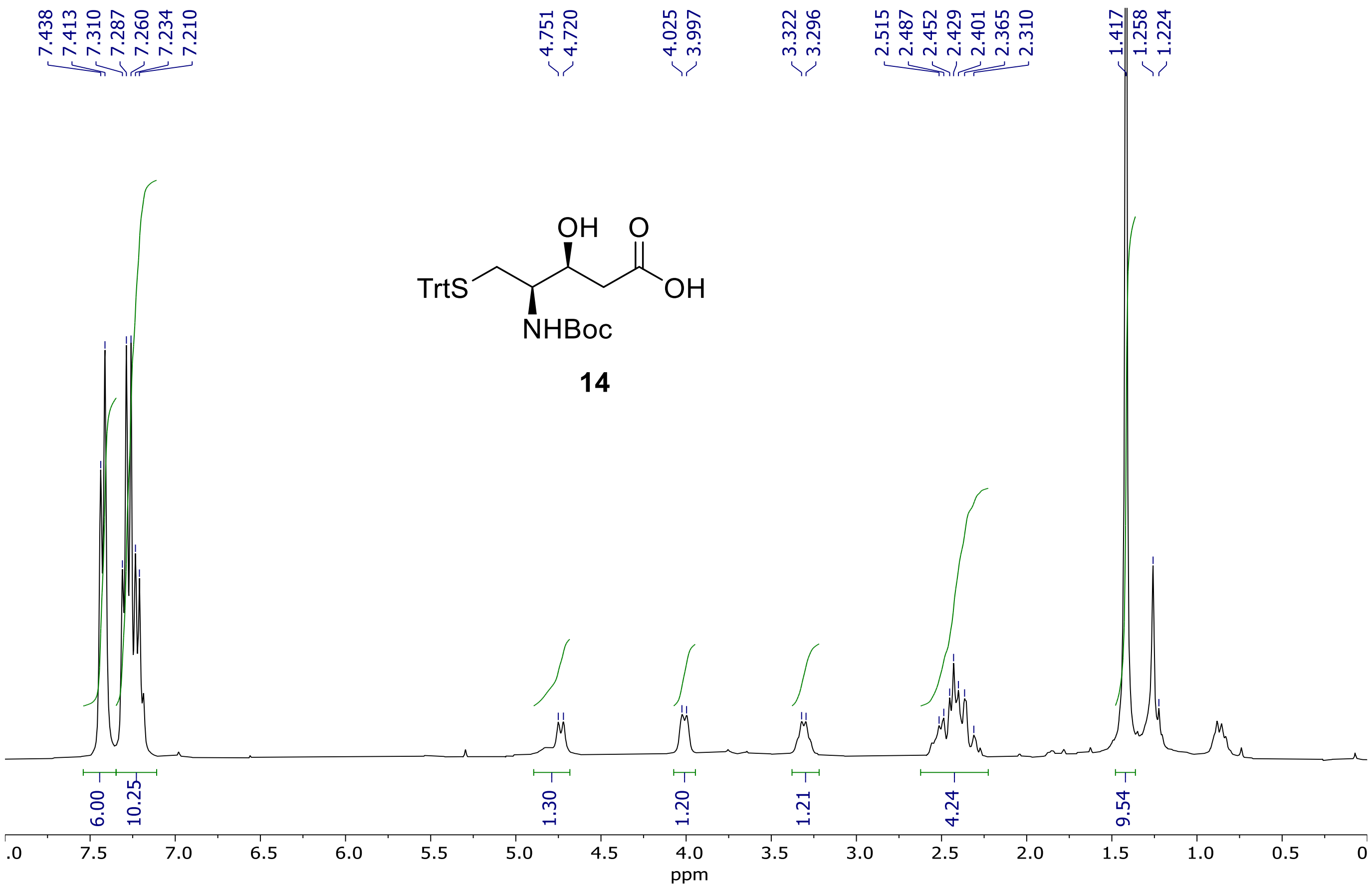

^{13}C NMR (75 MHz, $CDCl_3$) of **14**

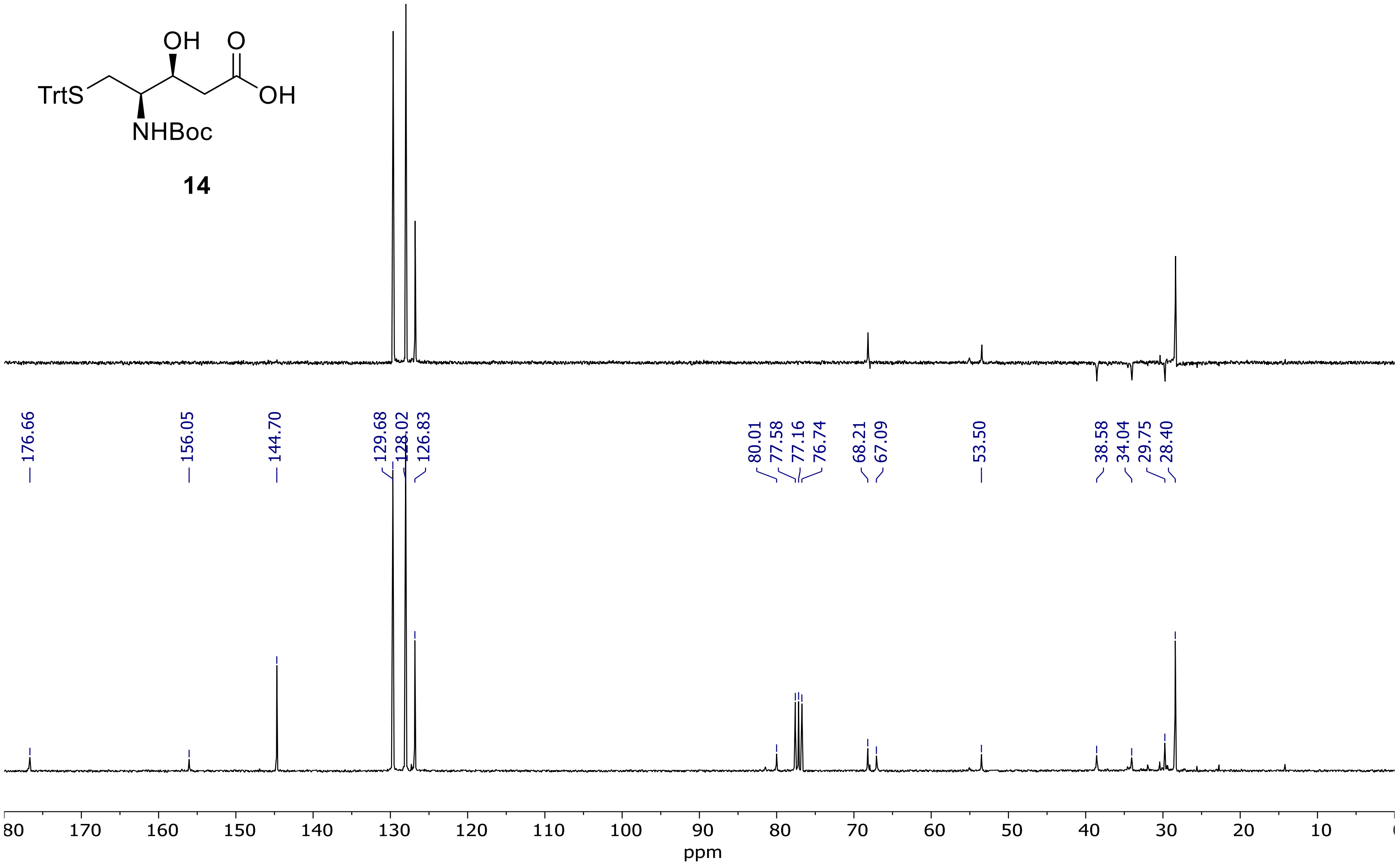

^{1}H-^{1}H COSY (75 MHz, $CDCl_3$) of **14**

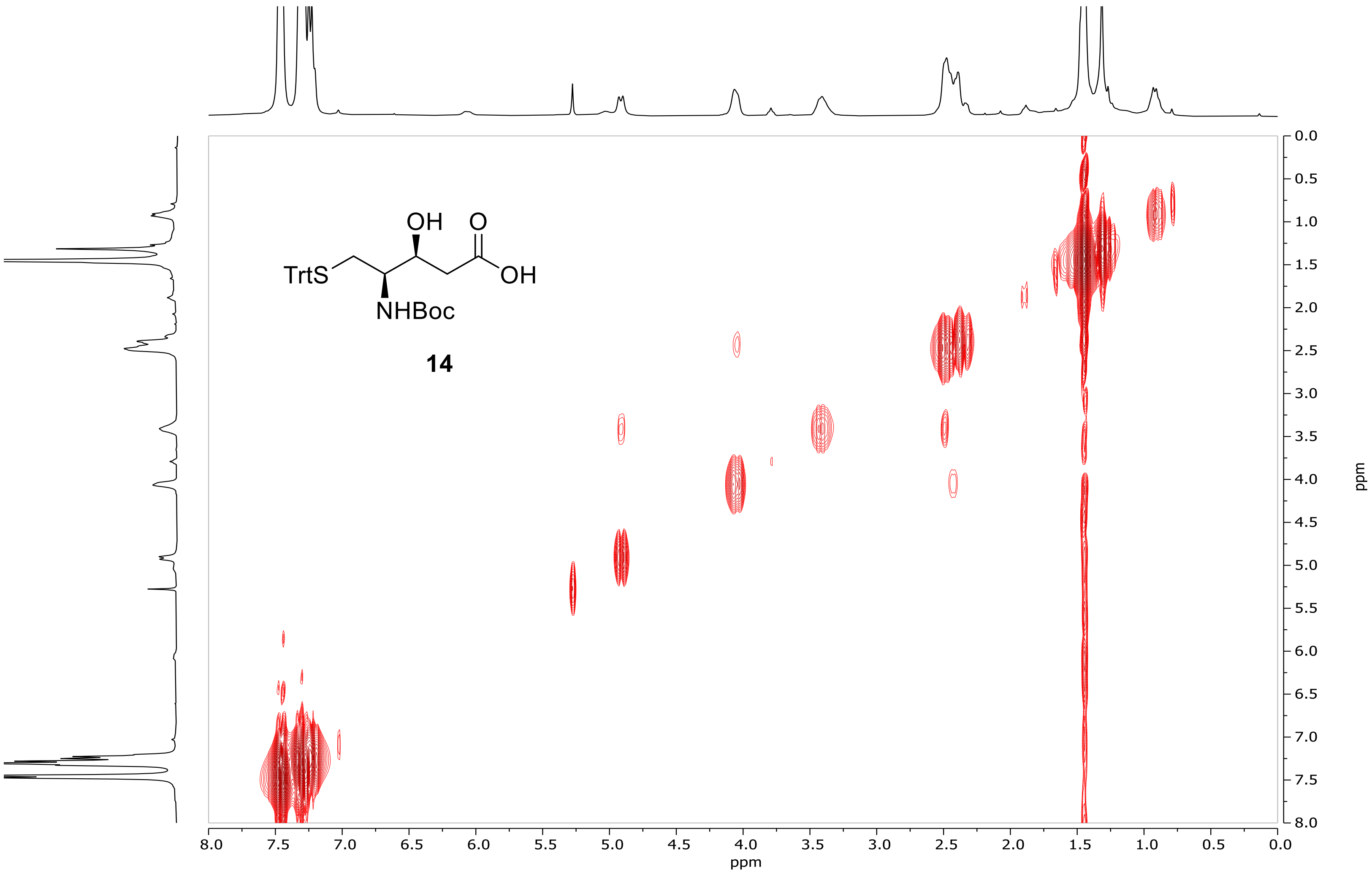

HSQC (75 MHz, $CDCl_3$) of **14**

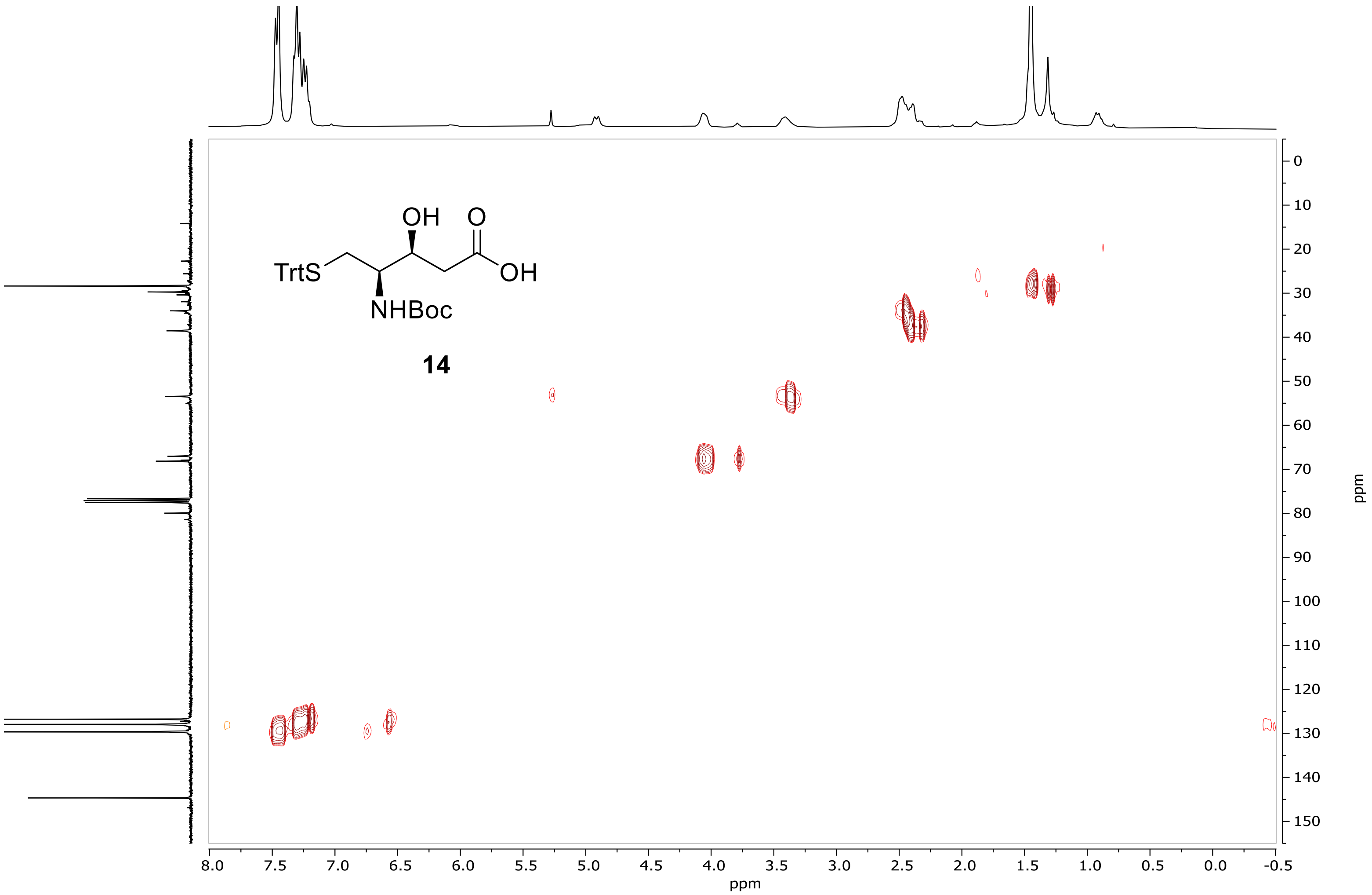

(+)-HRESIMS of **14**: Ion: *m/z*: 530.1980 ($[M+Na]^+$)

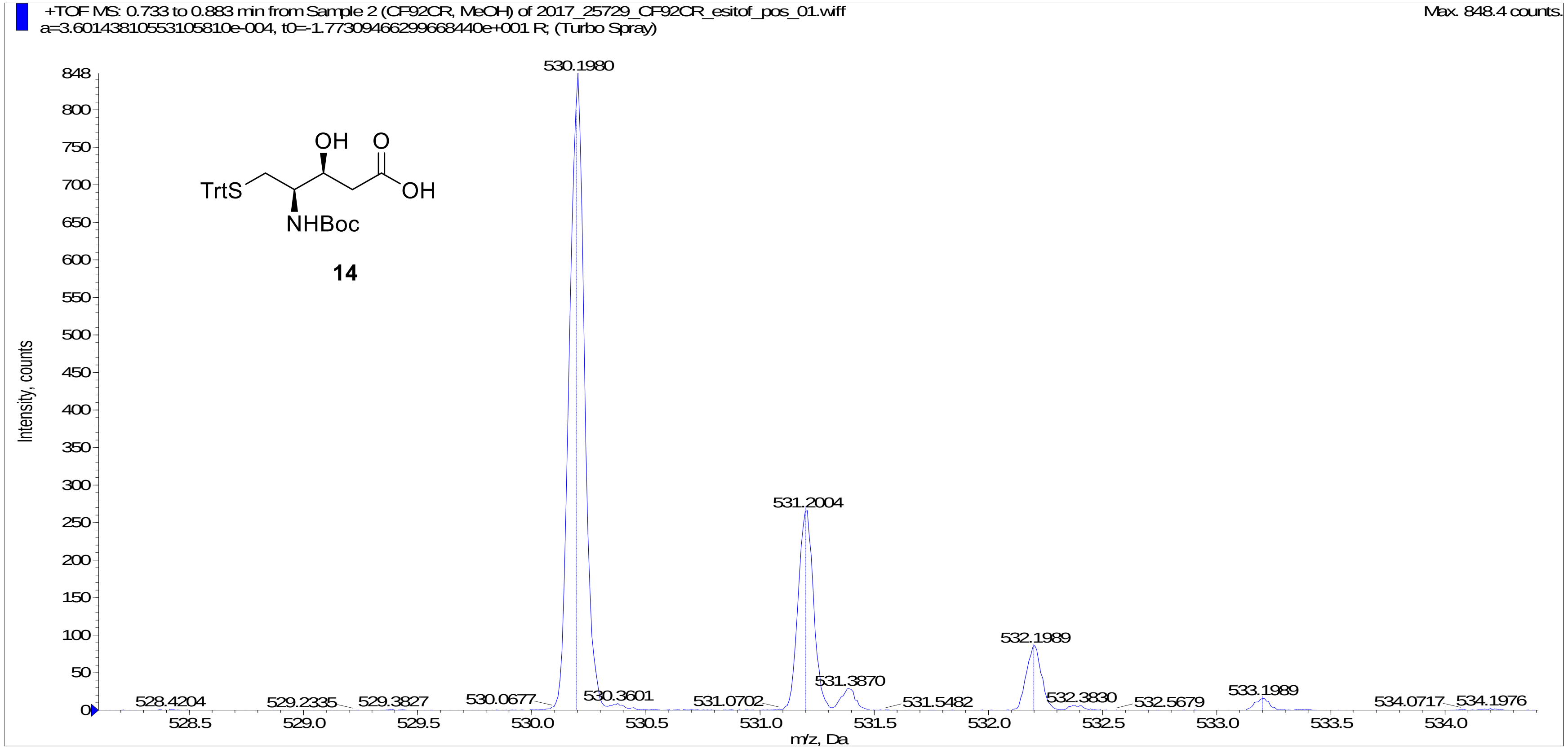

Formula	Calc m/z	Δ, mDa	Δ, ppm	DBE
$C_{29}H_{33}NO_5NaS$	530.1971	0.8335	1.5721	13.5

2.7 NMR and MS of 15

^{1}H NMR (400 MHz, $CDCl_3$) of **15**

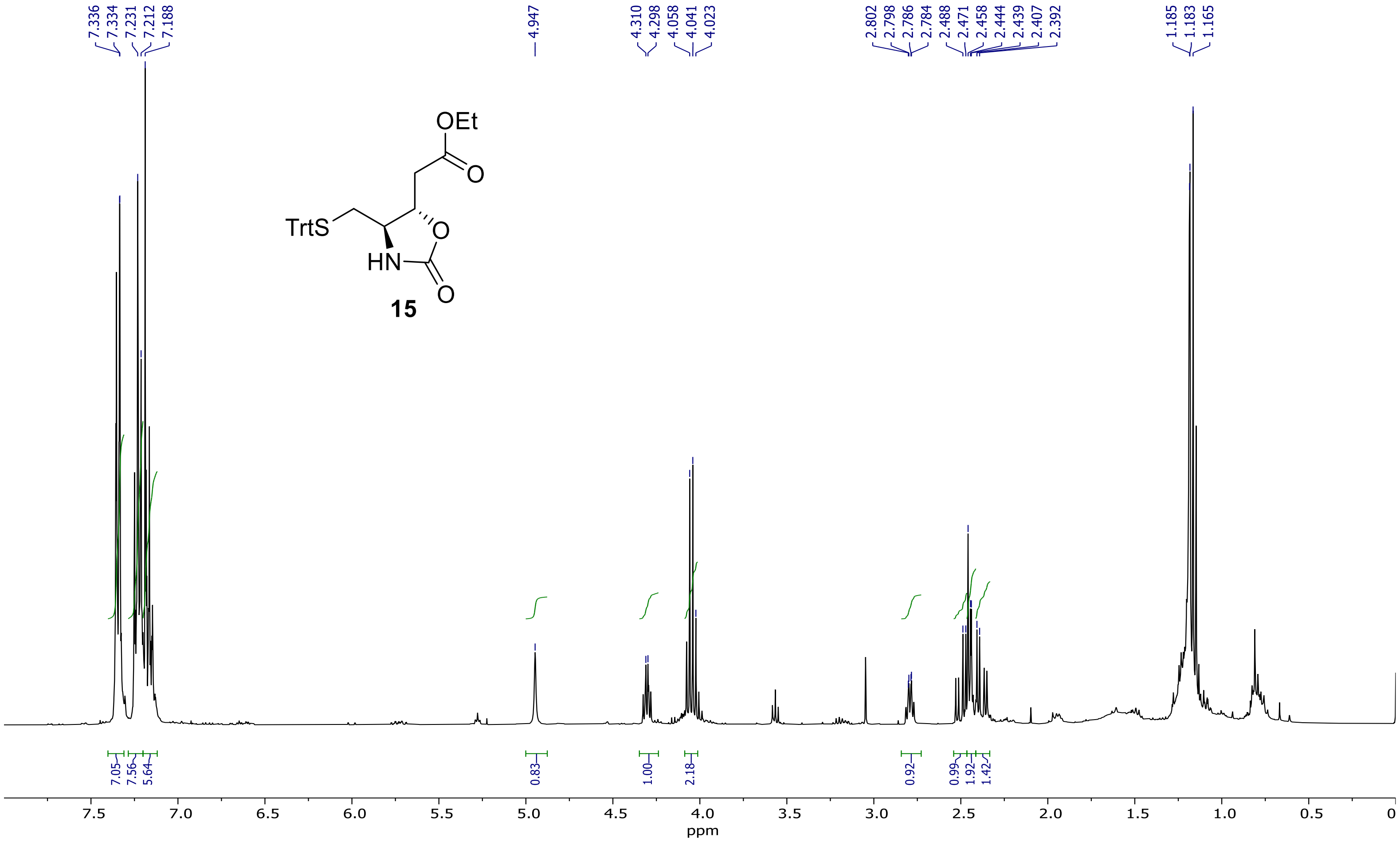

^{13}C NMR (100.13 MHz, $CDCl_3$) of **15**

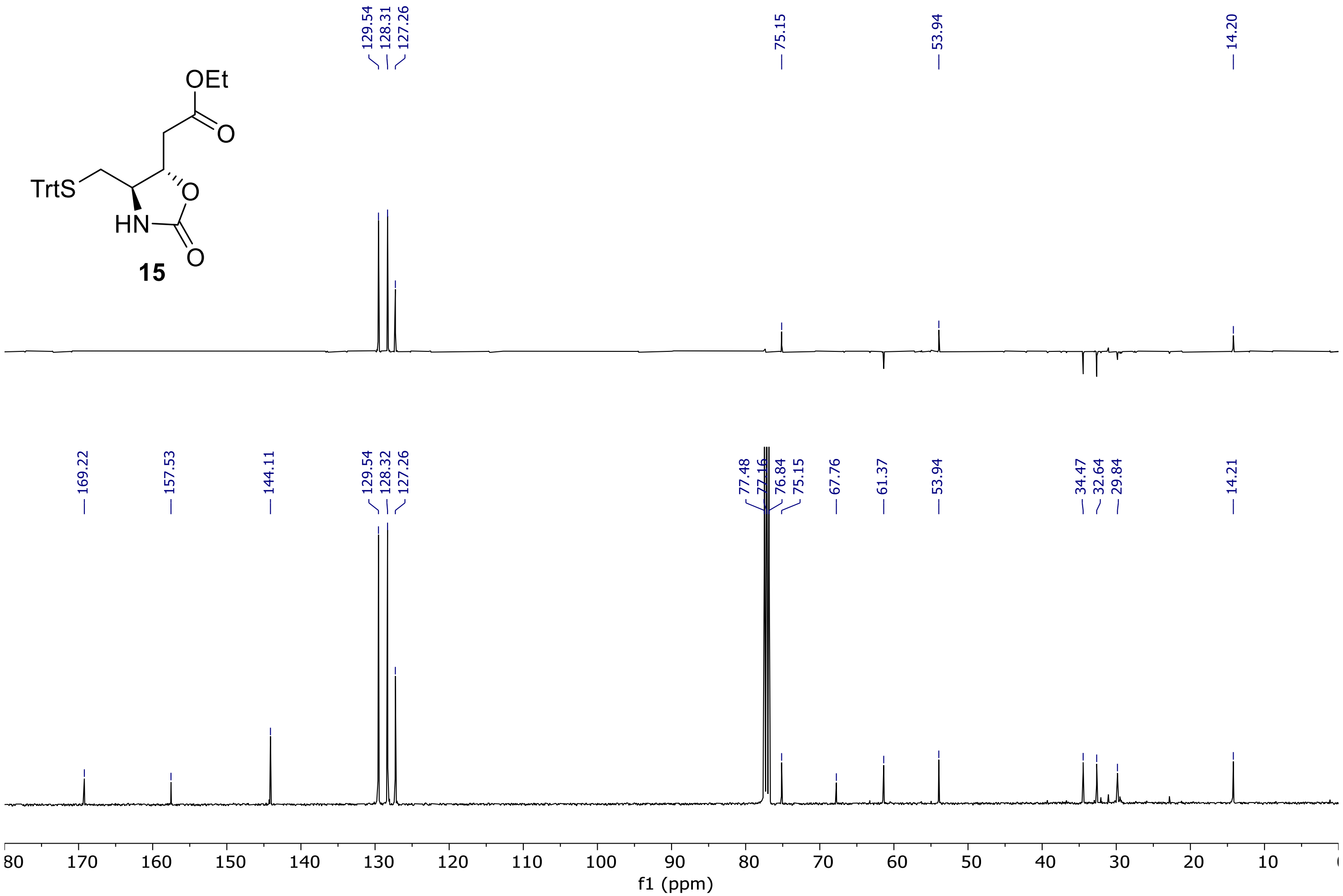

^{1}H-^{1}H COSY (100.13 MHz, $CDCl_3$) of **15**

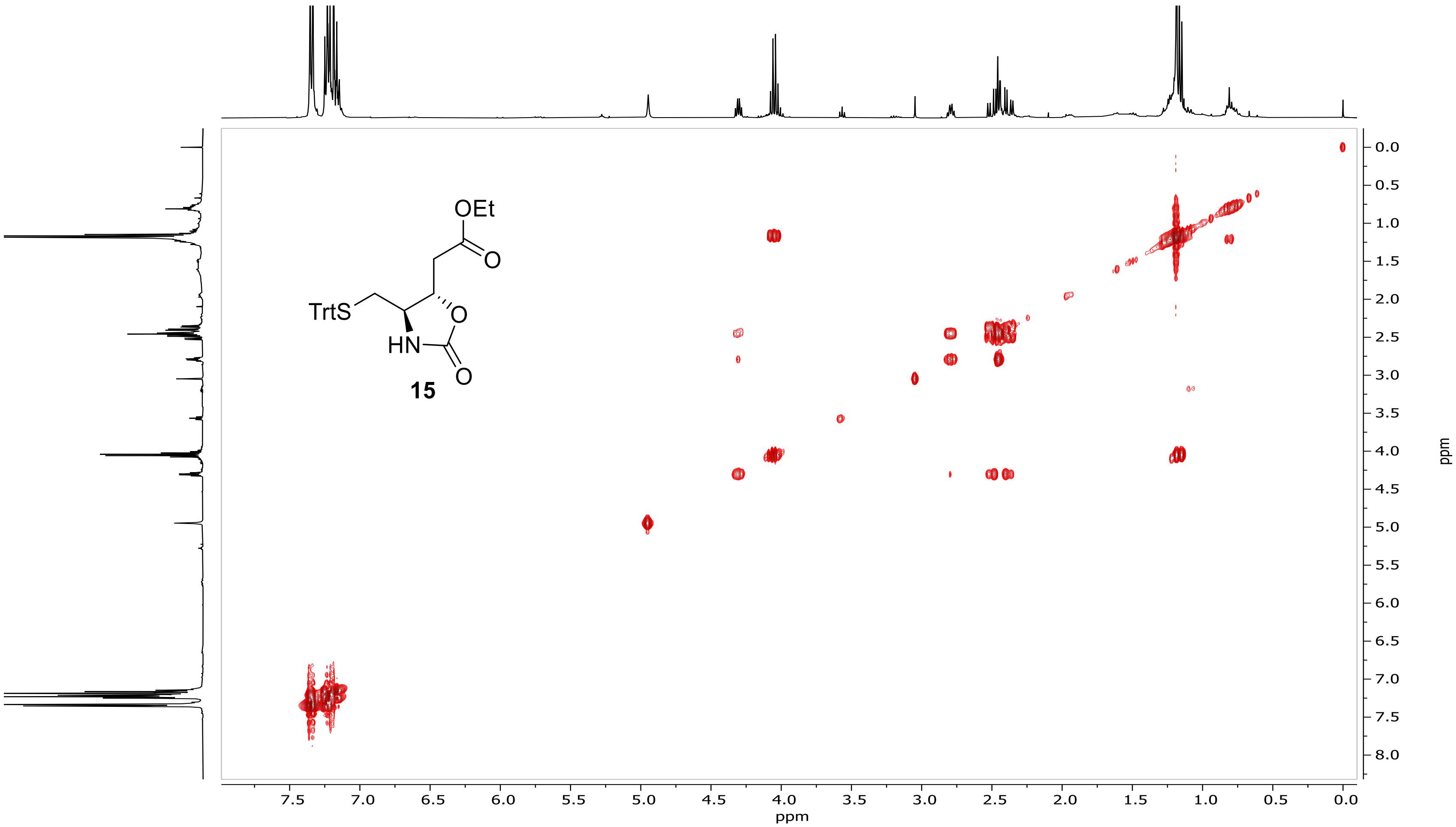

HSQC (100.13 MHz, $CDCl_3$) of **15**

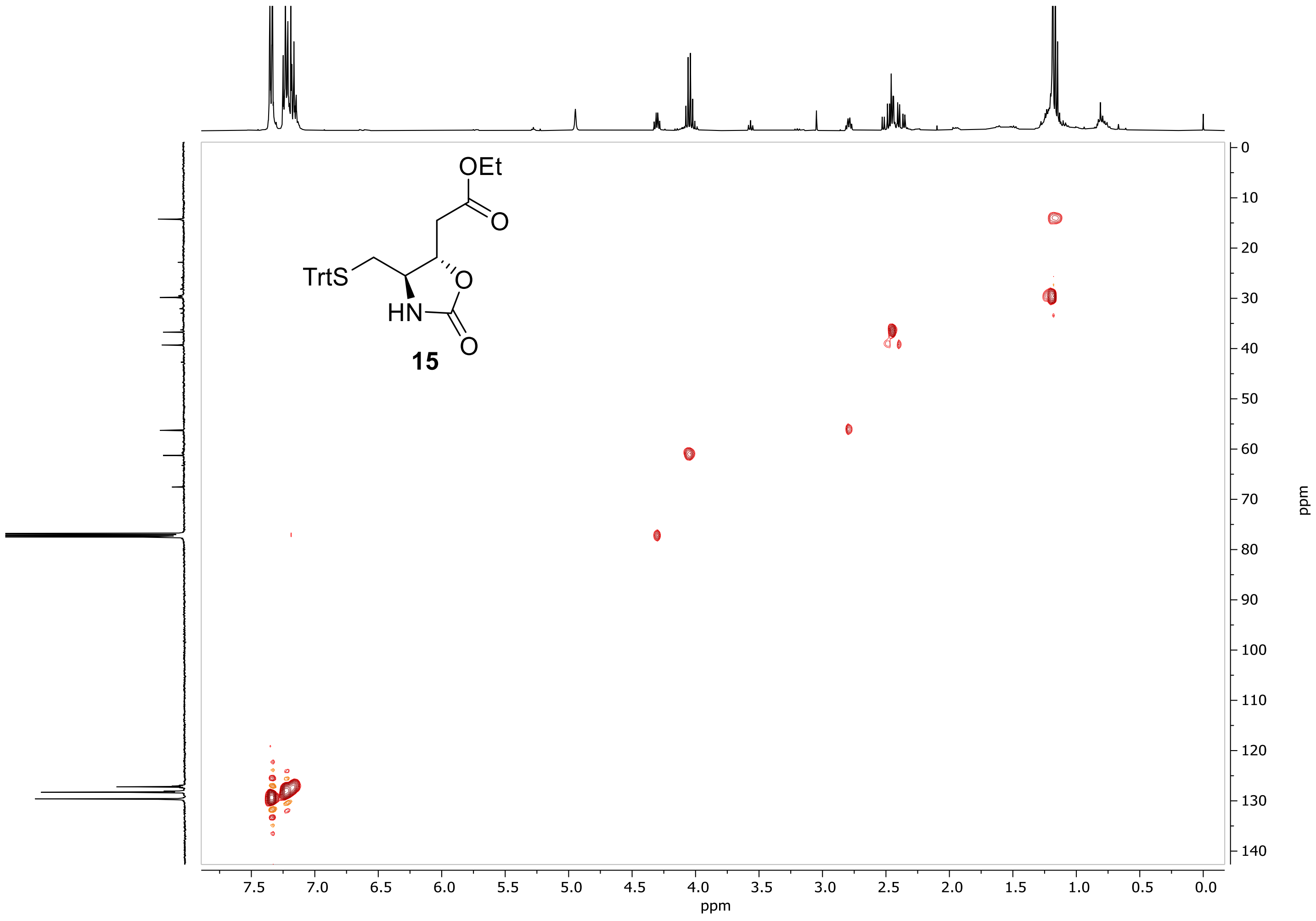

^{1}H-^{1}H NOESY of **15**

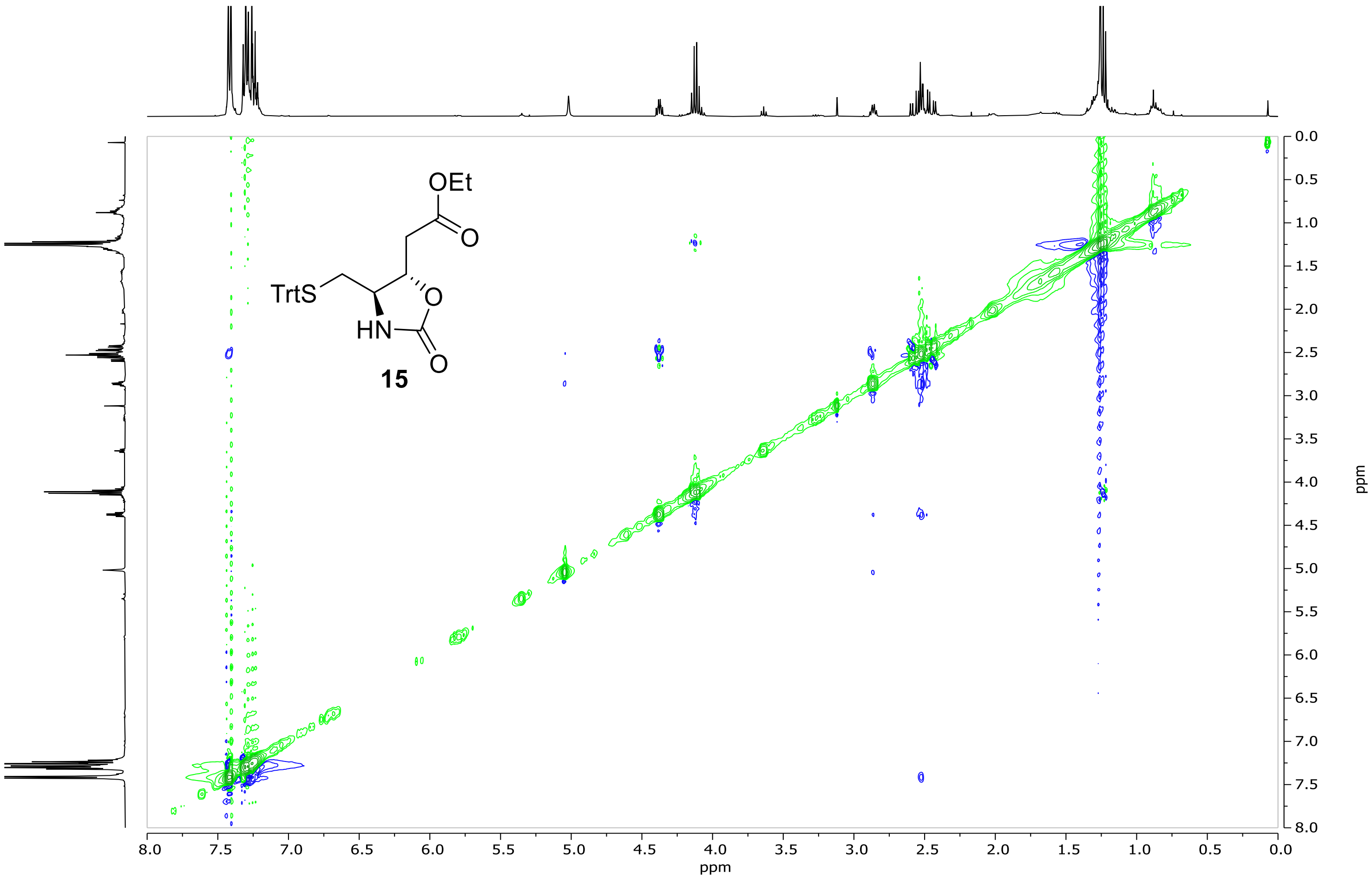

(+)-HRESIMS of **15**: Ion: *m/z*: 484.1563 ([M+Na]$^+$)

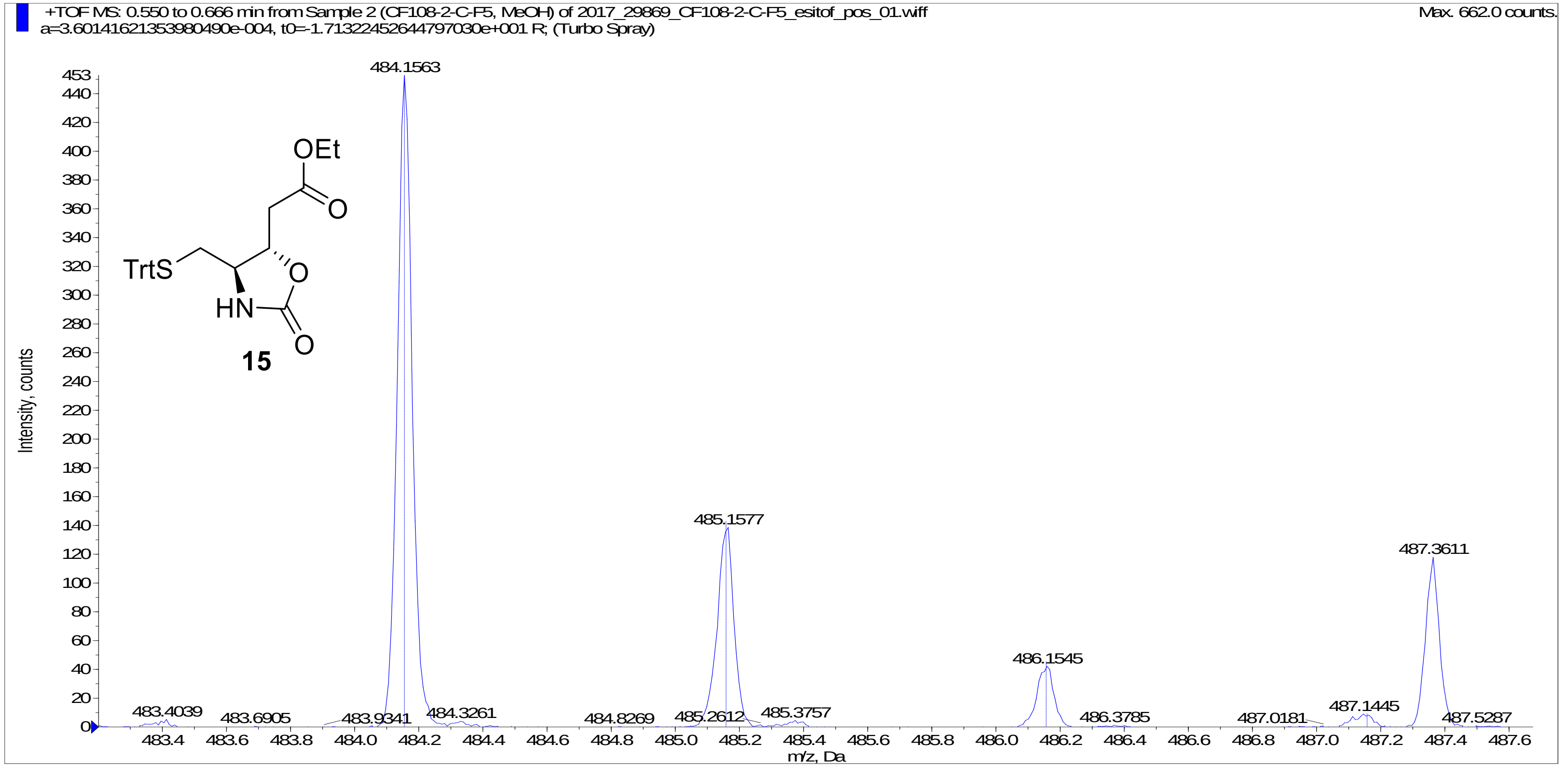

Formula	Calc m/z	Δ, mDa	Δ, ppm	DBE
$C_{27}H_{27}NO_4NaS$	484.1553	0.9984	2.0622	14.5

2.8 NMR and MS of 16

^{1}H NMR (400 MHz, $CDCl_3$) of **16**

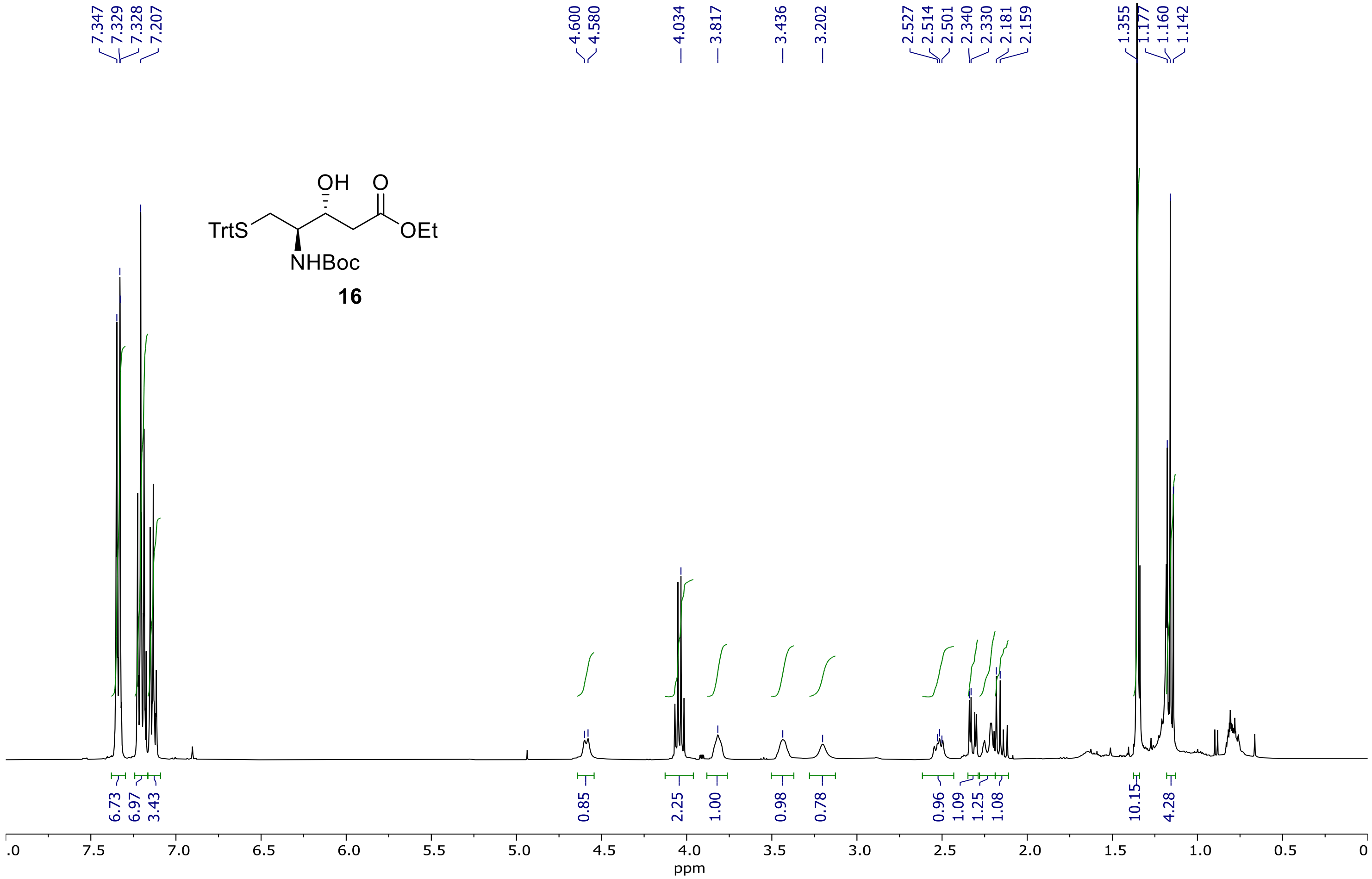

^{13}C NMR (100.13 MHz, $CDCl_3$) of **16**

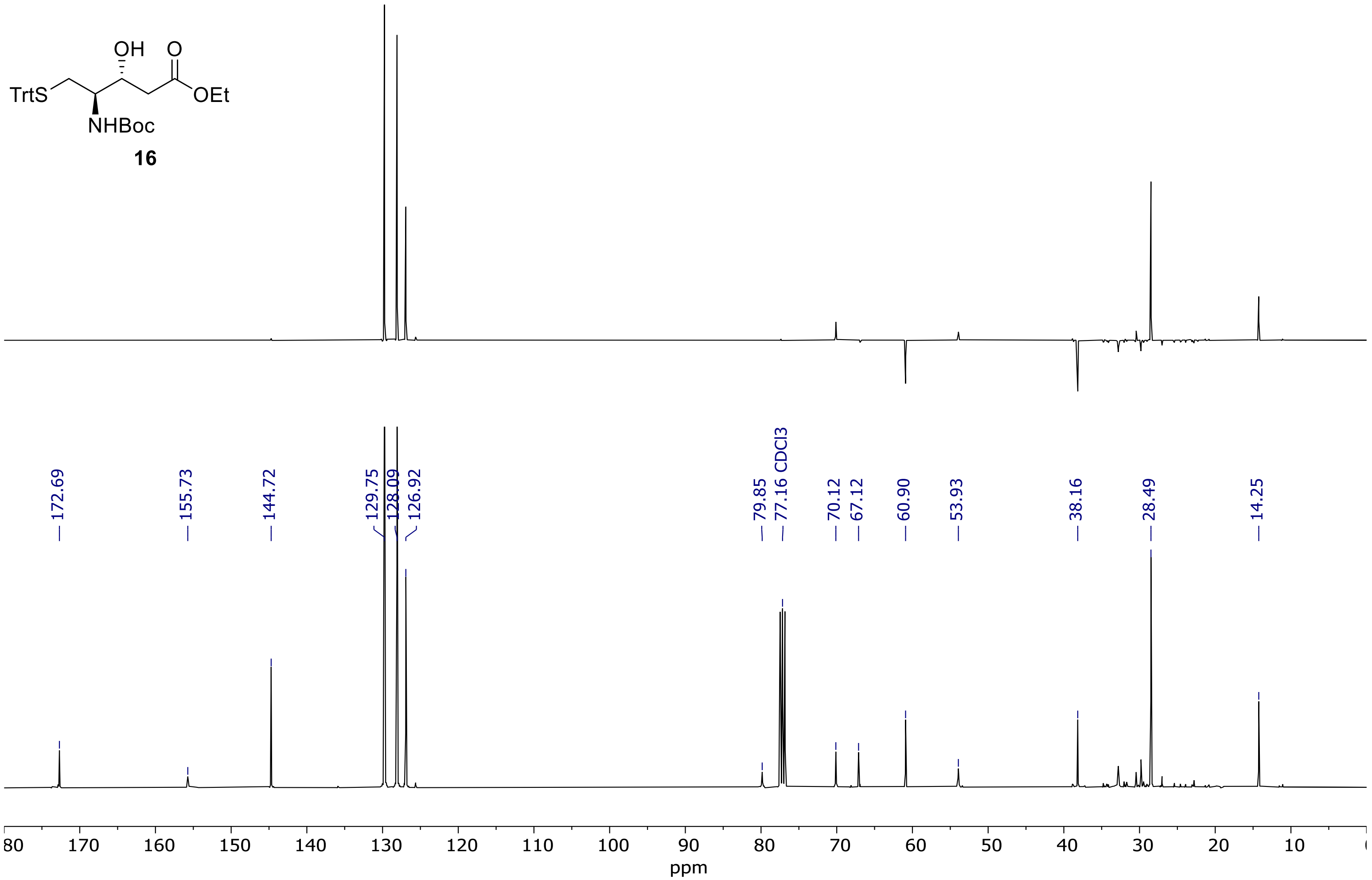

^{1}H-^{1}H COSY (400.13 MHz, $CDCl_3$) of **16**

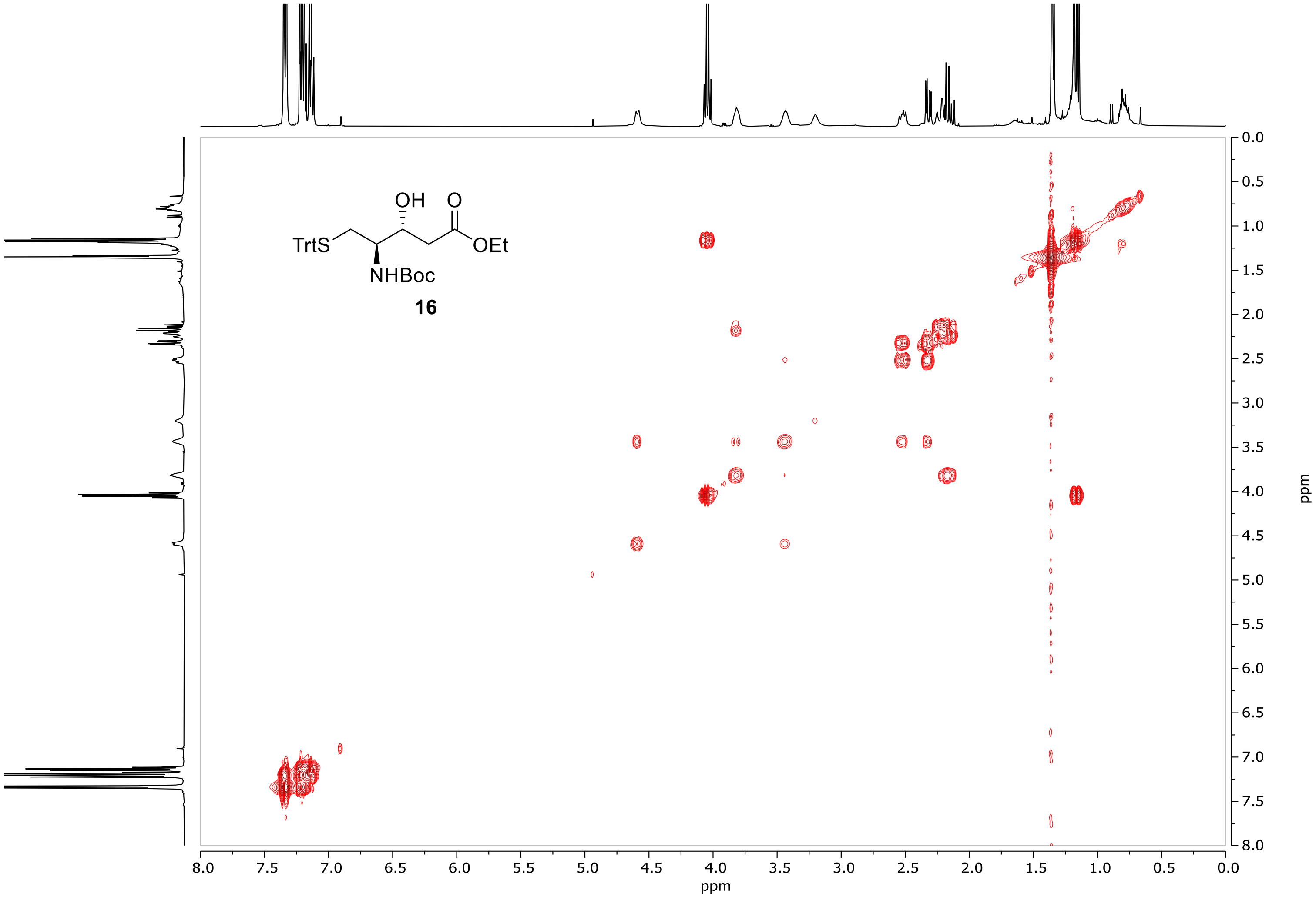

HSQC (400.13 MHz, $CDCl_3$) of **16**

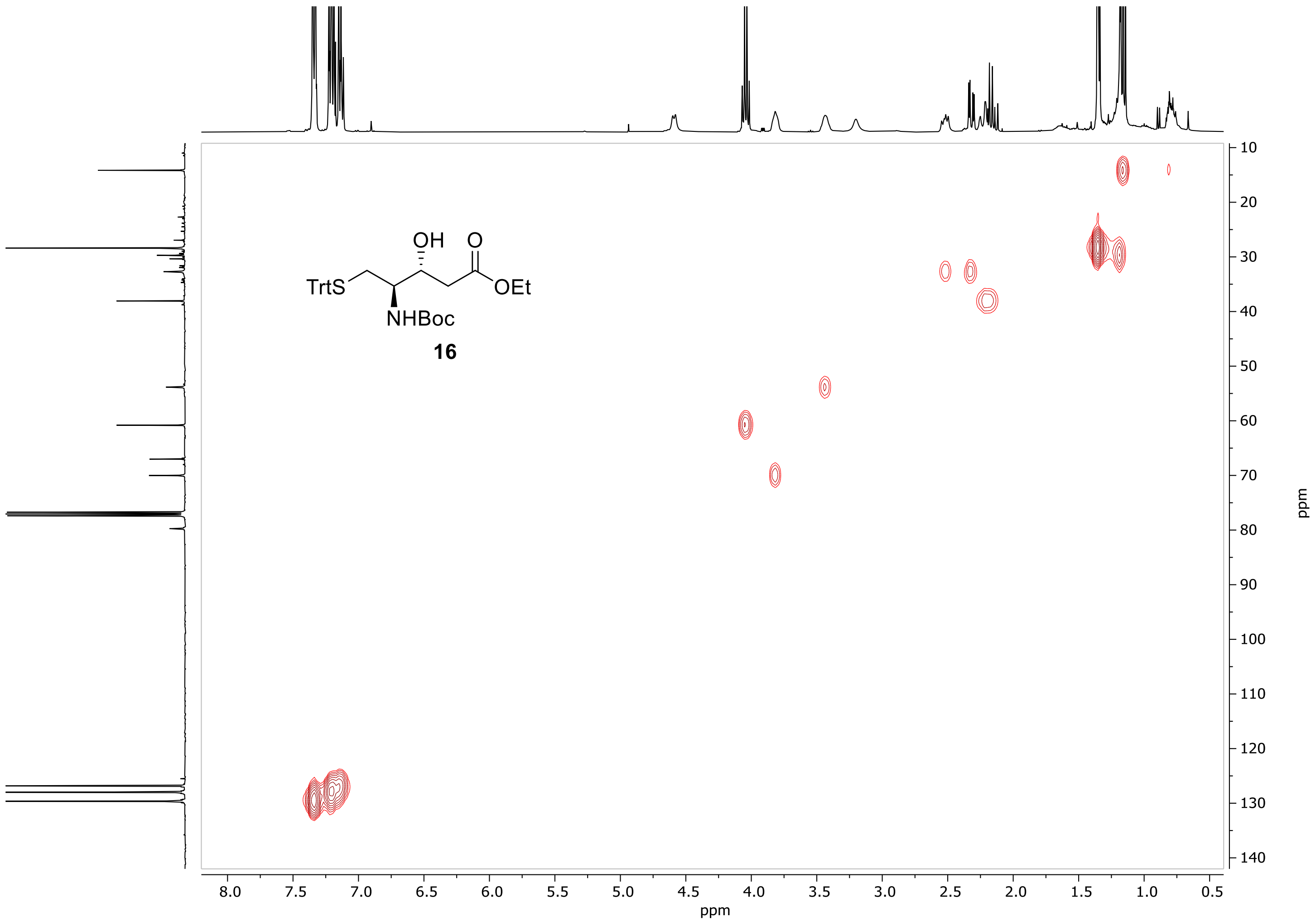

HRESIMS of **16**: Ion: *m/z*: 558.2302 ([M+Na]$^+$)

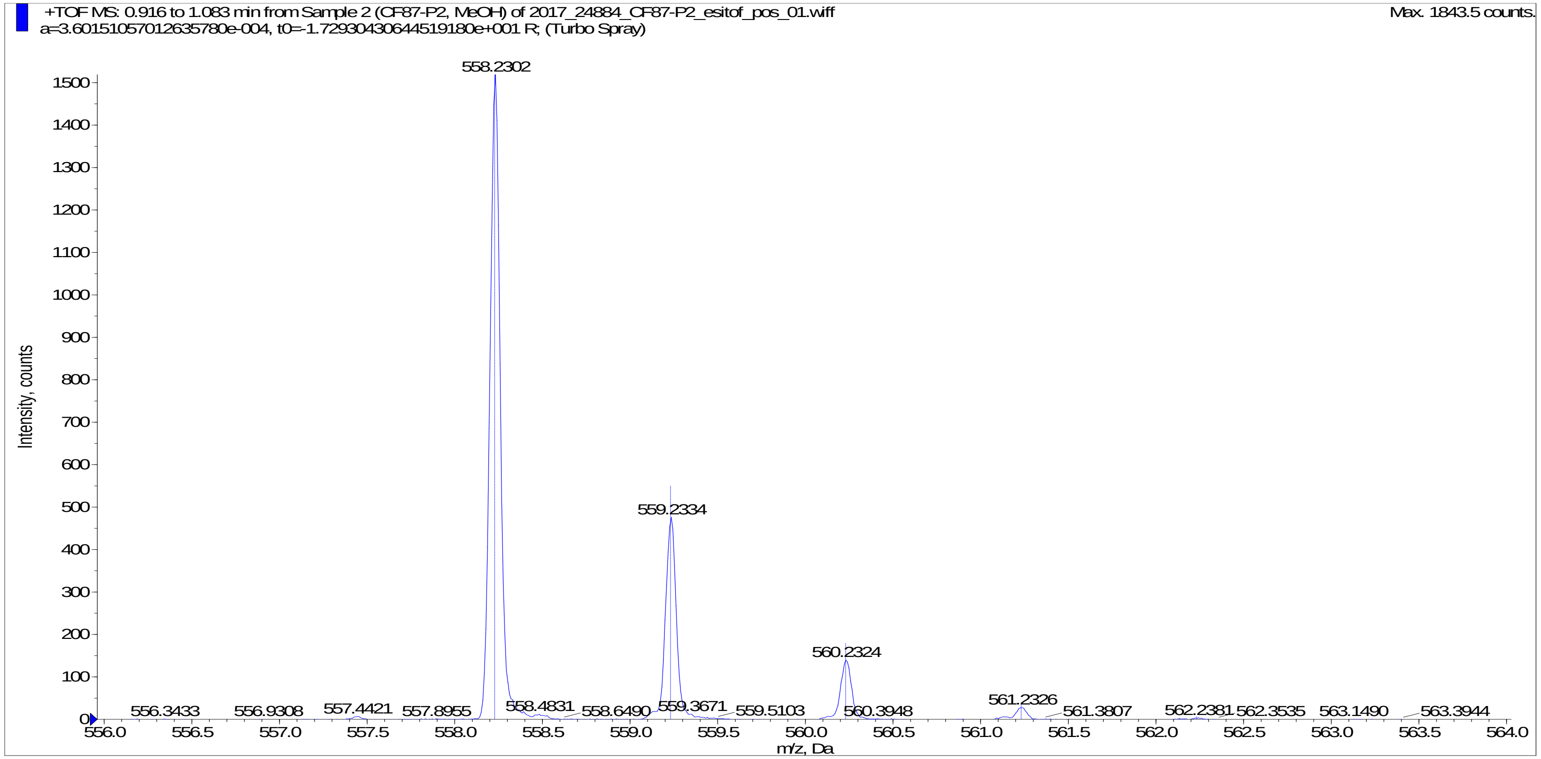

Formula	Calc m/z	Δ, mDa	Δ, ppm	DBE
$C_{31}H_{37}NO_5NaS$	558.2284	1.7333	3.1051	13.5

2.9 NMR and MS of 17

^1H NMR (400.13 MHz, $CDCl_3$) of **17**

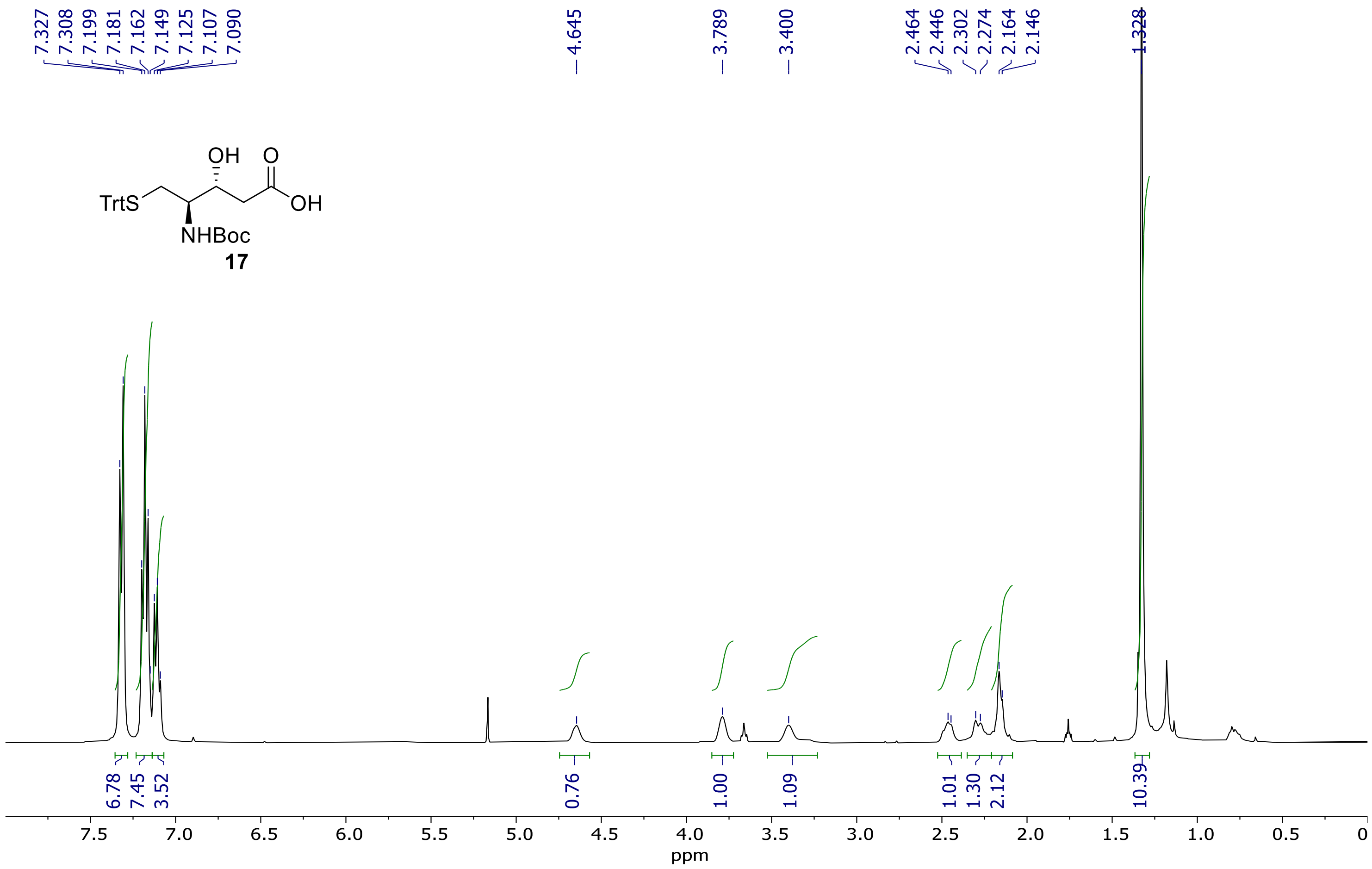

^{13}C NMR (100.13 MHz, $CDCl_3$) of **17**

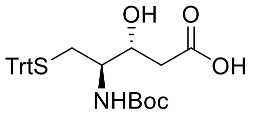

17

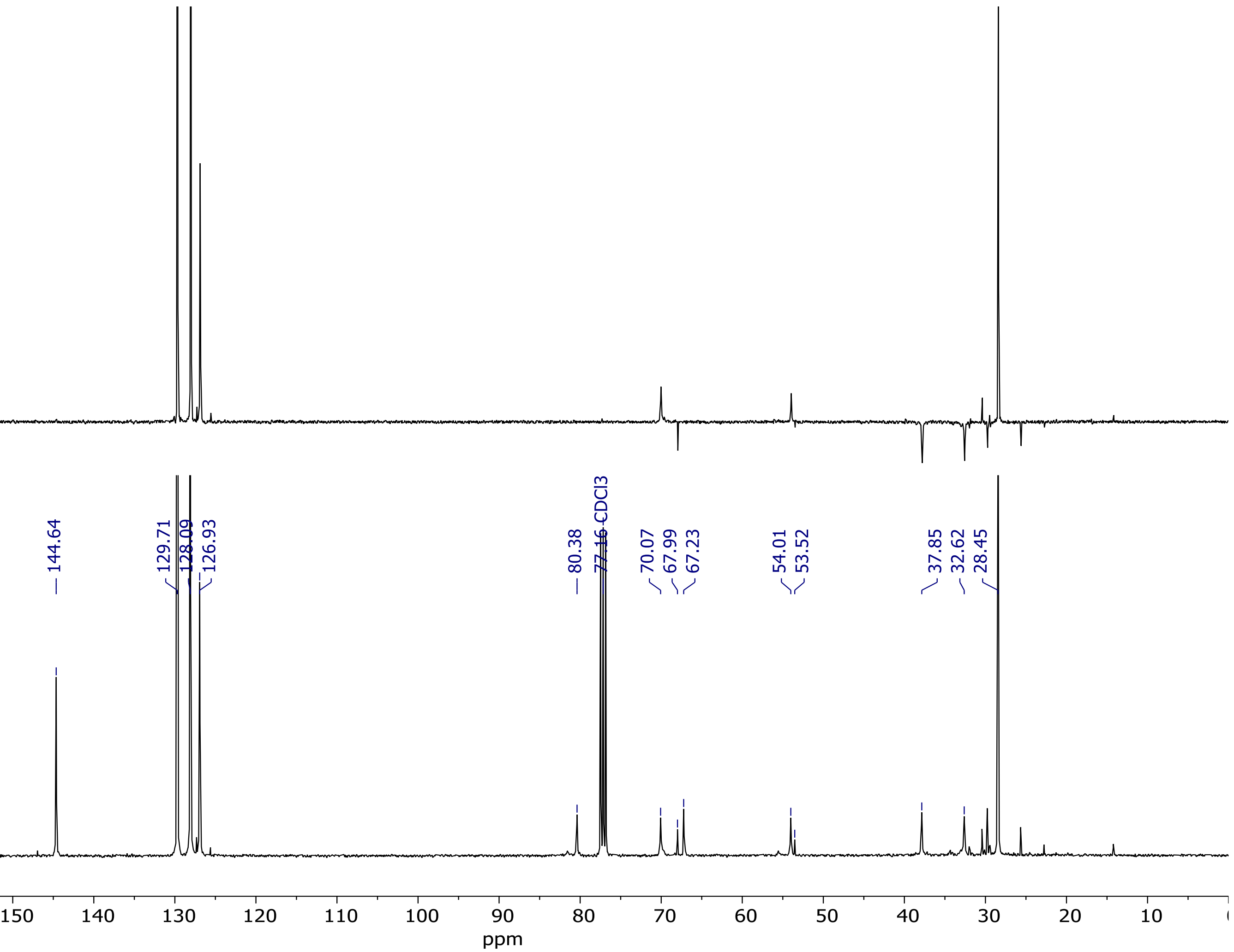

^{1}H-^{1}H COSY (400.13 MHz, $CDCl_3$) of **17**

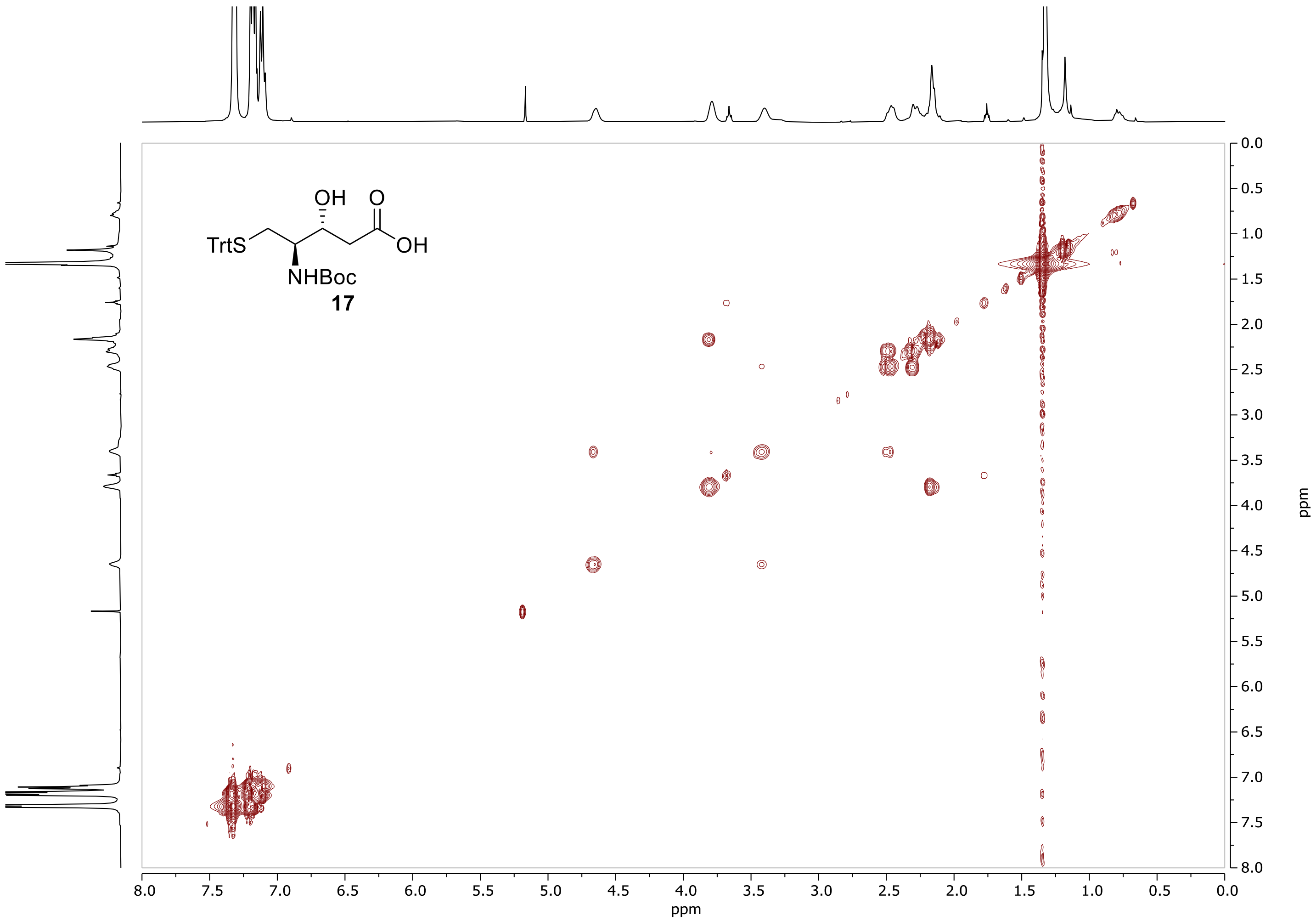

HSQC (400.13 MHz, $CDCl_3$) of **17**

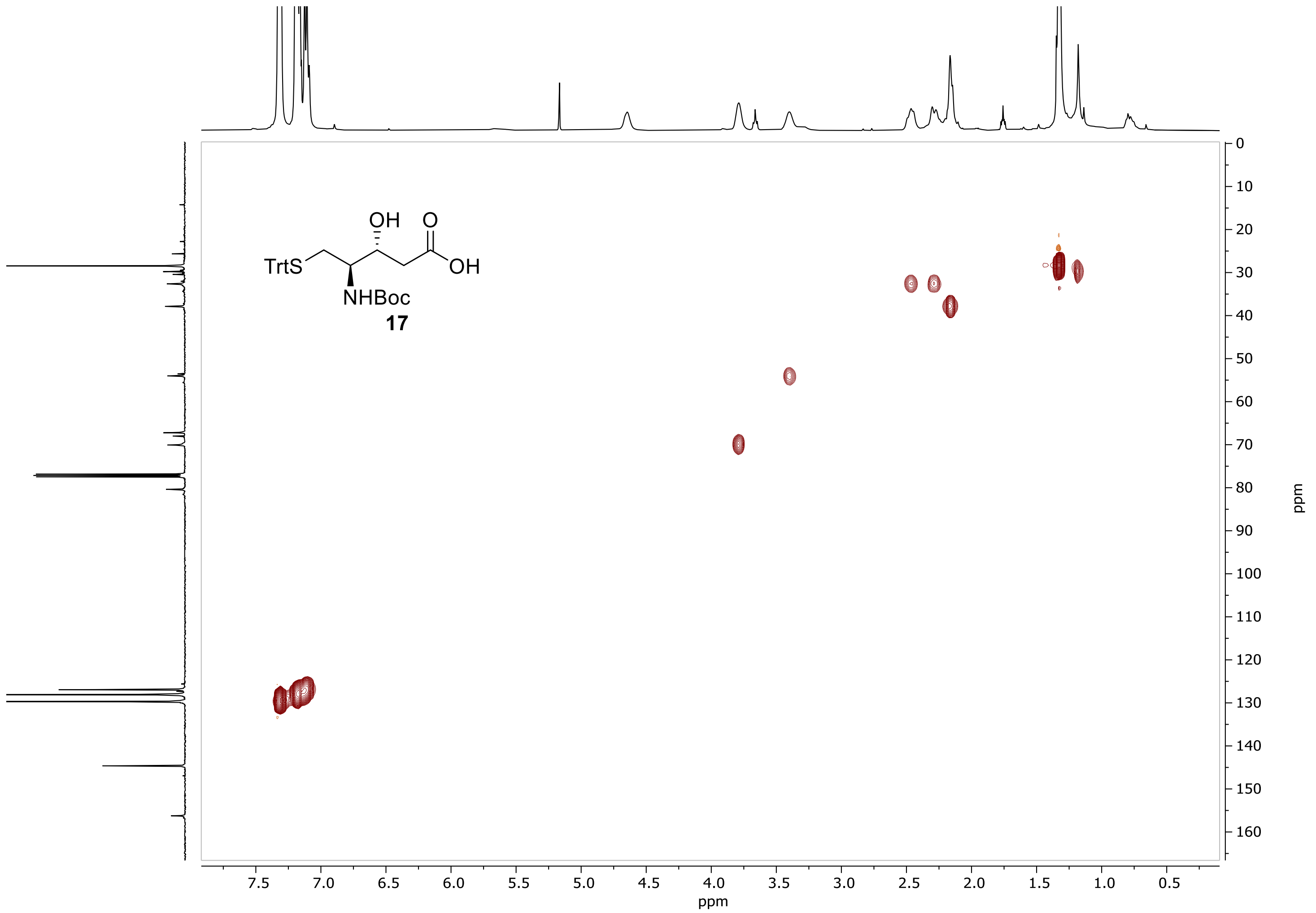

(+)-HRESIMS of **17**: Ion: *m/z*: 530.1968 [M+Na]$^+$

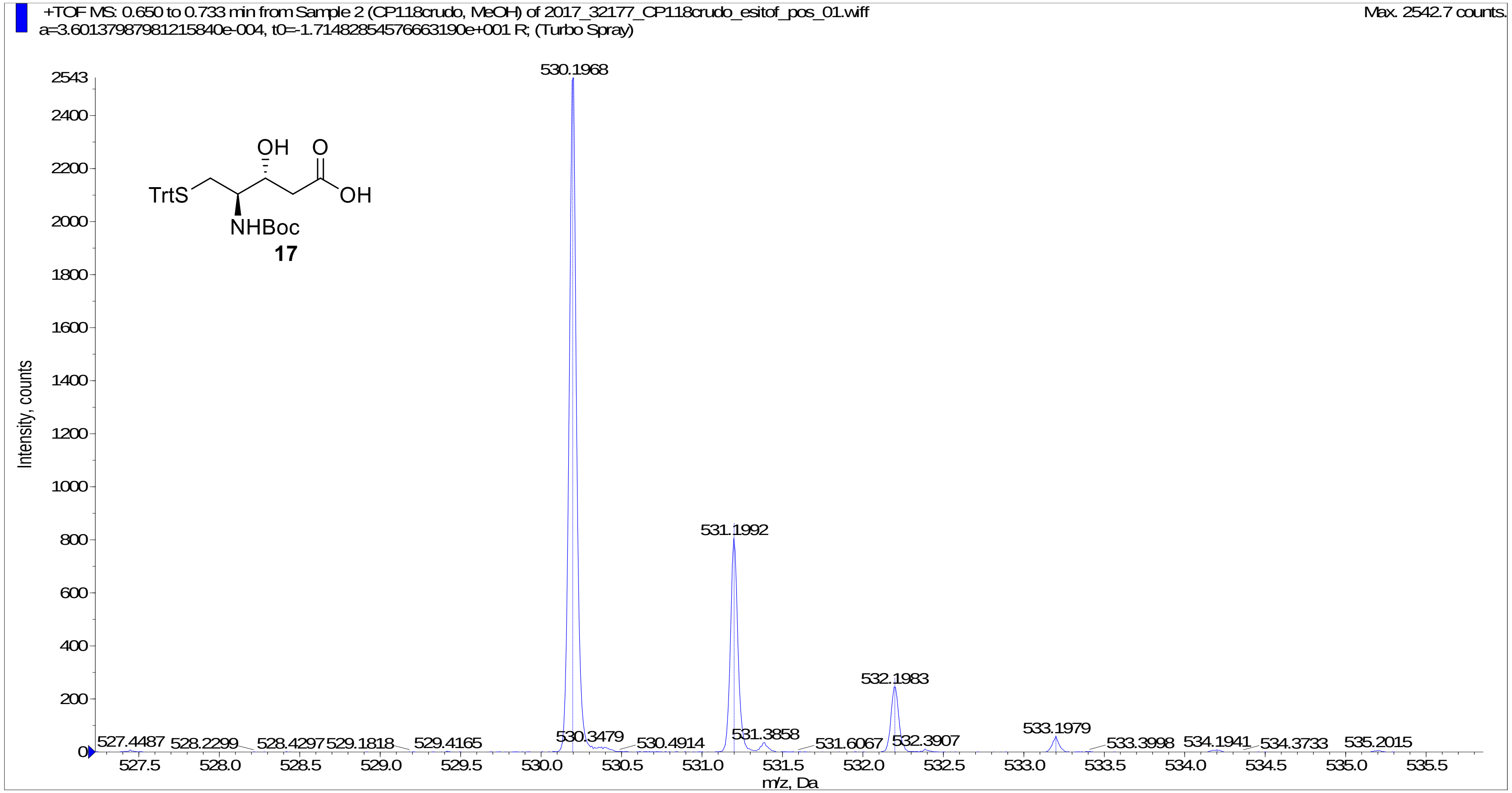

Formula	Calc m/z	Δ, mDa	Δ, ppm	DBE
$C_{29}H_{33}NO_5NaS$	530.1971	-0.3664	-0.6911	13.5

2.10 NMR and MS of 18

^{1}H NMR (400.13 MHz, $CDCl_3$) of **18**

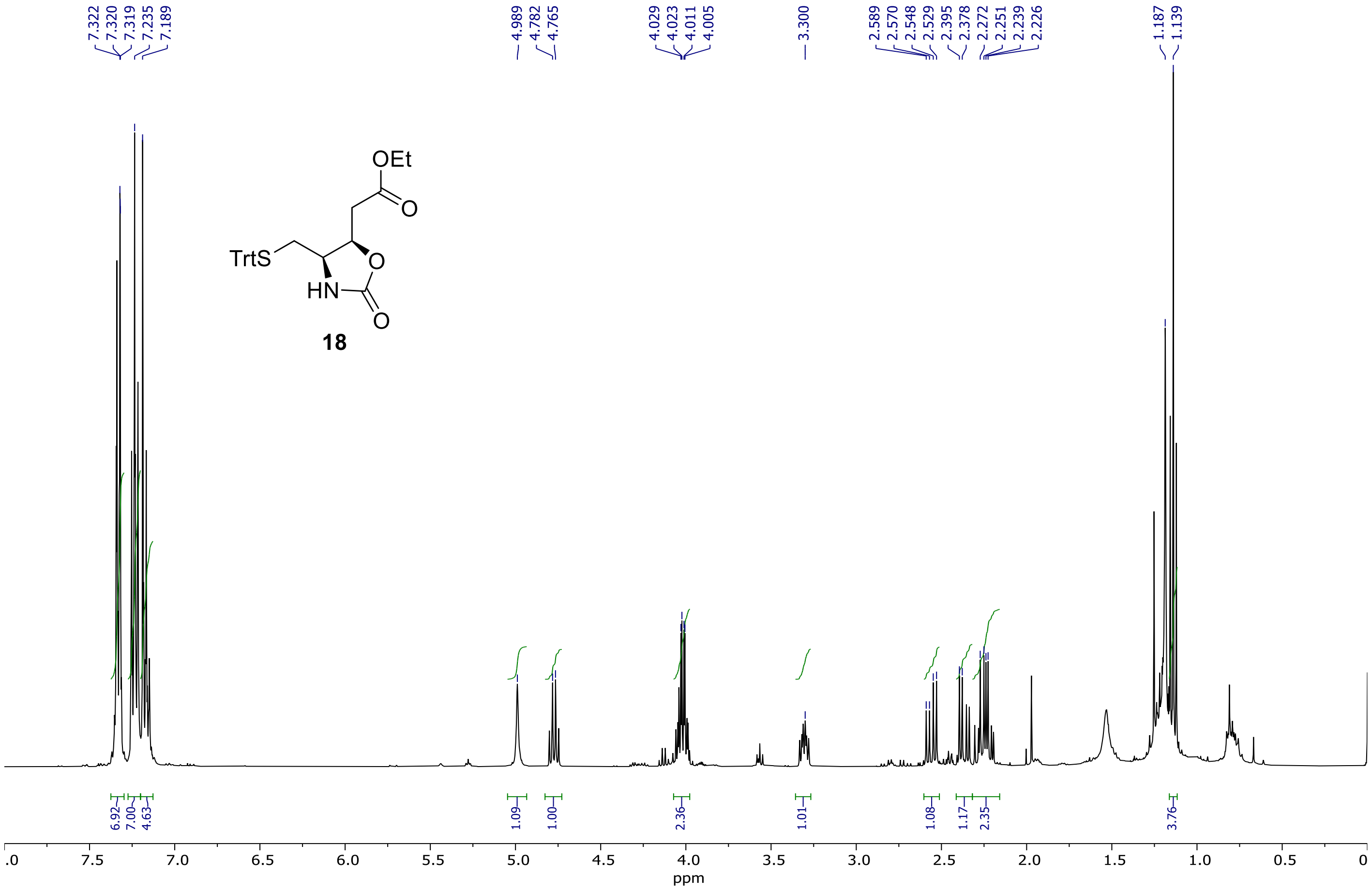

^{13}C NMR (100.13 MHz, $CDCl_3$) of **18**

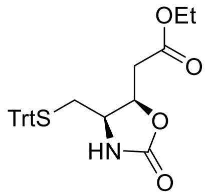

18

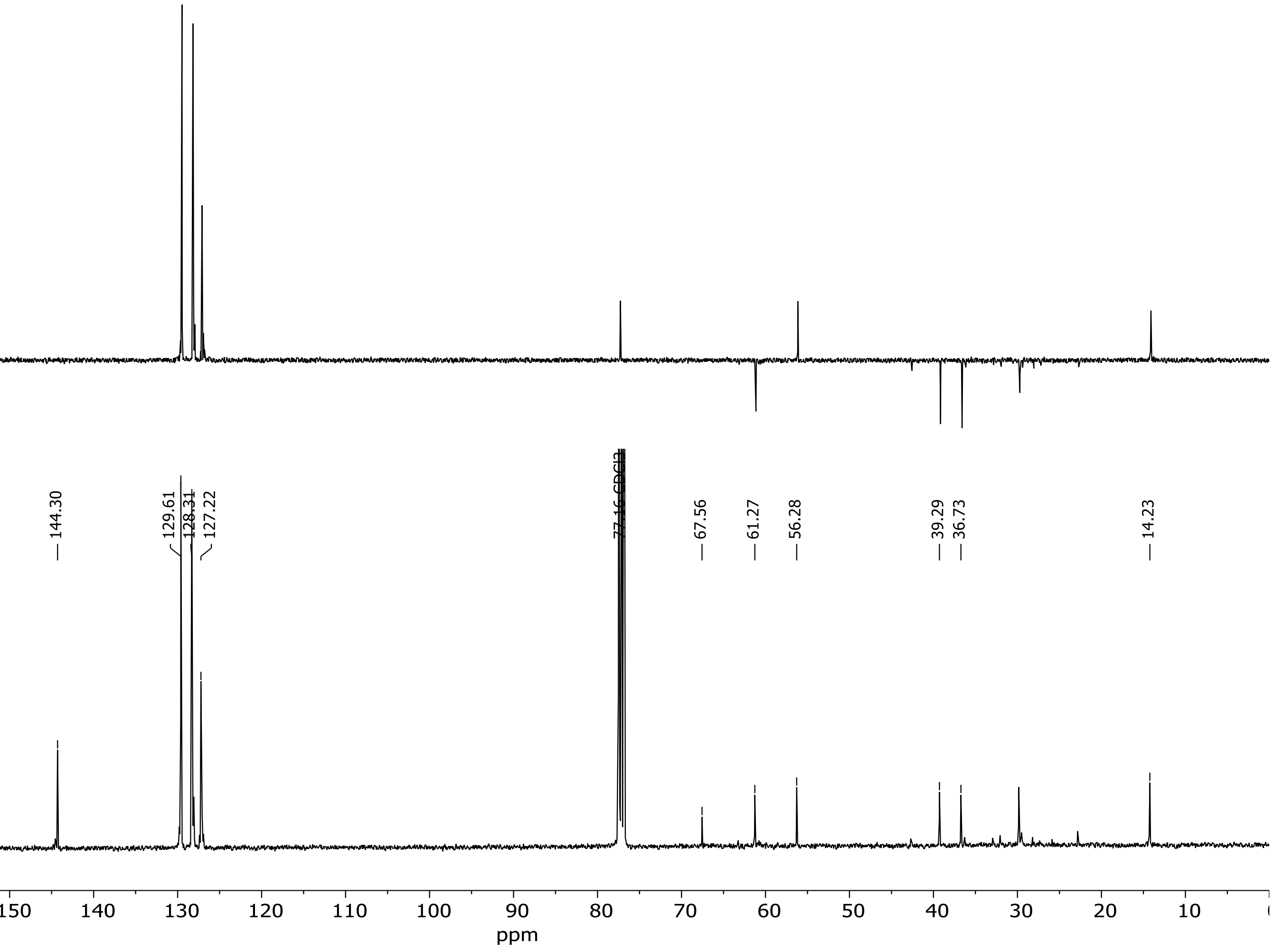

^{1}H-^{1}H COSY (400.13 MHz, $CDCl_3$) of **18**

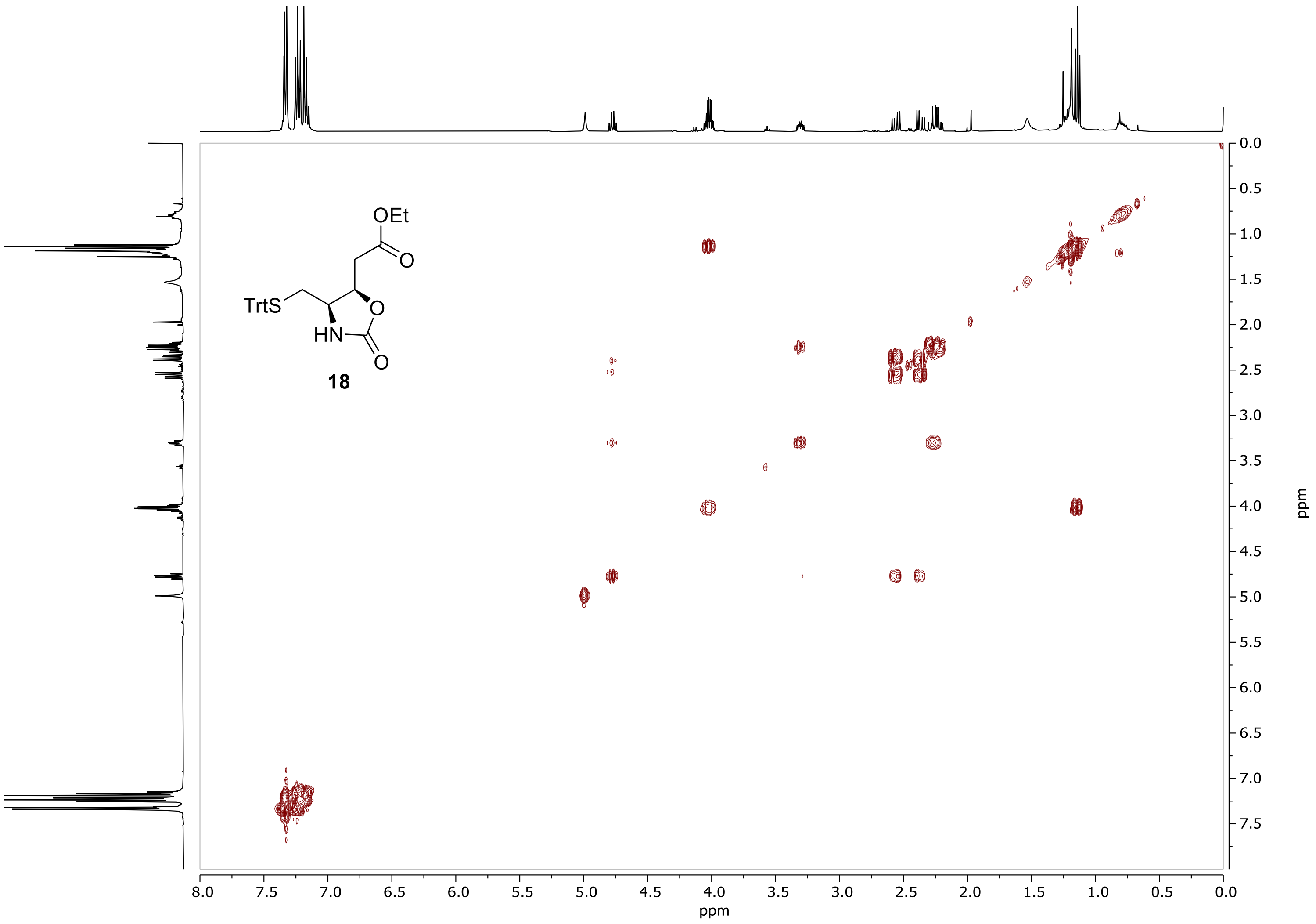

HSQC (400.13 MHz, $CDCl_3$) of **18**

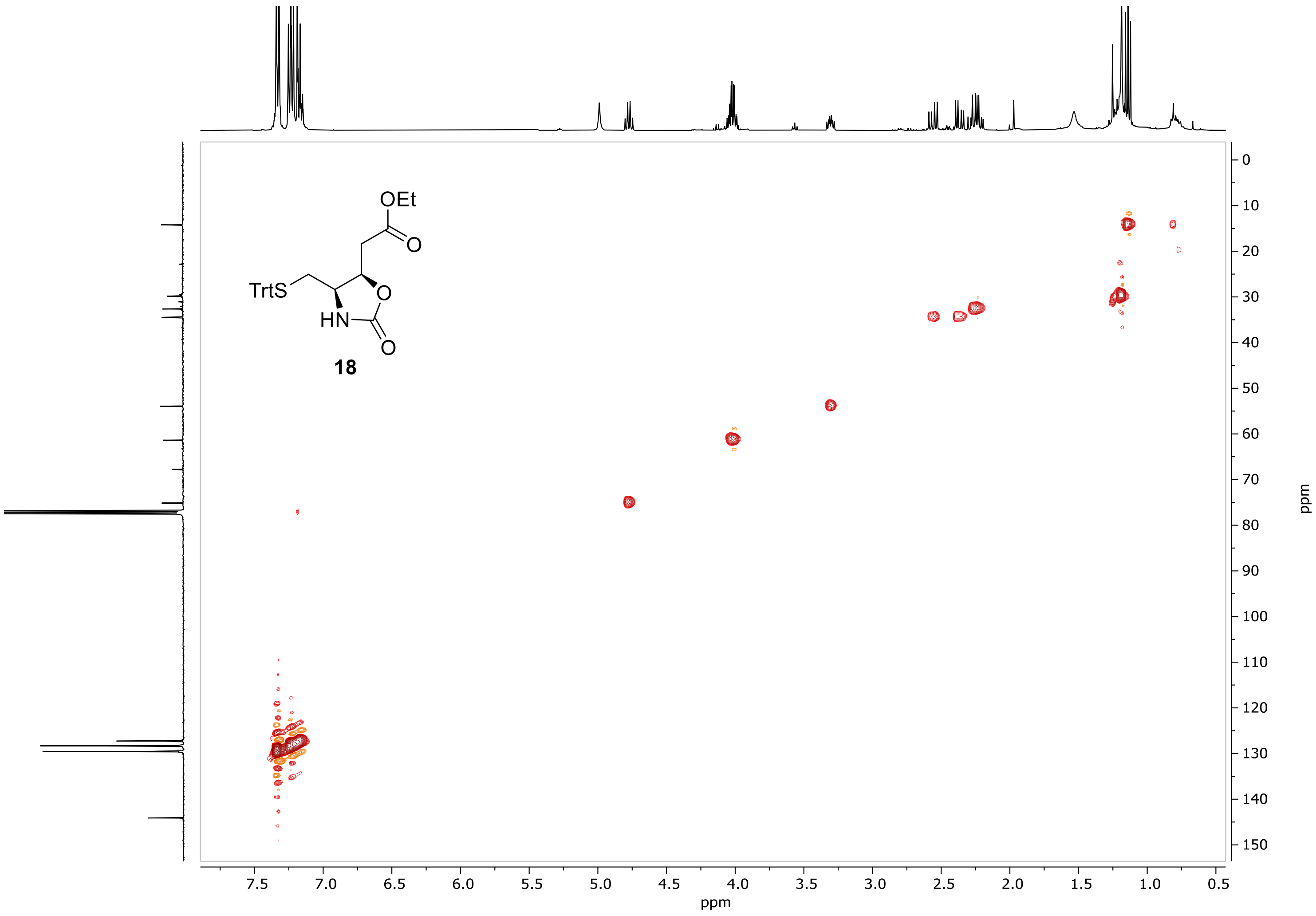

^{1}H-^{1}H NOESY of **18**

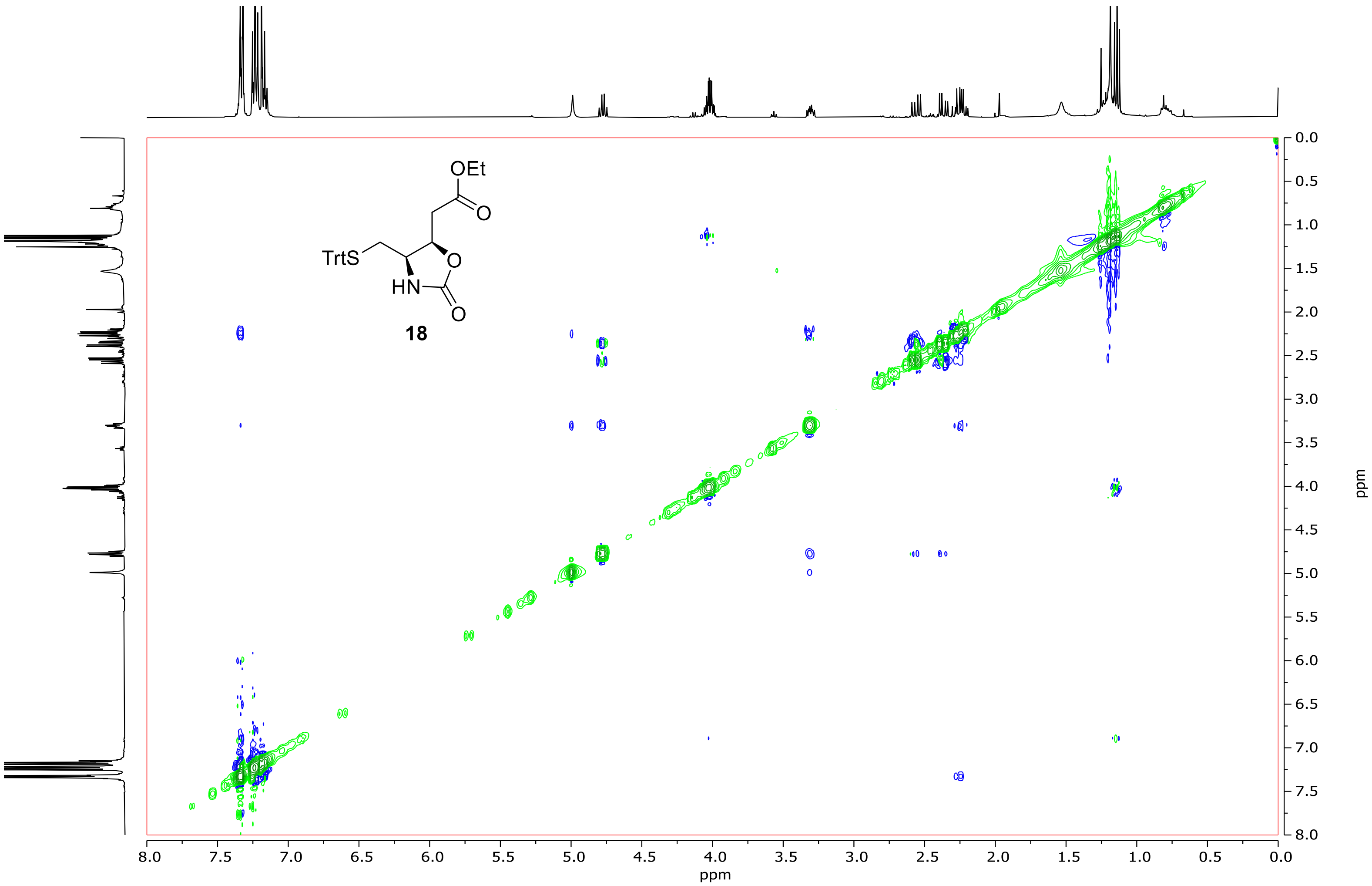

(+)-HRESIMS of **18**: Ion: *m/z*: 484.1574 $[M+Na]^+$

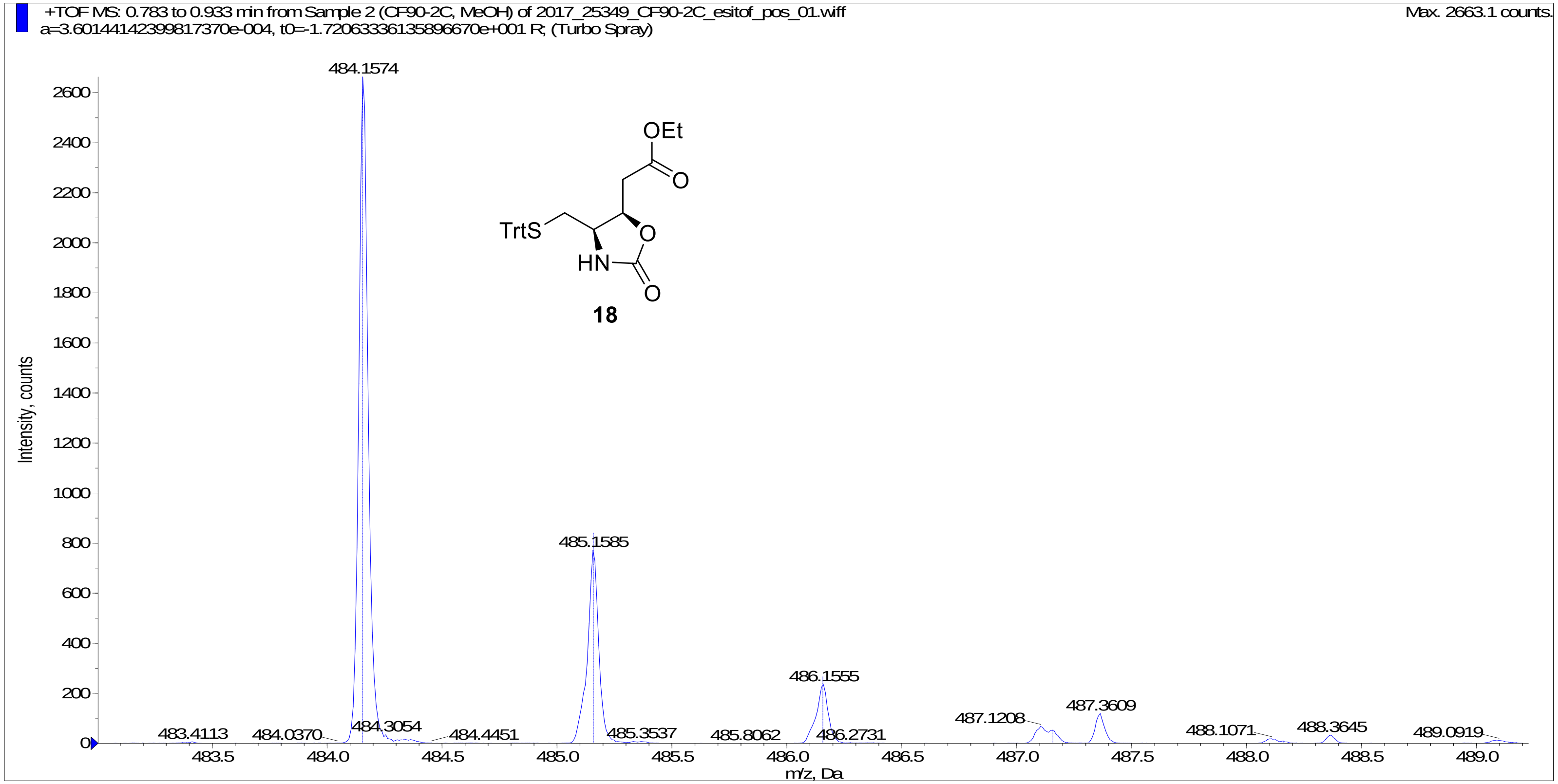

Formula	Calc m/z	Δ, mDa	Δ, ppm	DBE
$C_{27}H_{27}NO_4NaS$	484.1553	2.0984	4.3341	14.5

3. NMR and MS of intermediates of the synthesis of thiazole *syn* epimer 30 (Scheme 3)

3.1 NMR and MS of compound 19

^{1}H NMR (300.13 MHz, $CDCl_3$) of **19**

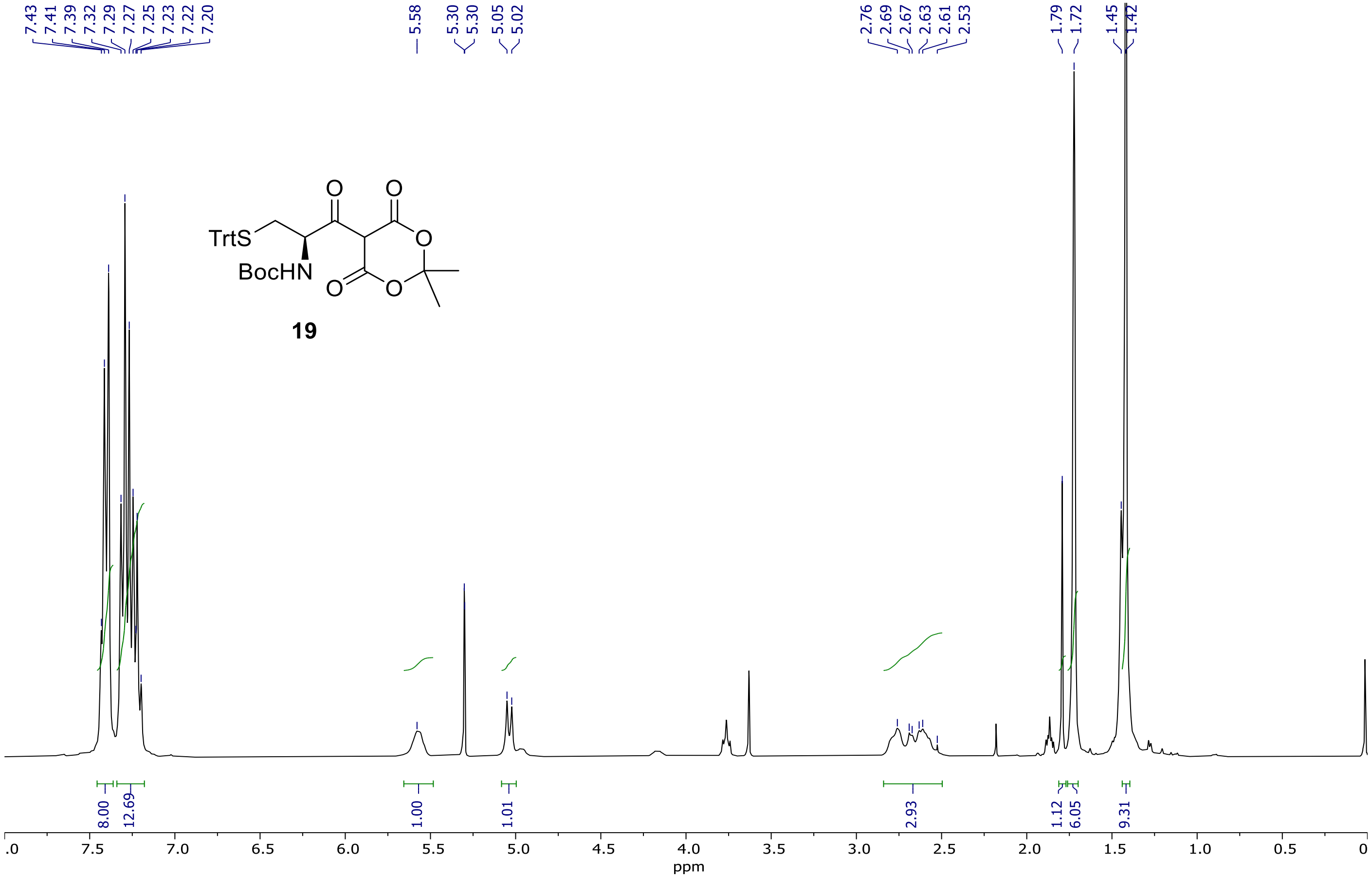

^{13}C NMR (75 MHz, $CDCl_3$) of **19**

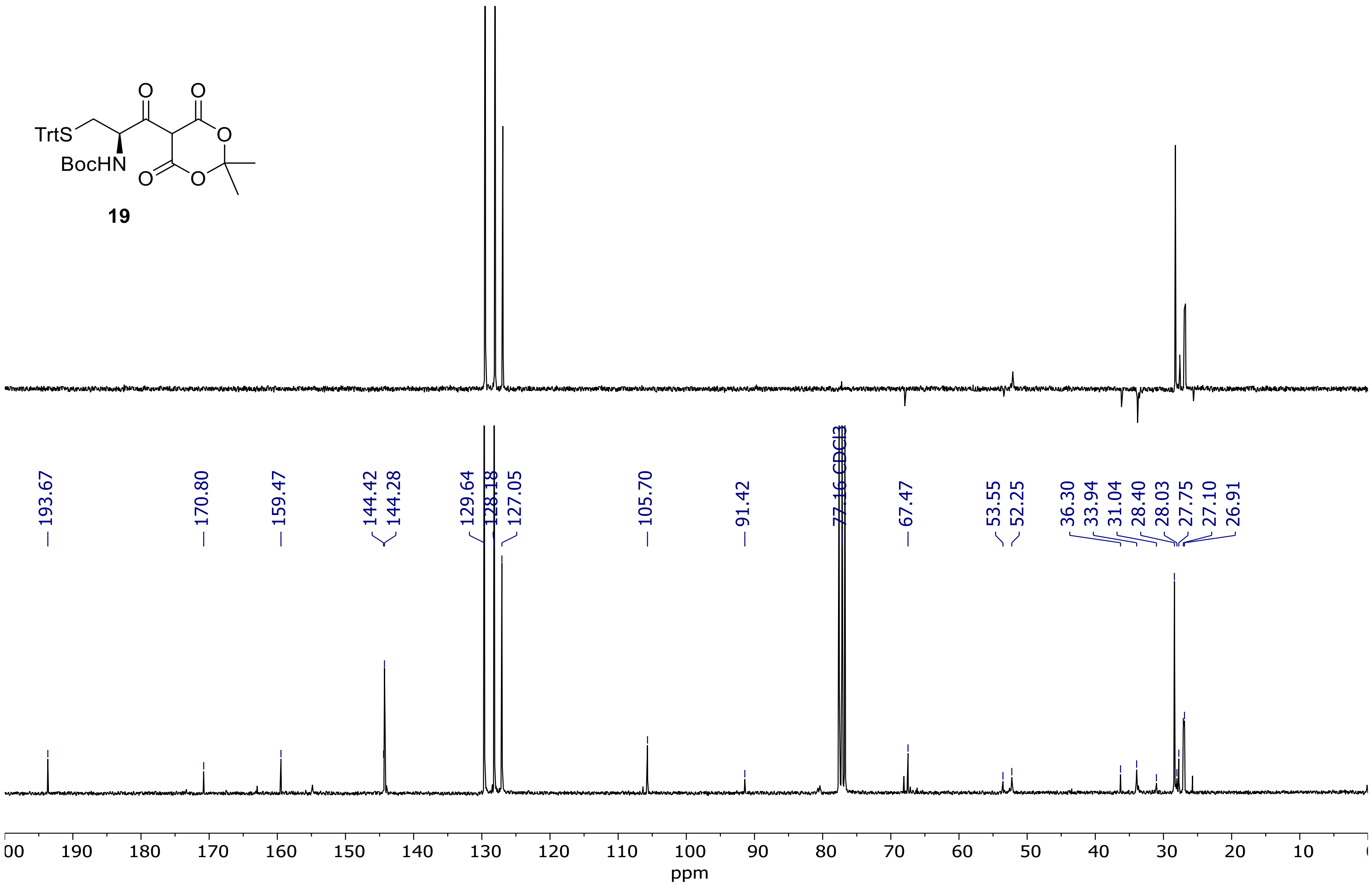

^{1}H-^{1}H COSY (300.13 MHz, $CDCl_3$) of **19**

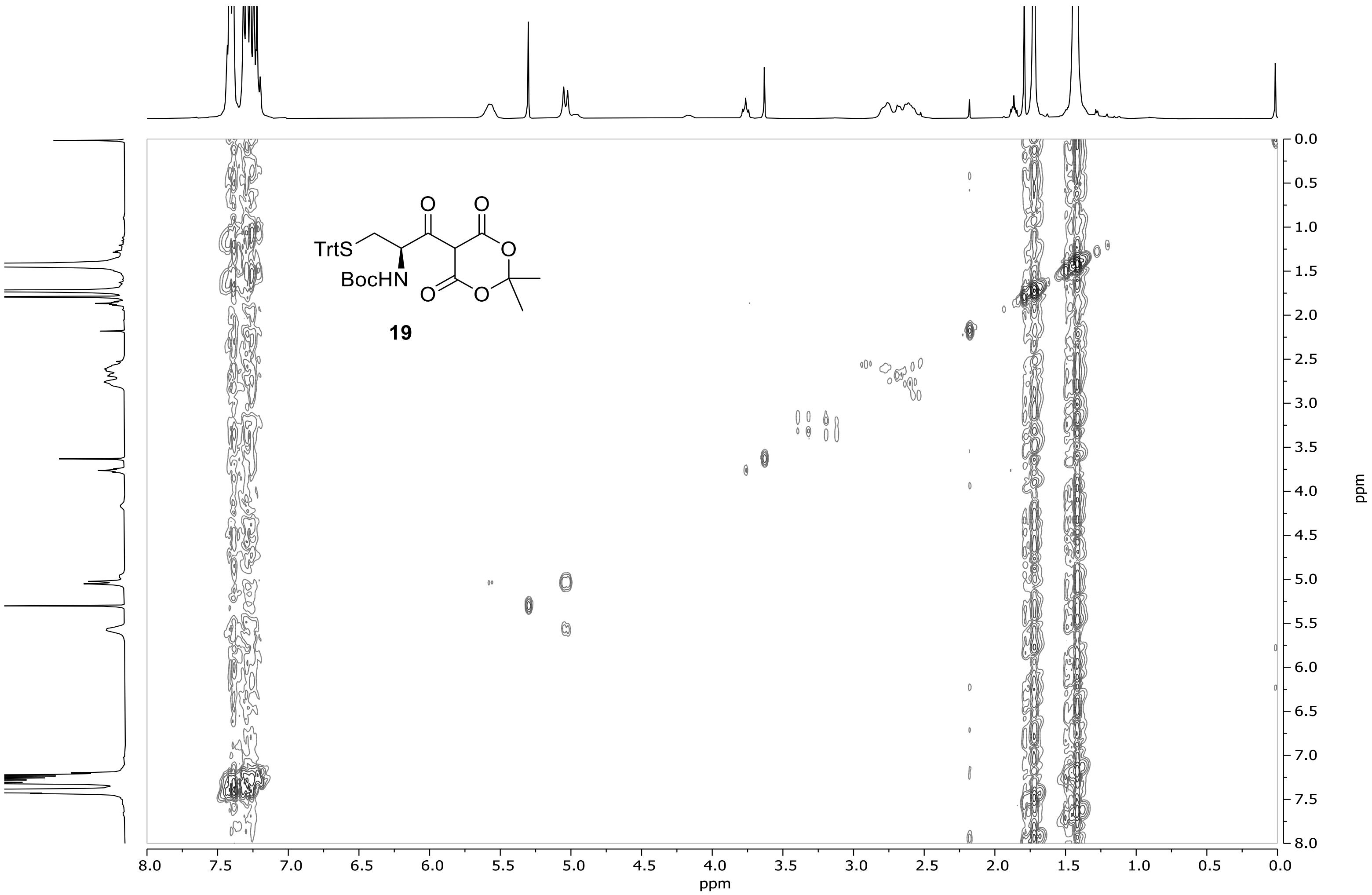

HSQC (300.13 MHz, $CDCl_3$) of **19**

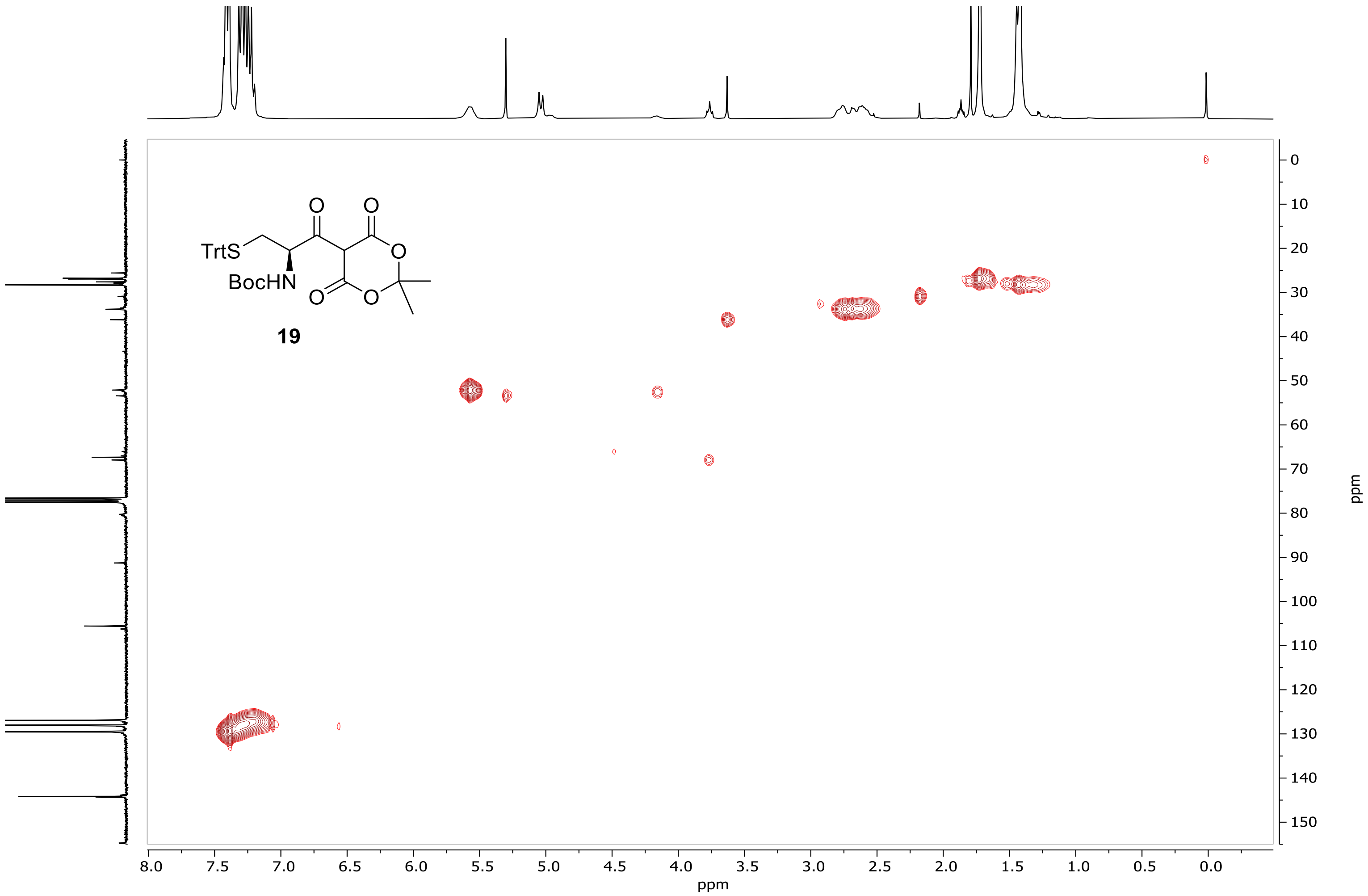

3.2 NMR and MS of 20

^{1}H NMR (400.13 MHz, $CDCl_3$) of **20**

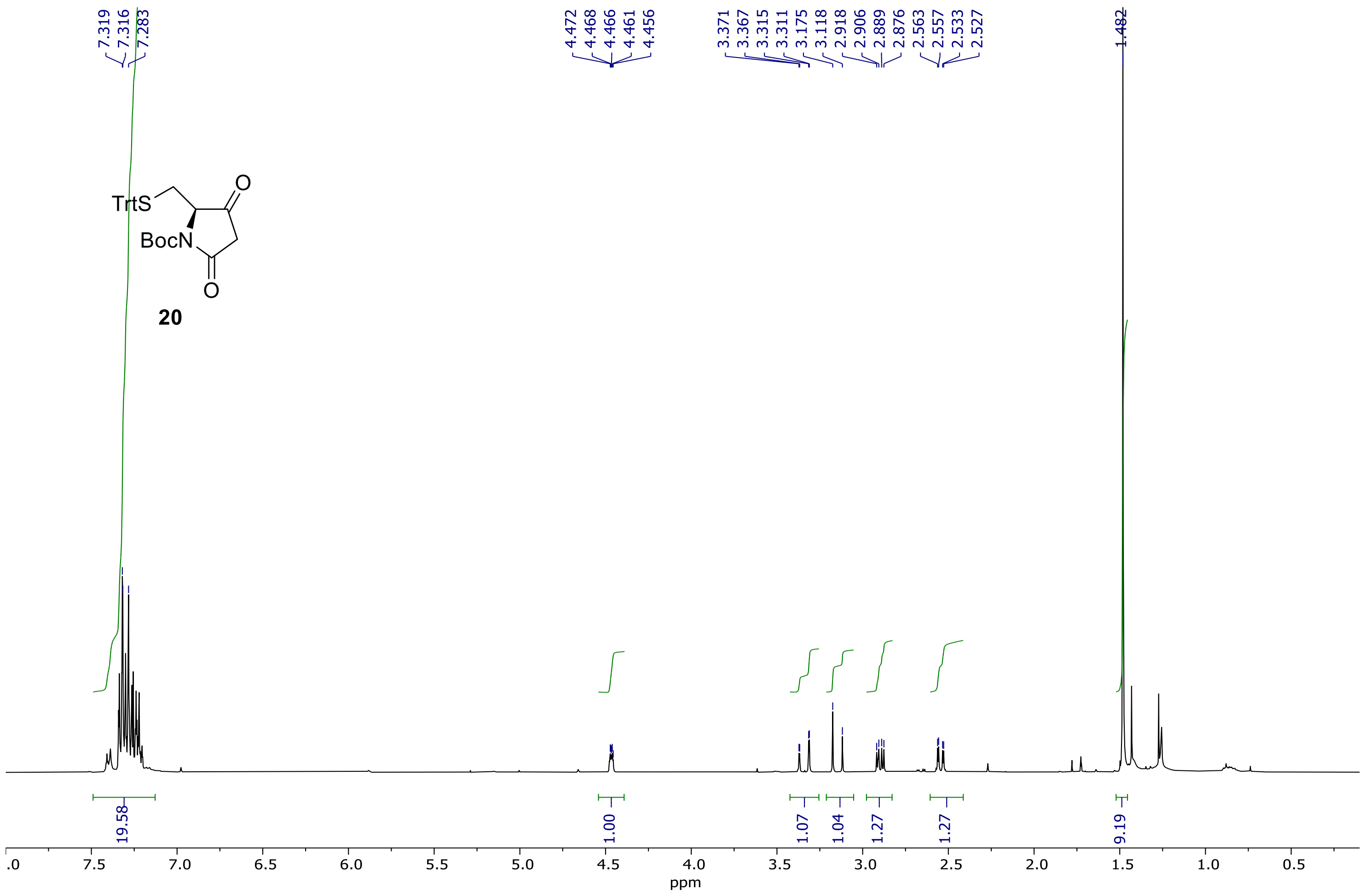

^{13}C NMR (100.13 MHz, $CDCl_3$) of **20**

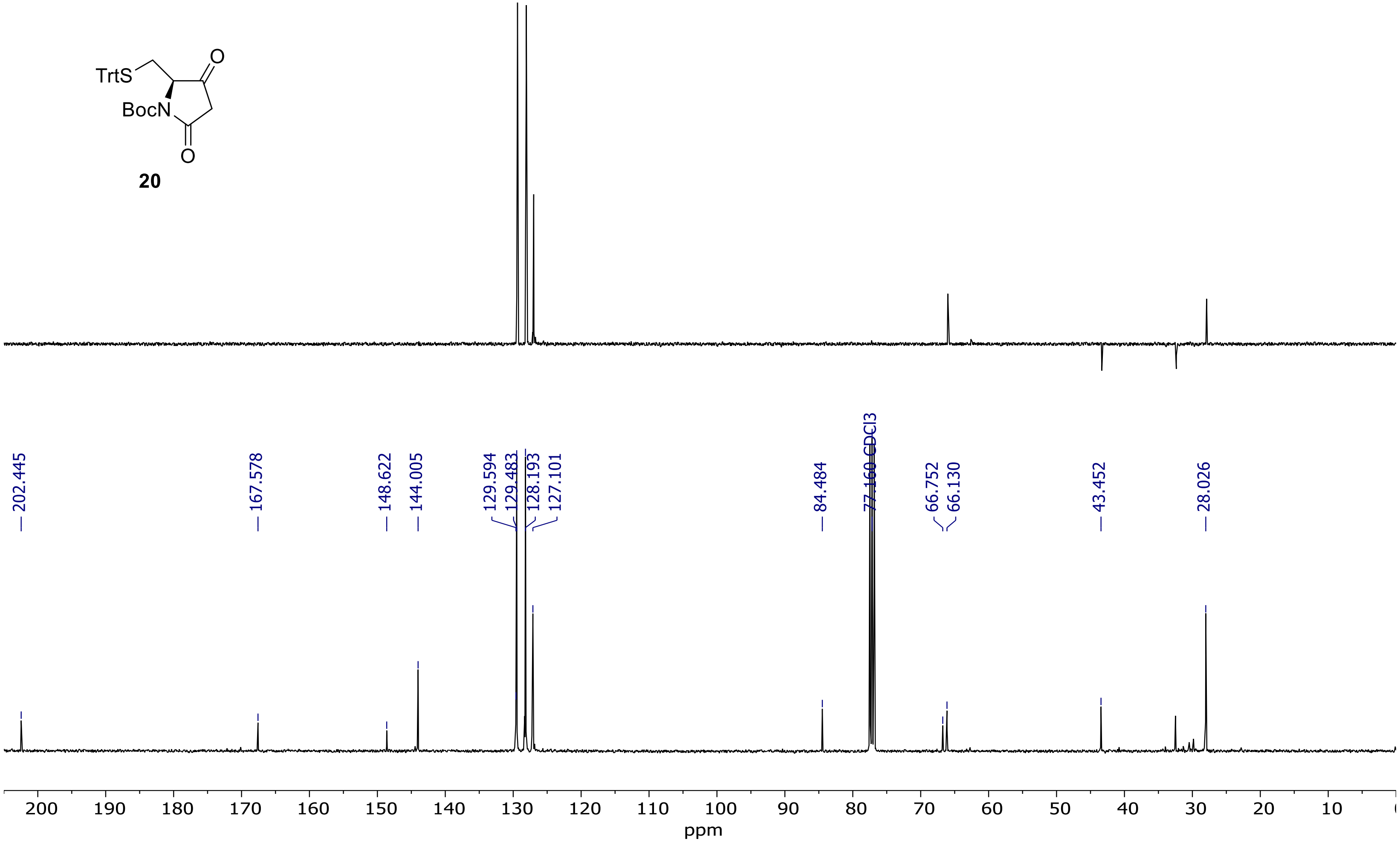

20

^{1}H-^{1}H COSY (100.13 MHz, $CDCl_3$) of **20**

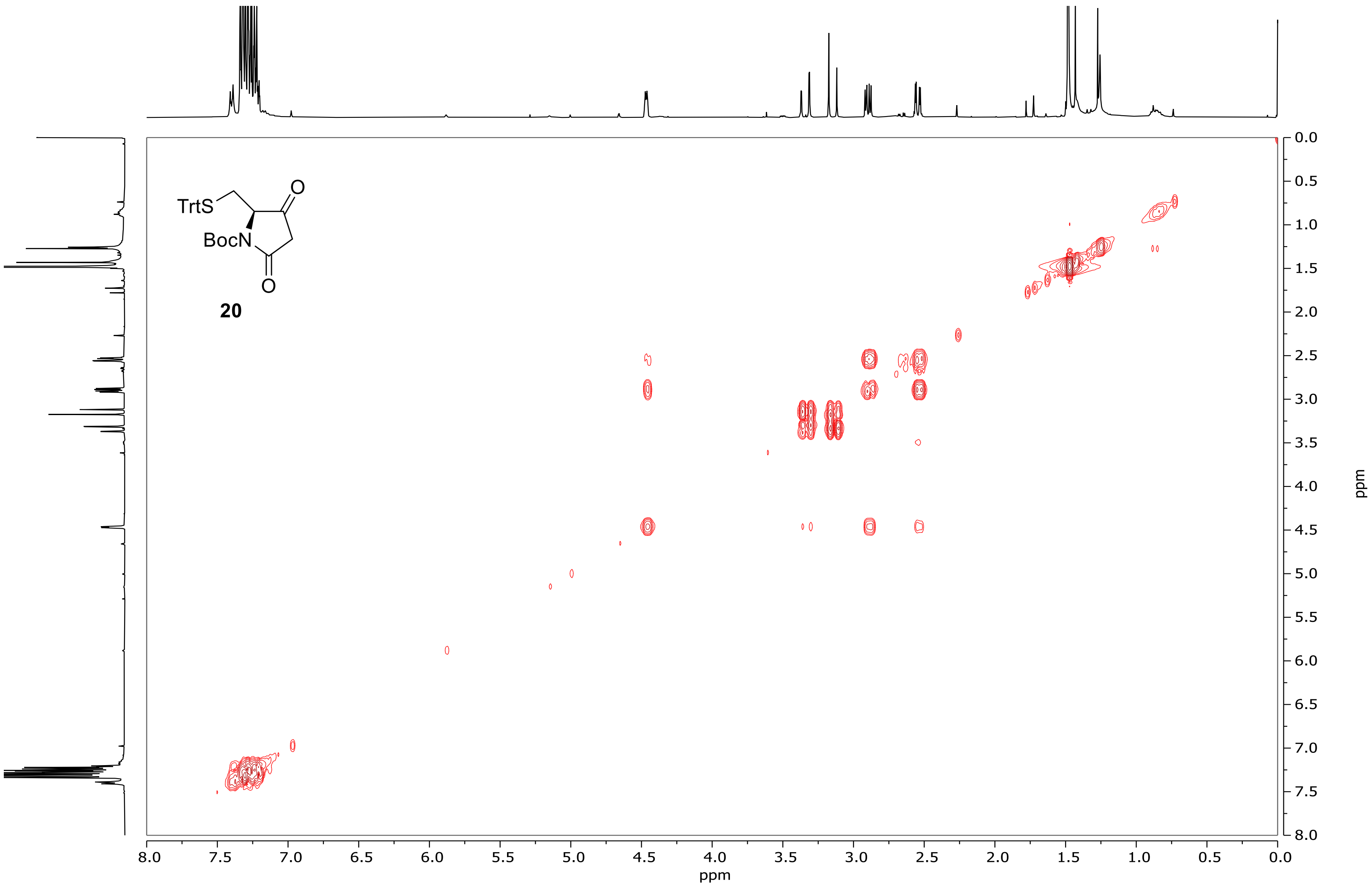

HSQC (400.13 MHz, $CDCl_3$) of **20**

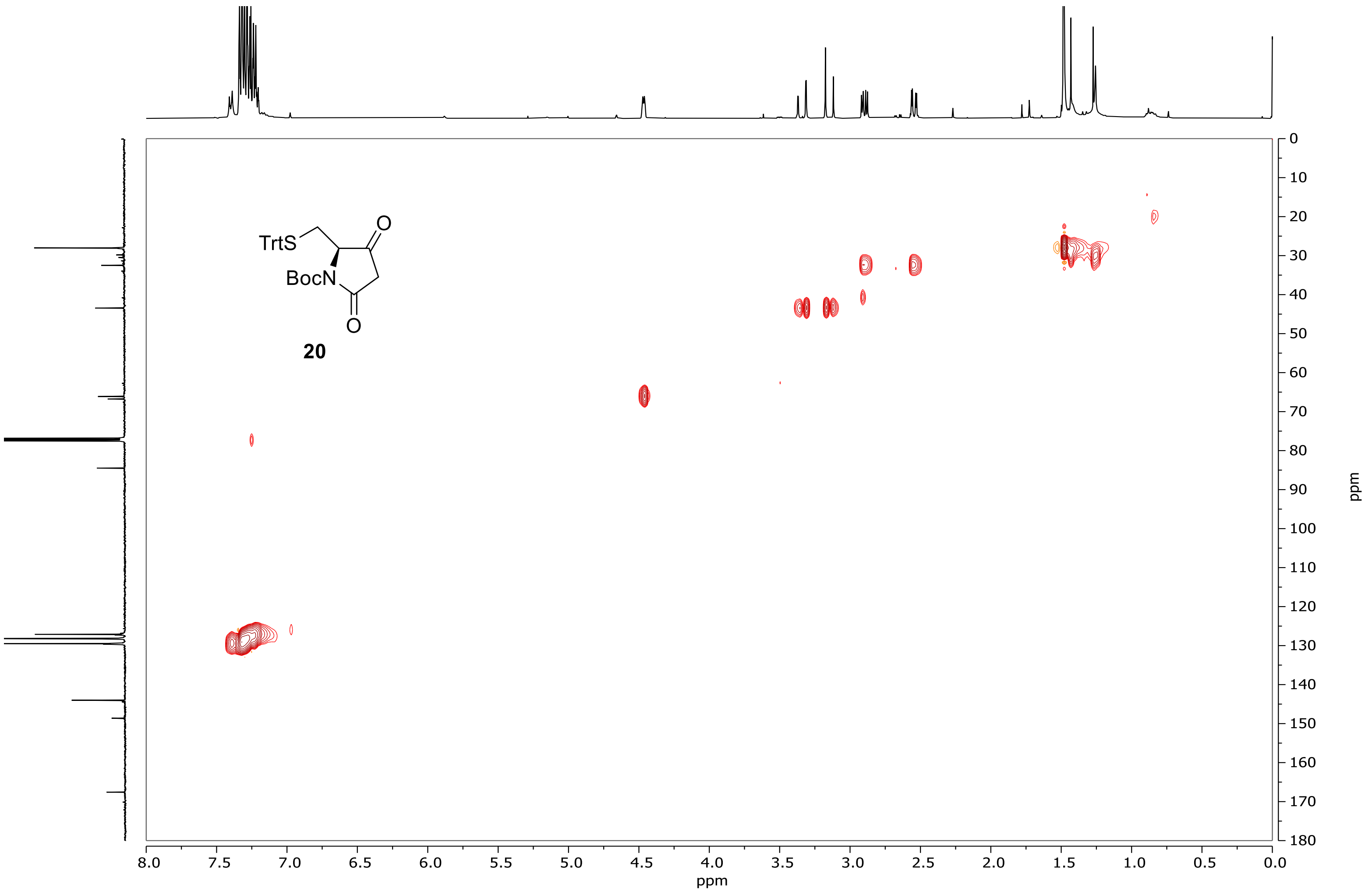

(+)-HRESIMS of **20**: Ion *m/z*: 510.1710 ([M+Na]$^+$)

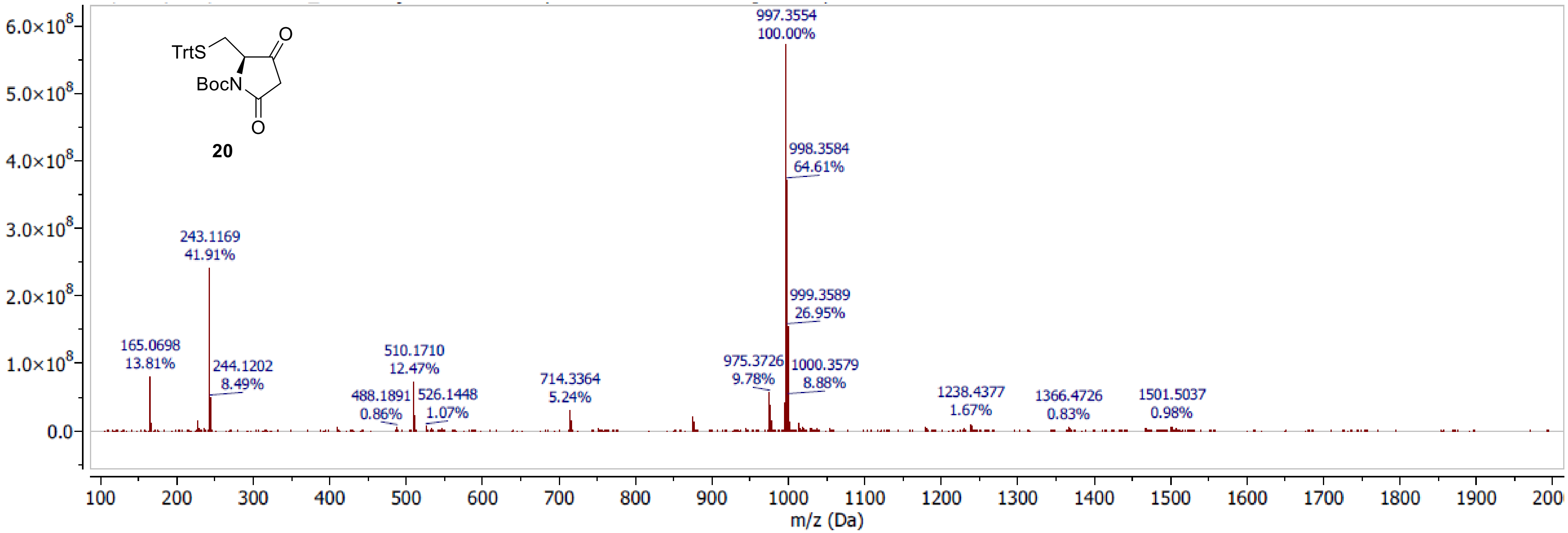

3.3 NMR and MS of 21

^{1}H NMR (300.13 MHz, $CDCl_3$) of **21**

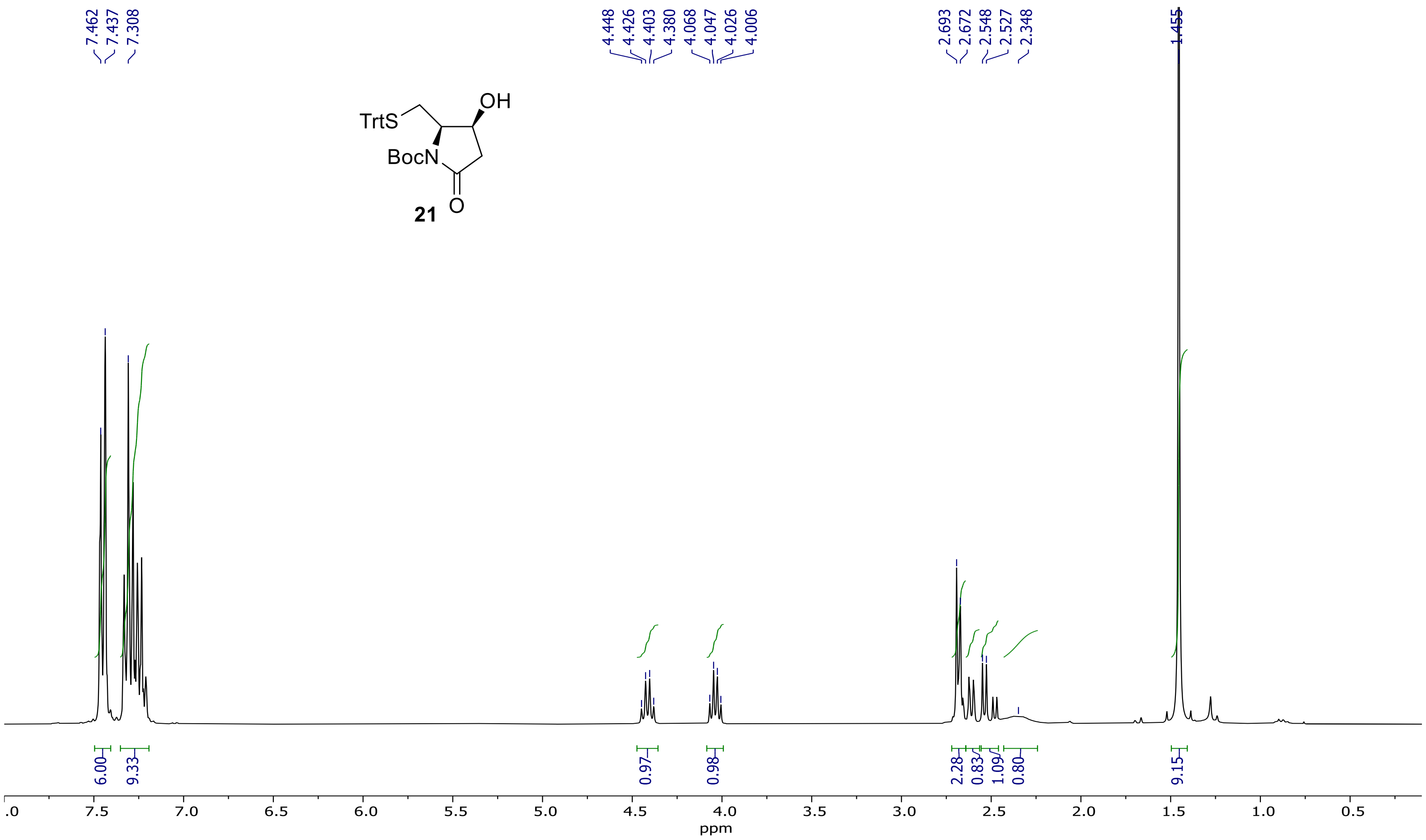

^{13}C NMR (75 MHz, $CDCl_3$) of **21**

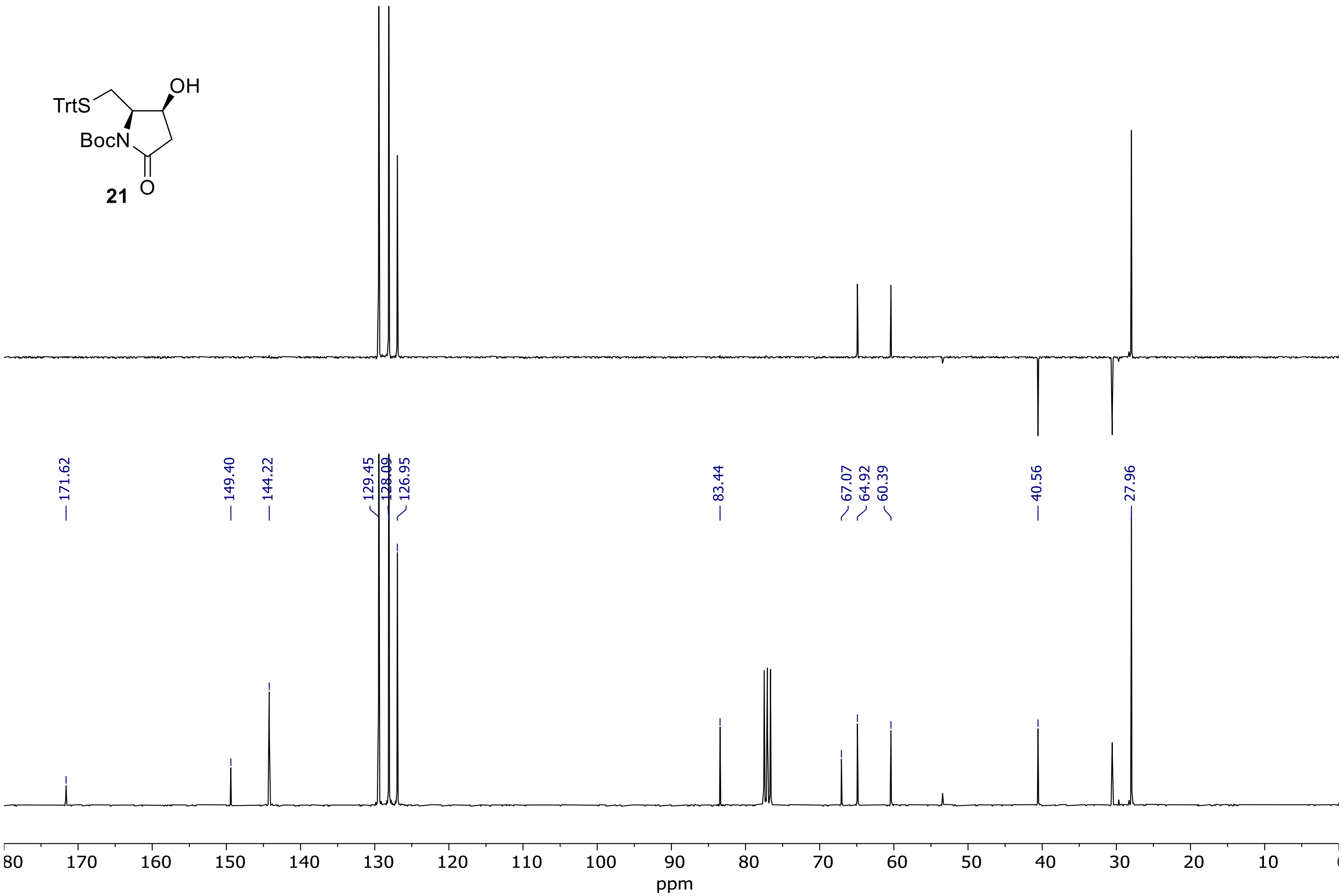

^{1}H-^{1}H COSY (300.13 MHz, $CDCl_3$) of **21**

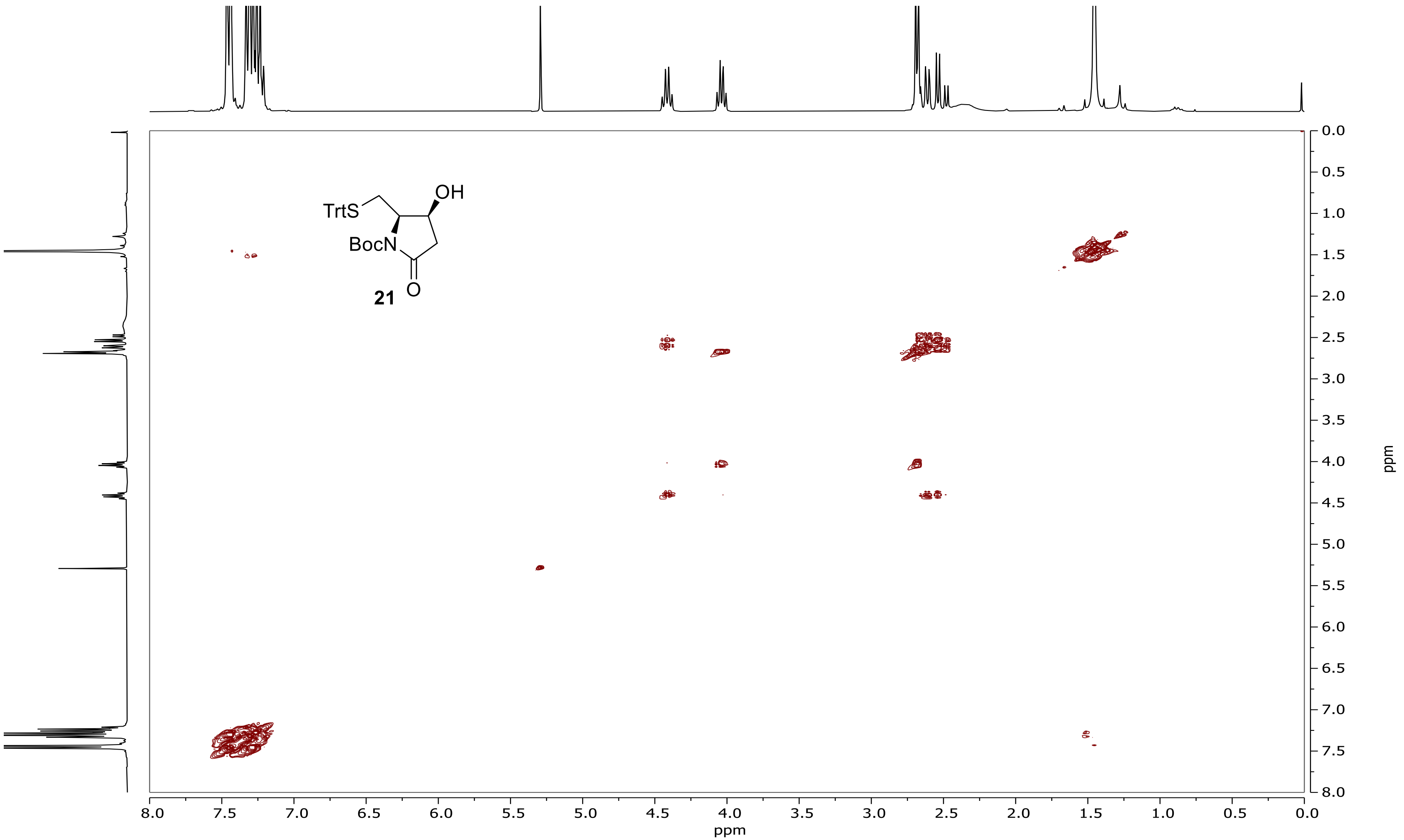

HSQC (300.13 MHz, $CDCl_3$) of **21**

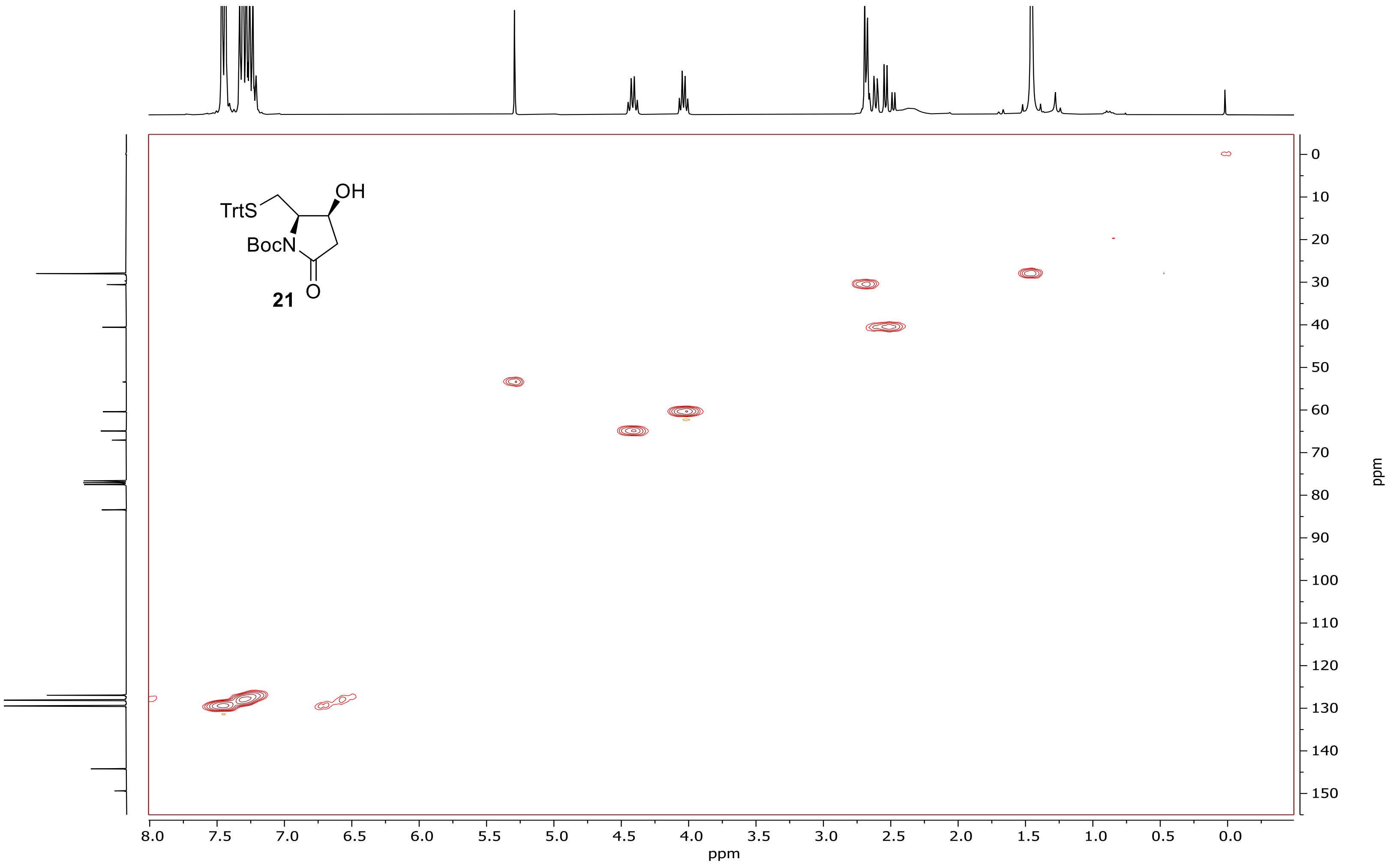

(+)-HRESIMS of **21**: Ion: *m/z*: 490.2035 $[M+H]^+$

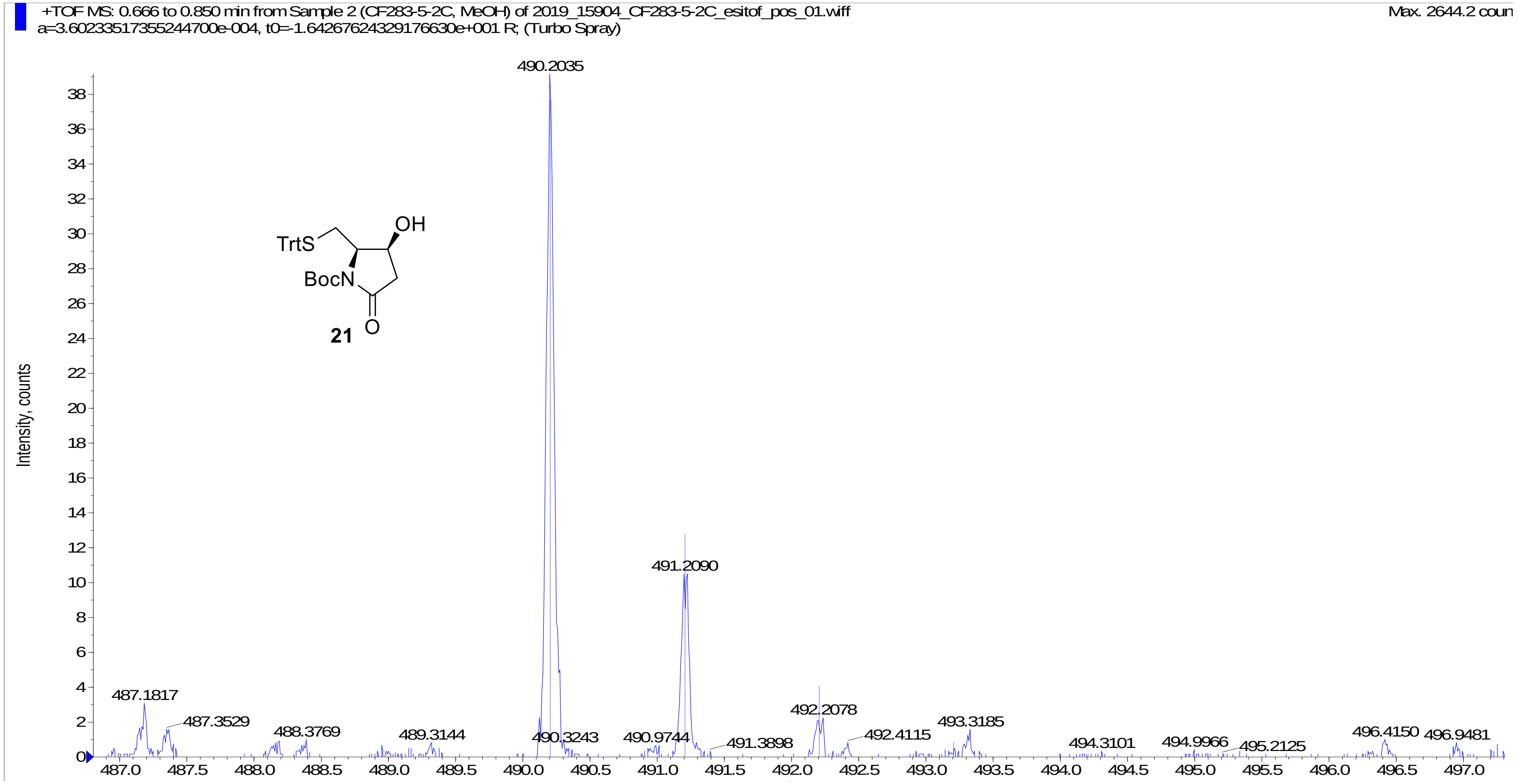

Formula	Calc m/z	Δ, mDa	Δ, ppm	DBE
$C_{29}H_{32}NO_4S$	490.2046	-1.157	-2.3603	14.5

3.4 NMR and MS of 22

^{1}H NMR (400.13 MHz, $CDCl_3$) of **22**

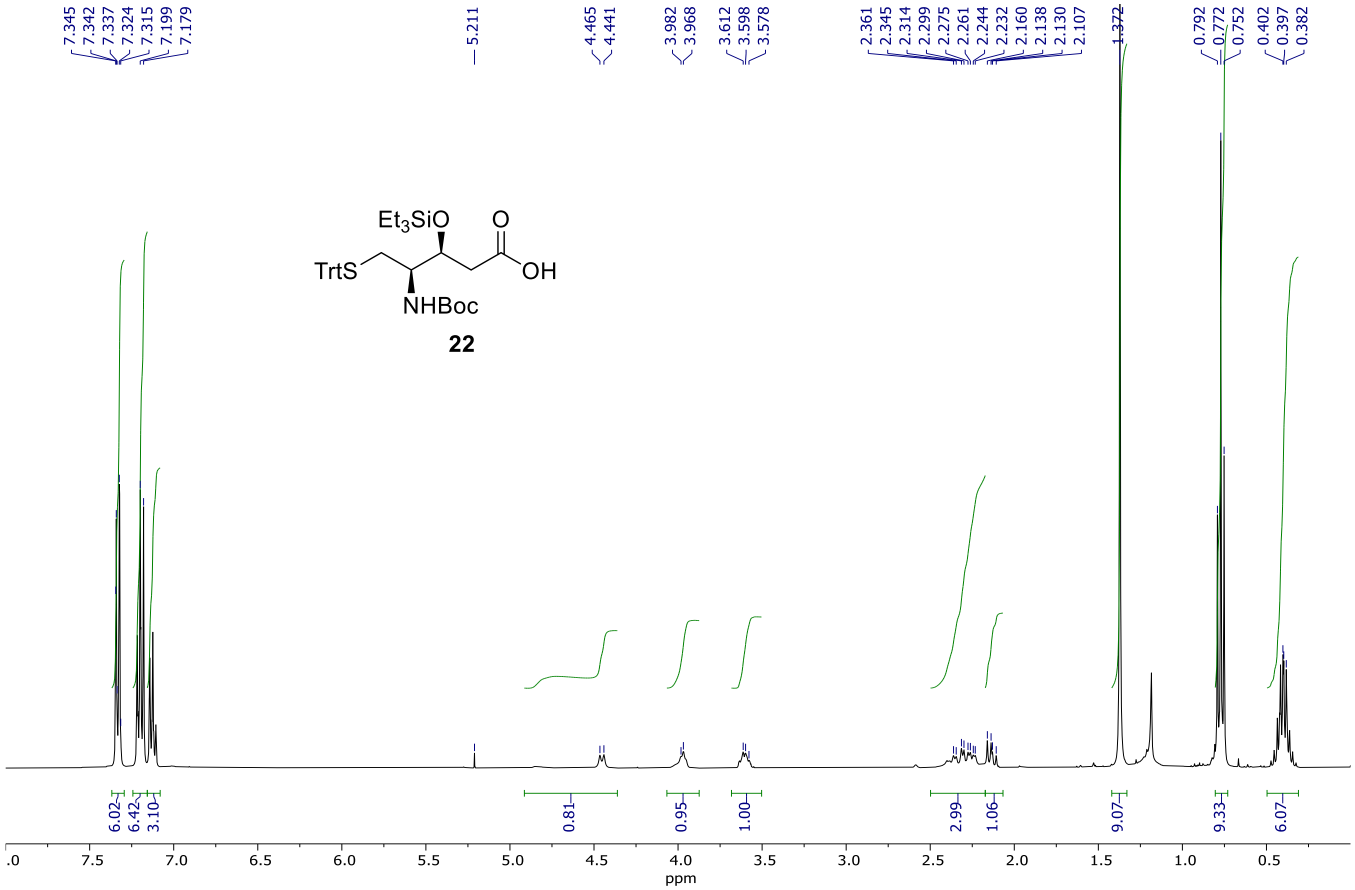

^{13}C NMR (100.13 MHz, $CDCl_3$) of **22**

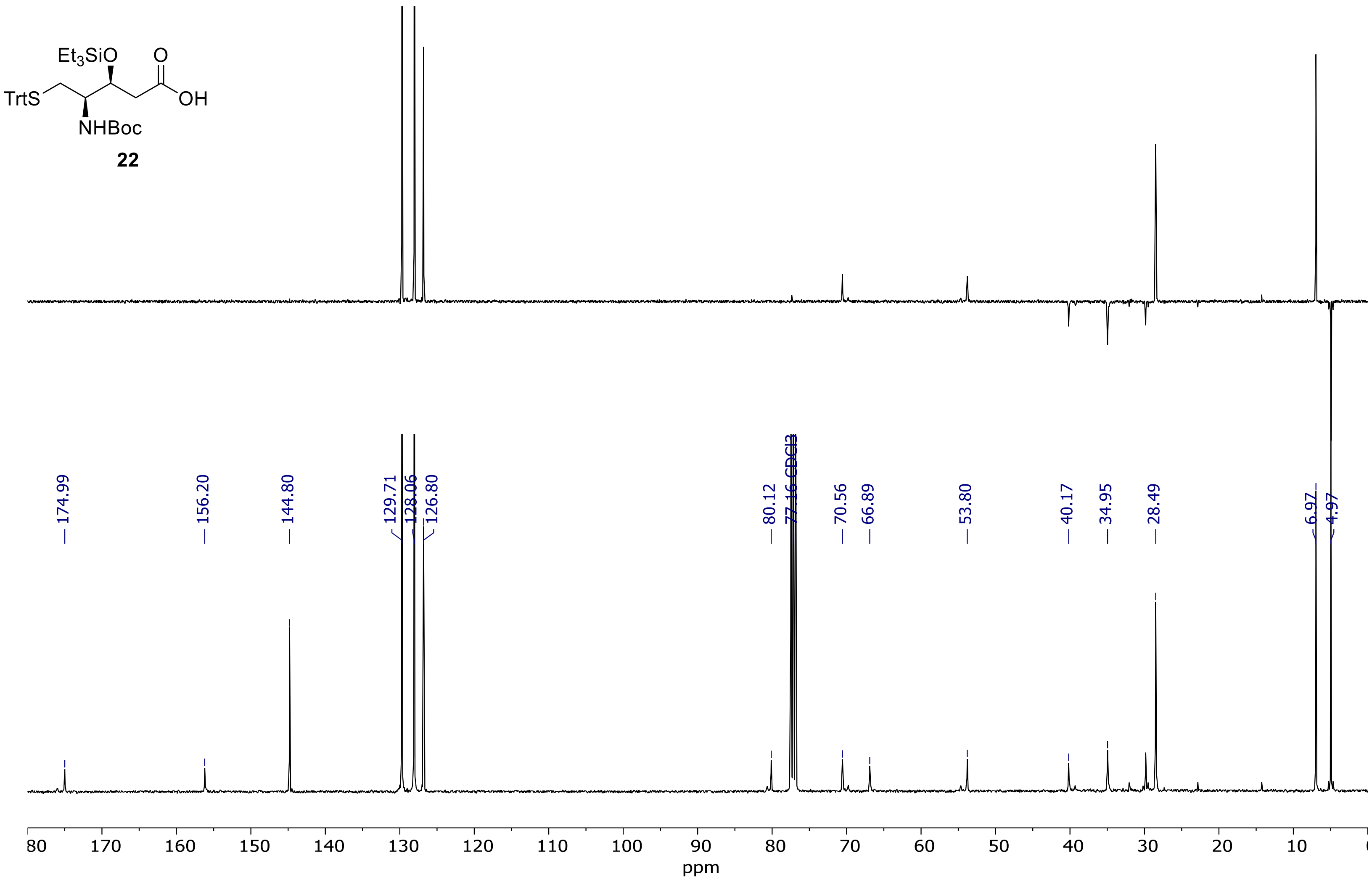

^{1}H-^{1}H COSY (400.13 MHz, $CDCl_3$) of **22**

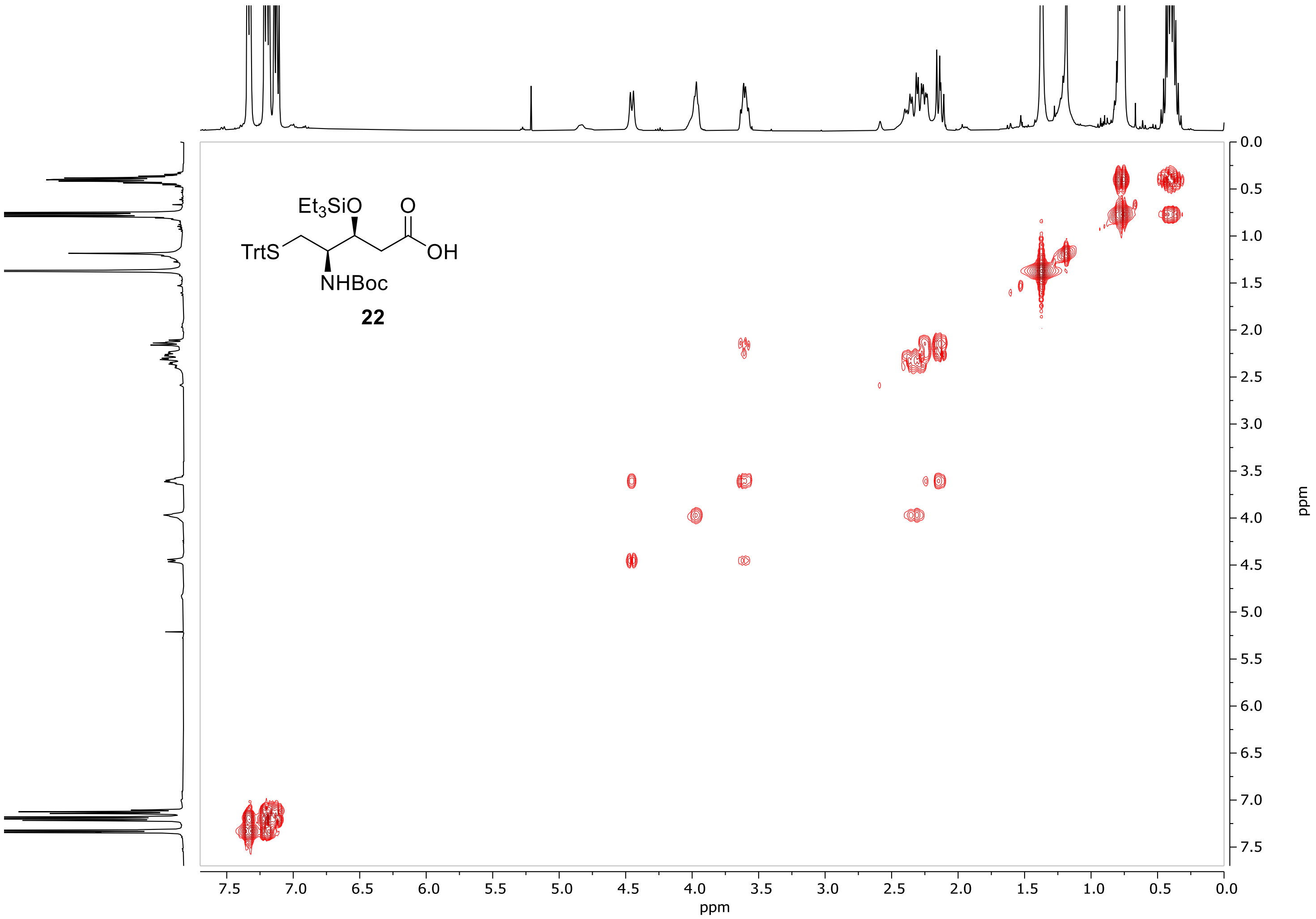

HSQC (400.13 MHz, $CDCl_3$) of **22**

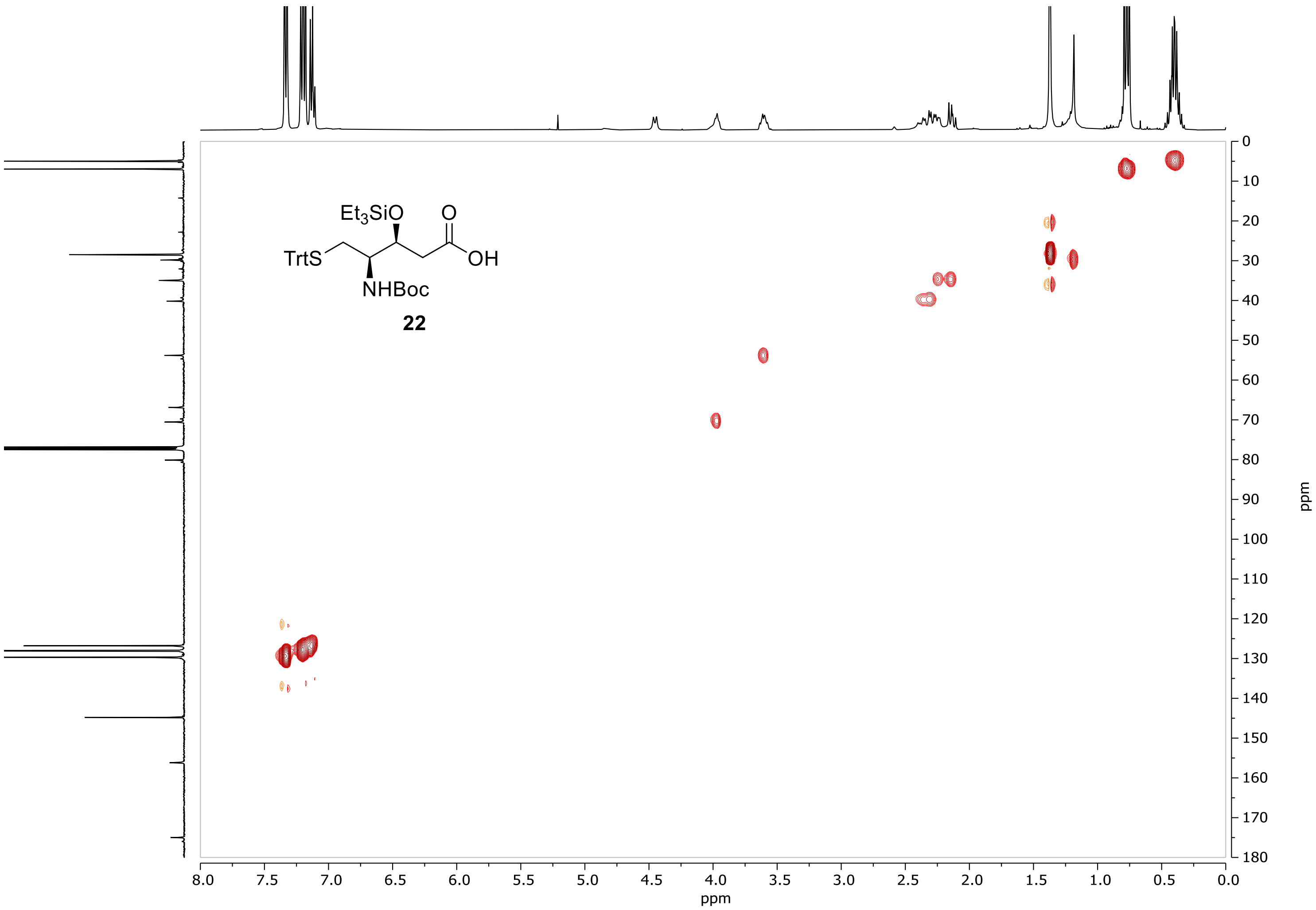

(+)-HRESIMS of **22**: Ion: m/z: 644.2831 $[M+Na]^+$

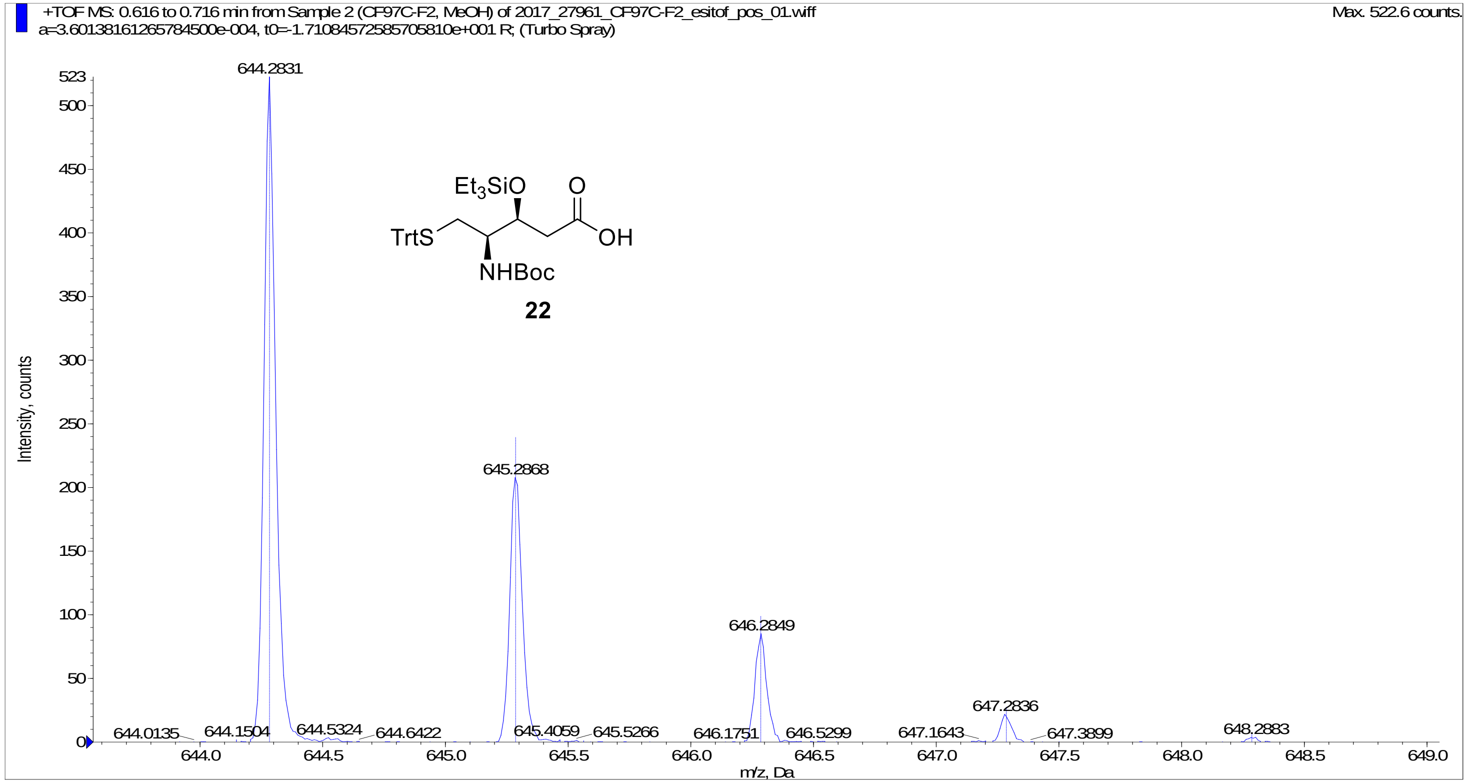

Formula	Calc m/z	Δ, mDa	Δ, ppm	DBE
$C_{35}H_{47}NO_5NaSiS$	644.2836	-0.5454	-0.8465	13.5

3.5 ^{1}H NMR (300 MHz, $CDCl_3$) of 24

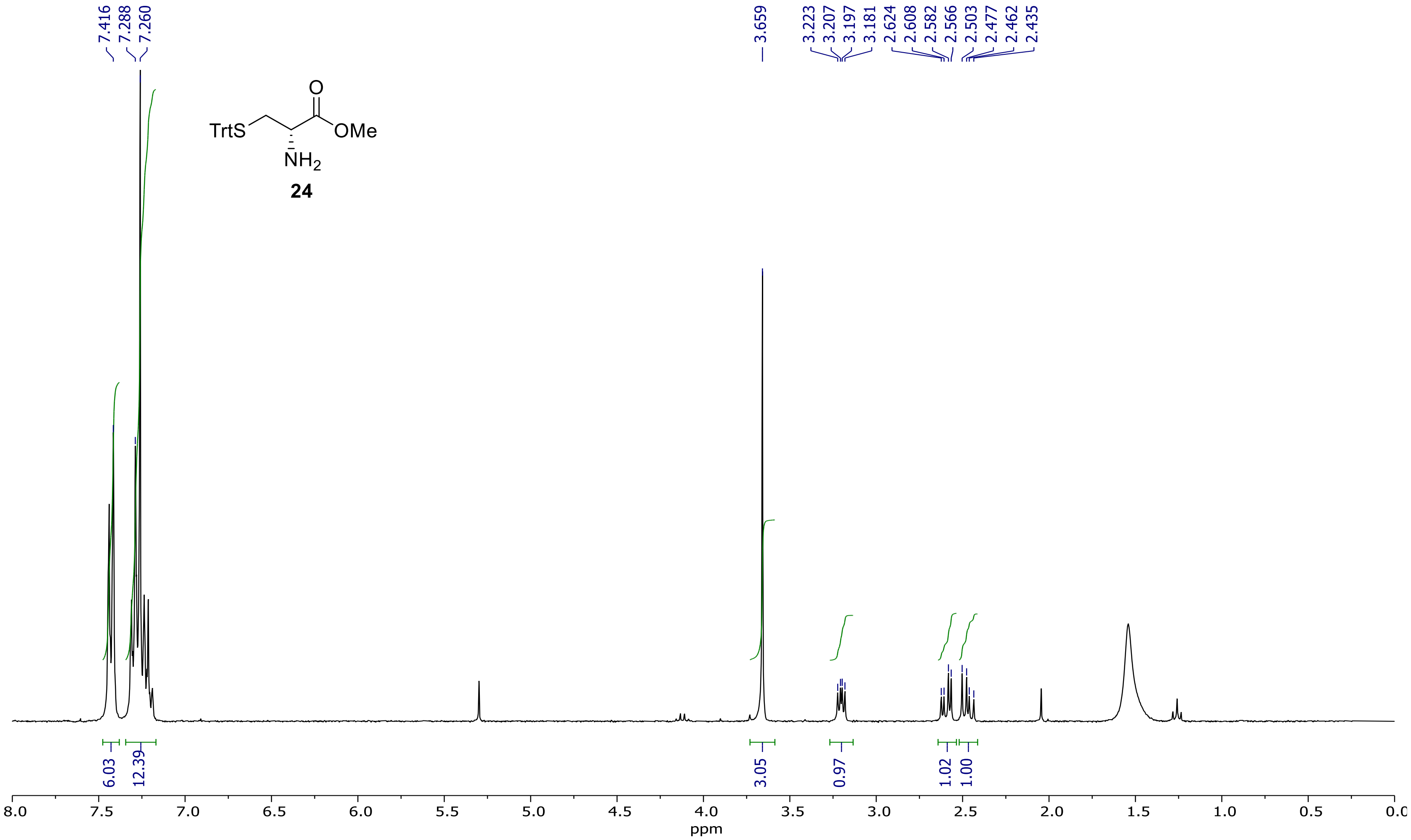

3.6 NMR and MS of compound 25

^1H NMR (300 MHz, $CDCl_3$) of **25**

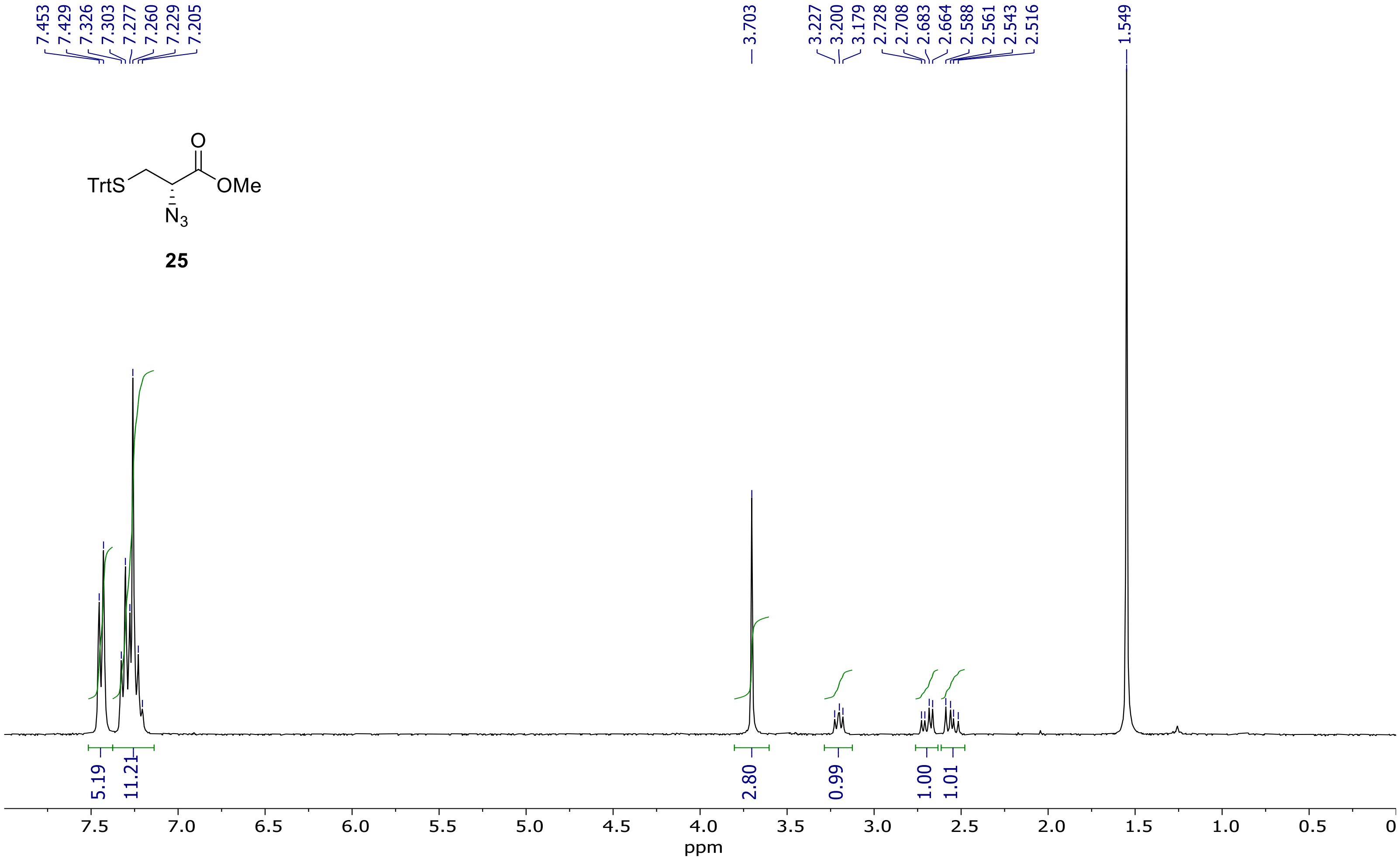

^{13}C NMR (75 MHz, $CDCl_3$) of **25**

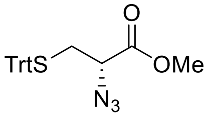

25

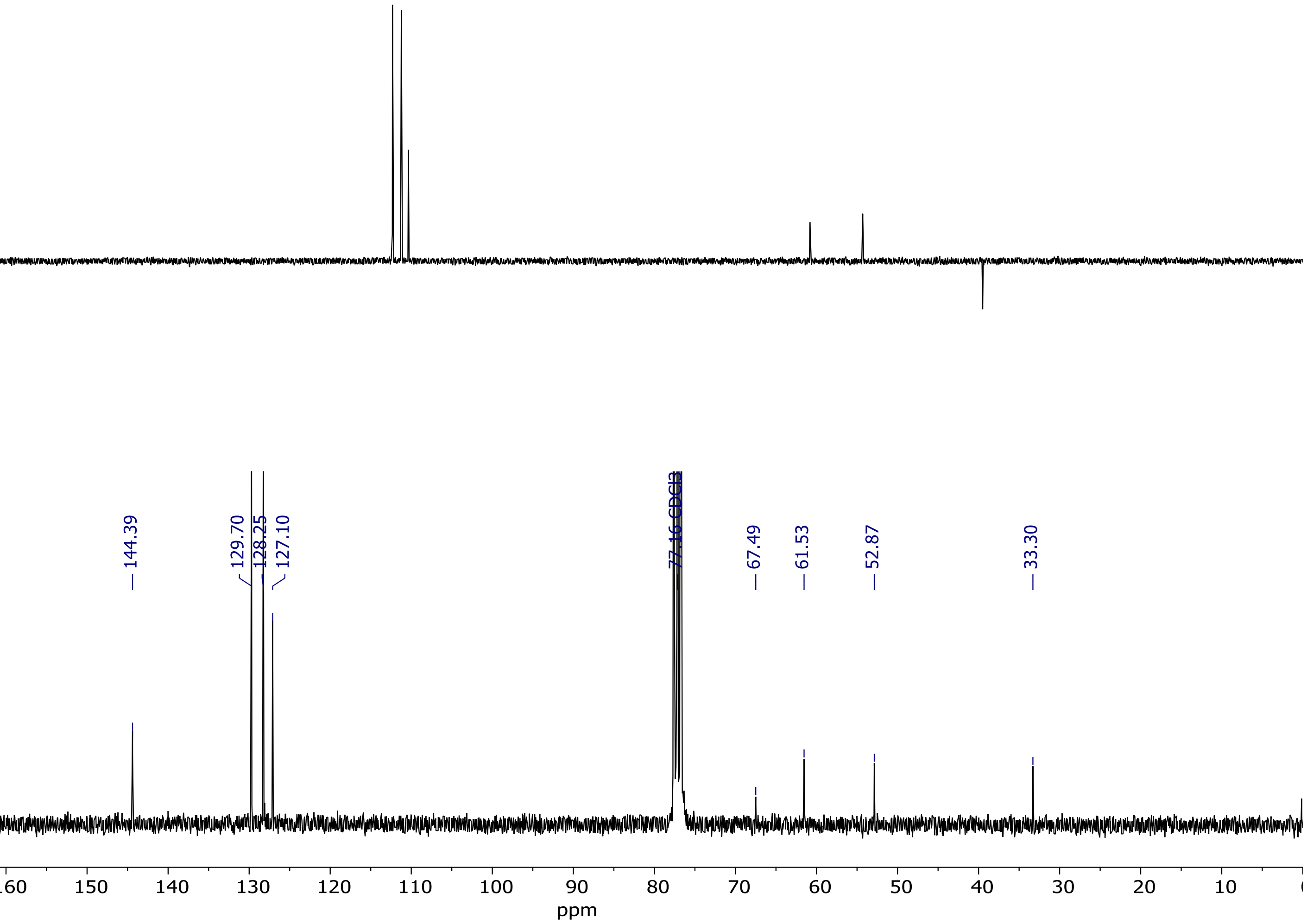

(+)-HRESIMS of **25**: Ion: *m/z*: 426.1242 ($[M+Na]^+$)

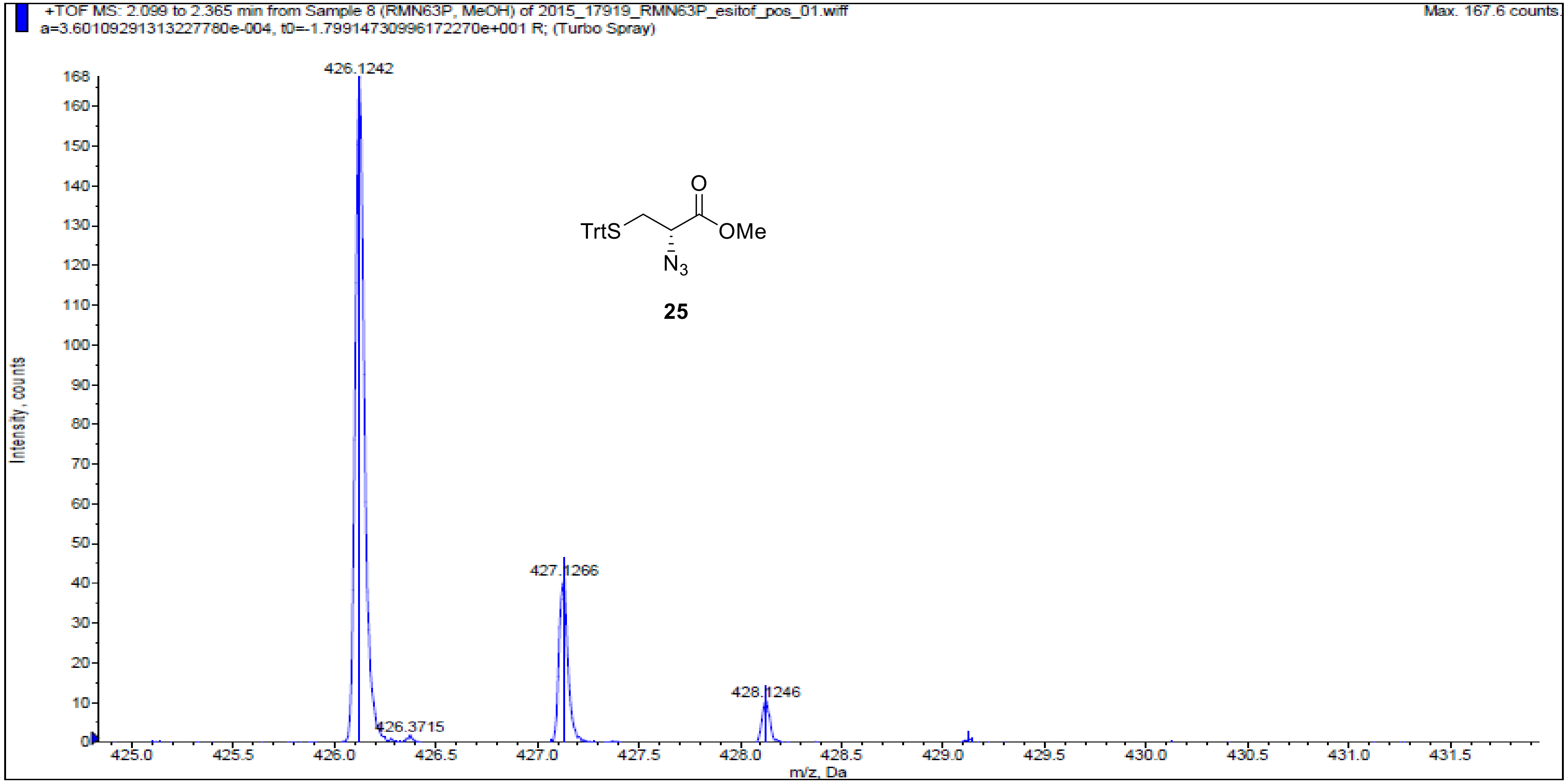

Formula	Calc m/z	Δ, mDa	Δ, ppm	DBE
$C_{23}H_{21}N_3NaO_2S$	426.1247	0.5	1.17	15.5

3.7 NMR and MS of 27

^{1}H NMR (300 MHz, $CDCl_3$) of **27**

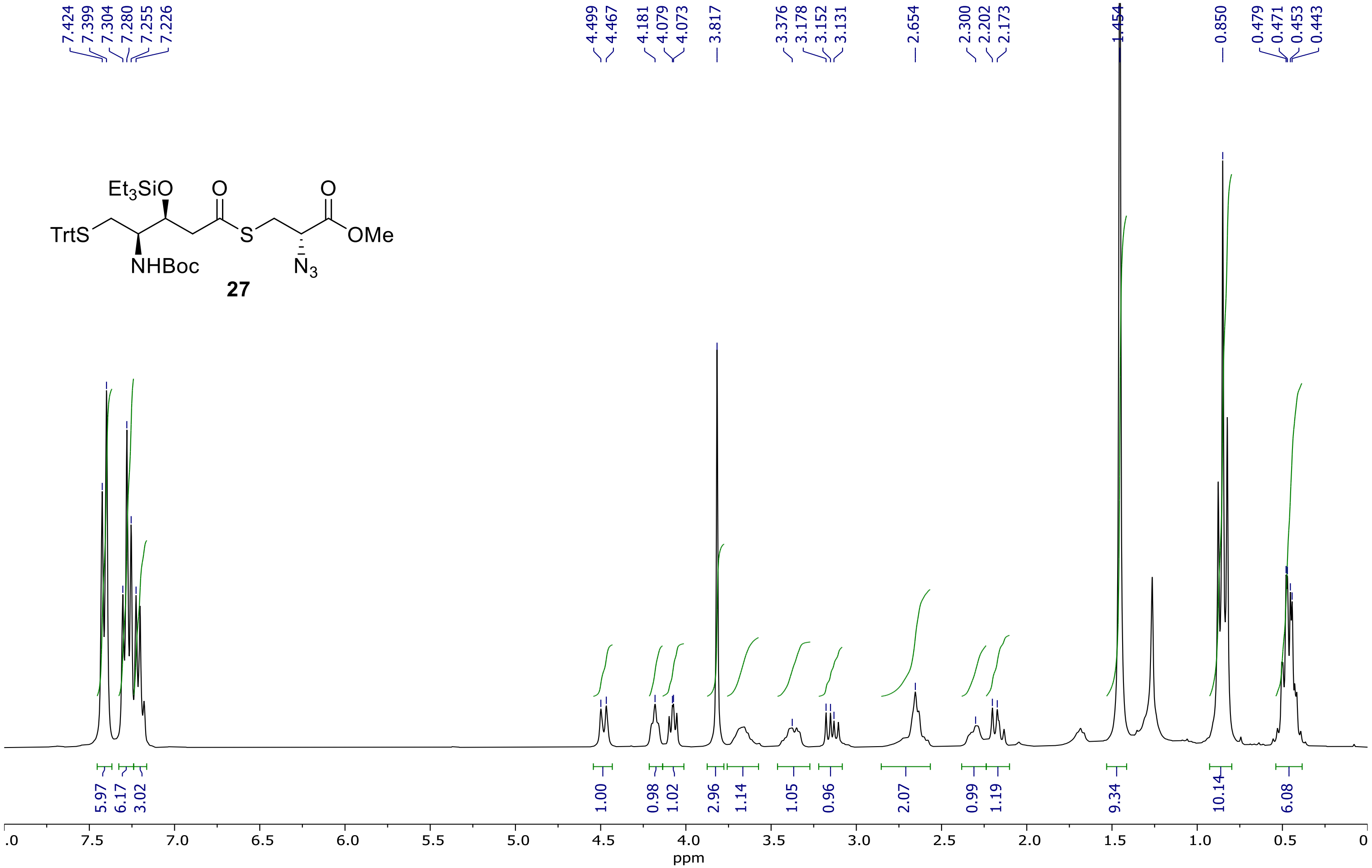

^{13}C NMR (75 MHz, $CDCl_3$) of **27**

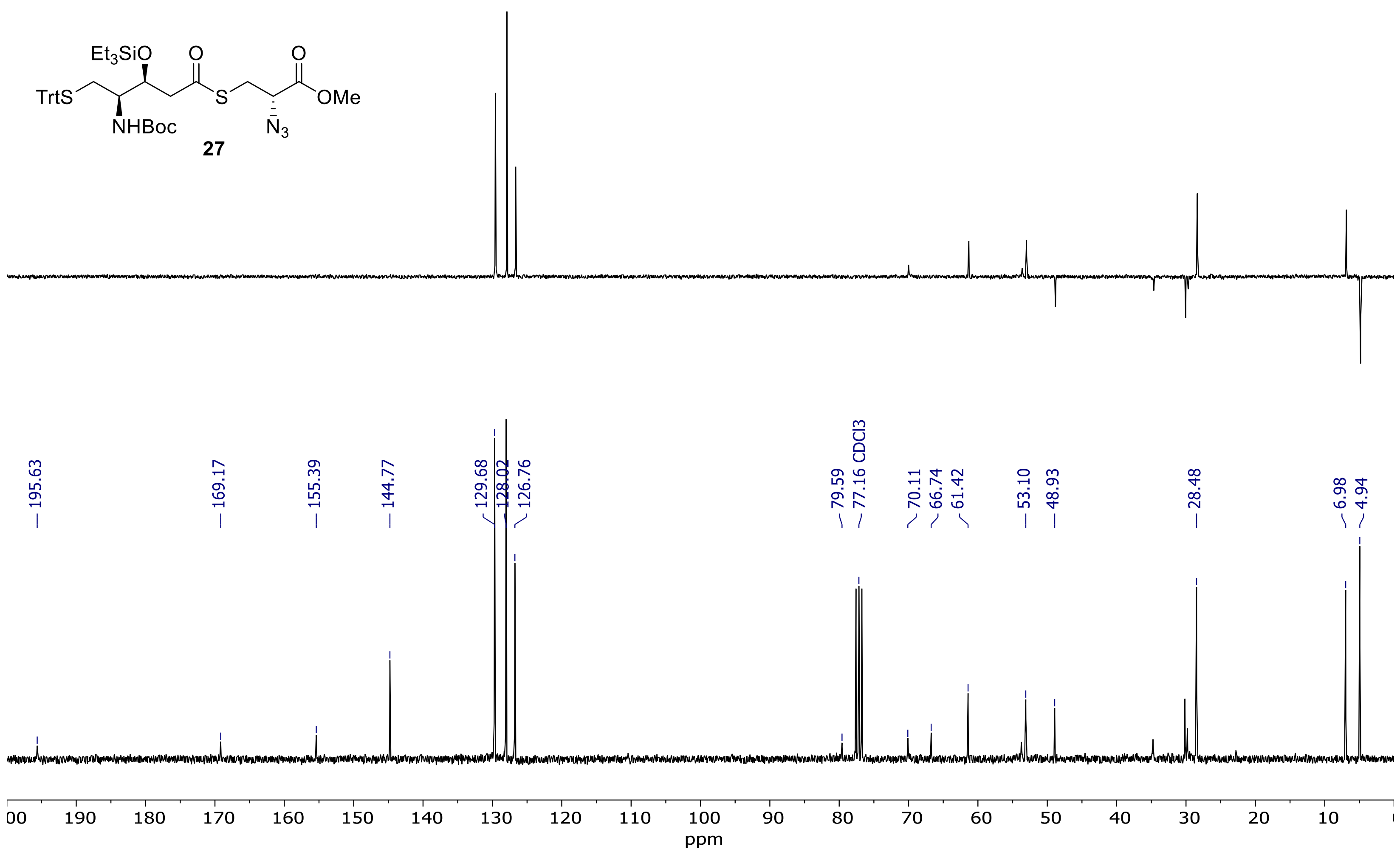

^{1}H-^{1}H COSY (300.13 MHz, $CDCl_3$) of **27**

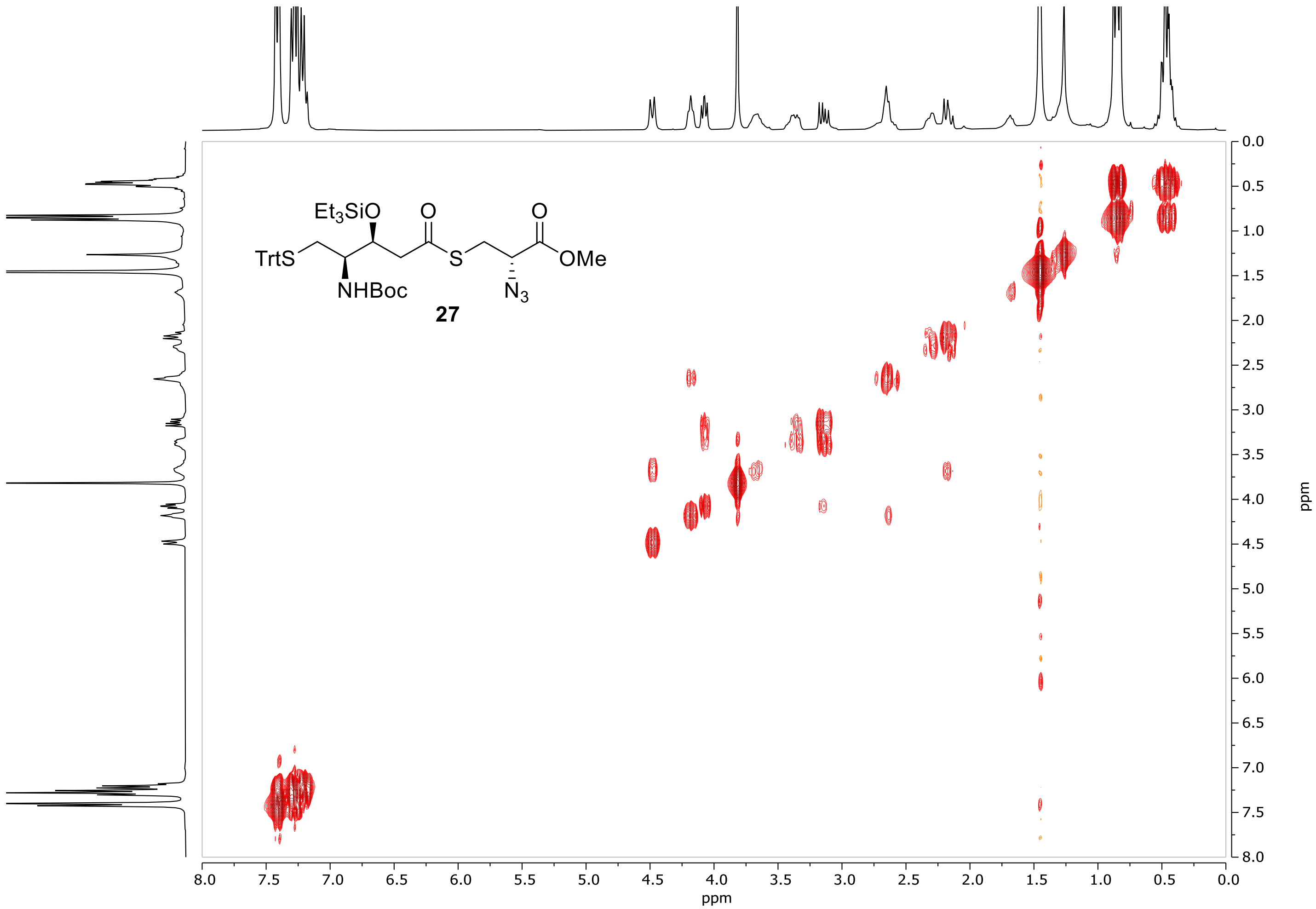

HSQC (300.13 MHz, $CDCl_3$) of **27**

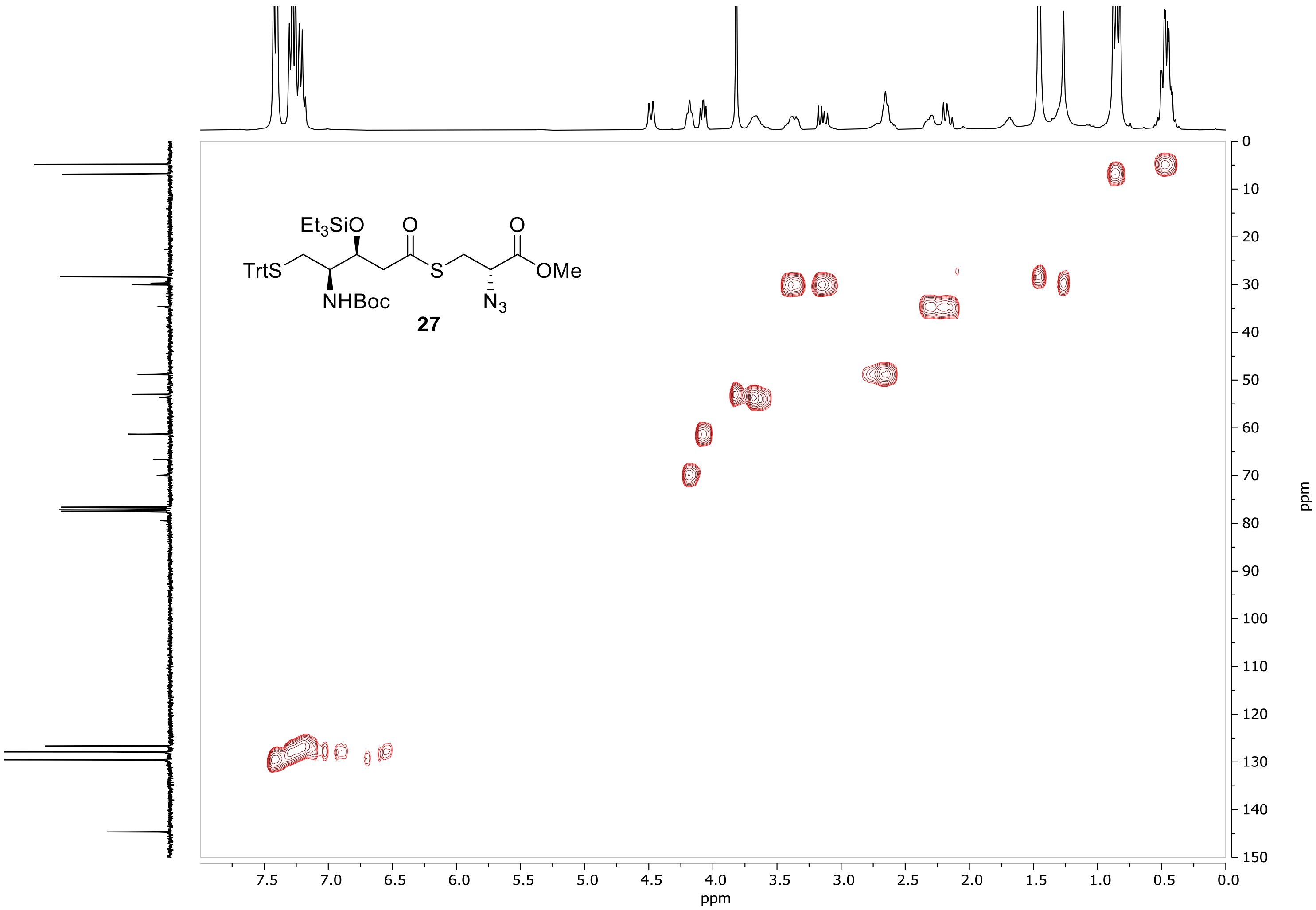

(+)-HRESIMS of **27**: Ion: *m/z*: 765.3179 ($[M+H]^+$)

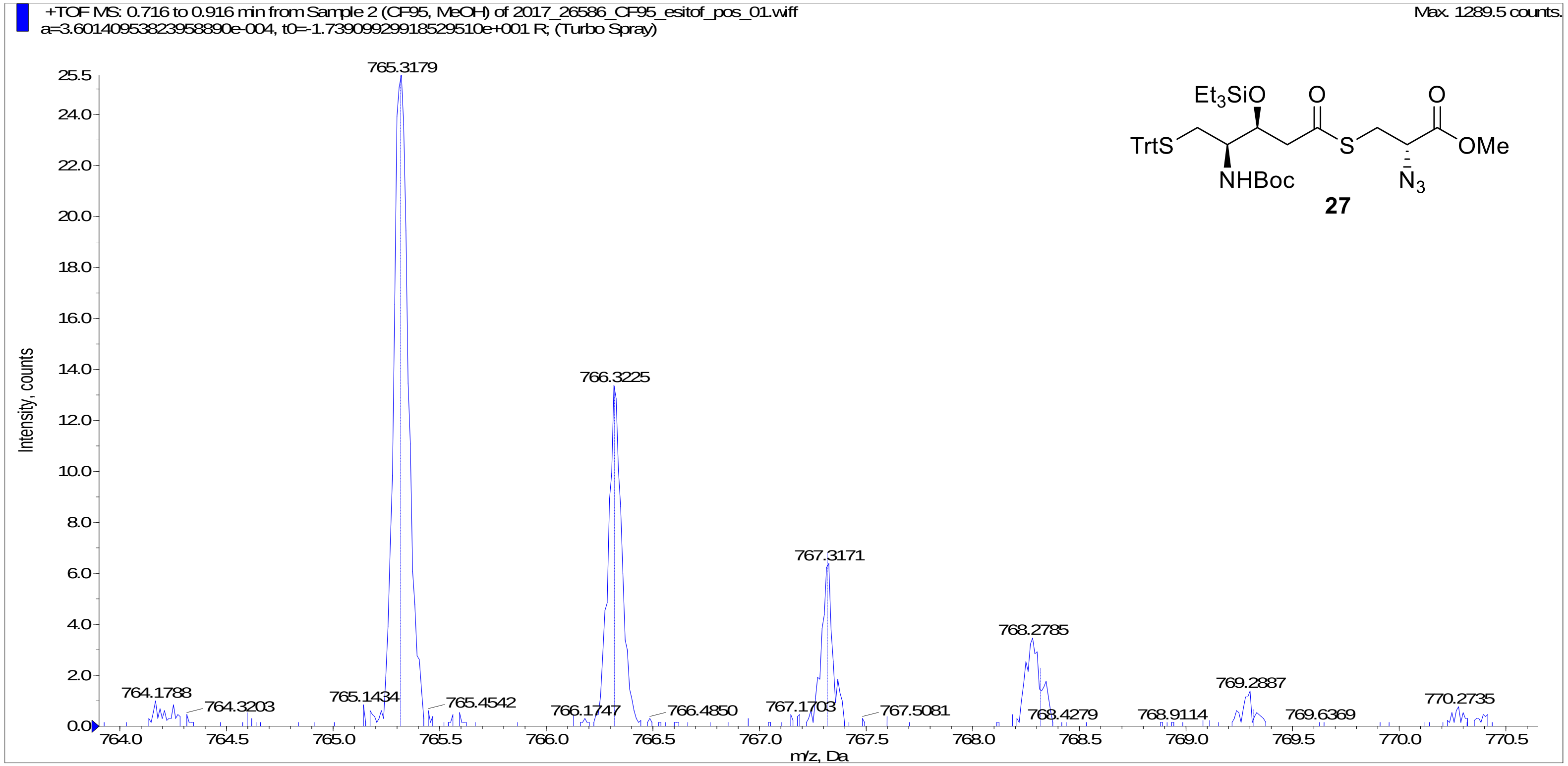

Formula	Calc m/z	Δ, mDa	Δ, ppm	DBE
$C_{39}H_{53}N_4O_6SiS_2$	765.3170	0.8655	1.131	16.5

3.8 NMR and MS of 28

^{1}H NMR (400 MHz, $CDCl_3$) of **28**

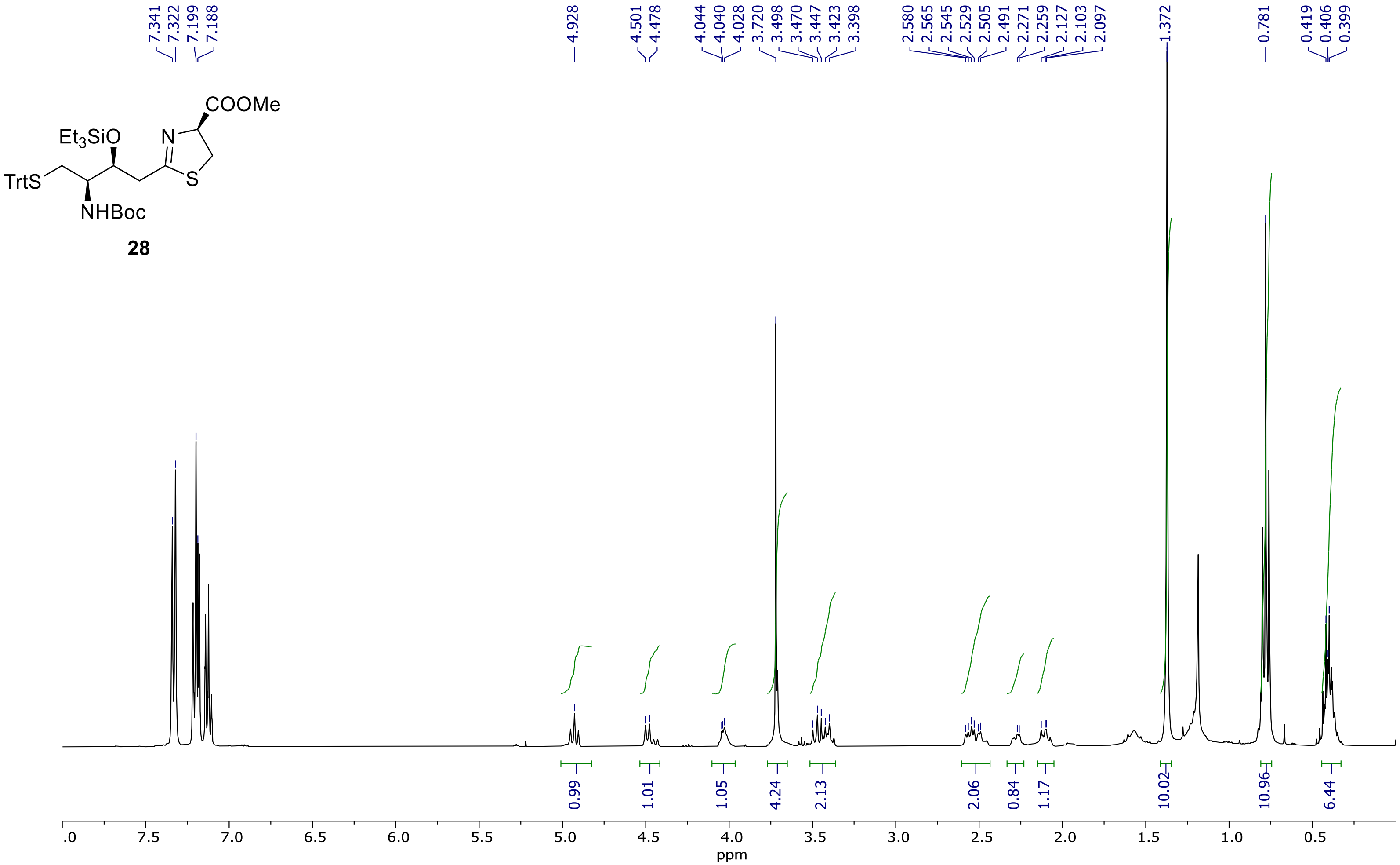

^{13}C NMR (100.13 MHz, $CDCl_3$) of **28**

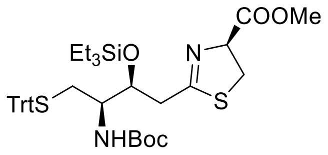

28

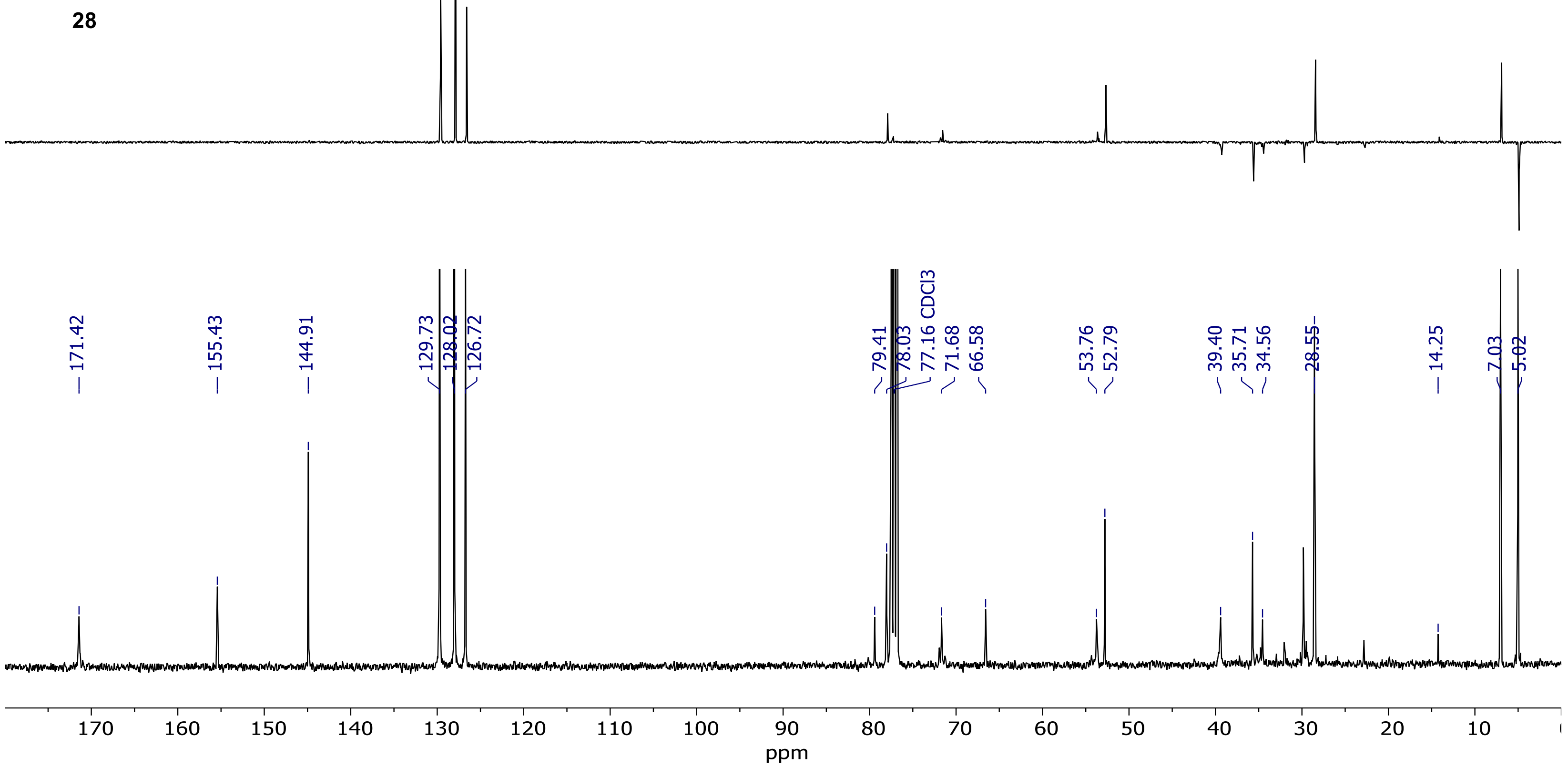

^{1}H-^{1}H COSY (100.13 MHz, $CDCl_3$) of **28**

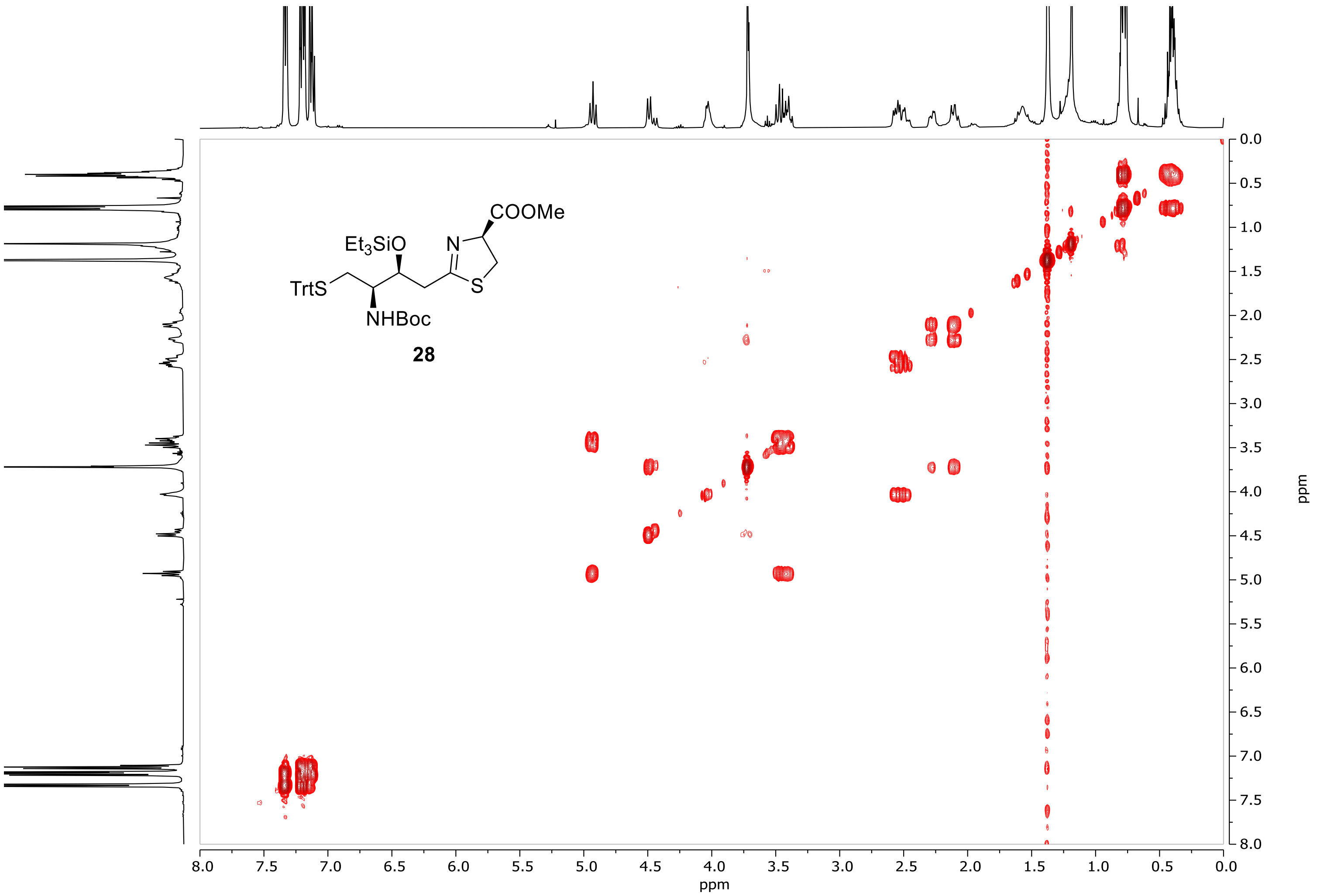

HSQC (100.13 MHz, $CDCl_3$) of **28**

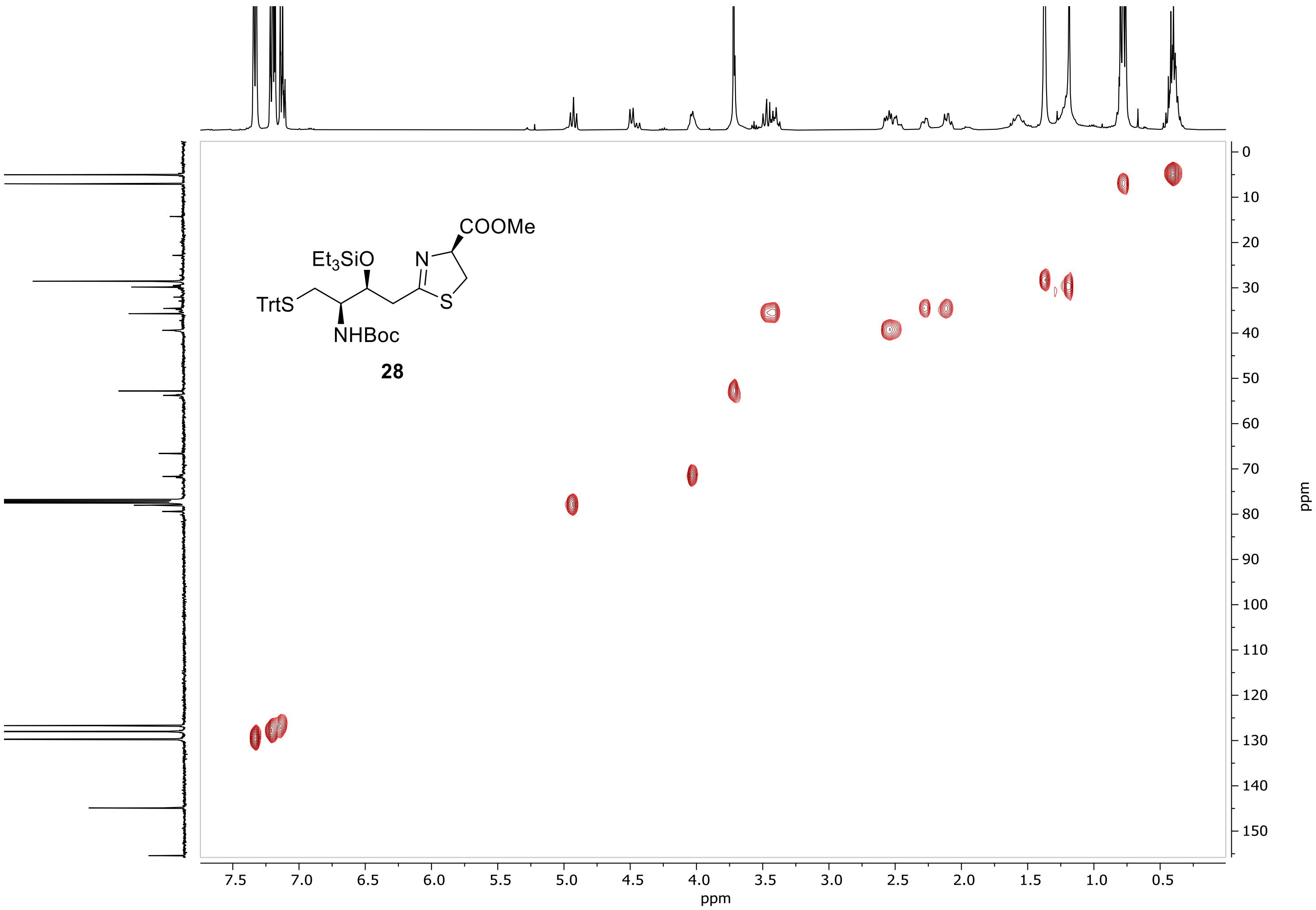

(+)-HRESIMS of **28**: Ion: *m/z*: 721.3173 ([M+H]$^{+}$)

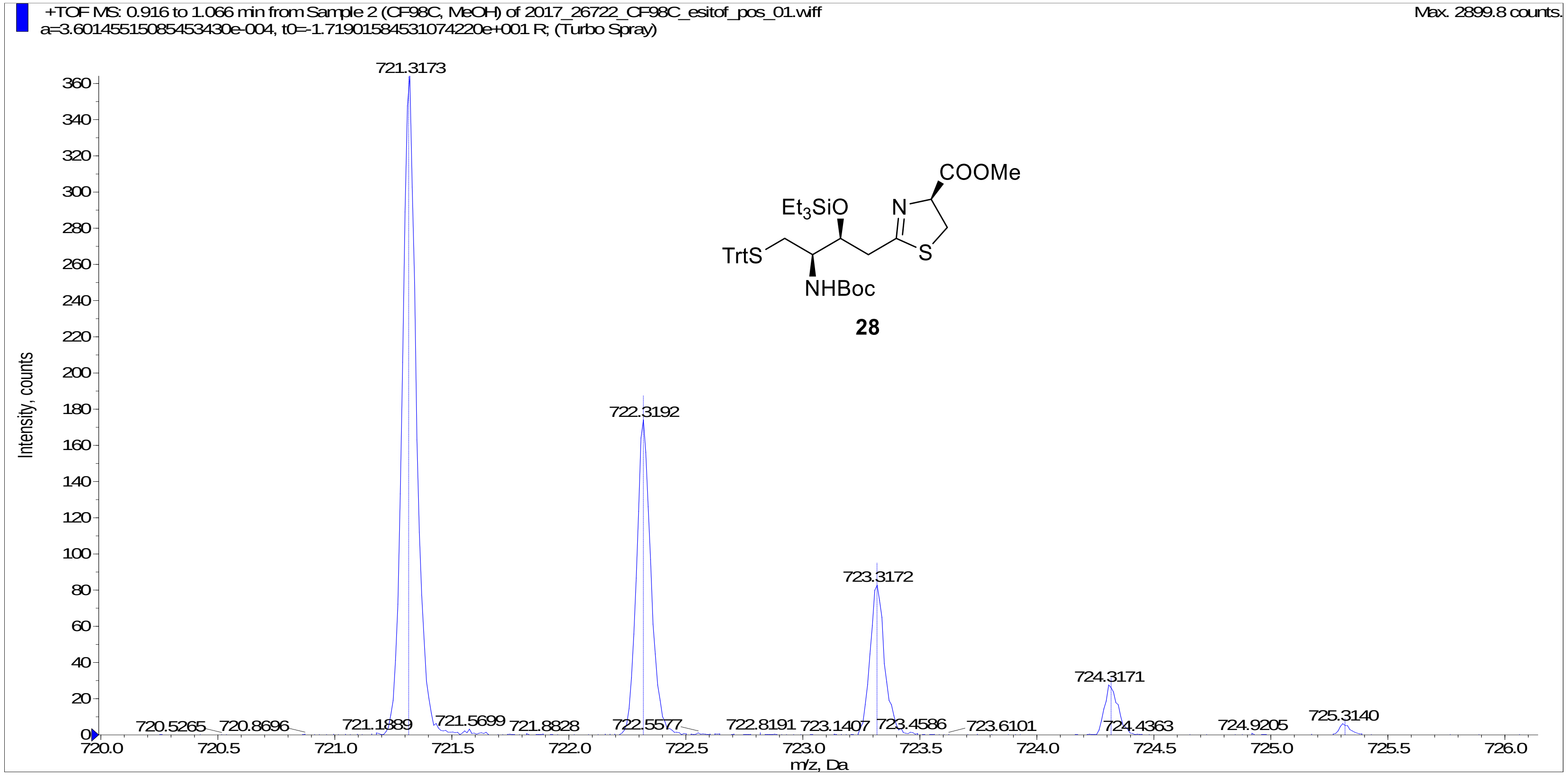

Formula	Calc m/z	Δ, mDa	Δ, ppm	DBE
$C_{39}H_{53}N_2O_5SiS_2$	721.3159	1.3282	1.8414	15.5

3.9 NMR and MS of 29

^{1}H NMR (400 MHz, $CDCl_3$) of **29**

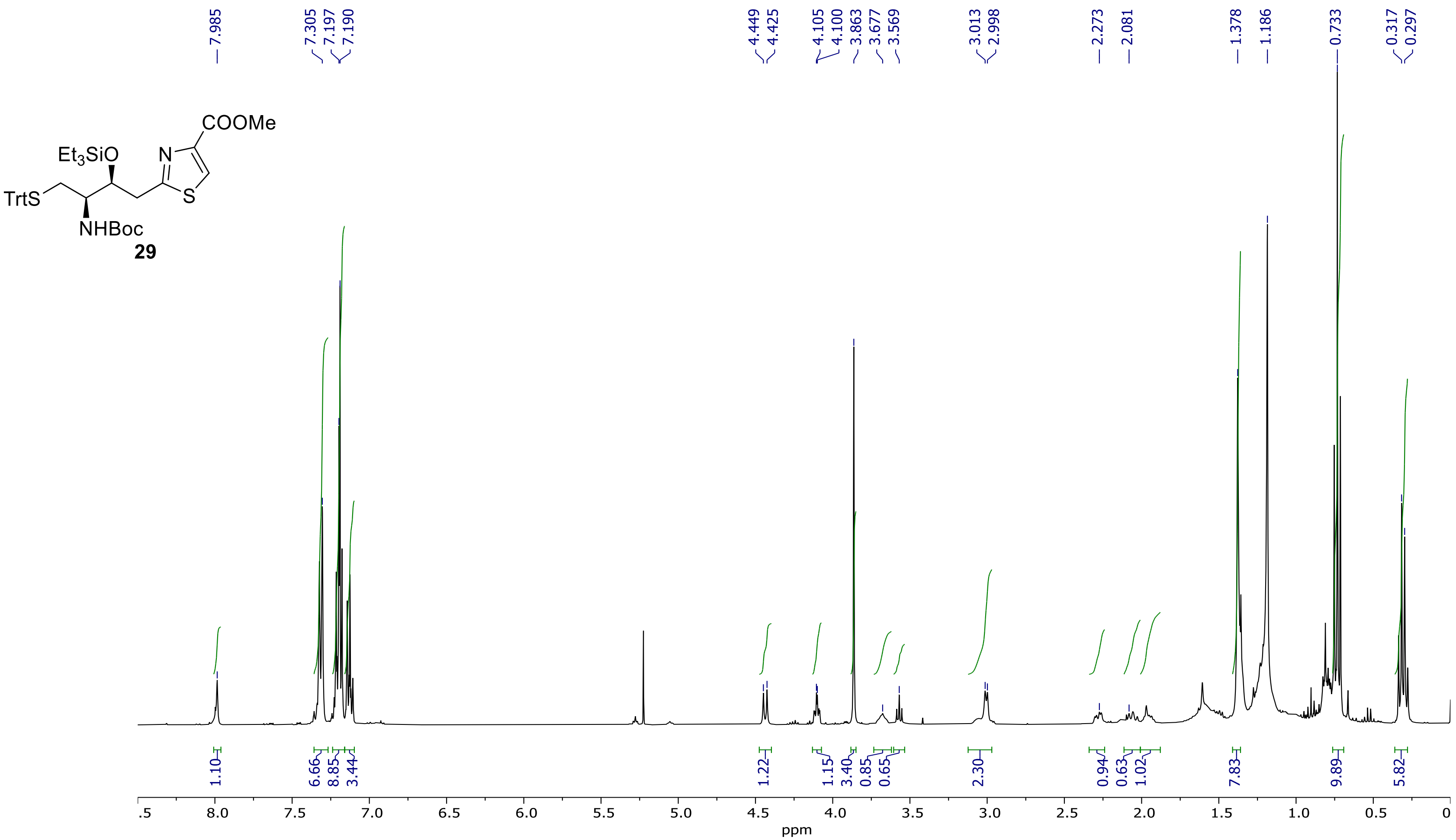

29

^{13}C NMR (100.13 MHz, $CDCl_3$) of **29**

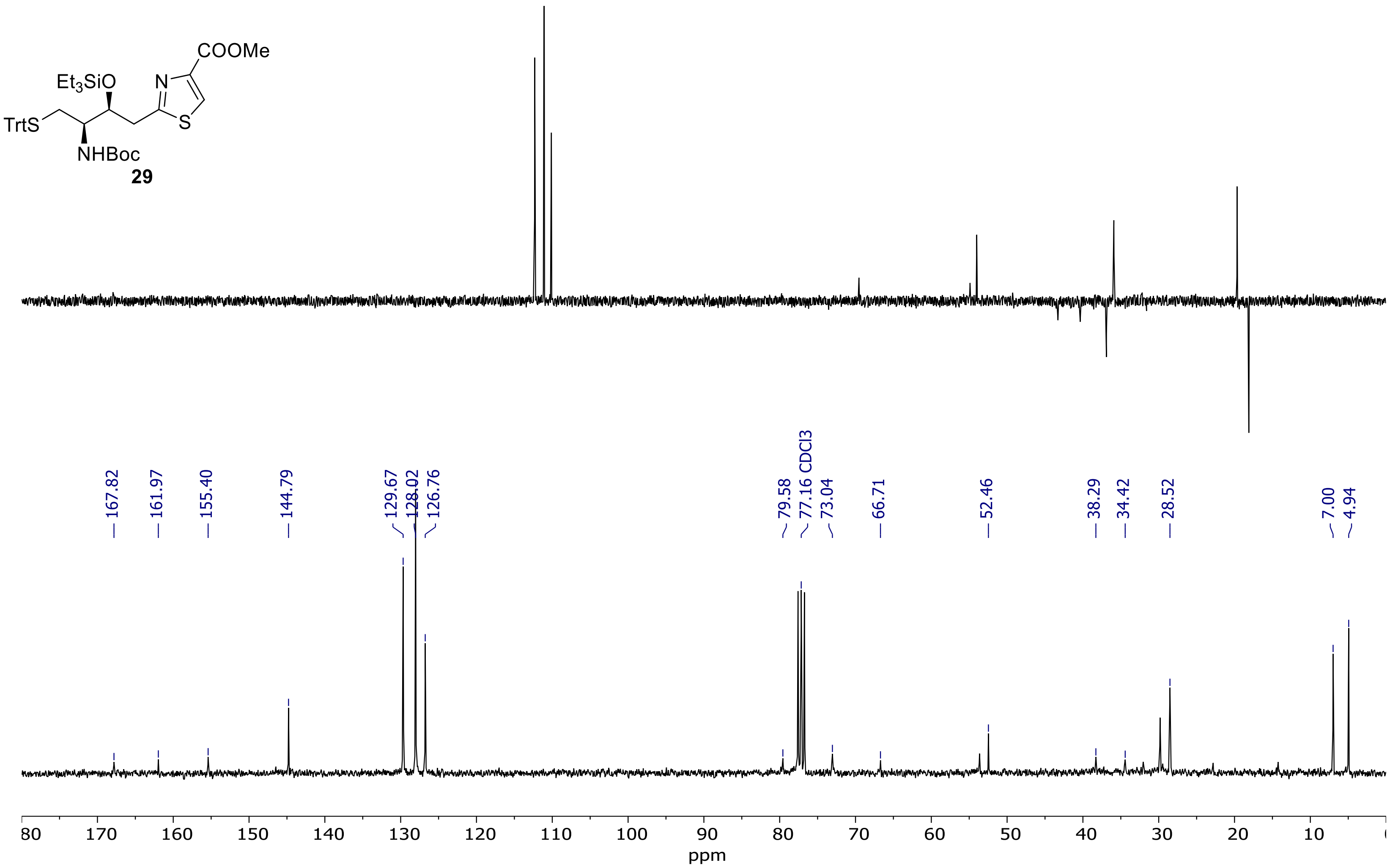

^{1}H-^{1}H COSY (400.13 MHz, $CDCl_3$) of **29**

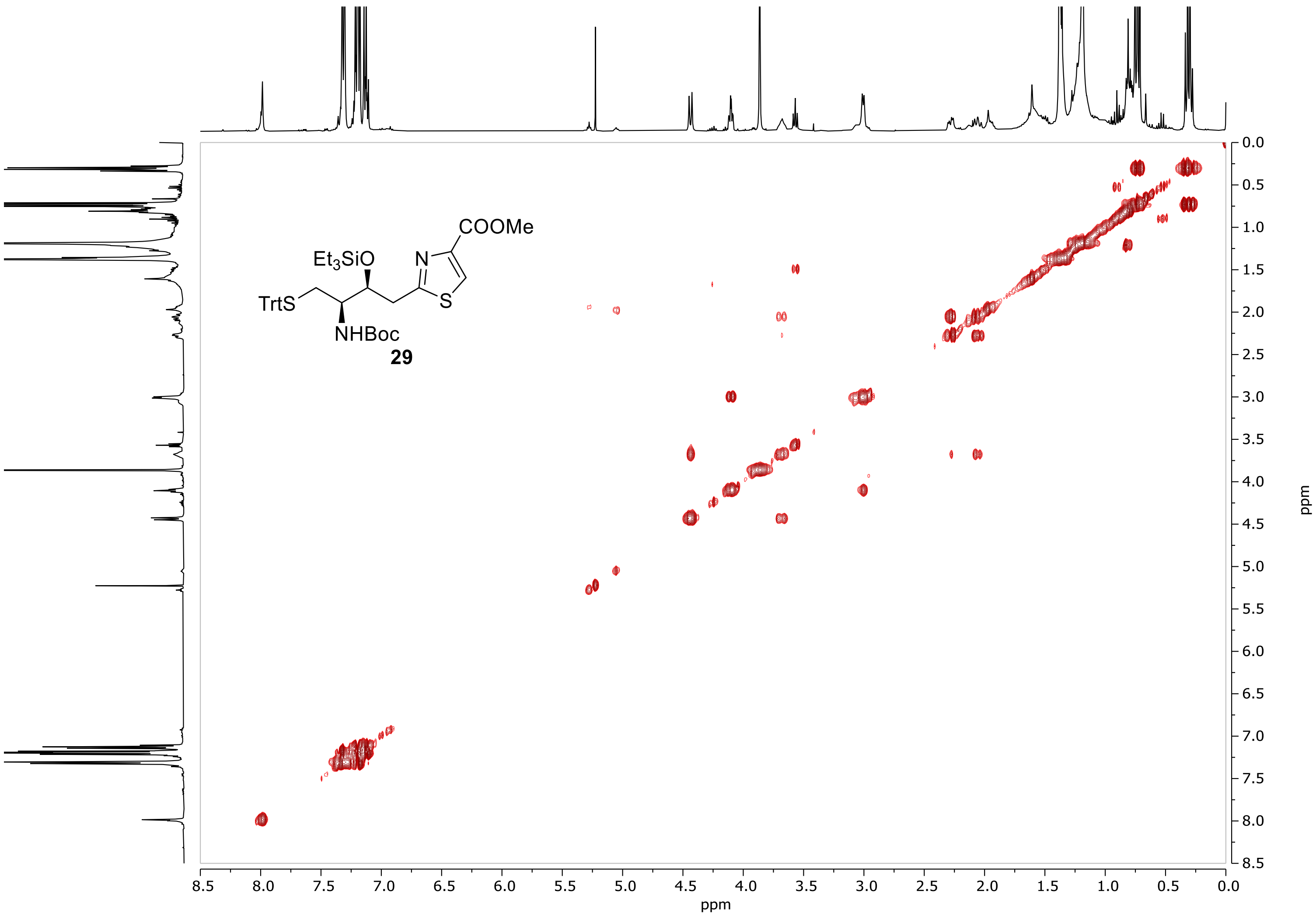

HSQC (400.13 MHz, $CDCl_3$) of **29**

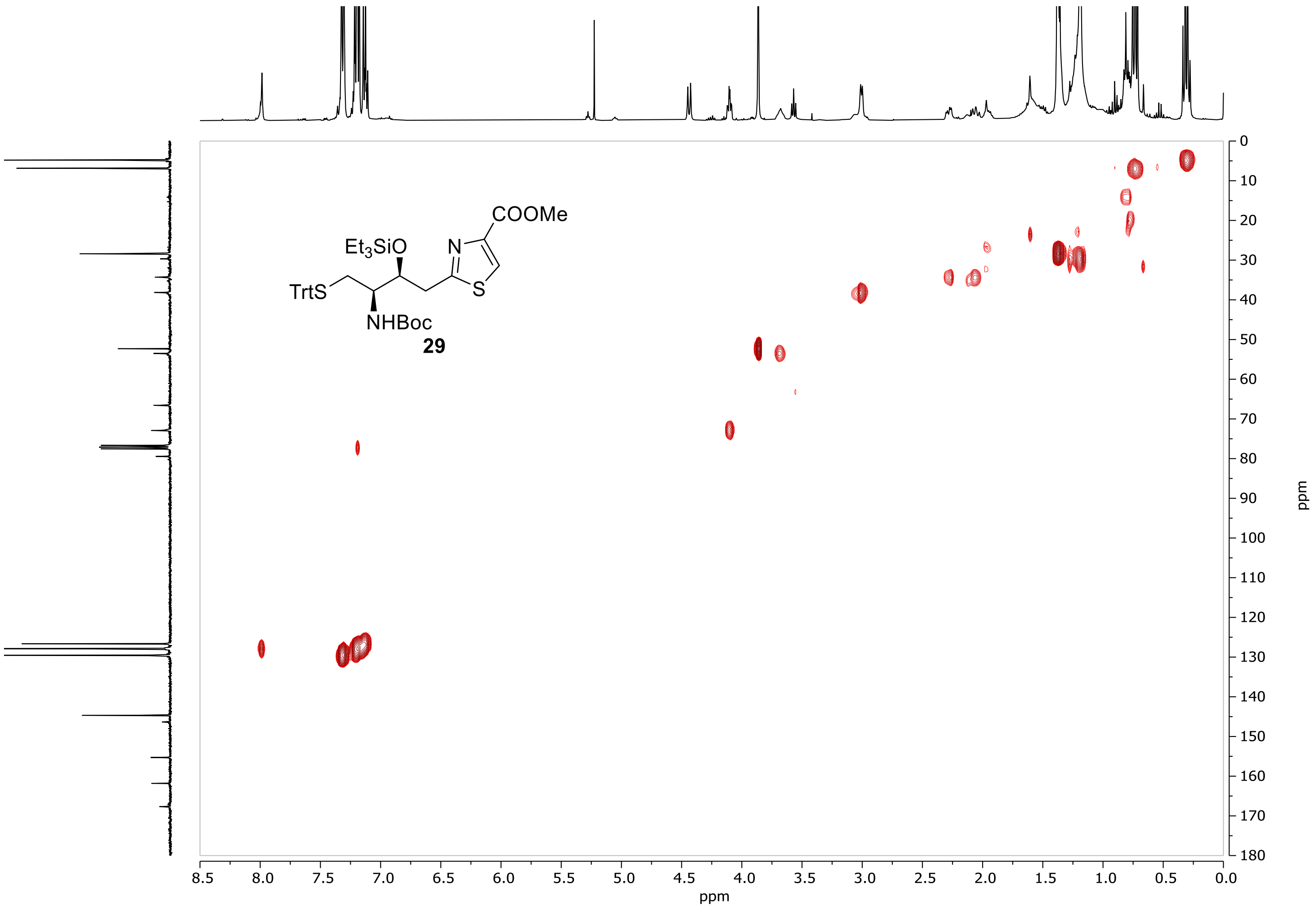

(+)-HRESIMS of **29**: Ion *m/z*: 719.3026 [M+H]$^+$

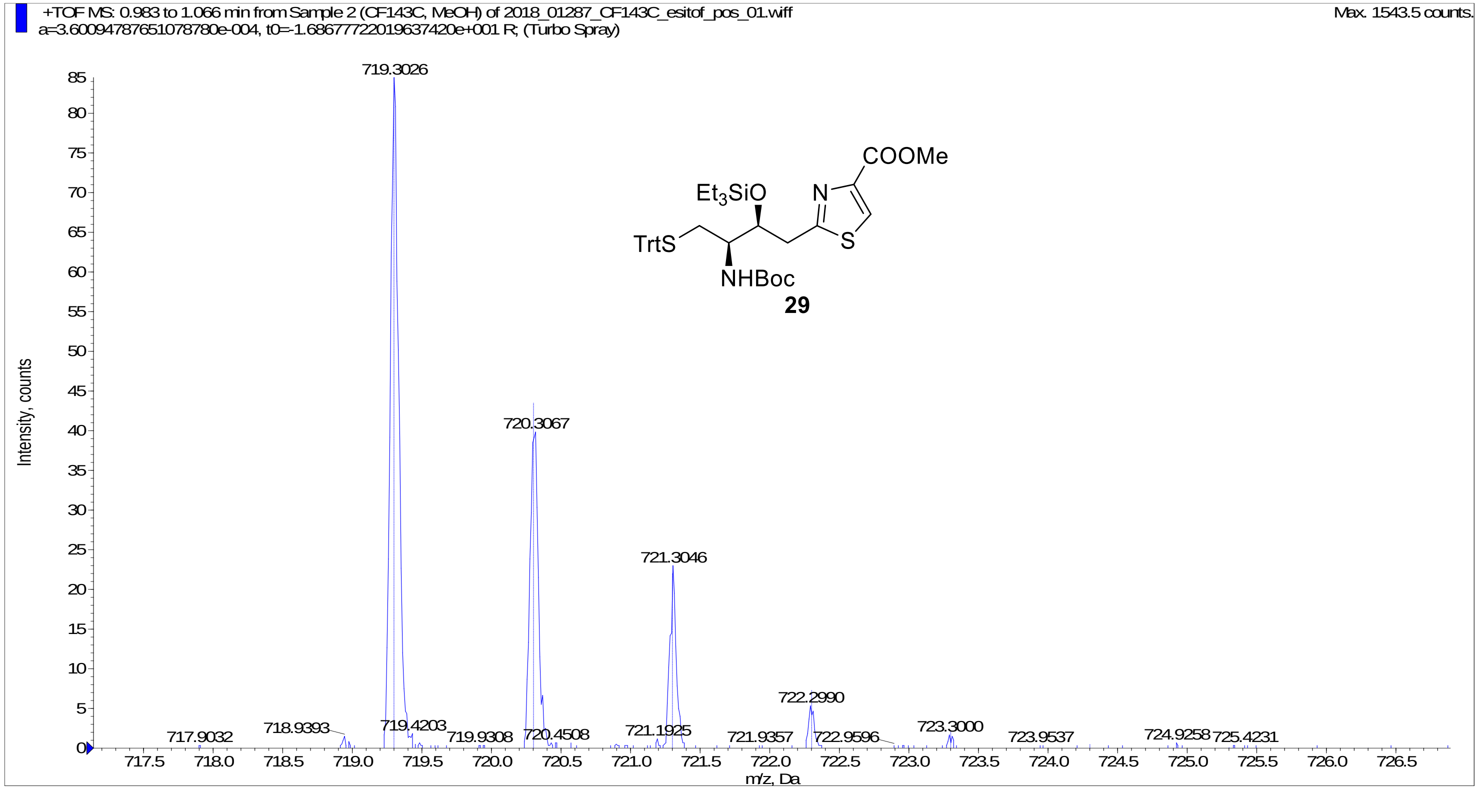

Formula	Calc m/z	Δ, mDa	Δ, ppm	DBE
$C_{39}H_{51}N_2O_5SiS_2$	719.3003	2.2783	3.1674	16.5

3.10 NMR and MS of 30

^{1}H NMR (300.13 MHz, $CDCl_3$ + CD_3OD) of **30**

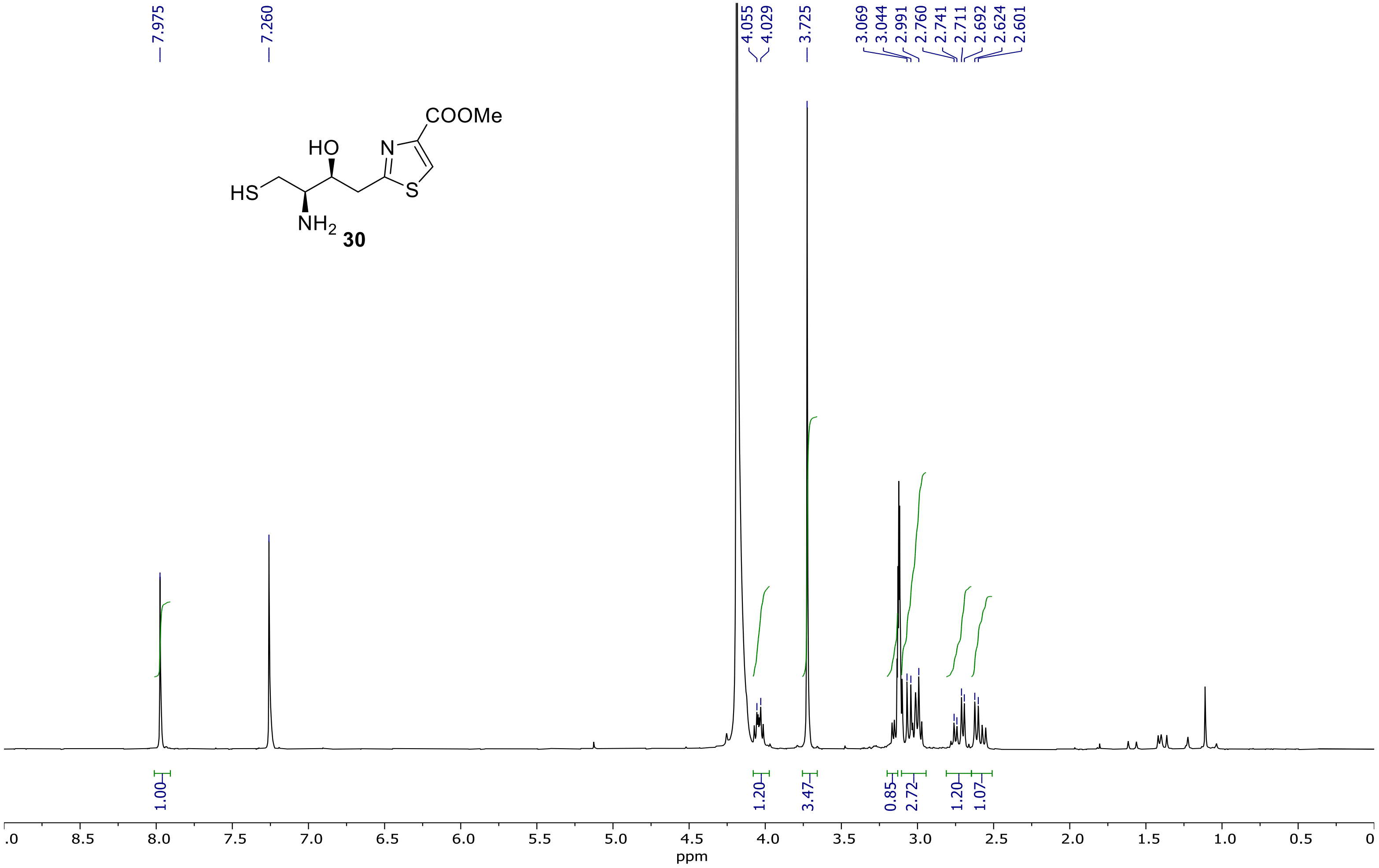

^{13}C NMR (75 MHz, CD_3OD) of **30**

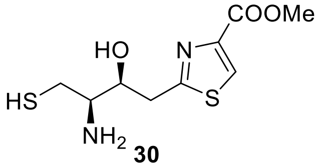
30

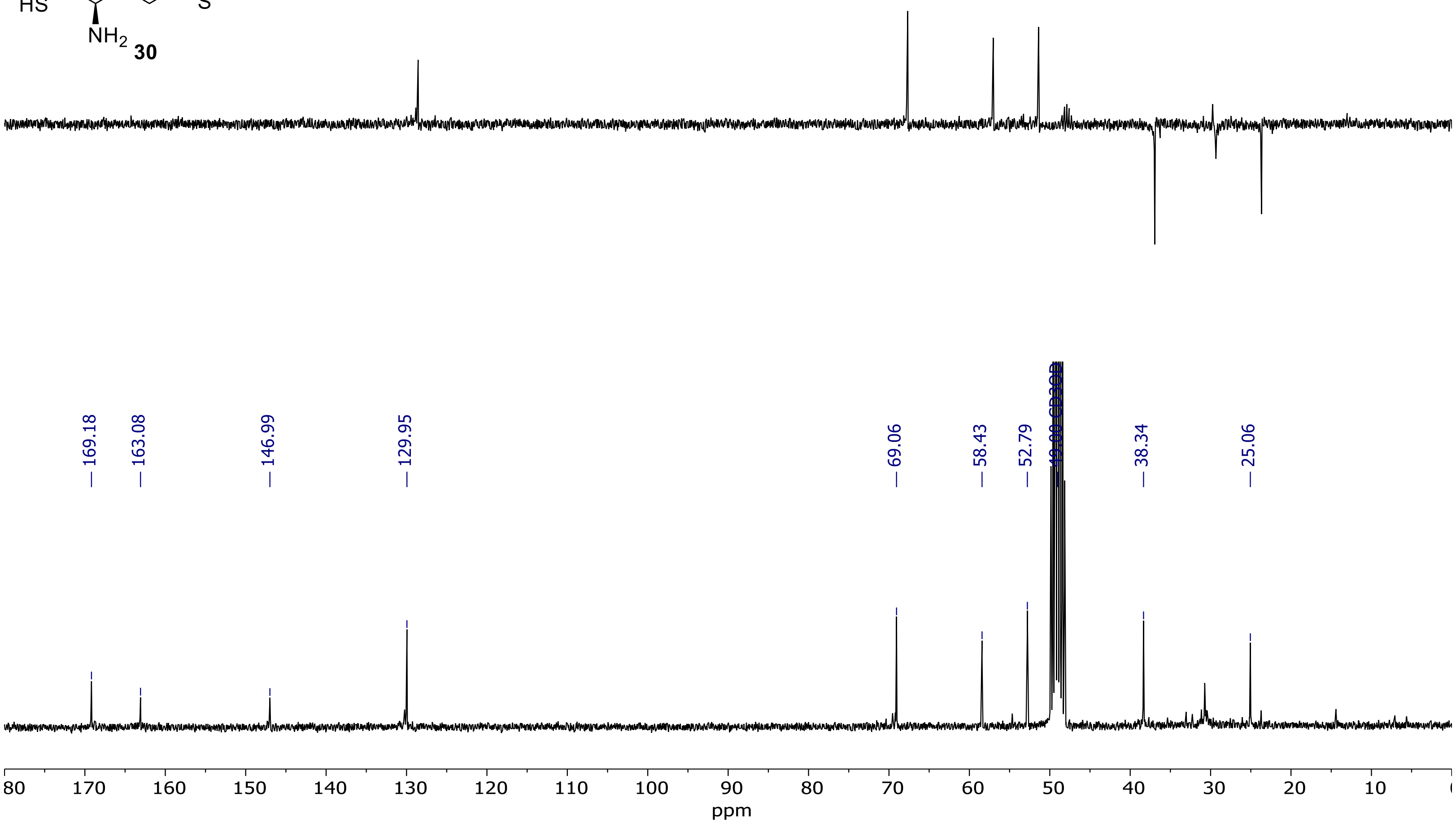

^{13}C NMR (75 MHz, $CDCl_3$ + CD_3OD) of **30**

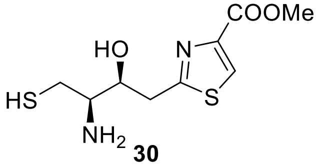

30

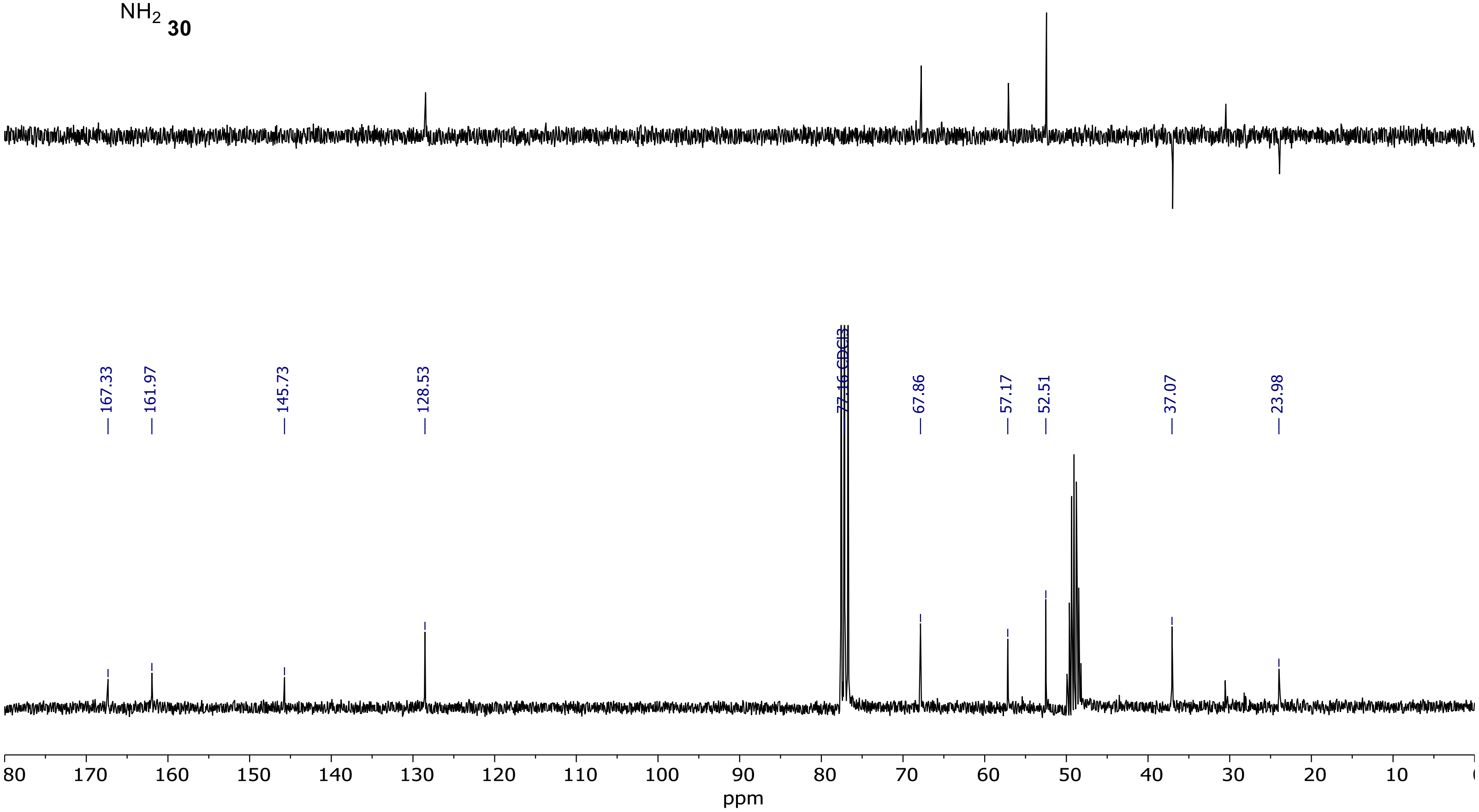

^{1}H-^{1}H COSY (300.13 MHz, CD_3OD) of **30**

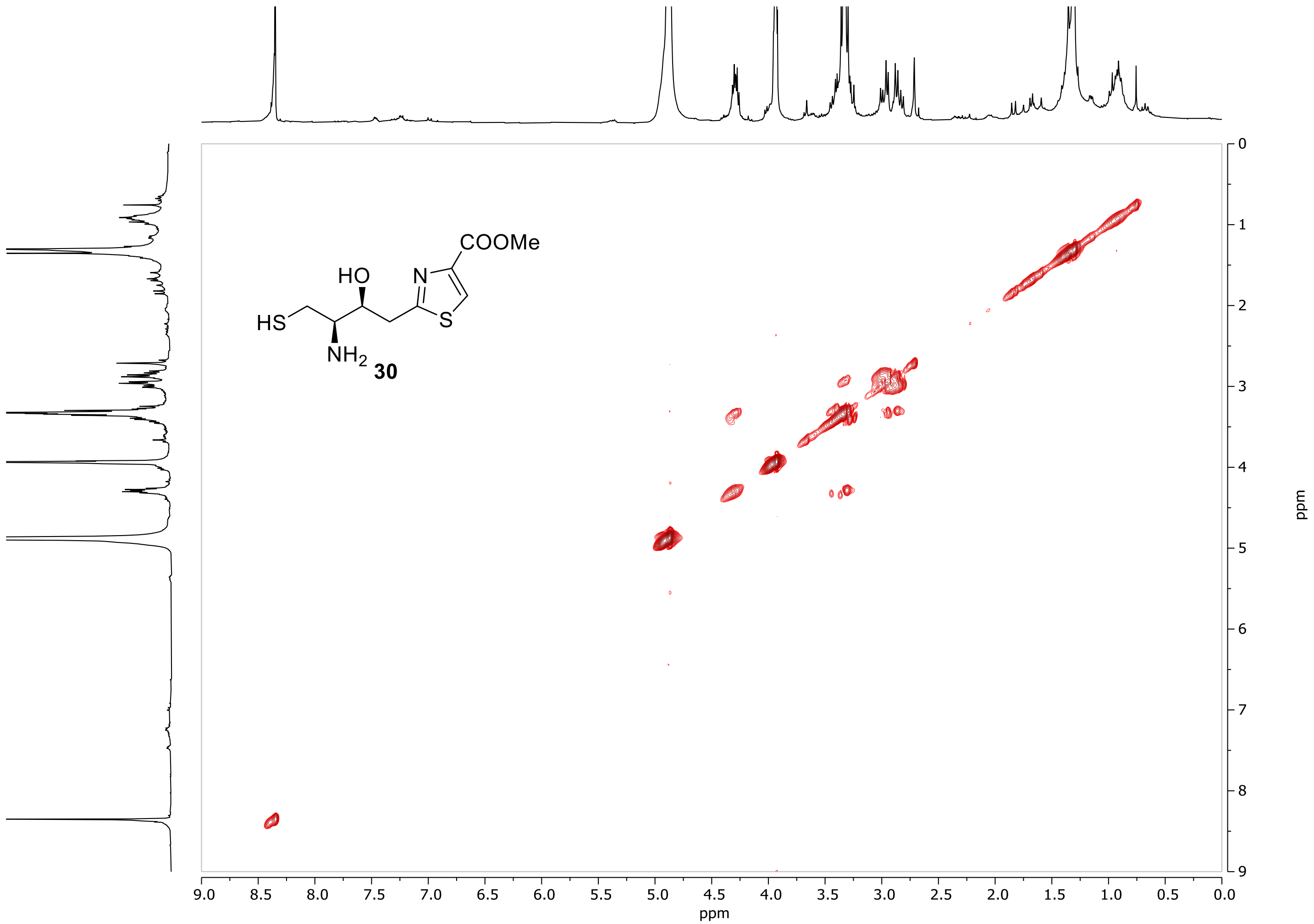

HSQC (300.13 MHz, CD_3OD) of **30**

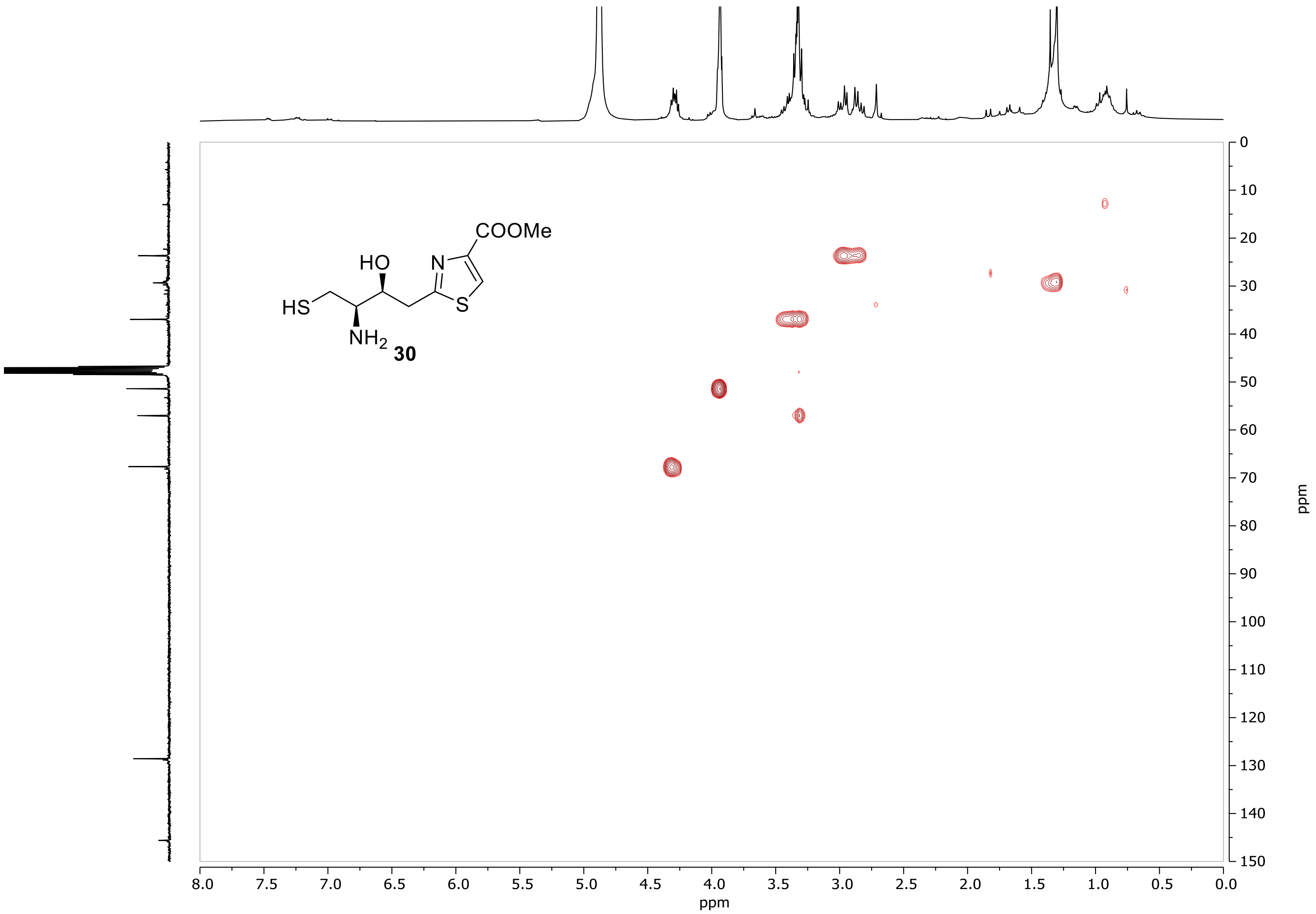

(+)-HRESIMS of **30**: Ion: m/z: 263.0529 ([M+H]$^+$)

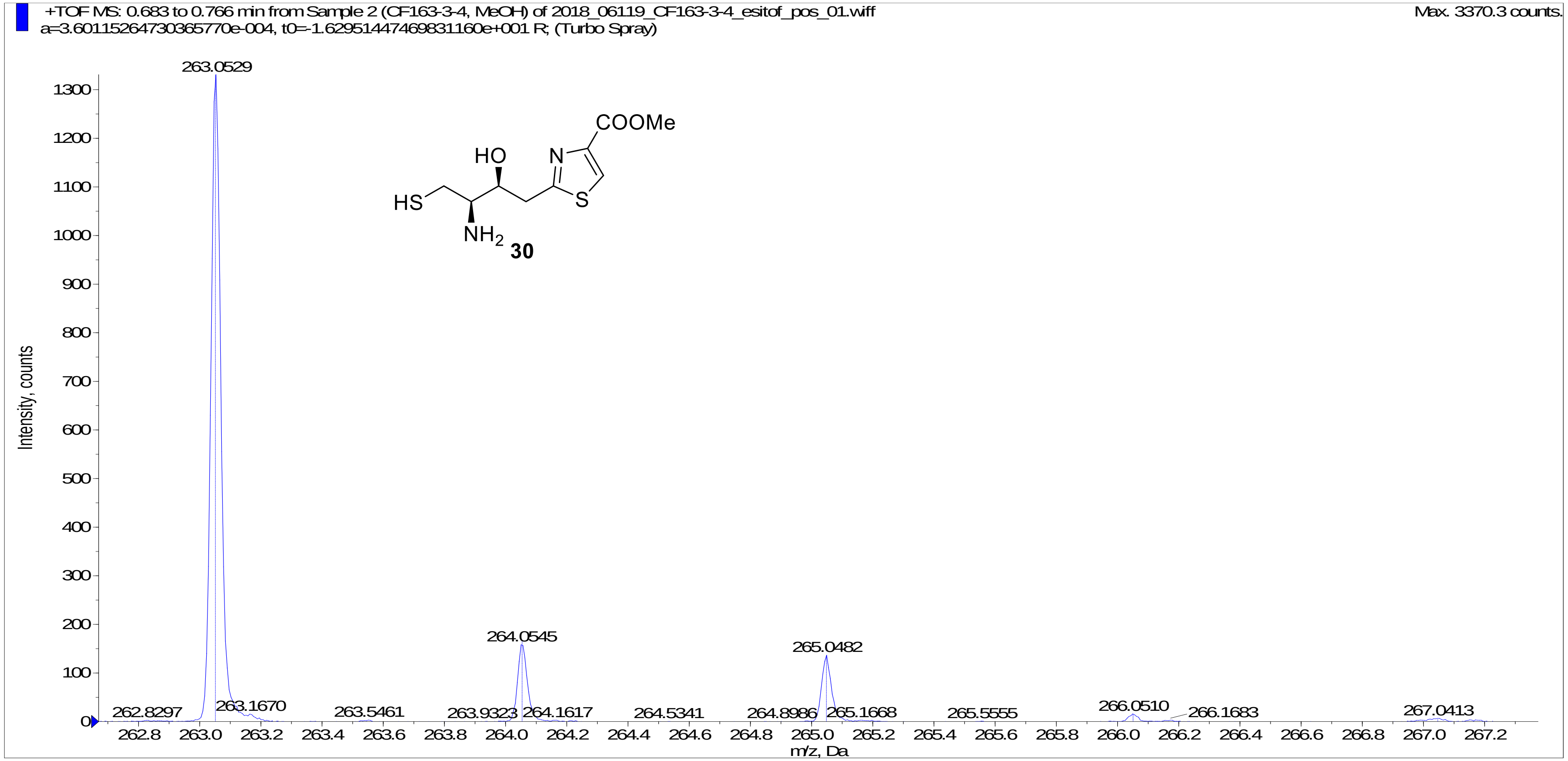

Formula	Calc m/z	Δ, mDa	Δ, ppm	DBE
$C_9H_{15}N_2O_3S_2$	263.0518	1.0374	3.9438	3.5

4. NMR and MS of intermediates from the synthesis of 13*S*-Pcb analogues 6 and 7 and Ga^{+3} complex 32 (Schemes 4 and 5)

4.1 NMR and MS of 31

^{1}H NMR (300.13 MHz, $CDCl_3$) of **31**

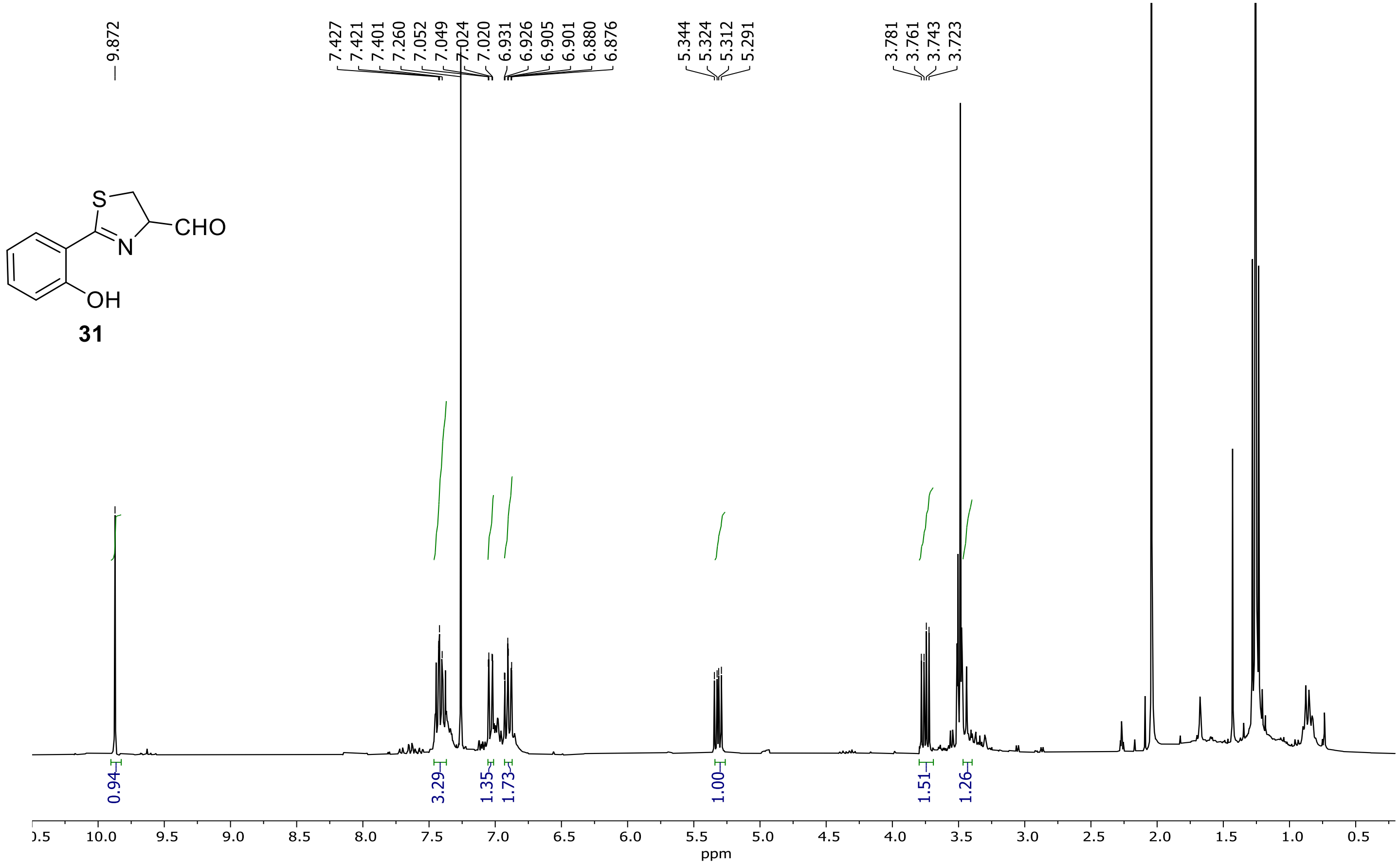

^{13}C NMR (75 MHz, $CDCl_3$) of **31**

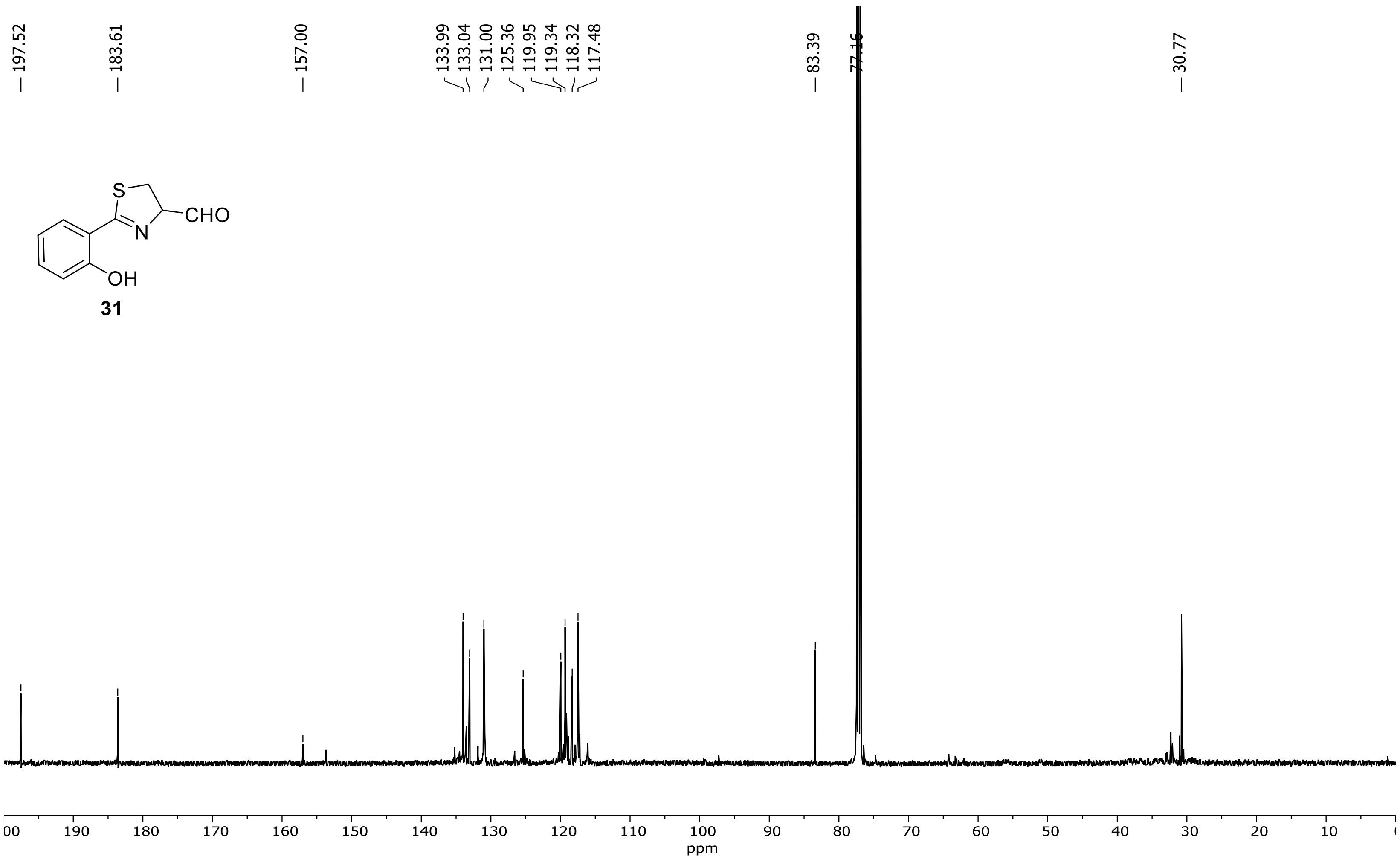

31

4.2 NMR and MS of 6a

Table S2: ^{1}H NMR (500.13 MHz) and ^{13}C NMR (125.13 MHz) spectral data of **6a** in CD_2Cl_2

C	Mult.	δ_C	δ_H mult (*J* Hz)
**1**	C	159.59	-
**2**	CH	117.41	6.97 bd (7.7)
**3**	CH	133.67	7.36 (t 7.7)
**4**	CH	119.27	6.87 t (7.7)
**5**	CH	131.08	7.41 dd (7.7, 1.4)
**6**	C	116.53	-
**7**	C	173.37	-
**8**	CH_2	34.95	*h*: 3.38 dd, (11.3, 7.8)<br>*l*: 3.55 dd, (11.3, 8.8)
**9**	CH	80.72	4.96 ddd (6.8, 7.8, 8.8)
**10**	CH	72.66	4.75 d (6.8)
**11**	CH_2	36.02	*h* : 2.93 t (9.9)<br>*l* : 3.05 dd (9.9, 6.1)
**12**	CH	69.10	3.30 ddd (9.9, 6.1, 3.6)
**13**	CH	70.37	4.24 dt (9.1, 3.6, 3.6)
**14**	CH_2	39.42	*h* : 3.30 dd (17.0 , 3.6)<br>*l* : 3.38 dd (17.0, 9.1)
**15**	C	168.76	-
**16**	CH	128.00	8.07 s
**17**	C	146.74	-
**18**	C	161.92	
**OMe**	CH_3	52.53	3.88 s

^1H NMR (500.13 MHz, CD_2Cl_2) of **6a**

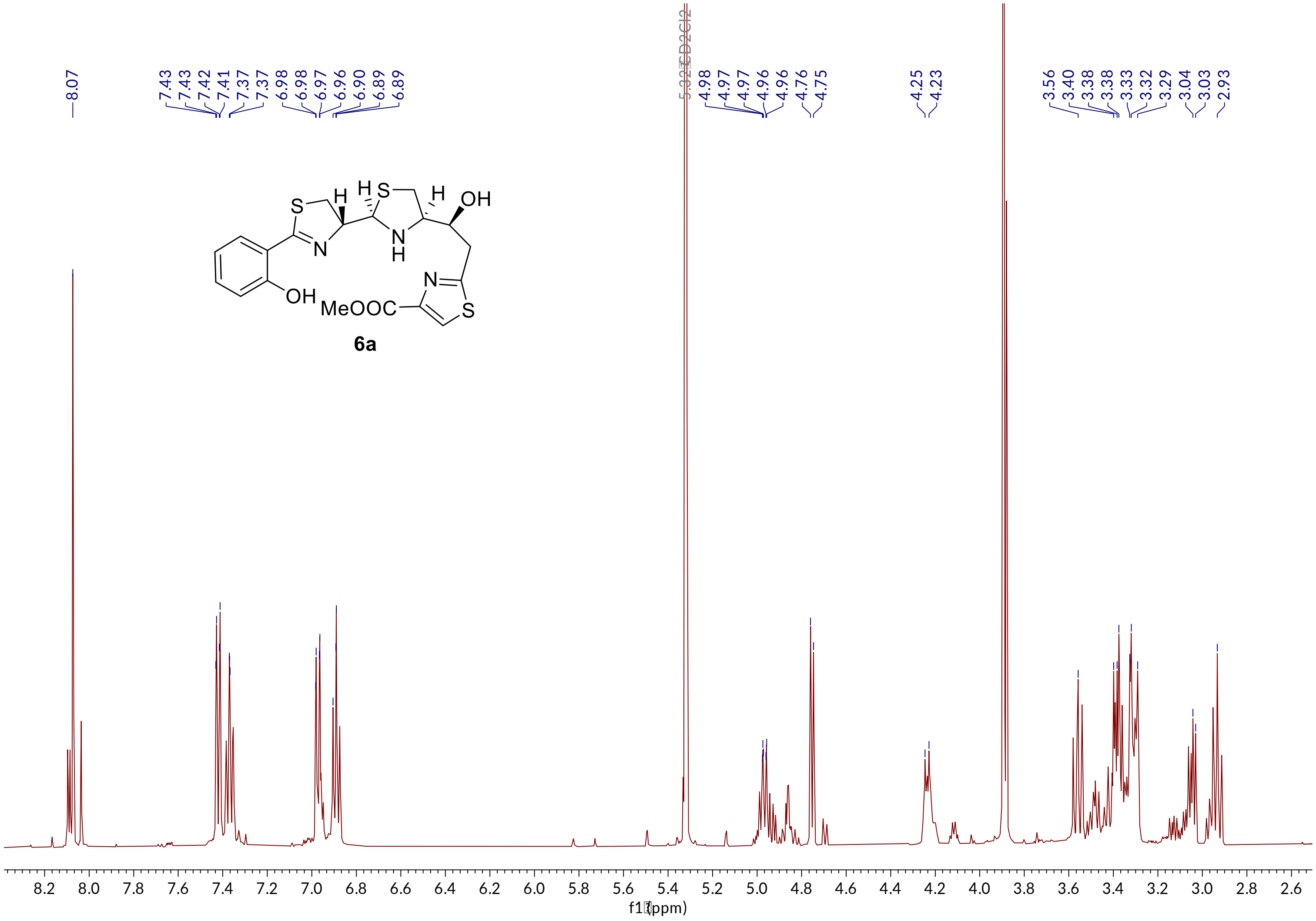

^{13}C NMR (125.13 MHz, CD_2Cl_2) of **6a**

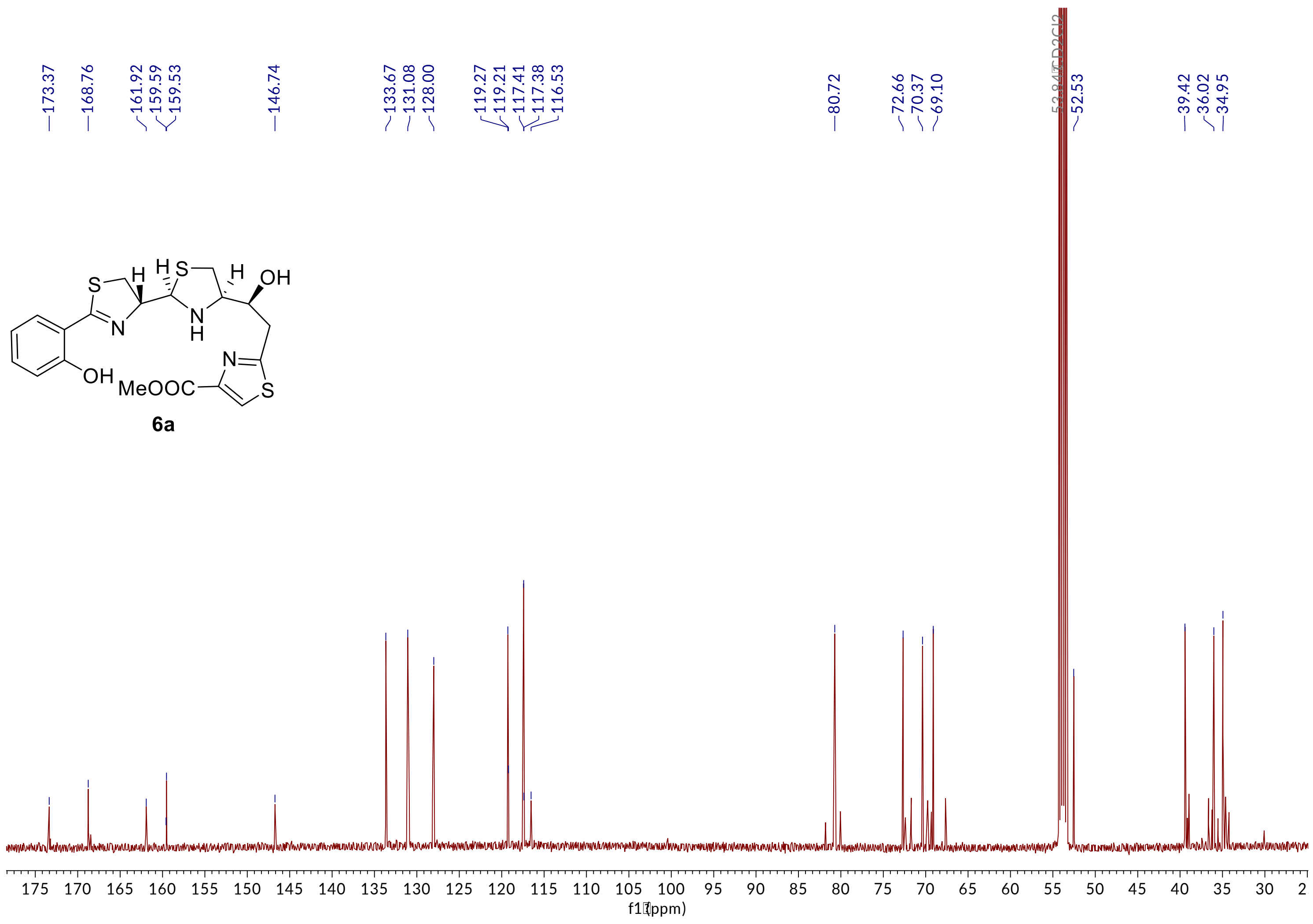

^1H-^1H COSY (500.13 MHz, CD_2Cl_2) of **6a**

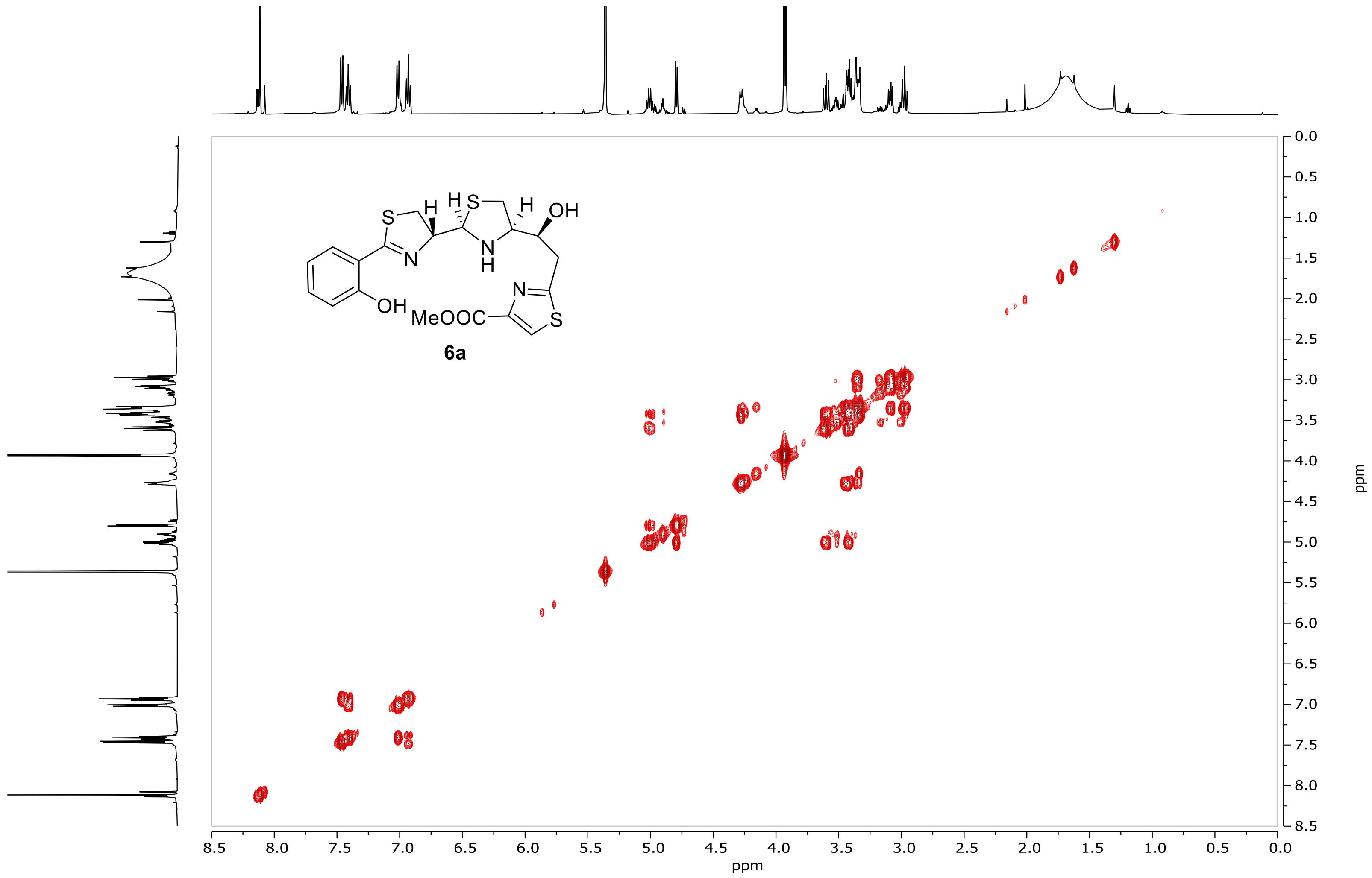

HSQC (500.13 MHz, CD_2Cl_2) of **6a**

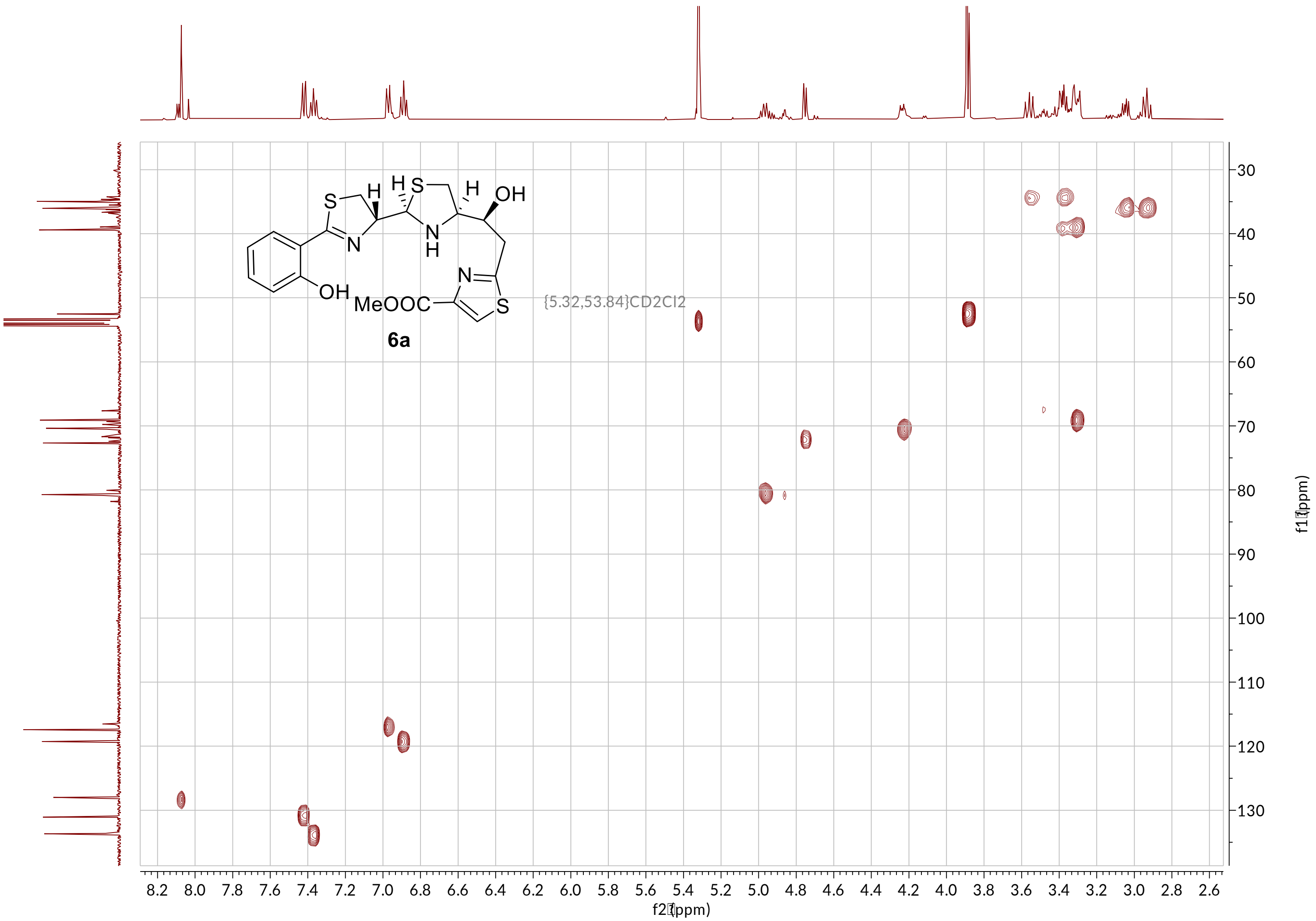

HMBC (500.13 MHz, CD_2Cl_2) of **6a**

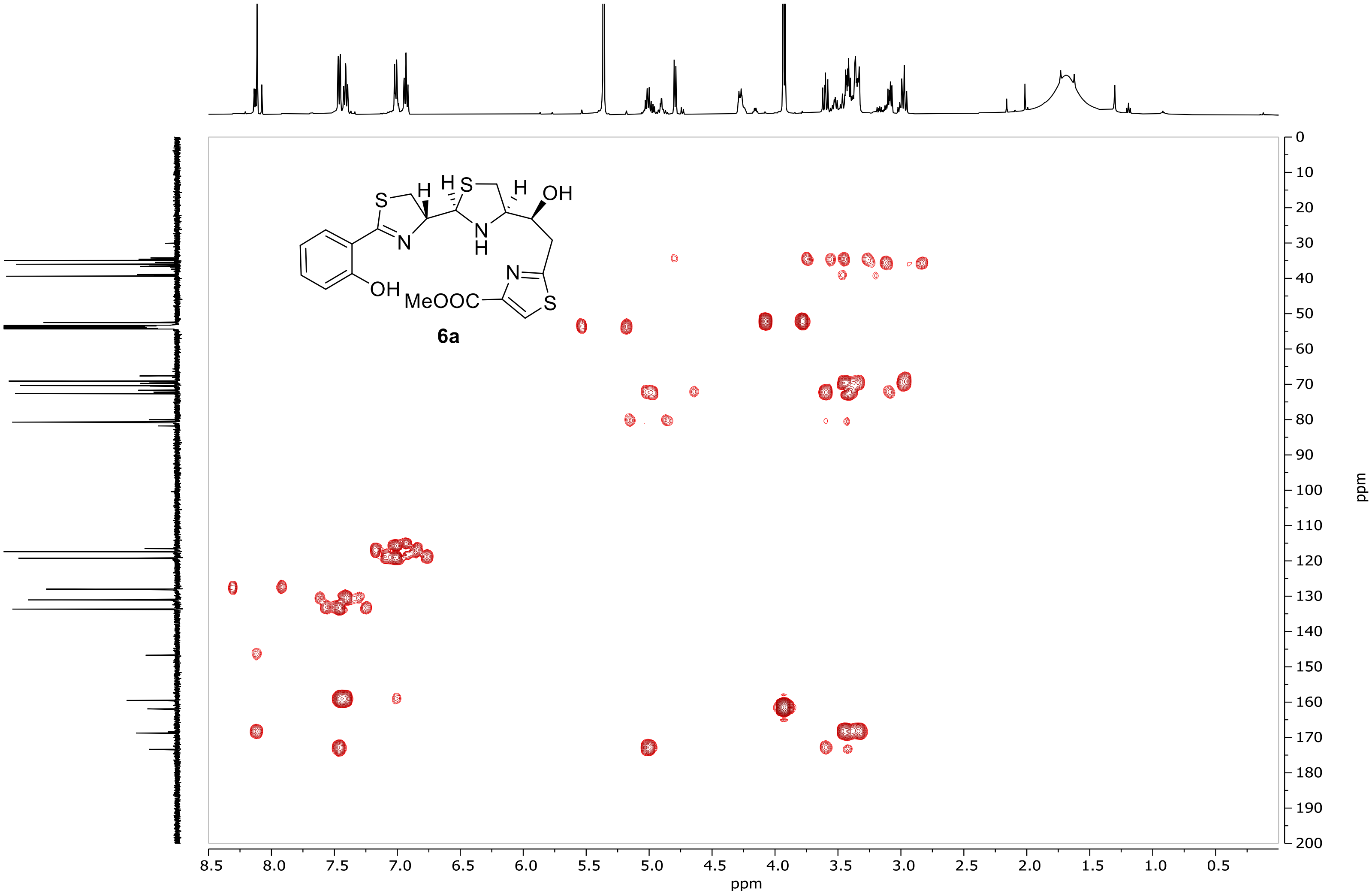

^{1}H-^{1}H NOESY (500.13 MHz, CD_2Cl_2) of **6a**

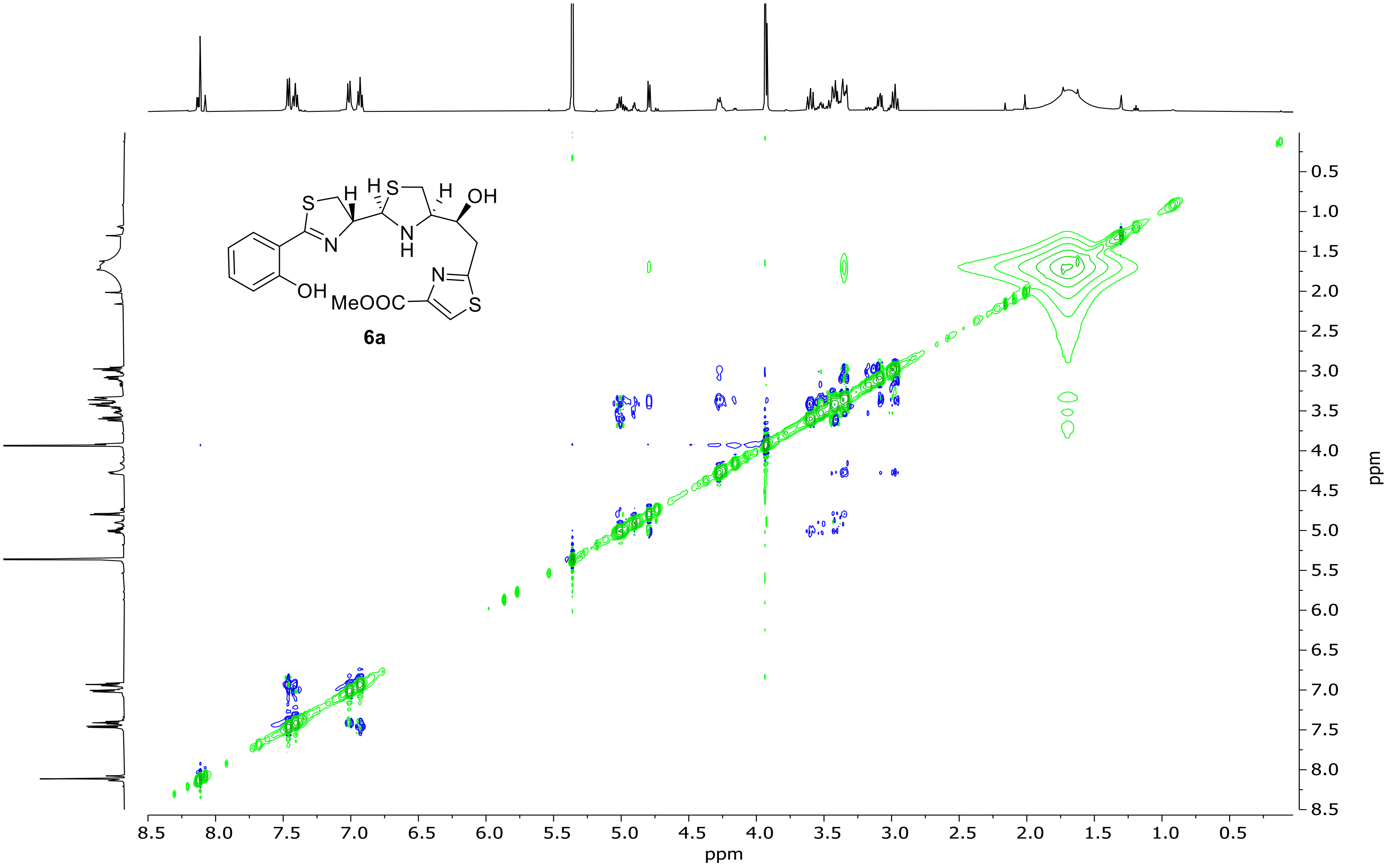

(+)-HRESIMS of **6a**: Ion: *m/z*: 452.0769 $[M+H]^+$

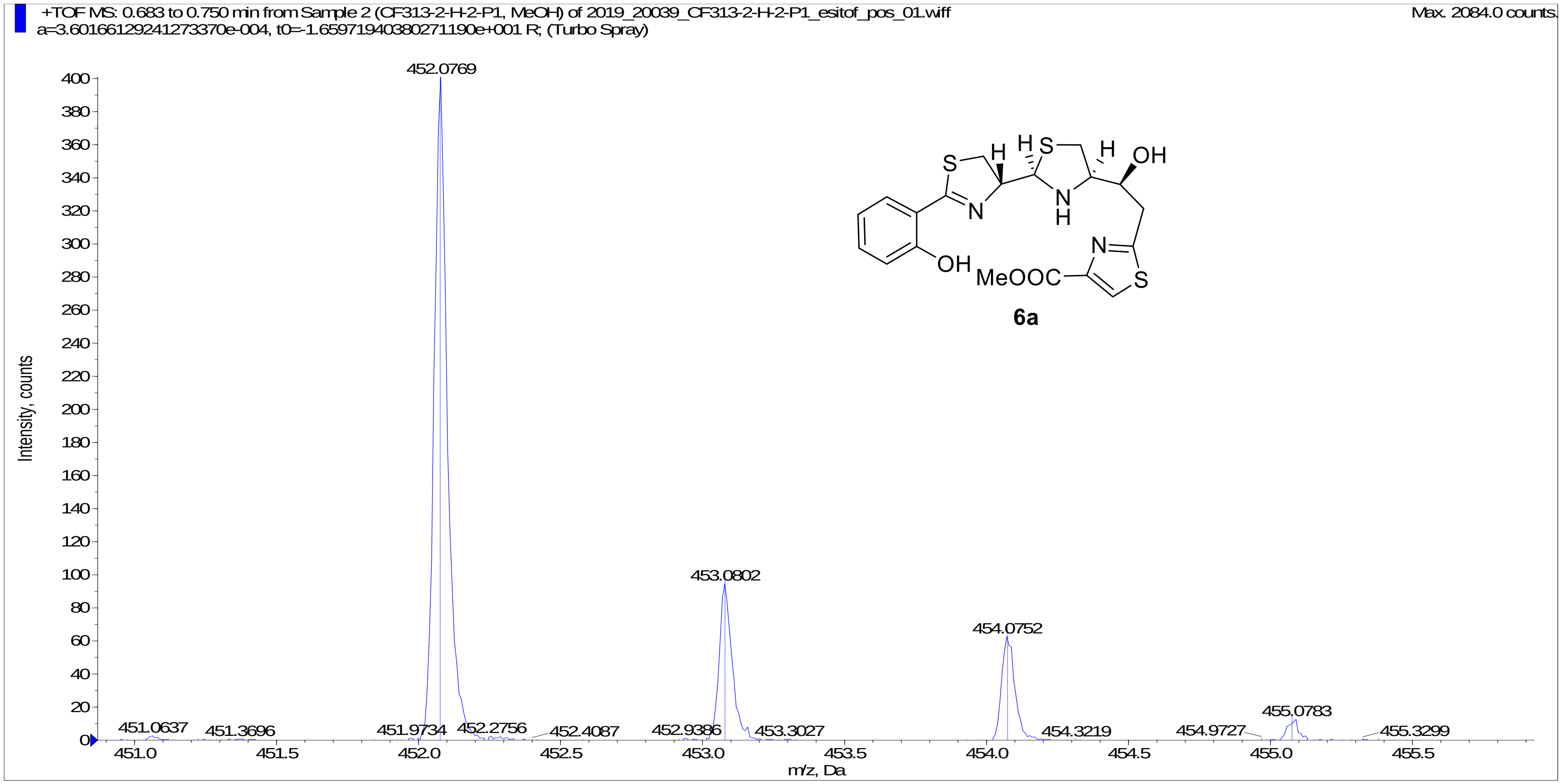

Formula	Calc m/z	Δ, mDa	Δ, ppm	DBE
$C_{19}H_{22}N_3O_4S_3$	452.0766	0.2017	0.4461	10.5

HPLC chromatogram and UV spectrum of **6a**

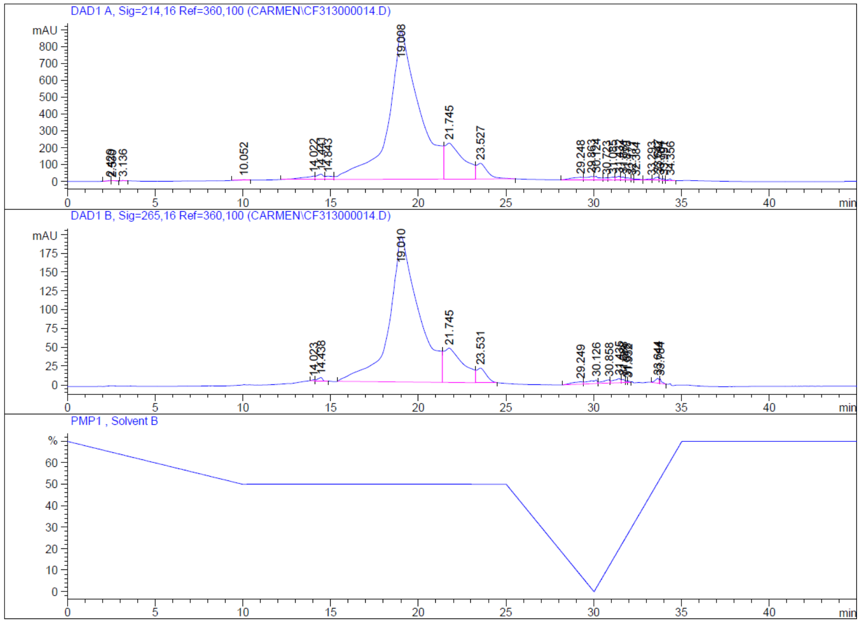

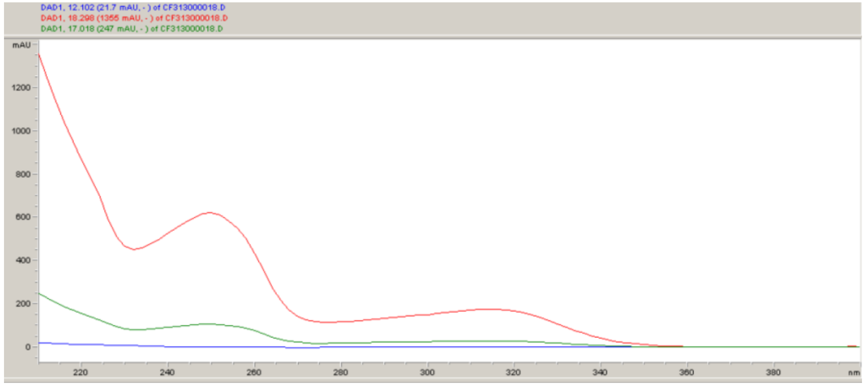

4.3 NMR and MS of a mixture of 6b and 6c (3:2)

Table S3: ^{1}H NMR and ^{13}C NMR chemical shift list of a mixture of **6b** and **6c** (3:2) in CD_2Cl_2.

		**6b**		**6c**	
**C**	**Mult.**	δ_C	δ_H **mult (*J* Hz)**	δ_C	δ_H **mult (*J* Hz)**
**1**	C	159.49	-	159.49	-
**2**	CH	117.34	6.96 bd (7.7)	117.31	6.96 bd (7.7)
**3**	CH	133.57	7.37 (t 7.7)	133.63	7.37 (t 7.7)
**4**	CH	119.19	6.88 t (7.7)	119.16	6.88 t (7.7)
**5**	CH	130.93	7.42 dd (7.7, 1.4)	130.84	7.42 dd (7.7, 1.4)
**6**	C	116.46	-	116.54	-
**7**	C	173.37	-	173.43	-
**8**	CH_2	34.16	*h*: 3.37 m	35.49	*h*:3.38 m
			l: 3.49 dd, (10.8, 8.8)		*l*: 3.55 dd (10.9, 7.9)
**9**	CH	81.72	4.99 dt (5.5, 8.8, 8.8)	79.93	4.83 ddd (8.2, 7.9, 8.7)
**10**	CH	72.39	4.91 d (5.5)	71.78	4.69 d (8.2)
**11**	CH_2	36.22	*h* : 2.94 t (9.8)	36.76	*h*: 2.93 t (9.8)
			l : 3.07 dd (9.8, 6.0)		*l*: 3.09 dd (9.8, 5.9)
**12**	CH	69.27	3.32 dd (9.8, 6.0)	69.63	3.43 dd (10.9, 5.9)
**13**	CH	70.41	4.21 bs	70.37	4.21 bs
**14**	CH_2	39.26	3.31-3.36 m	39.05	3.31-3.36 m
**15**	C	168.58	-	168.71	-
**16**	CH	128.05	8.10 s	128.07	8.09 s
**17**	C	146.57	-	146.57	-
**18**	C	161.87	-	161.87	-
**OMe**	CH_3	52.51	3.88 s	52.52	3.90 s

^{1}H NMR (500 MHz, CD_2Cl_2) of a mixture of **6b** and **6c** (3:2)

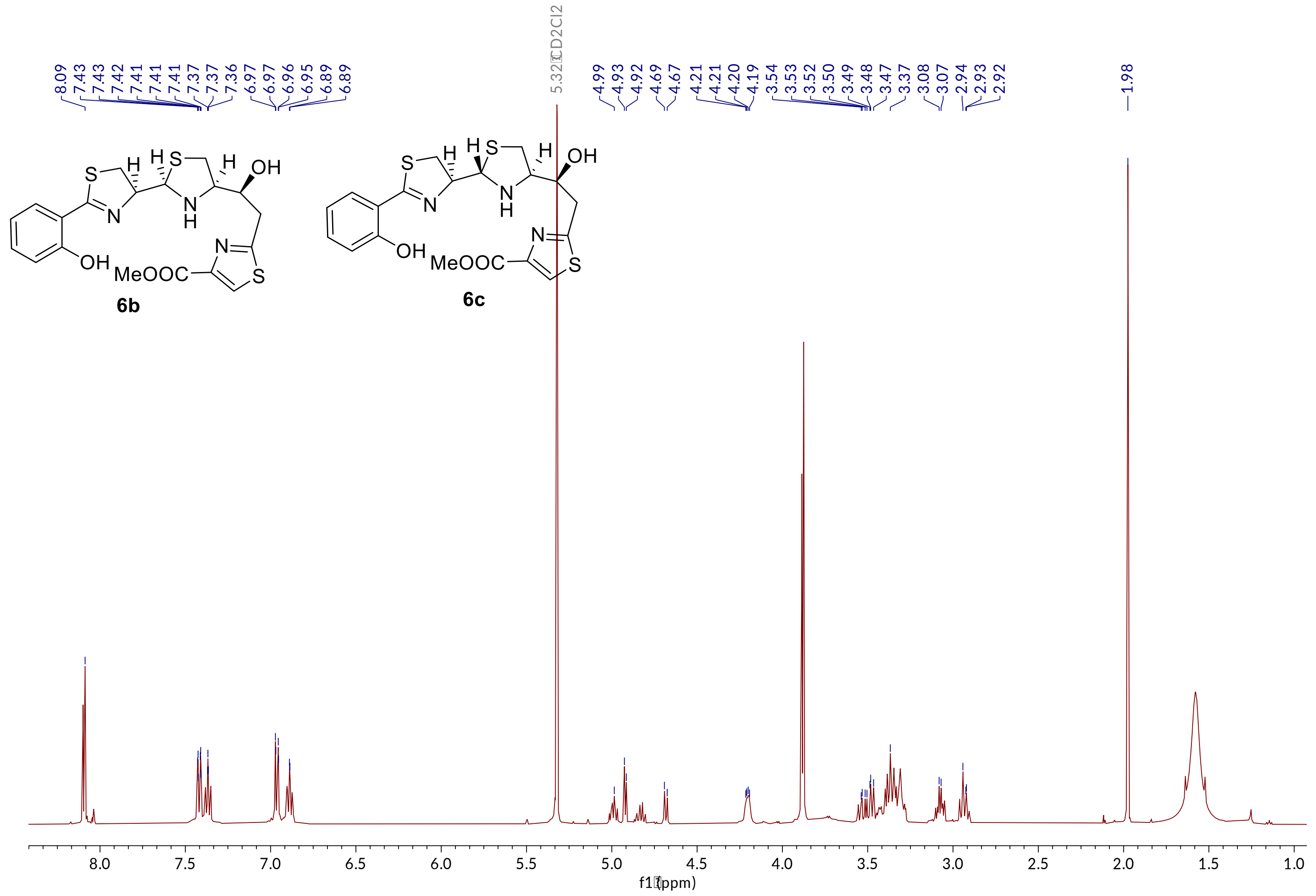

^{13}C NMR (125.13 MHz, CD_2Cl_2) of a mixture of **6b** and **6c** (3:2)

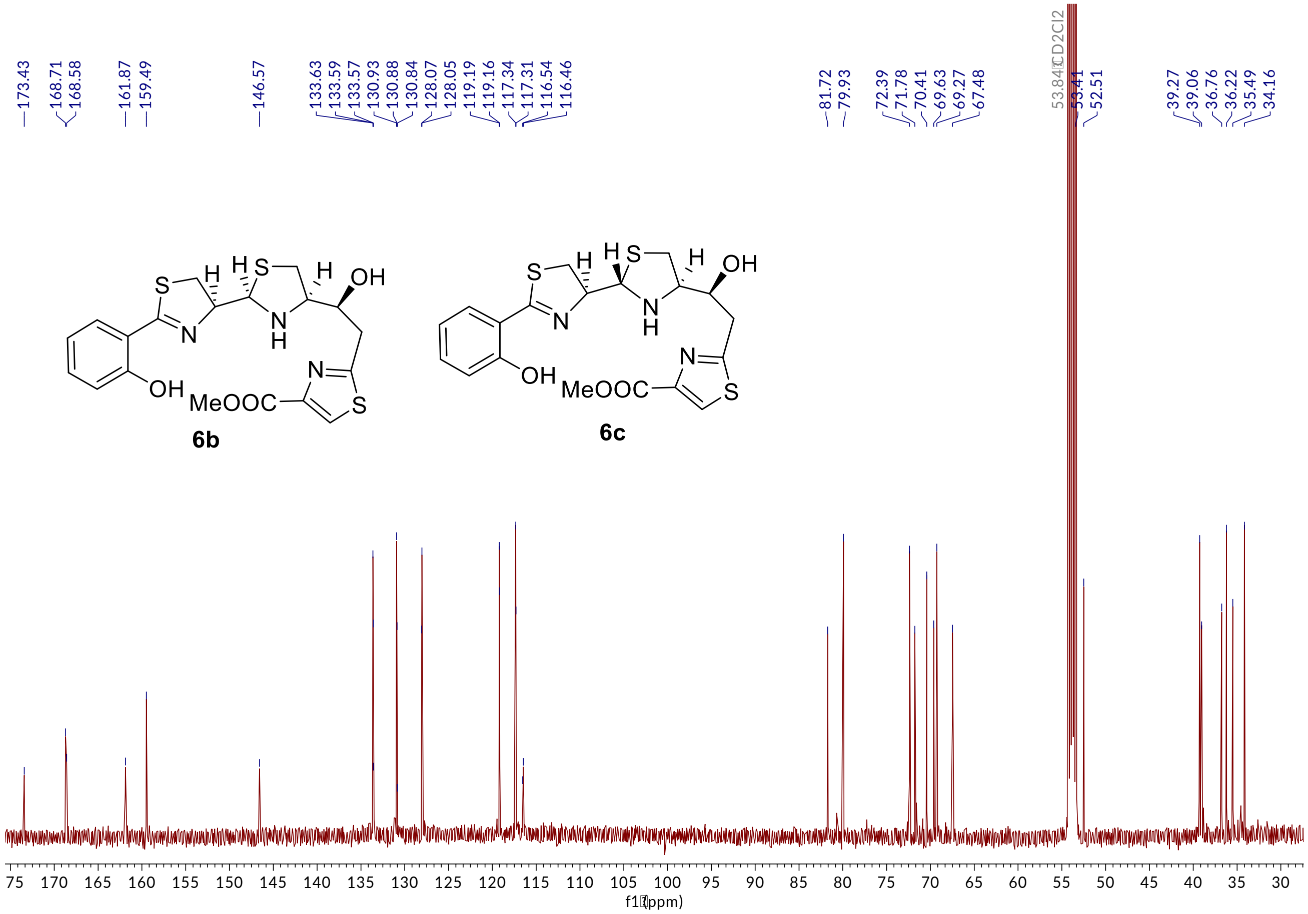

^1H-^1H COSY (500.13 MHz, CD_2Cl_2) of a mixture of **6b** and **6c** (3:2)

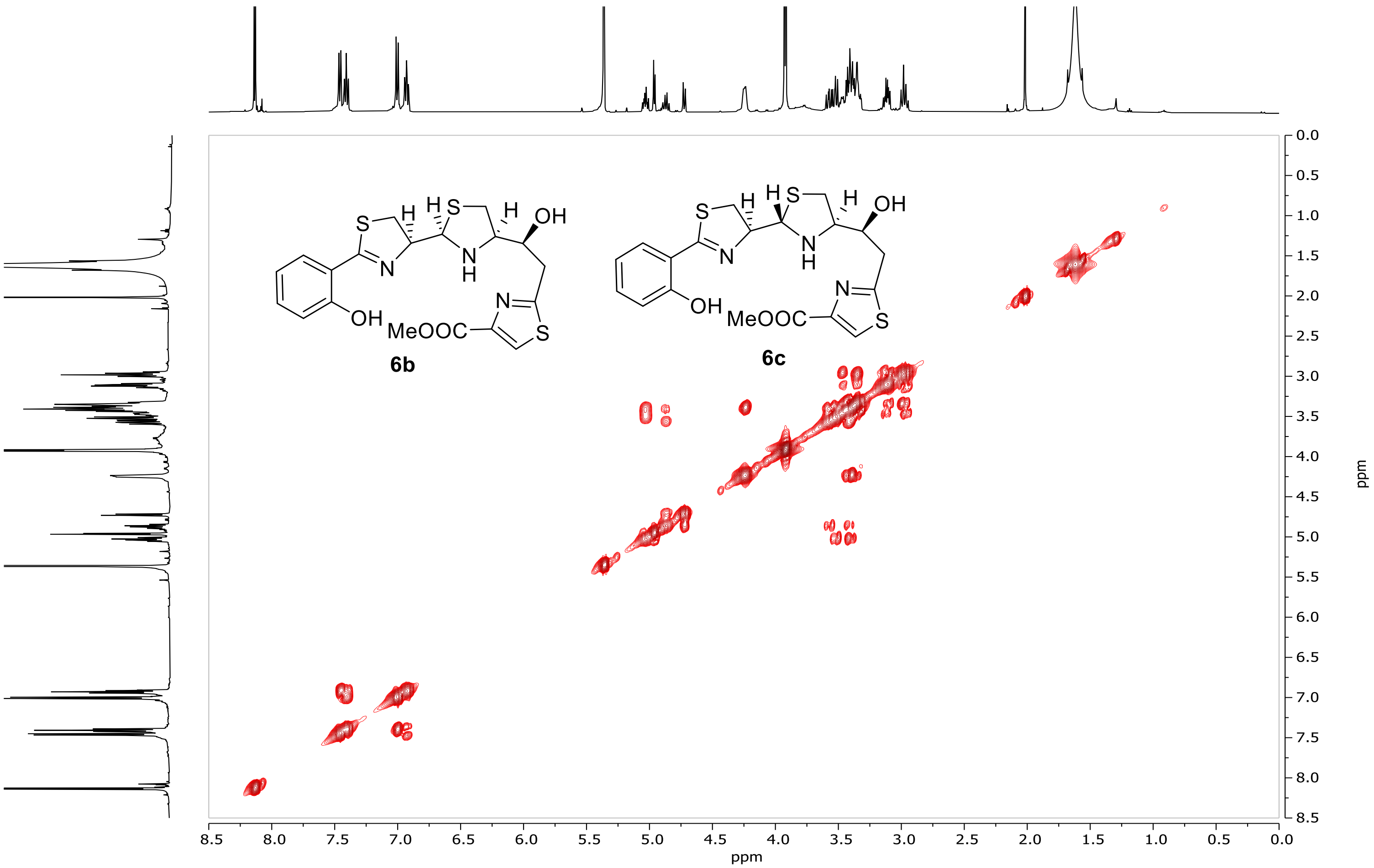

HSQC (500 MHz, CD_2Cl_2) of a mixture of **6b** and **6c** (3:2)

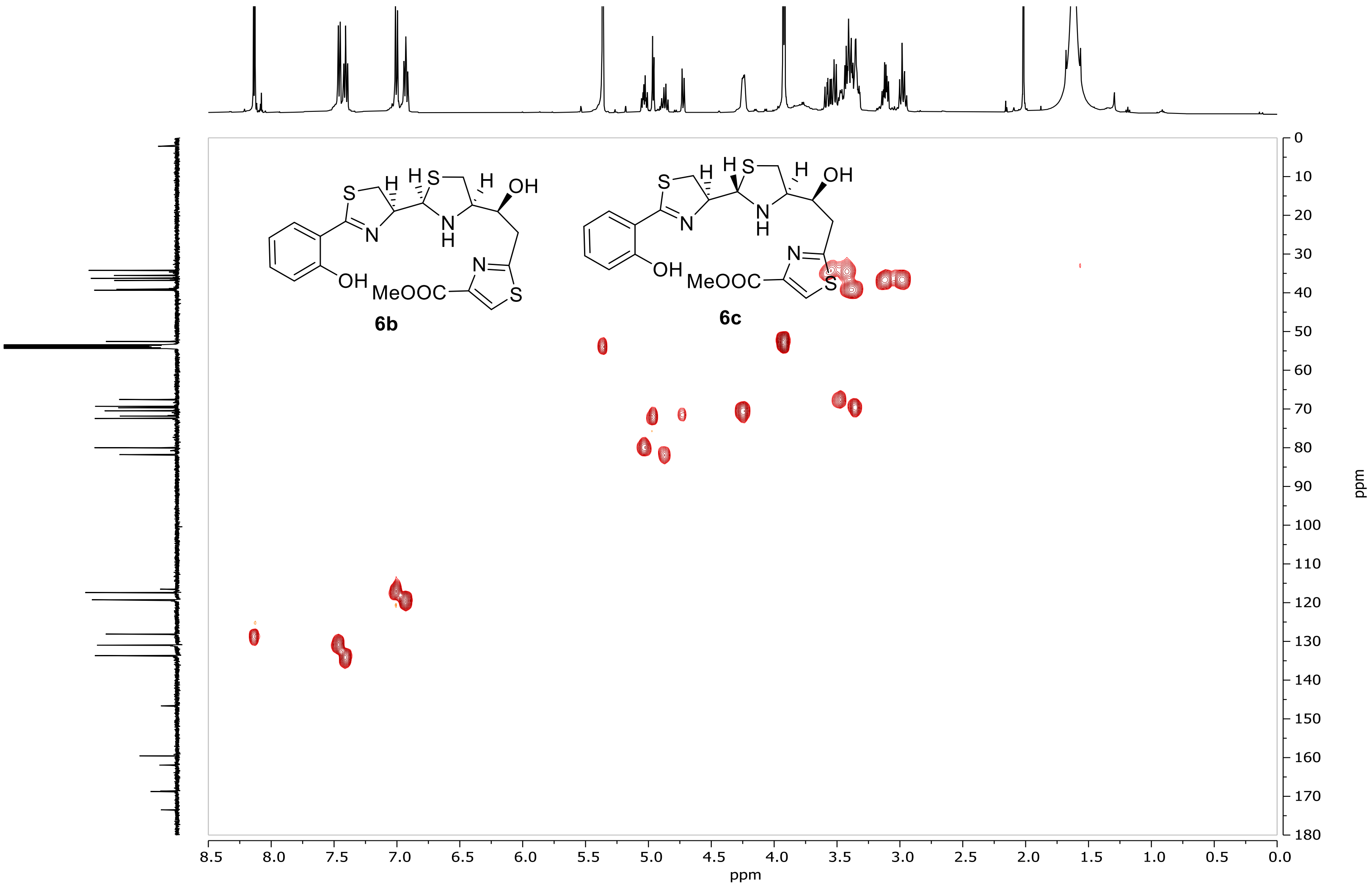

HMBC (500 MHz, CD_2Cl_2) of a mixture of **6b** and **6c** (3:2)

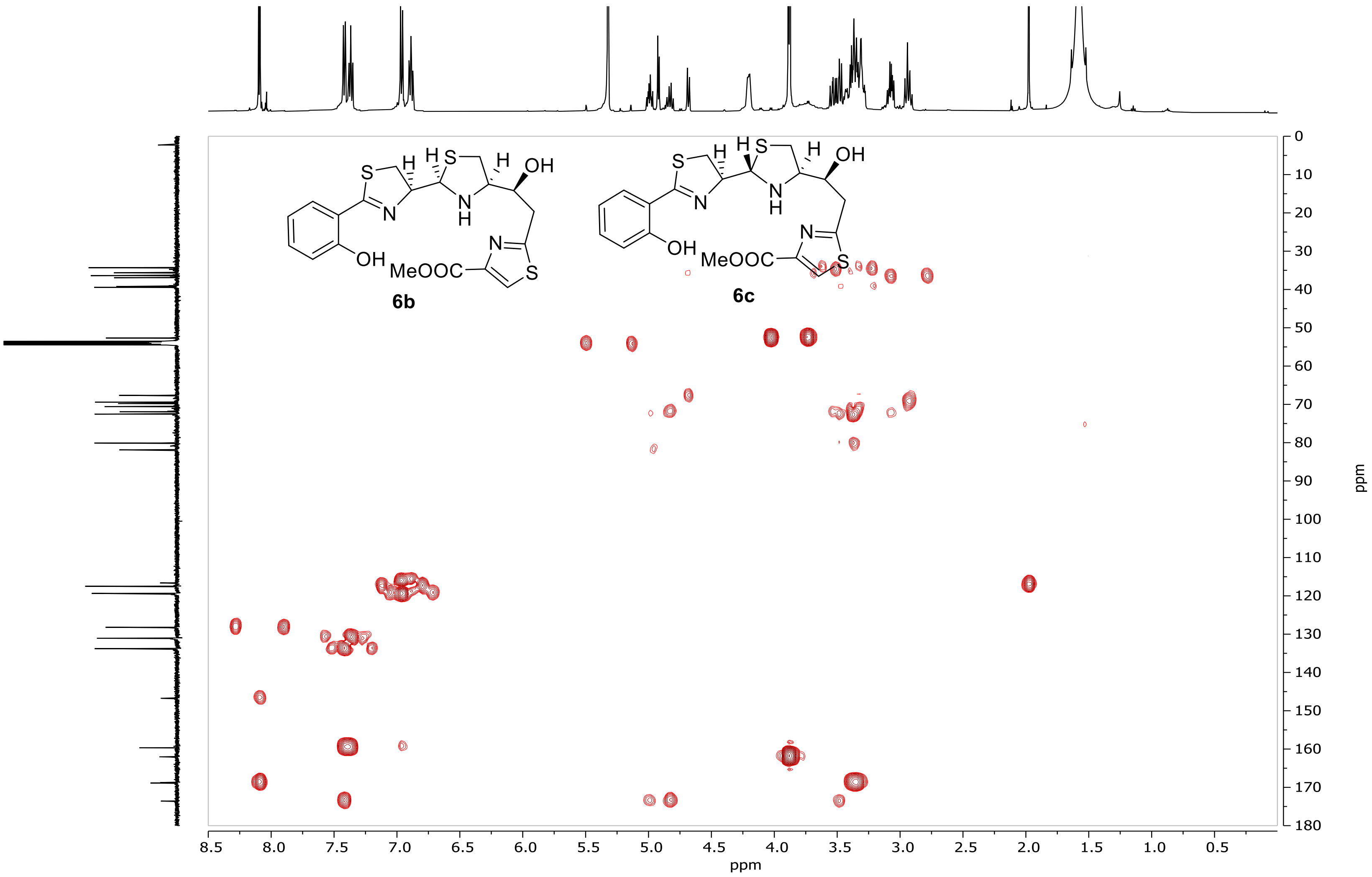

^{1}H-^{1}H NOESY of a mixture of **6b** and **6c** (3:2)

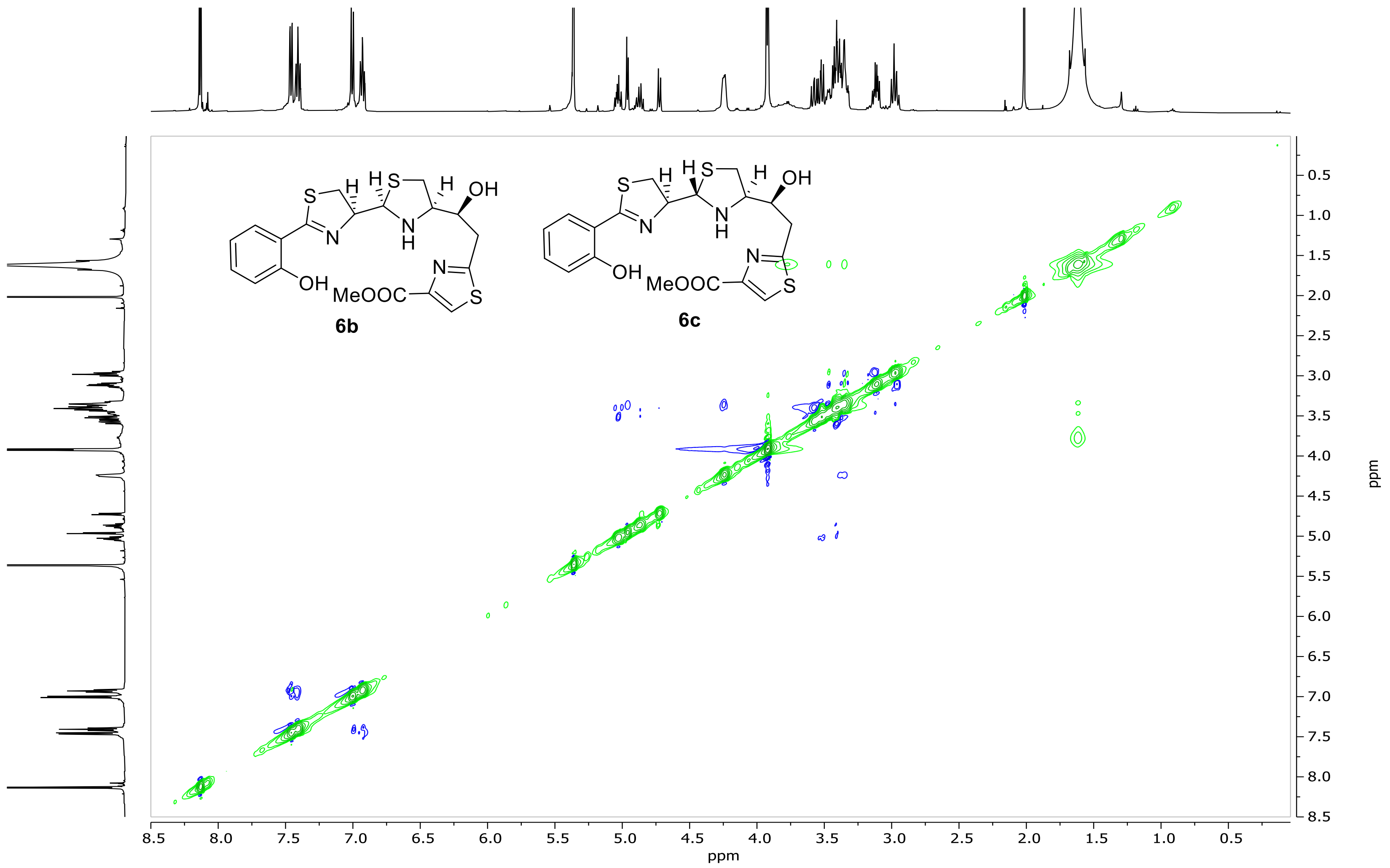

(+)-HRESIMS of a mixture of **6b** and **6c** (3:2): Ion: *m/z*: 452.0774 ($[M+H]^+$)

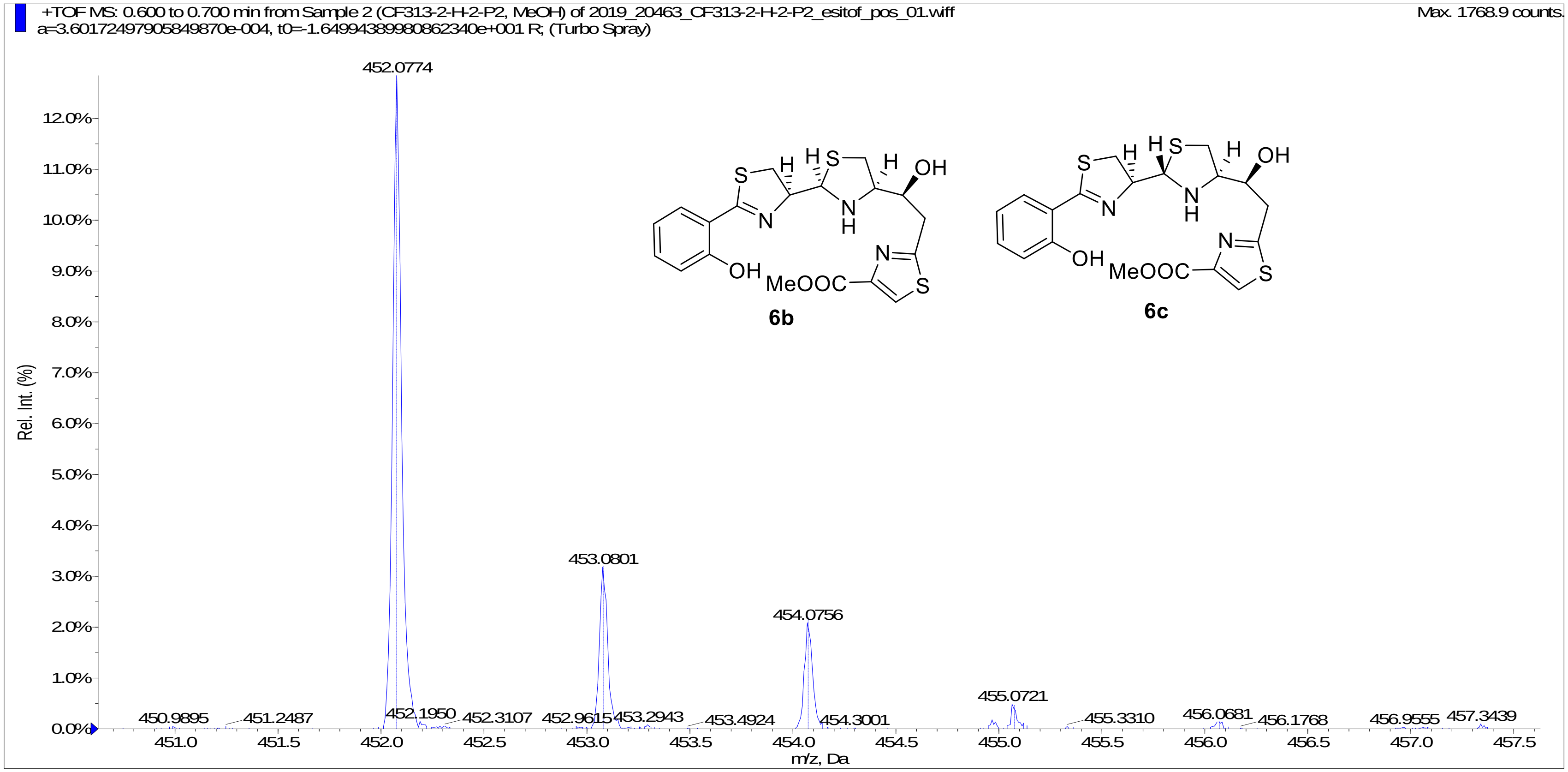

Formula	Calc m/z	Δ, mDa	Δ, ppm	DBE
$C_{19}H_{22}N_3O_4S_3$	452.0766	0.7017	1.5522	10.5

4.4 NMR and MS of 7a and 7d

^{1}H NMR (500.13 MHz, CD_3OD) of **7a** and **7d** (0.6:1)

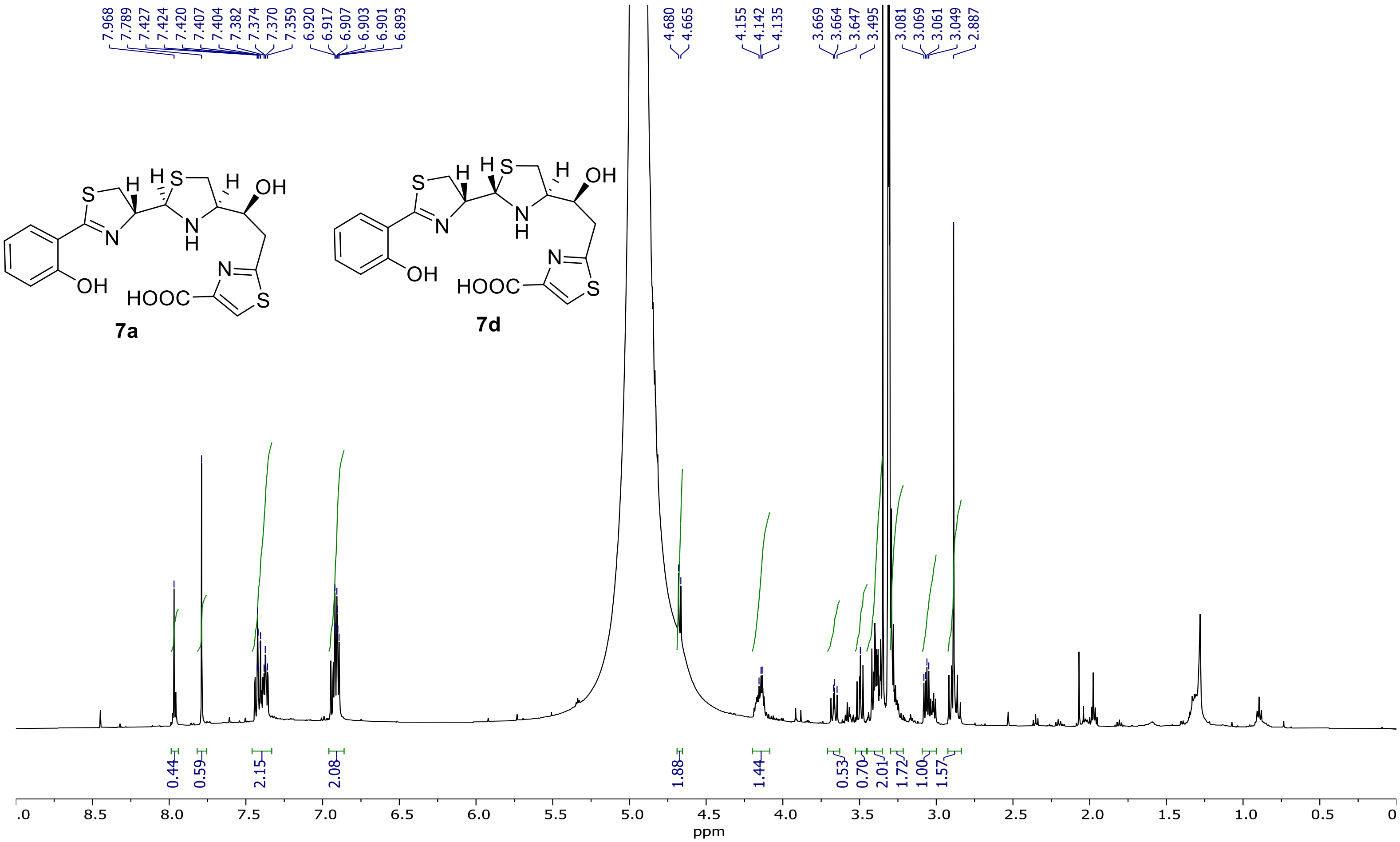

^{13}C NMR (500.13 MHz, CD_3OD) of **7a** and **7d** (0.6:1)

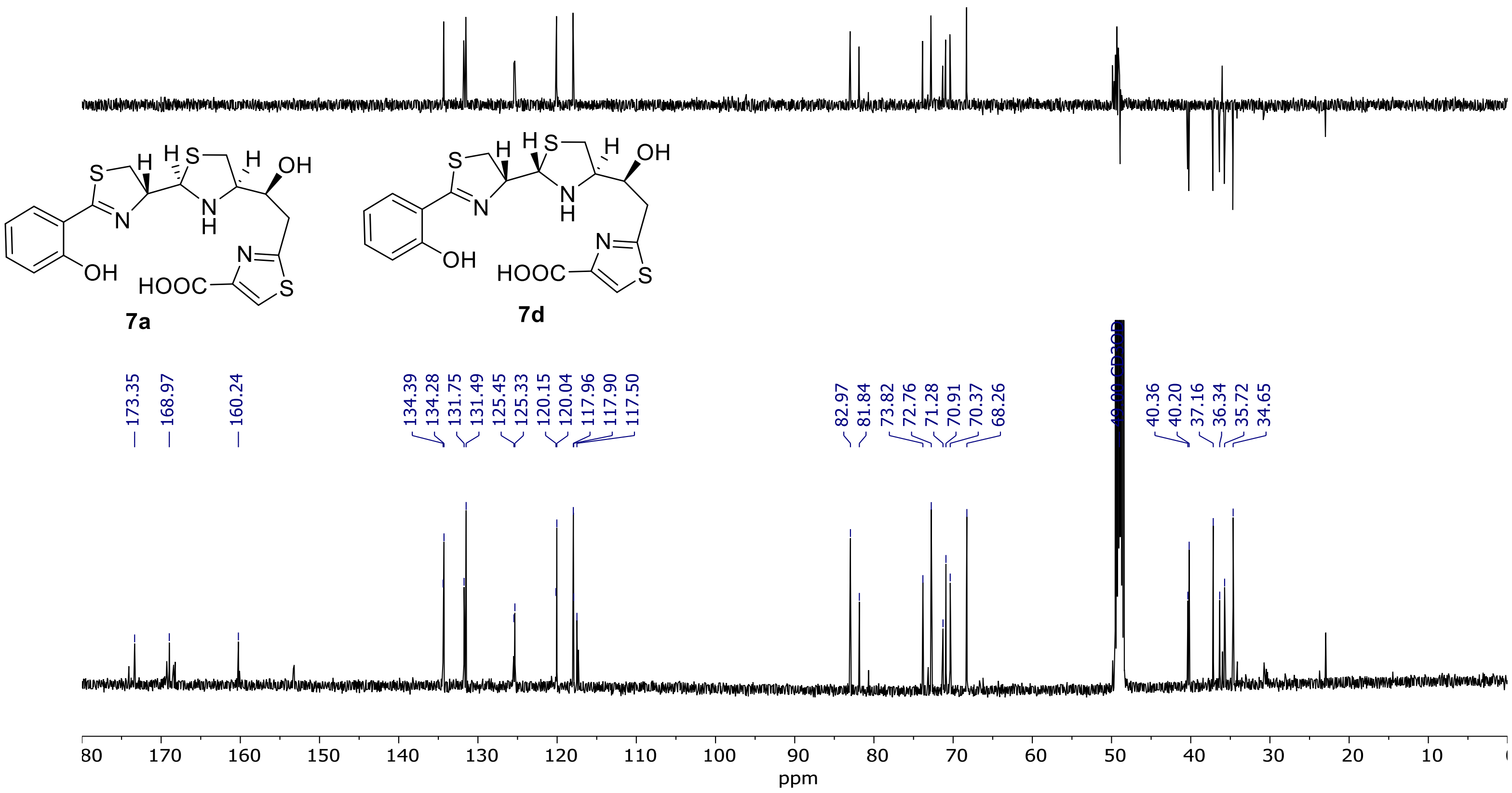

^{1}H-^{1}H COSY (500.13 MHz, CD_3OD) of **7a** and **7d** (0.6:1)

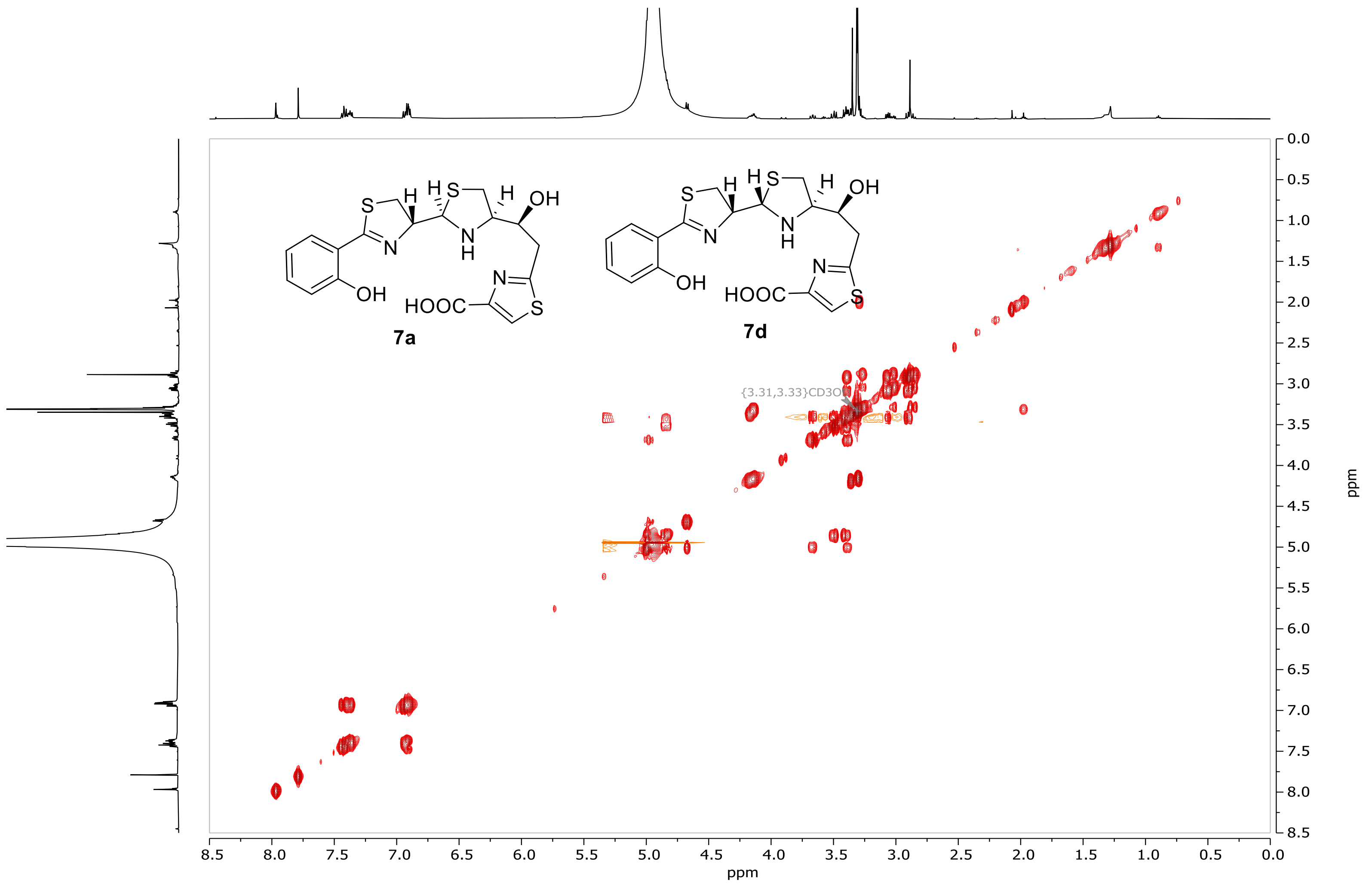

HSQC (500.13 MHz, CD_3OD) of **7a** and **7d** (0.6:1)

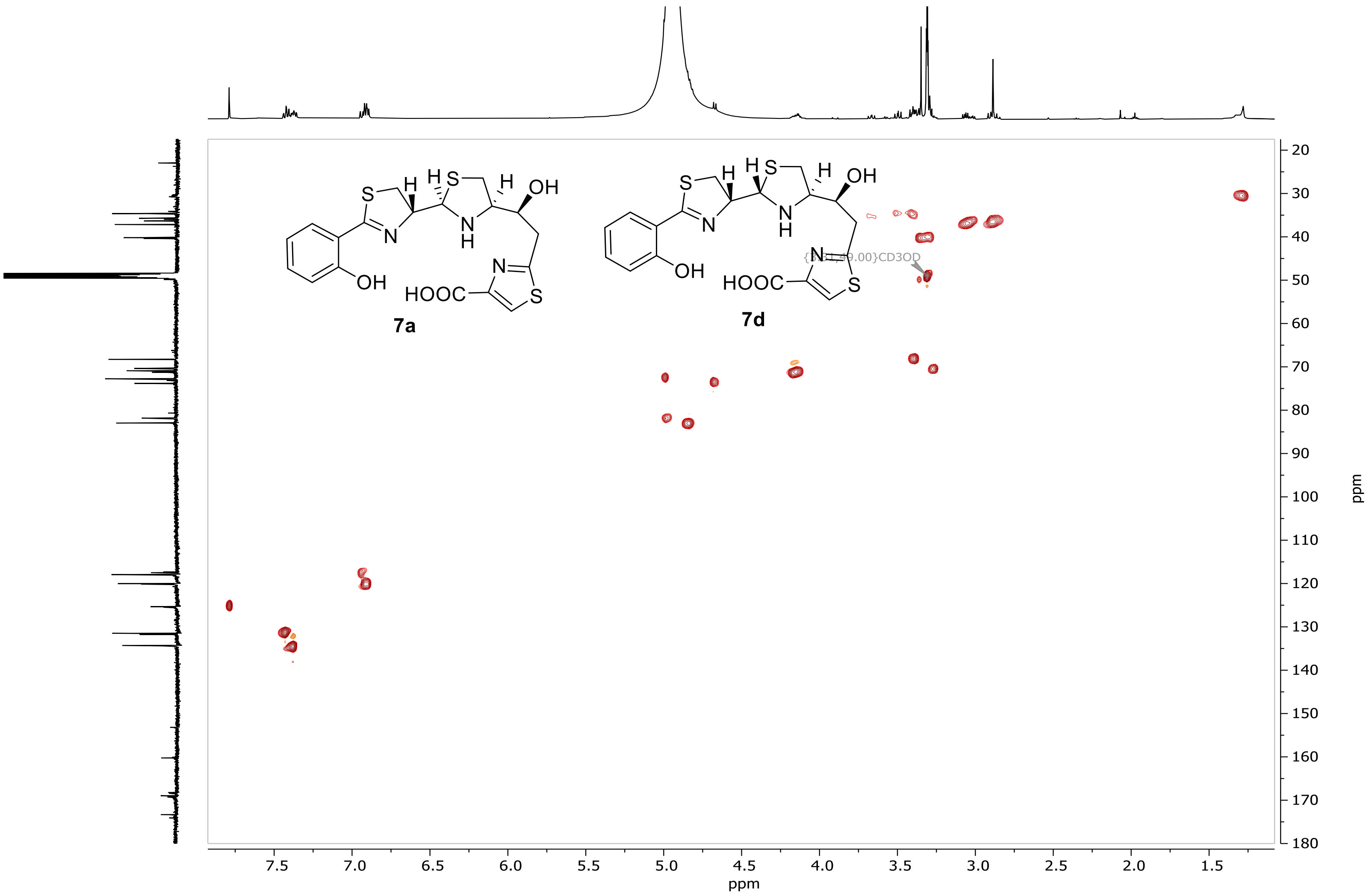

HMBC (500.13 MHz, CD_3OD) of **7a** and **7d** (0.6:1)

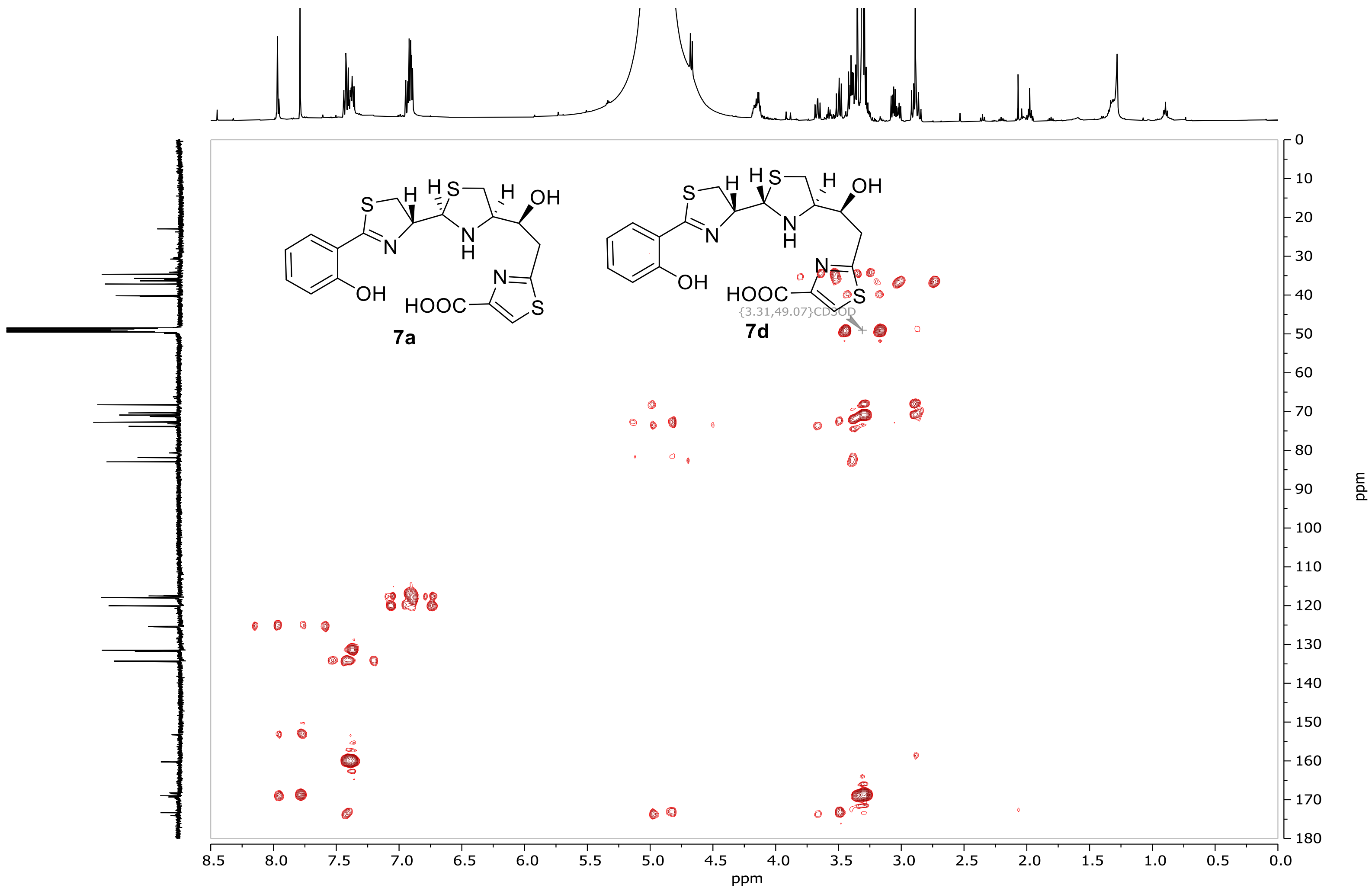

^{1}H-^{1}H NOESY (500.13 MHz, CD_3OD) of **7a** and **7d** (0.6:1)

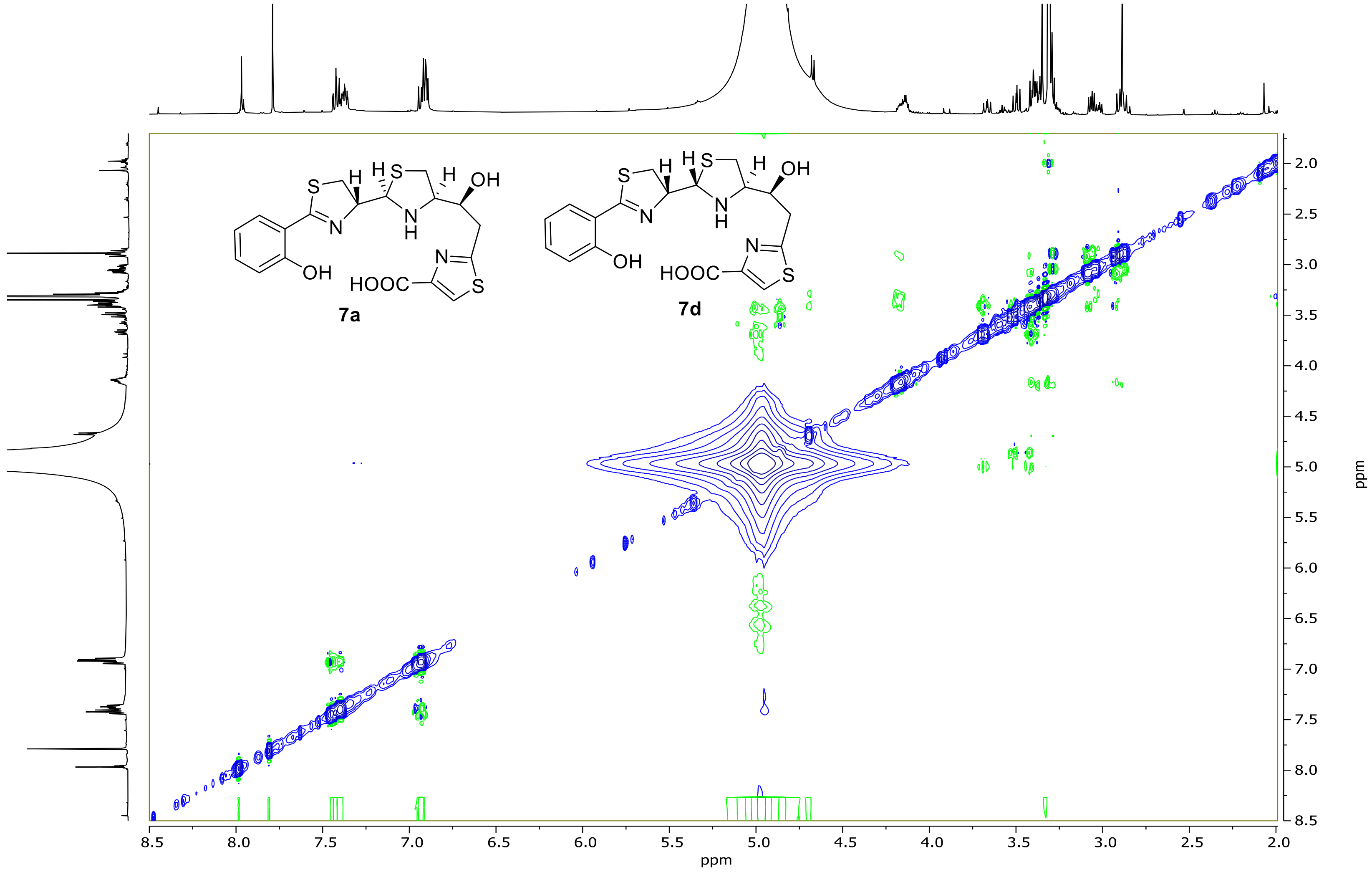

^{1}H-^{1}H ROESY (500.13 MHz, CD_3OD) of **7a** and **7d** (0.6:1)

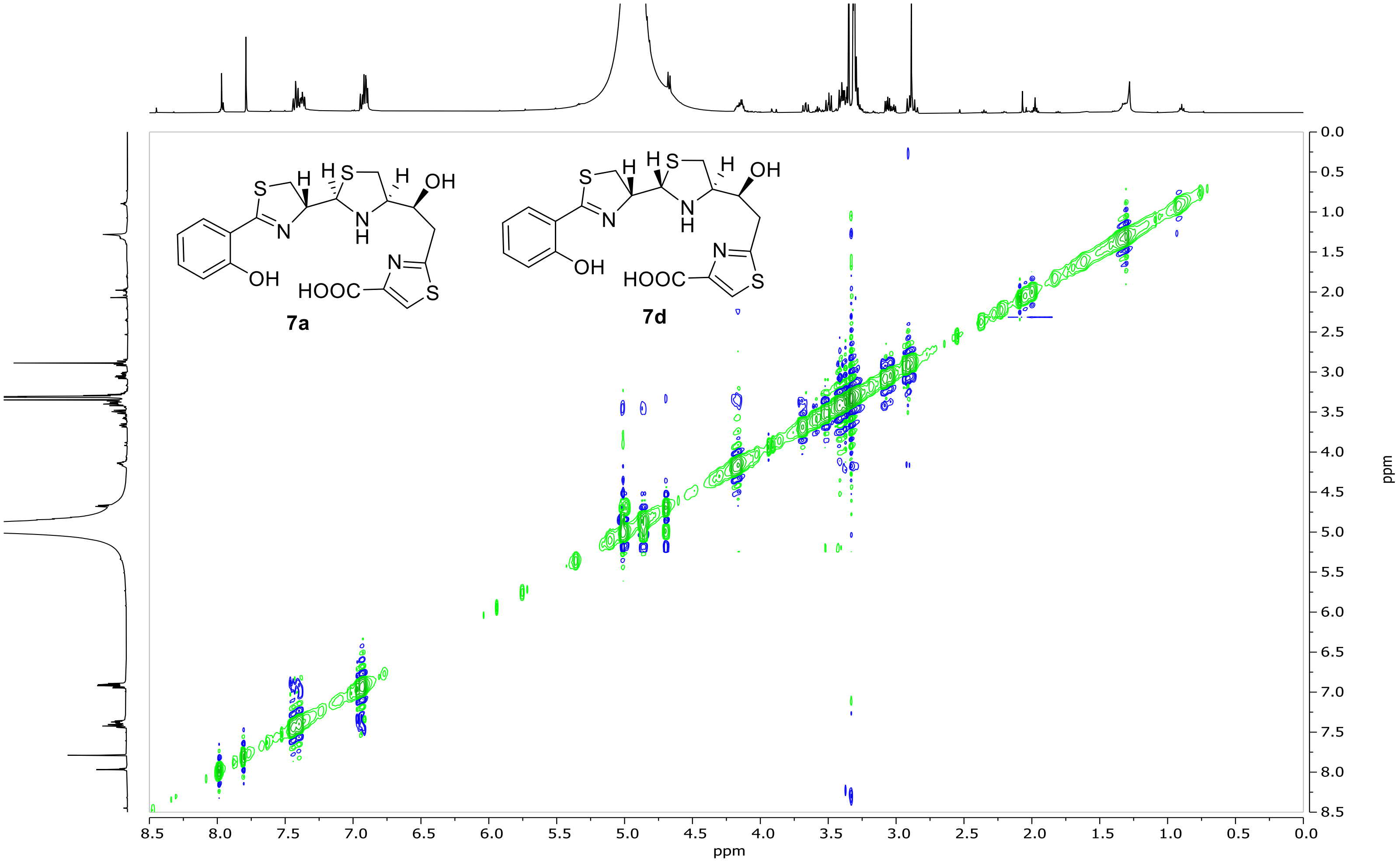

(-)-HRESIMS of **7a** and **7d** Ion: *m/z*: 436.0457 [M-H]$^{-}$

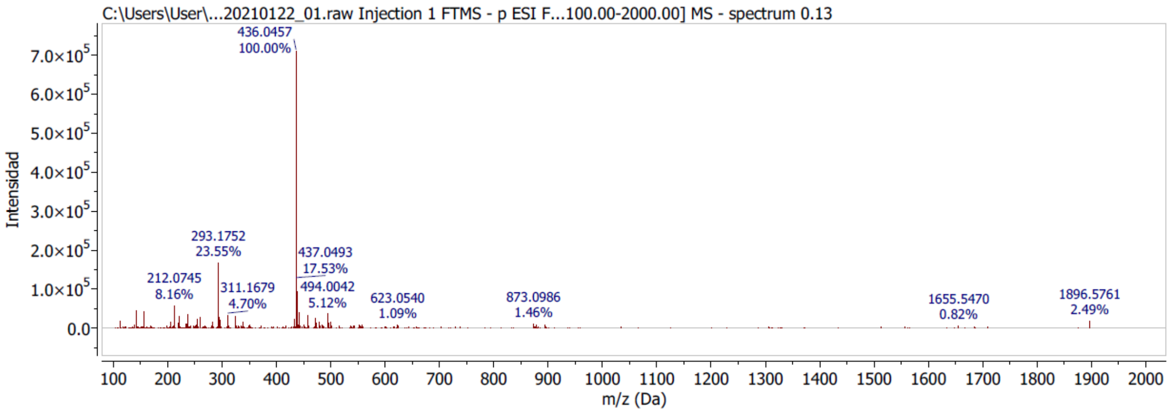

(+)-HRESIMS of **7a** and **7d** Ion: *m/z*: 438.0611 [M-H]$^{+}$

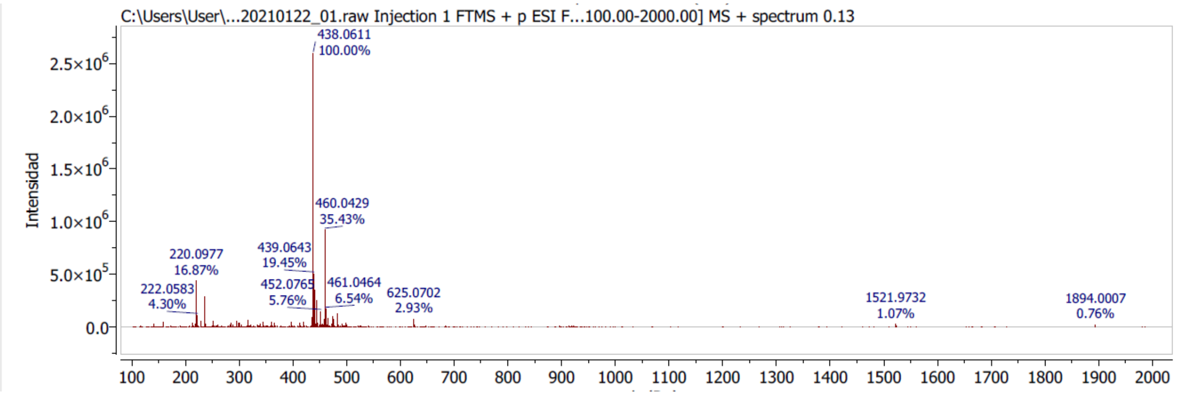

4.5 NMR and MS of a mixture of 7b and 7c (5:1)

Table S4: ^{1}H NMR and ^{13}C NMR chemical shift list of a mixture of **7b**:**7c** (5:1) in CD_3OD

		7b		7c	
C	Mult.	δ_C	δ_H mult (*J* Hz)	δ_C	δ_H mult (*J* Hz)
**1**	C	160.17	-	160.17	-
**2**	CH	117.93	6.94 dd (8.0, 1.0)	117.93	6.93 m
**3**	CH	134.39	7.38 (bt 8.0)	134.26	7.39 m
**4**	CH	120.11	6.91 t (8.0)	120.04	6.70 m
**5**	CH	131.66	7.43 dd (8.0, 1.4)	131.54	7.43 dd (8.0,1.4)
**6**	C	117.35	-	117.88	-
**7**	C	174.13	-	174.13	-
**8**	CH_2	34.11	*h*: 3.42 dd, (10.9, 9.2)	35.95	*h*:3.43. m
			l: 3.56 dd, (10.9, 8.8)		*l*: 3.59 dd (10.9, 8.4)
**9**	CH	80.67	5.11 m	83.78	4.79 m
**10**	CH	73.16	4.99 u.s.	72.99	4.67 d (9.1)
**11**	CH_2	36.39	*h* : 2.87 t (9.9)	37.31	*h*: 2.85 m
			l : 3.03 dd (9.9, 5.9)		*l*: 3.05 m
**12**	CH	70.36	3.33 u.s.	68.28	3.39 m
**13**	CH	71.31	4.16 dt (8.3, 4.3, 4.3)	70.77	4.11 m
**14**	CH_2	40.36	*h* : 3.30 m	40.39	*h*: 2.85 m
			l : 3.38 m		*l*: 3.04 m
**15**	C	168.45	-	168.45	-
**16**	CH	125.35	7.95 s	125.35	7.97 s
**17**	C	153.40	-	153.40	-
**18**	C	169.25		169.25	-

^{1}H NMR (500.13 MHz, CD_3OD) of a mixture of **7b** and **7c** (5:1)

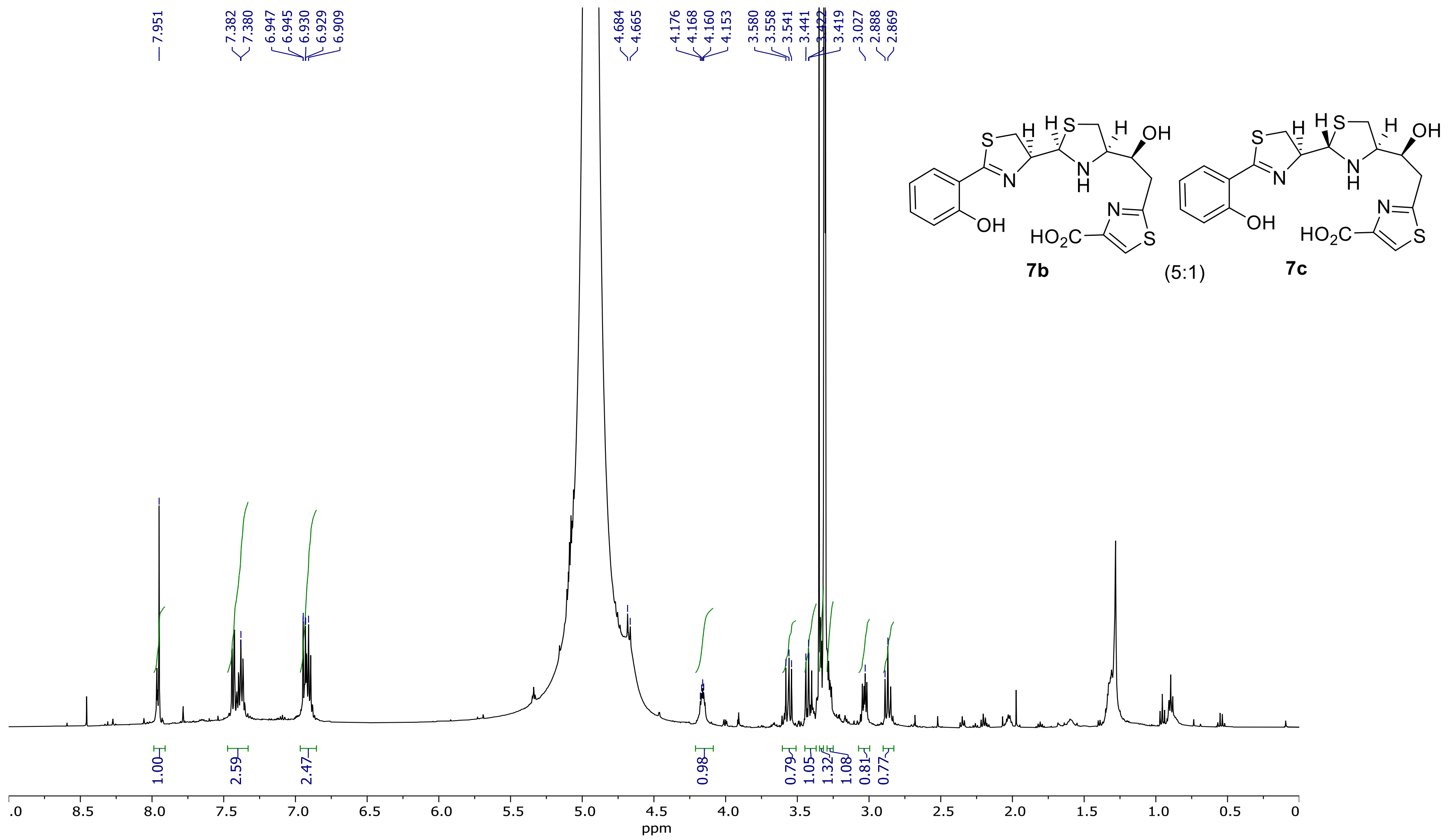

^{13}C NMR (125 MHz, CD_3OD) of a mixture of **7b** and **7c** (5:1)

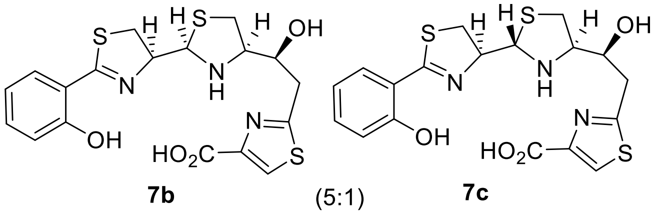

7b (5:1) **7c**

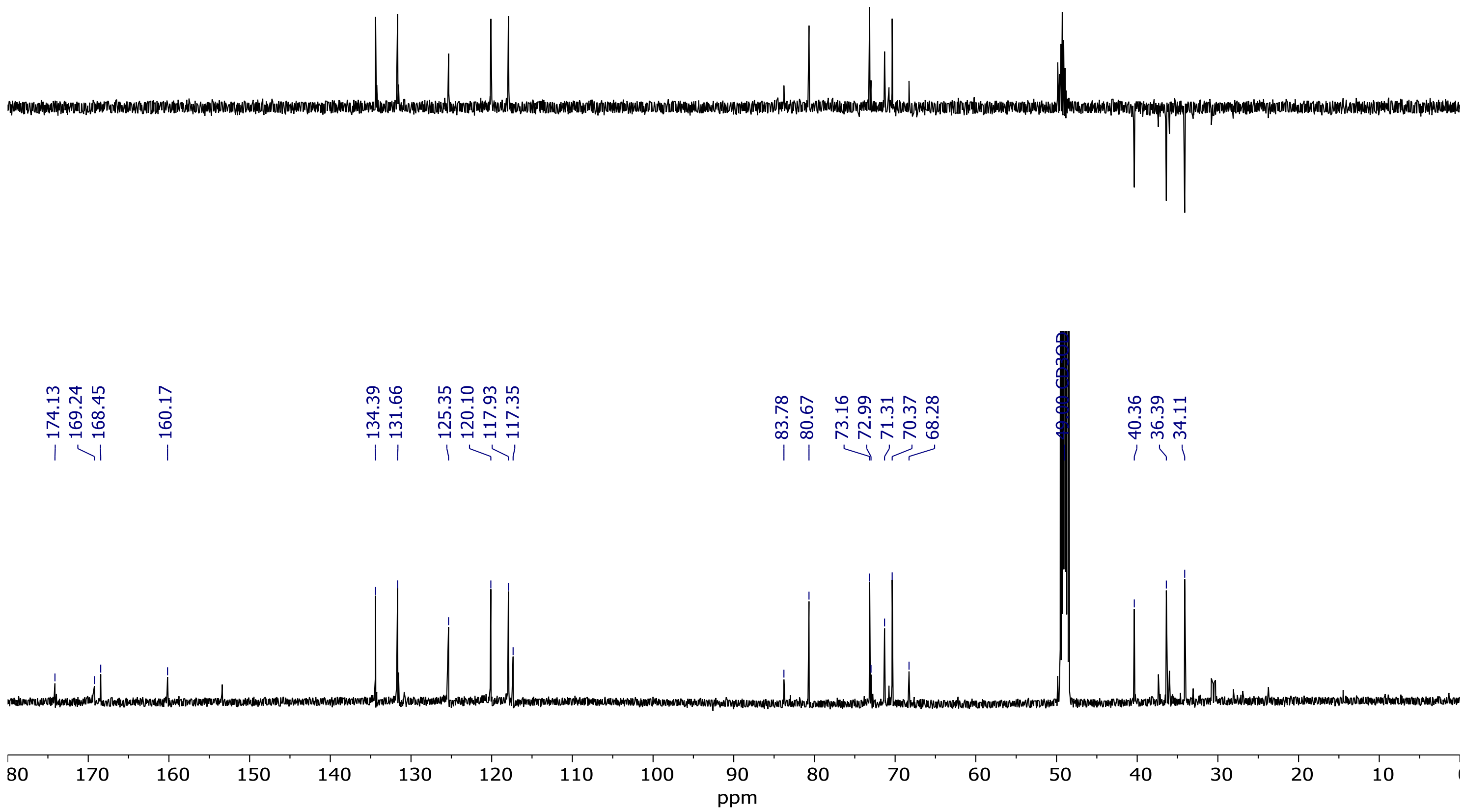

^{1}H-^{1}H COSY (500.13 MHz, CD_3OD) of a mixture of **7b** and **7c** (5:1)

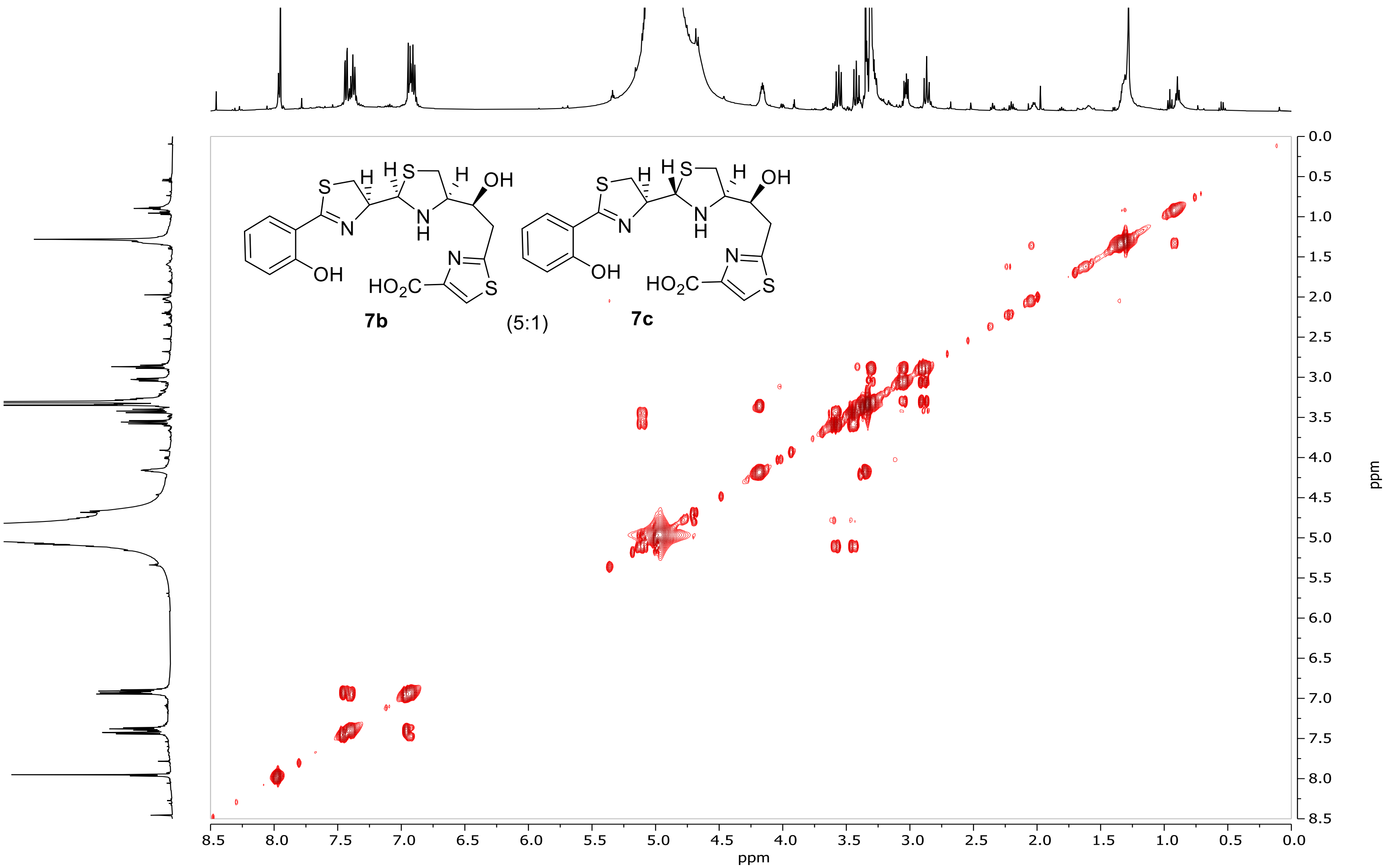

HSQC (500.13 MHz, CD_3OD) of a mixture of **7b** and **7c** (5:1)

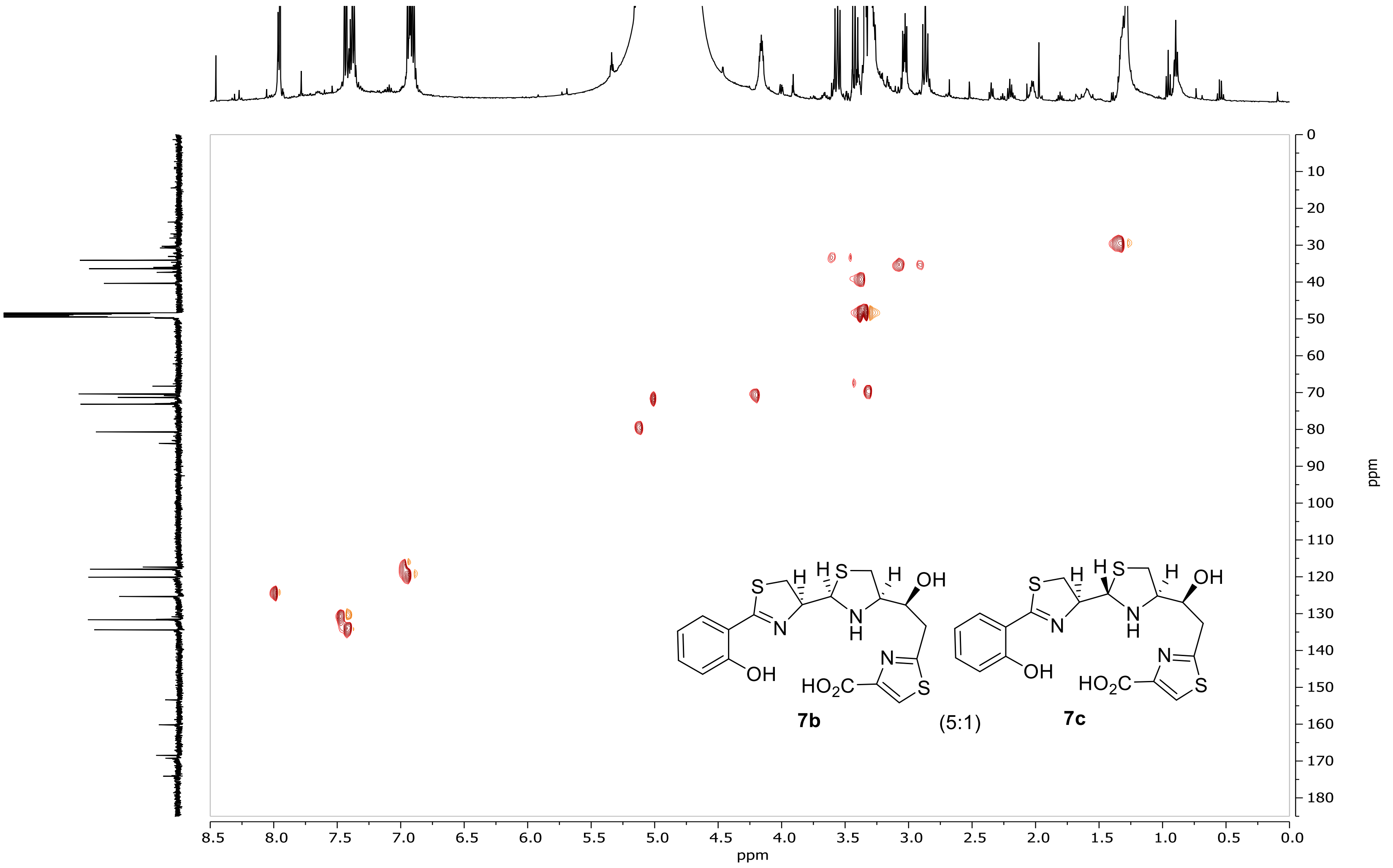

HMBC (500.13 MHz, CD_3OD) of a mixture of **7b** and **7c** (5:1)

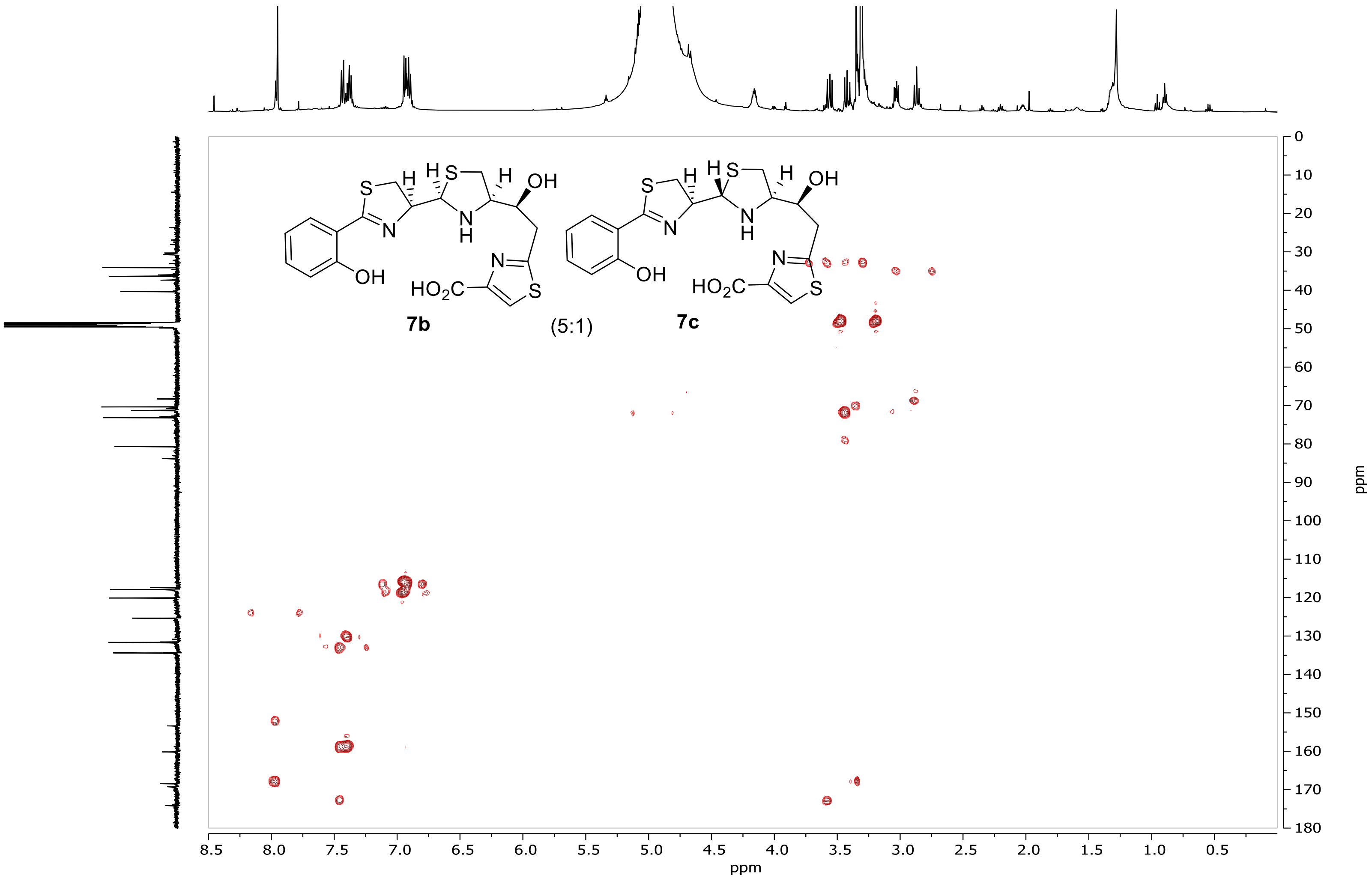

^{1}H-^{1}H NOESY (500.13 MHz, CD_3OD) of a mixture of **7b** and **7c** (5:1)

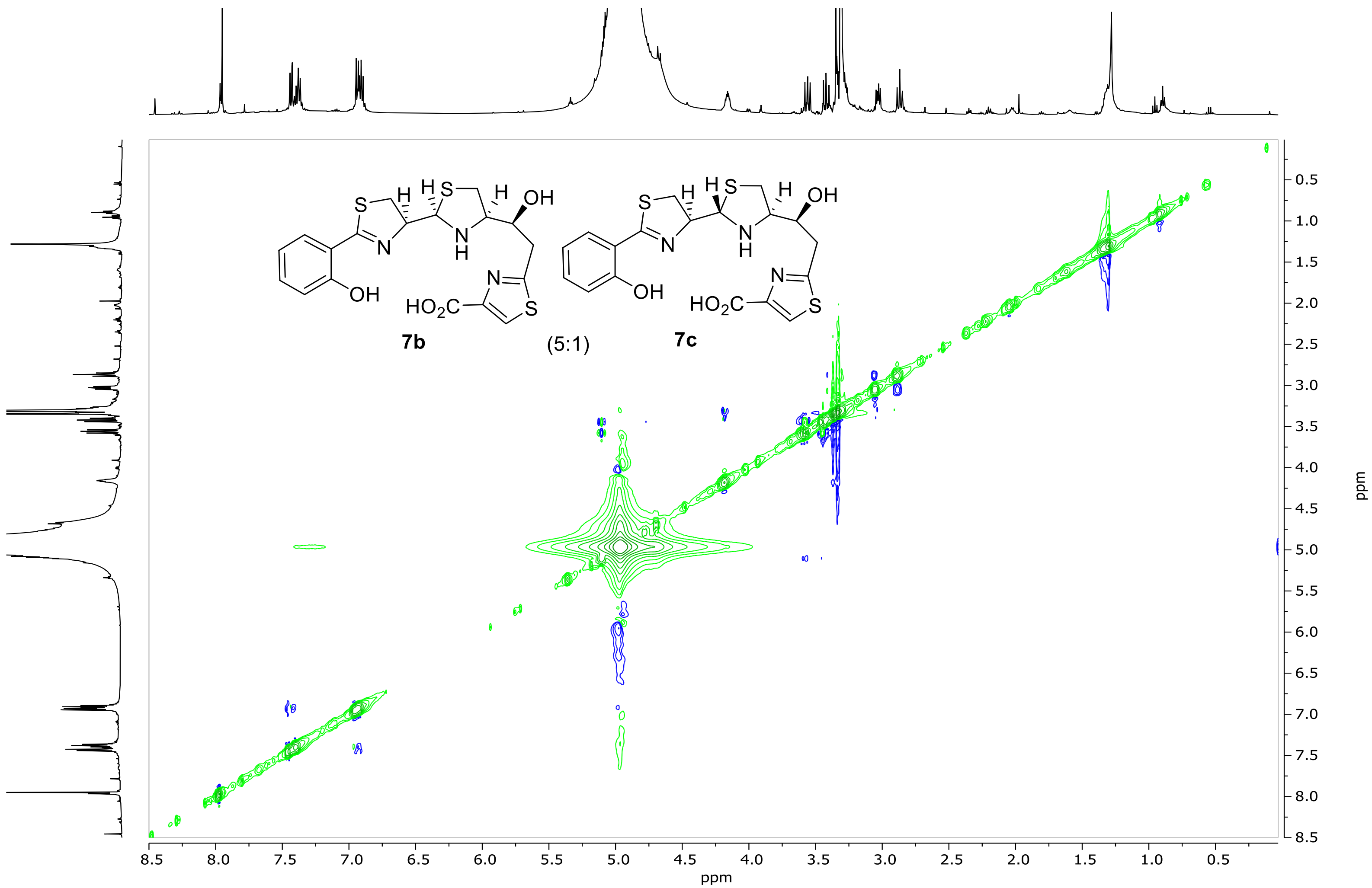

^{1}H-^{1}H ROESY (500.13 MHz, CD_3OD) of a mixture of **7b** and **7c** (5:1)

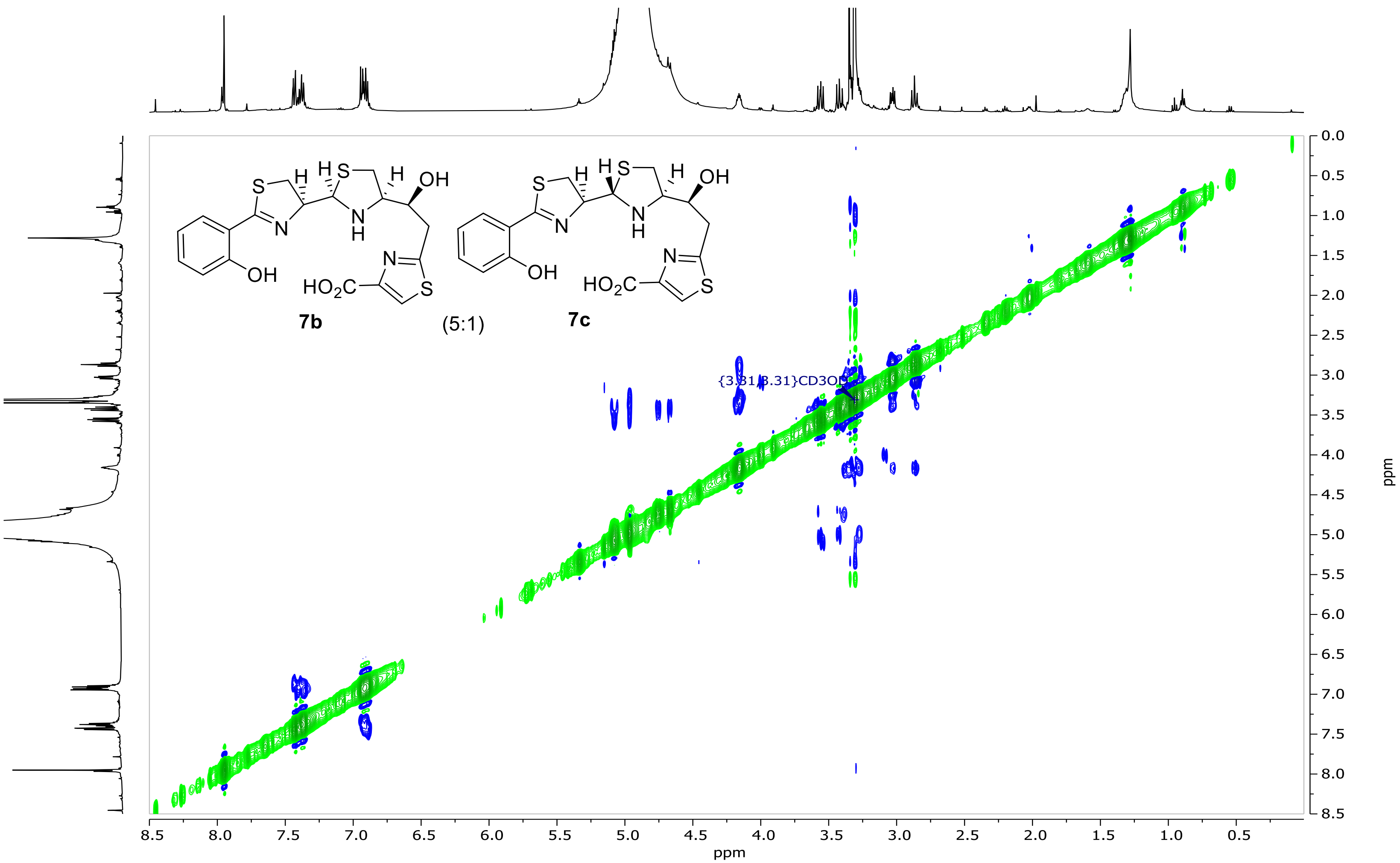

(-)-HRESIMS of a mixture of **7b** and **7c** (5:1): Ion: *m/z*: 436.0456 [M-H]$^-$

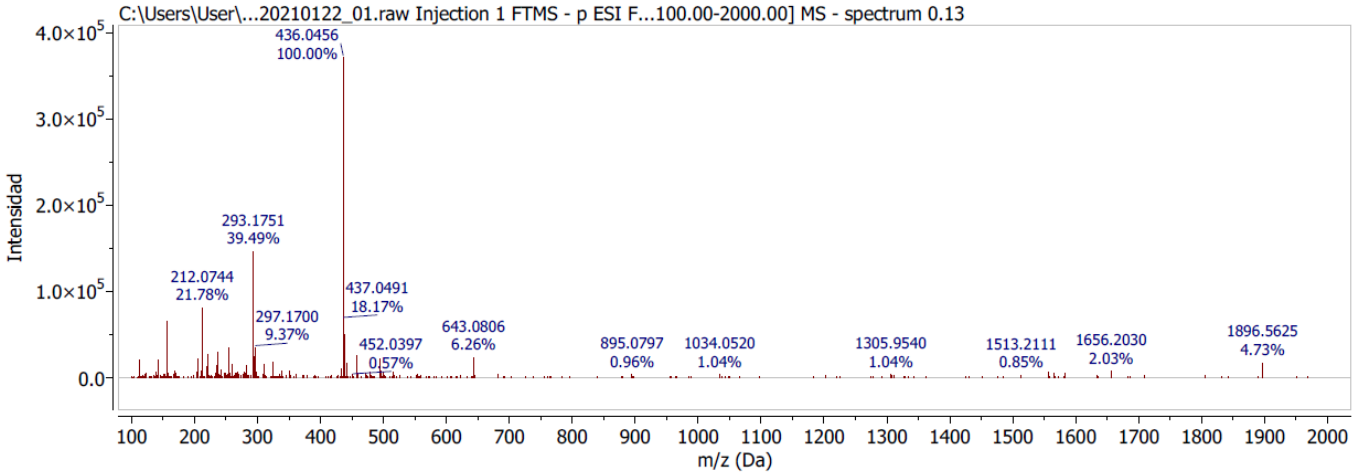

(+)-HRESIMS of a mixture of **7b:7c** (5:1): Ion: *m/z*: 438.0610 [M+H]$^+$

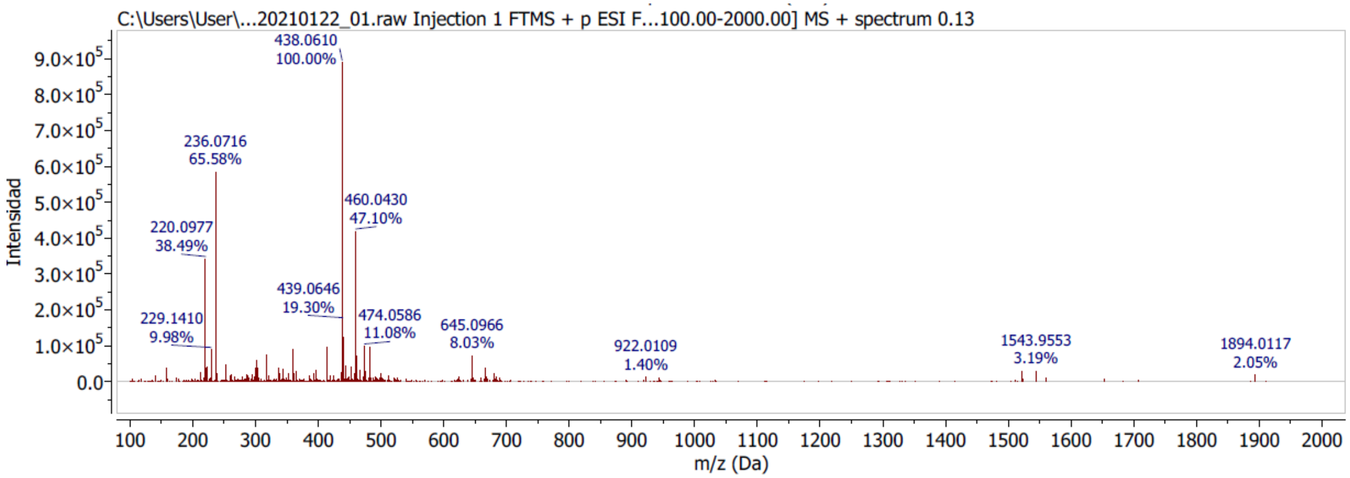

4.6 NMR and MS of a mixture of 7a-d

^{1}H NMR (500.13 MHz, CD_3OD) of **7a-d**

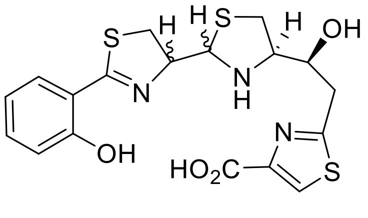

7a-d

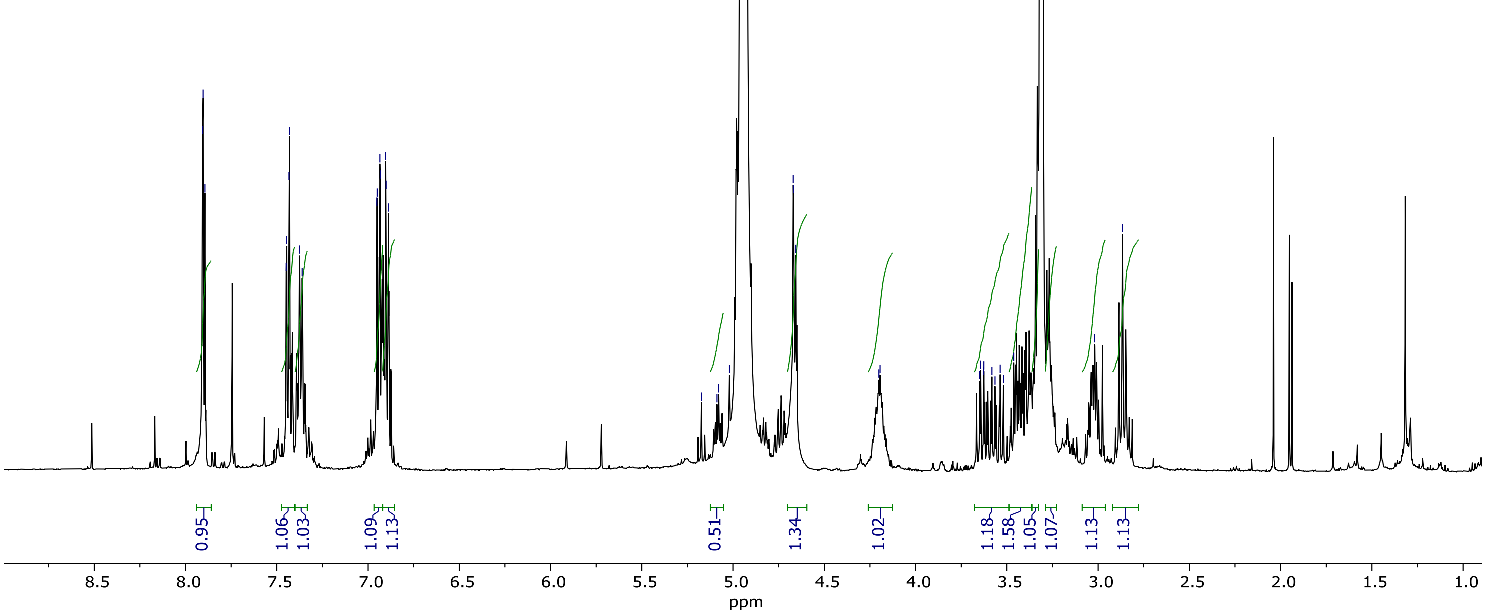

^{13}C NMR (125.13 MHz, CD_3COD) of **7a-d**

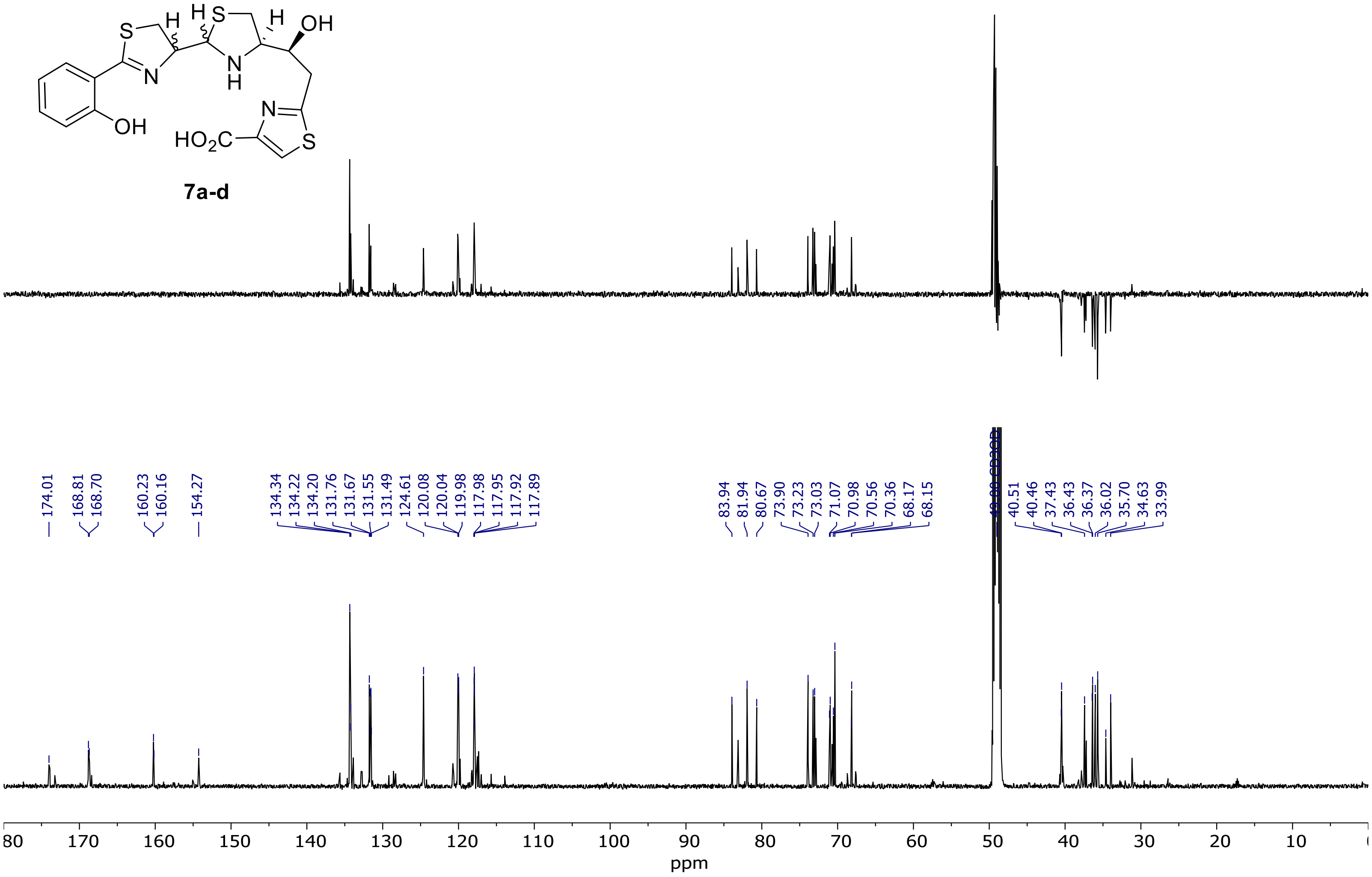

^{1}H-^{1}H COSY (500.13 MHz, CD_3OD) of **7a-d**

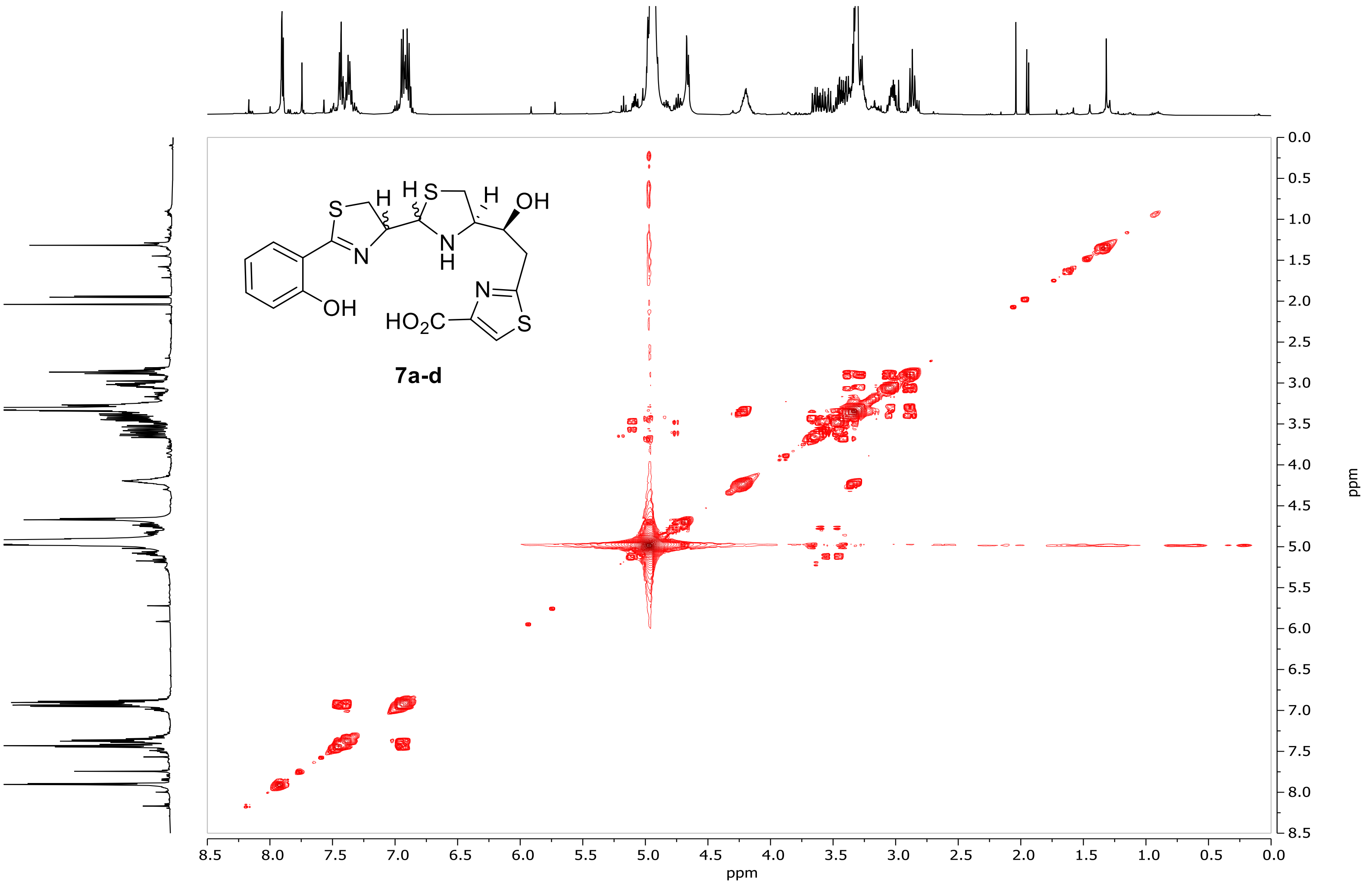

HSQC (500.13 MHz, CD_2Cl_2) of **7a-d**

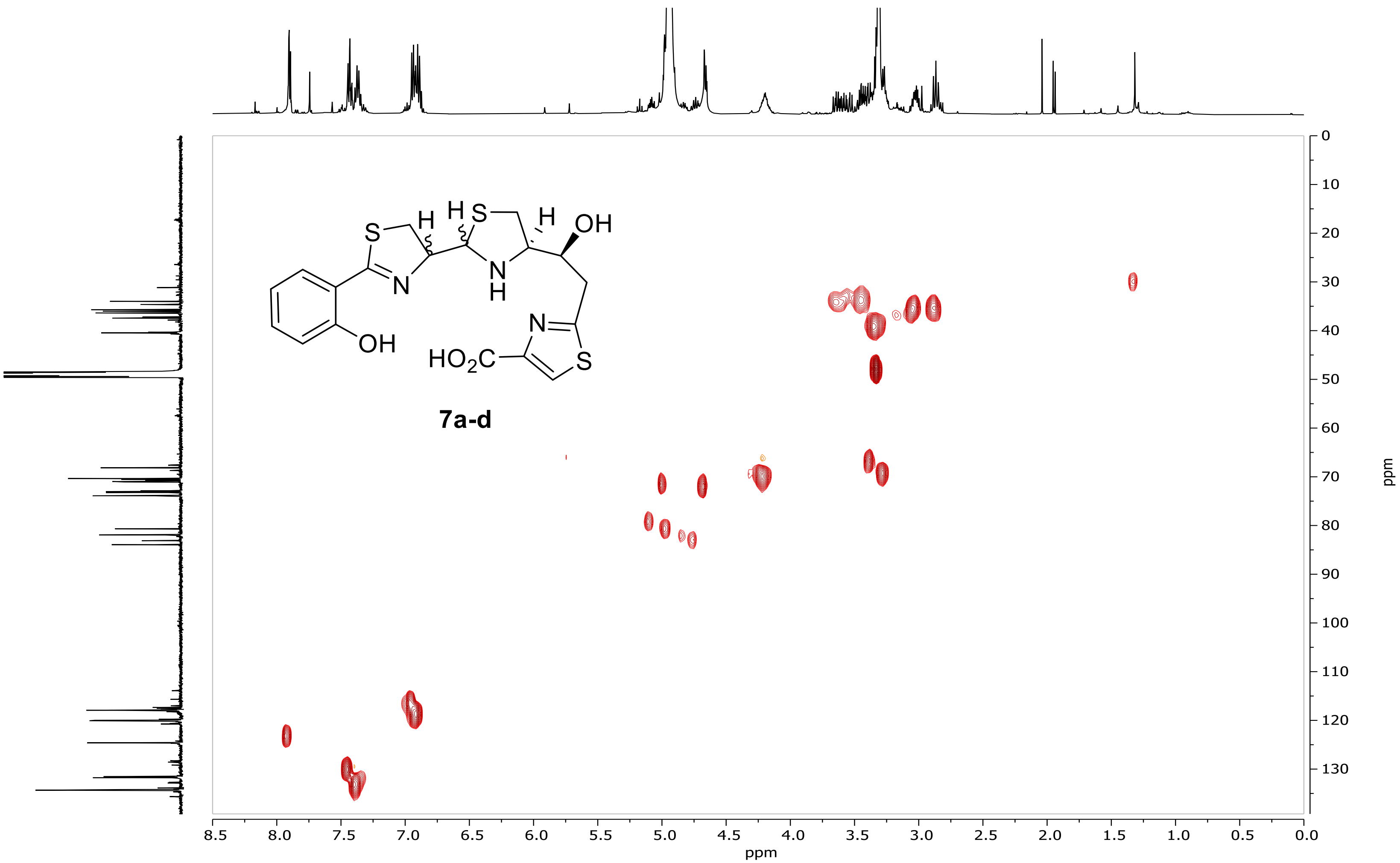

HMBC (500.13 MHz, CD_2Cl_2) of **7a-d**

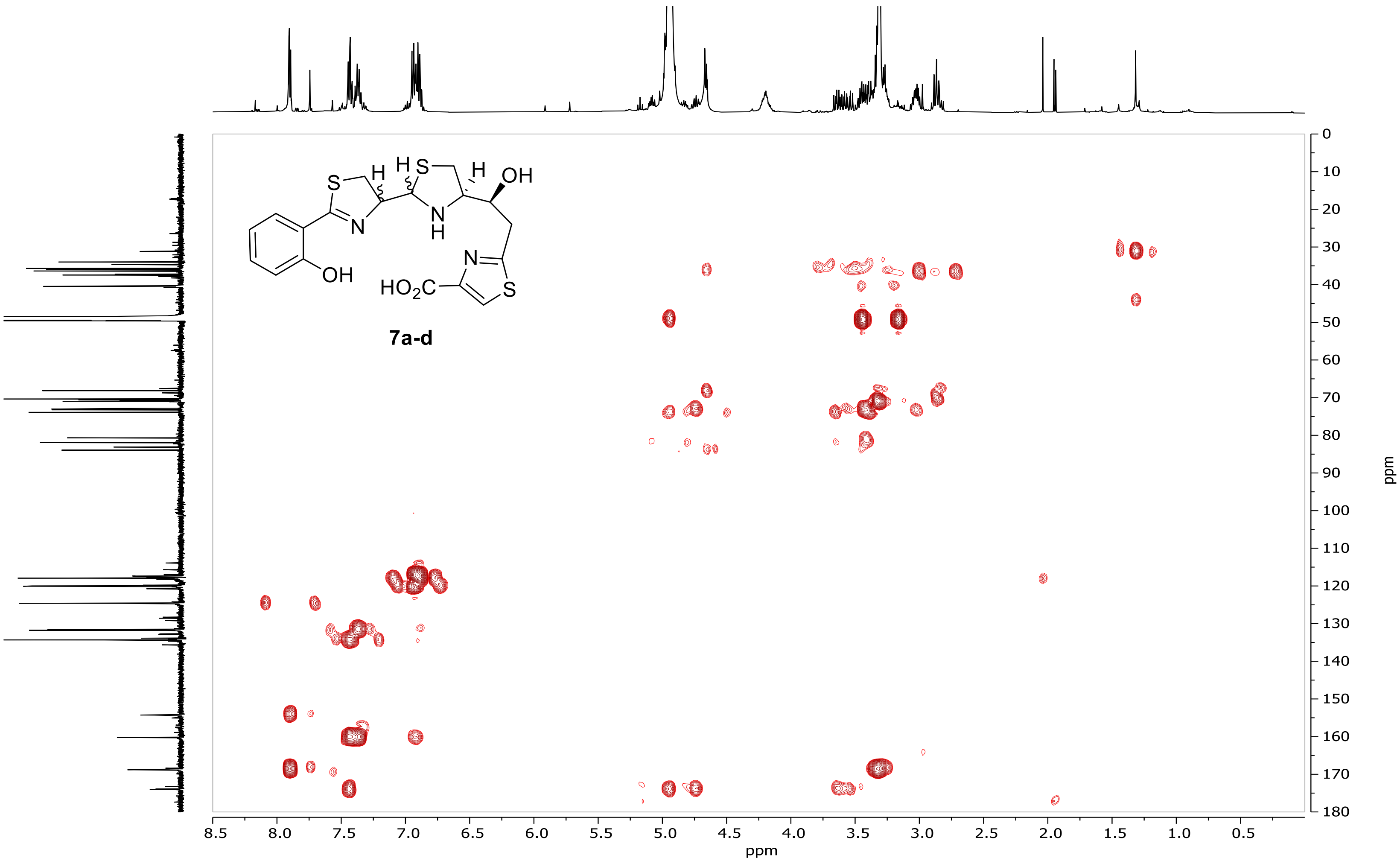

UV spectrum of **7a-d**

DAD1, 9.803 (80.5 mAU,Apx) Ref=6.383 of CF316-14.D

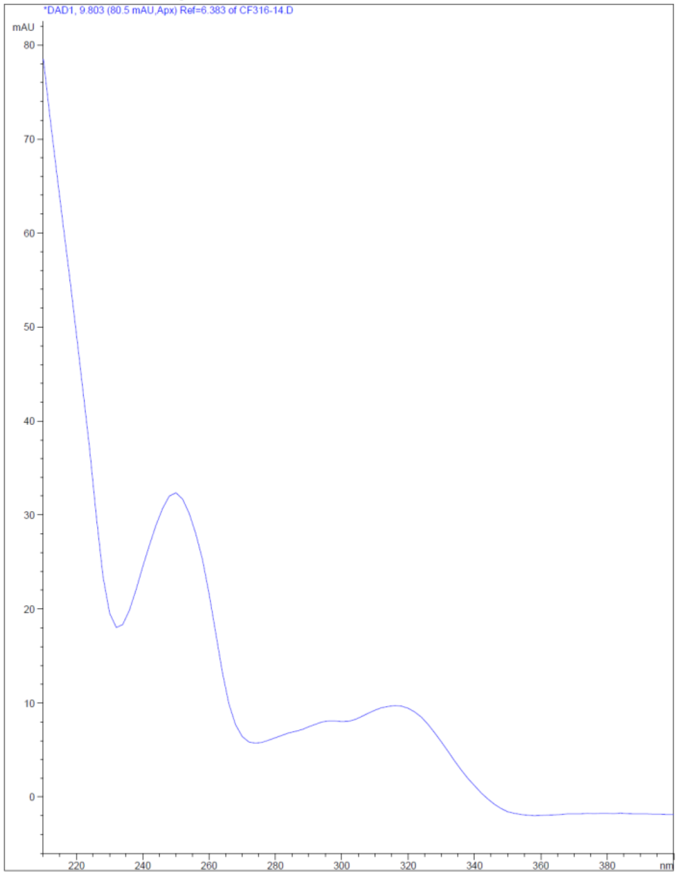

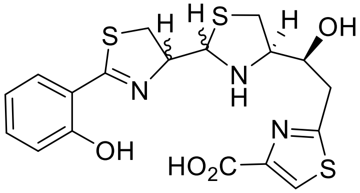

7a-d

(-)-HRESIMS of **7a-d**: Ion: *m/z*: 436.0465 [M-H]⁻

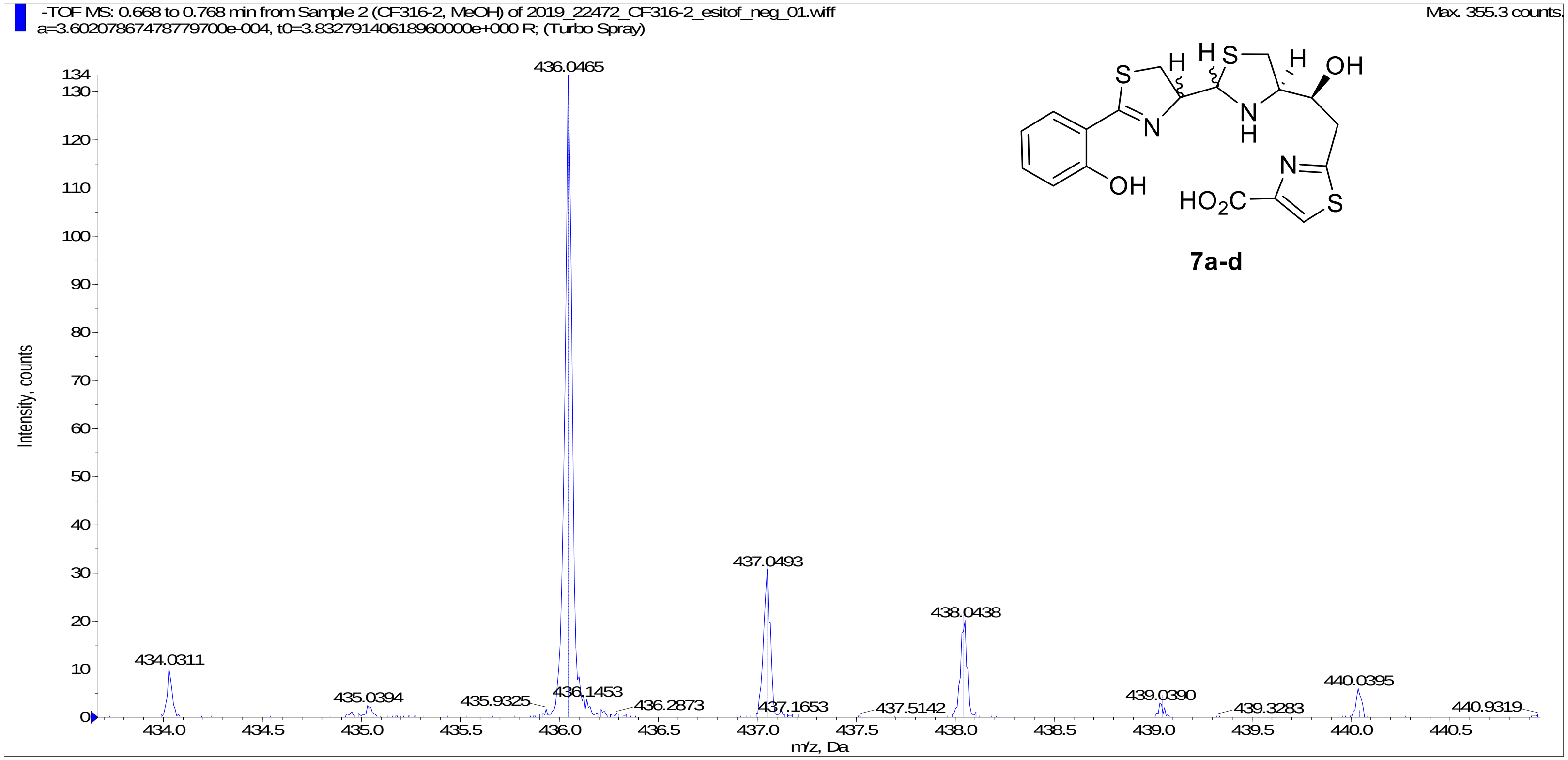

Formula	Calc m/z	Δ, mDa	Δ, ppm	DBE
$C_{18}H_{18}N_3O_4S_3$	436.0464	0.0047	0.0108	11.5

4.7 NMR and MS of compound of Ga^{+3} complex 32a

^{1}H NMR (500.13 MHz, CD_3OD) of **32**

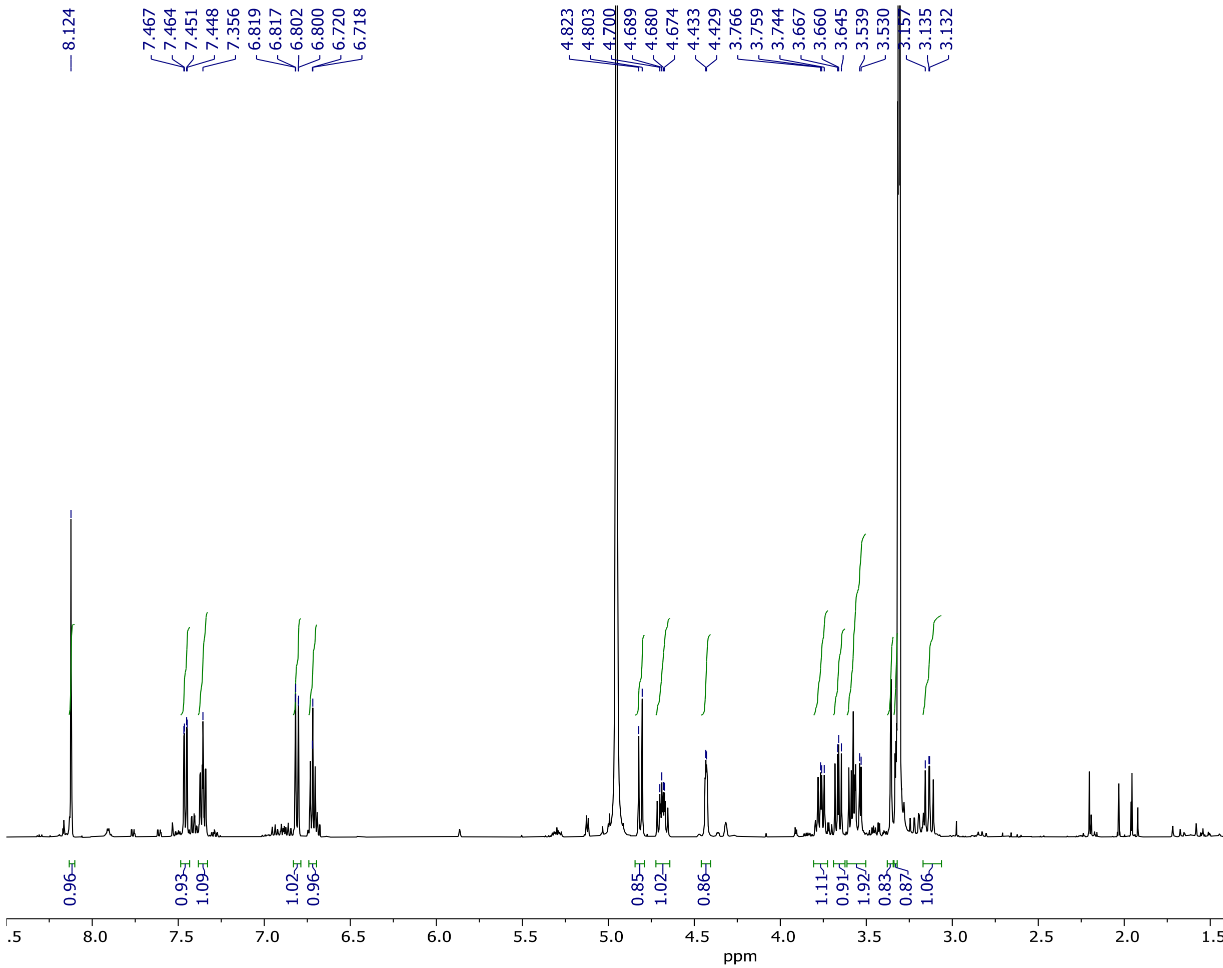

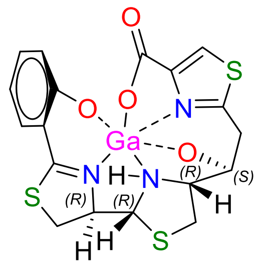

32a

^{13}C NMR (125.13 MHz, CD_3OD) of **32a**

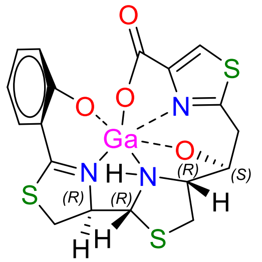

32a

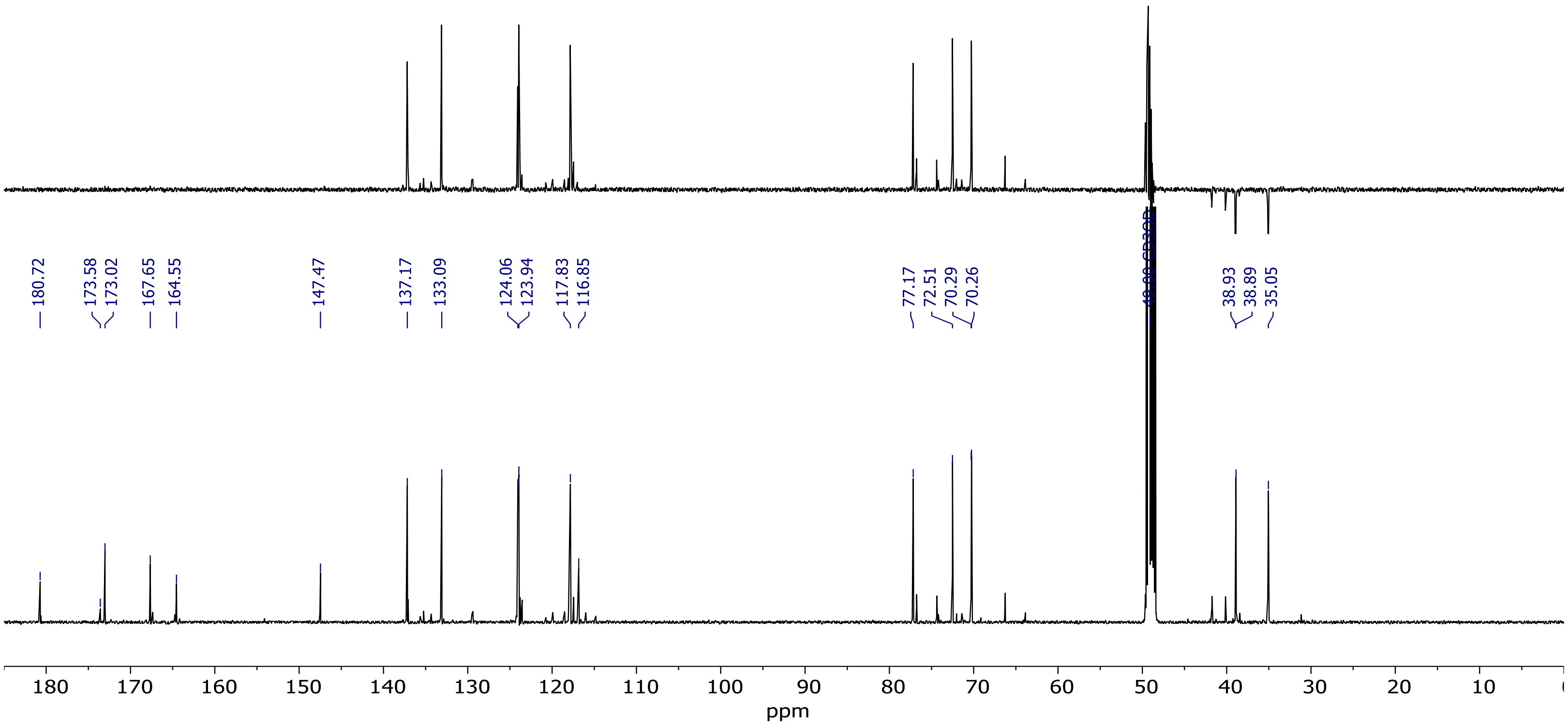

^{1}H-^{1}H COSY (125.13 MHz, CD_3OD) of **32a**

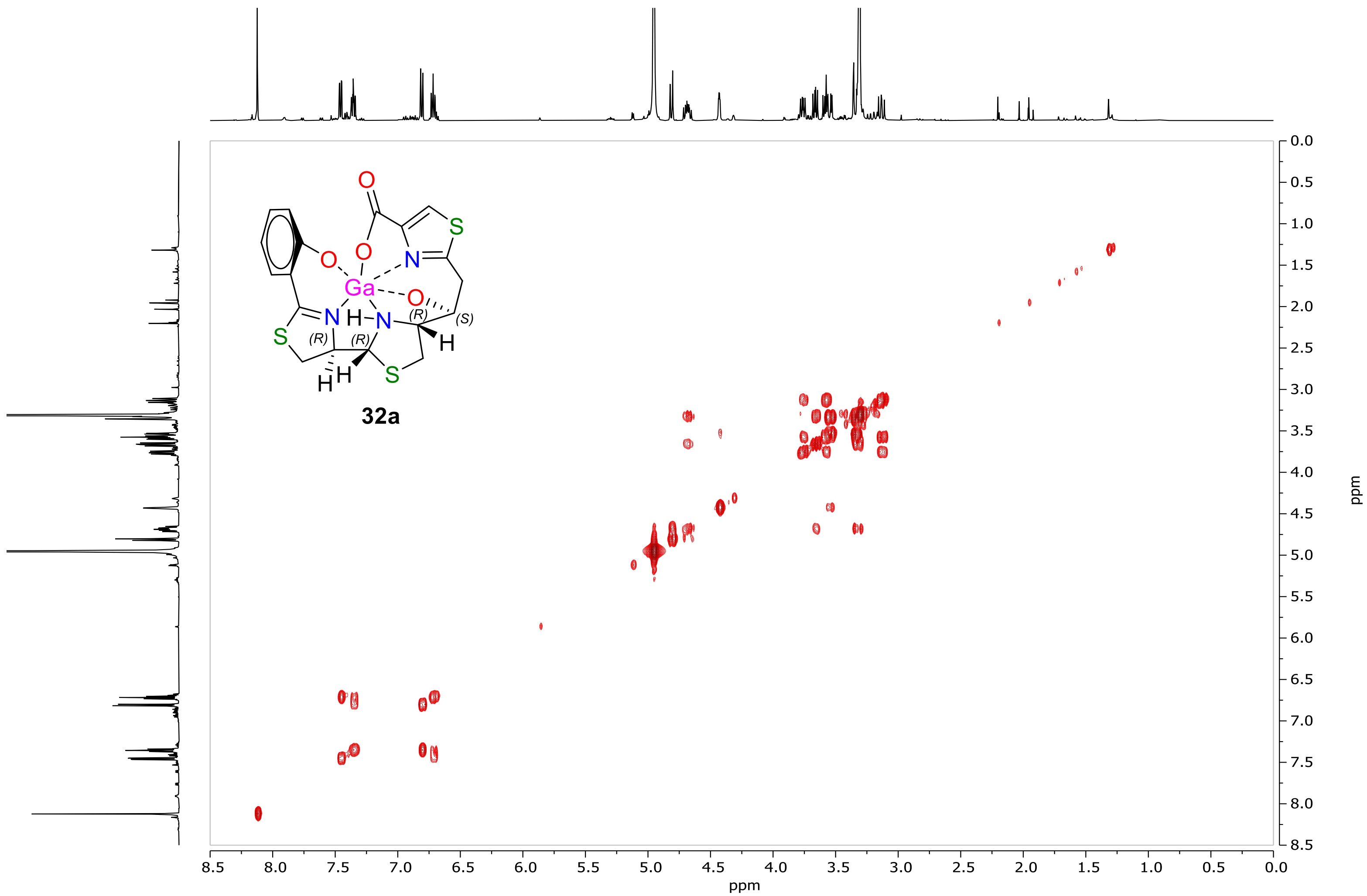

HSQC (500.13 MHz, CD_3OD) of **32a**

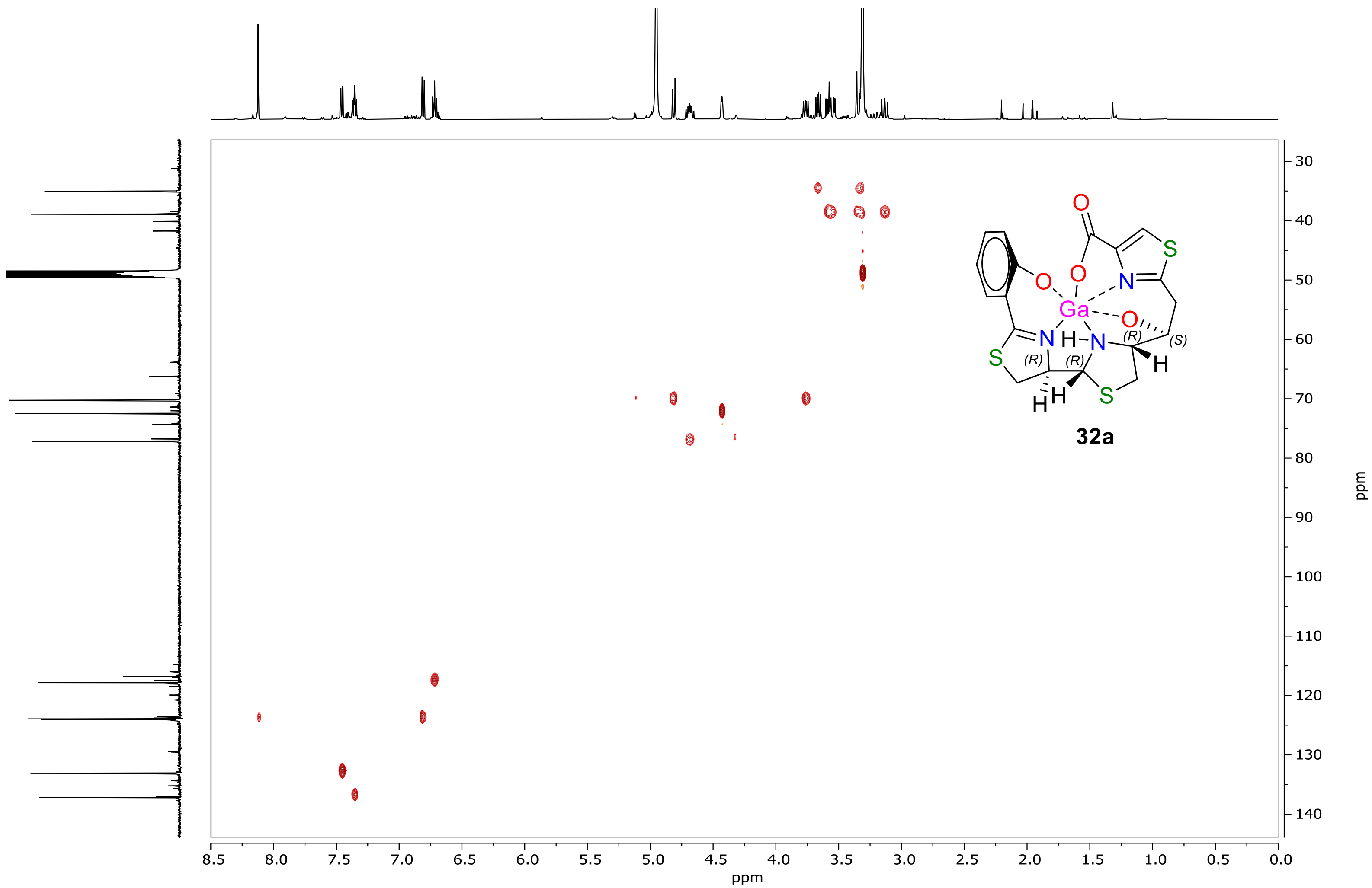

HMBC (500.13 MHz, CD_3OD) of **32a**

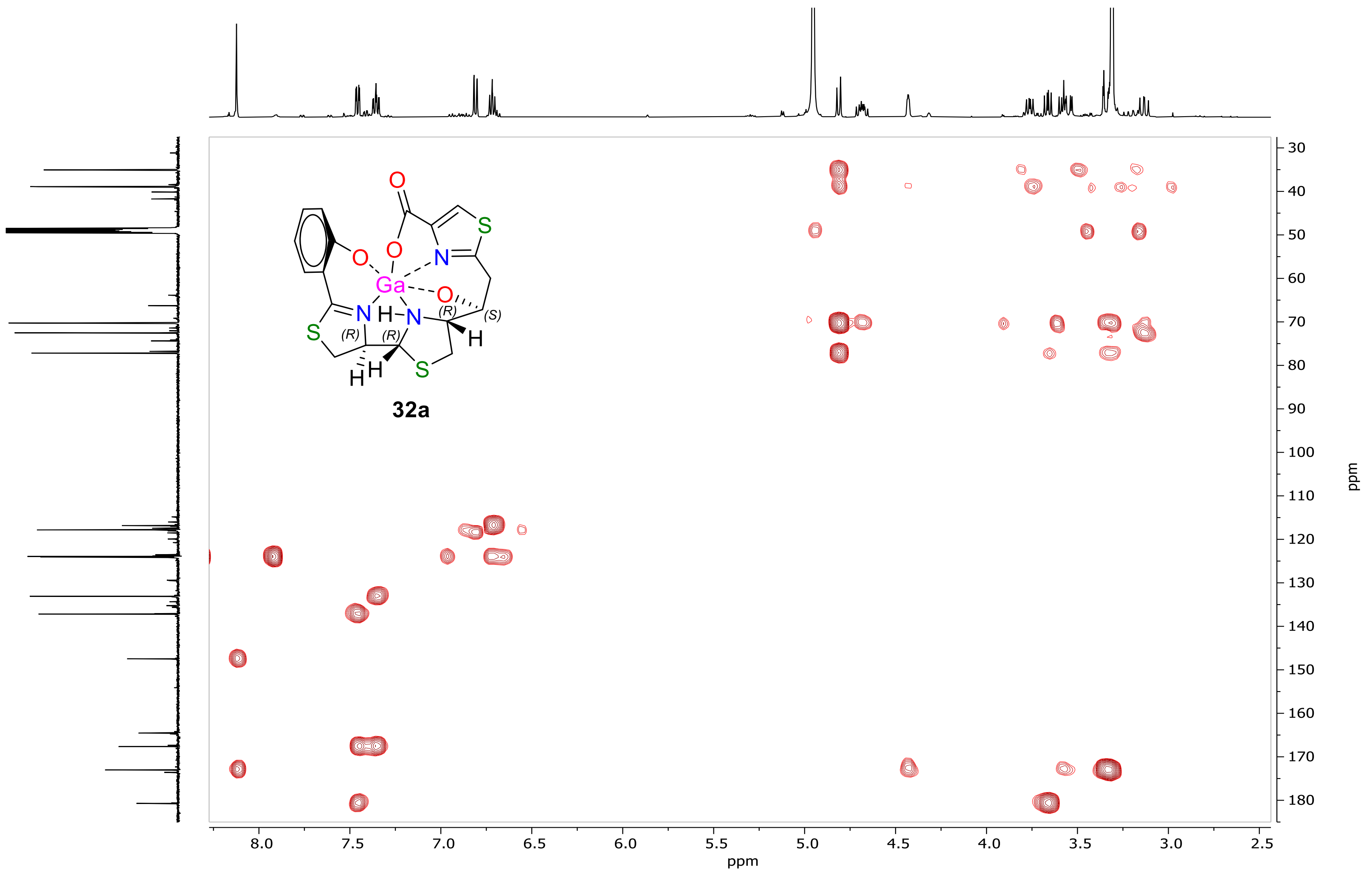

^1H-^1H NOESY of **32a** (CD_3OD)

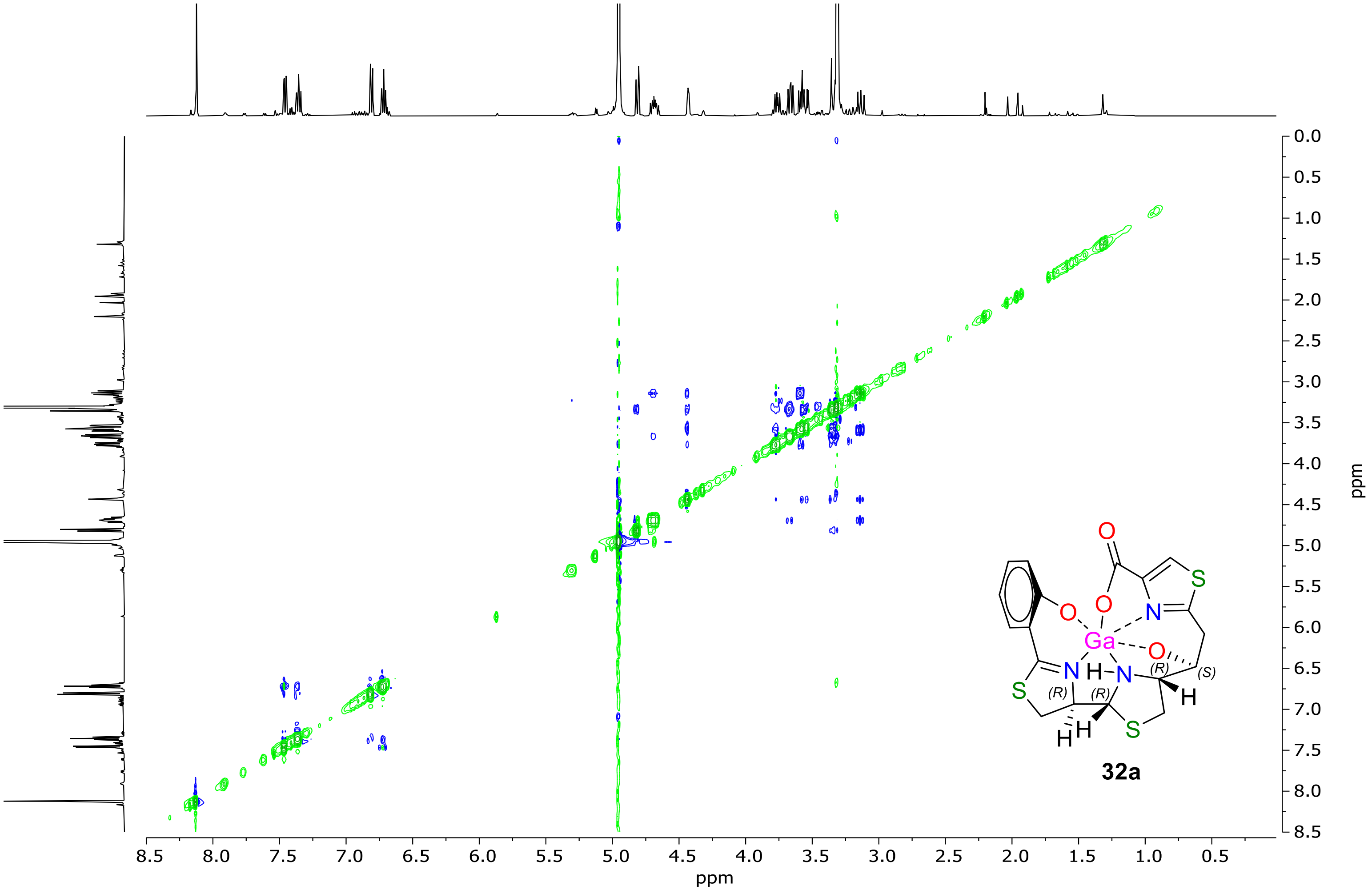

UV spectrum of **32a**

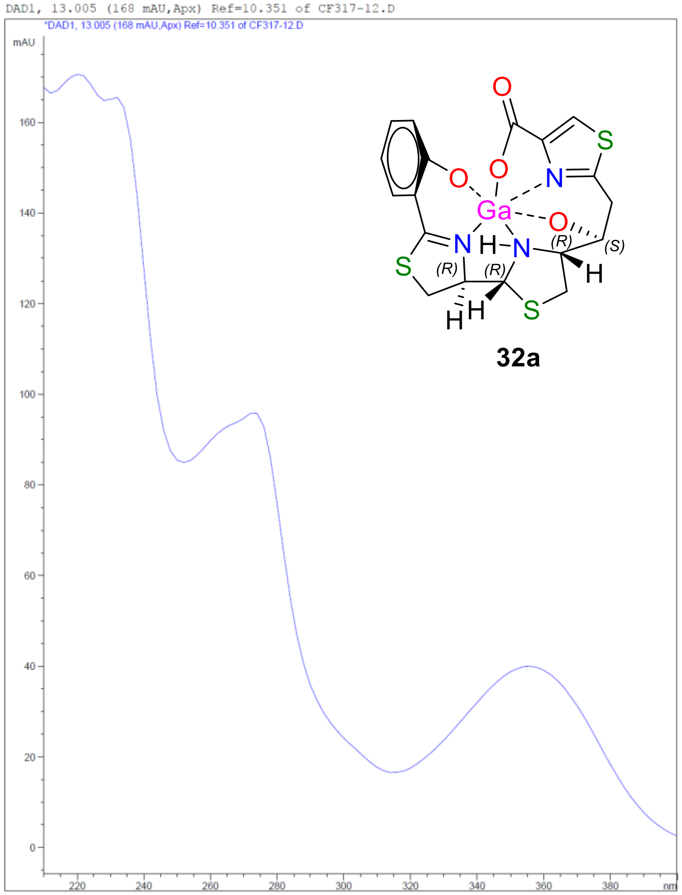

(-)-HRESIMS of **32a**: Ion: *m/z*: 501.9485 ([M-H]$^-$)

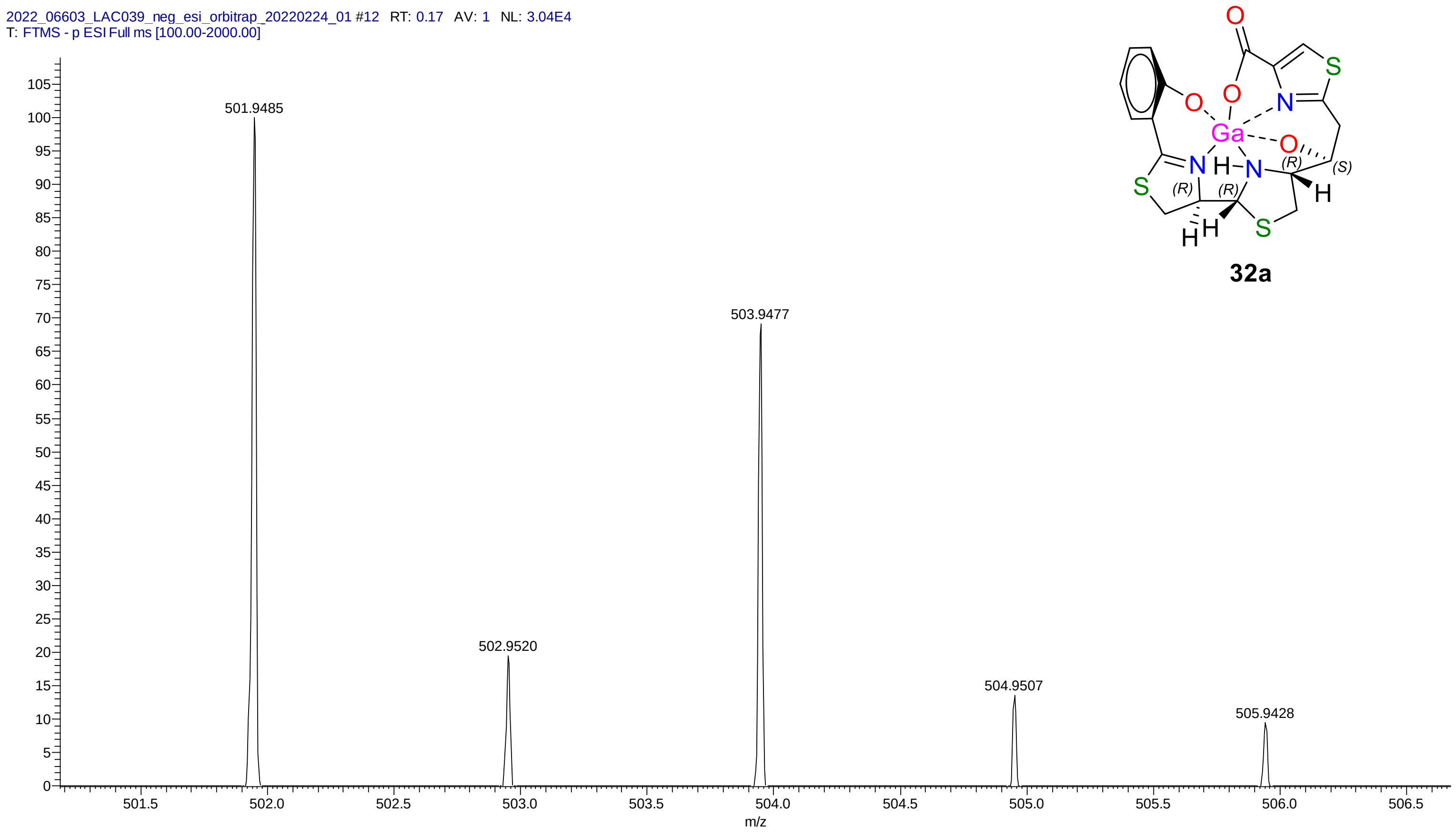

32a

Formula	Calc. *m/z*	Δ, mDa	Δ, ppm	DBE
$C_{18}H_{15}GaN_3O_4S_3$	501.9486	0.1	0.2	13.5

(+)-HRESIMS of **32**: Ion: *m/z*: 503.9636 ([M+H]$^+$), 525.9456 ([M+Na]$^+$)

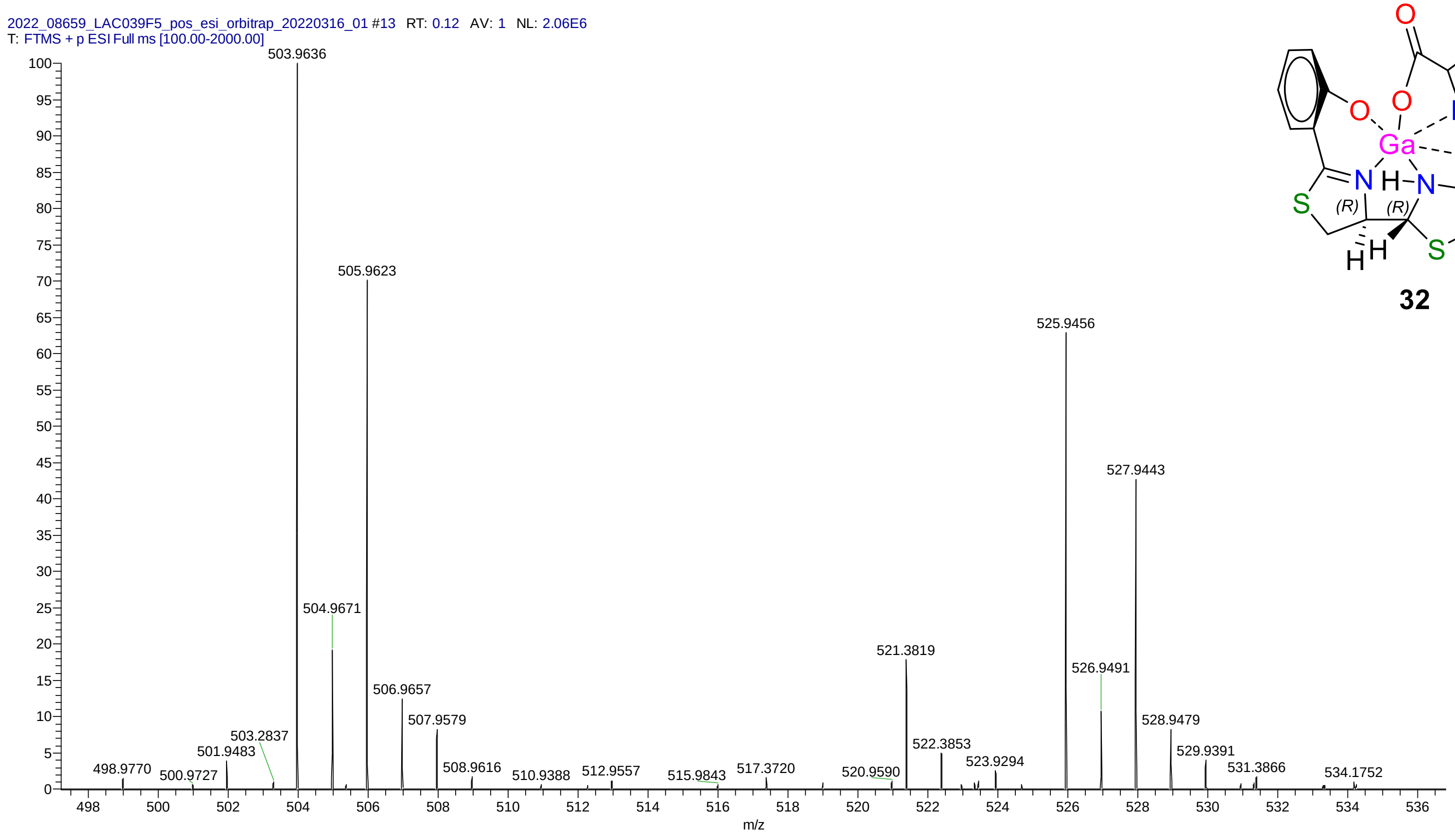

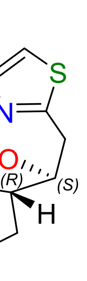

32

Formula	Calc. *m/z*	Δ, mDa	Δ, ppm	DBE
$C_{18}H_{17}GaN_3O_4S_3$, [M + H]$^+$	503.9637	0.1	0.2	13.5
$C_{18}H_{16}GaN_3O_4S_3Na$, [M+Na]$^+$	525.9456	0	0	13.5

5. NMR and MS of the intermediates from the stereoselective synthesis of thiazole intermediate *anti* epimer 39 (Scheme 7)

5.1 NMR and MS of 34

^{1}H NMR (400 MHz, $CDCl_3$) of **34**

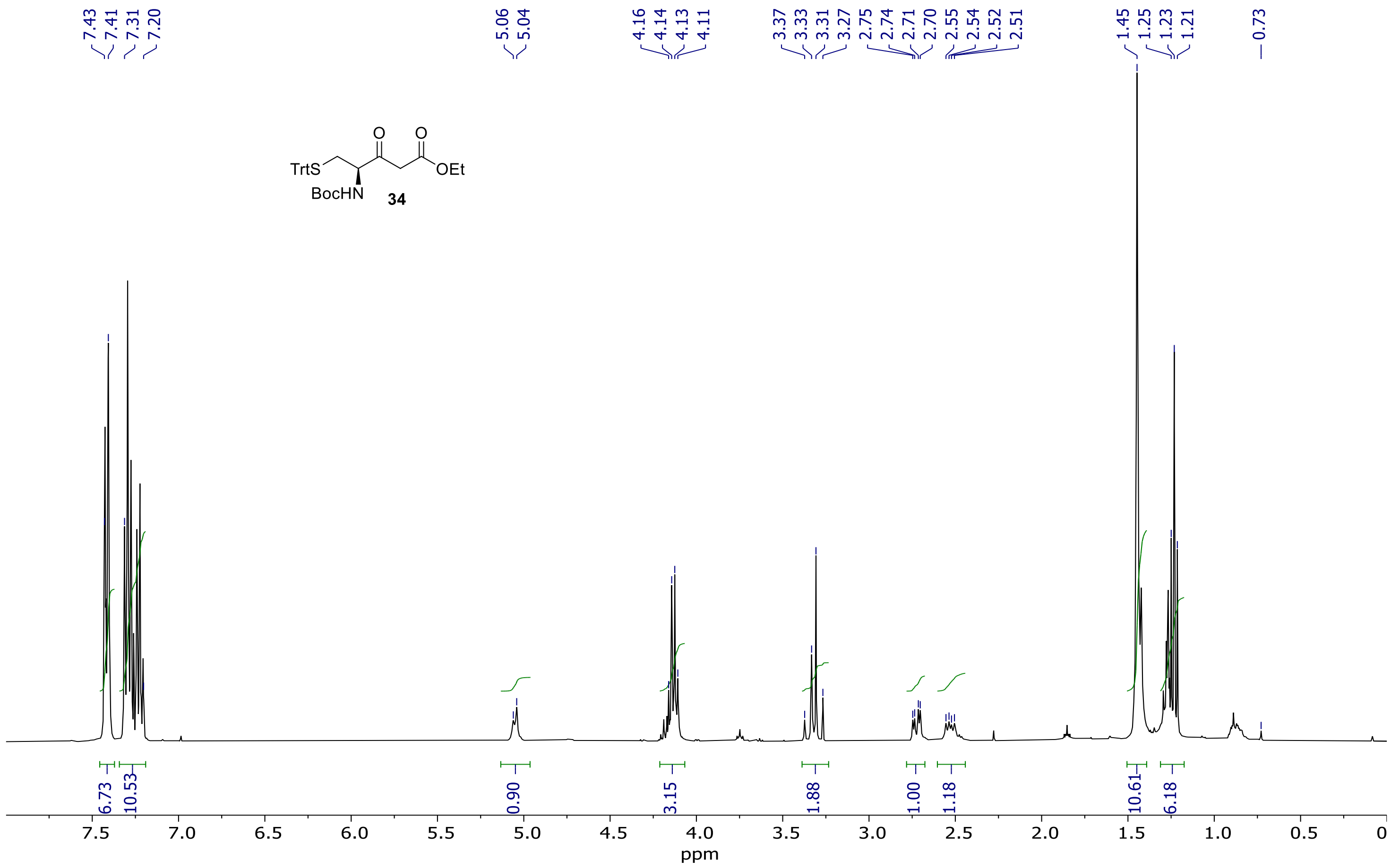

^{13}C NMR (100.13 MHz, $CDCl_3$) of **34**

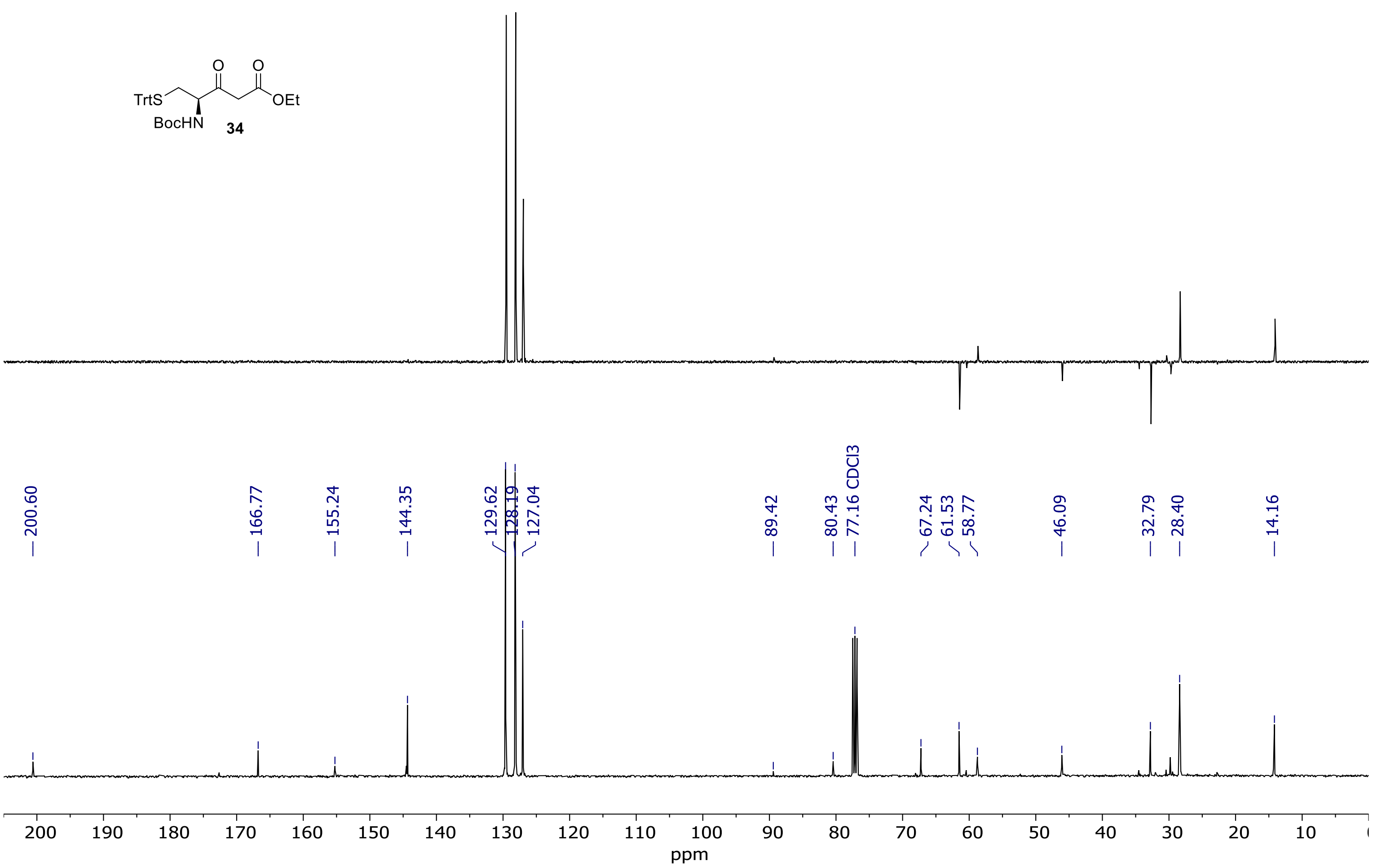

^{1}H-^{1}H COSY (400.13 MHz, CDCl3) of **34**

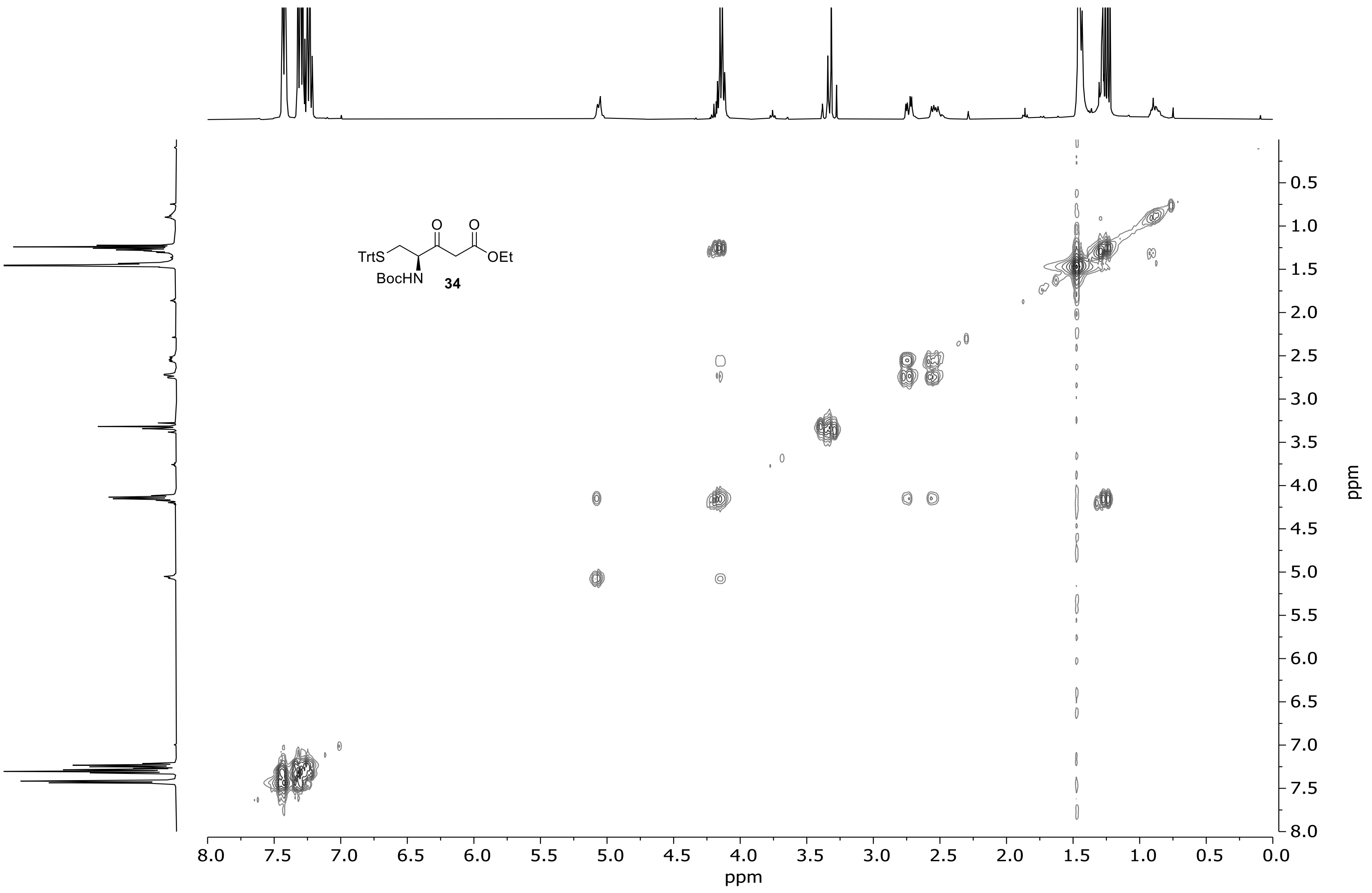

HSQC (400.13 MHz, $CDCl_3$) of **34**

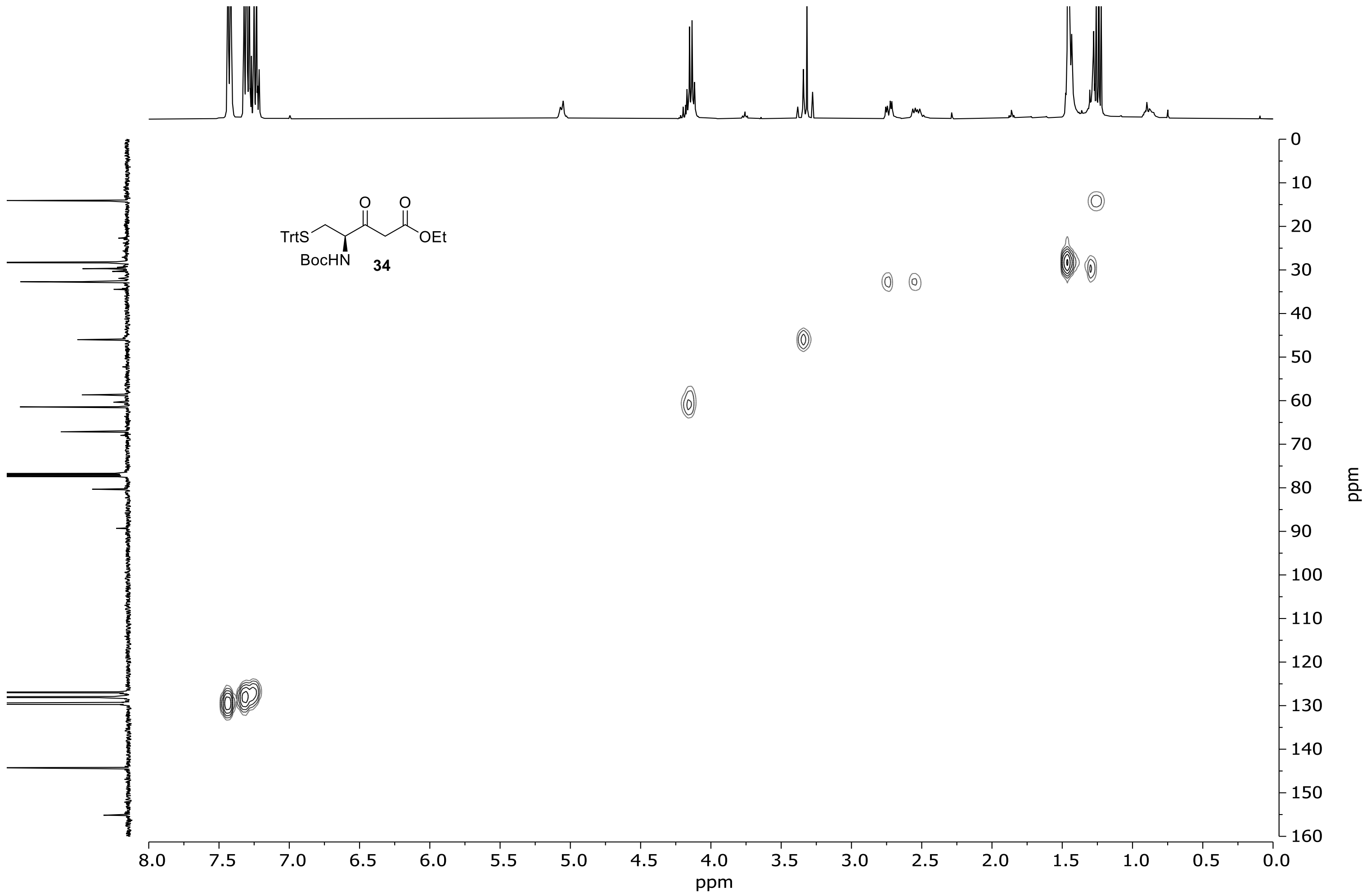

(+)-HRESIMS of **34**: Ion: *m/z*: 556.2133 ([M+Na]$^+$)

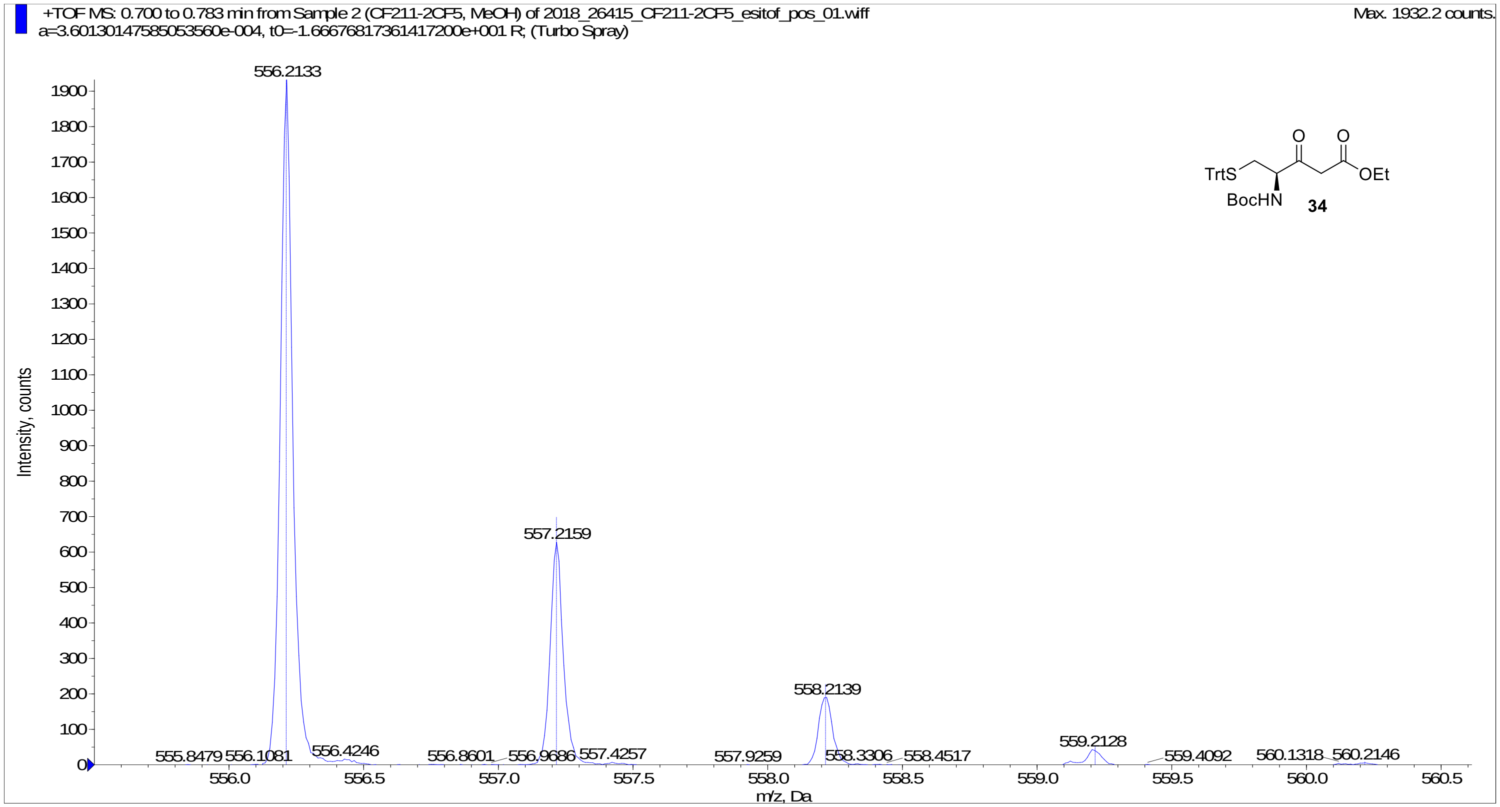

Formula	Calc m/z	Δ, mDa	Δ, ppm	DBE
$C_{31}H_{35}NO_5NaS$	556.2128	0.4834	0.8692	14.5

5.2 NMR and MS of 35

^{1}H NMR (300 MHz, $CDCl_3$) of **35**

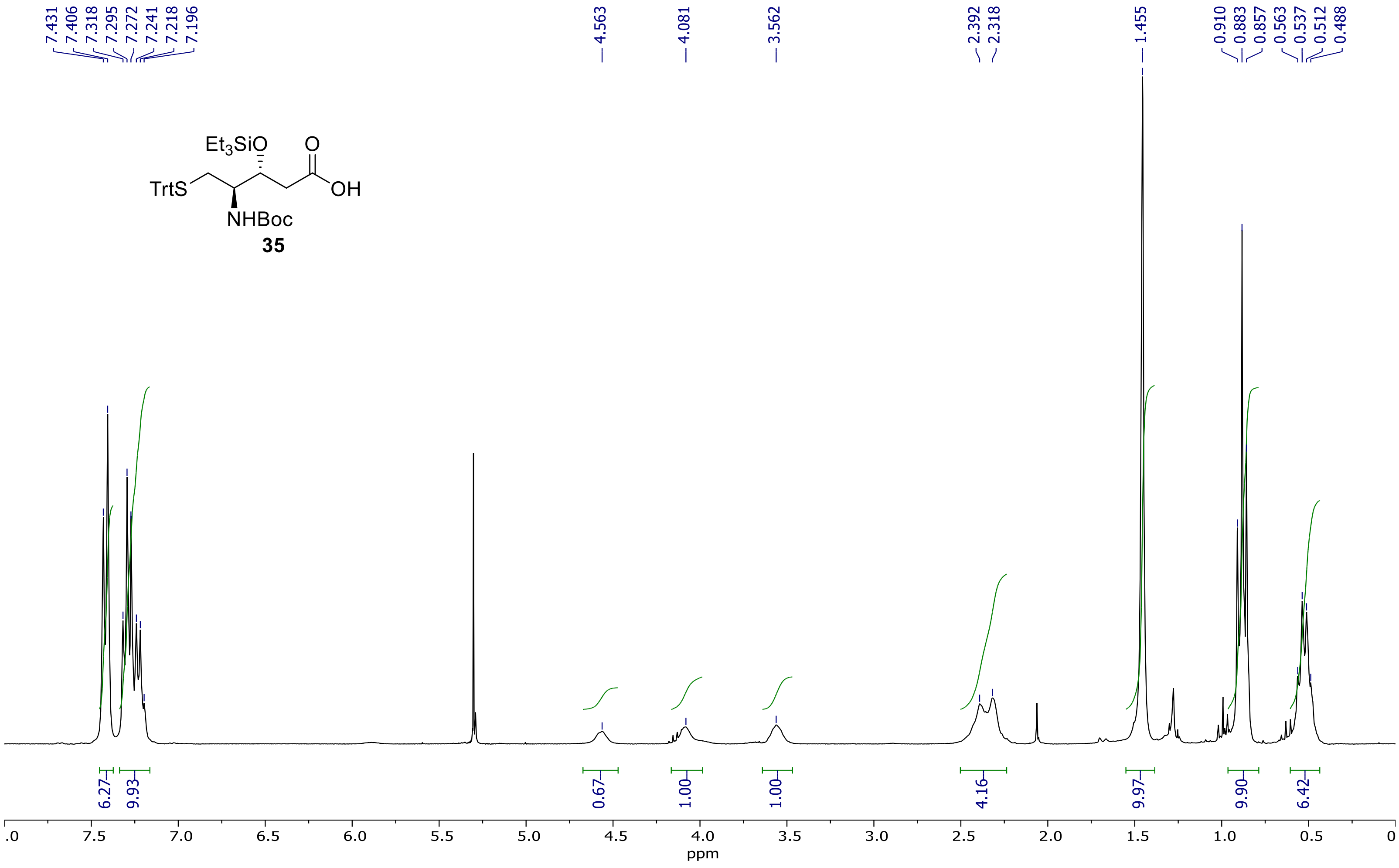

^{13}C NMR (75 MHz, $CDCl_3$) of **35**

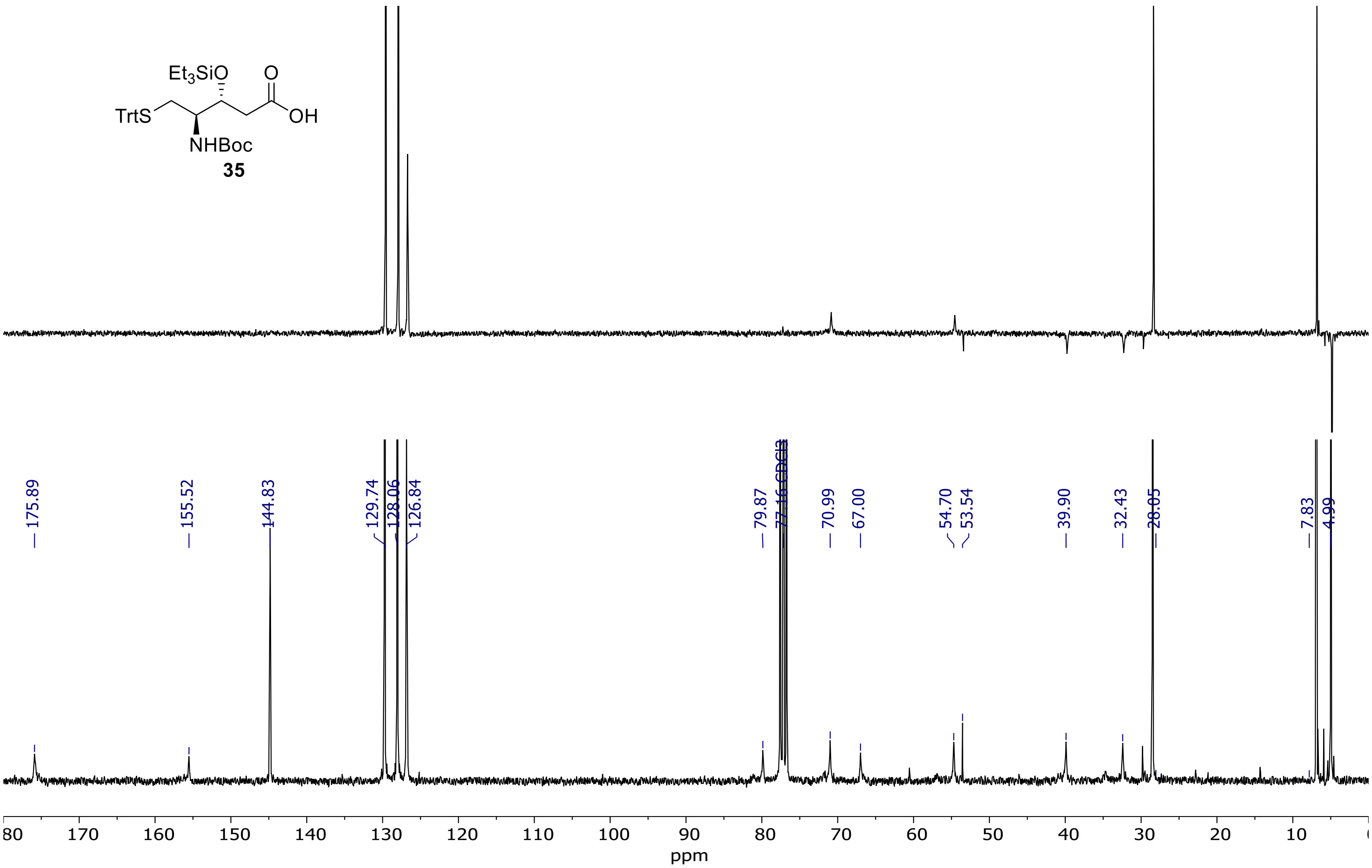

^{1}H-^{1}H COSY (75 MHz, CDCl$_3$) of **35**

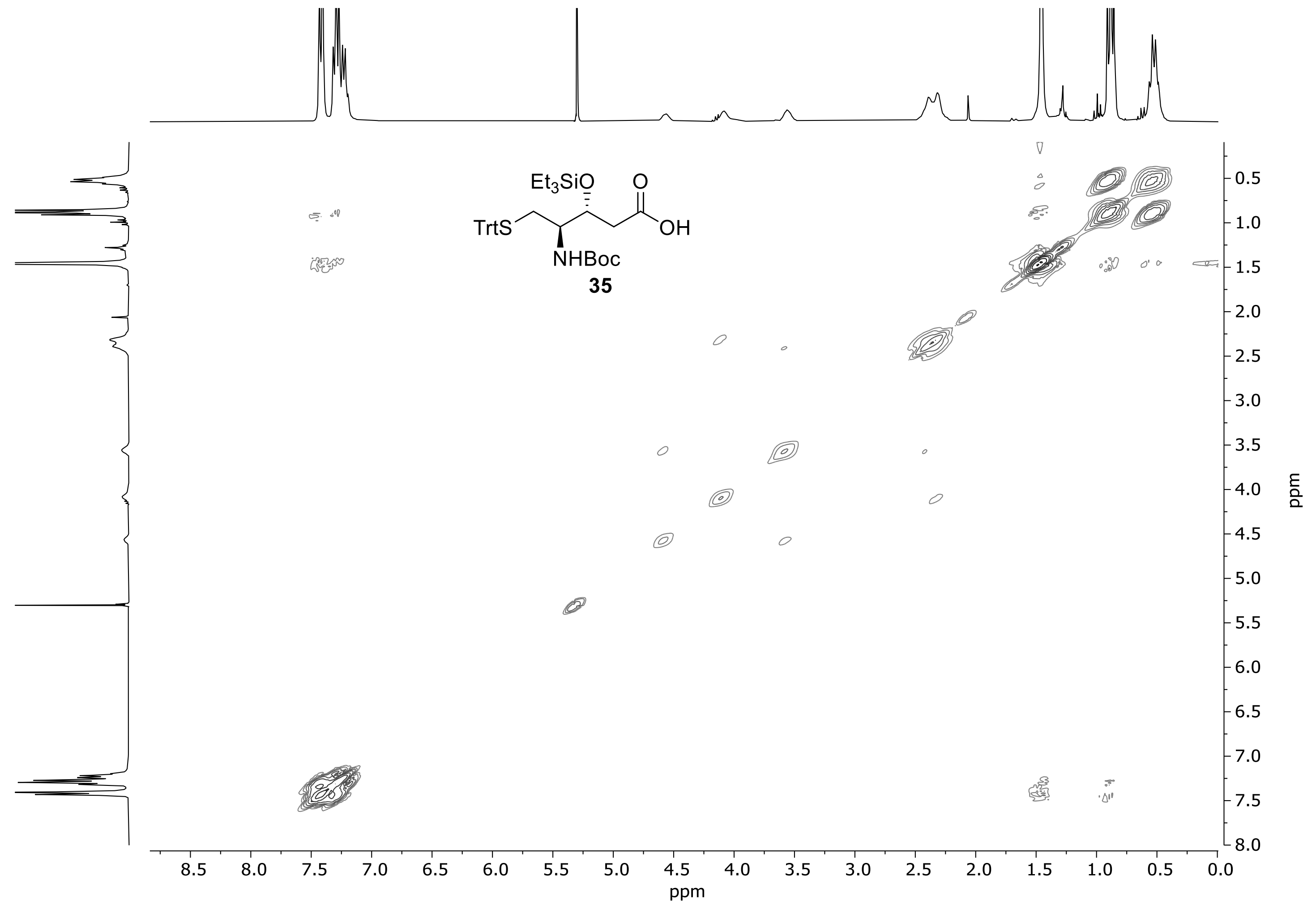

HSQC (300.13 MHz, $CDCl_3$) of **35**

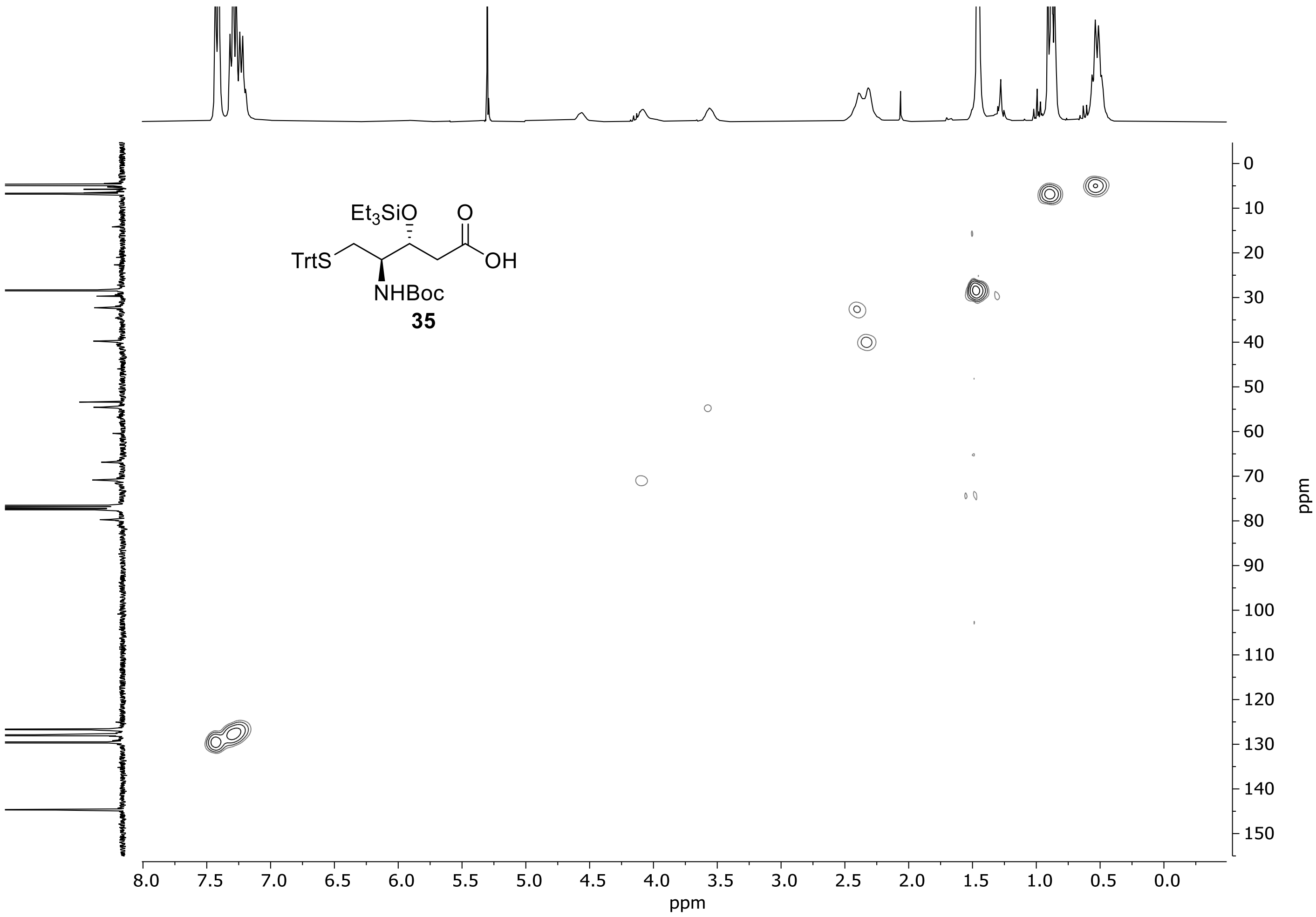

(+)-HRESIMS of **35**: Ion: *m/z*: 644.2828 $[M+Na]^+$

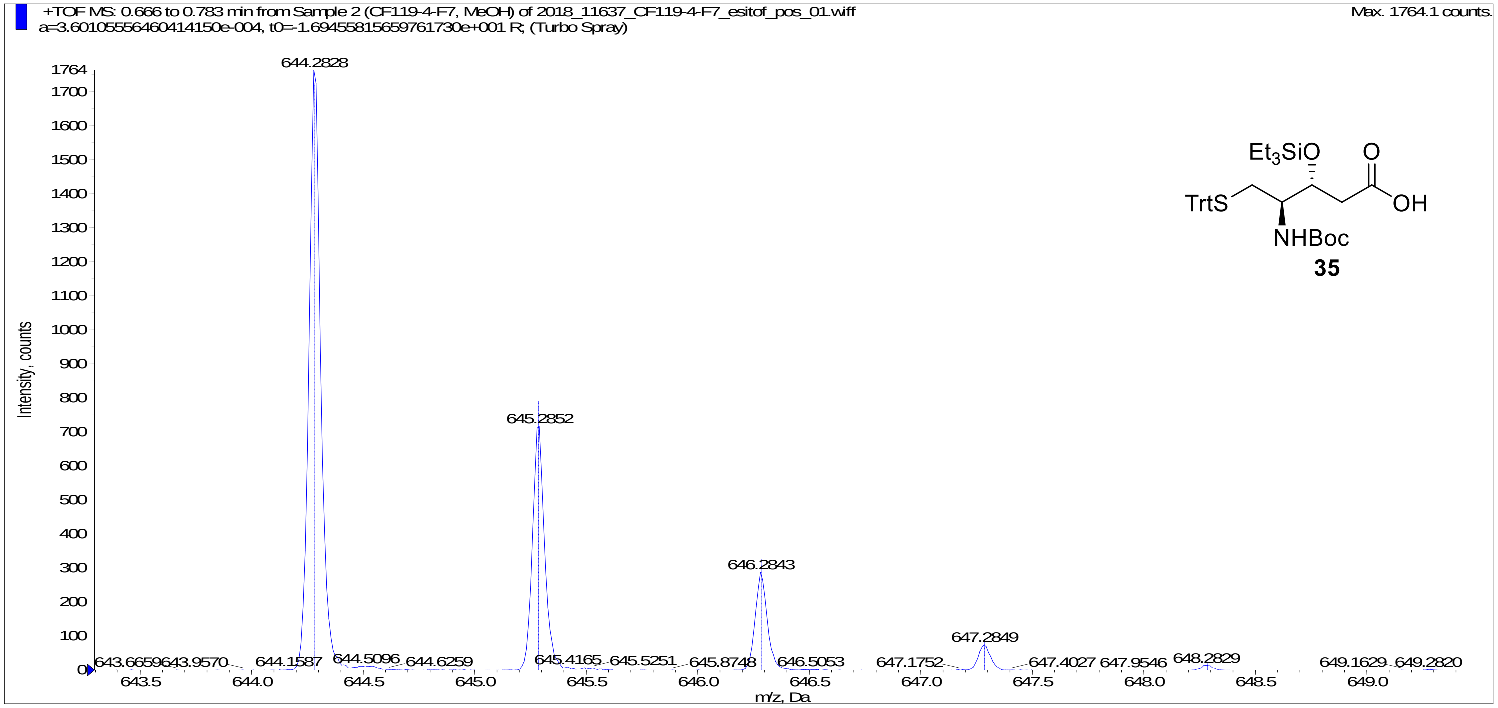

35

Formula	Calc m/z	Δ, mDa	Δ, ppm	DBE
$C_{35}H_{47}NO_5NaSiS$	644.2836	-0.8454	-1.3121	13.5

5.3 NMR and MS of 36

^{1}H NMR (400 MHz, $CDCl_3$) of **36**

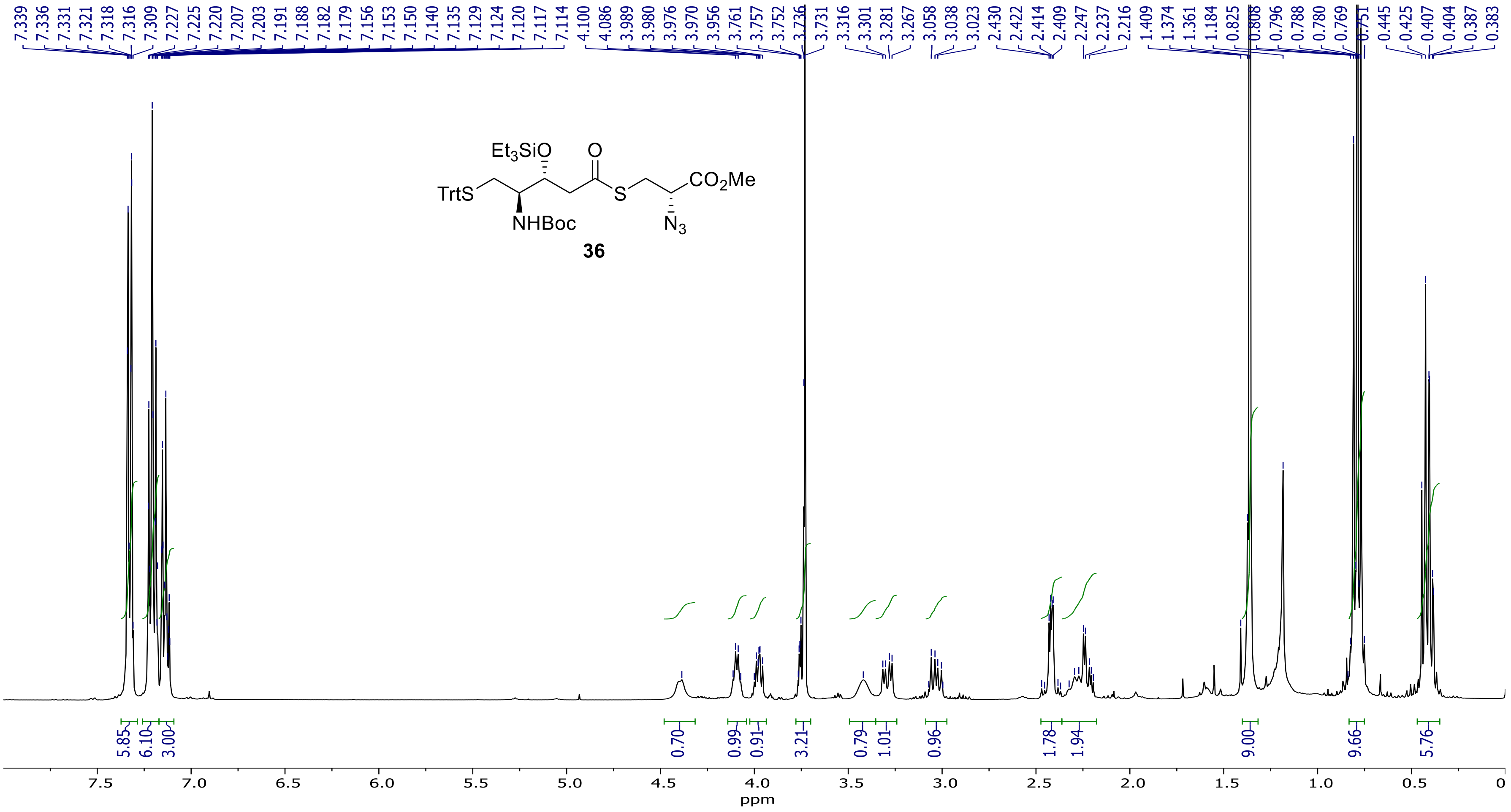

^{13}C NMR (100.13 MHz, $CDCl_3$) of **36**

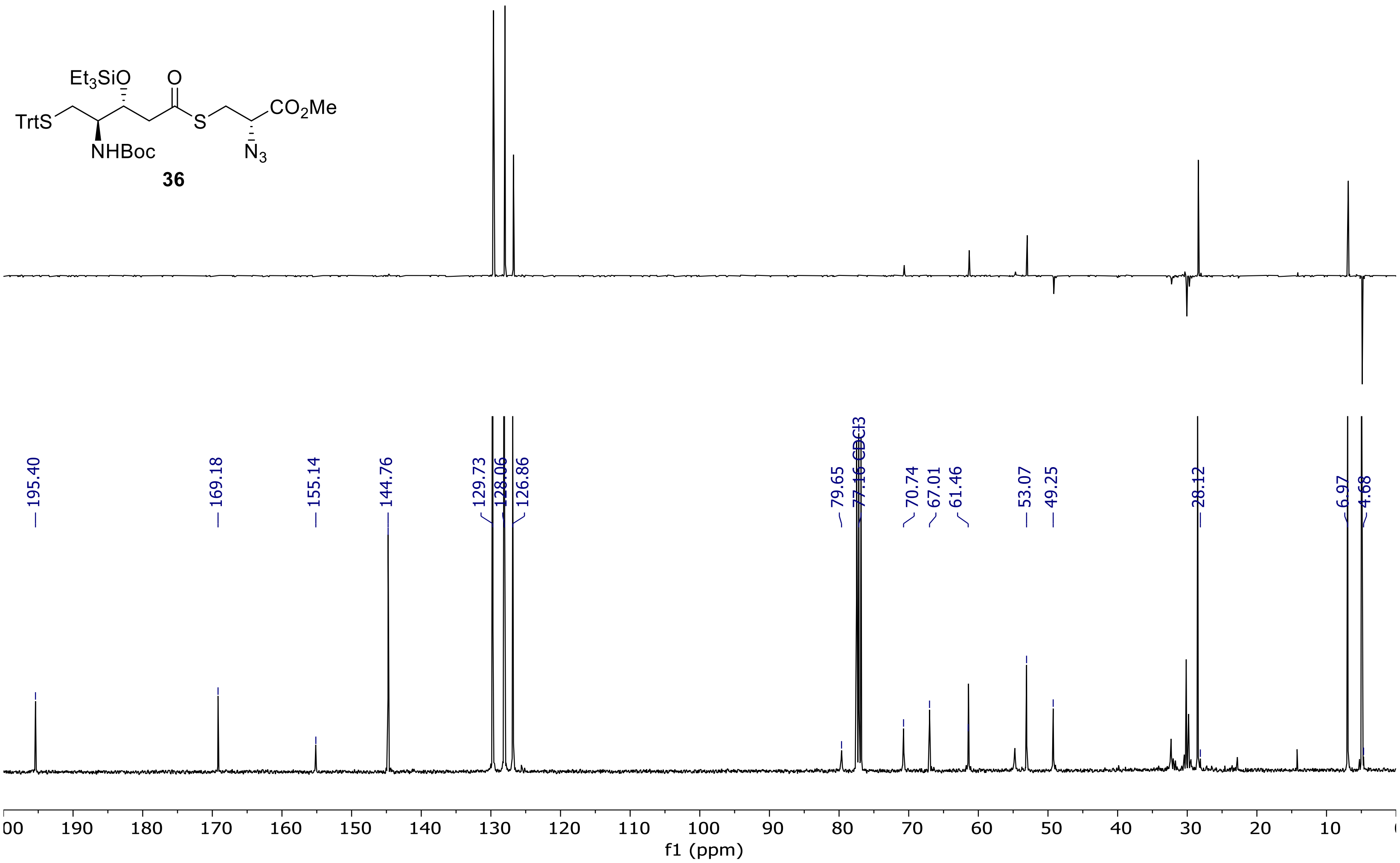

^{1}H-^{1}H COSY (400.13 MHz, $CDCl_3$) of **36**

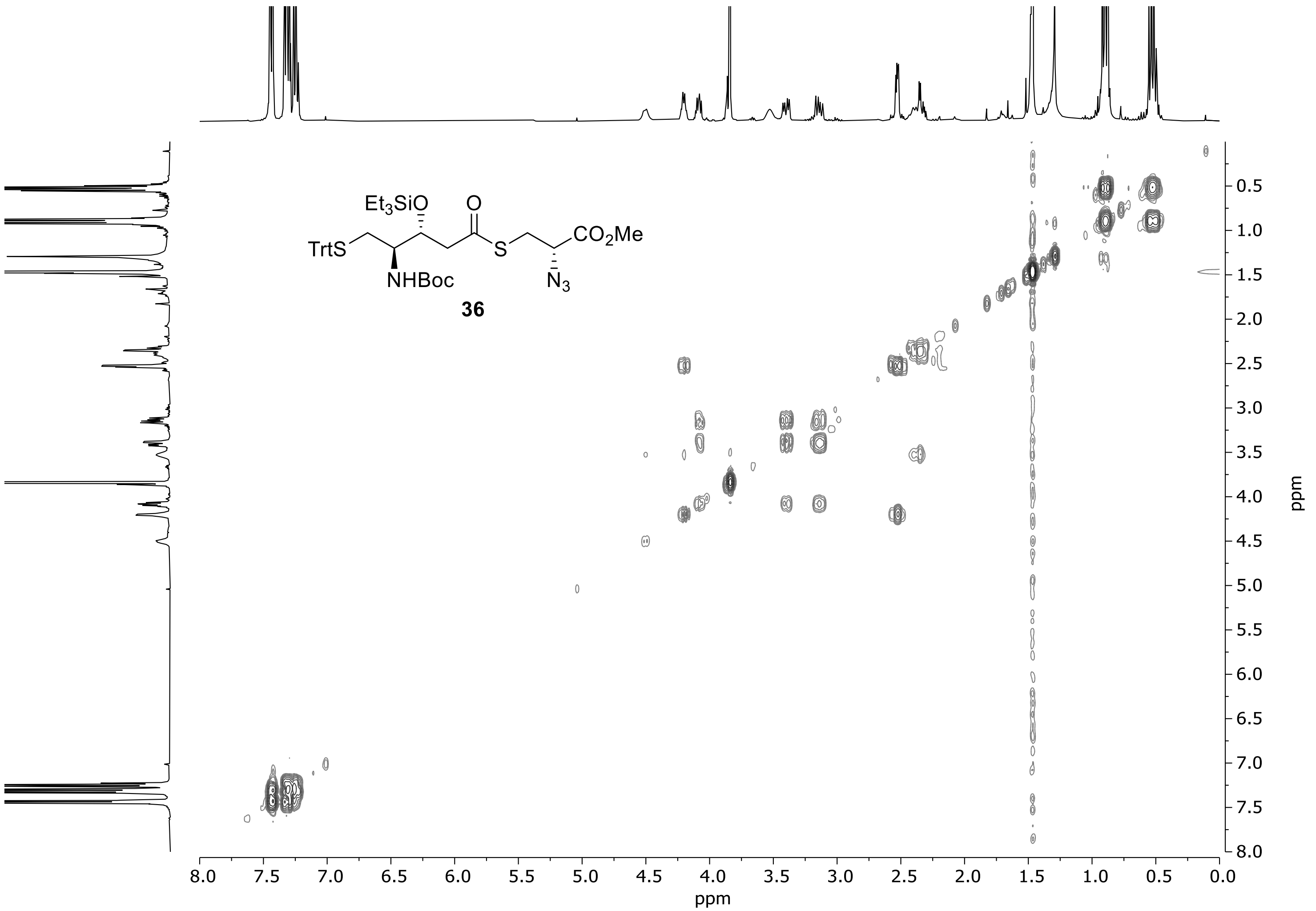

HSQC (400.13 MHz, CDCl3) of **36**

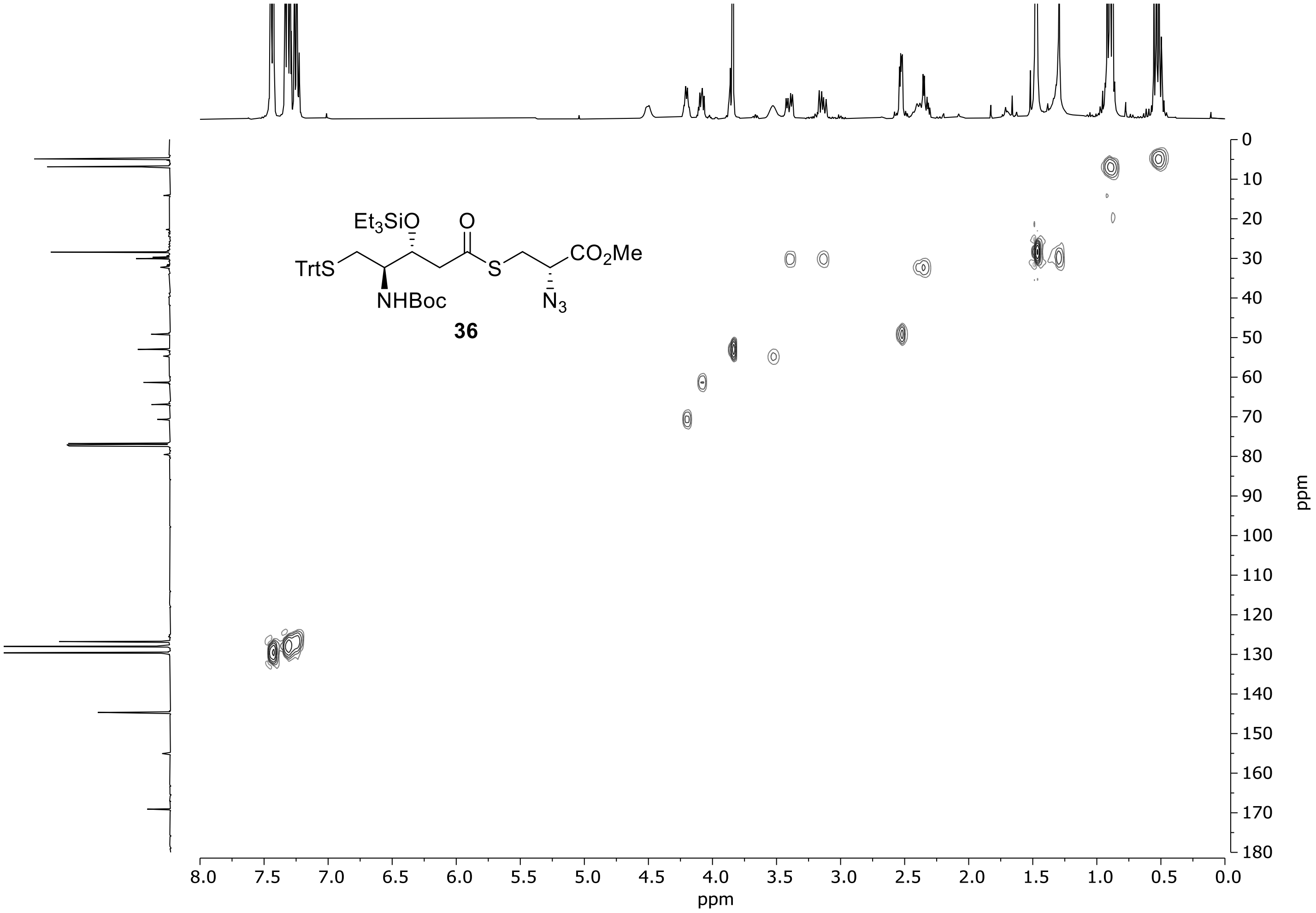

(+)-HRESIMS of **36**: Ion: *m/z*: 765.3181 ([M+H]$^+$)

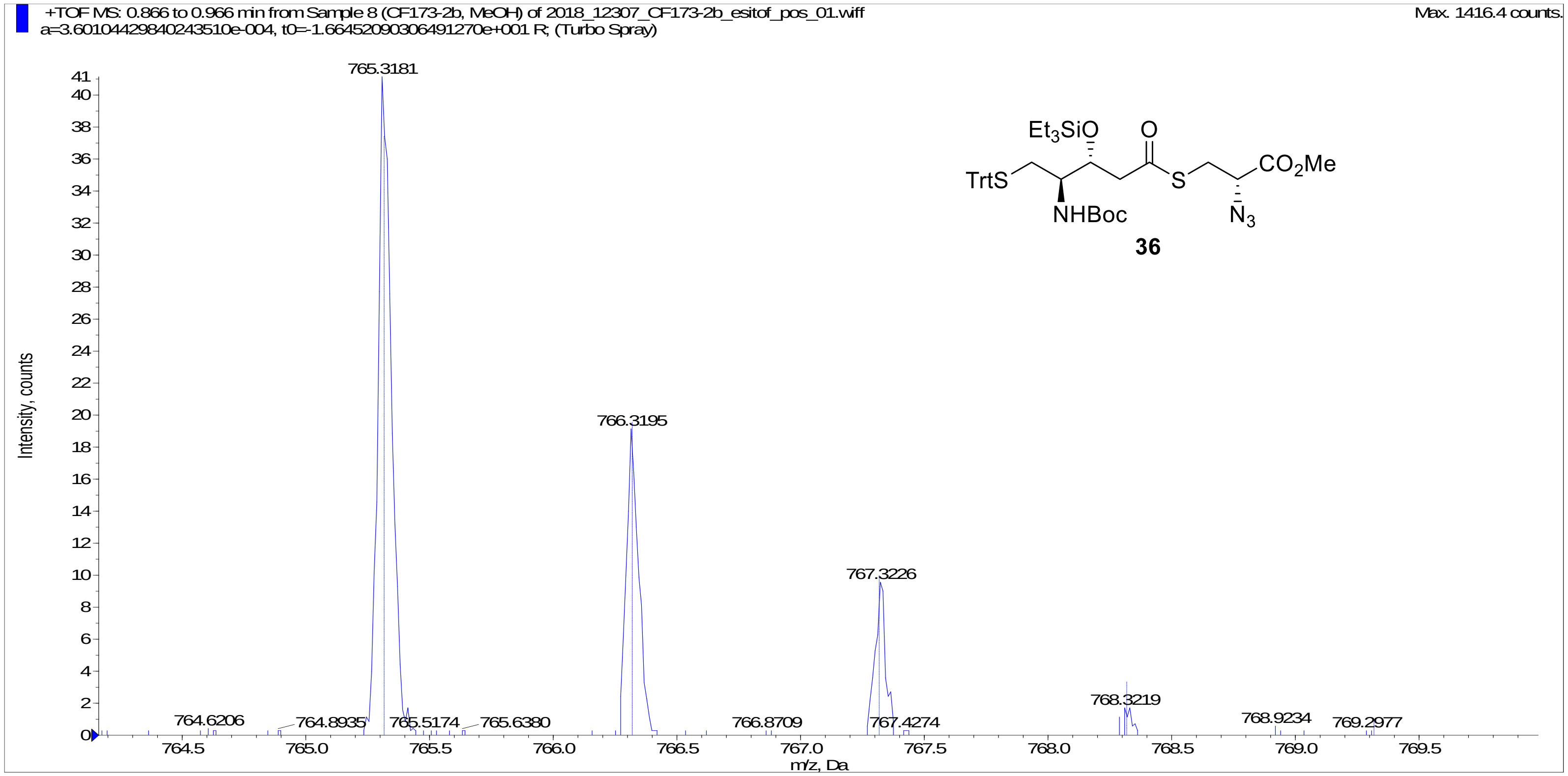

Formula	Calc m/z	Δ, mDa	Δ, ppm	DBE
$C_{39}H_{53}N_4O_6SiS_2$	765.3170	1.0655	1.3923	16.5

5.4 NMR and MS of 37

^{1}H NMR (400 MHz, $CDCl_3$) of **37**

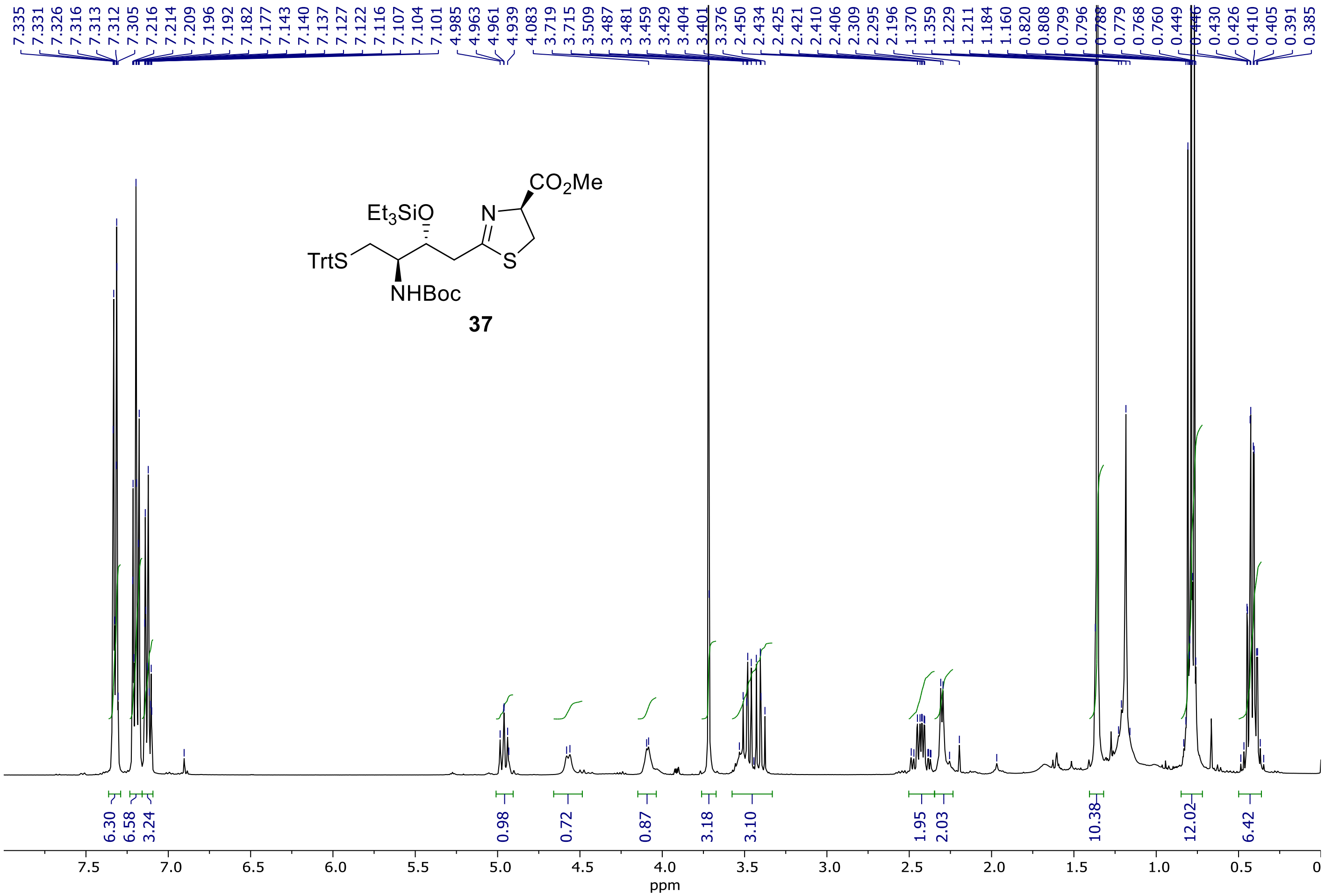

^{13}C NMR (100.13 MHz, $CDCl_3$) of **37**

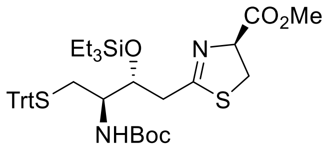

37

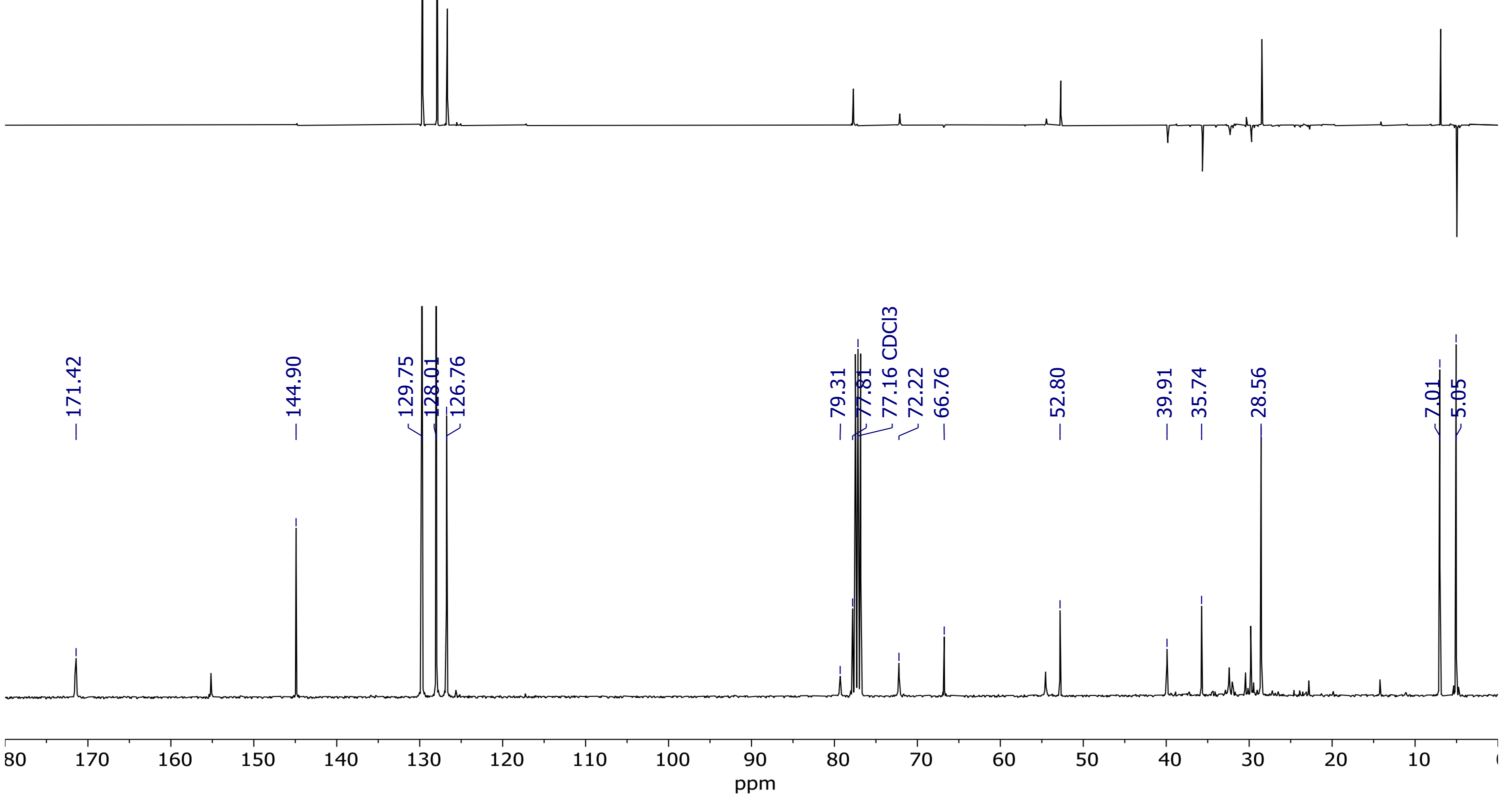

^1H-^1H COSY (400.13 MHz, $CDCl_3$) of **37**

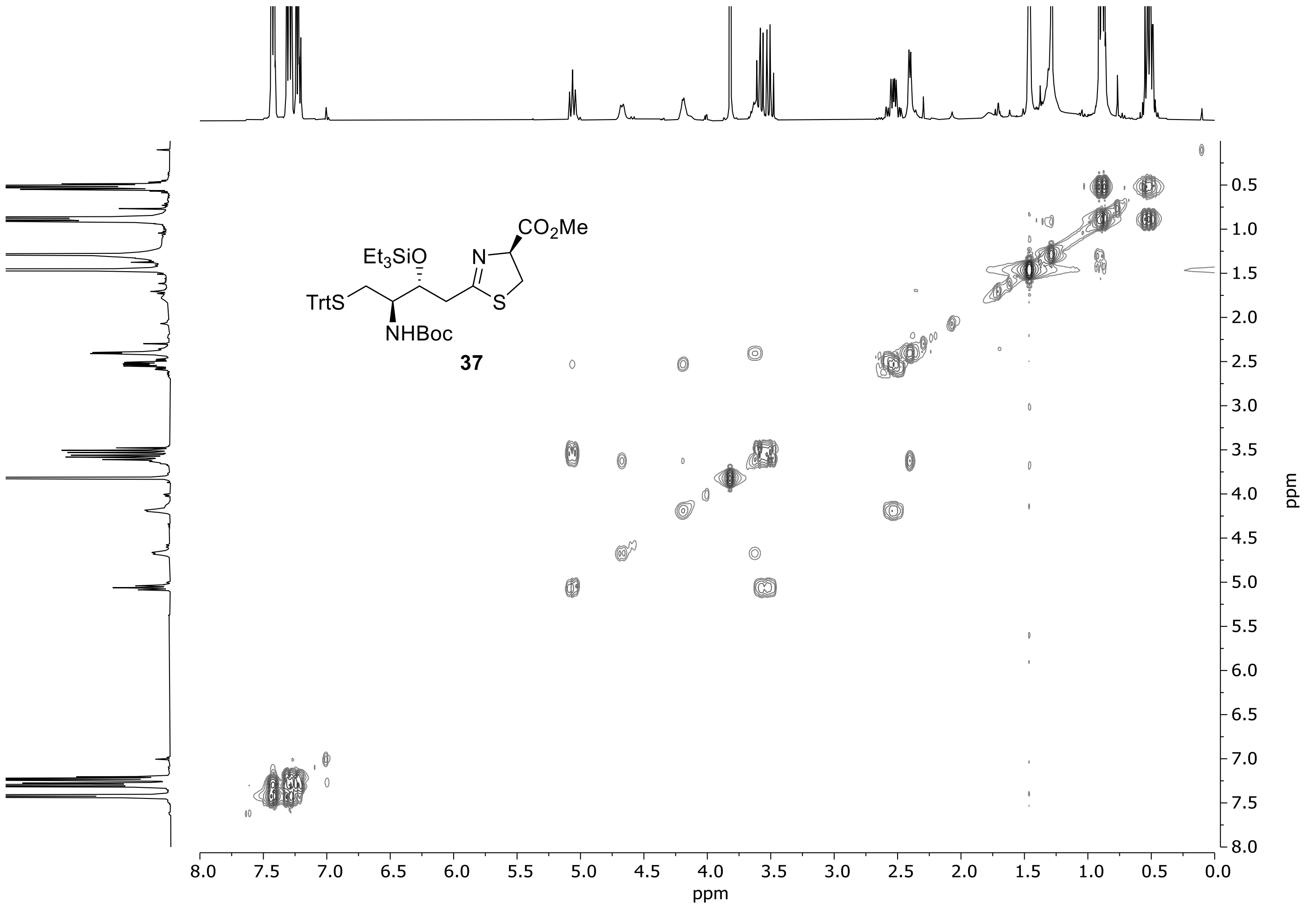

HSQC (400.13 MHz, $CDCl_3$) of **37**

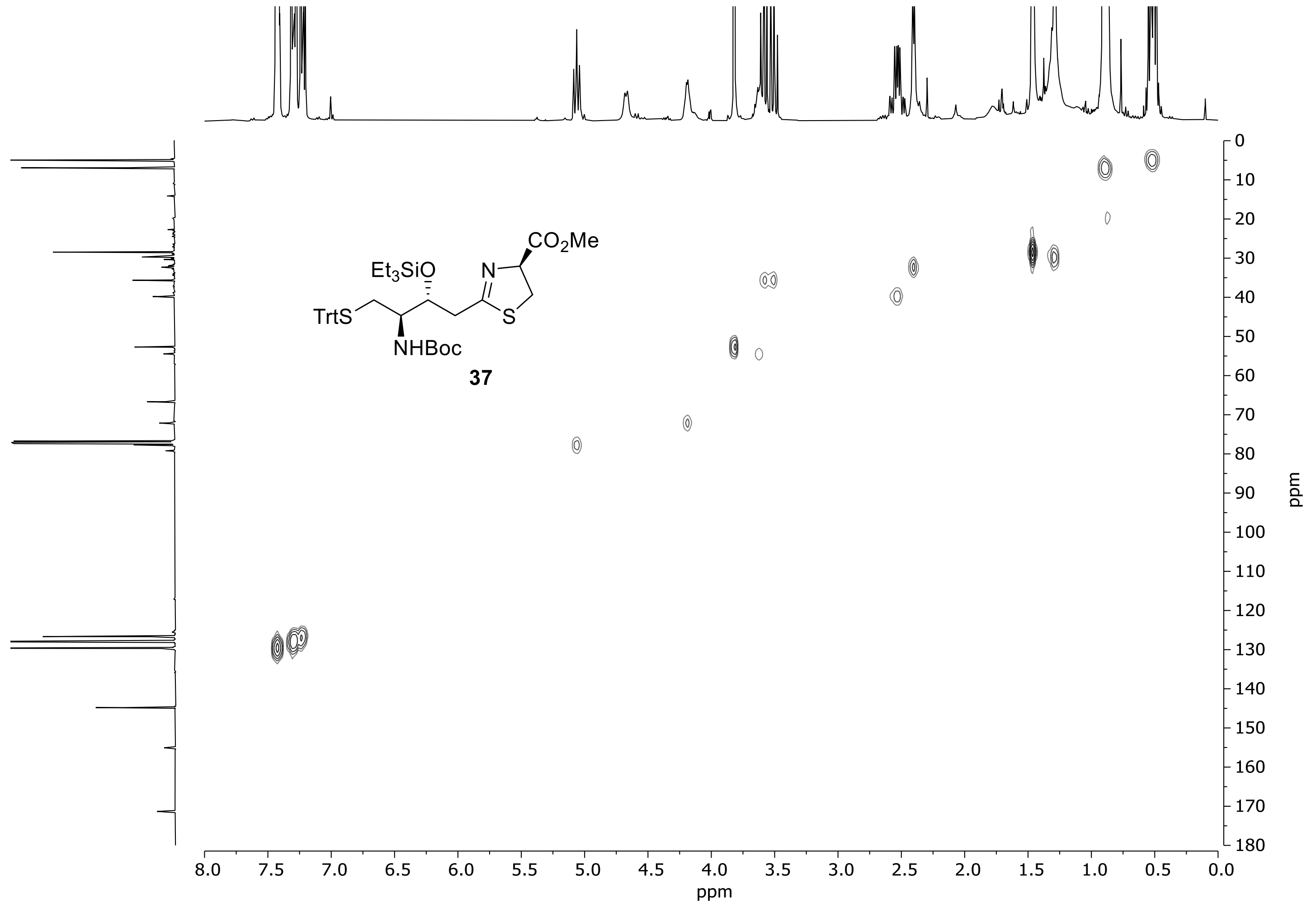

(+)-HRESIMS of **37**: Ion: *m/z*: 721.3170 $[M+H]^+$

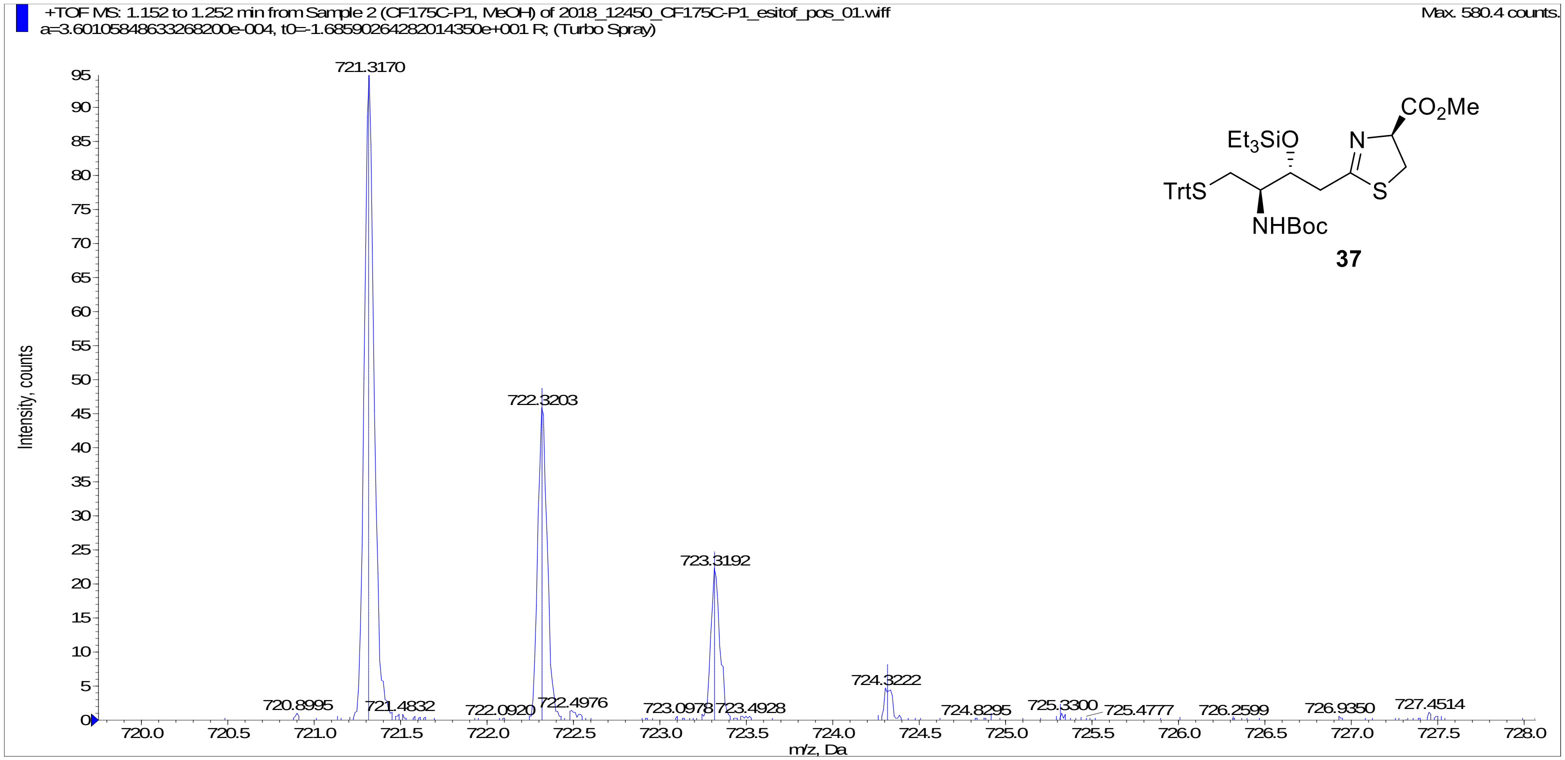

Formula	Calc m/z	Δ, mDa	Δ, ppm	DBE
$C_{39}H_{53}N_2O_5SiS_2$	721.3159	1.0282	1.4255	15.5

5.5 NMR and MS of 38

^{1}H NMR (300 MHz, CDCl$_3$) of **38**

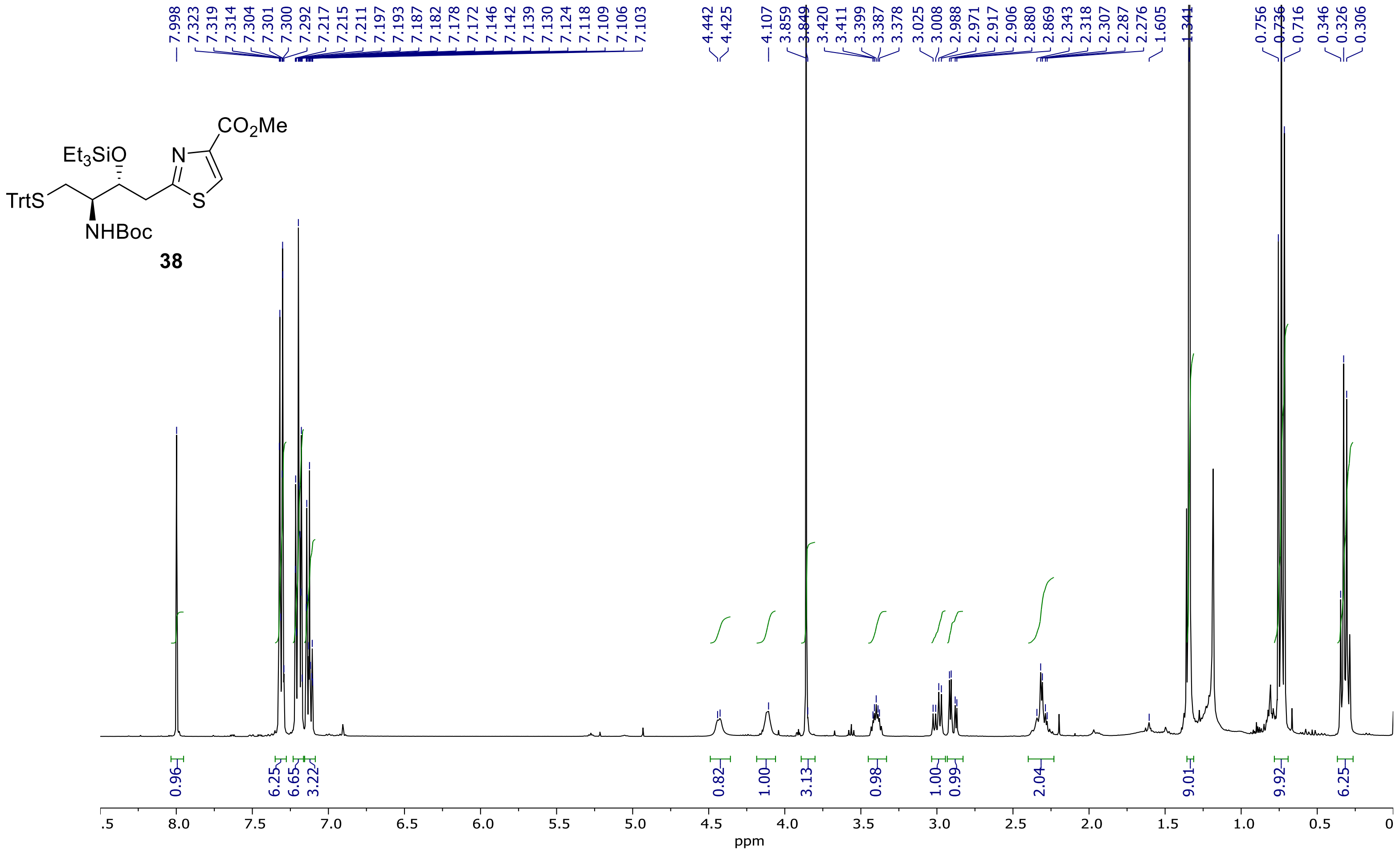

^{13}C NMR (75 MHz, CDCl$_3$) of **38**

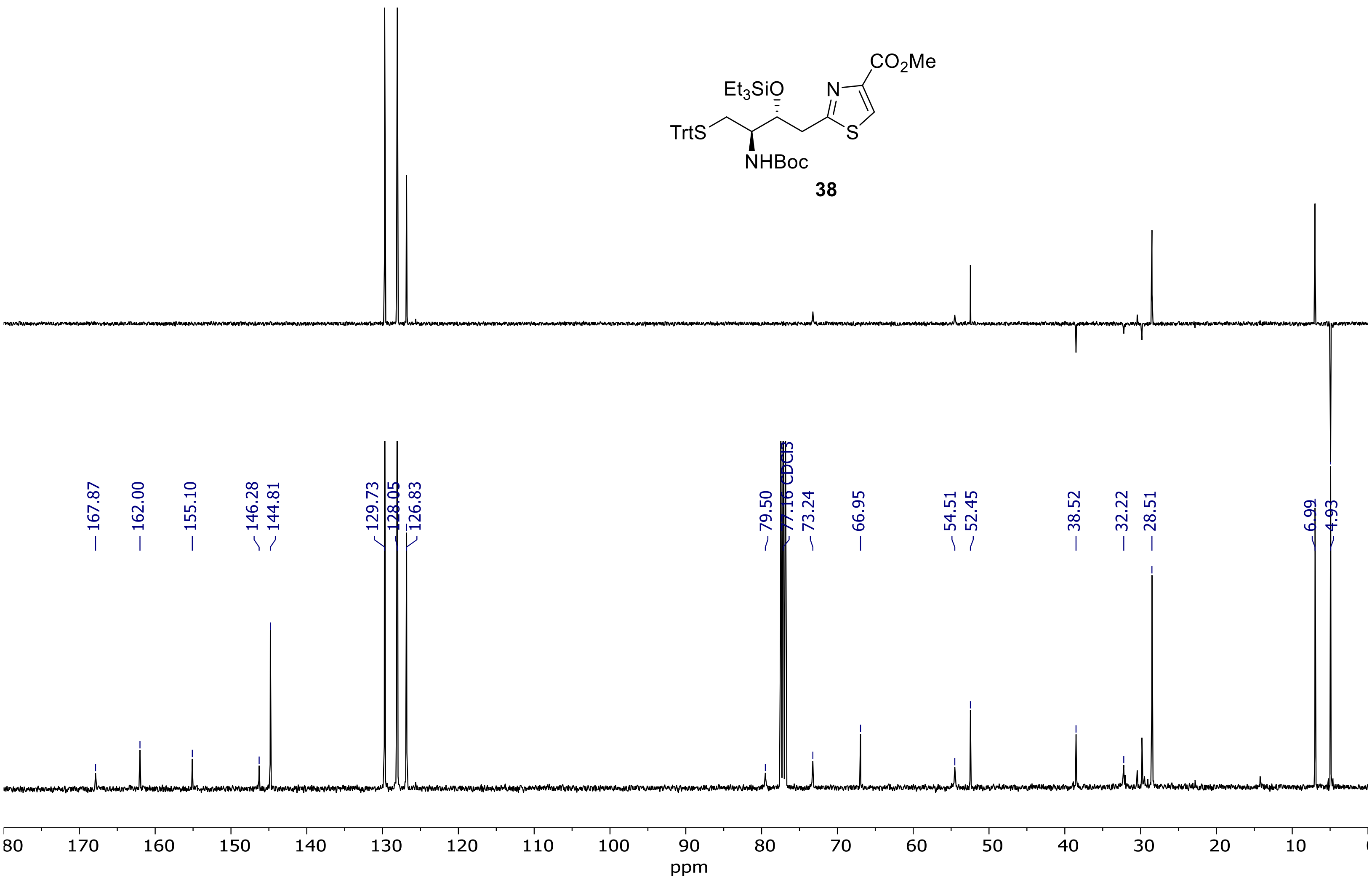

^{1}H-^{1}H COSY (300.13 MHz, $CDCl_3$) of **38**

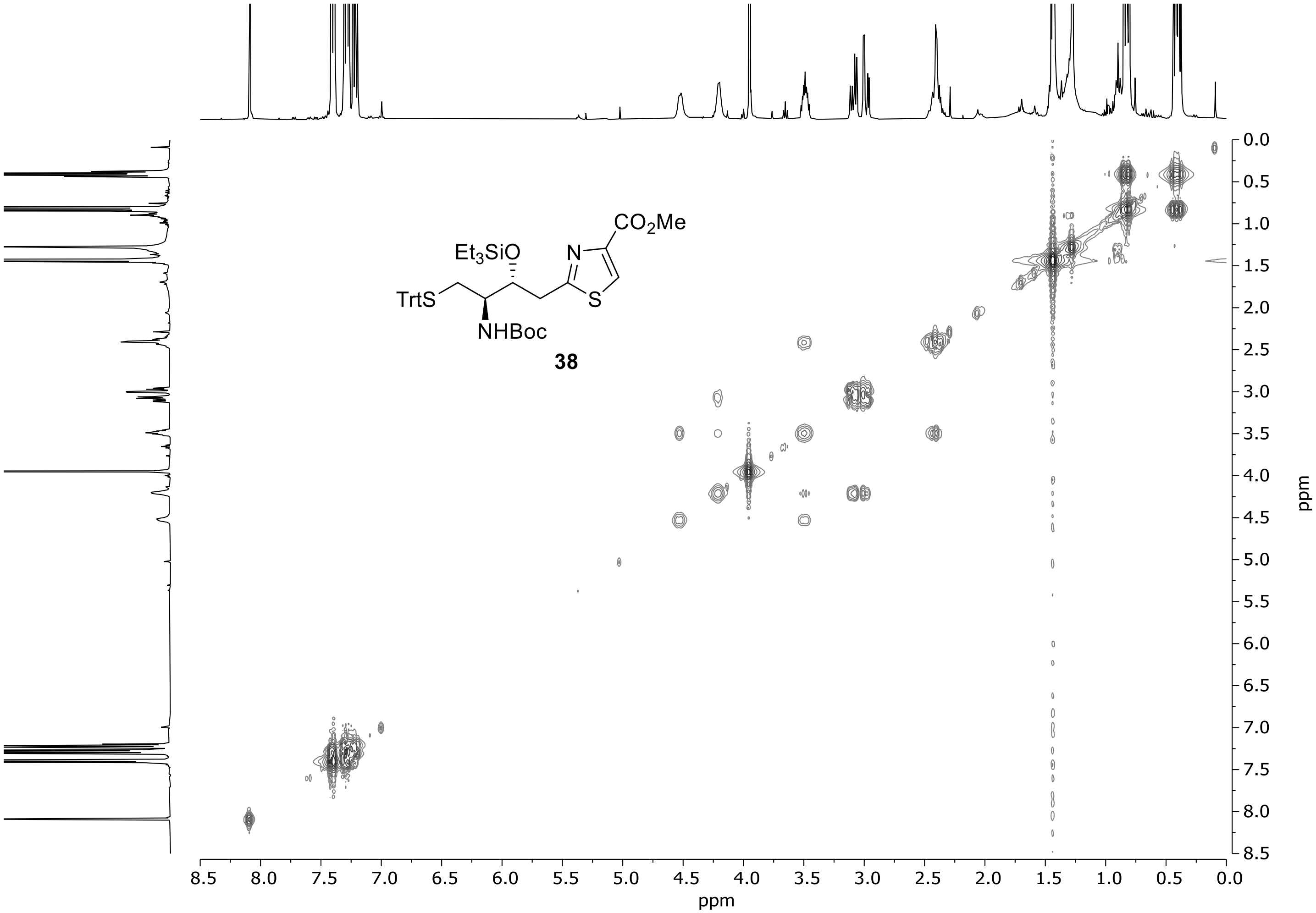

HSQC (300.13 MHz, $CDCl_3$) of **38**

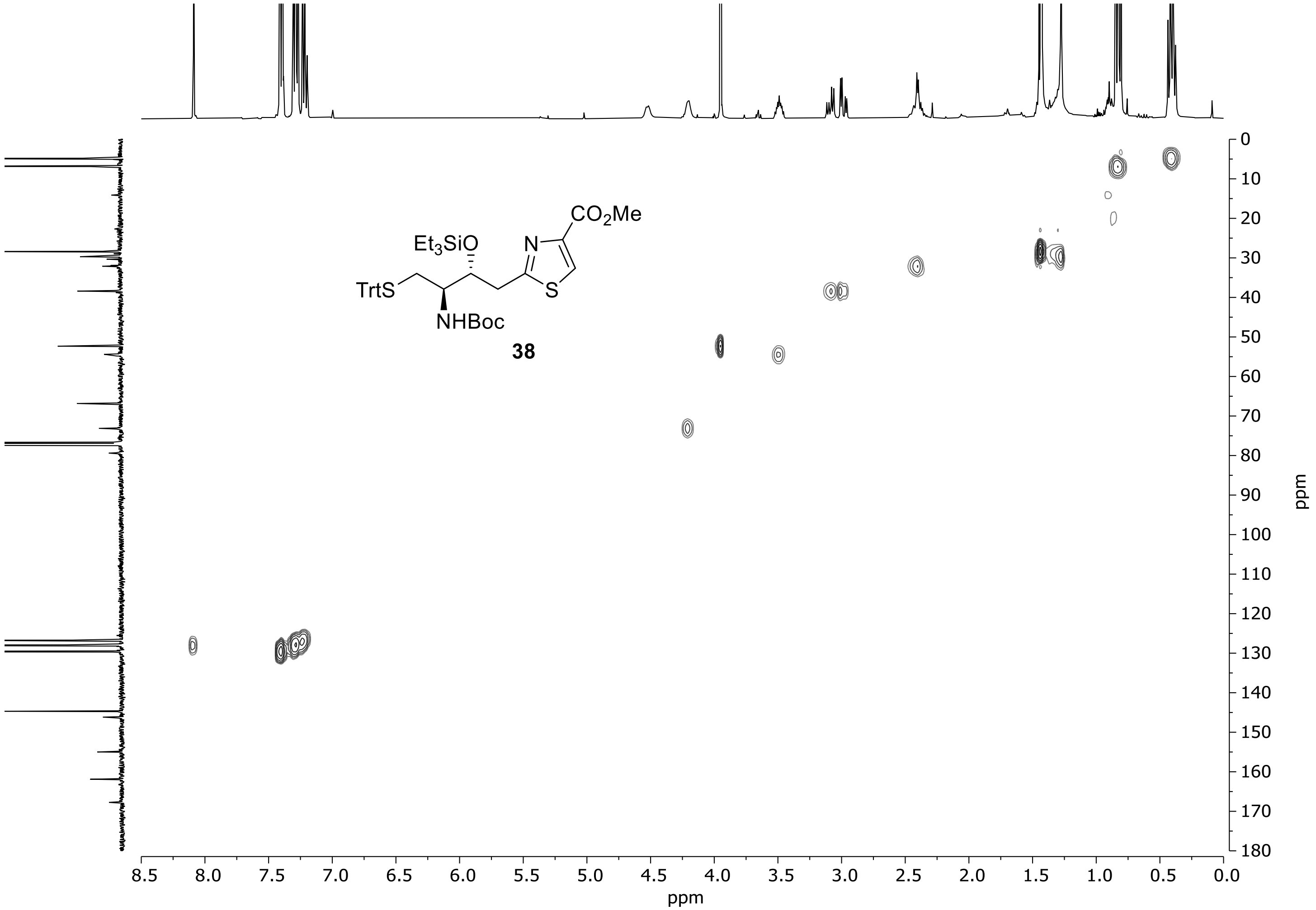

(+)-HRESIMS of **38**: Ion: *m/z*: 719.3024 $[M+H]^+$

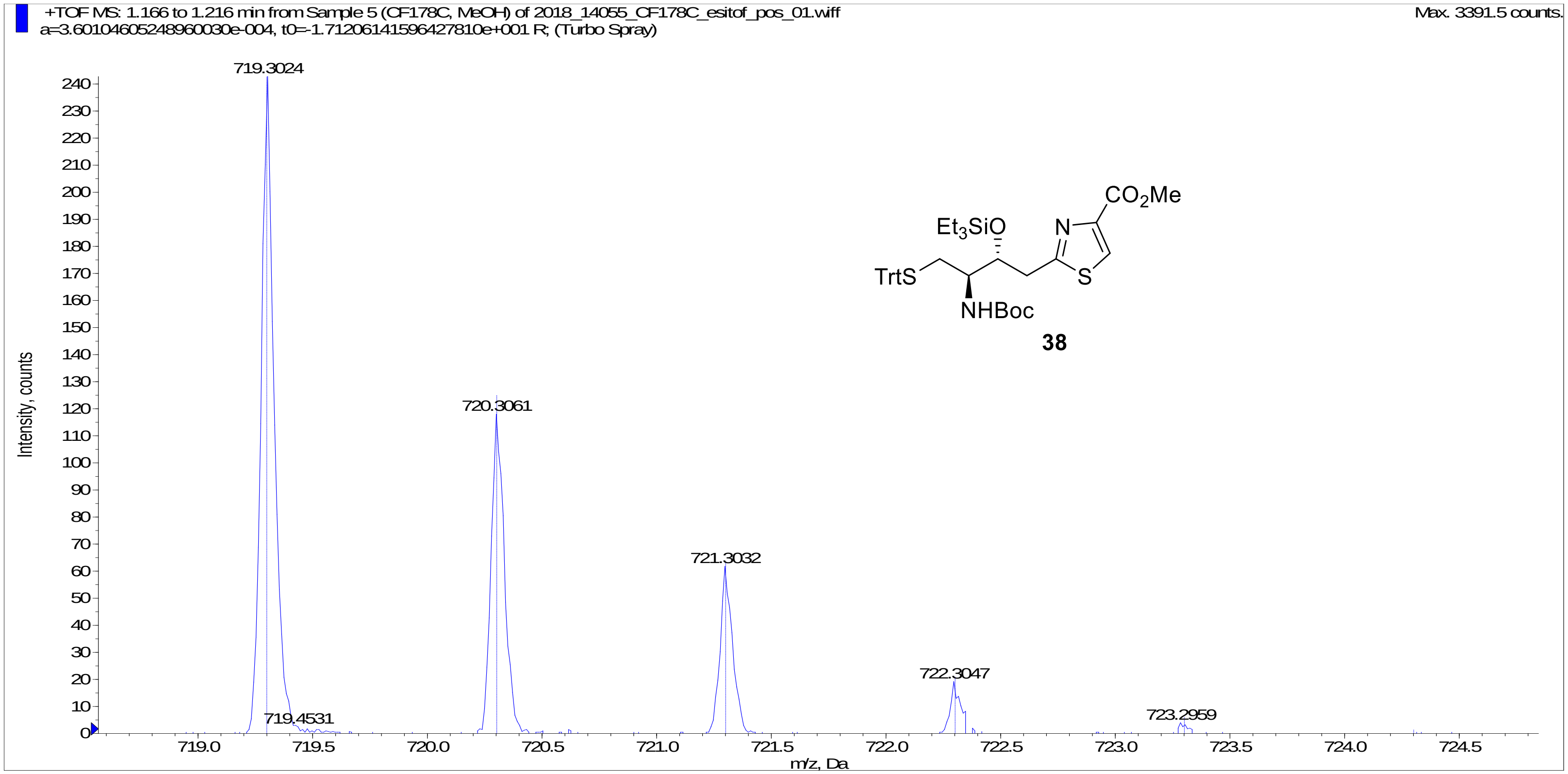

Formula	Calc m/z	Δ, mDa	Δ, ppm	DBE
$C_{39}H_{51}N_2O_5SiS_2$	719.3003	2.0783	2.8893	16.5

5.6 NMR and MS of 39

^{1}H NMR (500.13 MHz, $CDCl_3$) of **39**

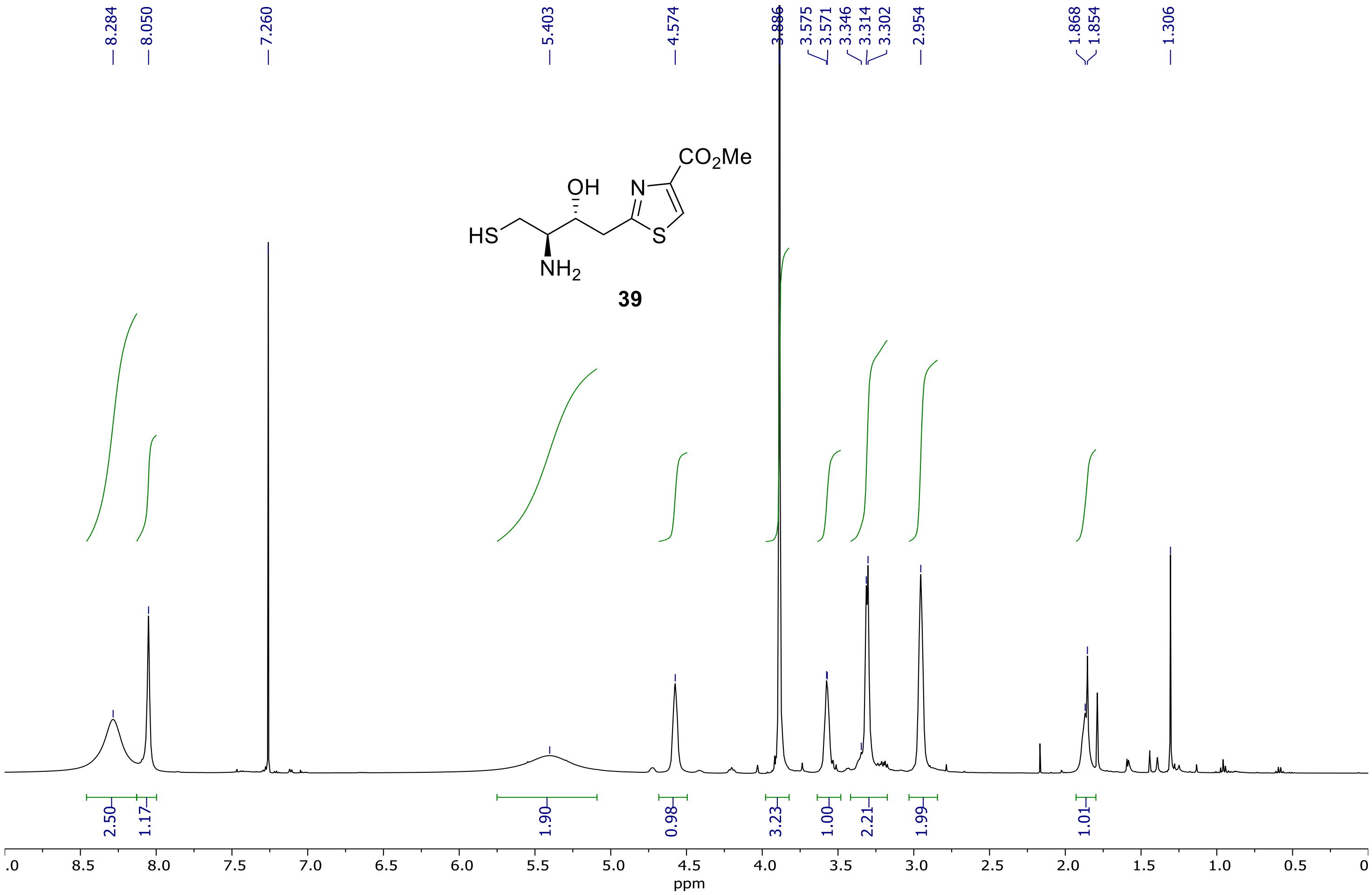

^{13}C NMR (125.13 MHz, $CDCl_3$) of **39**

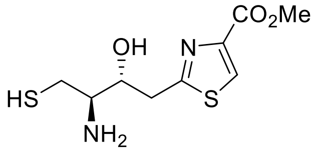

39

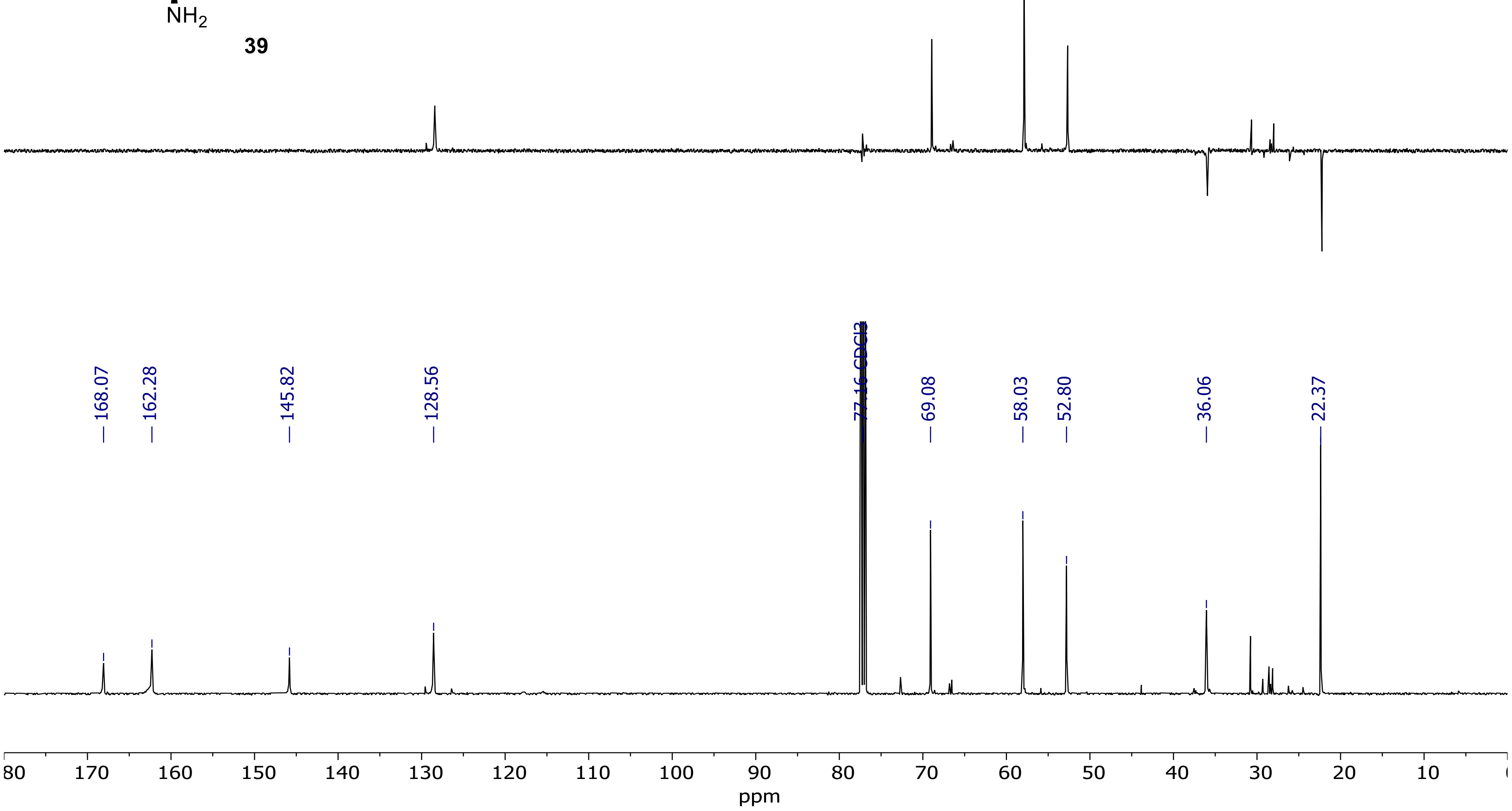

^{1}H-^{1}H COSY (500.13 MHz, $CDCl_3$) of **39**

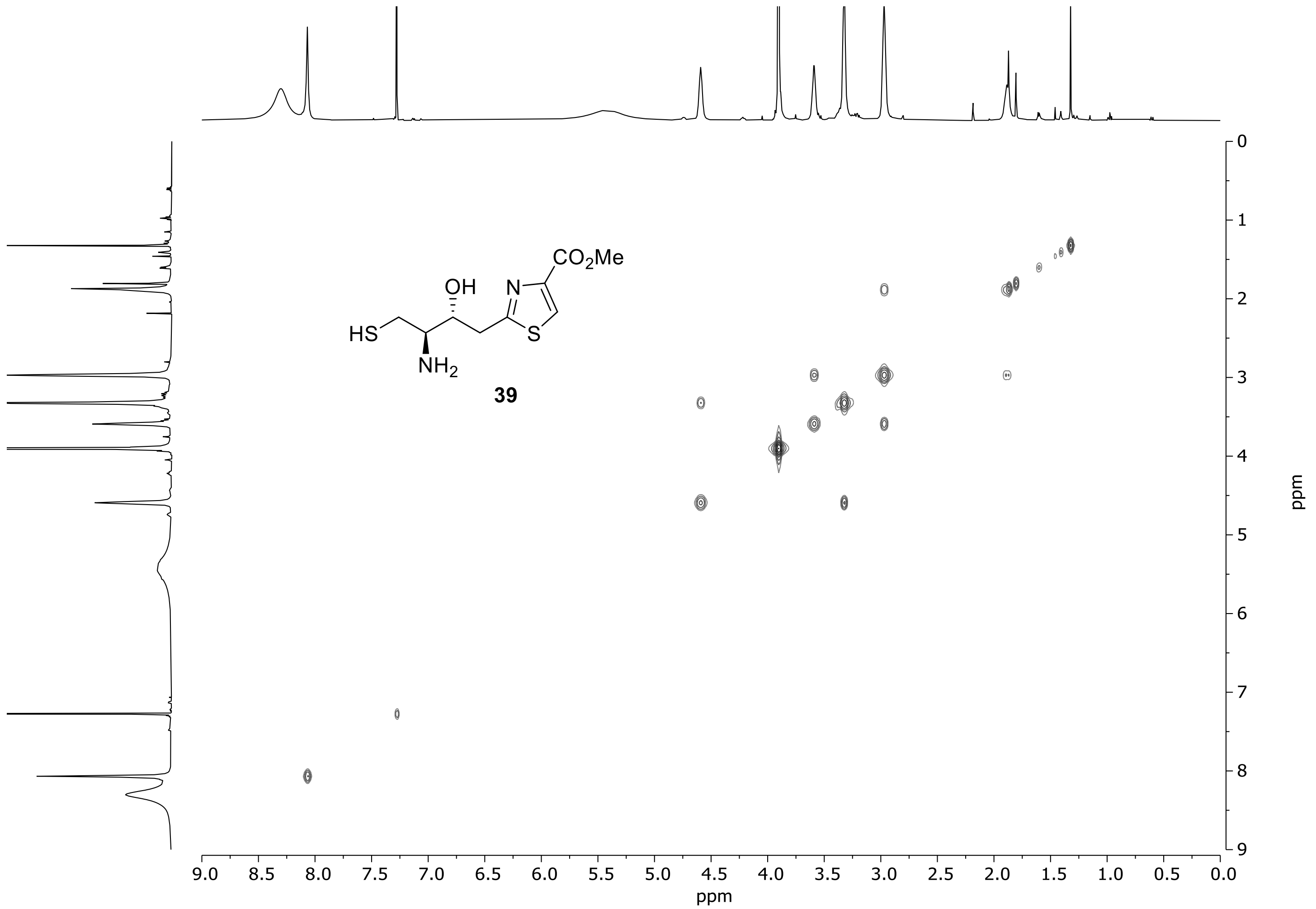

HSQC (500.13 MHz, $CDCl_3$) of **39**

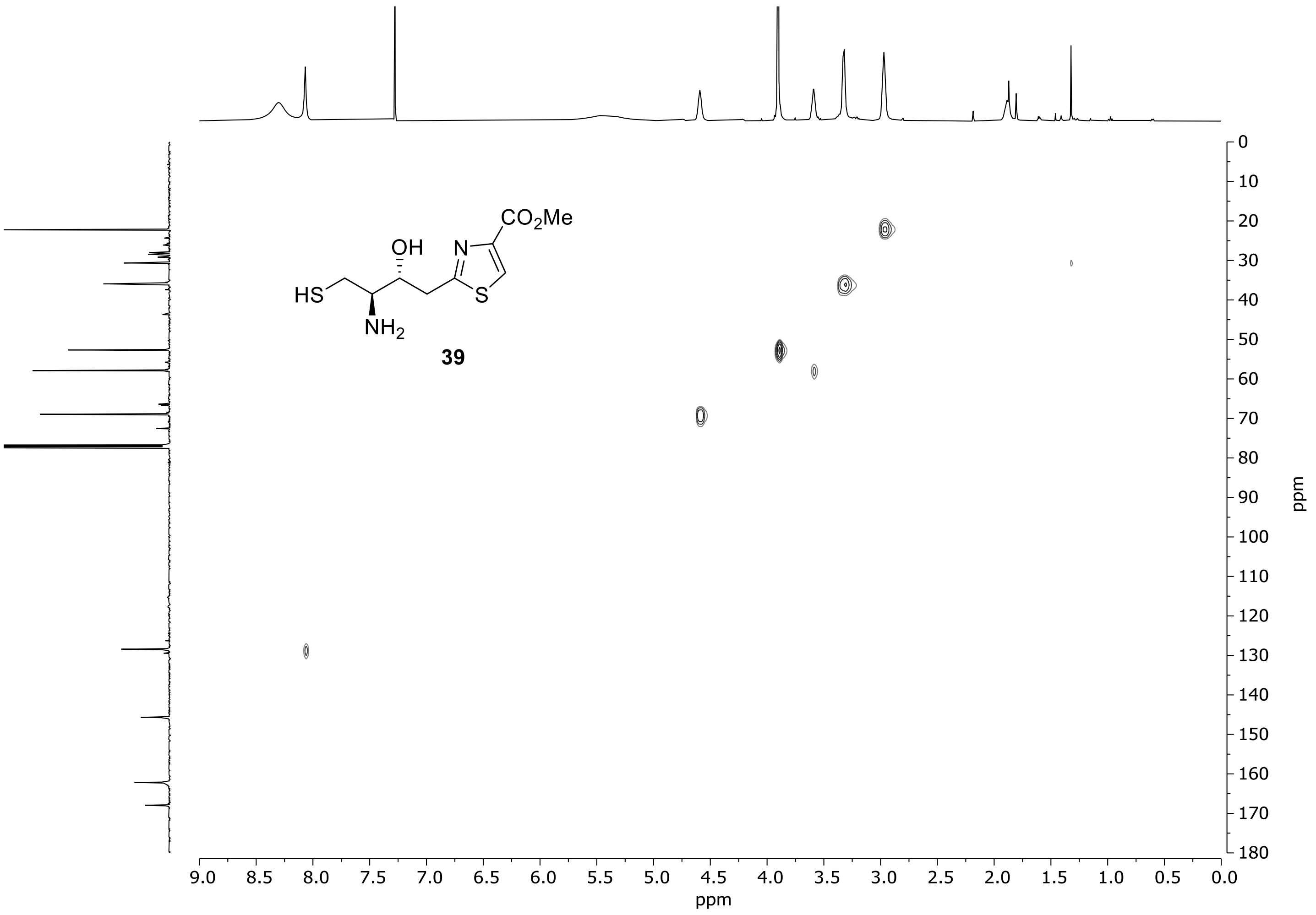

(+)-HRESIMS of **39**: Ion: *m/z*: 263.0520 $[M+H]^+$

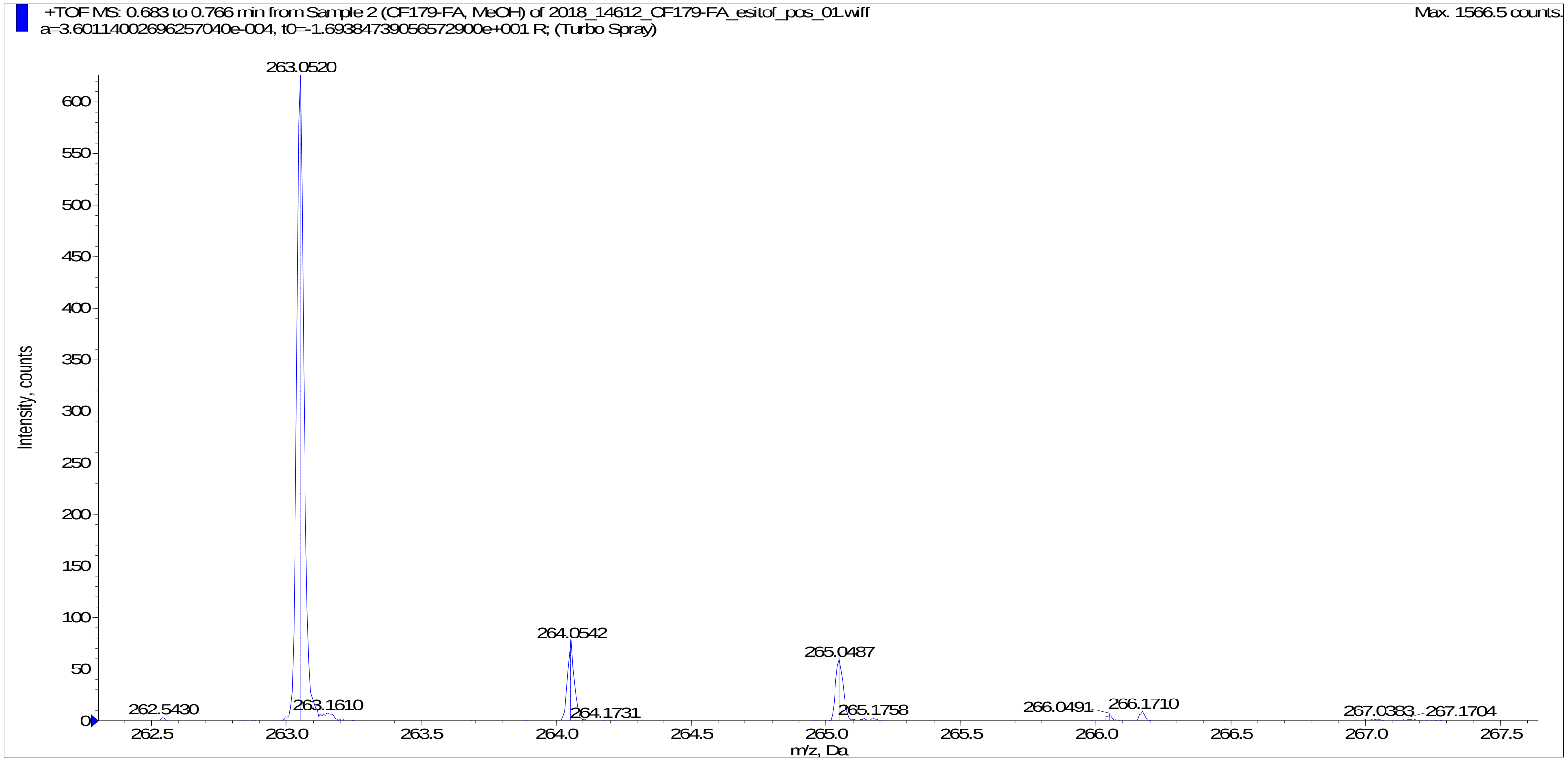

Formula	Calc m/z	Δ, mDa	Δ, ppm	DBE
$C_9H_{15}N_2O_3S_2$	263.0518	0.1374	0.5224	3.5

6. NMR and MS of the synthesis of (13*R*) Pcb thiazole analogues 9a-d and Ga^{3+} complexation attempts (Schemes 8 and 9).

6.1 NMR spectra of 8a

^{1}H NMR (500 MHz; CD_2Cl_2) of **8a**

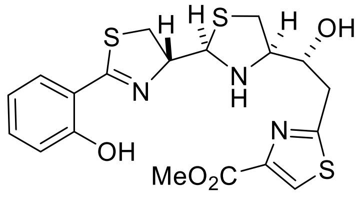

8a

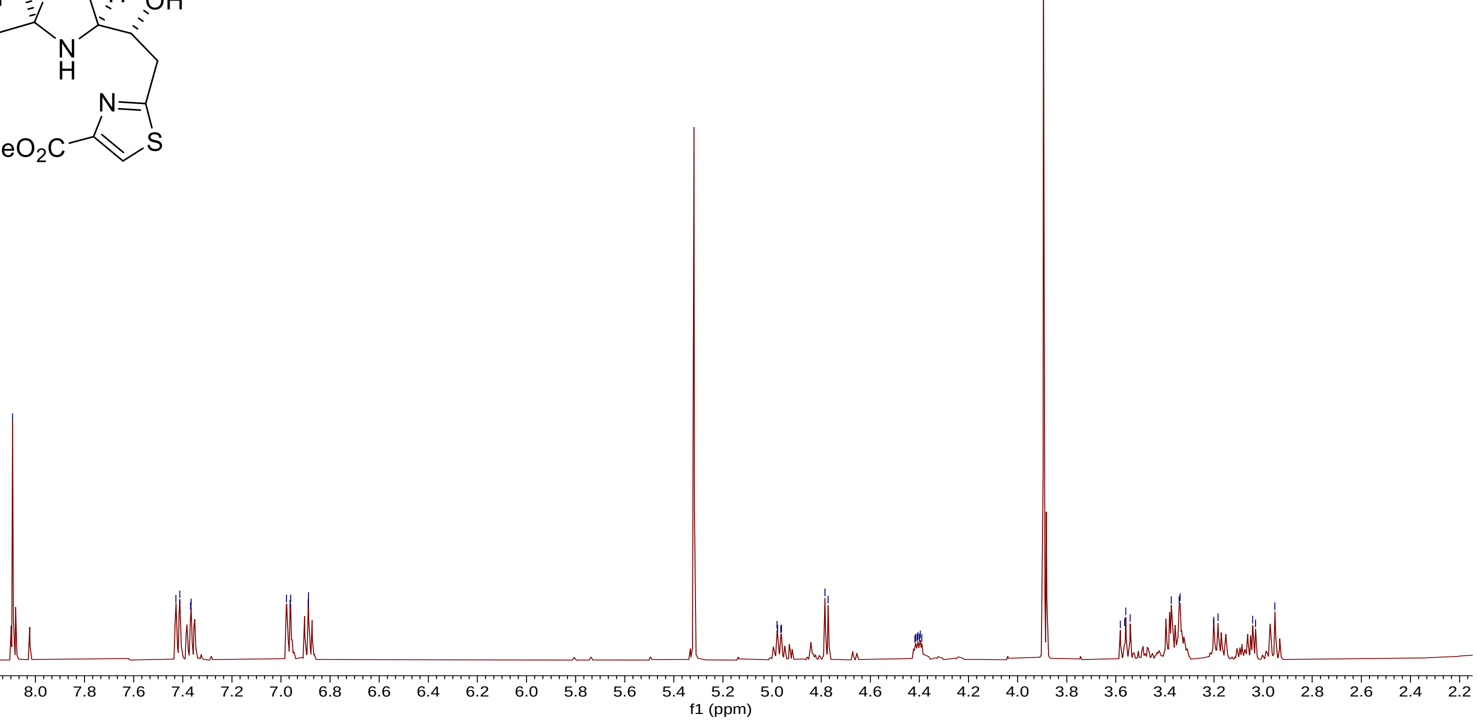

^{13}C NMR (125 MHz) of **8a**

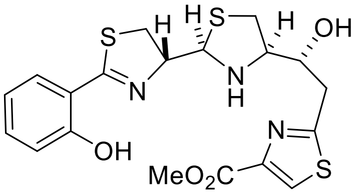

8a

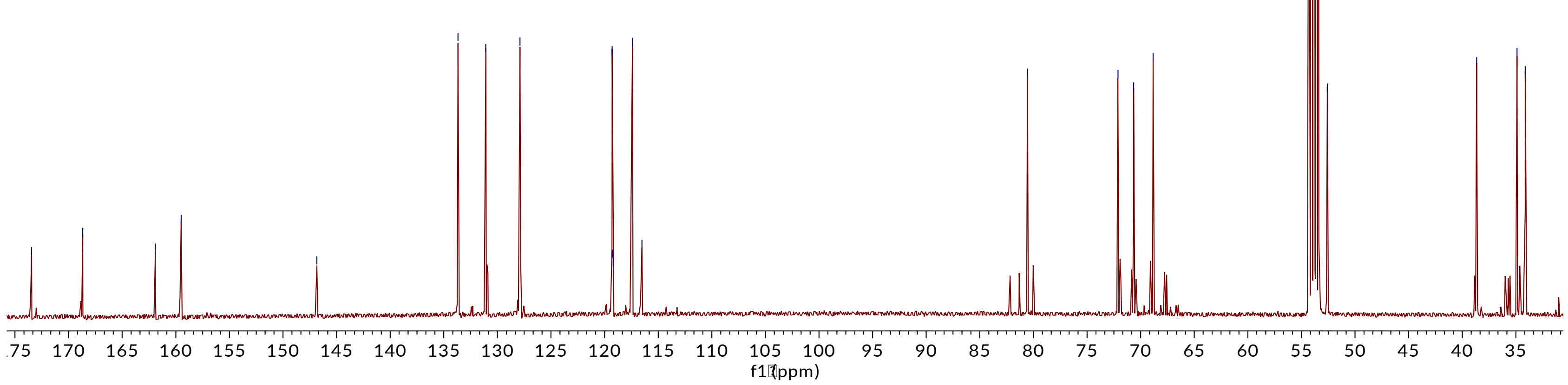

^{1}H-^{1}H COSY (500 MHz) of **8a**

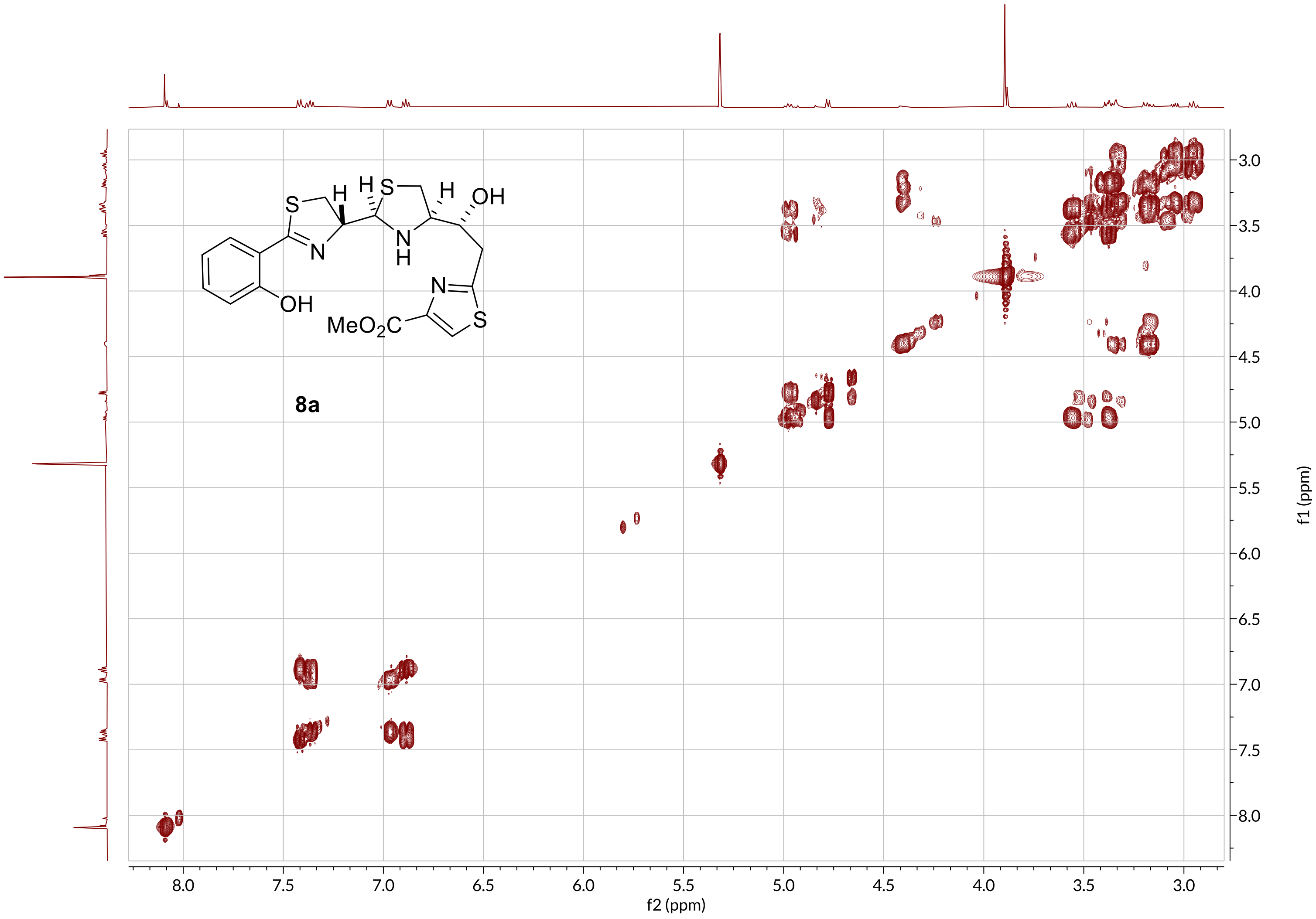

HSQC (500.13 MHz) of **8a**

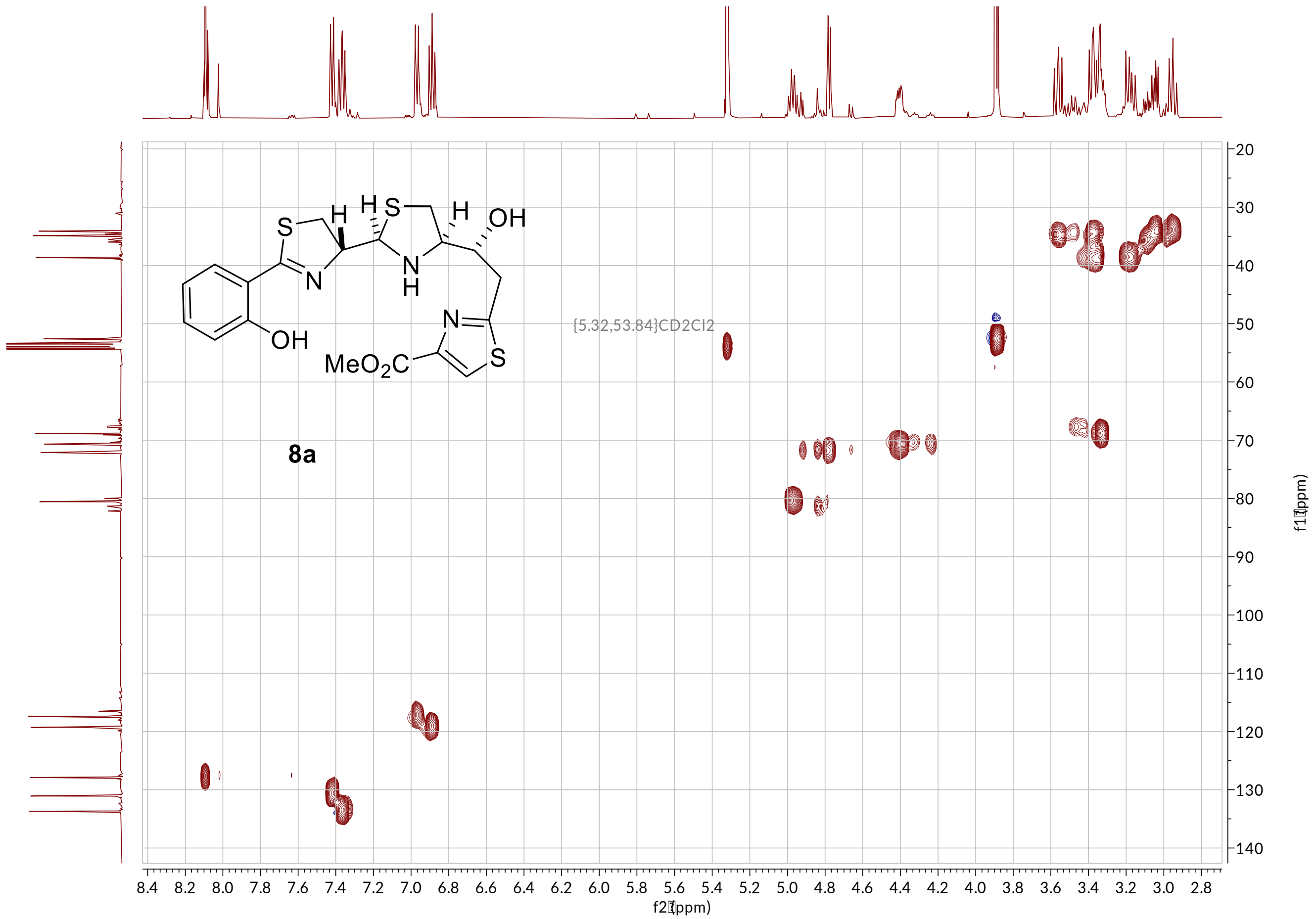

HMBC (500.13 MHz) of **8a**

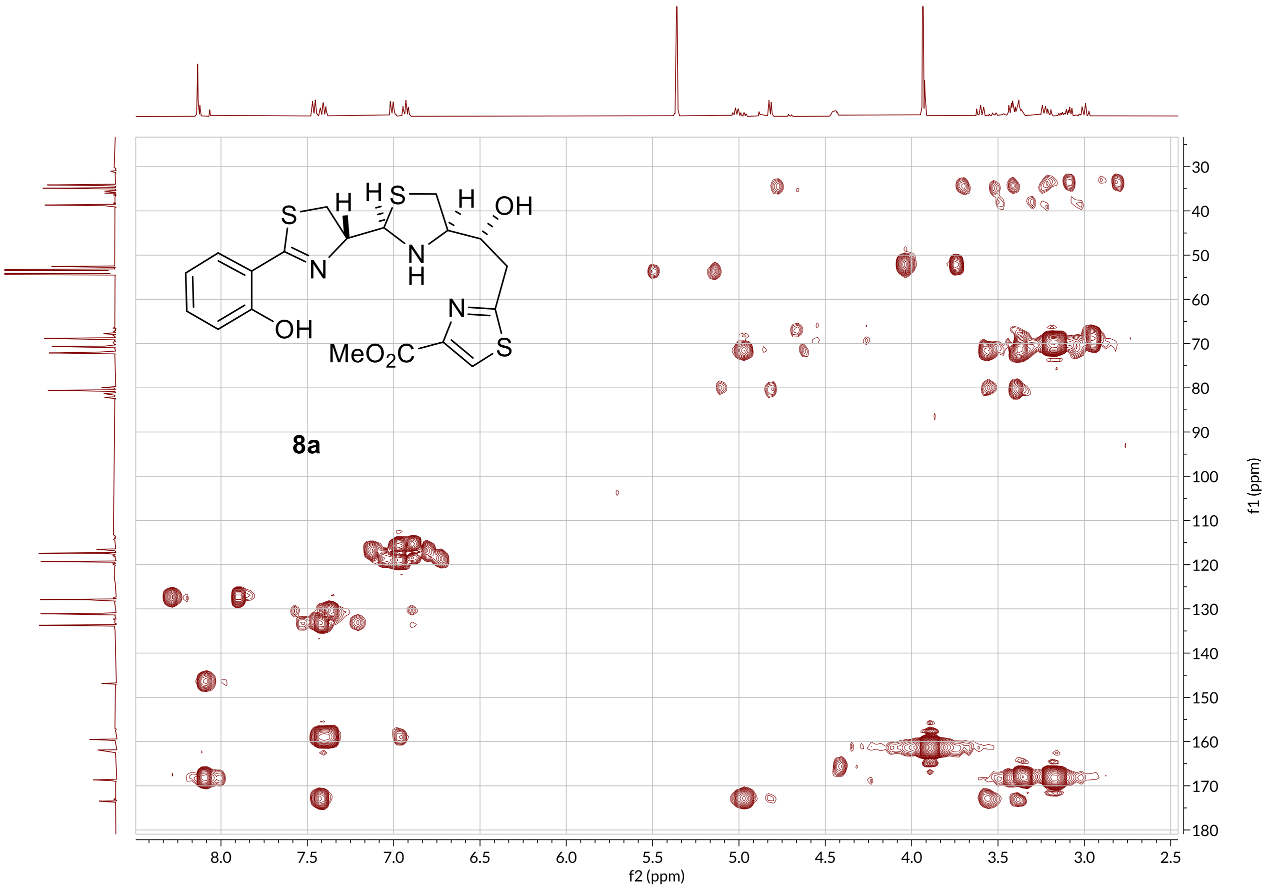

^{1}H-^{1}H NOESY (500.13 MHz) of **8a**

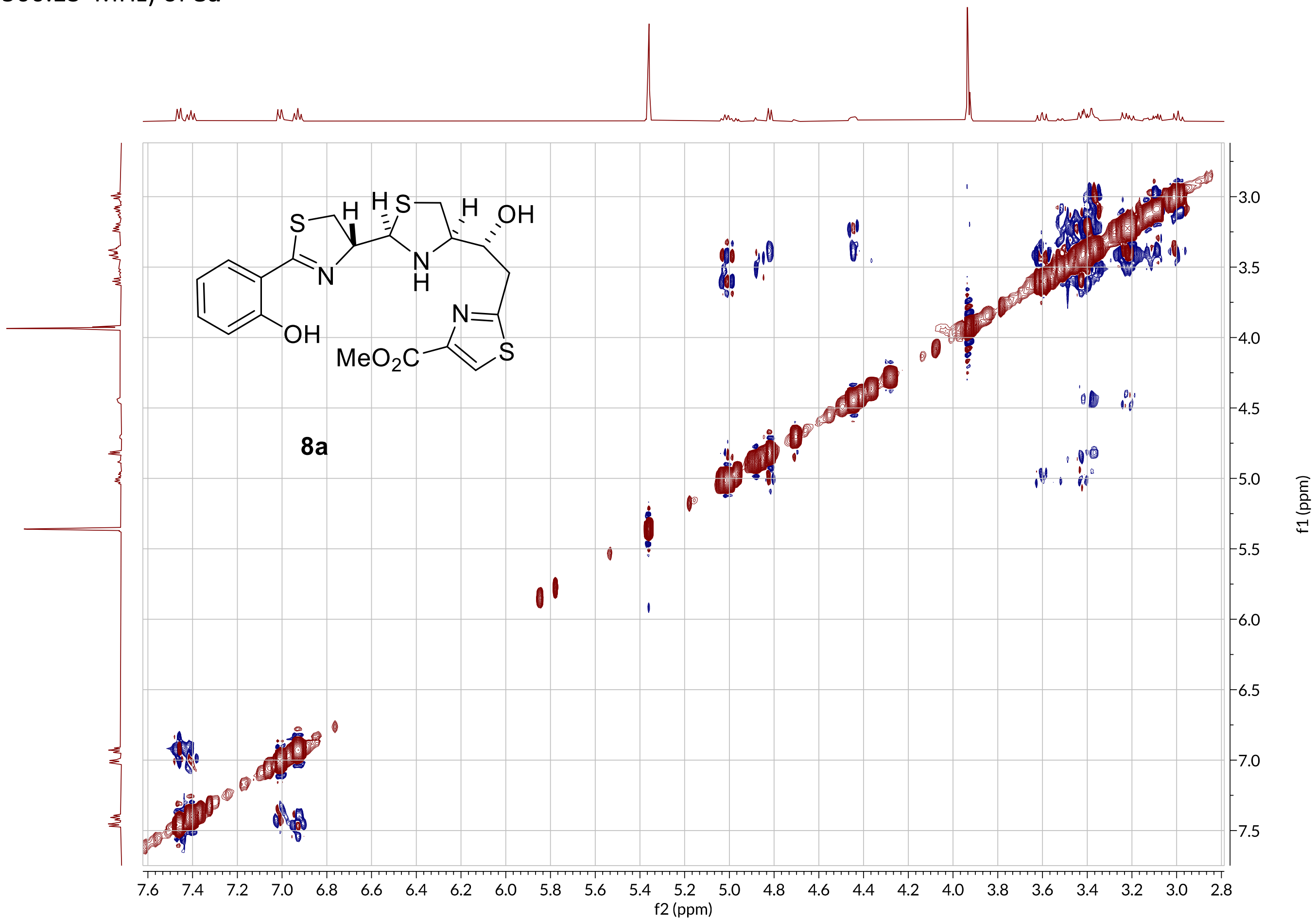

UV spectrum of **8a**

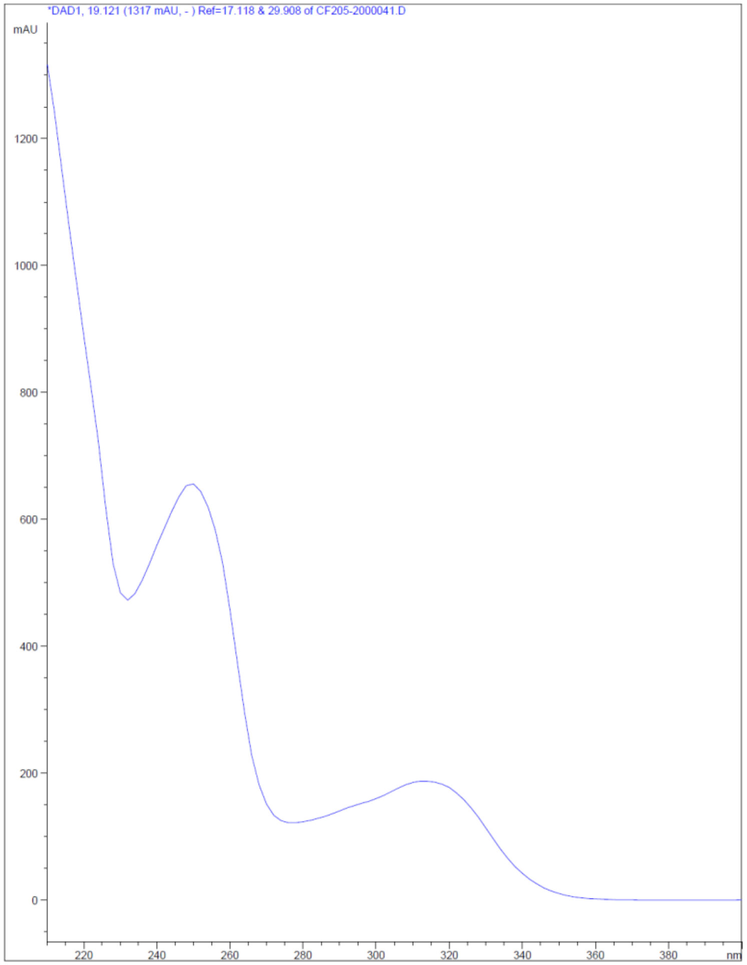

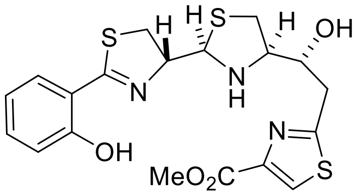

8a

(+)-HRESIMS of **8a**: Ion: *m/z*: 474.0594 $[M+Na]^+$ and 452.0774 $[M+H]^+$

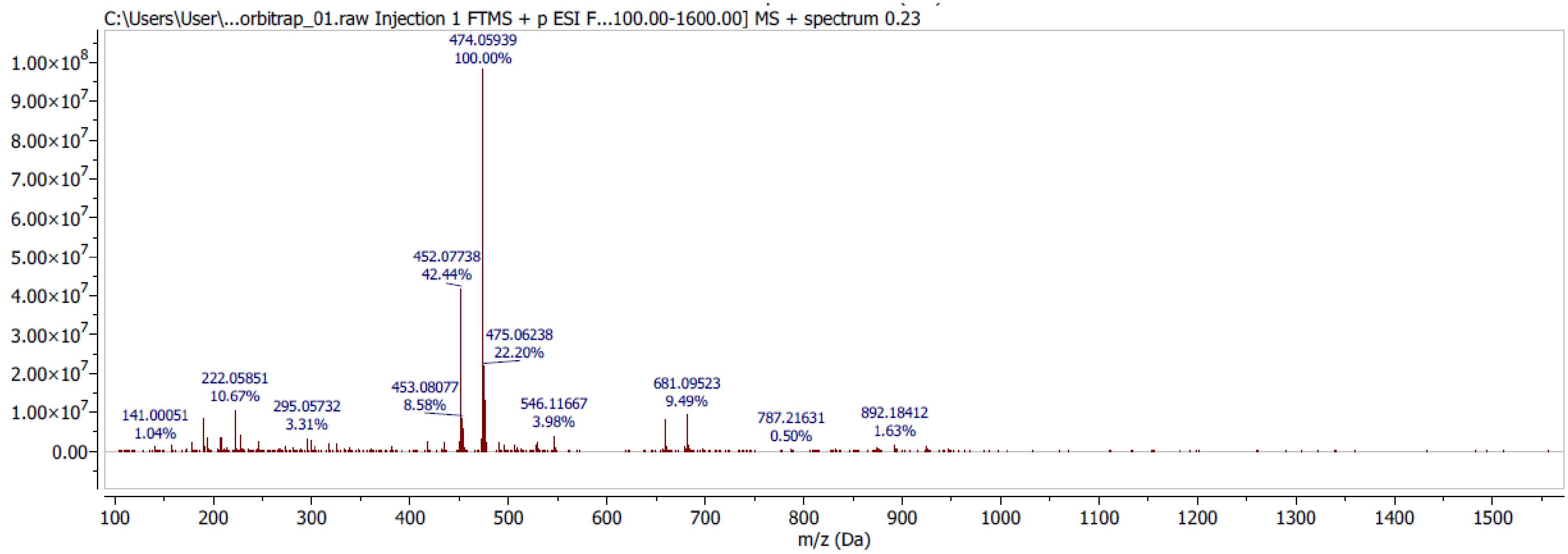

6.2 NMR and MS of NMR spectra of the mixture of (9*S*,10*R*,12*R*,13*R*)-8b and (9*S*,10*S*,12*R*,13*R*)-8c.

^{1}H NMR (500.13 MHz; CD_2Cl_2) of the mixture of **8b** and **8c** (4:3).

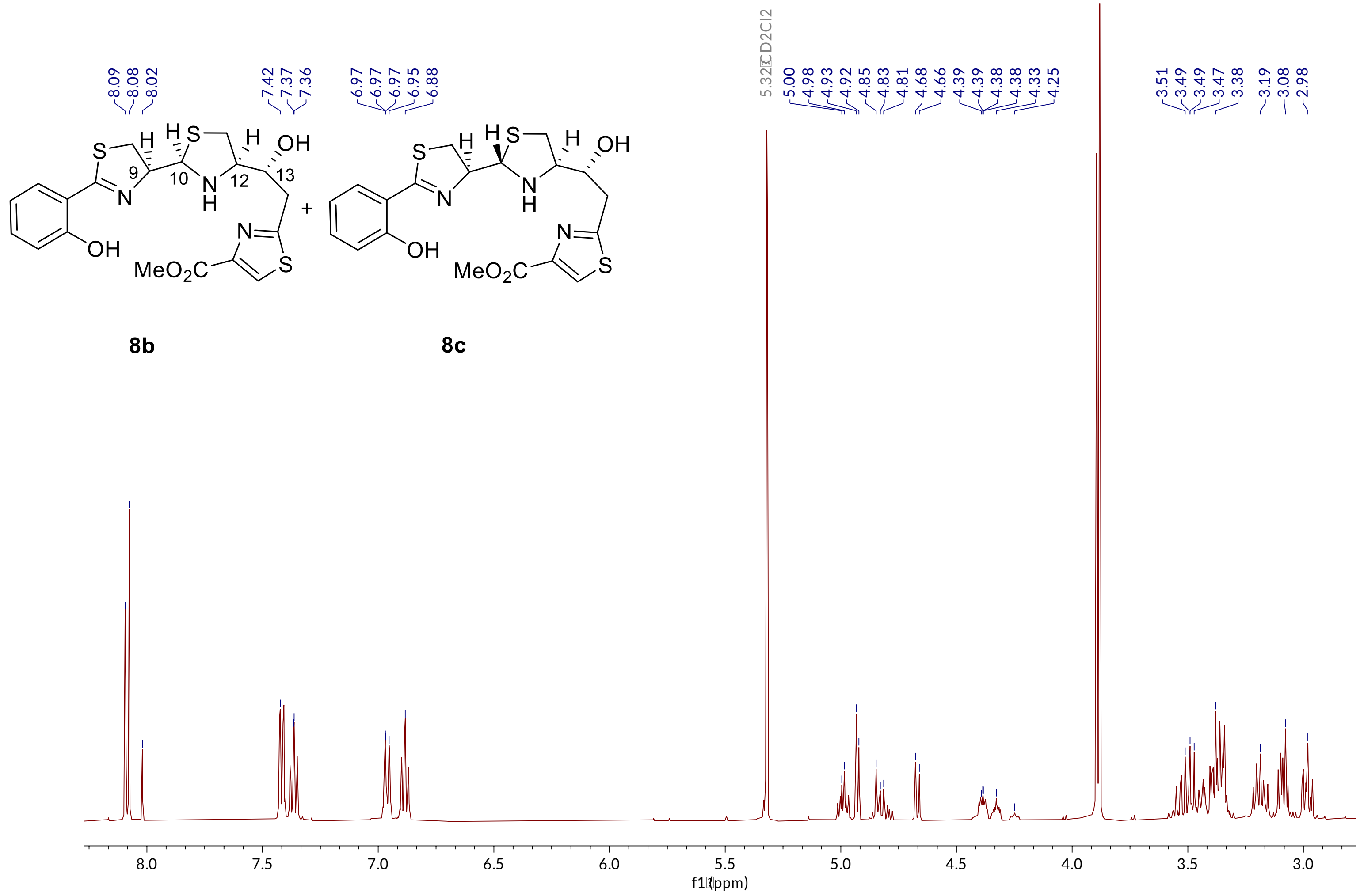

^{13}C NMR (125 MHz; CD_2Cl_2) of the mixture of **8b** and **8c** (4:3).

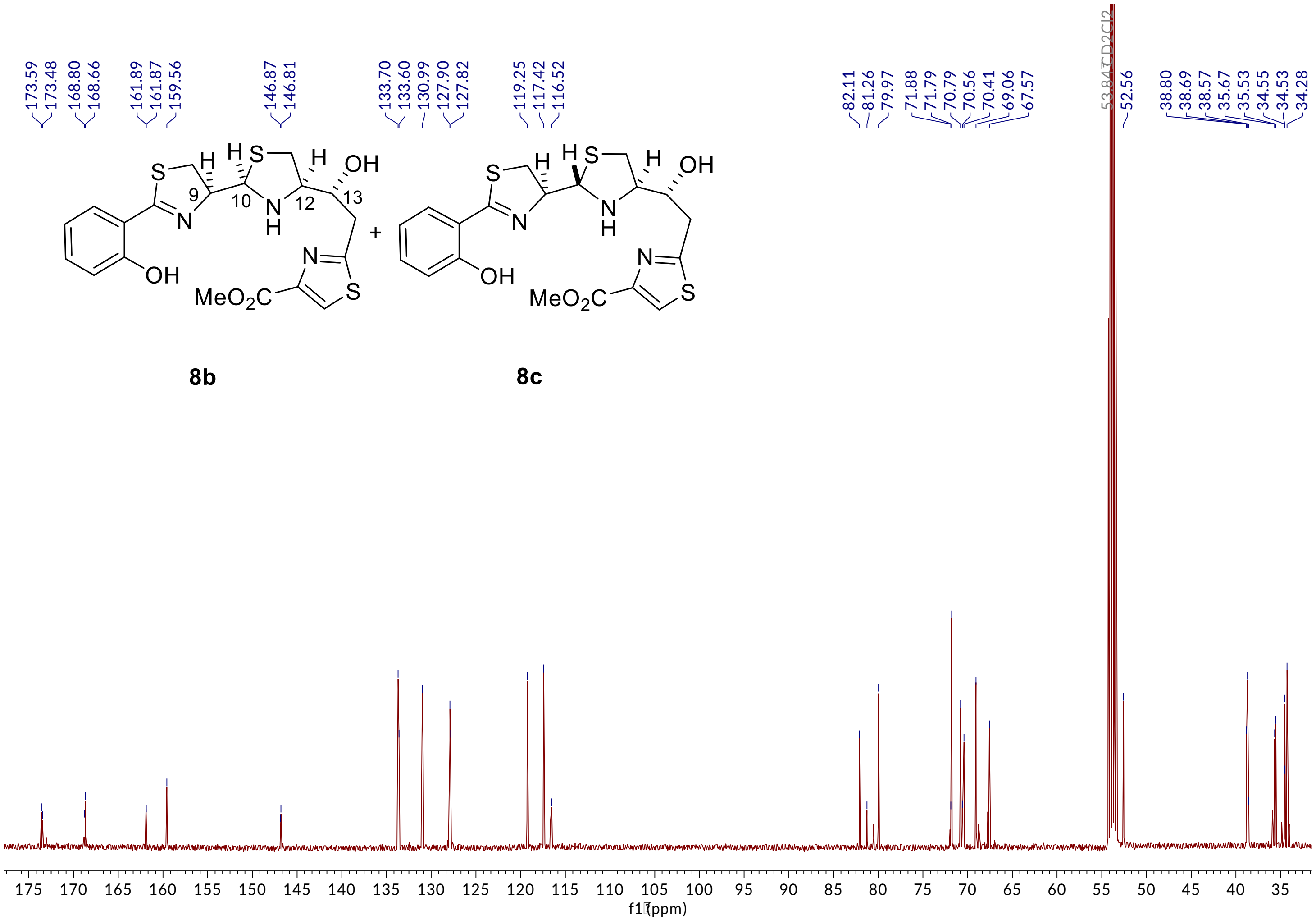

8b 8c

^{1}H-^{1}H COSY (500.13 MHz; CD_2Cl_2) of the mixture of **8b** and **8c** (4:3).

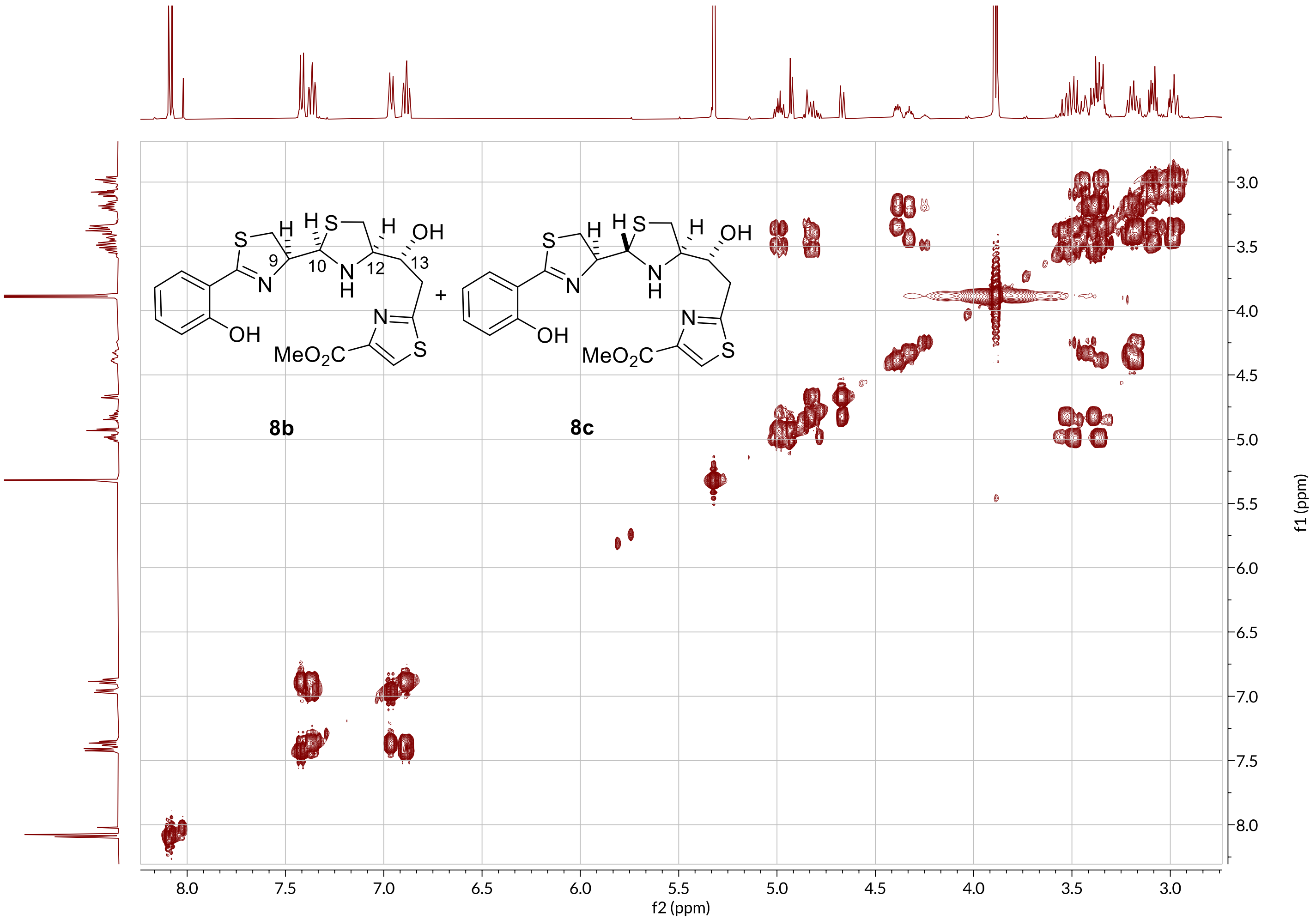

HSQC (500.13 MHz; CD_2Cl_2) of the mixture of **8b** and **8c** (4:3).

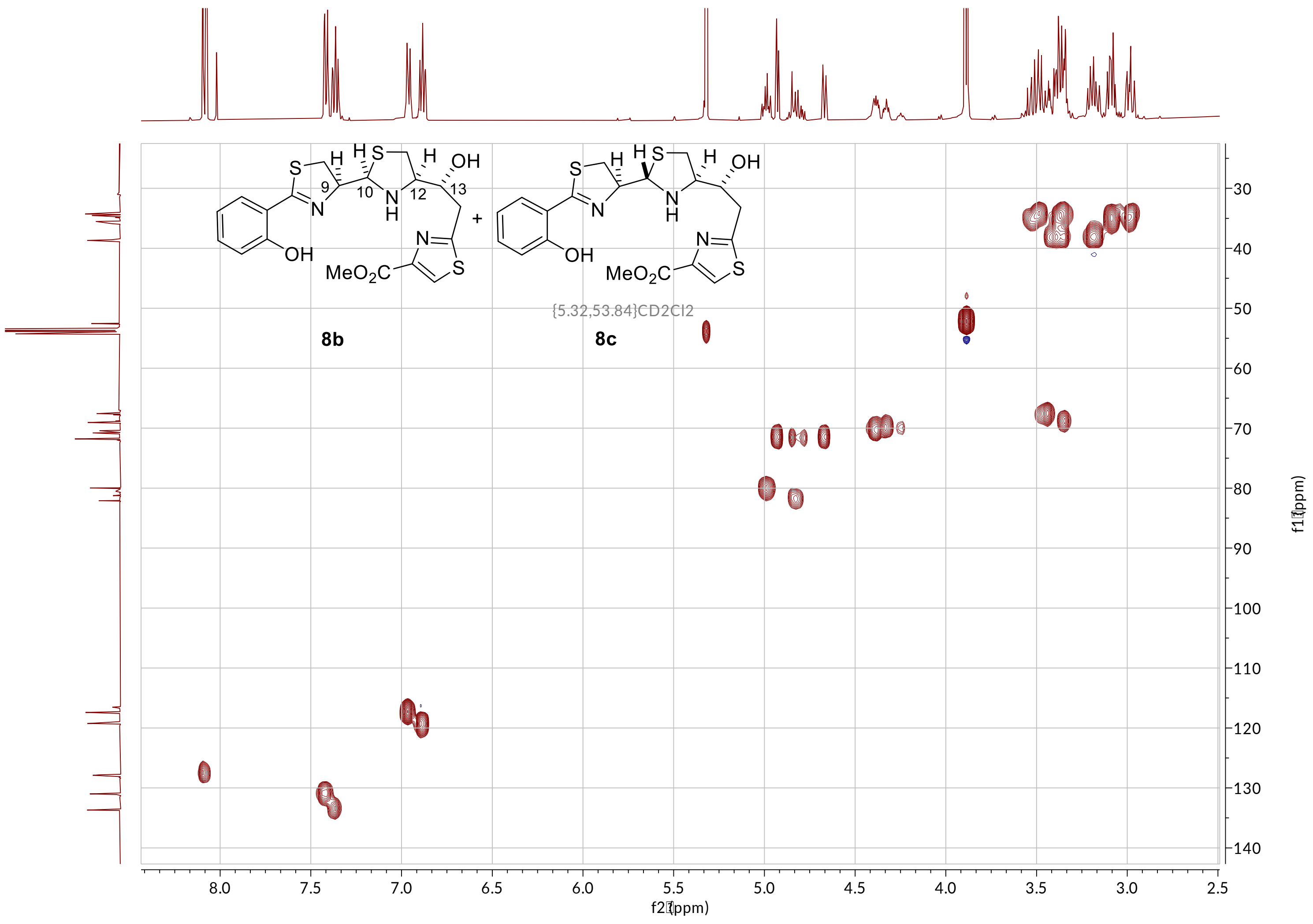

HMBC (500.13 MHz; CD_2Cl_2) of the mixture of **8b** and **8c** (4:3).

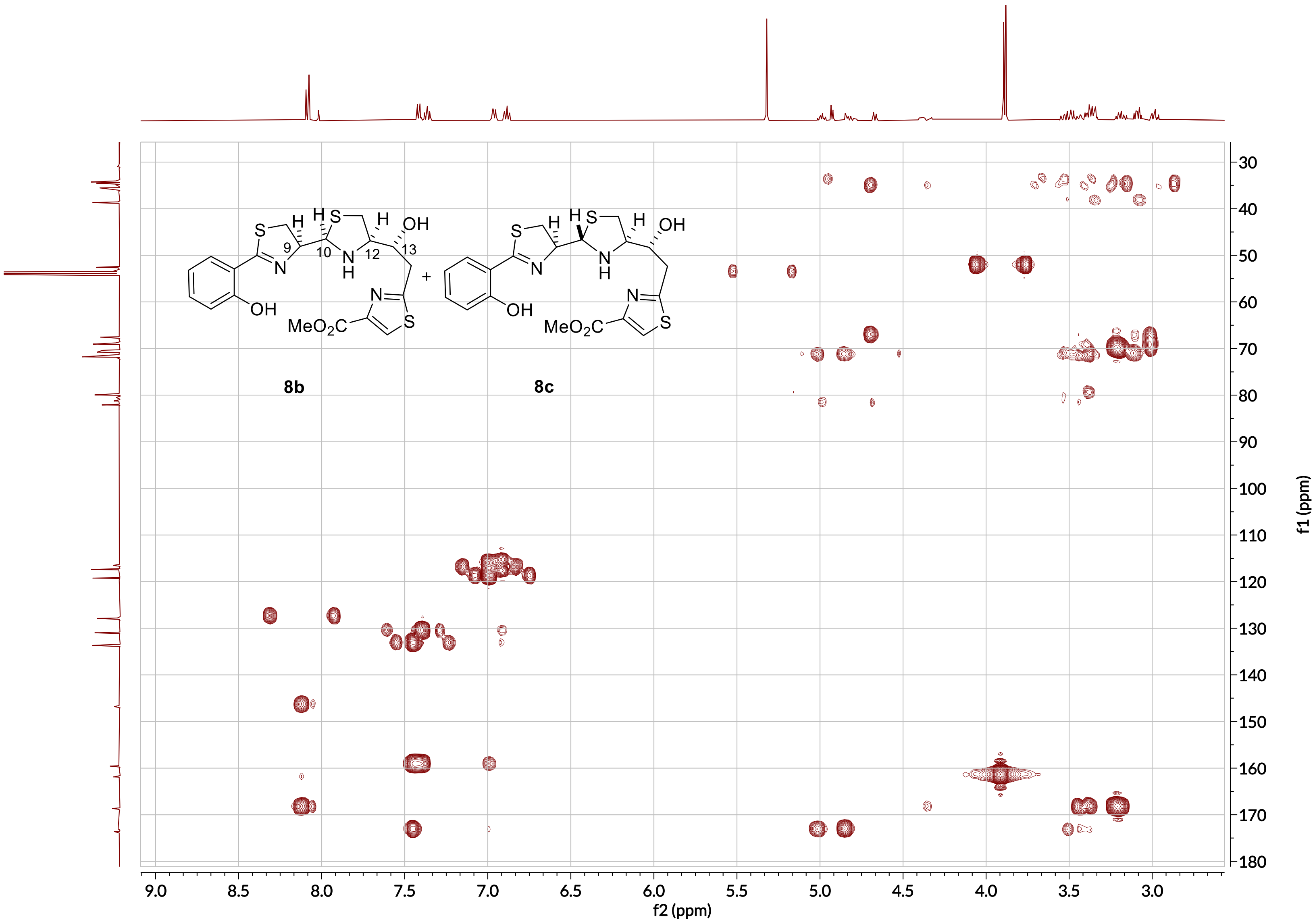

^1H-^1H NOESY (500.13 MHz; CD_2Cl_2) of the mixture of **8b** and **8c** (4:3)

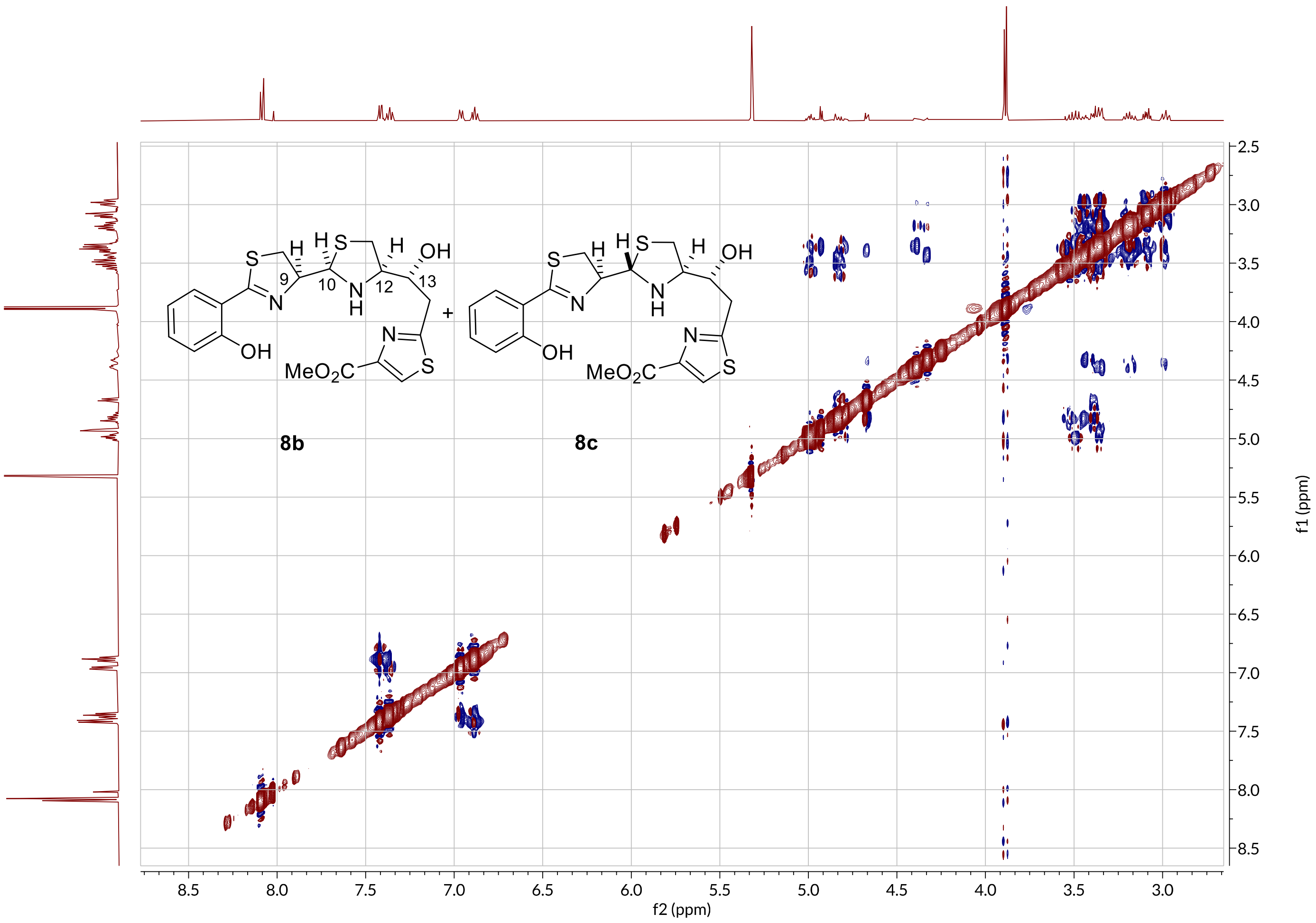

UV spectrum of the mixture **8b** and **8c**

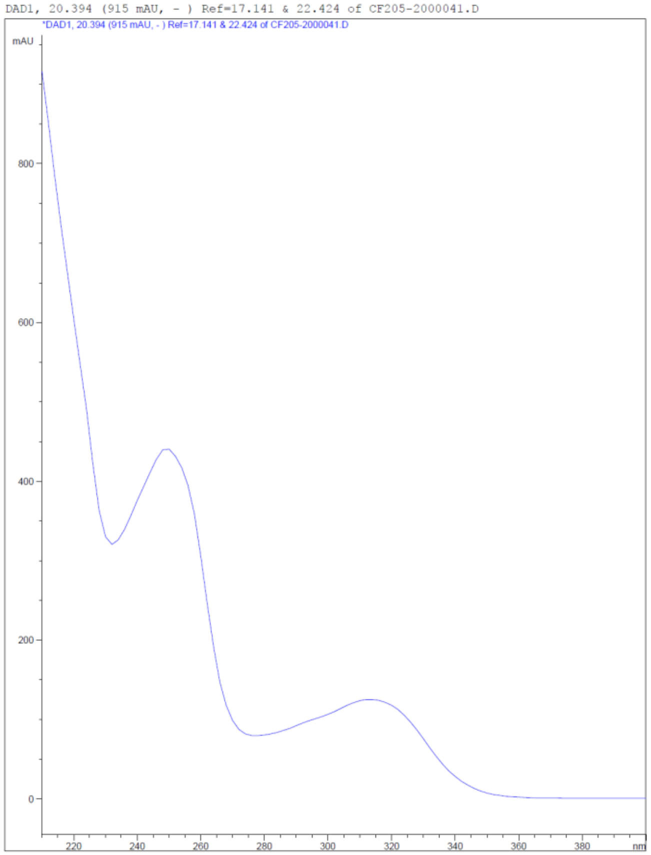

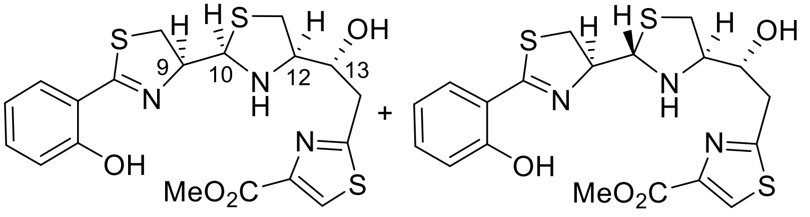

8b **8c**

(+)-HRESIMS of **8b** and **8c**: Ion: *m/z*: 452.0767 $[M+H]^+$

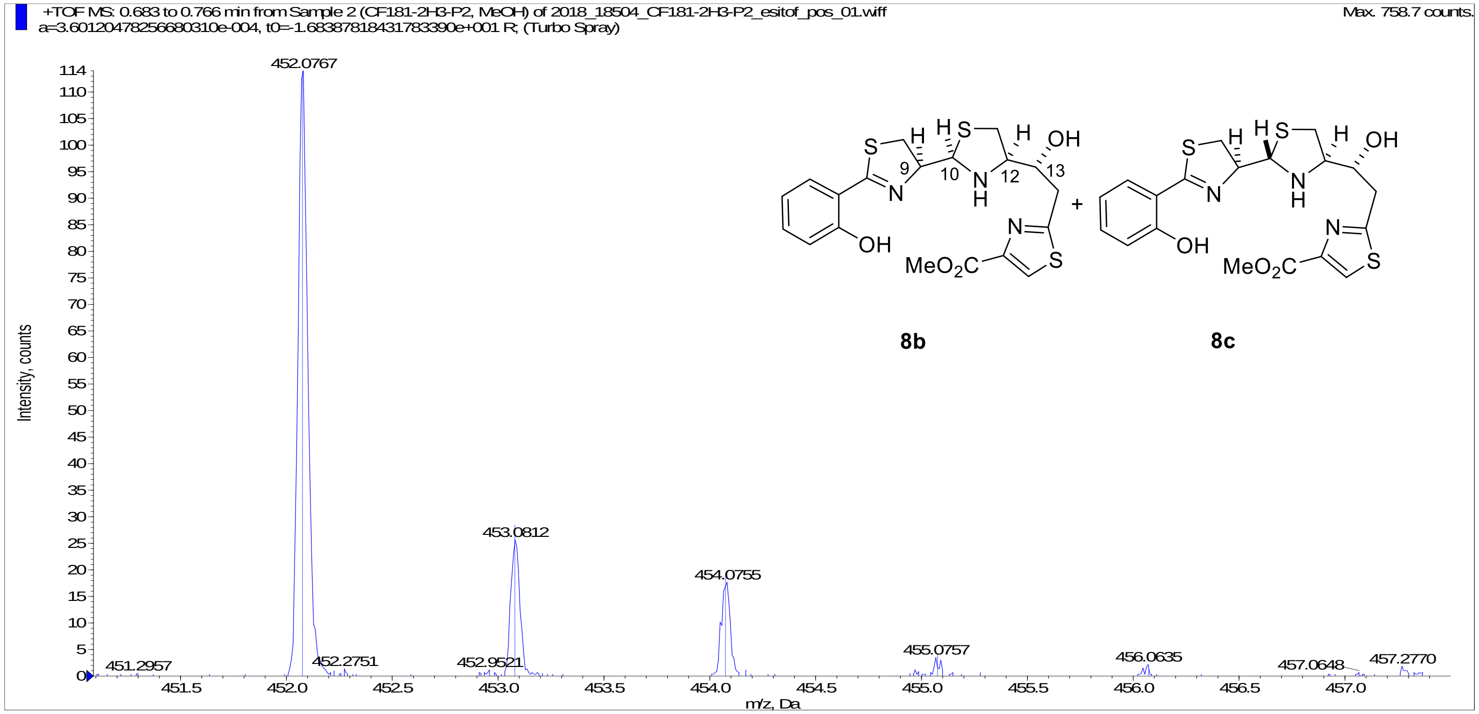

Formula	Calc *m/z*	Δ, mDa	Δ, ppm	DBE
$C_{19}H_{22}N_3O_4S_3$	452.0766	0.0017	0.0037	10.5

6.3 NMR spectra of the mixture of 9a and 9d

^{1}H NMR (500.13 MHz) in CD_3OD of the mixture **9a** and **9d** (1:2)

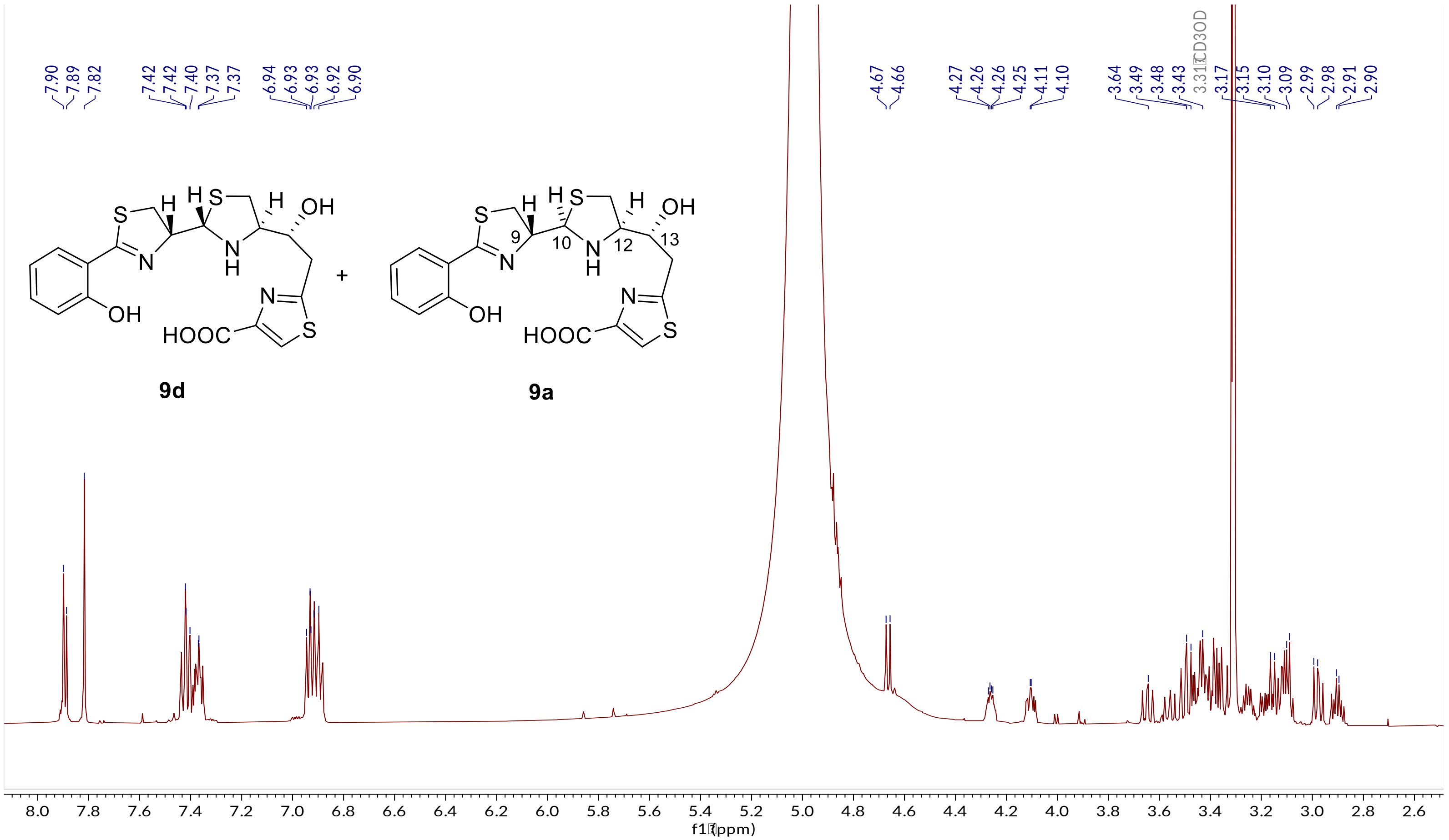

^{13}C NMR (125 MHz) in CD_3OD of the mixture **9a** and **9d** (1:2)

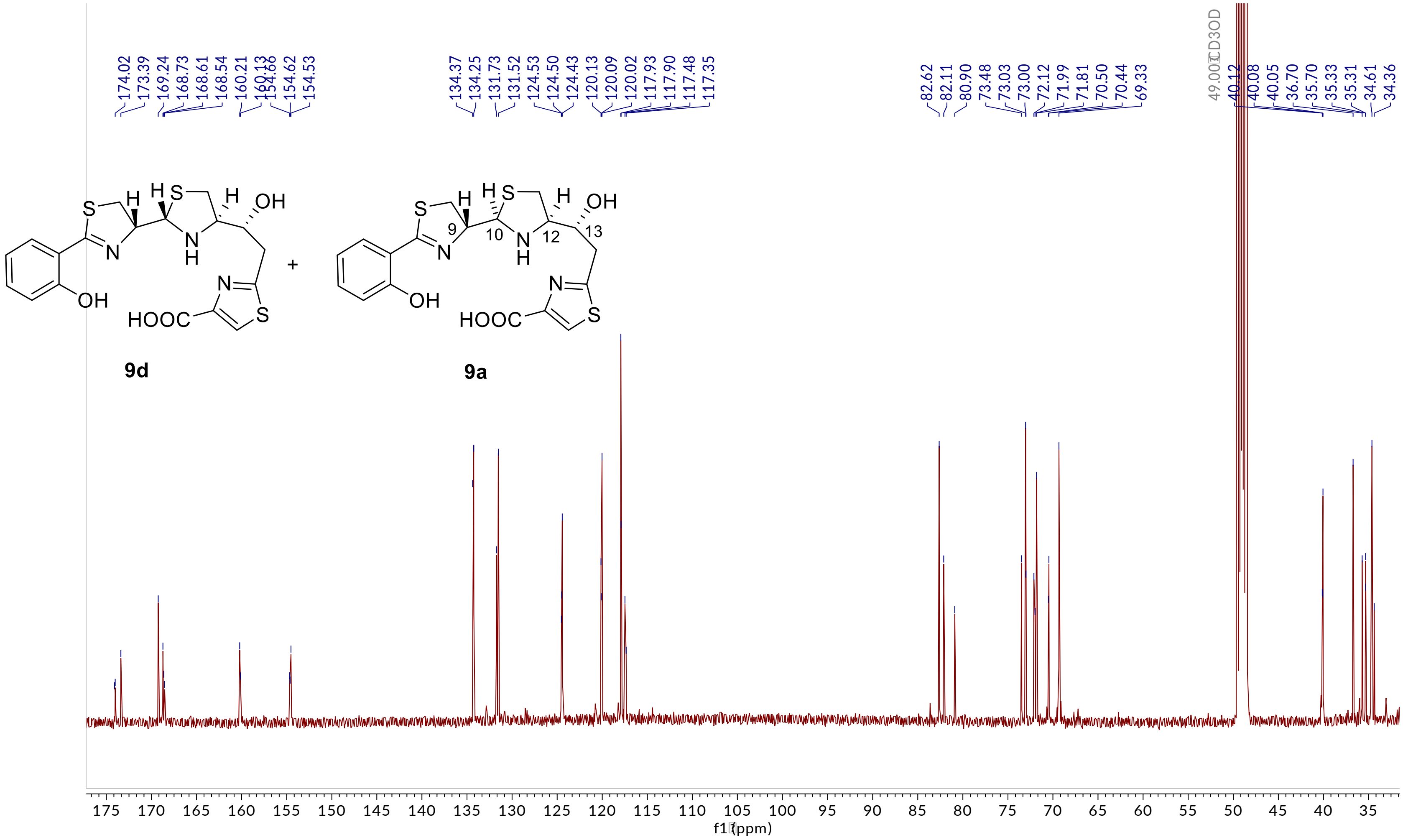

^{1}H-^{1}H COSY (500.13 MHz) in CD_3OD of the mixture **9a** and **9d** (1:2)

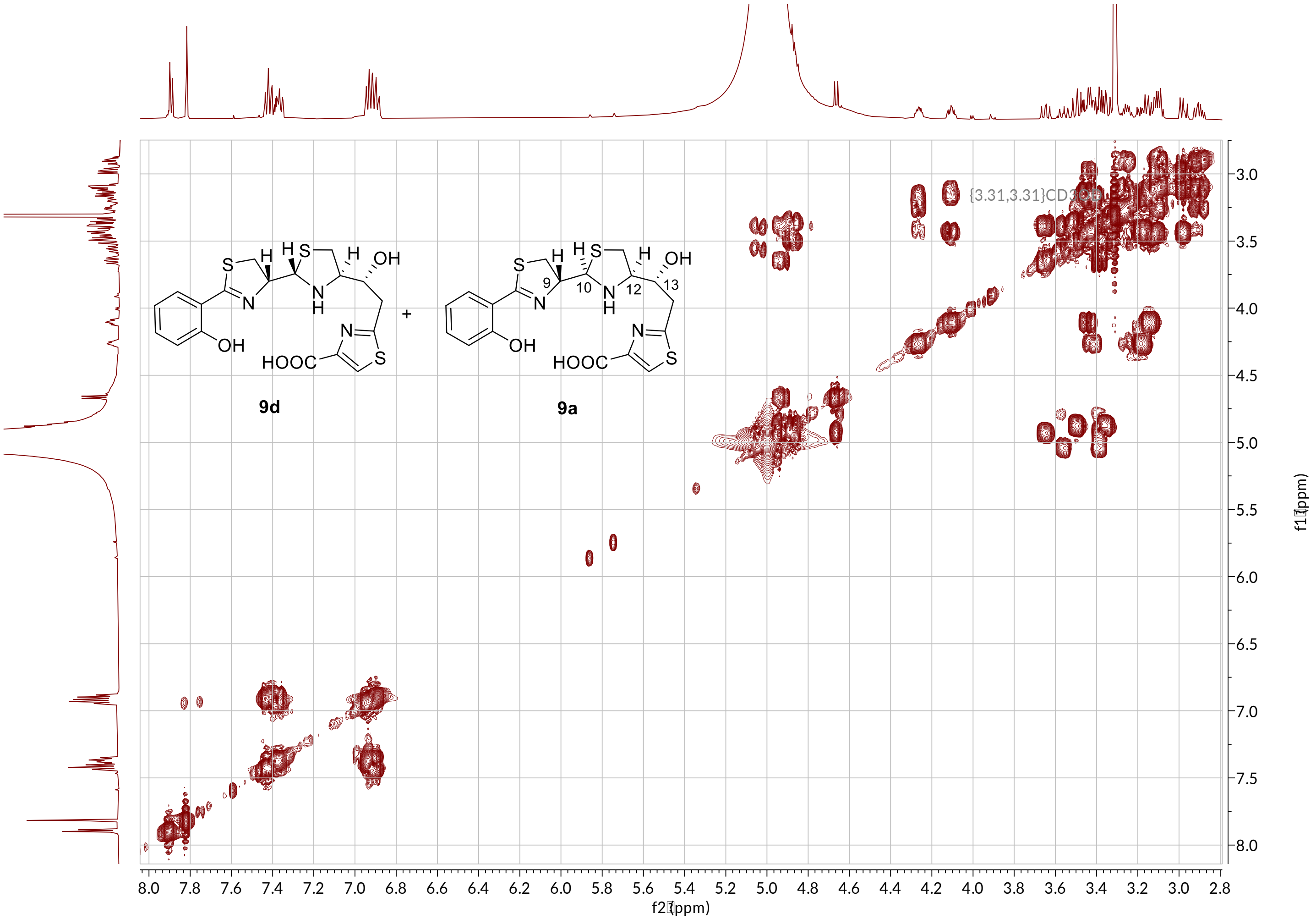

HSQC (500.13 MHz) in CD_3OD of the mixture **9a** and **9d** (1:2)

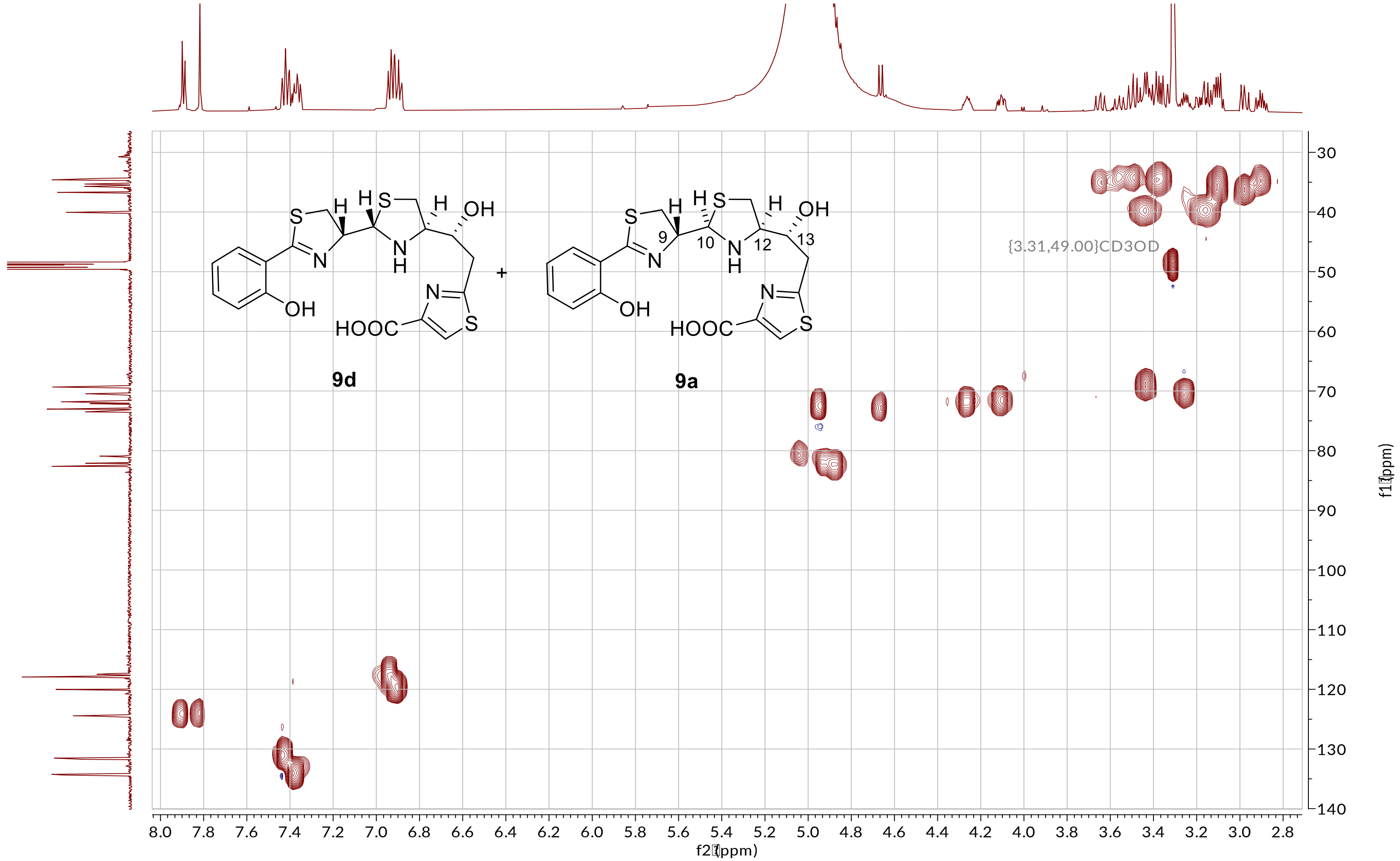

(+)-HRESIMS of the mixture of **9a** and **9d**: Ion: *m/z*: 438.0610 [M+H]$^{+}$

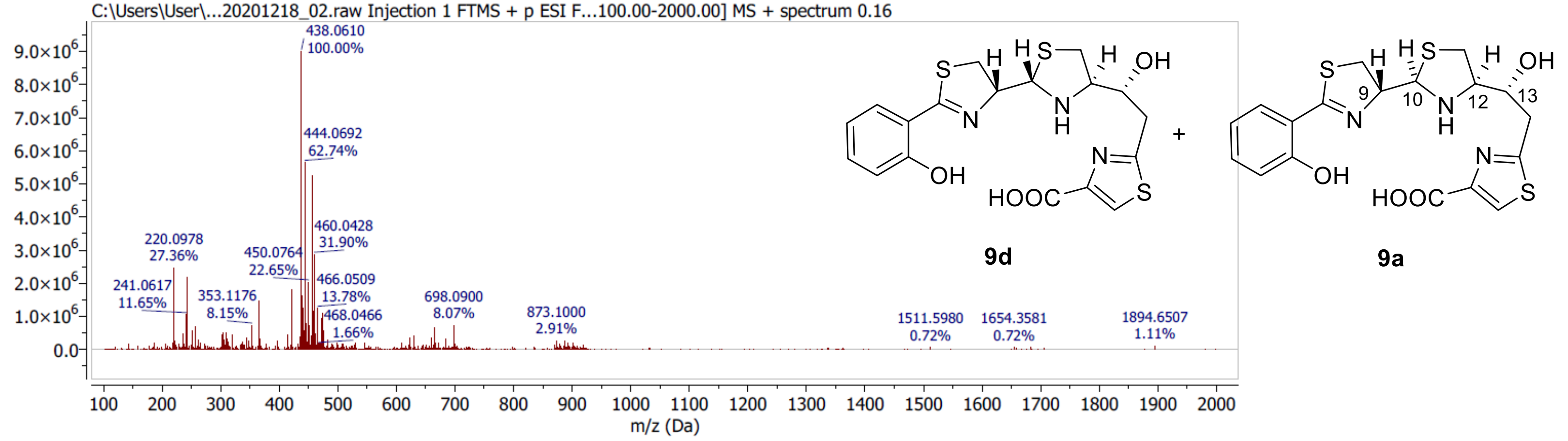

(-)-HRESIMS of the mixture of **9a** and **9d**: Ion: *m/z*: 436.0457 [M-H]$^{-}$

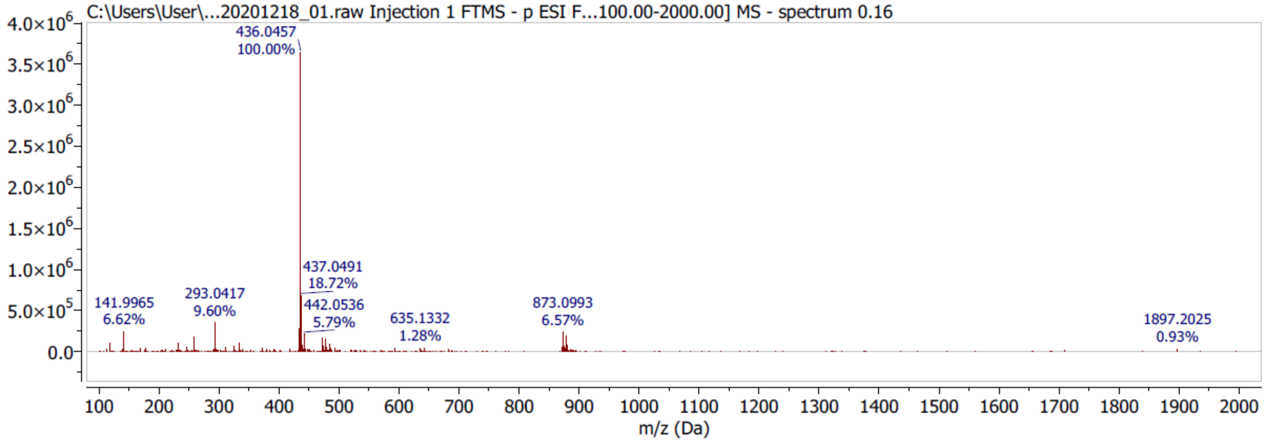

6.4 NMR spectra of the mixture of 9b and 9c

^{1}H NMR (500.13 MHz) in CD_3OD of the mixture of **9b** and **9c** (3.5:1)

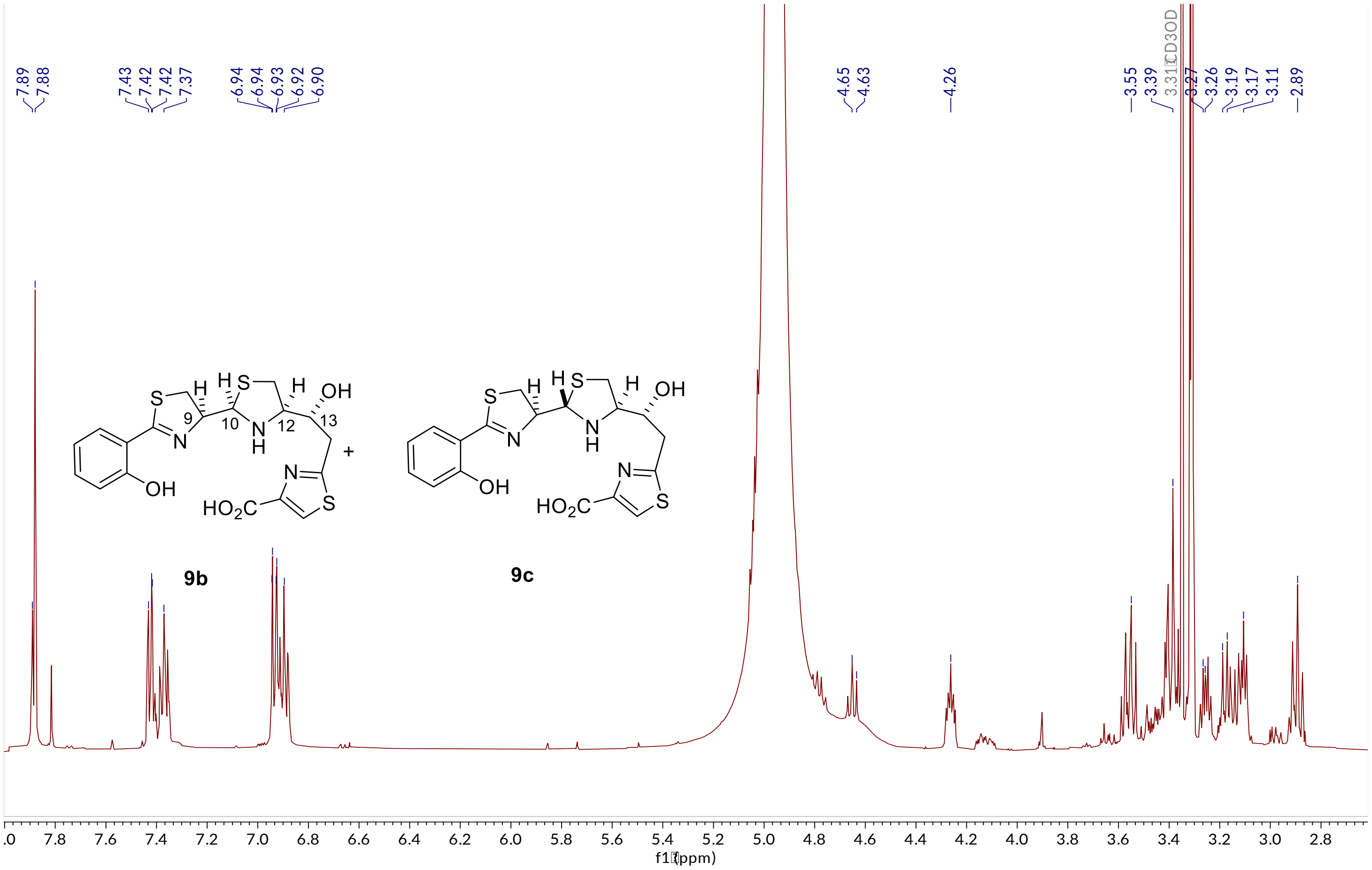

^{13}C NMR (125 MHz) in CD_3OD of the mixture of **9b** and **9c** (3.5:1)

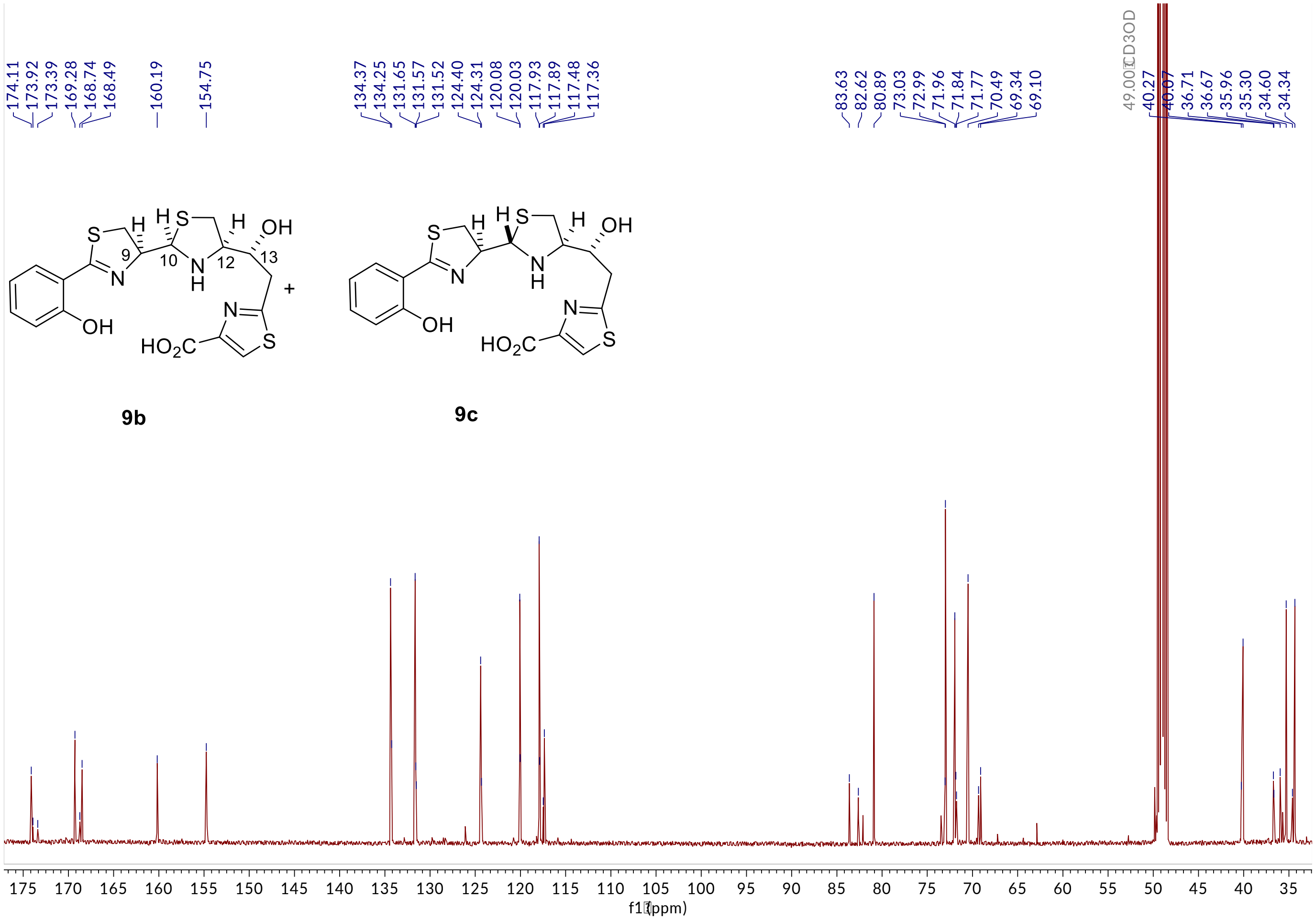

^{1}H-^{1}H COSY (500.13 MHz) in CD_3OD of the mixture of **9b** and **9c**(3.5:1)

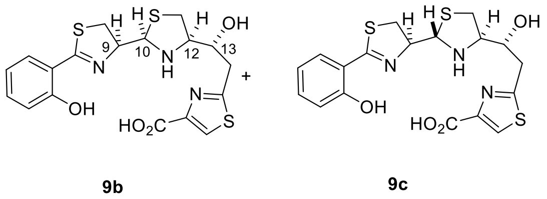

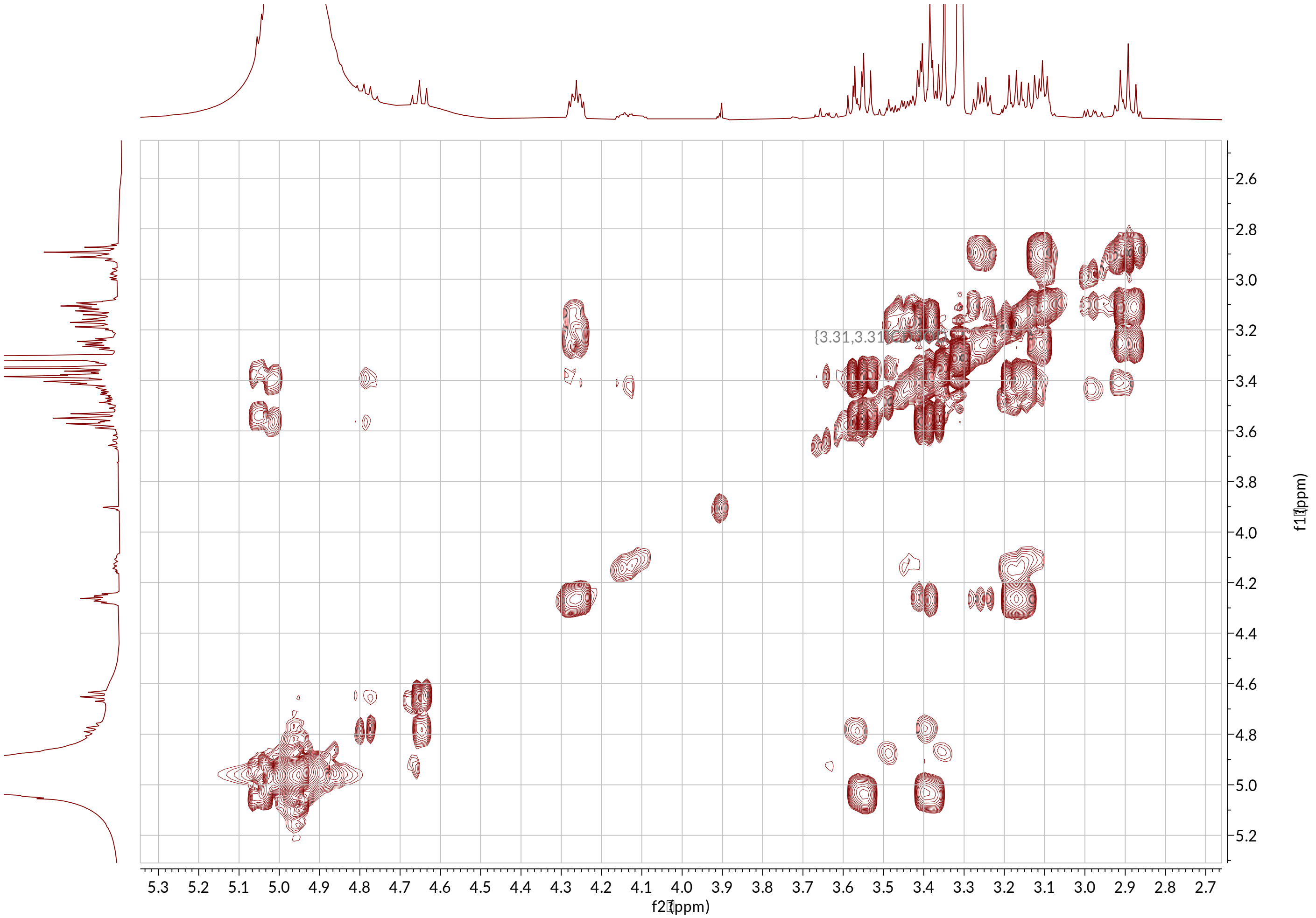

HSQC (500.13 MHz) in CD_3OD of the mixture of **9b** and **9c** (3.5:1)

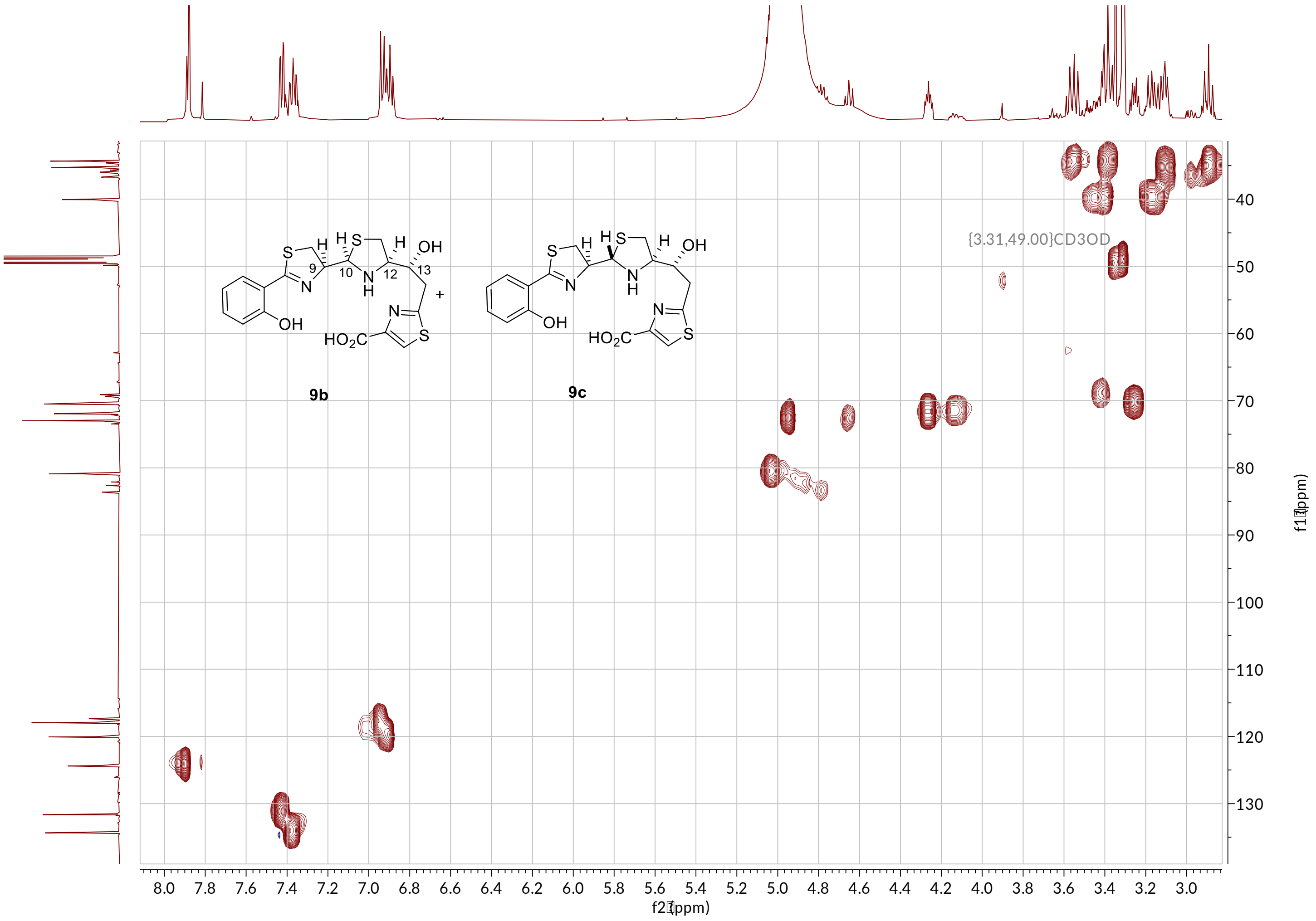

^1H-^1H NOESY (500.13 MHz) in CD_3OD of the mixture of **9b** and **9c** (3.5:1)

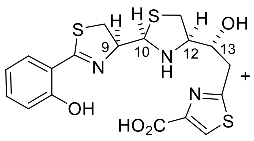

9b

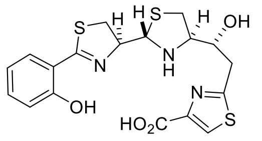

9c

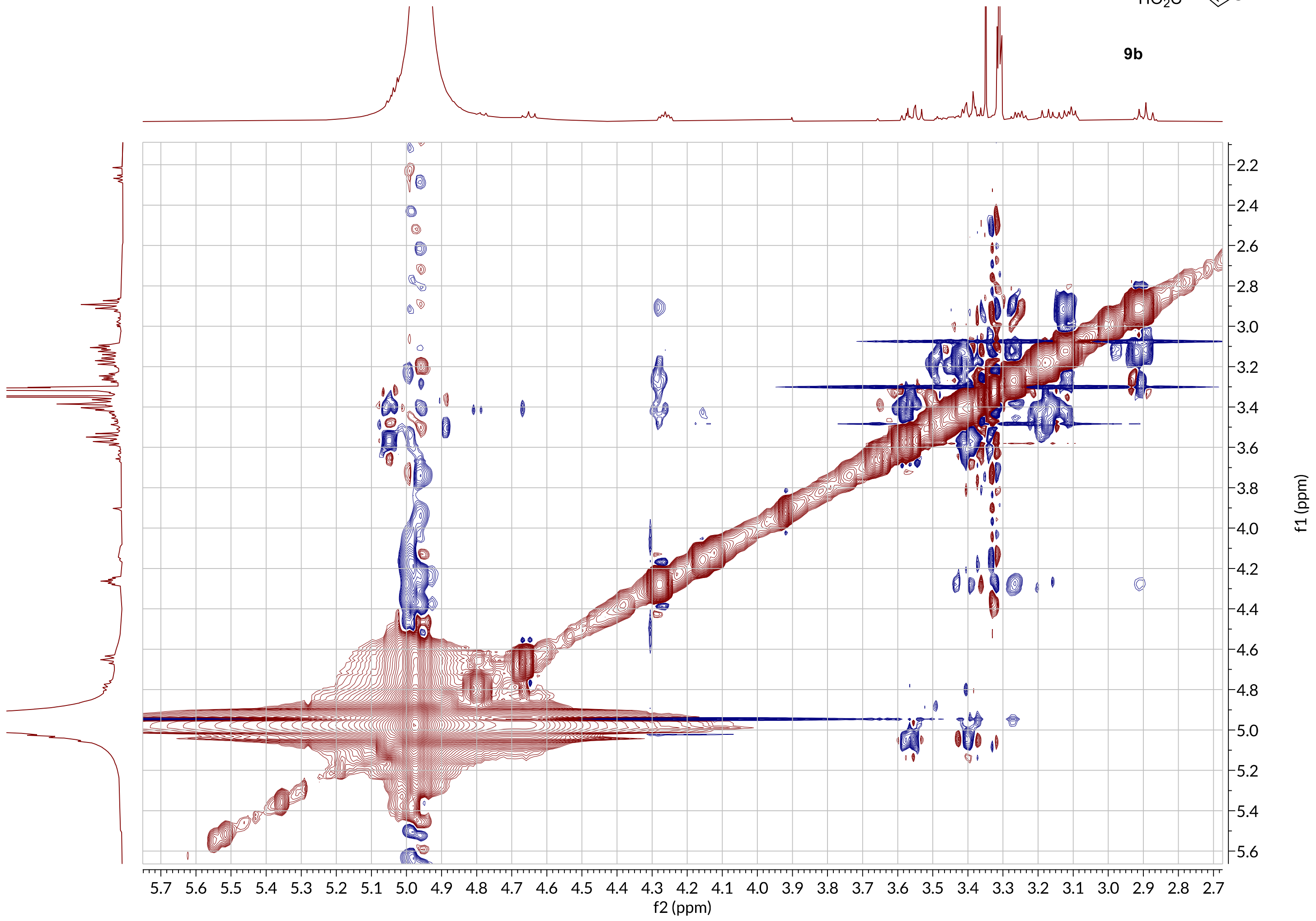

(-)-HRESIMS of the mixture of **9b** and **9c**: Ion: *m/z*: 436.0468 [M-H]$^-$

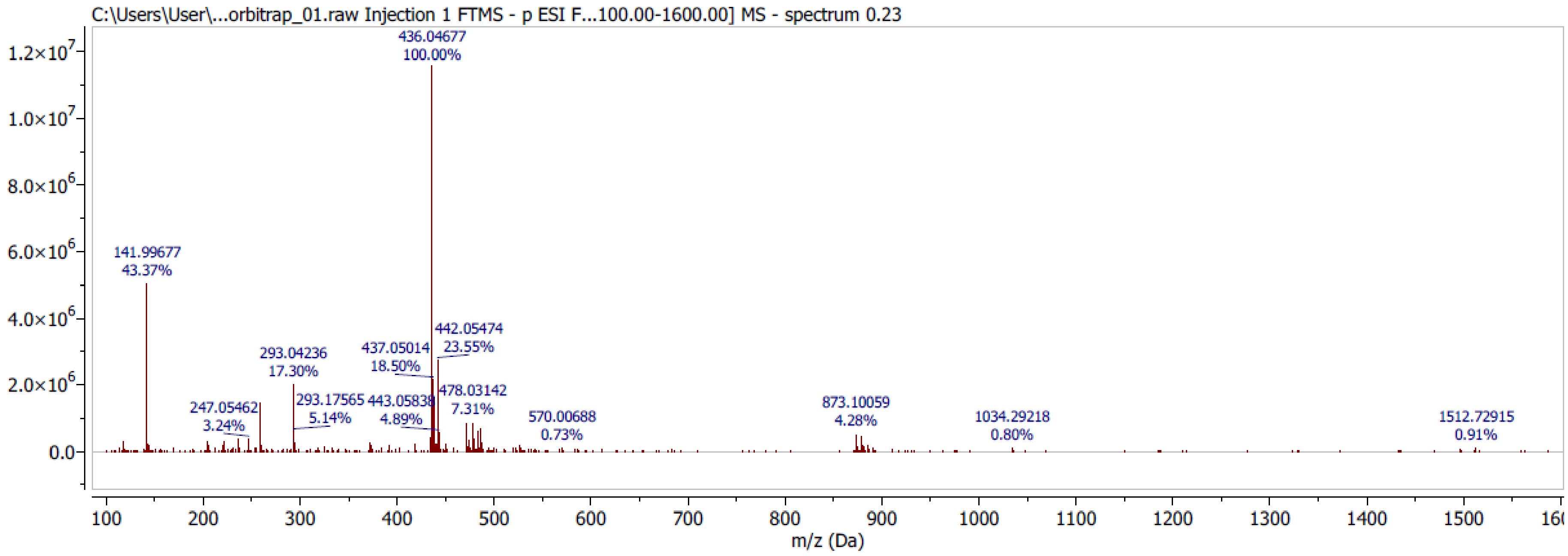

7. DFT Calculations

7.1 DFT coordinates of 32a

N(1)	1.363	0.860	0.147
N(2)	-1.259	1.459	0.535
H(3)	-1.843	1.242	1.338
N(4)	-2.029	-1.228	-0.069
O(5)	0.945	-1.661	-0.945
O(6)	-0.720	0.440	-1.859
O(7)	-0.107	-1.159	1.600
O(8)	-1.007	-2.692	2.998
S(9)	3.714	1.918	0.391
S(10)	-1.028	4.118	0.600
S(11)	-4.443	-1.974	-0.168
C(12)	2.234	-1.799	-0.748
C(13)	2.829	-3.032	-1.125
H(14)	2.172	-3.790	-1.541
C(15)	4.179	-3.276	-0.960
H(16)	4.587	-4.239	-1.251
C(17)	5.028	-2.293	-0.415
H(18)	6.086	-2.490	-0.277
C(19)	4.490	-1.073	-0.058
H(20)	5.136	-0.311	0.368
C(21)	3.112	-0.786	-0.219
C(22)	2.618	0.534	0.102
C(23)	2.278	2.953	0.914
H(24)	2.205	2.926	2.005
H(25)	2.436	3.978	0.582
C(26)	1.074	2.287	0.235
H(27)	0.979	2.661	-0.792
C(28)	-0.272	2.479	0.966
H(29)	-0.119	2.373	2.042
C(30)	-1.732	3.384	-0.931
H(31)	-2.588	3.981	-1.246
H(32)	-0.983	3.403	-1.727
C(33)	-2.143	1.939	-0.587
H(34)	-3.173	1.945	-0.220
C(35)	-1.992	0.991	-1.835
H(36)	-2.141	1.628	-2.722
C(37)	-3.114	-0.093	-1.946
C(38)	-3.100	-1.049	-0.794
C(39)	-3.452	-2.549	1.142
C(40)	-2.200	-2.024	1.037
C(41)	-1.025	-2.005	1.980
Ga(42)	-0.195	-0.304	-0.191
H(43)	-2.934	-0.645	-2.876
H(44)	-4.100	0.373	-2.035
H(45)	-3.853	-3.195	1.909

7.2 DFT coordinates of 32b

N(1)	1.244	1.319	-0.388
N(2)	-1.313	1.382	0.364
H(3)	-1.353	1.435	1.381
N(4)	-1.613	-1.547	0.078
O(5)	1.152	-1.227	-1.395
O(6)	-1.267	0.280	-1.947
O(7)	0.395	-2.520	2.838
S(8)	3.118	2.190	1.188
S(9)	-1.884	3.942	0.247
S(10)	-3.638	-3.019	0.440
C(11)	2.436	-1.236	-1.099

C(12)	3.210	-2.340	-1.537
H(13)	2.696	-3.112	-2.101
C(14)	4.559	-2.445	-1.250
H(15)	5.113	-3.310	-1.604
C(16)	5.217	-1.454	-0.500
H(17)	6.273	-1.544	-0.270
C(18)	4.496	-0.359	-0.065
H(19)	5.000	0.418	0.503
C(20)	3.122	-0.205	-0.364
C(21)	2.433	1.001	0.037
C(22)	1.431	2.936	1.394
H(23)	0.930	2.435	2.226
H(24)	1.535	4.000	1.606
C(25)	0.797	2.661	0.030
H(26)	1.203	3.392	-0.682
C(27)	-0.713	2.607	-0.198
H(28)	-0.801	2.485	-1.280
C(29)	-3.220	2.782	-0.338
H(30)	-4.108	2.955	0.271
H(31)	-3.458	3.019	-1.377
C(32)	-2.686	1.314	-0.196
H(33)	-3.311	0.782	0.522
C(34)	-2.591	0.493	-1.565
H(35)	-3.106	1.084	-2.337
C(36)	-3.372	-0.854	-1.480
C(37)	-2.811	-1.739	-0.409
C(38)	-2.272	-3.216	1.500
C(39)	-1.297	-2.327	1.164
C(40)	-0.024	-1.915	1.854
Ga(41)	-0.157	-0.183	-0.458
H(42)	-3.285	-1.348	-2.454
H(43)	-4.438	-0.682	-1.299
H(44)	-2.283	-3.934	2.306
O(45)	0.498	-0.847	1.321

7.3 DFT coordinates of 32c

N(1)	1.445	0.786	0.395
N(2)	-1.108	1.571	0.418
H(3)	-1.422	1.682	1.383
N(4)	-2.069	-1.182	-0.023
O(5)	0.910	-1.665	-0.827
O(6)	-0.712	0.415	-1.809
O(7)	-0.205	-1.096	1.713
O(8)	-1.183	-2.570	3.119
S(9)	3.838	1.751	0.685
S(10)	-0.892	4.182	0.294
S(11)	-4.464	-1.973	-0.223
C(12)	2.211	-1.814	-0.751
C(13)	2.757	-3.033	-1.232
H(14)	2.056	-3.768	-1.615
C(15)	4.114	-3.286	-1.215
H(16)	4.486	-4.236	-1.590
C(17)	5.019	-2.327	-0.723
H(18)	6.086	-2.525	-0.712
C(19)	4.526	-1.125	-0.258
H(20)	5.221	-0.379	0.116
C(21)	3.139	-0.834	-0.248
C(22)	2.688	0.449	0.244
C(23)	2.459	2.977	0.690
H(24)	2.627	3.703	1.483
H(25)	2.446	3.486	-0.277
C(26)	1.195	2.141	0.920
H(27)	0.970	2.065	1.992

C(28)	-0.048	2.559	0.138
H(29)	0.201	2.448	-0.920
C(30)	-2.227	3.424	-0.760
H(31)	-3.177	3.880	-0.483
H(32)	-2.017	3.658	-1.806
C(33)	-2.221	1.875	-0.513
H(34)	-3.154	1.594	-0.022
C(35)	-1.981	0.985	-1.813
H(36)	-2.091	1.645	-2.687
C(37)	-3.089	-0.103	-1.974
C(38)	-3.107	-1.041	-0.806
C(39)	-3.530	-2.501	1.146
C(40)	-2.283	-1.958	1.089
C(41)	-1.150	-1.917	2.079
Ga(42)	-0.201	-0.286	-0.098
H(43)	-2.870	-0.662	-2.892
H(44)	-4.076	0.354	-2.101
H(45)	-3.955	-3.140	1.906

7.4 DFT coordinates of 32d

N(1)	-1.336	-0.655	0.413
N(2)	1.164	-1.658	0.251
H(3)	1.970	-1.588	0.865
N(4)	2.265	0.947	-0.006
O(5)	-0.755	1.873	-0.671
O(6)	0.620	-0.115	-1.939
O(7)	0.422	1.028	1.741
O(8)	1.636	2.180	3.260
S(9)	-3.715	-1.279	1.235
S(10)	-0.093	-4.002	-0.228
S(11)	4.776	1.237	-0.143
C(12)	-2.057	1.977	-0.741
C(13)	-2.591	3.177	-1.283
H(14)	-1.879	3.931	-1.603
C(15)	-3.950	3.380	-1.416
H(16)	-4.312	4.310	-1.846
C(17)	-4.870	2.397	-1.005
H(18)	-5.937	2.553	-1.119
C(19)	-4.389	1.226	-0.455
H(20)	-5.092	0.458	-0.144
C(21)	-3.001	0.983	-0.296
C(22)	-2.565	-0.238	0.347
C(23)	-2.398	-2.541	1.495
H(24)	-2.507	-2.971	2.490
H(25)	-2.506	-3.320	0.739
C(26)	-1.088	-1.759	1.333
H(27)	-0.820	-1.298	2.294
C(28)	0.172	-2.568	0.906
H(29)	0.605	-2.991	1.814
C(30)	0.560	-3.074	-1.654
H(31)	0.990	-3.782	-2.364
H(32)	-0.237	-2.508	-2.140
C(33)	1.628	-2.123	-1.107
H(34)	2.578	-2.654	-0.996
C(35)	1.737	-0.920	-2.098
H(36)	1.732	-1.378	-3.102
C(37)	3.073	-0.112	-2.066
C(38)	3.264	0.664	-0.801
C(39)	3.952	1.818	1.276
C(40)	2.621	1.552	1.175
C(41)	1.494	1.632	2.170
Ga(42)	0.287	0.350	-0.131

H(43)	3.038	0.590	-2.908
H(44)	3.931	-0.770	-2.236
H(45)	4.491	2.276	2.092

7.5 DFT coordinates of 41

N(1)	-1.671	-0.321	0.316
N(2)	-0.148	-2.841	1.296
H(3)	-0.052	-3.388	2.145
N(4)	2.379	0.478	-0.648
O(5)	-0.386	1.706	-1.292
O(6)	0.439	-1.410	-1.026
O(7)	0.835	1.005	1.371
O(8)	2.424	2.133	2.474
S(9)	-4.265	-0.031	0.050
S(10)	-1.672	-4.468	-0.157
S(11)	4.792	-0.277	0.002
C(12)	-1.331	2.496	-0.775
C(13)	-1.297	3.870	-1.085
H(14)	-0.497	4.224	-1.726
C(15)	-2.251	4.745	-0.583
H(16)	-2.185	5.801	-0.826
C(17)	-3.289	4.279	0.236
H(18)	-4.026	4.964	0.639
C(19)	-3.369	2.923	0.515
H(20)	-4.176	2.547	1.136
C(21)	-2.408	2.014	0.026
C(22)	-2.610	0.575	0.202
C(23)	-3.679	-1.728	0.377
H(24)	-3.893	-1.971	1.421
H(25)	-4.209	-2.429	-0.268
C(26)	-2.160	-1.717	0.088
H(27)	-1.983	-1.904	-0.974
C(28)	-1.525	-2.903	0.891
H(29)	-2.127	-3.062	1.788
C(30)	0.050	-4.218	-0.774
H(31)	0.507	-5.200	-0.901
H(32)	0.018	-3.704	-1.735
C(33)	0.807	-3.360	0.277
H(34)	1.562	-3.958	0.797
C(35)	1.435	-2.070	-0.274
H(36)	1.638	-1.553	0.677
C(37)	2.763	-1.938	-1.048
C(38)	3.195	-0.523	-0.698
C(39)	4.375	1.461	0.429
H(40)	4.598	1.652	1.478
H(41)	4.972	2.126	-0.196
C(42)	2.857	1.624	0.143
C(43)	2.591	2.958	-0.574
H(44)	3.128	2.973	-1.526.
H(45)	2.945	3.785	0.047
H(46)	1.527	3.086	-0.773
C(47)	2.009	1.594	1.460
Ga(48)	0.227	0.256	-0.278
H(49)	2.590	-2.050	-2.122
H(50)	3.535	-2.645	-0.736

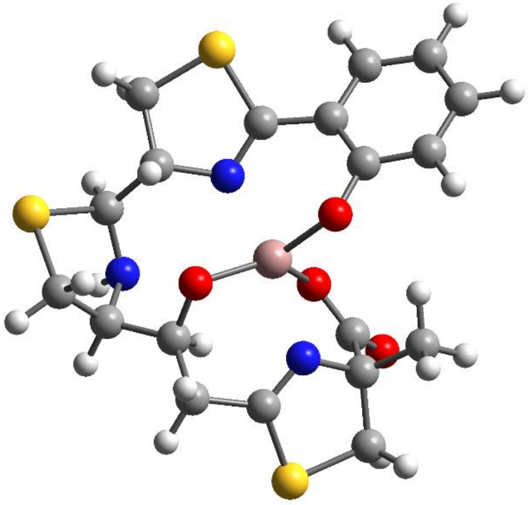

7.6 DFT-Model of the Ga^{3+} complex 41

7.7 DP4+ Analysis of 32a-d

Isomer 1: (*9R10R12R13S*)-**32a**
Isomer 2: (*9S10R12R13S*)-**32b**
Isomer 3: (*9S10S12R13S*)-**32c**
Isomer 4: (*9R10S12R13S*)-**32d**

Functional		Solvent?		Basis Set		Type of Data	
B3LYP		PCM		6-31G(d,p)		Unscaled Shifts	
		DP4+	99.90%	0.10%	0.00%	0.00%	-
Nuclei	sp2?	Experimental	Isomer 1	Isomer 2	Isomer 3	Isomer 4	Isomer 5
H	x	6.82	6.88	6.80	6.90	6.89	
H	x	7.38	7.57	7.49	7.60	7.57	
H	x	6.74	6.88	6.73	6.90	6.89	
H	x	7.48	7.57	7.49	7.48	7.51	
H		3.36	3.21	3.03	3.47	3.66	
H		3.68	3.50	3.61	3.47	3.30	
H		4.7	4.72	5.37	4.96	4.78	
H		4.84	4.72	5.18	4.60	5.23	
H		3.15	3.50	3.03	3.84	3.77	
H		3.59	3.39	3.50	3.47	3.45	
H		3.78	3.97	4.07	3.47	3.38	
H		4.45	4.64	4.48	4.45	4.40	
H		3.57	3.21	3.50	3.47	3.45	
H		3.36	3.50	3.03	3.15	3.05	
H	x	8.14	7.79	7.75	7.84	7.71	
C	x	167.65	168.36	167.47	168.06	167.46	
C	x	124.06	120.85	120.74	120.73	119.95	
C	x	137.17	135.33	135.47	135.14	133.90	
C	x	117.85	113.14	113.01	113.14	112.98	
C	x	133.12	132.34	132.66	133.04	130.41	
C	x	116.84	114.76	114.33	114.08	118.05	
C	x	180.72	183.54	184.12	183.81	191.30	
C		35.04	35.63	35.28	34.73	39.10	
C		77.18	75.42	77.16	73.52	70.05	
H		70.27	74.46	73.06	75.96	71.64	
C		38.89	41.11	40.54	47.59	48.14	
C		70.29	70.37	74.53	58.66	58.71	
C		72.51	74.38	72.88	78.69	79.41	
C		38.93	37.30	36.24	37.78	38.66	
C	x	173.02	181.19	180.78	182.13	180.51	
C	x	123.95	126.42	126.44	126.39	125.93	
C	x	147.46	145.56	145.38	146.00	145.04	
C	x	164.55	159.27	159.38	159.98	158.21	

Functional	Solvent?	Basis Set	Type of Data
B3LYP	PCM	6-31G(d,p)	Unscaled Shifts

	Isomer 1	Isomer 2	Isomer 3	Isomer 4	Isomer 5	Isomer 6
sDP4+ (H data)	74.52%	7.93%	17.53%	0.01%	-	-
sDP4+ (C data)	86.02%	13.98%	0.00%	0.00%	-	-
sDP4+ (all data)	98.30%	1.70%	0.00%	0.00%	-	-
uDP4+ (H data)	87.56%	6.44%	2.97%	3.03%	-	-
uDP4+ (C data)	54.87%	45.13%	0.00%	0.00%	-	-
uDP4+ (all data)	94.30%	5.70%	0.00%	0.00%	-	-
DP4+ (H data)	98.44%	0.77%	0.79%	0.00%	-	-
DP4+ (C data)	88.21%	11.79%	0.00%	0.00%	-	-
DP4+ (all data)	99.90%	0.10%	0.00%	0.00%	-	-
